# Adaptation of Fusarium Head Blight Pathogens to Changes in Agricultural Practices and Human Migration

**DOI:** 10.1002/advs.202401899

**Published:** 2024-08-05

**Authors:** Meixin Yang, Sandra Smit, Dick de Ridder, Jie Feng, Taiguo Liu, Jinrong Xu, Theo A. J. van der Lee, Hao Zhang, Wanquan Chen

**Affiliations:** ^1^ State Key Laboratory for Biology of Plant Disease and Insect Pests, Institute of Plant Protection Chinese Academy of Agricultural Sciences Beijing 100193 P. R. China; ^2^ Bioinformatics Group Wageningen University & Research Droevendaalsesteeg 1 Wageningen PB 6708 The Netherlands; ^3^ National Agricultural Experimental Station for Plant Protection, Gangu Ministry of Agriculture and Rural Affairs Tianshui 741200 P. R. China; ^4^ Department of Botany and Plant Pathology Purdue University West Lafayette IN 47907 USA; ^5^ Biointeractions and Plant Health Wageningen Plant Research Droevendaalsesteeg 1 Wageningen PB 6708 The Netherlands

**Keywords:** evolution trajectories, *Fusarium asiaticum*, Fusarium head blight, human migration, long‐distance dispersal

## Abstract

Fusarium head blight (FHB) is one of the most destructive wheat diseases worldwide. To understand the impact of human migration and changes in agricultural practices on crop pathogens, here population genomic analysis with 245 representative strains from a collection of 4,427 field isolates of *Fusarium asiaticum*, the causal agent of FHB in Southern China is conducted. Three populations with distinct evolution trajectories are identifies over the last 10,000 years that can be correlated with historically documented changes in agricultural practices due to human migration caused by the Southern Expeditions during the Jin Dynasty. The gradual decrease of 3ADON‐producing isolates from north to south along with the population structure and spore dispersal patterns shows the long‐distance (>250 km) dispersal of *F. asiaticum*. These insights into population dynamics and evolutionary history of FHB pathogens are corroborated by a genome‐wide analysis with strains originating from Japan, South America, and the USA, confirming the adaptation of FHB pathogens to cropping systems and human migration.

## Graphical abstract

Genomic analysis of 245 strains from 4,427 *Fusarium asiaticum* isolates reveals that its evolution over the last 10,000 years correlates with agricultural changes due to human migration during the Jin Dynasty's Southern Expeditions. By combining the chemotype composition, population structure, and spore dispersal patterns of *F. asiaticum*, the long‐distance (>250 km) dispersal of *Fusarium* is provide.

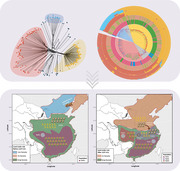

## 1 Introduction

Fusarium head blight (FHB) is an economically important disease on wheat, barley and oat, caused by *Fusarium* species.^[^ ^1^, ^2^, ^3^, ^4^ ^]^ FHB can result in yield losses ranging from 15% to 70% and infection reduces the quality of grains.^[^ ^5^ ^]^ In addition, infected grains accumulate mycotoxins, such as trichothecenes and zearalenone.^[^ ^6^, ^7^ ^]^ Mycotoxin‐contaminated produce poses a serious threat to human and livestock health^[^ ^8^ ^]^ and maximum levels of contamination are enforced globally to secure food safety.^[^ ^9^, ^10^, ^11^, ^12^ ^]^ FHB is caused by members of the *Fusarium graminearum* species complex (FGSC) and poses a threat in all wheat production regions around the globe, including China. FHB is the most important wheat disease in southern China, primarily caused by the FGSC species *F. asiaticum*. This species has also been identified as the main driver of FHB in other eastern Asian regions, including Japan and Korea.^[^ ^13^, ^14^, ^15^ ^]^ This species was also found in Iran, Nepal, and recently in North America, Brazil, and Uruguay.^[^ ^16^, ^17^, ^18^, ^19^ ^]^ *F. asiaticum* is genetically/molecularly classified based on the ability to produce different trichothecenes: deoxynivalenol (DON), 3‐acetyl‐deoxynivalenol (3ADON), 15‐acetyl‐deoxynivalenol (15ADON), or nivalenol (NIV).^[^ ^20^, ^21^ ^]^

Changes in agricultural practices, such as the introduction of new crop species or crop rotation schemes, are often accompanied by the emergence of associated pathogens and/or substantial transformations in the pathogen populations previously present on the wild ancestors of cultivated crops.^[^ ^22^ ^]^ The distribution of appropriate host species significantly contributes to the expansion of a pathogen's spread.^[^ ^23^ ^]^ Pathogens and their hosts frequently exhibit common evolutionary trajectories as a result of co‐evolution.^[^ ^24^ ^]^ Several recent studies have reported the preference of FHB pathogens for specific hosts and environments. For instance, *F. graminearum* exhibited higher prevalence in rotation systems involving wheat and maize, while *F. asiaticum* was observed to dominate in regions characterized by the rotation of rice and wheat, such as China, Korea and the USA.^[^ ^13^, ^16^, ^25^ ^]^ While these findings clearly indicate there is a relation between FHB pathogens, hosts and cropping systems, it does not provide information on the drivers and mechanisms.

The long‐distance dispersal (LDD) of plant pathogens plays a crucial role in the epidemiology and population structure. The LDD of plant pathogenic fungi is mediated by various factors, including human activities, host plant dispersal, and meteorological phenomena.^[^ ^26^ ^]^ Because of their strong host specificity, obligate biotrophic fungi are compelled to move to different places along with their hosts or alternate hosts to complete their life cycle. This characteristic can be exploited to trace the pathogen and measure the fusion of different populations using molecular methods. Therefore, large‐scale research on LDD has been focused on obligate biotrophic plant‐pathogenic fungi. For instance, Alexandros et al. (2022) showed the origin of *Blumeria graminis tritici* and the association of pathogen spread with historical human migration and trade based on 172 global genomic analyses.^[^ ^27^ ^]^ Alternatively, wind‐mediated long‐distance dispersal of biotrophic phytopathogenic fungal spores has the potential to disseminate plant disease across and even between continents, allowing the reemergence of diseases in regions where host plants are temporarily absent.^[^ ^28^ ^]^ Several studies have demonstrated that spores of airborne biotrophic pathogens can travel over thousands of kilometers, thus facilitating the spread of diseases, including rust, powdery mildew, and downy mildew diseases.^[^ ^29^ ^]^ However, non‐biotrophic fungi typically have a wide range of hosts and can survive on various plant debris in their necrotrophic stage, complicating the ability to trace their spread trajectory. Therefore, the LDD of non‐biotrophic fungi remains a subject of controversy. It is widely recognized that the prevalence of FHB is primarily attributed to local sources of *Fusarium*,^[^ ^5^ ^]^ considering the limited infection period of non‐biotrophic FHB pathogens. Several studies investigated the LDD of FHB pathogens focusing on catching *Fusarium* spores in the air during the infection period.^[^ ^30^, ^31^, ^32^ ^]^ To date, there is no study reporting the influence and epidemiological importance of LDD for the evolution of FHB pathogens.

Here, a novel collection of 245 high‐quality *F. asiaticum* genomes was generated and we applied integrated population‐genomic and pangenomic approaches to study the recent and historic dynamics within the *F. asiaticum* population in southern China. We uncovered the population structure, its diversity, and the drivers and mechanisms of this diversity and selection. We assessed the historic impact of human migration and cropping systems on the spread and LDD of *F. asiaticum*, providing new insights that help in the management of FHB in wheat. Finally, our analysis on a global panel of *F. asiaticum* corroborates the uncovered evolutionary trajectory and migration patterns identified in China and the suggested management strategies.

## 2 Results

### 2.1 A Collection of High‐Quality Genome Sequences and Annotation of 245 *F. asiaticum* Field Isolates

**Figure 1:**
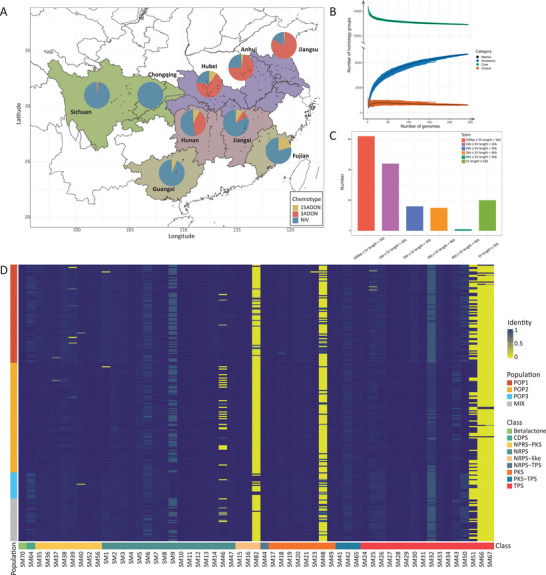
A) Map of China indicating the 189 sampling collection sites, and three chemotype distribution (15ADON, 3ADON, and NIV) of *F. asiaticum* in eight provinces and one direct‐administered municipality. Different colors in the map indicated four ecological regions, including 1) Hubei, Anhui, and Jiangsu, which are part of the MLRYR, were plain regions with wheat‐rice rotation system; 2) Sichuan and Chongqing are mountainous regions where rice predominates, but they also have high crop diversity; 3) Guangxi and Fujian are mountainous regions with double cropping of rice is cultivated; 4) Hunan and Jiangxi are transition regions between plains and mountains where double cropping of rice is practiced. B) Changes in the number of core, accessory, and unique groups upon the increase of genome analyzed. C) Numbers of different length intervals of large structural variations (≥ 500 bp) detected basing graph‐based pangenome of *F. asiaticum*. D) Secondary metabolite gene cluster pangenome of the *F. asiaticum* according to the evidence for backbone genes. The colored bars positioned beneath the heatmap correspond to the cluster type. The colored bars positioned on the left side of the heatmap correspond to the population. CDPS, Cyclodipeptide synthases; NRPS, nonribosomal peptide synthetase; PKS, polyketide synthase; TPS, terpene synthase.

To characterize the genetic diversity of *F. asiaticum* populations in Southern China, we obtained 4427 isolates from rice stubble or wheat heads from four ecological regions, covering eight provinces and one direct‐administered municipality, and 189 sampling sites (**Figure** **1**A, Table S1, Supporting Information). Multilocus genotyping was performed to confirm the *F. asiaticum* species identity and molecular chemotype identification was used to group the isolates into three distinct chemotypes: 3ADON, 15ADON, and NIV. Out of the total 4427 isolates, there were 2188 NIV producers, 1908 3ADON producers, and 331 15ADON producers. NIV isolates dominated in rice dominated regions, while 3ADON isolates predominated in regions with wheat‐rice rotation. The chemotype composition in each province was summarized in Figure 1A. Based on strain chemotype, geographic origin, and host plant, 245 representative strains were selected for further genomic analysis. A summary of the metadata of these isolates is shown in Table S1 (Supporting Information).

A chromosome‐level high‐quality reference genome assembly of strain 180197 (3ADON_MLRYR_Wheat) was generated by PacBio and Illumina sequencing. The genome assembly of 38.42 Mb consisted of four contigs representing the four chromosomes, with only a single gap(≈10 Kb) in chromosome II. The genome has a GC content of 48.2% with specific patches of low GC content at the telomeres and centromeres. The genome structure of *F. asiaticum* is very similar to that of sister species *F. graminearum* (reference genome PH‐1), as evidenced by a similar length (37.95 Mb for PH‐1), high average nucleotide identity (ANI) score of 97.47%, and extensive macrosynteny in a whole‐genome alignment of the two genomes, with variations primarily observed in the sub‐telomeric and some centromeric regions of the chromosomes (Figure S1, Supporting Information). The additional 244 assembled genomes were generated from Illumina sequencing data and their genome sizes ranged from 36.42 to 38.61 Mb, with an average GC content between 47.9% and 48.3%. The N50 values for the 244 additional strains ranged between 0.7 and 2.6 Mb, indicating that these assembled genomes were more fragmented but still of high quality.

All assemblies were annotated with BRAKER2 using an RNA‐seq data set from one *F. asiaticum* isolate as expression evidence and conserved ortholog proteins in Fungi Odb10 as protein evidence, generating 13826 to 14418 predicted protein‐coding genes per genome. The “benchmarking universal single‐copy orthologs” (BUSCO) method using the sordariomycetes_odb10 data set showed 99.3% to 99.7% completeness of the 3817 single‐copy orthologs. Notably, all 245 strains missed a set of three BUSCO genes, specifically 92304at147550, 83794at147550, and 110555at147550, suggesting these genes are likely not universal in the *Fusarium* genus.

Both the assemblies and annotations are highly consistent with the underlying data, they show high completeness, and they are similar to the high‐quality reference genome of *F. graminearum*. Using a single pipeline to annotate all assemblies ensures consistent annotation, making the data suitable for pangenomics.

### 2.2 Capturing Genetic Diversity of *F. asiaticum*

To capture the genetic diversity in *F. asiaticum*, we used 245 high‐quality genomes as input for PanTools and constructed a pangenome with 17769 homology groups (Table S2, Supporting Information), revealing a high proportion of core genes (84.79%), accessory genes (15.18%), and only few unique genes (0.02%) on average per genome. These genes were functionally annotated using various databases (**Table** **1**). Changes in the counts of core (shared by all), accessory (shared by some), and unique groups across various genome combinations indicated that the pangenome we constructed is closed, which means that the available diversity is largely covered by the selected 245 *F. asiaticum* genomes (Figure 1B).

**Table 1:** Number of diverse categories of functional annotations in core, accessory, and unique homology groups.

	Number	GO^a)^	SignalP	TF^b)^	Interpro	Pfam	COG^c)^	Secreted proteins
Core	11173	6536	1257	724	8961	8327	8871	1109
Accessory	5845	1523	411	166	2280	1954	2441	357
Unique	751	121	28	9	184	162	206	3
Total	17769	8180	1696	899	11425	10443	11518	1469

^a)^ Gene ontology;

^b)^ Transcription factor;

^c)^ Clusters of orthologous groups.

Single‐nucleotide polymorphisms (SNPs) and structural variations (SVs) were assessed in the pangenome. Firstly, we separately identified SNPs in core genes and in the full genomes, to compare diversity between these genomic regions. A comparison of phylogenies constructed using the two distinct types of SNPs demonstrated a high similarity (nNS = 0.301; nMC = 0.297), highlighting their robustness (Text S1, Supporting Information). Subsequently, a survey revealed SVs of various sizes (Figure 1C), including large SVs unique to specific genomes, in some cases indicative of horizontal gene transfer and/or ancient variation predating speciation (Text S2, Supporting Information).

A gene ontology (GO) analysis of all accessory groups revealed significant enrichment of 142 GO terms (p < 0.05). Among these genes, 10 were found under the cellular components category, 45 under molecular functions and 87 under biological processes (Table S3, Supporting Information). For instance, ABC transporters (GO:0008559) play important and diverse roles in both fungicide resistance and pathogenesis of *F. graminearum*.^[^ ^33^ ^]^ Azole transmembrane transport (GO:0015244) plays a crucial role in the sensitivity to azole antifungals.^[^ ^34^ ^]^ These accessory genes are likely involved in growth, development, and response to the environment of the pathogen, revealing its adaptive evolution. Subsequently, the functional diversity of *F. asiaticum* was assessed by testing for association between accessory genes and phenotypic traits, such as virulence on coleoptiles or mycotoxin production. Thirty candidate secreted proteins were identified (Table S4, Supporting Information), showing a significant presence/absence difference with coleoptile infection (P < 0.0001). We also identified distinct mycotoxin chemotypes (DON, 3ADON, 15ADON, and NIV) and found significant associations between the presence/absence of accessory genes and toxin production. Different transcription factors were involved in each chemotype (Text S3, Supporting Information). Following that, the genetic diversity of secondary metabolite (SM) gene clusters within the *F. asiaticum* pangenome was investigated. A total of 10914 SM clusters were predicted, showing diversity among 57 types and 9 classes (Figure 1D). Nonribosomal peptide synthetases (NRPS) and terpene cyclases (TPS) were the most common classes, while certain SM genes exhibited variations in presence/absence. In addition, differences in backbone gene length were observed, including the identification of four SM genes synthesizing the same compound with varying diversity among *F. asiaticum* genomes (Text S4, Supporting Information).

Both the analyses of SNPs and SVs, as well as the studies of accessory genes and SMs, indicated that there is an abundant and extensive genetic diversity within the *F. asiaticum* pangenome. The fact that the pangenome is closed and the phylogeny is stable provides a robust foundation for conducting population genomic analyses.

### 2.3 Identification of Three Distinct Populations and Strong Selection Pressure

**Figure 2:**
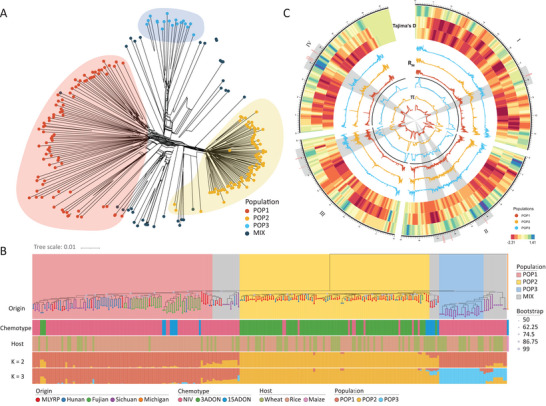
Population genetic structure of 245 *F. asiaticum*. A) Neighbor‐net phylogenetic network constructed with SplitsTree based on SNPs identified by reference‐based mapping of whole genome sequences to 180197. The three main groups and fuzzy strains which did not cluster with the main groups, generated by ADMIXTURE (K = 3), were color coded as indicated. B) A maximum likehood phylogeny, inferred from SNPs identified by reference‐based mapping of whole genome sequences to 180197, associated with samples‐collection information, including origin, chemotype, host and bar plots of individual ADMIXTURE membership coefficients at critical K = 2 and K = 3. PH‐1 *F. graminearum* as an outgroup. C) Selective sweep analyses of three populations. Sliding‐window values of intra‐population diversity and recombination (Tajima's D, Rm, and π) was calculated in 10 kb windows to identify genomic regions with signatures of selection in POP1, POP2, and POP3 populations of *F. asiaticum*.

After variant calling and quality control, a total of 516591 quality‐filtered nuclear SNPs and 35374 small indels were identified across the 245 *F. asiaticum* genomes. Both SNPs and indels were mainly found in upstream, downstream, and intergenic regions (Table S5, Supporting Information). Combination analyses of the patterns of genetic clustering and kinship analyses through parsimony informative SNPs delineated three distinct populations (POP1, POP2, and POP3, **Figure** **2**A, Text S5, Supporting Information).

A maximum likelihood phylogeny was constructed using the SNPs, and the three distinct population isolates, chemotype, geographic origin, and genotype information were highlighted in the tree. We found that the branches of the phylogenetic tree correspond strongly to the three populations (Figure 2A). POP1 is predominantly composed of NIV strains (79/87) and also shows a number of associated genotypes referred to as POP1 *sensu lato* , POP2 is more strictly delimited and dominated by 3ADON genotypes (85/97), and POP3 consists solely of NIV strains (23/23), revealing genetic differentiation and specific regional distributions (Figure 2B, Text S5, Supporting Information). Population structure analysis showed that the NIV strains exhibit a higher level of genetic variability compared with 3ADON strains. Notably, based on the phylogeny, 3ADON strains collected from the Middle and Lower Reaches of the Yangtze River (MLRYR) and Hunan were grouped in POP2. POP3 only contained strains originating from Sichuan (Figure 2B). To investigate genome‐wide genetic diversity, 10 kb non‐overlapping sliding windows were analyzed, revealing distinct patterns of nucleotide variation among POP1, POP2, and POP3. All three groups exhibit negative average Tajima's D values, indicating an excess of rare alleles in these populations. This pattern may be attributed to selective sweeps or population expansions. Moreover, nucleotide polymorphism (θ), average nucleotide diversity (π) and haplotype diversity (h) revealed reduced genetic variation in POP2 (**Table** **2**). These genetic patterns indicate that POP2 experienced a strong selective sweep and among the three populations appears to be the most stable/fixed population. In contrast, the genomic diversity of POP1 was much higher, with the highest average θ, π, and h, and the lowest Tajima's D, indicating it has high evolutionary potential. This is particularly the case if also the POP1 *sensu lato* isolates are included. POP3 showed slightly lower levels of diversity than POP1; values of Tajima's D were still negative but relatively high. The GO enrichment of population accessory genes in the three populations showed that these accessory genes were significantly enriched in 27 GO terms, including 12 terms for POP1, 10 terms for POP2, and 5 terms for POP3 (Figure S2, Supporting Information).

**Table 2:** Average genomic diversity within *F. asiaticum* populations.

	POP1	POP2	POP3
Tajima’ D	−1.2802	−1.1361	−0.2964
θ (nucleotide polymorphism)	0.00120	0.00060	0.00093
π (Nucleotide diversity)	0.00176	0.00084	0.00093
h (Haplotype diversity)	0.81552	0.62477	0.66516

To identify genes that are possible targets of selection, we performed pairwise comparisons between the three main populations and identified genomic regions contributing to differentiation. A linkage disequilibrium‐based approach using hapFlk and a site frequency spectrum‐based method combining Tajima's D and Fst values (Figure S3, Supporting Information) identified 18 overlapping regions containing 61 genes. To further delineate the regions under selection for each population at the genomic level, π, recombination events (Rm), Tajima's D values of each sliding‐window of intrapopulation and the candidate selection regions with genes were visualized on the chromosomes of 180197 (Figure 2C).

The combined results showed that regardless of the population, the distribution of SNPs across the genome varied widely and the SNP density was especially high in sub‐telomeric regions and some specific regions in the middle of chromosomes. Candidate selective regions similarly correspond to sub‐telomeric and central regions of the four chromosomes. Among the 61 genes identified, POP1 had 6, POP3 had 14, and POP2 had the most with 53; 6 genes were shared among all three populations. This indicates that POP2 was under stronger selection than POP1 and POP3.

### 2.4 POP2 (3ADON) Recently Diverged from POP3 (NIV)

**Figure 3:**
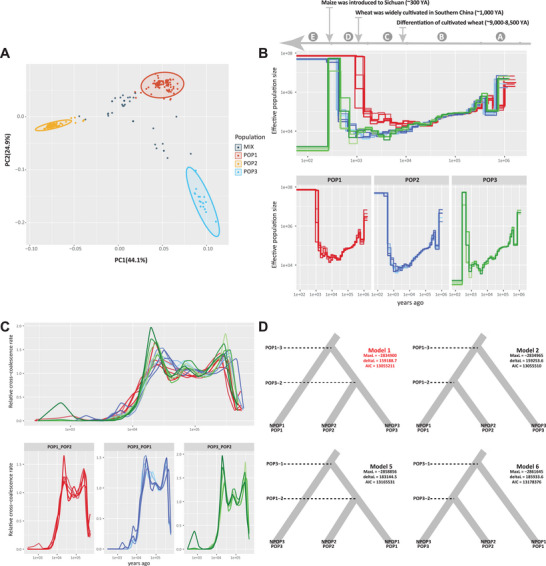
Effective population sizes and population split timing of three populations of *F. asiaticum*. A) PCoA inferred from SNPs showed a high correlation with three main populations. The PCoA was generated based on SNPs identified by mapping whole genome sequences to the reference genome 180197. B) Effective Population size diagram over time using the same mutation rate and eight of the most representative isolates in each population. The arrows indicated the crop evolutionary history in the matching period. C) Cross‐coalescence analysis over time using the same dataset as in A. D) Results of splits events inference with model schemes by fastsimcoal2. The four best models were illustrated (others were in Figure S3, Supporting Information). MaxL, deltaL and AIC values are shown below the model number in bold letters, respectively.

Next, we studied the diversification history of *F. asiaticum* populations. The ancestral population sizes and time of separation of the three populations of *F. asiaticum* were calculated using MSMC2. Representative strains were selected randomly based on the results of a principal coordinate analysis (PCoA, **Figure** **3**A). In the coalescence analysis, the three populations had distinct patterns of change in effective population size (Ne) (Figure 3B). During the earliest period (Period A), a drop in Ne indicates a short period of a genetic bottleneck. In period B, all three populations had similar Ne trajectories until ≈10000 years ago (YA). Thereafter in period C, POP1 first experienced a rise in Ne around 8000 YA, followed by a steep increase in Ne for all three populations in period D. In period E finally, the Ne of POP3 showed a rapid decrease around 250 YA. Cross‐coalescence analysis indicated different pairwise divergence between populations, although patterns of differentiation over time were similar (Figure 3C). POP1 diverged from the other populations slightly earlier than POP3 did from POP2, while POP2 and POP3 diverged rapidly.

Expanding upon previous analyses, our findings suggest that, in comparison to PO1 and POP3, POP2 exhibits lower genetic diversity, increased pathogenicity, increased host specificity. Therefore, POP2 cannot be the ancestorial pool in the demographic model test. Therefore, in total, eight demographic models were tested for possible gene flow among POP1, POP2 and POP3, using fastsimcoal2 and the site frequency spectrum for the three populations (Figure S4, Supporting Information). The most probable model (Model 1 in Figure 3D) proposes that POP2 and POP3 experienced gene flow from POP1, and POP2 diverged from POP3 thereafter. The results of TreeMix further supported the recent migrations from POP3 toward POP2 (Figure S5, Supporting Information).

In combination with the chemotype distribution information, our demographic model analyses indicated that the NIV population (POP1 and POP3) was older than the 3ADON population (POP2), consistent with the results of coalescent analysis.

### 2.5 High‐Resolution Method for Population Assignment of Worldwide Strains

In order to classify *Fusarium* strains from other countries, we used a k‐mer distance‐based phylogenetic tree to assign these strains to the different populations. The phylogeny was found to be highly consistent with the population structure analysis using ADMIXTURE (Figure S6, Supporting Information). The selected global diversity panel could be classified within the diversity found in China. Strains derived from North and South America grouped with the heterogenous population predominantly characterized by POP1 and the strains on this branch (POP1 *sensu lato*). In Japanese strains members of both POP1 and POP2 were identified. The Japanese strain that was identified as belonging to POP2 showed the 3ADON chemotype, matching the predominant chemotype of POP2 of Chinese strains. The global panel members that grouped with the heterogeneous population predominantly characterized by POP1 also exhibited the NIV chemotype associated with POP1. Strains from the United States and Brazil showed relatively low diversity and were genetically distinct from each other, indicating distinct and singular introduction of *F. asiaticum* in Brazil and the USA.

### 2.6 LongDistance Dispersal of *F. asiaticum* Populations and Its Importance for the Population Structure

**Figure 4:**
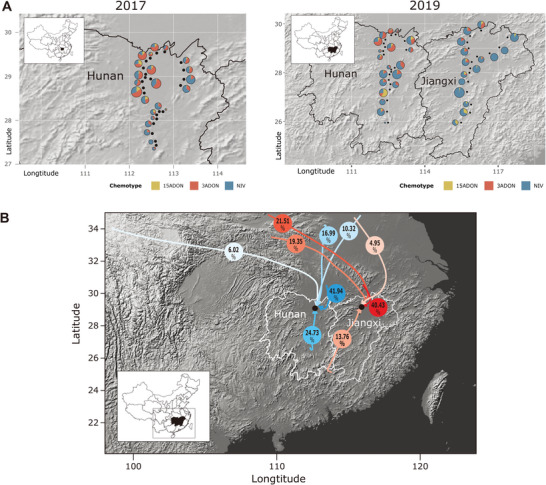
Long‐distance dispersal of *F. asiaticum* verification analyses. A) Pie charts illustrating the composition of trichothecenes chemotypes in *F. asiaticum* populations from Hunan and Jiangxi. The left panel displays the pie charts representing sampling sites in Hunan in 2017, while the right panel displays the pie charts representing sampling sites in Hunan and Jiangxi in 2019. B) Trajectory analyses were conducted to examine the dissemination routes of *F. asiaticum* to Hunan and Jiangxi on the topographic map. The left represents the air trajectory in Hunan during the simulation period from May 1st to 31st, 2017, at an altitude of 500 m. Similarly, the right represents the air trajectory in Jiangxi during the simulation period from March 1st to 31st, 2019, at an altitude of 500 m. Additional simulation results can be found in Figure S7 (Supporting Information).

Large‐scale sampling was conducted in 2017 and 2019, including diverse crop rotation systems and geographical regions. These included plains featuring rice‐wheat rotations (MLRYR), transitional regions between plains and mountains characterized by only rice cultivation (Hunan and Jiangxi), as well as mountainous areas where only rice cultivation prevailed (Guangxi and Fujian). Therefore, we utilized data from these two pivotal years for LDD analyses. Trichothecene identification tests showed that in regions with annual wheat‐rice crop rotation, such as MLRYR, the dominant chemotype was 3ADON. In the Guangxi and Fujian provinces, where rice cultivation is dominant and no wheat grown, the chemotype composition was similar (P = 0.5178) and no 3ADON producer was detected (**Table** **3**). In contrast, in the Hunan and Jiangxi provinces, where rice cultivation is dominant as well, we detected an unexpectedly high percentage of 3ADON producers, especially in Hunan, which is significantly different from other non‐wheat growing regions. For example, 42% of the 708 *F. asiaticum* isolates collected from Hunan in 2017, 34% of the 374 isolates from Hunan in 2019, and 8% of the 366 isolates from Jiangxi in 2019 were 3ADON producers. As visualized by the pie charts of chemotypes of *F. asiaticum* from each sampling site in Hunan in 2017 (**Figure** **4**A, left) and in Hunan and Jiangxi in 2019 (Figure 4A, right), the proportion of 3ADON of *F. asiaticum* decreased from north to south.

**Table 3:** Trichothecene type compositions in provinces with different cropping systems.

	Rotation	Year	NIV	3ADON	15ADON	P‐value^a)^
MLRYR	Wheat‐Rice	2019	69	407	34	< 2.2e^−16^
Hunan	Rice	2017	352	297	59	< 2.2e^−16^
Hunan		2019	210	127	32	< 2.2e^−16^
Jiangxi	Rice	2019	301	30	33	3.713e^−07^
Guangxi	Rice	2017	86	0	6	0.5178
Fujian	Rice	2017	232	0	24	–
Total			1255	861	188	–

^a)^ Fisher's exact test of comparison with Fujian population.

To identify the source populations of 3ADON in non‐wheat growing regions in Hunan and Jiangxi, an airflow trajectory analysis was conducted. At the sampling sites both in Hunan and Jiangxi at 60 m and 500 m above ground, during March to May in 2017 and 2019, four or five upper airflow trajectories remained after dimensionality reduction (Figure 4B; Figure S7, Supporting Information). The results indicate that from March to April, the period in which ascospores that initiate infections are released from plant debris, and in May, when numerous conidia are generated after infecting the wheat heads, the prevailing trajectories (accounting for an average 75.34% of (σ ± 0.17) of the trajectories) showed a strong directionality from the northeast or from MLRYR to Hunan and Jiangxi. In addition, there was no significant difference (p‐value = 0.3174) between the average trajectories at 60 m (75.95%, σ ± 0.14) and 500 m (74.73%, σ ± 0.20) from MLRYR to Hunan/Jiangxi, which indicates that the mountains and ridges presented only little barrier for these wind currents. The phylogeny (Figure 2B) demonstrates that POP2 samples in Hunan and MLRYR represent the same population, with intertwined genotypes originating from the different regions. These results are consistent with long‐distance (>250 km) spore migration of *F. asiaticum* from MLRYR to Hunan and Jiangxi.

## 3 Discussion

FHB is a global constraint in wheat production and occurs widely in China, especially in the MLRYR where FHB epidemics have been common in the last decades. *F. asiaticum* is the dominant causal species in these regions. To develop disease control strategies, it is important to clarify the genetic diversity, population composition and migration patterns. This study provides the first detailed report on the pangenomic composition of 245 *F. asiaticum* strains, and not only implicates specific genes associated with important traits such as virulence and toxin production, but also revealed the evolution of the species. Secondly, population genomic analyses clarified the composition and diversity of different *F. asiaticum* subpopulations and revealed the effects of crop rotation systems and historical human activities on FHB prevalence and population structure. Finally, we found evidence that *Fusarium* could spread over long distances (>250 km).

Although many *Fusarium* genomes have been sequenced,^[^ ^35^, ^36^ ^]^ most comparative genome studies have been performed using a single reference per species. Only one report used 20 *F. graminearum* strains to construct a pangenome and investigate the intra‐species diversity.^[^ ^37^ ^]^ Our study is the first pangenome analysis on *Fusarium* based on hundreds of high‐quality genomes.

The *F. asiaticum* pangenome presented here contains 17769 homology groups. When split into core, accessory and unique genes, the core group was found to be the largest, representing 63% of the homology groups. Heaps’ law revealed an alpha value of 1.038, suggesting that the *F. asiaticum* pangenome can be regarded as closed and the inclusion of new genomes is unlikely to change the gene set significantly. The 245 *F. asiaticum* genomes showed only 751 unique genes, i.e., around 3 unique genes per genome. Based on the predicted functional domains and high homology to previously characterized genes, it appears that these sequences may indeed contain uniquely present functional genes.

Although overall the genome composition is thus relatively consistent among *F. asiaticum* genomes, we still found a high number of accessory genes.^[^ ^5^, ^8^, ^45^ ^]^ These genes were significantly enriched for GO terms related to DNA biosynthesis, metabolism, and repair. This may be associated with adaptive evolution in different environments. For instance, survival in environments with UV radiation and extreme temperatures is critical for pathogens as these conditions cause DNA damage. Enhanced DNA metabolism and repair capabilities help improve the adaptability of pathogen populations to their environments.^[^ ^38^ ^]^ Additionally, mycotoxin chemotypes are among the significant variations between populations. We also found GO terms related to secondary metabolic pathways enriched in POP2, indicating that, besides mycotoxin metabolism, other metabolic processes may have diverged between populations, which needs further research. In addition, this diversity of gene presence/absence may be associated with phenotypic variation that we found in terms of pathogenicity and toxin production. Below we demonstrate that the prime candidate genes identified in our analysis are likely to be bona fide, as functional studies showed that close homologues were implicated in these processes. The production of secretory proteins (e.g., the secretome) is a strategy to manipulate the host during infection and differences in pathogenicity may arise from the secretory proteins within the accessory genome. Among the accessory genes associated with pathogenicity we identified a gene predicted to encode an amidohydrolase family protein, that was previously shown to play an important role in the biosynthesis of virulence factors such as Fusaoctaxin B upon infection of *F. graminearum* on wheat.^[^ ^39^ ^]^ We hypothesize that this gene could similarly contribute to virulence in *F. asiaticum* infection on wheat. Another gene significantly linked to virulence encoded an acetylxylan esterase; a homologue of this gene was shown to be required for virulence of necrotrophic fungal pathogens.^[^ ^40^ ^]^ A third accessory gene significantly linked to virulence encoded a protein with homology to the metallo‐beta‐lactamase superfamily that contributes to antibiotic resistance. Indeed, soil‐associated fungi typically have more endocannabinoid‐encoding genes than those from environments with lower microbial diversity because of the high antibiotic diversity in the soil environment.^[^ ^41^, ^42^, ^43^ ^]^ This gene was commonly detected in strains from POP1, and POP3, the NIV producers: of the 54 strains in which this gene was detected, 22 fell in POP1, 18 in POP3, and the remaining in the MIX category. This may explain why NIV producers were dominant on saprophytic hosts. Finally, a gene putatively encodes killer toxin subunits alpha/beta, components of secreted killer toxin related to pathogenicity.^[^ ^44^ ^]^ All 46 strains containing this putative killer toxin were NIV producers, among which 33 are in POP1 and others in MIX. Overall, the above‐described candidate genes were mainly found in POP1 or POP3 (NIV), consistent with the conclusions obtained from the population diversity analysis: POP1 and POP3 are more diverse than POP2.

Gene expression is typically mediated by transcription factors (TFs) and accessory genes encoding TFs may play a role in toxin production. Among the accessory genes associated with toxin production, 22 were TFs. Among these 22 predicted TFs, 16 showed similarities with hypothetical proteins found in the NCBI nr database. Six other accessory genes matched to TFs associated with different functions in *Fusarium* that have been reported in previous studies.^[^ ^45^, ^46^, ^47^, ^48^ ^]^ Note that while we identified several candidate genes linked to pathogenicity or toxin production, their actual functions need to be verified by subsequent experiments; as these genes are part of the accessory genome, relying solely on a single genome or a limited number of genomes could result in the oversight of specific genes. Hence, it is important to carefully select the appropriate genome in which to either disrupt or introduce the gene of interest. Consequently, a comprehensive analysis of the pangenome becomes indispensable for the future exploration of fungal functional genomes.

We performed a comprehensive pangenome analysis of *F. asiaticum* to exhaustively characterize the diversity of biosynthetic gene clusters (BGCs), coding for specialized metabolites (SMs). In a previous study, Tralamazza et al. (2019) found 50 gene clusters in 2 *F. asiaticum* strains.^[^ ^49^ ^]^ We found 7 additional BGCs, three of which (SM82, SM66, and SM67) showed presence/absence diversity in the *F. asiaticum* pangenome, with high missing rates of 88.6%, 96.3% and 96.3% respectively. This perhaps makes them difficult to detect in studies with a small number of strains. Notably, four other new detected BGCs were present in all 245 genomes of *F. asiaticum*. Among the known SM clusters, three (SM46, SM48, and SM51) showed both length variations and presence/absence diversity in our *F. asiaticum* pangenome. In addition, there was a range of allelic diversity and genetic relationships between SM backbone genes. For instance, in four strains, we found significantly shorter length of backbone proteins of SM39 due to early stop codons introduced by deletions or point mutations. A previous study reported diversity in a koraiol synthase gene, as it was found in at least three functionally redundant copies on the supernumerary chromosome in *F. poae*.^[^ ^50^ ^]^ Our analyses also revealed multiple copies of koraiol synthase (SM33, SM51, SM66, and SM67) in *F. asiaticum*, one belonging to the core and three to the accessory genome. SM66 and SM67 consistently co‐occurred in 9 strains, 7 of which were collected from rice, consistent with a previous study that as the overwintering host of *Fusarium*, rice stubble play an important role in maintaining the diversity.^[^ ^51^ ^]^ Previously, Hoogendoorn et al. (2018) suggested that redundant copies of the koraiol synthase gene could enable the development of new functions and evolutionary innovations.^[^ ^50^ ^]^ The roles of the newly identified koraiol synthase gene remain to be determined.

Our study provides a novel pangenome‐based approach that facilitates the discovery of new SMs, explores genetic variation within these compounds, and enhances our understanding of their evolutionary processes.

Previous studies showed that intra‐species subpopulations of *Fusarium* correspond to trichothecene chemotypes. Using VNTR markers for molecular identification, in several North American studies the FHB pathogens could be divided into two groups related to their trichothecene chemotypes^[^ ^52^, ^53^, ^54^, ^55^, ^56^ ^]^ and a similar VNTR‐based population analysis grouped genetic clades according to chemotypes for *F. asiaticum* in China.^[^ ^16^ ^]^ Nevertheless, our current, more elaborate analyses delineated populations which were only partially consistent with strain chemotypes. We found a significant differentiation of the NIV chemotype and these were found in both POP1 and POP3. The observed population genetic patterns suggest that there have been recent bottlenecks in POP2 and POP3. The limited geographic range of POP2 isolates detected in the MLRYR and Hunan and the geographic isolation of POP3 in Sichuan are reminiscent of some of the other species in the FGSC that are (mostly) confined to specific regions.^[^ ^57^ ^]^ However, our comprehensive and large‐scale sampling also identified some isolates that were associated with POP1 (POP1 *sensu lato*) and some mixed isolates indicating that, although genetic exchange between POP1, POP2 and POP3 is greatly restricted, there might still be some geneflow between these populations, in particular between POP1 and POP2 as these were found in the same geographic area. This extremely limited geneflow further complicates previous debates on clade and species demarcations. As our study shows that these populations are under different selection pressures, they are likely to further drift apart. This could explain the formation of species complexes as observed in several taxonomic groups in *Fusarium*, including the FGSC.

**Figure 5:**
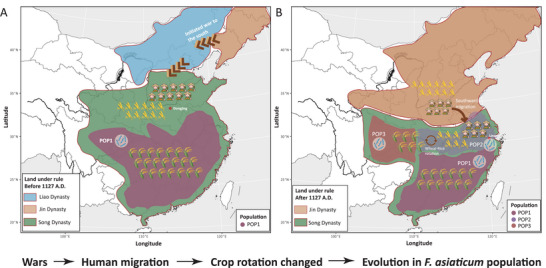
Diagram illustrating the human migration caused by war, southern Invasion by the Jin Dynasty, leading to changes in the cultivation system in the middle and lower reaches of the Yangtze River, and subsequently resulting in the population differentiation of F. asiaticum. A) Boundaries of the Song, Liao and Jin dynasty, crop systems and distribution of *F. asiaticum* population, before 1127 A.D. B) After the war, the boundaries of the Song and Jin dynasty, crop systems and distribution of *F. asiaticum* population after 1127 A.D.

The variations in effective population sizes (Ne) and split time in *F. asiaticum* populations POP1, POP2, and POP3 indicated potential links between the evolution of FHB pathogens and societal changes, human migration, and agricultural practices. Both demographic models and TreeMix analysis unveiled gene flow from POP1 to both POP2 and POP3, suggesting that POP1 served as the ancestral population (Model A in Figure 3D), which aligns with the observed increase in the Ne of POP1 around 8000 years ago (YA). As early as 8000 YA, the cultivation of rice had already been practiced in the southern regions of China.^[^ ^58^ ^]^ The timing of the onset of increase in Ne of POP1 and the emergence of cultivated rice highly coincided with each other. With the emergence and cultivation of rice, the ancestral population (POP1) subsequently witnessed a noticeable increase in its Ne. Bread wheat (*Triticum aestivum*), an important crop in human agriculture, originated around 8500 to 9000 YA through hybridization between a domesticated tetraploid progenitor and *Aegilops tauschii*.^[^ ^59^ ^]^ The widespread cultivation of wheat in Northern China occurred during the Han Dynasty (2229‐1803 YA) and gained popularity during the Tang Dynasty (1405‐1116 YA).^[^ ^56^ ^]^ Subsequently, the rapid population growth, increasing demand for food, and advances in planting and cooking techniques fostered the establishment of high‐yield wheat during the earlier period of the Song Dynasty (1063‐896 YA).^[^ ^60^, ^61^ ^]^ This has contributed to the wider and more widespread cultivation of wheat in Northern China. About 1000 YA, China experienced a cold period.^[^ ^62^ ^]^ The extremely cold climate led to frequent southward invasions by northern nomadic tribes seeking more suitable living conditions. Thereafter, the Jin tribes (898‐897 YA) from Northeast China moved southward, seizing control of northern China by overthrowing the Liao dynasty and occupying the northern territories of the Song dynasty. This forced the Song dynasty to relocate its capital from the Yellow River to the Yangtze River basin. Consequently, a large number of people from the north migrated to the south due to the continuous wars in the northern region, which led to an agricultural change in the south of China from rice‐based cultivation to wheat‐rice cropping rotation,^[^ ^63^, ^64^, ^65^ ^]^ (**Figure** **5**). Based on previous studies,^[^ ^51^ ^]^ the population of *F. asiaticum* would increase dramatically due to this multi‐host per year crop rotation. This is supported by our Ne analysis, which also showed that the effective size of the three populations increased dramatically from approximately 1000 YA.

With the increasing popularity of wheat‐rice rotation in Southern China, the ancestral population POP1 initially experienced growth due to the suitable environment. Over the course of several decades to a century of selection, the Ne of POP3 and POP2 underwent a significant increase, suggesting that they may possess greater adaptability to the new ecological conditions (Figure 5). The increase in the sizes of both populations occurred during a similar period, making it challenging to determine their evolutionary order solely through Ne analysis. To gain insights into the relationship between these populations, we estimated gene flow using both fastsimcoal2 and TreeMix. Both methods consistently indicated that POP3 originated from POP1, suggesting that this population likely experienced a rapid expansion throughout Southern China. However, the MLRYR is more accessible as it is a large plain area, and the predominance of wheat‐rice rotation could rapidly emerge with the influx of northern migrants. This might have resulted in the swift formation of a new local phylogenetic population (POP2) with enhanced adaptability to wheat, following a brief period of POP3 expansion. Notably, the Ne of POP2 started to expand approximately 800 YA, aligning with the widespread adoption of the rice‐wheat rotation system. The unique environment characterized by two cropping seasons per year and the highly conducive conditions for FHB in MLRYR likely facilitated the emergence of the 3ADON group, which is more adaptable to wheat, after which its population grew rapidly and gradually replaced the NIV population on wheat. In addition, we found that the population diversity of the pathogen was related to topography and crop diversity. Regions such as Sichuan, Hunan, and Fujian, where high‐diversity populations POP1 and POP3 are distributed, are mostly mountainous with predominantly small‐scale farming and high crop diversity. In contrast, the plains of the MLRYR feature more uniform crop cultivation, primarily large‐scale rice‐wheat rotation. The local POP2 exhibits the lowest level of diversity and the highest number of genes under selection, suggesting it is subjected to stronger selection pressure. Several studies have reported that *F. asiaticum* is found in rice‐growing regions of Japan, Korea, Uruguay, Brazil, and the United States,^[^ ^13^, ^17^, ^18^ ^]^ predominantly the NIV chemotype. Only the wheat regions in the MLRYR in China predominantly feature the 3ADON chemotype. These regions differ in topography and climate, but the most significant difference is that the wheat regions in the MLRYR strictly practice rice‐wheat rotation. Consequently, the strong selection pressure from wheat cultivation has led to the formation of POP2.

In Southwest China (Sichuan) however, although, wheat was introduced by northern migrants, the adoption of wheat‐rice rotation was not as widespread as in MLRYR due to the region's mountainous geography. The lower selection pressure on wheat allowed POP3 to persist in this area. Subsequently, the effective population size of POP3 experienced a rapid decrease around 250 YA. This decline can be attributed to changes in agricultural practices. In the mid‐17th century, the fall of the Ming Dynasty and wars by Manchu nomads led to widespread starvation and a significant reduction in Sichuan's population and the destruction of the agricultural economy approximately 300 YA.^[^ ^66^ ^]^ Additionally, the introduction of maize to Sichuan around the same time may have favored other members of the *F. graminearum* clade, such as *F. graminearum* and *F. meridionale*, which could have contributed to the decline of *F. asiaticum* in Sichuan.^[^ ^67^ ^]^ These factors likely explain the significant decline observed in the POP3 population around 250 years ago.

Our findings suggest that POP1 represents an ancient group of *F. asiaticum*, which began to flourish with the introduction and widespread cultivation of rice. The population migration caused by the war led to the spread of wheat in the southern regions, resulting in the formation and expansion of the POP2 population in the MLRYR. With the rapid proliferation of rice‐wheat rotation in the MLRYR, POP2 with greater environmental adaptability to wheat emerged in a short period, driven by strong wheat selection pressures. *F. asiaticum* is widely distributed in Asian regions such as China, Japan, and Korea. Recent discoveries have also found it in countries with limited rice production, such as the United States, Brazil and Uruguay. The primary focus of this study was *F. asiaticum* in southern China, for which a dedicated comprehensive survey was conducted and a strict phenotyping procedure was performed. However, we aimed to provide a global context as well. Population genomics of *F. asiaticum* from diverse sources can be utilized to trace the origin, migration routes and evolutionary trajectory of this pathogens, providing possibilities to investigate the evolution of *F. asiaticum* on a global scale. Our study demonstrated that employing k‐mer‐based genomic information for constructing a phylogenetic tree is a rapid and an accurate method for population assignment of new *F. asiaticum* strains. The analysis of the global panel confirmed that the diversity found in China was comprehensive, as no significant new diversity was found in strains from the global panel. We confirmed that strains on rice generally belong to POP1 *sensu late*. The low genetic diversity found in strains in South America and the USA indicates a recent singular introduction with the typical associated genetic bottleneck. In contrast, the population in Japan is diverse and comprises the presence of members of POP1 and POP2. The association of POP2 members with wheat was also confirmed. While Japan is traditionally known for rice cultivation, wheat cultivation is also practiced. In this study, strains from Japan were detected within the POP2 or MIX population. The global spread of POP1 isolates indicates this is the most ancient and dispersed population. The fact that these are NIV producers that are found on rice, rather than POP2 isolates associated with wheat, indicates that wheat is unlikely to be the source of this spread, which aligns with China's status as a non‐wheat‐exporting country (https://www.tridge.com/intelligences/wheat/CN). However, international rice trade remains a potential way for the dissemination of the pathogen. Southeastern China, Japan, and Korea are traditional rice cultivation regions. Strains in Southeastern China were mainly POP1, and the NIV strains from Japan in this study also belonged to the POP1 heterogenous population. Additionally, Southwest (including India), Southeast and South Asian regions (including Thailand and the Philippines) serve as major global rice trade hubs, lacking comprehensive research on *Fusarium* populations, and the presence of *F. asiaticum* is therefore uncertain. The assignment of strains to the POP1‐based heterogenous population in the United States and Brazil suggests that the pathogen may be disseminated through rice trade originating from these areas. Finally, members of POP3 were not found in the global panel, corroborating the conclusions that these are geographically confined to Sichuan. Our study indicates that a k‐mer based‐phylogeny can rapidly provide crucial evidence for the traceback of diverse sources of *Fusarium*.

The unique practice of employing a one‐year wheat‐rice crop rotation within the MLRYR results in two phased/dual selection pressures on the *Fusarium* population, favoring the proliferation of 3ADON‐producing *F. asiaticum*.^[^ ^51^ ^]^ In contrast, where wheat is not included in the rotation, as is the case in parts of southern China,^[^ ^68^ ^]^ South Korea,^[^ ^13^ ^]^ Brazil^[^ ^18^ ^]^ and Eastern Uruguay,^[^ ^17^ ^]^ only NIV‐producing isolates are identified. An unexpectedly high percentage of 3ADON producers was found in regions where rice is the only cultivated crop. These regions are geographically close to the wheat‐rice growing areas of the MLRYR. In contrast, no 3ADON was detected in the rice‐growing regions more distant or more geographically separated by mountains from wheat‐growing areas, like Fujian and Guangxi. The chemotype composition of *F. asiaticum* revealed a gradual decrease in the proportion of 3ADON from the wheat‐rice rotation regions (MLRYR) towards rice growing regions (Hunan and Jiangxi) in the plain areas and completely missing 3ADON in adjacent mountainous regions (Figure 4A). We hypothesized that 3ADON producers in Hunan and Jiangxi may originate from the wheat‐growing regions of MLRYR, which are hundreds of kilometers distant, but have a high abundance of inoculum. Such long‐distance dispersal (LDD) has been extensively studied in obligate biotrophic plant‐pathogenic fungi, where it has been clearly demonstrated that they can spread over thousands of kilometers. However, for non‐biotrophic fungi, which have a wide host range, there are no reports or direct evidence suggesting their capability for LDD. Given the strong association of 3ADON populations with wheat, coupled with the findings of this study, we believe that examining the example of 3ADON of *F. asiaticum* can help further explore the possibility of LDD in non‐biotrophic fungi.

First, we established that the 3ADON populations in rice‐growing regions and MLRYR represent a single population. We used whole genome‐based analyses, including neighbor‐net phylogenetic network, maximum likelihood phylogeny and ancestry population analyses, and confirmed that 3ADON producers in Hunan and MLRYR are representatives of one population. What factors then prompted the migration of 3ADON from the MLRYR to Hunan or Jiangxi across hundreds of kilometers in the rice‐growing region? Recent studies demonstrated the potential of FHB pathogens for atmospheric dispersion over short and middle distances. Francl et al. (1999) observed viable spores of FHB pathogens trapped on the roofs of buildings located several kilometers away from the inoculum sources.^[^ ^30^ ^]^ In addition, at 40–320 m above ground, abundant viable inoculum of FHB pathogens was found that could cause FHB on a susceptible cultivar of spring wheat.^[^ ^69^ ^]^ Prussin et al. (2015) used three microsatellite markers to validate that the *Fusarium* isolates recaptured at 750 m were genotypically identical to the inoculated spores. However, the recaptured clonal inoculum ratio was extremely low, for instance 6.7% (1/15) in 2011 and 0% (0/5) in 2012.^[^ ^70^ ^]^ So far, there is no direct evidence that FGSC members can be dispersed in the atmosphere over long distances and cause infection. In this study, we found a gradual change in the proportion of 3ADON over Hunan and Jiangxi from north to south. Human activities, seeds, soil, or harvesting tools can cause long‐distance migration of pathogens. However, in our study, we found a uniform gradient distribution of the two chemotypes, which is not only related to distance but also closely associated with topography. Geographic barriers have the most significant and direct impact on airflow dispersal. Compared to factors related to human activities, airflow is more likely to form such a uniform gradient distribution. Although most areas in Hunan and Jiangxi are mountainous, there are transitional regions between plains and mountainous areas from MLRYR to Hunan and from MLRYR to Jiangxi (Figure 4B) that may facilitate the dispersal of airborne *Fusarium* spores. The airflow trajectory cluster simulation over the flat plains incorporating various factors, including the period when ascospores are released (March to April) and when conidia are generated (May), as well as distinct heights (60 m and 500 m above ground), supported the potential for LDD of 3ADON strains from the MLRYR to adjacent regions. Combined with existing reports of high‐altitude capture of *Fusarium* spores,^[^ ^30^, ^69^, ^70^ ^]^ spore dispersal by airflow is the primary mode of long‐distance spread, although other methods may also contribute to some extent. Taken together, our population genomic and airflow trajectory analyses suggested that LDD (>250 km) played a significant role in the epidemiology and population structure of *F. asiaticum*. To our knowledge, this is the first report directly confirming that non‐biotrophic fungi can be dispersed across long distances.

There have been reports on LDD of pathogens causing infection. For instance, Chen et al. (2014) unveiled that after undergoing over‐summering, urediniospores of wheat stripe rust located in southeastern Gansu regions could be carried by winds to northwestern Hubei in autumn and result in the wheat stripe rust in the subsequent growing season.^[^ ^71^ ^]^ However, unlike wheat stripe rust pathogen, which can infect leaves during the broad growth period of wheat, the FHB pathogen has an extremely short period for infecting wheat spikes, only during the flowering period. Therefore, long‐distance dissemination may not directly contribute to local infection of FHB. This is consistent with the primary inoculum of FHB being mainly derived from local overwintered plant residues,^[^ ^72^, ^73^ ^]^ as supported by previous studies that found that FHB pathogens can remain viable for at least 1–2 years,^[^ ^74^ ^]^ and even longer than 2 years,^[^ ^75^ ^]^ within plant debris present on or above the soil surface, thereby serving as a prolonged source of inoculum. On the other hand, the use of tillage to bury the residues of host crops and the practice of crop rotation with non‐hosts has been demonstrated to mitigate both the intensity of FHB and the accumulation of deoxynivalenol (DON) in infected grain. We found although the timing of FHB pathogen spore dispersal in the air was unlikely to directly lead to the infection of wheat, the local pathogens composition and its evolution are strongly affected over very long distances. With respect to disease management this demonstrates that consideration should be given to the potential influx caused by the LDD by air of plant pathogens from highly infected areas that could change the population structure and include more adapted, more pathogenic and fungicide resistance genotypes.

## 4 Experimental Section

### Sample Collection

From 2014 to 2019, a large‐scale of FHB investigation was conducted, and samples were collected in eight provinces and one direct‐administered municipality, including Sichuan, Hubei, Anhui, Jiangsu, Hunan, Jiangxi, Guangxi, Fujian and Chongqing. Based on the geographical conditions and cropping systems, these regions can be classified into four ecological regions: 1) the Middle‐Lower reaches of the Yangtze River (MLRYR) represents a plain region, including Hubei, Anhui, and Jiangsu. Strict wheat‐rice rotation dominated this region, and *Fusarium* strains were collected from wheat heads and perithecia on rice debris; 2) Southwest region represents a mountainous region including Sichuan and Chongqing. This region had high crop diversity, rice was predominant and had a small acreage of wheat which rotated with rice, maize, soybean and several other crops. *Fusarium* strains were collected from wheat heads and perithecia on rice debris in this region; 3) The south and east regions, including Guangxi and Fujian, also represent mountainous regions, but the cropping system was different from Southwest region. Wheat was not grown and double cropping of rice were common in this region. So *Fusarium* strains were obtained only from rice stubble; 4) Hunan and Jiangxi represent transition regions between plains and mountains, similar with south and east region, rice was predominant and no wheat grown in this region. *Fusarium* strains were obtained only from rice stubble.

Species and trichothecene chemotype were identified by the multilocus genotyping (MLGT) assay.^[^ ^76^ ^]^ A total of 4427 *F. asiaticum* strains were collected. Then 245 represented *F. asiaticum* isolates were selected, from four ecological regions in Southern China, which included six provinces (Anhui, Jiangsu, Hubei, Hunan, Sichuan, and Fujian), for the population genomic analysis. There were three types of trichothecene chemotype among 245 strains, including NIV, 3ADON, and 15ADON. The sampling information of the above strains was described in detail in Table S1 (Supporting Information).

### Pathogenicity Measurement

A medium‐susceptible wheat cultivar (Annong 8455) was used for pathogenicity determination. NaClO (30%) was used for seed sterilization with 1 minute treatment followed by several rinses with sterile water. Afterwards, seeds were soaked in distilled water for 24 hours (h). Germination was carried out in trays with 100% humidity for 2 days. A total of 15 seeds with good germinability with a coleoptile length between 2 to 3 cm were plated in 90‐mm dishes with filter paper. The sterilized filter paper dipped with conidial suspension (106 conidia ml^−1^) at the 2–3 mm clipped coleoptile apex. The plates were inoculated in an illuminated incubator (25 °C, 95% humidity, 16 h light‐8 h darkness cycle). Seven days after inoculation, the length of the lesion on the coleoptile was measured.

### Toxin yield Determination

Twenty‐five grams of rice were placed into conical flasks, soaked, steam‐sterilized with an autoclave, and subsequently inoculated with five 1‐cm‐diameter mycelial plugs taken from the actively growing colonies on potato dextrose agar in each conical flask. The mixtures were then incubated under 28 °C for 7 days, with each sample being conducted in triplicate. A IKA Tube Mill was used to grind each sample into a fine powder. A total of 5 g of the sample was extracted using 25 mL of 80% (v/v) acetonitrile through 2 min of shaking followed by centrifugation at 9,000 rpm for 3 min at room temperature. Next, the resulting supernatant was purified using a multifunctional purification column and an 0.22 mm nylon filter.

Chromatography, for the measurement of DON, 3ADON, 15ADON, and NIV toxins in the samples, respectively, was conducted using a Waters Scientific reversed phase UPLC BEH C18 column (100 × 2.1 mm, 1.7 µm). Methanol (A) and 10 mmol l^−1^ ammonium acetate (B) were used as the mobile phases. The gradient elution program employed was as follows: 0–3 min, 20–50% A; 3–6 min, 50–90% A; 6–7 min, 90% A; 7–8 min, 90‐20% A; 8–10 min, 20% A. The instrument was utilized for both positive and negative ionization modes. Quantification was achieved via multiple reaction monitoring (MRM). The source and desolvation gas temperatures were set to 150 °C and 350 °C, respectively. The desolvation and nebulizer gas flows (N2) were adjusted to 650 and 50 l/h, respectively. The collision gas (Ar) was delivered at a flow rate of 0.14 ml min^−1^.

### Whole‐Genome Sequencing and Assembly

Whole genome sequencing of *F. asiaticum* was performed by Biomarker Technologies Company (Beijing, China). 245 independent libraries were normalized to equal amounts and pooled, and then sequenced using the Illumina HiSeq × Ten platform with 2 × 150 bp paired‐end reads at ≈50X coverage per sample. One of those strains (180197) was also used for PacBio sequencing. A PacBio library was constructed using the manufacturer’ s protocol and sequenced on the PacBio RSII system. In addition, nine *F. asiaticum* strains selected to represent the global diversity (four strains from the United States, three from Brazil, two from Japan) were also sequenced.

### Reference Genome Assembly

The genome assembly of the strain sequenced by PacBio (180197) used two tools: NextDenovo v2.5.0^[^ ^77^ ^]^ with default parameters, except read cutoff 15k and Flye v2.3.8^[^ ^78^ ^]^ with ‐g 37.5m, ‐i 2, followed by NextPolish v1.3.1^[^ ^79^ ^]^ to polish both assemblies. The two assemblies were manually merged to generate four chromosome‐level sequences. The ribosomal genome was assembled using Illumina data and the single rDNA copy of PH‐1. Bwa‐mem2 v2.2.1^[^ ^80^ ^]^ and SAMtools v1.9^[^ ^81^ ^]^ were used to generate the alignment file and coverage depth file of the rRNA gene, normalized based on the average coverage of strain 180197. The SPAdes assember v3.15^[^ ^82^ ^]^ was used to assemble the single copy rDNA sequences, which were concatenated and copy‐number adjusted onto the fourth chromosome. Finally, the assembled genome was polished using Illumina reads through Pilon v1.24.^[^ ^83^ ^]^

### Assembly of Other Genomes

Illumina paired de‐multiplexed FASTQ files were processed using the Casava v1.8 program. Raw data were quality controlled using fastp v0.21.0^[^ ^84^ ^]^ with forced polyG tail trimming (‐g) and minimum quality Phred score ≥ 20 (‐q 20). The SPAdes assembler v3.15 was used for *de novo* assembly, generating contigs of the nuclear genomes of the sequenced strains. K‐mers series “31, 61, 91, 121” were selected. The sidr package was used to decontaminate. The nt database in NCBI and the reference genome (180197) were merged into a BLAST database. Sequences not belonging to Ascomycota contigs were removed (https://github.com/damurdock/SIDR). A custom script was used to remove short contigs less than 500 bp. Contigs between 500 bp and 1000 bp were checked for sequence identity ≥70% and coverage ≥80% against with 180197, and those with GC contents within the mean ± 2 × standard error confidence interval were kept. The RagTag^[^ ^85^ ^]^ software with default parameters and a custom script were used for ordering and orienting the contig‐level assemblies into 4 chromosomes, using strain 180197 as the reference. The sordariomycetes_odb10 (for Sordariomycete species) of BUSCO V5^[^ ^86^ ^]^ was used for the evaluation of the assembly completeness in genic regions.

### Genome Annotation

Full gene structure annotation proceeded using BRAKER2 v2.1.6^[^ ^87^ ^]^ with the ProtHint pipeline. Both RNA‐seq and proteins were used for supporting gene evidence during the annotation process. First, a repeat library of each sample genome was ab initio constructed using RepeatModeler2 v2.0.1^[^ ^88^ ^]^ with default settings, and then the library was used to softmask the genome using RepeatMasker v4.1.1 (http://repeatmasker.org/). Then alignment of the RNA‐Seq data to the masked genome was performed using HiSAT2 v2.2.1.^[^ ^89^ ^]^ Protein evidence was composed of OrthoDB v10^[^ ^90^ ^]^ fungi ortholog sequences, TRI genes and standard strain PH‐1 genes.

COG functional annotation was performed using EggNOG‐mapper v2.1.5^[^ ^91^ ^]^ with DIAMOND mapping mode. Gene Ontology terms, protein motifs and domains were annotated using InterProScan,^[^ ^92^ ^]^ by searching against database, including Pfam, TIGRFAM. Standalone antiSMASH v6.1.1^[^ ^93^ ^]^ was used to predict BGC's. ClusterProfiler v4.6.2^[^ ^94^ ^]^ was employed to conduct GO enrichment analyses on R v4.2.2 with screening criteria of p.adjust <0.05.

### Pangenome Construction and Investigation

A high‐quality pangenome was constructed in a modified version of PanTools v3^[^ ^95^ ^]^ using a k‐mer length of 17, including 245 *F. asiaticum* and 1 *F. graminearum* PH‐1. Structural and functional annotations of each strain were added in the pangenome. The optimal setting to group the homologous proteins was determined on the BUSCO genes, which were expected to represent a single group with a single member from each genome, using the optimal_grouping function with –longest‐transcript parameters to calculate the optimal homology groups with the longest transcripts. BUSCO analyses were performed with BUSCO v5 in PanTools with the busco_protein function.

Core homology groups were defined as groups shared by all 245 genomes. Those only present in one genome were defined as unique groups. Homology groups that were present in some, but not all genomes were defined as accessory groups. The PanTools metrics function was used for generating relevant metrics of the pangenome and the individual sequences and genomes. The gene_classification function was used to classify the pangenome's gene repertoire as core, accessory or unique. The core_unique_thresholds function was used to test the effect of different thresholds of unique and thresholds of core cut‐offs between 1 and 100%. The grouping_overview function provided all homology groups of the different grouping setting. The pangenome_structure_genes function generated the accessory, core and unique homology group numbers. The functional_classification function identified the accessory, core and unique gene functional annotations, including GO, InterPro, PFAM, TIGRFAM and antiSMASH. The function_overview function generated the summary files of each type of functional annotation. A phylogeny was created with the Maximum Likelihood (ML) algorithm using the SNPs identified in the single copy core homology groups, using the core_phylogeny function.

Subsequently, an association analysis between accessory homology groups and phenotyping data were performed. We used the information of accessory homologous groups obtained from the gene_classification function in PanTools to convert the information of accessory genes in each genome into a presence/absence format. Then the pathogenicity, content of 3ADON, DON, NIV, and 15ADON toxins was individually assessed to determine whether significant differences exist in the presence/absence in these phenotypes across each accessory group within the pangenome of *F. asiaticum* using a t‐test in R. Accessory homology groups significantly related to the pathogenicity and toxin content of DON, 15ADON, 3ADON and NIV, respectively, were generated at p‐adjust (FDR) < 0.0001. Finally, based on the results of the functional annotation, homology groups predicted were selected to contain coding TFs from those significantly correlated with pathogenicity, and homology groups predicted to contain coding secreted proteins from those significantly correlated with toxin content. To assess the function of the unique proteins in the pangenome, a BLASTP search was conducted of the unique proteins identified by the gene_classification function in PanTools, against the NCBI nr database.

The analysis of Biosynthetic Gene Clusters (BGCs) combined the antiSMASH prediction results and the pangenome homology group information. The following analyses were performed on the backbone genes of predicted BGCs: (i) identifying the backbone genes homology groups in *F. asiaticum* pangenome; (ii) extracting the gene nucleotide sequences of the backbone genes; (iii) investigating the diversity in each homology group.

### Read Mapping and Variant Calling

Three different types of variants were detected, including SNPs, indels and structural variants (SVs). SNP and small indel calling were performed by the Genome Analysis Tool Kit (GATK v4.2.6.0).^[^ ^96^ ^]^ The reference genome assembly (180197) index files were made using bwa‐mem2 and SAMtools. CreateSequenceDictionary was used on 180197 with default parameters. The other 244 *F. asiaticum* strains were aligned to 180197 using bwa‐mem2 with default parameters. SAMtools view with ‐buS, sort and index were used to sort and index the alignment files. Mate‐pair information was corrected, and duplicates were marked using FixMateInformation and MarkDuplicates functions, respectively, followed by generating the indexed files using samtools. HaplotypeCaller was used for SNPs and indels discovery followed by combining the GVCF files of all strains and then joint‐genotyping with GenotypeGVCFs. Subsequently, variants were filtered using VariantFiltration with the following parameters: ‘QD < 2.0, MQ < 40.0, FS > 60.0, SOR > 3.0, MQRankSum < −12.5, ReadPosRankSum < −8.0’. Indels and SNPs were then separated using SelectVariants. vcfR and a custom R script were used for excluding SNPs and indels below the 5% lowest and above the 5% highest depth summed across samples. Variants with missing data in more than one sample were excluded using ‐max‐missing 1 in VCFtools v0.1.16.^[^ ^97^ ^]^ SNPs and indels were annotated using SnpEff v4.5.^[^ ^98^ ^]^ Variants were categorized based on their positions on the reference (180197) chromosome (including introns, exons, intergenic regions, and splicing sites).

To survey for structural variation (SV) in *F. asiaticum*, a reference‐free whole‐genome alignment and a pangenome graph construction were performed with Cactus v2.2.3.^[^ ^99^ ^]^ Using 245 *F. asiaticum* genome sequencing files, a structural variation graph was generated using the ‘cactus‐minigraph’ function, and the genomic coordinates of 180197 were utilized solely as positional information. Then each input assembly was mapped back to the graph with minigraph using “cactus‐graphmap' function. Next, the Cactus multiple genome alignment was generated through assembly‐to‐graph minigraph mapping with ‘cactus‐align”. The final pangenome graph and indexes were produced using the “cactus‐graphmap‐join” function. Subsequently, high‐confidence SVs with length larger than 500 bp, allele frequency (AF) larger than 5% and allele numbers (AN) larger than 200 were selected. Finally, the SVs were subjected to BLAST in the nt database to compare them with reported genomes.

### Population Genetic Analyses

ADMIXTURE v1.3.0^[^ ^100^ ^]^ was used for population genetic structure investigation using the high‐quality SNPs. To reduce the impact of linkage disequilibrium (LD) and the computational load, SNP sites based on the observed sample correlation coefficients were filtered. PLINK v1.9^[^ ^101^ ^]^ with ‘–indep‐pairwise 100 50 0.2’ was performed to filter SNP sites. To identify the best number of clusters K fitting the data, 5‐fold cross‐validation was performed for K values ranging for 2 to 7. A network phylogeny was inferred for the data generated from ADMIXTURE analysis using SplitsTree v4.18.3^[^ ^102^ ^]^ with the NeighborNet algorithm. Principal Coordinates Analysis (PCoA) was performed on the same filtered SNP set using PLINK, and the first two principal components were visualized using the ggplot2 package in R v4.1.2. A whole‐genome SNP phylogeny was constructed with 1000 bootstraps using the IQTREE2 v2.1.3^[^ ^103^ ^]^ automatic selection model and visualized using iTOL v6.7.3.^[^ ^104^ ^]^ Phylogenies were compared using TreeCmp^[^ ^105^ ^]^ by calculating the normalized Nodal Splitted metric with L2 norm (nNS) and normalized matching‐cluster (nMC) values.

The whole‐genome nucleotide diversity (π), nucleotide polymorphism (θ), haplotype diversity (h), Tajima's D, recombination events (Rm), and the fixation index (Fst), in 10 kb non‐overlapping sliding windows, were estimated for each population. Using the chromosomes of 180197 as reference, π, recombination events and Tajima's D for each population were visualized in Circos v0.69‐8.^[^ ^106^ ^]^

### Demographic Analysis of *F. asiaticum* Evolutionary History

Multiple Sequentially Markovian Coalescent (MSMC2) v2.1.1^[^ ^107^ ^]^ was used for inferring the demographic history of the *F. asiaticum* populations. Coalescence analyses utilize a backward‐in‐time algorithm to reconstruct the lineage of genomes by randomly tracing their history from the present generation. Demography analysis and cross‐coalescence analysis were conducted using randomly selected eight and four strains for each population, chosen to best represent the population based on the PCoA result. Each analysis was run for five iterations in two distinct steps. First, the strategy described above was used to call SNPs and generate a VCF file. Then bedtools was used to calculate the coverage of each site in each strain and mask sites with coverage higher than quantile 99.9% or frequency lower than 6, after which the sites in all eight strains in each population were combined. After that, a custom script was used to generate the correct input format files for MSMC2. Due to the inaccurate reconstruction of the most recent generations by these inference methods,^[^ ^108^, ^109^ ^]^ they were excluded from interpretation.

Complex evolutionary models were simulated and evaluated for their likelihood of being accurate using coalescence simulation methods (fastsimcoal2).^[^ ^110^ ^]^ In this study, the order of split events were tested in three main populations. In this analysis, the unfolded SFS and focused exclusively on derived alleles were employed. The models were simulated with 100000 runs per replicate (‐n) for 100 replicates performing parameter estimation by “‐d −0 ‐l 10 ‐n 100000 ‐L 40 ‐s 0 ‐M ‐q”. Other scenario tests were in Figure S4 (Supporting Information). In general, the most probable scenarios were also the ones that receive the strongest support. The Akaike Information Criterion (AIC) was used to assess model quality.

### Detection of Selective Sweeps

To identify the signatures of selection in the genome in each population of *F. asiaticum*, three different statistics were used for optimizing efficiency rates and minimizing false‐positive rates, including Tajima's D, Fst, and HapFLK v1.3.0.^[^ ^111^ ^]^ Tajima's D and Fst of each population were calculated as described above. Tajima's D and Fst values were then combined to identify potential selective sweep regions. The top 1% of the Fst windows and the top/bottom 1% quantile of the Tajima's D windows of each population were selected. The HapFLK approach takes the haplotype structure of the population into account. Haplotype clusters per chromosome were estimated using cross‐validation‐based with fastPHASE, at a set cluster value of 40.^[^ ^112^ ^]^ Then HapFLK was computed individually on each chromosome and loci with the lowest 5% of p‐values were considered to be under selection, with the 5‐kb regions surrounding the site as the selected region. Finally, a custom script was used to extract the overlapping regions under selection and the genes involved.

### Population Assignment of *F. asiaticum* Isolates Based on k‐mer Analysis

Nine additional *F. asiaticum* isolates selected as a global reference panel were sequenced to assess the genome variation on global level by population assignment. To balance the population assignment, representative strains from the 245 Chinese strains were selected based on the results of population structure analyses (five strains from POP1, five from POP2, five from POP3, and ten MIX strains). In total 34 whole genomes of *F. asiaticum* and *F. graminearum* (PH‐1) as an outgroup were used to construct a phylogenetic tree based on the k‐mer distance module in PanTools. This allowed positioning these isolates within the phylogenetic tree of the isolates from China. To verify the correct grouping based on the k‐mer distance a population structure analysis of all 254 *F. asiaticum* isolates (245 strains from China, nine strains from other countries) was conducted in ADMIXTURE using whole‐genome SNPs as described in the section “Pangenome construction and investigation”.

### Trajectory Simulation Analysis of F. asiaticum Migration

To determine the possible direction of *F. asiaticum* transport from the MLRYR to Hunan and Jiangxi, the trajectory of atmospheric air masses carrying particulate material was examined, and the associated trajectory and conceivable source areas were established. The backward trajectory simulation was calculated by TrajStat v1.4.8 plugin in MeteoInfoMap v2.2.1, using Hybrid Single‐Particle Lagrangian Integrated Trajectory (HYSPLIT)^[^ ^113^ ^]^ model with the weekly meteorological data from the National Oceanic and Atmospheric Administration (NOAA) (ftp://arlftp.arlhq.noaa.gov/pub/archives/gdas1). Three representative locations were selected in Hunan and Jiangxi, respectively. We simulated that the spores of *Fusarium* would be released at 18:00, 21:00, 24:00, 03:00, and 06:00 UTC every day. Continuous transport was simulated for 72 h to determine the forward airflow trajectories. Based on the sampling collection time, Hunan was simulated at a height of 60 m and 500 m above ground from 1st March to 31st May in 2017 and 2019, and for Jiangxi at a height of 60 m and 500 m above ground from 1st March to 31st May in 2019. Subsequently, modelled trajectories were combined, and the clustering function was applied to decrease the dimensionality. Finally, 18 modelled air‐mass particle history trajectories were generated.

### Data and Code Availability

Raw genome reads, genome assemblies and annotation files of 245 *F. asiaticum* isolates were deposited into the National Center for Biotechnology Information in BioProject database (https://www.ncbi.nlm.nih.gov/bioproject) under the accession PRJNA847718 and the Figshare database (https://figshare.com/projects/Adaptation_of_Fusarium_Head_Blight_Pathogens_to_Changes_in_Agricultural_Practices_and_Human_Migration/213958). The code examined in this research has been detailed in the Methods section and can be accessed on GitHub via the following link: https://github.com/ymxNancy/Code_Fasiaticum_pangenome.

## Conflict of Interest

The authors declare no conflict of interest.

## Author Contributions

M.Y., H.Z., T.A.J.L., J.F., T.L., W.C. performed conceptualization; M.Y., H.Z., T.A.J.L., S.S. performed methodology; M.Y., H.Z., T.A.J.L., S.S. performed investigation; M.Y., H.Z. performed visualization; H.Z., T.A.J.L., W.C. performed supervision; M.Y., H.Z. wrote—the original draft; S.S., D.R., J.X., T.A.J.L., H.Z., W.C. wrote—review & edited the original draft

## Supporting information

Supporting Information

## Data Availability

URL: https://github.com/ymxNancy/Code_Fasiaticum_pangenome.
