# Supplementary material for: Adaptation of Fusarium Head Blight Pathogens to Changes in Agricultural Practices and Human Migration
Source: Adv Sci (Weinh). 2024 Aug 5;11(36):2401899. doi: 10.1002/advs.202401899 (PMC11423162; doi:10.1002/advs.202401899)
Supplement: Supplementary file 1 — Supporting Information [file ADVS-11-2401899-s001.pdf]

ADVANCED SCIENCE
Open Access

Supporting Information

for *Adv. Sci.*, DOI 10.1002/advs.202401899

Adaptation of Fusarium Head Blight Pathogens to Changes in Agricultural Practices and Human Migration

Meixin Yang, Sandra Smit, Dick de Ridder, Jie Feng, Taiguo Liu, Jinrong Xu, Theo A. J. van der Lee, Hao Zhang* and Wanquan Chen**

Supplemental Text

Supplemental Text S1

Over 84% of the homology groups in the *F. asiaticum* pangenome belong to the core group

The 245 high-quality *F. asiaticum* genomes served as input to construct a *F. asiaticum* pangenome using PanTools. The calculated F scores of the correct grouping were higher than 99.9% with protein sequence similarities ranging from 95% to 55%. We selected 55% protein sequence similarity as the cutoff for shared genes as genes could show lower conservation levels compared to (generally more conserved) BUSCO genes (Table S6). To assess the representativeness of 245 strains used in this study, we performed a simulation test with 10,000 re-samplings. As the number of included genomes increases, the core, accessory and unique homology groups tended to plateau (Figure 1B), indicating that the *F. asiaticum* pangenome is representative of existing gene diversity. The Heaps' law value of 1.038 also supports the 245 genomes as a sufficient representation of the genomic diversity in the entire *F. asiaticum* population. In total, the *F. asiaticum* protein pangenome comprised 17,769 homology groups, including 11,173 core groups, 5,845 accessory groups and 751 unique groups. An average *F. asiaticum* genome consists of 84.79% (σ 0.49) core genes, 15.18% (σ 0.48) accessory genes, and only 0.02% (σ 0.02) unique genes. In order to interpret the various homology groups biologically, we incorporated information from the Gene Ontology (GO), SignalP, transcription factors (TF), InterPro, Pfam, COG databases, specialized metabolites (SM), and secretory proteins (Table 1).

Firstly, we compard the correlation between the core genome and the whole genome of *F. asiaticum* pangenome. A comparison of two SNP-based phylogenetic trees was performed. A total of 159,127 SNPs were identified and used to generate the core phylogeny of the strains. In addition a total of 516,591 whole genome SNPs were generated by variant calling and filtration steps, taking the 180197 genome as reference. The measurement of congruence involved normalized Nodal Splitted metric with L2 norm (nNS) and normalized matching-cluster (nMC) values, which ranged from 0 to 1. Lower nNS and nMC scores reflect higher levels of congruence between trees: A score of 0 for both nNS and nMC indicates complete congruence between trees, while a score of 1 indicates a lack of congruence. The comparison of the phylogenies based on the core SNPs and the whole genome SNPs revealed relatively low nNS (= 0.301) and nMC (= 0.297) values, suggesting highly similar phylogenies. The result revealed that although a completely different set of SNPs were identified in the core genome and whole genome the phylogeny is similar and robust.

Supplemental Text S2

Structural variation analysis of *F. asiaticum* pangenome

To survey for structural variation (SV) in *F. asiaticum*, a reference-free whole-genome alignment and a pangenome graph construction were performed in Minigraph-Cactus using the reference genome of

isolate 180197. To identify high-confidence SVs, we selected those of 500bp or larger, a threshold with an allele frequency (AF) larger than 0.05 and an allele number (AN) larger than 200. In total 104 positions with structural variations (SVs) were identified. Most of SVs were 500 bp - 1 kb (64), followed by 1kb - 2kb (44) and ≥ 5kb (20) (Figure 1C). Subsequently, to identify the diversity of the SVs in *F. asiaticum*, we focused on 12 positions with SVs length ≥ 5kb. All the genes involved in these 12 SV regions were hypothetical proteins. To further clarify the origin of these larger SVs, the nucleotide sequences of these SVs were analysed by BLAST against the nt database. One SV involved a 9,751 bp sequence uniquely present in the reference genome of isolate 180197, harboring a long terminal repeat (LTR) region. This region can only be found in the NCBI database of chromosomal level assemblies of *F. asiaticum*, *F. graminearum* and *F. culmorum* genomes, which indicated that this region was present in *Fusarium* and also exemplify that highly repetitive sequences are difficult to assemble with short reads sequencing techniques. Another SV region containing a sequence of 10,272 bp was present in only 2 *F. asiaticum* genomes and using BLAST had the best hit in a *F. oxysporum* genome with 84% query coverage and 97.84% nucleotide identity. This result suggests that this sequence was likely obtained via horizontal gene transfer. Thirdly, there were 1,466 bp, 16,807 bp and 429 bp structural variation sequences present in 23, 14 and 36 *F. asiaticum* genomes respectively, while they were not found in other *Fusarium* genomes. This indicated these structural variants may have arisen after speciation of *F. asiaticum* and subsequent spread to a limited number of isolates by horizontal gene transfer. The remaining larger SVs were also detected in other *Fusarium* species, indicating they are diverse, ancient, and likely predate speciation of *F. asiaticum*.

Supplemental Text S3

Accessory homology groups associated with plant infection and mycotoxin production

In the *F. asiaticum* pangenome, 5,845 accessory groups (32.9%) were identified. After removing those genes that were present/absent in more than 235/245 genomes (which would reduce the statistical power), the remaining 3,027 accessary genes that were tested for their association with pathogenicity on coleoptiles and/or mycotoxin production. We identified 437 accessory genes showing significant presence/absence difference with coleoptile pathogenicity ($P < 0.0001$) of which 30 were predicted to encode secreted proteins (Table S4). Among these 30 candidate secretory proteins, 25 were hypothetical proteins in *Fusarium* and one did not show significant homology to any protein in the database (NCBI accessed). The remaining four genes showed significant matches to known secretory proteins in *Fusarium*. Based on the toxin profile, we recognized five types of mycotoxins in these strains: DON, 3ADON, 15ADON, and NIV, and verified the association between the presence/absence of accessory genes and toxin production. In total, 403 genes showed significant presence/absence difference ($P < 0.0001$) among 3ADON producing isolates including 14 genes that were predicted to encode transcription factors (Table S4). Similar results were obtained for the other four chemotypes: 15ADON with 385 significant genes including 7 TFs; DON with 260 significant genes comprising 5 TFs; and finally, NIV with 110 significant genes including 2 TFs. The largest number of TFs (14) is associated with 3ADON production. 11 were uniquely present in the 3ADON population, two of the remaining three were associated with production of 15ADON, and the

remaining one was associated with the productions 3ADON, 15ADON and DON. Of the seven TFs potentially associated with 15ADON production, one TF was associated with both 3ADON and 15ADON, one TF potentially associated with both 3ADON and DON production. In addition, one TF was potentially associated with both DON and NIV production and four 15ADON-specific TFs were detected. Finally, in DON-producing strains, 2 specific TFs were observed, while only one specific TF seemed associated with NIV-producing isolates.

Supplemental Text S4

The high diversity of SM gene clusters in *F. aisaticum* pangenome is reflected not only in presence/absence, absence rates, but also in the similarity of backbone genes sequence.

A total of 10,914 secondary metabolite (SM) gene clusters was predicted in the *F. asiaticum* pangenome with an average of 44.5 SM gene clusters (σ 1.24) per genome. There were 57 kinds of SMs, 9 categories in total (Figure 1D), including several hybrid clusters that contained multiple classes of backbone genes. The class of nonribosomal peptides synthetase (NRPS) and terpenes cyclase (TPS) were the most frequent SM gene cluster categories (n = 16 respectively), followed by polyketide synthase (PKS) and NPRS-PKS (n = 8, respectively). Among the 57 SM, 51 were found in all 245 *F. asiaticum* genomes, while 6 showed presence/absence diversity. In the 6 accessory SM gene clusters, the range of missing frequencies ranged from 10.2% to 96.3% and the present backbone proteins were not completely conserved. We identified significantly shorter backbone proteins due to deletion, or early stop caused by point mutations. For example, in SM48, due to deletions and early nonsense mutations, 6 types of proteins of different lengths were found among the strains. Similar mutations were also observed in 51 other conserved SM clusters.

Notably, we found SM33, SM51, SM66, and SM67 gene clusters were predicted to encode enzymes that synthesize the same compound, koraiol. Whereas these four SM backbone genes showed different diversity among *F. asiaticum* genomes in our study. SM33 was extremely conserved in all *F. asiaticum* genomes. SM51 was missing in 42.4% of the 245 genomes but highly conserved among those that contained the gene. SM66 and SM67 were always present simultaneously, detected in 9 genomes. For SM66, the backbone gene was significantly shorter in three genomes due to an early nonsense mutation caused by a point mutation.

Supplemental Text S5

Identification of three distinct populations

A total of 516,591 parsimony informative SNPs in 245 *F. asiaticum* genomes were identified and used for population structure and relationship analysis. A high-resolution network phylogeny was constructed from the whole genome SNPs. The individual ancestry and ADMIXTURE proportions for each genome were also inferred. At K = 3, the ADMIXTURE corresponded well with the structure found in the network phylogeny (Figure 2A). A clear separation of three groups was found, with the

admixed samples positioned between the clusters. Moreover, the network indicated few meiotic recombination and outcrossing between isolates, but most isolates did not show such signatures of recombination and are therefore likely the result of only clonal reproduction or selfing. Note that no identical genome sequences were obtained and all isolates were genetically distinct. We assigned each *F. asiaticum* isolate to either POP1, POP2, POP3 and MIX based on this ADMIXTURE analysis. In subsequent analyses, we focused on the three main genetic clusters (POP1, POP2 and POP3).

To explore the phylogenetic relationship among the strains, we constructed a maximum likelihood phylogeny using the published genome of *F. graminearum* strain PH-1 as an outgroup with a total of 915,953 parsimony informative whole genome SNPs. We also flagged related metadata features of each isolate on the phylogeny, including chemotype, geographic origin, host and ancestry proportions in K = 2 and K = 3 clusters (Figure 2B).

Tables

Table S1 Sheet1 The collection information of all F. asiaticum strains in this study

**Strains**	**Year**	**Province**	**Host**	**Species**	**Toxins**	**Latitude**	Longitude	Altitude	Wheat variety	Field type
180965	2018	Sichuan	Wheat	*F. asiaticum*	NIV	29.38913802	104.432672	461.83255	Commercialized variety	Farmer owned field
180964	2018	Sichuan	Wheat	*F. asiaticum*	NIV	29.38913802	104.432672	461.83255	Commercialized variety	Farmer owned field
180961	2018	Sichuan	Wheat	*F. asiaticum*	NIV	29.38913802	104.432672	461.83255	Commercialized variety	Farmer owned field
180960	2018	Sichuan	Wheat	*F. asiaticum*	NIV	29.38913802	104.432672	461.83255	Commercialized variety	Farmer owned field
180959	2018	Sichuan	Wheat	*F. asiaticum*	NIV	29.38913802	104.432672	461.83255	Commercialized variety	Farmer owned field
180958	2018	Sichuan	Wheat	*F. asiaticum*	NIV	29.38913802	104.432672	461.83255	Commercialized variety	Farmer owned field
180957	2018	Sichuan	Wheat	*F. asiaticum*	NIV	29.38913802	104.432672	461.83255	Commercialized variety	Farmer owned field
180968	2018	Sichuan	Wheat	*F. asiaticum*	NIV	29.39178402	104.430066	454.927856	Commercialized variety	Farmer owned field
172123	2017	Sichuan	Wheat	*F. asiaticum*	NIV	29.590293	104.975397	328	Commercialized variety	Farmer owned field
172122	2017	Sichuan	Wheat	*F. asiaticum*	NIV	29.590293	104.975397	328	Commercialized variety	Farmer owned field
171670	2017	Sichuan	Rice	*F. asiaticum*	NIV	29.618008	105.025732	305.3		Farmer owned field
171669	2017	Sichuan	Rice	*F. asiaticum*	NIV	29.618008	105.025732	305.3		Farmer owned field
171668	2017	Sichuan	Rice	*F. asiaticum*	NIV	29.618008	105.025732	305.3		Farmer owned field
171666	2017	Sichuan	Rice	*F. asiaticum*	NIV	29.618008	105.025732	305.3		Farmer owned field
171665	2017	Sichuan	Rice	*F. asiaticum*	NIV	29.618008	105.025732	305.3		Farmer owned field
171664	2017	Sichuan	Rice	*F. asiaticum*	NIV	29.618008	105.025732	305.3		Farmer owned field
171663	2017	Sichuan	Rice	*F. asiaticum*	NIV	29.618008	105.025732	305.3		Farmer owned field
171662	2017	Sichuan	Rice	*F. asiaticum*	NIV	29.618008	105.025732	305.3		Farmer owned field
171661	2017	Sichuan	Rice	*F. asiaticum*	3ADON	29.618008	105.025732	305.3		Farmer owned field
171660	2017	Sichuan	Rice	*F. asiaticum*	NIV	29.618008	105.025732	305.3		Farmer owned field
171659	2017	Sichuan	Rice	*F. asiaticum*	NIV	29.618008	105.025732	305.3		Farmer owned field
171658	2017	Sichuan	Rice	*F. asiaticum*	NIV	29.618008	105.025732	305.3		Farmer owned field
180555	2018	Hubei	Rice	*F. asiaticum*	15ADON	29.88329496	111.903684	41.798851		Farmer owned field
180554	2018	Hubei	Rice	*F. asiaticum*	15ADON	29.88329496	111.903684	41.798851		Farmer owned field
180553	2018	Hubei	Rice	*F. asiaticum*	15ADON	29.88329496	111.903684	41.798851		Farmer owned field
180552	2018	Hubei	Rice	*F. asiaticum*	NIV	29.88329496	111.903684	41.798851		Farmer owned field
180551	2018	Hubei	Rice	*F. asiaticum*	15ADON	29.88329496	111.903684	41.798851		Farmer owned field
180550	2018	Hubei	Rice	*F. asiaticum*	15ADON	29.88329496	111.903684	41.798851		Farmer owned field

180548	2018	Hubei	Rice	*F. asiaticum*	15ADON	29.88329496	111.903684	41.798851	Farmer owned field
180547	2018	Hubei	Rice	*F. asiaticum*	NIV	29.88329496	111.903684	41.798851	Farmer owned field
180546	2018	Hubei	Rice	*F. asiaticum*	15ADON	29.88329496	111.903684	41.798851	Farmer owned field
180545	2018	Hubei	Rice	*F. asiaticum*	NIV	29.88329496	111.903684	41.798851	Farmer owned field
180544	2018	Hubei	Rice	*F. asiaticum*	3ADON	29.88329496	111.903684	41.798851	Farmer owned field
180543	2018	Hubei	Rice	*F. asiaticum*	15ADON	29.88329496	111.903684	41.798851	Farmer owned field
180542	2018	Hubei	Rice	*F. asiaticum*	15ADON	29.88329496	111.903684	41.798851	Farmer owned field
180541	2018	Hubei	Rice	*F. asiaticum*	3ADON	29.88329496	111.903684	41.798851	Farmer owned field
180540	2018	Hubei	Rice	*F. asiaticum*	3ADON	29.88329496	111.903684	41.798851	Farmer owned field
180539	2018	Hubei	Rice	*F. asiaticum*	NIV	29.88329496	111.903684	41.798851	Farmer owned field
180538	2018	Hubei	Rice	*F. asiaticum*	NIV	29.88329496	111.903684	41.798851	Farmer owned field
180537	2018	Hubei	Rice	*F. asiaticum*	15ADON	29.88329496	111.903684	41.798851	Farmer owned field
180380	2018	Hubei	Rice	*F. asiaticum*	NIV	29.90943502	114.504874	44.087009	Farmer owned field
180379	2018	Hubei	Rice	*F. asiaticum*	NIV	29.90943502	114.504874	44.087009	Farmer owned field
180378	2018	Hubei	Rice	*F. asiaticum*	3ADON	29.90943502	114.504874	44.087009	Farmer owned field
180376	2018	Hubei	Rice	*F. asiaticum*	NIV	29.90943502	114.504874	44.087009	Farmer owned field
180375	2018	Hubei	Rice	*F. asiaticum*	NIV	29.90943502	114.504874	44.087009	Farmer owned field
180374	2018	Hubei	Rice	*F. asiaticum*	NIV	29.90943502	114.504874	44.087009	Farmer owned field
180373	2018	Hubei	Rice	*F. asiaticum*	NIV	29.90943502	114.504874	44.087009	Farmer owned field
180372	2018	Hubei	Rice	*F. asiaticum*	NIV	29.90943502	114.504874	44.087009	Farmer owned field
180371	2018	Hubei	Rice	*F. asiaticum*	NIV	29.90943502	114.504874	44.087009	Farmer owned field
180368	2018	Hubei	Rice	*F. asiaticum*	NIV	29.90943502	114.504874	44.087009	Farmer owned field
180367	2018	Hubei	Rice	*F. asiaticum*	NIV	29.90943502	114.504874	44.087009	Farmer owned field
180366	2018	Hubei	Rice	*F. asiaticum*	NIV	29.90943502	114.504874	44.087009	Farmer owned field
171657	2017	Sichuan	Rice	*F. asiaticum*	NIV	29.618008	105.025732	305.3	Farmer owned field
171656	2017	Sichuan	Rice	*F. asiaticum*	NIV	29.618008	105.025732	305.3	Farmer owned field
171654	2017	Sichuan	Rice	*F. asiaticum*	NIV	29.618008	105.025732	305.3	Farmer owned field
171653	2017	Sichuan	Rice	*F. asiaticum*	NIV	29.618008	105.025732	305.3	Farmer owned field
171652	2017	Sichuan	Rice	*F. asiaticum*	NIV	29.618008	105.025732	305.3	Farmer owned field
171651	2017	Sichuan	Rice	*F. asiaticum*	NIV	29.618008	105.025732	305.3	Farmer owned field
171650	2017	Sichuan	Rice	*F. asiaticum*	NIV	29.618008	105.025732	305.3	Farmer owned field
171649	2017	Sichuan	Rice	*F. asiaticum*	NIV	29.618008	105.025732	305.3	Farmer owned field
171648	2017	Sichuan	Rice	*F. asiaticum*	NIV	29.618008	105.025732	305.3	Farmer owned field
171647	2017	Sichuan	Rice	*F. asiaticum*	NIV	29.618008	105.025732	305.3	Farmer owned field
171646	2017	Sichuan	Rice	*F. asiaticum*	NIV	29.618008	105.025732	305.3	Farmer owned field

171645	2017	Sichuan	Rice	*F. asiaticum*	NIV	29.618008	105.025732	305.3		Farmer owned field
171644	2017	Sichuan	Rice	*F. asiaticum*	NIV	29.618008	105.025732	305.3		Farmer owned field
171643	2017	Sichuan	Rice	*F. asiaticum*	NIV	29.618008	105.025732	305.3		Farmer owned field
171642	2017	Sichuan	Rice	*F. asiaticum*	NIV	29.618008	105.025732	305.3		Farmer owned field
171641	2017	Sichuan	Rice	*F. asiaticum*	NIV	29.618008	105.025732	305.3		Farmer owned field
171640	2017	Sichuan	Rice	*F. asiaticum*	NIV	29.618008	105.025732	305.3		Farmer owned field
171639	2017	Sichuan	Rice	*F. asiaticum*	NIV	29.618008	105.025732	305.3		Farmer owned field
180956	2018	Sichuan	Wheat	*F. asiaticum*	NIV	30.06710398	104.222467	419.035309	Commercialized variety	Farmer owned field
180952	2018	Sichuan	Wheat	*F. asiaticum*	NIV	30.06710398	104.222467	419.035309	Commercialized variety	Farmer owned field
180951	2018	Sichuan	Wheat	*F. asiaticum*	NIV	30.06710398	104.222467	419.035309	Commercialized variety	Farmer owned field
180950	2018	Sichuan	Wheat	*F. asiaticum*	NIV	30.06710398	104.222467	419.035309	Commercialized variety	Farmer owned field
180923	2018	Sichuan	Wheat	*F. asiaticum*	NIV	30.06710398	104.222467	419.035309	Commercialized variety	Farmer owned field
180922	2018	Sichuan	Wheat	*F. asiaticum*	NIV	30.06710398	104.222467	419.035309	Commercialized variety	Farmer owned field
180921	2018	Sichuan	Wheat	*F. asiaticum*	NIV	30.06710398	104.222467	419.035309	Commercialized variety	Farmer owned field
180920	2018	Sichuan	Wheat	*F. asiaticum*	NIV	30.06710398	104.222467	419.035309	Commercialized variety	Farmer owned field
180917	2018	Sichuan	Wheat	*F. asiaticum*	NIV	30.06710398	104.222467	419.035309	Commercialized variety	Farmer owned field
180914	2018	Sichuan	Wheat	*F. asiaticum*	NIV	30.06710398	104.222467	419.035309	Commercialized variety	Farmer owned field
180913	2018	Sichuan	Wheat	*F. asiaticum*	NIV	30.06710398	104.222467	419.035309	Commercialized variety	Farmer owned field
180912	2018	Sichuan	Wheat	*F. asiaticum*	NIV	30.06710398	104.222467	419.035309	Commercialized variety	Farmer owned field
180911	2018	Sichuan	Wheat	*F. asiaticum*	NIV	30.06710398	104.222467	419.035309	Commercialized variety	Farmer owned field
180910	2018	Sichuan	Wheat	*F. asiaticum*	NIV	30.06710398	104.222467	419.035309	Commercialized variety	Farmer owned field
180909	2018	Sichuan	Wheat	*F. asiaticum*	NIV	30.06710398	104.222467	419.035309	Commercialized variety	Farmer owned field
180908	2018	Sichuan	Wheat	*F. asiaticum*	NIV	30.06710398	104.222467	419.035309	Commercialized variety	Farmer owned field
180907	2018	Sichuan	Wheat	*F. asiaticum*	NIV	30.06710398	104.222467	419.035309	Commercialized variety	Farmer owned field
191481	2019	Anhui	Wheat	*F. asiaticum*	NIV	30.14408296	118.739256	297.216919	Commercialized variety	Farmer owned field
191478	2019	Anhui	Wheat	*F. asiaticum*	15ADON	30.14408296	118.739256	297.216919	Commercialized variety	Farmer owned field
191477	2019	Anhui	Wheat	*F. asiaticum*	NIV	30.14408296	118.739256	297.216919	Commercialized variety	Farmer owned field
191476	2019	Anhui	Wheat	*F. asiaticum*	NIV	30.14408296	118.739256	297.216919	Commercialized variety	Farmer owned field
191475	2019	Anhui	Wheat	*F. asiaticum*	NIV	30.14408296	118.739256	297.216919	Commercialized variety	Farmer owned field
191474	2019	Anhui	Wheat	*F. asiaticum*	NIV	30.14408296	118.739256	297.216919	Commercialized variety	Farmer owned field
191473	2019	Anhui	Wheat	*F. asiaticum*	NIV	30.14408296	118.739256	297.216919	Commercialized variety	Farmer owned field
191471	2019	Anhui	Wheat	*F. asiaticum*	NIV	30.14408296	118.739256	297.216919	Commercialized variety	Farmer owned field
191468	2019	Anhui	Wheat	*F. asiaticum*	NIV	30.14408296	118.739256	297.216919	Commercialized variety	Farmer owned field
191467	2019	Anhui	Wheat	*F. asiaticum*	NIV	30.14408296	118.739256	297.216919	Commercialized variety	Farmer owned field
191466	2019	Anhui	Wheat	*F. asiaticum*	NIV	30.14408296	118.739256	297.216919	Commercialized variety	Farmer owned field

191465	2019	Anhui	Wheat	*F. asiaticum*	15ADON	30.14408296	118.739256	297.216919	Commercialized variety	Farmer owned field
191464	2019	Anhui	Wheat	*F. asiaticum*	NIV	30.14408296	118.739256	297.216919	Commercialized variety	Farmer owned field
191462	2019	Anhui	Wheat	*F. asiaticum*	NIV	30.14408296	118.739256	297.216919	Commercialized variety	Farmer owned field
191460	2019	Anhui	Wheat	*F. asiaticum*	3ADON	30.14408296	118.739256	297.216919	Commercialized variety	Farmer owned field
191459	2019	Anhui	Wheat	*F. asiaticum*	NIV	30.14408296	118.739256	297.216919	Commercialized variety	Farmer owned field
191458	2019	Anhui	Wheat	*F. asiaticum*	NIV	30.14408296	118.739256	297.216919	Commercialized variety	Farmer owned field
191457	2019	Anhui	Wheat	*F. asiaticum*	NIV	30.14408296	118.739256	297.216919	Commercialized variety	Farmer owned field
191454	2019	Anhui	Wheat	*F. asiaticum*	NIV	30.14408296	118.739256	297.216919	Commercialized variety	Farmer owned field
191453	2019	Anhui	Wheat	*F. asiaticum*	NIV	30.14408296	118.739256	297.216919	Commercialized variety	Farmer owned field
190043	2019	Anhui	Wheat	*F. asiaticum*	3ADON	30.19380701	118.865659	650.646667	Commercialized variety	Farmer owned field
194025	2019	Anhui	Rice	*F. asiaticum*	3ADON	30.19380701	118.865659	650.646667		Farmer owned field
194024	2019	Anhui	Rice	*F. asiaticum*	3ADON	30.19380701	118.865659	650.646667		Farmer owned field
194023	2019	Anhui	Rice	*F. asiaticum*	NIV	30.19380701	118.865659	650.646667		Farmer owned field
194022	2019	Anhui	Rice	*F. asiaticum*	NIV	30.19380701	118.865659	650.646667		Farmer owned field
194021	2019	Anhui	Rice	*F. asiaticum*	NIV	30.19380701	118.865659	650.646667		Farmer owned field
194020	2019	Anhui	Rice	*F. asiaticum*	NIV	30.19380701	118.865659	650.646667		Farmer owned field
194019	2019	Anhui	Rice	*F. asiaticum*	NIV	30.19380701	118.865659	650.646667		Farmer owned field
194017	2019	Anhui	Rice	*F. asiaticum*	NIV	30.19380701	118.865659	650.646667		Farmer owned field
194016	2019	Anhui	Rice	*F. asiaticum*	NIV	30.19380701	118.865659	650.646667		Farmer owned field
194015	2019	Anhui	Rice	*F. asiaticum*	NIV	30.19380701	118.865659	650.646667		Farmer owned field
194014	2019	Anhui	Rice	*F. asiaticum*	NIV	30.19380701	118.865659	650.646667		Farmer owned field
194013	2019	Anhui	Rice	*F. asiaticum*	NIV	30.19380701	118.865659	650.646667		Farmer owned field
194012	2019	Anhui	Rice	*F. asiaticum*	NIV	30.19380701	118.865659	650.646667		Farmer owned field
194011	2019	Anhui	Rice	*F. asiaticum*	NIV	30.19380701	118.865659	650.646667		Farmer owned field
194010	2019	Anhui	Rice	*F. asiaticum*	NIV	30.19380701	118.865659	650.646667		Farmer owned field
194009	2019	Anhui	Rice	*F. asiaticum*	NIV	30.19380701	118.865659	650.646667		Farmer owned field
194008	2019	Anhui	Rice	*F. asiaticum*	NIV	30.19380701	118.865659	650.646667		Farmer owned field
194007	2019	Anhui	Rice	*F. asiaticum*	NIV	30.19380701	118.865659	650.646667		Farmer owned field
194006	2019	Anhui	Rice	*F. asiaticum*	NIV	30.19380701	118.865659	650.646667		Farmer owned field
194005	2019	Anhui	Rice	*F. asiaticum*	NIV	30.19380701	118.865659	650.646667		Farmer owned field
194004	2019	Anhui	Rice	*F. asiaticum*	NIV	30.19380701	118.865659	650.646667		Farmer owned field
194003	2019	Anhui	Rice	*F. asiaticum*	NIV	30.19380701	118.865659	650.646667		Farmer owned field
194002	2019	Anhui	Rice	*F. asiaticum*	NIV	30.19380701	118.865659	650.646667		Farmer owned field
160205	2016	Anhui	Rice	*F. asiaticum*	3ADON	30.6542879	117.4921052	14		Farmer owned field
191545	2019	Anhui	Wheat	*F. asiaticum*	3ADON	30.65441396	117.43052	34.784958	Commercialized variety	Farmer owned field

191544	2019	Anhui	Wheat	*F. asiaticum*	3ADON	30.65441396	117.43052	34.784958	Commercialized variety	Farmer owned field
191542	2019	Anhui	Wheat	*F. asiaticum*	15ADON	30.65441396	117.43052	34.784958	Commercialized variety	Farmer owned field
191541	2019	Anhui	Wheat	*F. asiaticum*	15ADON	30.65441396	117.43052	34.784958	Commercialized variety	Farmer owned field
191540	2019	Anhui	Wheat	*F. asiaticum*	3ADON	30.65441396	117.43052	34.784958	Commercialized variety	Farmer owned field
191539	2019	Anhui	Wheat	*F. asiaticum*	3ADON	30.65441396	117.43052	34.784958	Commercialized variety	Farmer owned field
191538	2019	Anhui	Wheat	*F. asiaticum*	3ADON	30.65441396	117.43052	34.784958	Commercialized variety	Farmer owned field
191537	2019	Anhui	Wheat	*F. asiaticum*	3ADON	30.65441396	117.43052	34.784958	Commercialized variety	Farmer owned field
180904	2018	Sichuan	Wheat	*F. asiaticum*	NIV	30.06710398	104.222467	419.035309	Commercialized variety	Farmer owned field
180900	2018	Sichuan	Wheat	*F. asiaticum*	NIV	30.06710398	104.222467	419.035309	Commercialized variety	Farmer owned field
194674	2019	Sichuan	Wheat	*F. asiaticum*	NIV	30.3333	103.3835	NA	Commercialized variety	Farmer owned field
194673	2019	Sichuan	Wheat	*F. asiaticum*	NIV	30.3333	103.3835	NA	Commercialized variety	Farmer owned field
194672	2019	Sichuan	Wheat	*F. asiaticum*	NIV	30.3333	103.3835	NA	Commercialized variety	Farmer owned field
194671	2019	Sichuan	Wheat	*F. asiaticum*	NIV	30.3333	103.3835	NA	Commercialized variety	Farmer owned field
194758	2019	Sichuan	Rice	*F. asiaticum*	NIV	30.392	103.3443	NA		Farmer owned field
194754	2019	Sichuan	Rice	*F. asiaticum*	NIV	30.392	103.3443	NA		Farmer owned field
194752	2019	Sichuan	Rice	*F. asiaticum*	NIV	30.392	103.3443	NA		Farmer owned field
194748	2019	Sichuan	Rice	*F. asiaticum*	NIV	30.392	103.3443	NA		Farmer owned field
SCJY2061	2014	Sichuan	Rice	*F. asiaticum*	NIV	30.446597	104.525323	384		Farmer owned field
SCJY2059	2014	Sichuan	Rice	*F. asiaticum*	NIV	30.446597	104.525323	384		Farmer owned field
SCJY2058	2014	Sichuan	Rice	*F. asiaticum*	NIV	30.446597	104.525323	384		Farmer owned field
SCJY2057	2014	Sichuan	Rice	*F. asiaticum*	NIV	30.446597	104.525323	384		Farmer owned field
SCJY2055	2014	Sichuan	Rice	*F. asiaticum*	NIV	30.446597	104.525323	384		Farmer owned field
191536	2019	Anhui	Wheat	*F. asiaticum*	3ADON	30.65441396	117.43052	34.784958	Commercialized variety	Farmer owned field
191452	2019	Anhui	Wheat	*F. asiaticum*	3ADON	30.65441396	117.43052	34.784958	Commercialized variety	Farmer owned field
191451	2019	Anhui	Wheat	*F. asiaticum*	3ADON	30.65441396	117.43052	34.784958	Commercialized variety	Farmer owned field
191450	2019	Anhui	Wheat	*F. asiaticum*	3ADON	30.65441396	117.43052	34.784958	Commercialized variety	Farmer owned field
191449	2019	Anhui	Wheat	*F. asiaticum*	3ADON	30.65441396	117.43052	34.784958	Commercialized variety	Farmer owned field
191448	2019	Anhui	Wheat	*F. asiaticum*	NIV	30.65441396	117.43052	34.784958	Commercialized variety	Farmer owned field
191447	2019	Anhui	Wheat	*F. asiaticum*	3ADON	30.65441396	117.43052	34.784958	Commercialized variety	Farmer owned field
191446	2019	Anhui	Wheat	*F. asiaticum*	NIV	30.65441396	117.43052	34.784958	Commercialized variety	Farmer owned field
191445	2019	Anhui	Wheat	*F. asiaticum*	3ADON	30.65441396	117.43052	34.784958	Commercialized variety	Farmer owned field
191444	2019	Anhui	Wheat	*F. asiaticum*	3ADON	30.65441396	117.43052	34.784958	Commercialized variety	Farmer owned field
191443	2019	Anhui	Wheat	*F. asiaticum*	3ADON	30.65441396	117.43052	34.784958	Commercialized variety	Farmer owned field
192081	2019	Anhui	Wheat	*F. asiaticum*	NIV	30.67082598	117.365424	37.724396	Commercialized variety	Farmer owned field
192080	2019	Anhui	Wheat	*F. asiaticum*	3ADON	30.67082598	117.365424	37.724396	Commercialized variety	Farmer owned field

192078	2019	Anhui	Wheat	*F. asiaticum*	3ADON	30.67082598	117.365424	37.724396	Commercialized variety	Farmer owned field
192077	2019	Anhui	Wheat	*F. asiaticum*	3ADON	30.67082598	117.365424	37.724396	Commercialized variety	Farmer owned field
192076	2019	Anhui	Wheat	*F. asiaticum*	3ADON	30.67082598	117.365424	37.724396	Commercialized variety	Farmer owned field
192075	2019	Anhui	Wheat	*F. asiaticum*	3ADON	30.67082598	117.365424	37.724396	Commercialized variety	Farmer owned field
192074	2019	Anhui	Wheat	*F. asiaticum*	NIV	30.67082598	117.365424	37.724396	Commercialized variety	Farmer owned field
192073	2019	Anhui	Wheat	*F. asiaticum*	NIV	30.67082598	117.365424	37.724396	Commercialized variety	Farmer owned field
192072	2019	Anhui	Wheat	*F. asiaticum*	3ADON	30.67082598	117.365424	37.724396	Commercialized variety	Farmer owned field
192071	2019	Anhui	Wheat	*F. asiaticum*	NIV	30.67082598	117.365424	37.724396	Commercialized variety	Farmer owned field
192070	2019	Anhui	Wheat	*F. asiaticum*	3ADON	30.67082598	117.365424	37.724396	Commercialized variety	Farmer owned field
192069	2019	Anhui	Wheat	*F. asiaticum*	3ADON	30.67082598	117.365424	37.724396	Commercialized variety	Farmer owned field
192068	2019	Anhui	Wheat	*F. asiaticum*	3ADON	30.67082598	117.365424	37.724396	Commercialized variety	Farmer owned field
192067	2019	Anhui	Wheat	*F. asiaticum*	3ADON	30.67082598	117.365424	37.724396	Commercialized variety	Farmer owned field
192066	2019	Anhui	Wheat	*F. asiaticum*	3ADON	30.67082598	117.365424	37.724396	Commercialized variety	Farmer owned field
192065	2019	Anhui	Wheat	*F. asiaticum*	3ADON	30.67082598	117.365424	37.724396	Commercialized variety	Farmer owned field
192064	2019	Anhui	Wheat	*F. asiaticum*	3ADON	30.67082598	117.365424	37.724396	Commercialized variety	Farmer owned field
192063	2019	Anhui	Wheat	*F. asiaticum*	NIV	30.67082598	117.365424	37.724396	Commercialized variety	Farmer owned field
192062	2019	Anhui	Wheat	*F. asiaticum*	NIV	30.67082598	117.365424	37.724396	Commercialized variety	Farmer owned field
193974	2019	Jiangsu	Rice	*F. asiaticum*	15ADON	28.62087197	112.339678	77.464882		Farmer owned field
193972	2019	Jiangsu	Rice	*F. asiaticum*	NIV	28.62087197	112.339678	77.464882		Farmer owned field
193970	2019	Jiangsu	Rice	*F. asiaticum*	15ADON	28.62087197	112.339678	77.464882		Farmer owned field
193969	2019	Jiangsu	Rice	*F. asiaticum*	15ADON	28.62087197	112.339678	77.464882		Farmer owned field
193968	2019	Jiangsu	Rice	*F. asiaticum*	NIV	28.62087197	112.339678	77.464882		Farmer owned field
193966	2019	Jiangsu	Rice	*F. asiaticum*	NIV	28.62087197	112.339678	77.464882		Farmer owned field
193965	2019	Jiangsu	Rice	*F. asiaticum*	3ADON	28.62087197	112.339678	77.464882		Farmer owned field
193963	2019	Jiangsu	Rice	*F. asiaticum*	NIV	28.62087197	112.339678	77.464882		Farmer owned field
193961	2019	Jiangsu	Rice	*F. asiaticum*	3ADON	28.62087197	112.339678	77.464882		Farmer owned field
193959	2019	Jiangsu	Rice	*F. asiaticum*	NIV	28.62087197	112.339678	77.464882		Farmer owned field
193956	2019	Jiangsu	Rice	*F. asiaticum*	3ADON	28.62087197	112.339678	77.464882		Farmer owned field
193955	2019	Jiangsu	Rice	*F. asiaticum*	NIV	28.62087197	112.339678	77.464882		Farmer owned field
193954	2019	Jiangsu	Rice	*F. asiaticum*	3ADON	28.62087197	112.339678	77.464882		Farmer owned field
193953	2019	Jiangsu	Rice	*F. asiaticum*	3ADON	28.62087197	112.339678	77.464882		Farmer owned field
193952	2019	Jiangsu	Rice	*F. asiaticum*	3ADON	28.62087197	112.339678	77.464882		Farmer owned field
193951	2019	Jiangsu	Rice	*F. asiaticum*	3ADON	28.62087197	112.339678	77.464882		Farmer owned field
193950	2019	Jiangsu	Rice	*F. asiaticum*	3ADON	28.62087197	112.339678	77.464882		Farmer owned field
193949	2019	Jiangsu	Rice	*F. asiaticum*	3ADON	28.62087197	112.339678	77.464882		Farmer owned field

160863	2016	Jiangsu	Rice	*F. asiaticum*	3ADON	31.457735	121.13055	4		Farmer owned field
192133	2019	Jiangsu	Wheat	*F. asiaticum*	3ADON	31.72359603	118.91675	17.568762	Commercialized variety	Farmer owned field
192132	2019	Jiangsu	Wheat	*F. asiaticum*	3ADON	31.72359603	118.91675	17.568762	Commercialized variety	Farmer owned field
192131	2019	Jiangsu	Wheat	*F. asiaticum*	3ADON	31.72359603	118.91675	17.568762	Commercialized variety	Farmer owned field
192130	2019	Jiangsu	Wheat	*F. asiaticum*	3ADON	31.72359603	118.91675	17.568762	Commercialized variety	Farmer owned field
192129	2019	Jiangsu	Wheat	*F. asiaticum*	3ADON	31.72359603	118.91675	17.568762	Commercialized variety	Farmer owned field
192128	2019	Jiangsu	Wheat	*F. asiaticum*	3ADON	31.72359603	118.91675	17.568762	Commercialized variety	Farmer owned field
192127	2019	Jiangsu	Wheat	*F. asiaticum*	3ADON	31.72359603	118.91675	17.568762	Commercialized variety	Farmer owned field
192126	2019	Jiangsu	Wheat	*F. asiaticum*	3ADON	31.72359603	118.91675	17.568762	Commercialized variety	Farmer owned field
192125	2019	Jiangsu	Wheat	*F. asiaticum*	3ADON	31.72359603	118.91675	17.568762	Commercialized variety	Farmer owned field
192124	2019	Jiangsu	Wheat	*F. asiaticum*	3ADON	31.72359603	118.91675	17.568762	Commercialized variety	Farmer owned field
160898	2016	Jiangsu	Rice	*F. asiaticum*	3ADON	31.982751	120.277138	2		Farmer owned field
JSNTRG051	2014	Jiangsu	Rice	*F. asiaticum*	3ADON	32.054126	118.972022	28		Commercial farm
JSNTRG050	2014	Jiangsu	Rice	*F. asiaticum*	3ADON	32.054126	118.972022	28		Commercial farm
JSNTRG048-2	2014	Jiangsu	Rice	*F. asiaticum*	3ADON	32.054126	118.972022	28		Commercial farm
JSNTRG047	2014	Jiangsu	Rice	*F. asiaticum*	3ADON	32.054126	118.972022	28		Commercial farm
JSNTRG046	2014	Jiangsu	Rice	*F. asiaticum*	3ADON	32.054126	118.972022	28		Commercial farm
JSNTRG044-2	2014	Jiangsu	Rice	*F. asiaticum*	NIV	32.054126	118.972022	28		Commercial farm
JSNTRG044	2014	Jiangsu	Rice	*F. asiaticum*	3ADON	32.054126	118.972022	28		Commercial farm
JSNTRG043-2	2014	Jiangsu	Rice	*F. asiaticum*	NIV	32.054126	118.972022	28		Commercial farm
JSNTRG042-2	2014	Jiangsu	Rice	*F. asiaticum*	3ADON	32.054126	118.972022	28		Commercial farm
JSNTRG041	2014	Jiangsu	Rice	*F. asiaticum*	3ADON	32.054126	118.972022	28		Commercial farm
JSNTRG040	2014	Jiangsu	Rice	*F. asiaticum*	3ADON	32.054126	118.972022	28		Commercial farm
JSNTRG039-2	2014	Jiangsu	Rice	*F. asiaticum*	3ADON	32.054126	118.972022	28		Commercial farm
JSNTRG039	2014	Jiangsu	Rice	*F. asiaticum*	3ADON	32.054126	118.972022	28		Commercial farm
JSNTRG038-2	2014	Jiangsu	Rice	*F. asiaticum*	NIV	32.054126	118.972022	28		Commercial farm
JSNTRG035-2	2014	Jiangsu	Rice	*F. asiaticum*	NIV	32.054126	118.972022	28		Commercial farm
JSNTRG034-2	2014	Jiangsu	Rice	*F. asiaticum*	NIV	32.054126	118.972022	28		Commercial farm
JSNTRG031-2	2014	Jiangsu	Rice	*F. asiaticum*	3ADON	32.054126	118.972022	28		Commercial farm
JSNTRG031-1	2014	Jiangsu	Rice	*F. asiaticum*	NIV	32.054126	118.972022	28		Commercial farm
JSNTRG030-2	2014	Jiangsu	Rice	*F. asiaticum*	3ADON	32.054126	118.972022	28		Commercial farm
JSNTRG030-1	2014	Jiangsu	Rice	*F. asiaticum*	NIV	32.054126	118.972022	28		Commercial farm
JSNTRG029	2014	Jiangsu	Rice	*F. asiaticum*	3ADON	32.054126	118.972022	28		Commercial farm
JSNTRG026	2014	Jiangsu	Rice	*F. asiaticum*	3ADON	32.054126	118.972022	28		Commercial farm
JSNTRG025	2014	Jiangsu	Rice	*F. asiaticum*	3ADON	32.054126	118.972022	28		Commercial farm

JSNTRG020	2014	Jiangsu	Rice	*F. asiaticum*	3ADON	32.054126	118.972022	28		Commercial farm
JSNTRG019-3	2014	Jiangsu	Rice	*F. asiaticum*	3ADON	32.054126	118.972022	28		Commercial farm
JSNTRG017	2014	Jiangsu	Rice	*F. asiaticum*	3ADON	32.054126	118.972022	28		Commercial farm
JSNTRG016-2	2014	Jiangsu	Rice	*F. asiaticum*	3ADON	32.054126	118.972022	28		Commercial farm
JSNTRG010	2014	Jiangsu	Rice	*F. asiaticum*	3ADON	32.054126	118.972022	28		Commercial farm
JSNTRG009	2014	Jiangsu	Rice	*F. asiaticum*	3ADON	32.054126	118.972022	28		Commercial farm
JSNTRG008	2014	Jiangsu	Rice	*F. asiaticum*	3ADON	32.054126	118.972022	28		Commercial farm
JSNTRG007	2014	Jiangsu	Rice	*F. asiaticum*	3ADON	32.054126	118.972022	28		Commercial farm
JSNTRG005	2014	Jiangsu	Rice	*F. asiaticum*	3ADON	32.054126	118.972022	28		Commercial farm
JSNTRG003	2014	Jiangsu	Rice	*F. asiaticum*	3ADON	32.054126	118.972022	28		Commercial farm
JSNTRG002	2014	Jiangsu	Rice	*F. asiaticum*	NIV	32.054126	118.972022	28		Commercial farm
JSNTRG001	2014	Jiangsu	Rice	*F. asiaticum*	NIV	32.054126	118.972022	28		Commercial farm
JSNJ038	2014	Jiangsu	Rice	*F. asiaticum*	3ADON	32.054126	118.972022	28		Commercial farm
JSNJ035	2014	Jiangsu	Rice	*F. asiaticum*	3ADON	32.054126	118.972022	28		Commercial farm
JSNJ027	2014	Jiangsu	Rice	*F. asiaticum*	3ADON	32.054126	118.972022	28		Commercial farm
JSNJ018	2014	Jiangsu	Rice	*F. asiaticum*	NIV	32.054126	118.972022	28		Commercial farm
JSNJ015	2014	Jiangsu	Rice	*F. asiaticum*	3ADON	32.054126	118.972022	28		Commercial farm
192865	2019	Jiangsu	Wheat	*F. asiaticum*	NIV	32.05939201	119.192636	117.100487	Commercialized variety	Farmer owned field
192864	2019	Jiangsu	Wheat	*F. asiaticum*	3ADON	32.05939201	119.192636	117.100487	Commercialized variety	Farmer owned field
192863	2019	Jiangsu	Wheat	*F. asiaticum*	3ADON	32.05939201	119.192636	117.100487	Commercialized variety	Farmer owned field
192862	2019	Jiangsu	Wheat	*F. asiaticum*	3ADON	32.05939201	119.192636	117.100487	Commercialized variety	Farmer owned field
192860	2019	Jiangsu	Wheat	*F. asiaticum*	3ADON	32.05939201	119.192636	117.100487	Commercialized variety	Farmer owned field
192859	2019	Jiangsu	Wheat	*F. asiaticum*	NIV	32.05939201	119.192636	117.100487	Commercialized variety	Farmer owned field
192857	2019	Jiangsu	Wheat	*F. asiaticum*	3ADON	32.05939201	119.192636	117.100487	Commercialized variety	Farmer owned field
192856	2019	Jiangsu	Wheat	*F. asiaticum*	3ADON	32.05939201	119.192636	117.100487	Commercialized variety	Farmer owned field
192855	2019	Jiangsu	Wheat	*F. asiaticum*	3ADON	32.05939201	119.192636	117.100487	Commercialized variety	Farmer owned field
192854	2019	Jiangsu	Wheat	*F. asiaticum*	3ADON	32.05939201	119.192636	117.100487	Commercialized variety	Farmer owned field
192853	2019	Jiangsu	Wheat	*F. asiaticum*	NIV	32.05939201	119.192636	117.100487	Commercialized variety	Farmer owned field
192852	2019	Jiangsu	Wheat	*F. asiaticum*	3ADON	32.05939201	119.192636	117.100487	Commercialized variety	Farmer owned field
192850	2019	Jiangsu	Wheat	*F. asiaticum*	3ADON	32.05939201	119.192636	117.100487	Commercialized variety	Farmer owned field
192849	2019	Jiangsu	Wheat	*F. asiaticum*	3ADON	32.05939201	119.192636	117.100487	Commercialized variety	Farmer owned field
192848	2019	Jiangsu	Wheat	*F. asiaticum*	3ADON	32.05939201	119.192636	117.100487	Commercialized variety	Farmer owned field
192847	2019	Jiangsu	Wheat	*F. asiaticum*	NIV	32.05939201	119.192636	117.100487	Commercialized variety	Farmer owned field
192846	2019	Jiangsu	Wheat	*F. asiaticum*	3ADON	32.05939201	119.192636	117.100487	Commercialized variety	Farmer owned field
192845	2019	Jiangsu	Wheat	*F. asiaticum*	3ADON	32.05939201	119.192636	117.100487	Commercialized variety	Farmer owned field

171552	2017	Jiangsu	Rice	*F. asiaticum*	3ADON	32.24675403	120.153571	NA		Farmer owned field
171551	2017	Jiangsu	Rice	*F. asiaticum*	3ADON	32.24675403	120.153571	NA		Farmer owned field
171550	2017	Jiangsu	Rice	*F. asiaticum*	3ADON	32.24675403	120.153571	NA		Farmer owned field
192941	2019	Jiangsu	Wheat	*F. asiaticum*	3ADON	32.25813196	119.375347	74.785568	Commercialized variety	Farmer owned field
192935	2019	Jiangsu	Wheat	*F. asiaticum*	3ADON	32.25813196	119.375347	74.785568	Commercialized variety	Farmer owned field
JSYZ112	2014	Jiangsu	Rice	*F. asiaticum*	NIV	32.270763	119.254133	4		Commercial farm
JSYZ110	2014	Jiangsu	Rice	*F. asiaticum*	3ADON	32.270763	119.254133	4		Commercial farm
JSYZ108	2014	Jiangsu	Rice	*F. asiaticum*	3ADON	32.270763	119.254133	4		Commercial farm
JSYZ105	2014	Jiangsu	Rice	*F. asiaticum*	15ADON	32.270763	119.254133	4		Commercial farm
JSYZ102	2014	Jiangsu	Rice	*F. asiaticum*	3ADON	32.270763	119.254133	4		Commercial farm
JSYZ101	2014	Jiangsu	Rice	*F. asiaticum*	3ADON	32.270763	119.254133	4		Commercial farm
JSYZ098	2014	Jiangsu	Rice	*F. asiaticum*	3ADON	32.270763	119.254133	4		Commercial farm
JSYZ097	2014	Jiangsu	Rice	*F. asiaticum*	NIV	32.270763	119.254133	4		Commercial farm
180365	2018	Hubei	Rice	*F. asiaticum*	NIV	29.90943502	114.504874	44.087009		Farmer owned field
180364	2018	Hubei	Rice	*F. asiaticum*	NIV	29.90943502	114.504874	44.087009		Farmer owned field
180363	2018	Hubei	Rice	*F. asiaticum*	3ADON	29.90943502	114.504874	44.087009		Farmer owned field
180362	2018	Hubei	Rice	*F. asiaticum*	NIV	29.90943502	114.504874	44.087009		Farmer owned field
180361	2018	Hubei	Rice	*F. asiaticum*	NIV	29.90943502	114.504874	44.087009		Farmer owned field
180360	2018	Hubei	Rice	*F. asiaticum*	NIV	29.90943502	114.504874	44.087009		Farmer owned field
180359	2018	Hubei	Rice	*F. asiaticum*	NIV	29.90943502	114.504874	44.087009		Farmer owned field
180358	2018	Hubei	Rice	*F. asiaticum*	NIV	29.90943502	114.504874	44.087009		Farmer owned field
180357	2018	Hubei	Rice	*F. asiaticum*	NIV	29.90943502	114.504874	44.087009		Farmer owned field
180356	2018	Hubei	Rice	*F. asiaticum*	NIV	29.90943502	114.504874	44.087009		Farmer owned field
180355	2018	Hubei	Rice	*F. asiaticum*	NIV	29.90943502	114.504874	44.087009		Farmer owned field
180354	2018	Hubei	Rice	*F. asiaticum*	NIV	29.90943502	114.504874	44.087009		Farmer owned field
180353	2018	Hubei	Rice	*F. asiaticum*	NIV	29.90943502	114.504874	44.087009		Farmer owned field
180352	2018	Hubei	Rice	*F. asiaticum*	NIV	29.90943502	114.504874	44.087009		Farmer owned field
180351	2018	Hubei	Rice	*F. asiaticum*	15ADON	29.989779	114.76211	72.181686		Farmer owned field
180350	2018	Hubei	Rice	*F. asiaticum*	NIV	29.989779	114.76211	72.181686		Farmer owned field
180349	2018	Hubei	Rice	*F. asiaticum*	NIV	29.989779	114.76211	72.181686		Farmer owned field
JSYZ092	2014	Jiangsu	Rice	*F. asiaticum*	NIV	32.270763	119.254133	4		Commercial farm
JSYZ091	2014	Jiangsu	Rice	*F. asiaticum*	3ADON	32.270763	119.254133	4		Commercial farm
JSYZ090	2014	Jiangsu	Rice	*F. asiaticum*	3ADON	32.270763	119.254133	4		Commercial farm
JSYZ089	2014	Jiangsu	Rice	*F. asiaticum*	3ADON	32.270763	119.254133	4		Commercial farm
JSYZ087	2014	Jiangsu	Rice	*F. asiaticum*	3ADON	32.270763	119.254133	4		Commercial farm

JSYZ082	2014	Jiangsu	Rice	*F. asiaticum*	15ADON	32.270763	119.254133	4		Commercial farm
JSYZ081	2014	Jiangsu	Rice	*F. asiaticum*	3ADON	32.270763	119.254133	4		Commercial farm
JSYZ079	2014	Jiangsu	Rice	*F. asiaticum*	3ADON	32.270763	119.254133	4		Commercial farm
JSYZ078	2014	Jiangsu	Rice	*F. asiaticum*	3ADON	32.270763	119.254133	4		Commercial farm
JSYZ076	2014	Jiangsu	Rice	*F. asiaticum*	15ADON	32.270763	119.254133	4		Commercial farm
192061	2019	Anhui	Wheat	*F. asiaticum*	3ADON	30.67082598	117.365424	37.724396	Commercialized variety	Farmer owned field
190075	2019	Anhui	Wheat	*F. asiaticum*	NIV	30.88018798	118.91113	NA	Commercialized variety	Commercial farm
190073	2019	Anhui	Wheat	*F. asiaticum*	NIV	30.88018798	118.91113	NA	Commercialized variety	Commercial farm
190071	2019	Anhui	Wheat	*F. asiaticum*	NIV	30.88018798	118.91113	NA	Commercialized variety	Commercial farm
190068	2019	Anhui	Wheat	*F. asiaticum*	3ADON	30.88018798	118.91113	NA	Commercialized variety	Commercial farm
190067	2019	Anhui	Wheat	*F. asiaticum*	NIV	30.88018798	118.91113	NA	Commercialized variety	Commercial farm
190066	2019	Anhui	Wheat	*F. asiaticum*	3ADON	30.88018798	118.91113	NA	Commercialized variety	Commercial farm
190065	2019	Anhui	Wheat	*F. asiaticum*	3ADON	30.88018798	118.91113	NA	Commercialized variety	Commercial farm
190064	2019	Anhui	Wheat	*F. asiaticum*	3ADON	30.88018798	118.91113	NA	Commercialized variety	Commercial farm
190063	2019	Anhui	Wheat	*F. asiaticum*	NIV	30.88018798	118.91113	NA	Commercialized variety	Commercial farm
190062	2019	Anhui	Wheat	*F. asiaticum*	3ADON	30.88018798	118.91113	NA	Commercialized variety	Commercial farm
190061	2019	Anhui	Wheat	*F. asiaticum*	3ADON	30.88018798	118.91113	NA	Commercialized variety	Commercial farm
190060	2019	Anhui	Wheat	*F. asiaticum*	3ADON	30.88018798	118.91113	NA	Commercialized variety	Commercial farm
190059	2019	Anhui	Wheat	*F. asiaticum*	NIV	30.88018798	118.91113	NA	Commercialized variety	Commercial farm
190058	2019	Anhui	Wheat	*F. asiaticum*	3ADON	30.88018798	118.91113	NA	Commercialized variety	Commercial farm
190057	2019	Anhui	Wheat	*F. asiaticum*	3ADON	30.88018798	118.91113	NA	Commercialized variety	Commercial farm
190056	2019	Anhui	Wheat	*F. asiaticum*	3ADON	30.88018798	118.91113	NA	Commercialized variety	Commercial farm
190054	2019	Anhui	Wheat	*F. asiaticum*	NIV	30.88018798	118.91113	NA	Commercialized variety	Commercial farm
190053	2019	Anhui	Wheat	*F. asiaticum*	NIV	30.88018798	118.91113	NA	Commercialized variety	Commercial farm
190052	2019	Anhui	Wheat	*F. asiaticum*	3ADON	30.88018798	118.91113	NA	Commercialized variety	Commercial farm
190051	2019	Anhui	Wheat	*F. asiaticum*	3ADON	30.88018798	118.91113	NA	Commercialized variety	Commercial farm
190050	2019	Anhui	Wheat	*F. asiaticum*	NIV	30.88018798	118.91113	NA	Commercialized variety	Commercial farm
190049	2019	Anhui	Wheat	*F. asiaticum*	3ADON	30.88018798	118.91113	NA	Commercialized variety	Commercial farm
FT048	2014	Anhui	Rice	*F. asiaticum*	3ADON	30.94522	118.828377	15		Commercial farm
FT045	2014	Anhui	Rice	*F. asiaticum*	3ADON	30.94522	118.828377	15		Commercial farm
FT040	2014	Anhui	Rice	*F. asiaticum*	NIV	30.94522	118.828377	15		Commercial farm
FT039	2014	Anhui	Rice	*F. asiaticum*	NIV	30.94522	118.828377	15		Commercial farm
FT037	2014	Anhui	Rice	*F. asiaticum*	3ADON	30.94522	118.828377	15		Commercial farm
FT027	2014	Anhui	Rice	*F. asiaticum*	NIV	30.94522	118.828377	15		Commercial farm
FT025-2	2014	Anhui	Rice	*F. asiaticum*	3ADON	30.94522	118.828377	15		Commercial farm

FT025	2014	Anhui	Rice	*F. asiaticum*	NIV	30.94522	118.828377	15		Commercial farm
FT024	2014	Anhui	Rice	*F. asiaticum*	3ADON	30.94522	118.828377	15		Commercial farm
FT022	2014	Anhui	Rice	*F. asiaticum*	3ADON	30.94522	118.828377	15		Commercial farm
FT020	2014	Anhui	Rice	*F. asiaticum*	3ADON	30.94522	118.828377	15		Commercial farm
FT019	2014	Anhui	Rice	*F. asiaticum*	3ADON	30.94522	118.828377	15		Commercial farm
180348	2018	Hubei	Rice	*F. asiaticum*	NIV	29.989779	114.76211	72.181686		Farmer owned field
180347	2018	Hubei	Rice	*F. asiaticum*	NIV	29.989779	114.76211	72.181686		Farmer owned field
180346	2018	Hubei	Rice	*F. asiaticum*	NIV	29.989779	114.76211	72.181686		Farmer owned field
180345	2018	Hubei	Rice	*F. asiaticum*	15ADON	29.989779	114.76211	72.181686		Farmer owned field
180344	2018	Hubei	Rice	*F. asiaticum*	15ADON	29.989779	114.76211	72.181686		Farmer owned field
180343	2018	Hubei	Rice	*F. asiaticum*	NIV	29.989779	114.76211	72.181686		Farmer owned field
180342	2018	Hubei	Rice	*F. asiaticum*	NIV	29.989779	114.76211	72.181686		Farmer owned field
180341	2018	Hubei	Rice	*F. asiaticum*	3ADON	29.989779	114.76211	72.181686		Farmer owned field
180340	2018	Hubei	Rice	*F. asiaticum*	NIV	29.989779	114.76211	72.181686		Farmer owned field
180338	2018	Hubei	Rice	*F. asiaticum*	NIV	29.989779	114.76211	72.181686		Farmer owned field
180336	2018	Hubei	Rice	*F. asiaticum*	NIV	29.989779	114.76211	72.181686		Farmer owned field
180334	2018	Hubei	Rice	*F. asiaticum*	NIV	29.989779	114.76211	72.181686		Farmer owned field
180333	2018	Hubei	Rice	*F. asiaticum*	NIV	29.989779	114.76211	72.181686		Farmer owned field
180332	2018	Hubei	Rice	*F. asiaticum*	NIV	29.989779	114.76211	72.181686		Farmer owned field
180331	2018	Hubei	Rice	*F. asiaticum*	NIV	29.989779	114.76211	72.181686		Farmer owned field
180330	2018	Hubei	Rice	*F. asiaticum*	NIV	29.989779	114.76211	72.181686		Farmer owned field
180329	2018	Hubei	Rice	*F. asiaticum*	NIV	29.989779	114.76211	72.181686		Farmer owned field
180328	2018	Hubei	Rice	*F. asiaticum*	NIV	29.989779	114.76211	72.181686		Farmer owned field
180326	2018	Hubei	Rice	*F. asiaticum*	NIV	29.989779	114.76211	72.181686		Farmer owned field
180325	2018	Hubei	Rice	*F. asiaticum*	NIV	29.989779	114.76211	72.181686		Farmer owned field
180324	2018	Hubei	Rice	*F. asiaticum*	NIV	29.989779	114.76211	72.181686		Farmer owned field
180323	2018	Hubei	Rice	*F. asiaticum*	NIV	29.989779	114.76211	72.181686		Farmer owned field
180322	2018	Hubei	Rice	*F. asiaticum*	NIV	29.989779	114.76211	72.181686		Farmer owned field
180321	2018	Hubei	Rice	*F. asiaticum*	NIV	29.989779	114.76211	72.181686		Farmer owned field
180320	2018	Hubei	Rice	*F. asiaticum*	NIV	29.989779	114.76211	72.181686		Farmer owned field
180319	2018	Hubei	Rice	*F. asiaticum*	NIV	29.989779	114.76211	72.181686		Farmer owned field
180318	2018	Hubei	Rice	*F. asiaticum*	NIV	29.989779	114.76211	72.181686		Farmer owned field
180317	2018	Hubei	Rice	*F. asiaticum*	3ADON	29.989779	114.76211	72.181686		Farmer owned field
180316	2018	Hubei	Rice	*F. asiaticum*	NIV	29.989779	114.76211	72.181686		Farmer owned field
180315	2018	Hubei	Rice	*F. asiaticum*	NIV	29.989779	114.76211	72.181686		Farmer owned field

180314	2018	Hubei	Rice	*F. asiaticum*	15ADON	29.989779	114.76211	72.181686		Farmer owned field
180313	2018	Hubei	Rice	*F. asiaticum*	NIV	29.989779	114.76211	72.181686		Farmer owned field
180312	2018	Hubei	Rice	*F. asiaticum*	NIV	29.989779	114.76211	72.181686		Farmer owned field
180311	2018	Hubei	Rice	*F. asiaticum*	NIV	29.989779	114.76211	72.181686		Farmer owned field
180310	2018	Hubei	Rice	*F. asiaticum*	NIV	29.989779	114.76211	72.181686		Farmer owned field
180309	2018	Hubei	Rice	*F. asiaticum*	NIV	29.989779	114.76211	72.181686		Farmer owned field
181447	2018	Hubei	Rice	*F. asiaticum*	3ADON	29.99109898	112.53872	31.112852		Farmer owned field
181446	2018	Hubei	Rice	*F. asiaticum*	3ADON	29.99109898	112.53872	31.112852		Farmer owned field
181445	2018	Hubei	Rice	*F. asiaticum*	NIV	29.99109898	112.53872	31.112852		Farmer owned field
181444	2018	Hubei	Rice	*F. asiaticum*	3ADON	29.99109898	112.53872	31.112852		Farmer owned field
181443	2018	Hubei	Rice	*F. asiaticum*	3ADON	29.99109898	112.53872	31.112852		Farmer owned field
181442	2018	Hubei	Rice	*F. asiaticum*	NIV	29.99109898	112.53872	31.112852		Farmer owned field
181441	2018	Hubei	Rice	*F. asiaticum*	3ADON	29.99109898	112.53872	31.112852		Farmer owned field
181440	2018	Hubei	Rice	*F. asiaticum*	3ADON	29.99109898	112.53872	31.112852		Farmer owned field
181438	2018	Hubei	Rice	*F. asiaticum*	3ADON	29.99109898	112.53872	31.112852		Farmer owned field
181437	2018	Hubei	Rice	*F. asiaticum*	3ADON	29.99109898	112.53872	31.112852		Farmer owned field
181436	2018	Hubei	Rice	*F. asiaticum*	3ADON	29.99109898	112.53872	31.112852		Farmer owned field
181434	2018	Hubei	Rice	*F. asiaticum*	3ADON	29.99109898	112.53872	31.112852		Farmer owned field
191990	2019	Hubei	Wheat	*F. asiaticum*	3ADON	30.04730299	112.153793	35.333096	Commercialized variety	Farmer owned field
191989	2019	Hubei	Wheat	*F. asiaticum*	3ADON	30.04730299	112.153793	35.333096	Commercialized variety	Farmer owned field
191988	2019	Hubei	Wheat	*F. asiaticum*	15ADON	30.04730299	112.153793	35.333096	Commercialized variety	Farmer owned field
191987	2019	Hubei	Wheat	*F. asiaticum*	3ADON	30.04730299	112.153793	35.333096	Commercialized variety	Farmer owned field
191986	2019	Hubei	Wheat	*F. asiaticum*	3ADON	30.04730299	112.153793	35.333096	Commercialized variety	Farmer owned field
191985	2019	Hubei	Wheat	*F. asiaticum*	NIV	30.04730299	112.153793	35.333096	Commercialized variety	Farmer owned field
191984	2019	Hubei	Wheat	*F. asiaticum*	3ADON	30.04730299	112.153793	35.333096	Commercialized variety	Farmer owned field
191983	2019	Hubei	Wheat	*F. asiaticum*	3ADON	30.04730299	112.153793	35.333096	Commercialized variety	Farmer owned field
191982	2019	Hubei	Wheat	*F. asiaticum*	15ADON	30.04730299	112.153793	35.333096	Commercialized variety	Farmer owned field
191981	2019	Hubei	Wheat	*F. asiaticum*	15ADON	30.04730299	112.153793	35.333096	Commercialized variety	Farmer owned field
191980	2019	Hubei	Wheat	*F. asiaticum*	3ADON	30.04730299	112.153793	35.333096	Commercialized variety	Farmer owned field
191979	2019	Hubei	Wheat	*F. asiaticum*	3ADON	30.04730299	112.153793	35.333096	Commercialized variety	Farmer owned field
191978	2019	Hubei	Wheat	*F. asiaticum*	3ADON	30.04730299	112.153793	35.333096	Commercialized variety	Farmer owned field
191977	2019	Hubei	Wheat	*F. asiaticum*	15ADON	30.04730299	112.153793	35.333096	Commercialized variety	Farmer owned field
191976	2019	Hubei	Wheat	*F. asiaticum*	3ADON	30.04730299	112.153793	35.333096	Commercialized variety	Farmer owned field
191975	2019	Hubei	Wheat	*F. asiaticum*	3ADON	30.04730299	112.153793	35.333096	Commercialized variety	Farmer owned field
191974	2019	Hubei	Wheat	*F. asiaticum*	15ADON	30.04730299	112.153793	35.333096	Commercialized variety	Farmer owned field

191973	2019	Hubei	Wheat	*F. asiaticum*	NIV	30.04730299	112.153793	35.333096	Commercialized variety	Farmer owned field
191972	2019	Hubei	Wheat	*F. asiaticum*	NIV	30.04730299	112.153793	35.333096	Commercialized variety	Farmer owned field
191971	2019	Hubei	Wheat	*F. asiaticum*	3ADON	30.04730299	112.153793	35.333096	Commercialized variety	Farmer owned field
191970	2019	Hubei	Wheat	*F. asiaticum*	15ADON	30.04730299	112.153793	35.333096	Commercialized variety	Farmer owned field
180657	2018	Hubei	Wheat	*F. asiaticum*	3ADON	30.04730299	112.153793	35.333096	Commercialized variety	Farmer owned field
180656	2018	Hubei	Wheat	*F. asiaticum*	3ADON	30.04730299	112.153793	35.333096	Commercialized variety	Farmer owned field
180655	2018	Hubei	Wheat	*F. asiaticum*	15ADON	30.04730299	112.153793	35.333096	Commercialized variety	Farmer owned field
180654	2018	Hubei	Wheat	*F. asiaticum*	3ADON	30.04730299	112.153793	35.333096	Commercialized variety	Farmer owned field
180653	2018	Hubei	Wheat	*F. asiaticum*	3ADON	30.04730299	112.153793	35.333096	Commercialized variety	Farmer owned field
180652	2018	Hubei	Wheat	*F. asiaticum*	3ADON	30.04730299	112.153793	35.333096	Commercialized variety	Farmer owned field
180651	2018	Hubei	Wheat	*F. asiaticum*	3ADON	30.04730299	112.153793	35.333096	Commercialized variety	Farmer owned field
180650	2018	Hubei	Wheat	*F. asiaticum*	NIV	30.04730299	112.153793	35.333096	Commercialized variety	Farmer owned field
180649	2018	Hubei	Wheat	*F. asiaticum*	NIV	30.04730299	112.153793	35.333096	Commercialized variety	Farmer owned field
180648	2018	Hubei	Wheat	*F. asiaticum*	NIV	30.04730299	112.153793	35.333096	Commercialized variety	Farmer owned field
180647	2018	Hubei	Wheat	*F. asiaticum*	NIV	30.04730299	112.153793	35.333096	Commercialized variety	Farmer owned field
180646	2018	Hubei	Wheat	*F. asiaticum*	3ADON	30.04730299	112.153793	35.333096	Commercialized variety	Farmer owned field
180645	2018	Hubei	Wheat	*F. asiaticum*	3ADON	30.04730299	112.153793	35.333096	Commercialized variety	Farmer owned field
180644	2018	Hubei	Wheat	*F. asiaticum*	3ADON	30.04730299	112.153793	35.333096	Commercialized variety	Farmer owned field
180643	2018	Hubei	Wheat	*F. asiaticum*	3ADON	30.04730299	112.153793	35.333096	Commercialized variety	Farmer owned field
180642	2018	Hubei	Wheat	*F. asiaticum*	3ADON	30.04730299	112.153793	35.333096	Commercialized variety	Farmer owned field
180641	2018	Hubei	Wheat	*F. asiaticum*	3ADON	30.04730299	112.153793	35.333096	Commercialized variety	Farmer owned field
180640	2018	Hubei	Wheat	*F. asiaticum*	3ADON	30.04730299	112.153793	35.333096	Commercialized variety	Farmer owned field
180639	2018	Hubei	Wheat	*F. asiaticum*	3ADON	30.04730299	112.153793	35.333096	Commercialized variety	Farmer owned field
180638	2018	Hubei	Wheat	*F. asiaticum*	15ADON	30.04730299	112.153793	35.333096	Commercialized variety	Farmer owned field
192172	2019	Hubei	Wheat	*F. asiaticum*	3ADON	30.05112296	112.430032	37.095524	Commercialized variety	Farmer owned field
192171	2019	Hubei	Wheat	*F. asiaticum*	3ADON	30.05112296	112.430032	37.095524	Commercialized variety	Farmer owned field
192170	2019	Hubei	Wheat	*F. asiaticum*	3ADON	30.05112296	112.430032	37.095524	Commercialized variety	Farmer owned field
192169	2019	Hubei	Wheat	*F. asiaticum*	3ADON	30.05112296	112.430032	37.095524	Commercialized variety	Farmer owned field
192168	2019	Hubei	Wheat	*F. asiaticum*	3ADON	30.05112296	112.430032	37.095524	Commercialized variety	Farmer owned field
192167	2019	Hubei	Wheat	*F. asiaticum*	3ADON	30.05112296	112.430032	37.095524	Commercialized variety	Farmer owned field
192166	2019	Hubei	Wheat	*F. asiaticum*	NIV	30.05112296	112.430032	37.095524	Commercialized variety	Farmer owned field
192165	2019	Hubei	Wheat	*F. asiaticum*	15ADON	30.05112296	112.430032	37.095524	Commercialized variety	Farmer owned field
192164	2019	Hubei	Wheat	*F. asiaticum*	NIV	30.05112296	112.430032	37.095524	Commercialized variety	Farmer owned field
192163	2019	Hubei	Wheat	*F. asiaticum*	3ADON	30.05112296	112.430032	37.095524	Commercialized variety	Farmer owned field
192162	2019	Hubei	Wheat	*F. asiaticum*	3ADON	30.05112296	112.430032	37.095524	Commercialized variety	Farmer owned field

192161	2019	Hubei	Wheat	*F. asiaticum*	3ADON	30.05112296	112.430032	37.095524	Commercialized variety	Farmer owned field
192160	2019	Hubei	Wheat	*F. asiaticum*	3ADON	30.05112296	112.430032	37.095524	Commercialized variety	Farmer owned field
192159	2019	Hubei	Wheat	*F. asiaticum*	3ADON	30.05112296	112.430032	37.095524	Commercialized variety	Farmer owned field
192158	2019	Hubei	Wheat	*F. asiaticum*	3ADON	30.05112296	112.430032	37.095524	Commercialized variety	Farmer owned field
192157	2019	Hubei	Wheat	*F. asiaticum*	3ADON	30.05112296	112.430032	37.095524	Commercialized variety	Farmer owned field
192155	2019	Hubei	Wheat	*F. asiaticum*	3ADON	30.05112296	112.430032	37.095524	Commercialized variety	Farmer owned field
191887	2019	Hubei	Wheat	*F. asiaticum*	NIV	30.06588198	114.882799	26.362423	Commercialized variety	Farmer owned field
191886	2019	Hubei	Wheat	*F. asiaticum*	3ADON	30.06588198	114.882799	26.362423	Commercialized variety	Farmer owned field
191885	2019	Hubei	Wheat	*F. asiaticum*	NIV	30.06588198	114.882799	26.362423	Commercialized variety	Farmer owned field
191884	2019	Hubei	Wheat	*F. asiaticum*	NIV	30.06588198	114.882799	26.362423	Commercialized variety	Farmer owned field
191883	2019	Hubei	Wheat	*F. asiaticum*	NIV	30.06588198	114.882799	26.362423	Commercialized variety	Farmer owned field
191882	2019	Hubei	Wheat	*F. asiaticum*	15ADON	30.06588198	114.882799	26.362423	Commercialized variety	Farmer owned field
191881	2019	Hubei	Wheat	*F. asiaticum*	3ADON	30.06588198	114.882799	26.362423	Commercialized variety	Farmer owned field
191880	2019	Hubei	Wheat	*F. asiaticum*	NIV	30.06588198	114.882799	26.362423	Commercialized variety	Farmer owned field
191879	2019	Hubei	Wheat	*F. asiaticum*	3ADON	30.06588198	114.882799	26.362423	Commercialized variety	Farmer owned field
191878	2019	Hubei	Wheat	*F. asiaticum*	NIV	30.06588198	114.882799	26.362423	Commercialized variety	Farmer owned field
191877	2019	Hubei	Wheat	*F. asiaticum*	3ADON	30.06588198	114.882799	26.362423	Commercialized variety	Farmer owned field
191876	2019	Hubei	Wheat	*F. asiaticum*	NIV	30.06588198	114.882799	26.362423	Commercialized variety	Farmer owned field
191875	2019	Hubei	Wheat	*F. asiaticum*	3ADON	30.06588198	114.882799	26.362423	Commercialized variety	Farmer owned field
191874	2019	Hubei	Wheat	*F. asiaticum*	3ADON	30.06588198	114.882799	26.362423	Commercialized variety	Farmer owned field
191873	2019	Hubei	Wheat	*F. asiaticum*	NIV	30.06588198	114.882799	26.362423	Commercialized variety	Farmer owned field
191872	2019	Hubei	Wheat	*F. asiaticum*	3ADON	30.06588198	114.882799	26.362423	Commercialized variety	Farmer owned field
191871	2019	Hubei	Wheat	*F. asiaticum*	NIV	30.06588198	114.882799	26.362423	Commercialized variety	Farmer owned field
191870	2019	Hubei	Wheat	*F. asiaticum*	3ADON	30.06588198	114.882799	26.362423	Commercialized variety	Farmer owned field
191869	2019	Hubei	Wheat	*F. asiaticum*	3ADON	30.06588198	114.882799	26.362423	Commercialized variety	Farmer owned field
191868	2019	Hubei	Wheat	*F. asiaticum*	15ADON	30.06588198	114.882799	26.362423	Commercialized variety	Farmer owned field
191867	2019	Hubei	Wheat	*F. asiaticum*	3ADON	30.06588198	114.882799	26.362423	Commercialized variety	Farmer owned field
191866	2019	Hubei	Wheat	*F. asiaticum*	3ADON	30.06588198	114.882799	26.362423	Commercialized variety	Farmer owned field
191865	2019	Hubei	Wheat	*F. asiaticum*	15ADON	30.06588198	114.882799	26.362423	Commercialized variety	Farmer owned field
191864	2019	Hubei	Wheat	*F. asiaticum*	15ADON	30.06588198	114.882799	26.362423	Commercialized variety	Farmer owned field
191863	2019	Hubei	Wheat	*F. asiaticum*	15ADON	30.06588198	114.882799	26.362423	Commercialized variety	Farmer owned field
191968	2019	Hubei	Wheat	*F. asiaticum*	3ADON	30.10676103	113.016938	37.91993	Commercialized variety	Farmer owned field
191967	2019	Hubei	Wheat	*F. asiaticum*	3ADON	30.10676103	113.016938	37.91993	Commercialized variety	Farmer owned field
191966	2019	Hubei	Wheat	*F. asiaticum*	3ADON	30.10676103	113.016938	37.91993	Commercialized variety	Farmer owned field
191965	2019	Hubei	Wheat	*F. asiaticum*	3ADON	30.10676103	113.016938	37.91993	Commercialized variety	Farmer owned field

191964	2019	Hubei	Wheat	*F. asiaticum*	3ADON	30.10676103	113.016938	37.91993	Commercialized variety	Farmer owned field
191963	2019	Hubei	Wheat	*F. asiaticum*	15ADON	30.10676103	113.016938	37.91993	Commercialized variety	Farmer owned field
191962	2019	Hubei	Wheat	*F. asiaticum*	3ADON	30.10676103	113.016938	37.91993	Commercialized variety	Farmer owned field
191961	2019	Hubei	Wheat	*F. asiaticum*	3ADON	30.10676103	113.016938	37.91993	Commercialized variety	Farmer owned field
191960	2019	Hubei	Wheat	*F. asiaticum*	3ADON	30.10676103	113.016938	37.91993	Commercialized variety	Farmer owned field
191959	2019	Hubei	Wheat	*F. asiaticum*	3ADON	30.10676103	113.016938	37.91993	Commercialized variety	Farmer owned field
191847	2019	Hubei	Wheat	*F. asiaticum*	3ADON	30.10676103	113.016938	37.91993	Commercialized variety	Farmer owned field
191846	2019	Hubei	Wheat	*F. asiaticum*	3ADON	30.10676103	113.016938	37.91993	Commercialized variety	Farmer owned field
191845	2019	Hubei	Wheat	*F. asiaticum*	3ADON	30.10676103	113.016938	37.91993	Commercialized variety	Farmer owned field
191844	2019	Hubei	Wheat	*F. asiaticum*	3ADON	30.10676103	113.016938	37.91993	Commercialized variety	Farmer owned field
191843	2019	Hubei	Wheat	*F. asiaticum*	15ADON	30.10676103	113.016938	37.91993	Commercialized variety	Farmer owned field
191841	2019	Hubei	Wheat	*F. asiaticum*	3ADON	30.10676103	113.016938	37.91993	Commercialized variety	Farmer owned field
191902	2019	Hubei	Wheat	*F. asiaticum*	3ADON	30.17940204	111.834731	67.529762	Commercialized variety	Farmer owned field
191901	2019	Hubei	Wheat	*F. asiaticum*	3ADON	30.17940204	111.834731	67.529762	Commercialized variety	Farmer owned field
191900	2019	Hubei	Wheat	*F. asiaticum*	3ADON	30.17940204	111.834731	67.529762	Commercialized variety	Farmer owned field
191898	2019	Hubei	Wheat	*F. asiaticum*	3ADON	30.17940204	111.834731	67.529762	Commercialized variety	Farmer owned field
191897	2019	Hubei	Wheat	*F. asiaticum*	3ADON	30.17940204	111.834731	67.529762	Commercialized variety	Farmer owned field
191895	2019	Hubei	Wheat	*F. asiaticum*	3ADON	30.17940204	111.834731	67.529762	Commercialized variety	Farmer owned field
191894	2019	Hubei	Wheat	*F. asiaticum*	3ADON	30.17940204	111.834731	67.529762	Commercialized variety	Farmer owned field
191893	2019	Hubei	Wheat	*F. asiaticum*	15ADON	30.17940204	111.834731	67.529762	Commercialized variety	Farmer owned field
191890	2019	Hubei	Wheat	*F. asiaticum*	15ADON	30.17940204	111.834731	67.529762	Commercialized variety	Farmer owned field
191888	2019	Hubei	Wheat	*F. asiaticum*	15ADON	30.17940204	111.834731	67.529762	Commercialized variety	Farmer owned field
191785	2019	Hubei	Wheat	*F. asiaticum*	3ADON	30.23301403	112.352811	28.518013	Commercialized variety	Commercial farm
191784	2019	Hubei	Wheat	*F. asiaticum*	3ADON	30.23301403	112.352811	28.518013	Commercialized variety	Commercial farm
191783	2019	Hubei	Wheat	*F. asiaticum*	3ADON	30.23301403	112.352811	28.518013	Commercialized variety	Commercial farm
191782	2019	Hubei	Wheat	*F. asiaticum*	3ADON	30.23301403	112.352811	28.518013	Commercialized variety	Commercial farm
191781	2019	Hubei	Wheat	*F. asiaticum*	3ADON	30.23301403	112.352811	28.518013	Commercialized variety	Commercial farm
191780	2019	Hubei	Wheat	*F. asiaticum*	NIV	30.23301403	112.352811	28.518013	Commercialized variety	Commercial farm
191779	2019	Hubei	Wheat	*F. asiaticum*	3ADON	30.23301403	112.352811	28.518013	Commercialized variety	Commercial farm
191778	2019	Hubei	Wheat	*F. asiaticum*	3ADON	30.23301403	112.352811	28.518013	Commercialized variety	Commercial farm
191777	2019	Hubei	Wheat	*F. asiaticum*	3ADON	30.23301403	112.352811	28.518013	Commercialized variety	Commercial farm
191776	2019	Hubei	Wheat	*F. asiaticum*	3ADON	30.23301403	112.352811	28.518013	Commercialized variety	Commercial farm
191767	2019	Hubei	Wheat	*F. asiaticum*	3ADON	30.23301403	112.352811	28.518013	Commercialized variety	Commercial farm
191766	2019	Hubei	Wheat	*F. asiaticum*	3ADON	30.23301403	112.352811	28.518013	Commercialized variety	Commercial farm
191765	2019	Hubei	Wheat	*F. asiaticum*	3ADON	30.23301403	112.352811	28.518013	Commercialized variety	Commercial farm

191764	2019	Hubei	Wheat	*F. asiaticum*	3ADON	30.23301403	112.352811	28.518013	Commercialized variety	Commercial farm
191763	2019	Hubei	Wheat	*F. asiaticum*	3ADON	30.23301403	112.352811	28.518013	Commercialized variety	Commercial farm
191761	2019	Hubei	Wheat	*F. asiaticum*	NIV	30.23301403	112.352811	28.518013	Commercialized variety	Commercial farm
191760	2019	Hubei	Wheat	*F. asiaticum*	3ADON	30.23301403	112.352811	28.518013	Commercialized variety	Commercial farm
191759	2019	Hubei	Wheat	*F. asiaticum*	3ADON	30.23301403	112.352811	28.518013	Commercialized variety	Commercial farm
191758	2019	Hubei	Wheat	*F. asiaticum*	3ADON	30.23301403	112.352811	28.518013	Commercialized variety	Commercial farm
191757	2019	Hubei	Wheat	*F. asiaticum*	3ADON	30.23301403	112.352811	28.518013	Commercialized variety	Commercial farm
191756	2019	Hubei	Wheat	*F. asiaticum*	3ADON	30.23301403	112.352811	28.518013	Commercialized variety	Commercial farm
191755	2019	Hubei	Wheat	*F. asiaticum*	15ADON	30.23301403	112.352811	28.518013	Commercialized variety	Commercial farm
191754	2019	Hubei	Wheat	*F. asiaticum*	3ADON	30.23301403	112.352811	28.518013	Commercialized variety	Commercial farm
191752	2019	Hubei	Wheat	*F. asiaticum*	3ADON	30.23301403	112.352811	28.518013	Commercialized variety	Commercial farm
181075	2018	Hubei	Wheat	*F. asiaticum*	3ADON	30.23301403	112.352811	28.518013	Commercialized variety	Commercial farm
JSYZ076	2014	Jiangsu	Rice	*F. asiaticum*	3ADON	32.270763	119.254133	4		Commercial farm
JSYZ072	2014	Jiangsu	Rice	*F. asiaticum*	NIV	32.270763	119.254133	4		Commercial farm
JSYZ071	2014	Jiangsu	Rice	*F. asiaticum*	3ADON	32.270763	119.254133	4		Commercial farm
JSYZ070	2014	Jiangsu	Rice	*F. asiaticum*	3ADON	32.270763	119.254133	4		Commercial farm
JSYZ069	2014	Jiangsu	Rice	*F. asiaticum*	3ADON	32.270763	119.254133	4		Commercial farm
JSYZ068	2014	Jiangsu	Rice	*F. asiaticum*	3ADON	32.270763	119.254133	4		Commercial farm
JSYZ067	2014	Jiangsu	Rice	*F. asiaticum*	3ADON	32.270763	119.254133	4		Commercial farm
181074	2018	Hubei	Wheat	*F. asiaticum*	3ADON	30.23301403	112.352811	28.518013	Commercialized variety	Commercial farm
181073	2018	Hubei	Wheat	*F. asiaticum*	3ADON	30.23301403	112.352811	28.518013	Commercialized variety	Commercial farm
181072	2018	Hubei	Wheat	*F. asiaticum*	3ADON	30.23301403	112.352811	28.518013	Commercialized variety	Commercial farm
181071	2018	Hubei	Wheat	*F. asiaticum*	3ADON	30.23301403	112.352811	28.518013	Commercialized variety	Commercial farm
181070	2018	Hubei	Wheat	*F. asiaticum*	3ADON	30.23301403	112.352811	28.518013	Commercialized variety	Commercial farm
181069	2018	Hubei	Wheat	*F. asiaticum*	15ADON	30.23301403	112.352811	28.518013	Commercialized variety	Commercial farm
181067	2018	Hubei	Wheat	*F. asiaticum*	3ADON	30.23301403	112.352811	28.518013	Commercialized variety	Commercial farm
181066	2018	Hubei	Wheat	*F. asiaticum*	3ADON	30.23301403	112.352811	28.518013	Commercialized variety	Commercial farm
181065	2018	Hubei	Wheat	*F. asiaticum*	3ADON	30.23301403	112.352811	28.518013	Commercialized variety	Commercial farm
181064	2018	Hubei	Wheat	*F. asiaticum*	3ADON	30.23301403	112.352811	28.518013	Commercialized variety	Commercial farm
181063	2018	Hubei	Wheat	*F. asiaticum*	3ADON	30.23301403	112.352811	28.518013	Commercialized variety	Commercial farm
180536	2018	Hubei	Rice	*F. asiaticum*	3ADON	30.23301403	112.352811	28.518013		Commercial farm
180535	2018	Hubei	Rice	*F. asiaticum*	3ADON	30.23301403	112.352811	28.518013		Commercial farm
180534	2018	Hubei	Rice	*F. asiaticum*	3ADON	30.23301403	112.352811	28.518013		Commercial farm
180533	2018	Hubei	Rice	*F. asiaticum*	3ADON	30.23301403	112.352811	28.518013		Commercial farm
180532	2018	Hubei	Rice	*F. asiaticum*	3ADON	30.23301403	112.352811	28.518013		Commercial farm

180531	2018	Hubei	Rice	*F. asiaticum*	3ADON	30.23301403	112.352811	28.518013	Commercial farm
180529	2018	Hubei	Rice	*F. asiaticum*	3ADON	30.23301403	112.352811	28.518013	Commercial farm
180528	2018	Hubei	Rice	*F. asiaticum*	NIV	30.23301403	112.352811	28.518013	Commercial farm
180526	2018	Hubei	Rice	*F. asiaticum*	3ADON	30.23301403	112.352811	28.518013	Commercial farm
180525	2018	Hubei	Rice	*F. asiaticum*	3ADON	30.23301403	112.352811	28.518013	Commercial farm
180524	2018	Hubei	Rice	*F. asiaticum*	3ADON	30.23301403	112.352811	28.518013	Commercial farm
180523	2018	Hubei	Rice	*F. asiaticum*	3ADON	30.23301403	112.352811	28.518013	Commercial farm
180522	2018	Hubei	Rice	*F. asiaticum*	3ADON	30.23301403	112.352811	28.518013	Commercial farm
180521	2018	Hubei	Rice	*F. asiaticum*	3ADON	30.23301403	112.352811	28.518013	Commercial farm
180519	2018	Hubei	Rice	*F. asiaticum*	3ADON	30.23301403	112.352811	28.518013	Commercial farm
180518	2018	Hubei	Rice	*F. asiaticum*	3ADON	30.23301403	112.352811	28.518013	Commercial farm
180517	2018	Hubei	Rice	*F. asiaticum*	3ADON	30.23301403	112.352811	28.518013	Commercial farm
180515	2018	Hubei	Rice	*F. asiaticum*	3ADON	30.23301403	112.352811	28.518013	Commercial farm
180514	2018	Hubei	Rice	*F. asiaticum*	NIV	30.23301403	112.352811	28.518013	Commercial farm
180513	2018	Hubei	Rice	*F. asiaticum*	3ADON	30.23301403	112.352811	28.518013	Commercial farm
180512	2018	Hubei	Rice	*F. asiaticum*	3ADON	30.23301403	112.352811	28.518013	Commercial farm
180511	2018	Hubei	Rice	*F. asiaticum*	3ADON	30.23301403	112.352811	28.518013	Commercial farm
180510	2018	Hubei	Rice	*F. asiaticum*	3ADON	30.23301403	112.352811	28.518013	Commercial farm
FT017	2014	Anhui	Rice	*F. asiaticum*	3ADON	30.94522	118.828377	15	Commercial farm
FT016	2014	Anhui	Rice	*F. asiaticum*	NIV	30.94522	118.828377	15	Commercial farm
FT014	2014	Anhui	Rice	*F. asiaticum*	3ADON	30.94522	118.828377	15	Commercial farm
AH14098	2014	Anhui	Rice	*F. asiaticum*	3ADON	30.94522	118.828377	15	Commercial farm
14084-2	2014	Anhui	Rice	*F. asiaticum*	3ADON	30.94522	118.828377	15	Commercial farm
14012-2	2014	Anhui	Rice	*F. asiaticum*	3ADON	30.94522	118.828377	15	Commercial farm
14009-2	2014	Anhui	Rice	*F. asiaticum*	3ADON	30.94522	118.828377	15	Commercial farm
14116	2014	Anhui	Rice	*F. asiaticum*	3ADON	30.94522	118.828377	15	Commercial farm
14114	2014	Anhui	Rice	*F. asiaticum*	3ADON	30.94522	118.828377	15	Commercial farm
14112	2014	Anhui	Rice	*F. asiaticum*	3ADON	30.94522	118.828377	15	Commercial farm
14111	2014	Anhui	Rice	*F. asiaticum*	NIV	30.94522	118.828377	15	Commercial farm
14108	2014	Anhui	Rice	*F. asiaticum*	3ADON	30.94522	118.828377	15	Commercial farm
14106	2014	Anhui	Rice	*F. asiaticum*	3ADON	30.94522	118.828377	15	Commercial farm
14104	2014	Anhui	Rice	*F. asiaticum*	NIV	30.94522	118.828377	15	Commercial farm
14103	2014	Anhui	Rice	*F. asiaticum*	NIV	30.94522	118.828377	15	Commercial farm
14102	2014	Anhui	Rice	*F. asiaticum*	3ADON	30.94522	118.828377	15	Commercial farm
14101	2014	Anhui	Rice	*F. asiaticum*	3ADON	30.94522	118.828377	15	Commercial farm

14095	2014	Anhui	Rice	*F. asiaticum*	3ADON	30.94522	118.828377	15		Commercial farm
14094	2014	Anhui	Rice	*F. asiaticum*	3ADON	30.94522	118.828377	15		Commercial farm
14092	2014	Anhui	Rice	*F. asiaticum*	3ADON	30.94522	118.828377	15		Commercial farm
14091	2014	Anhui	Rice	*F. asiaticum*	NIV	30.94522	118.828377	15		Commercial farm
14085	2014	Anhui	Rice	*F. asiaticum*	3ADON	30.94522	118.828377	15		Commercial farm
14084	2014	Anhui	Rice	*F. asiaticum*	3ADON	30.94522	118.828377	15		Commercial farm
14083	2014	Anhui	Rice	*F. asiaticum*	3ADON	30.94522	118.828377	15		Commercial farm
14081	2014	Anhui	Rice	*F. asiaticum*	NIV	30.94522	118.828377	15		Commercial farm
14078	2014	Anhui	Rice	*F. asiaticum*	NIV	30.94522	118.828377	15		Commercial farm
14077	2014	Anhui	Rice	*F. asiaticum*	3ADON	30.94522	118.828377	15		Commercial farm
14075	2014	Anhui	Rice	*F. asiaticum*	3ADON	30.94522	118.828377	15		Commercial farm
14073	2014	Anhui	Rice	*F. asiaticum*	NIV	30.94522	118.828377	15		Commercial farm
14072	2014	Anhui	Rice	*F. asiaticum*	NIV	30.94522	118.828377	15		Commercial farm
14068	2014	Anhui	Rice	*F. asiaticum*	NIV	30.94522	118.828377	15		Commercial farm
14067	2014	Anhui	Rice	*F. asiaticum*	3ADON	30.94522	118.828377	15		Commercial farm
14064	2014	Anhui	Rice	*F. asiaticum*	3ADON	30.94522	118.828377	15		Commercial farm
14063	2014	Anhui	Rice	*F. asiaticum*	3ADON	30.94522	118.828377	15		Commercial farm
14062	2014	Anhui	Rice	*F. asiaticum*	NIV	30.94522	118.828377	15		Commercial farm
14061	2014	Anhui	Rice	*F. asiaticum*	3ADON	30.94522	118.828377	15		Commercial farm
14056	2014	Anhui	Rice	*F. asiaticum*	NIV	30.94522	118.828377	15		Commercial farm
14054	2014	Anhui	Rice	*F. asiaticum*	NIV	30.94522	118.828377	15		Commercial farm
14053	2014	Anhui	Rice	*F. asiaticum*	NIV	30.94522	118.828377	15		Commercial farm
14052	2014	Anhui	Rice	*F. asiaticum*	3ADON	30.94522	118.828377	15		Commercial farm
14051	2014	Anhui	Rice	*F. asiaticum*	3ADON	30.94522	118.828377	15		Commercial farm
14050	2014	Anhui	Rice	*F. asiaticum*	NIV	30.94522	118.828377	15		Commercial farm
14049	2014	Anhui	Rice	*F. asiaticum*	3ADON	30.94522	118.828377	15		Commercial farm
14048	2014	Anhui	Rice	*F. asiaticum*	3ADON	30.94522	118.828377	15		Commercial farm
14047	2014	Anhui	Rice	*F. asiaticum*	3ADON	30.94522	118.828377	15		Commercial farm
14044	2014	Anhui	Rice	*F. asiaticum*	3ADON	30.94522	118.828377	15		Commercial farm
14043	2014	Anhui	Rice	*F. asiaticum*	3ADON	30.94522	118.828377	15		Commercial farm
14042	2014	Anhui	Rice	*F. asiaticum*	3ADON	30.94522	118.828377	15		Commercial farm
14041	2014	Anhui	Rice	*F. asiaticum*	3ADON	30.94522	118.828377	15		Commercial farm
14040	2014	Anhui	Rice	*F. asiaticum*	3ADON	30.94522	118.828377	15		Commercial farm
14038	2014	Anhui	Rice	*F. asiaticum*	3ADON	30.94522	118.828377	15		Commercial farm
14037	2014	Anhui	Rice	*F. asiaticum*	NIV	30.94522	118.828377	15		Commercial farm

14036	2014	Anhui	Rice	*F. asiaticum*	3ADON	30.94522	118.828377	15		Commercial farm
14033	2014	Anhui	Rice	*F. asiaticum*	NIV	30.94522	118.828377	15		Commercial farm
14032	2014	Anhui	Rice	*F. asiaticum*	3ADON	30.94522	118.828377	15		Commercial farm
14031	2014	Anhui	Rice	*F. asiaticum*	3ADON	30.94522	118.828377	15		Commercial farm
14030	2014	Anhui	Rice	*F. asiaticum*	NIV	30.94522	118.828377	15		Commercial farm
14029	2014	Anhui	Rice	*F. asiaticum*	NIV	30.94522	118.828377	15		Commercial farm
14028	2014	Anhui	Rice	*F. asiaticum*	3ADON	30.94522	118.828377	15		Commercial farm
14027	2014	Anhui	Rice	*F. asiaticum*	NIV	30.94522	118.828377	15		Commercial farm
14026	2014	Anhui	Rice	*F. asiaticum*	3ADON	30.94522	118.828377	15		Commercial farm
14025	2014	Anhui	Rice	*F. asiaticum*	3ADON	30.94522	118.828377	15		Commercial farm
14024	2014	Anhui	Rice	*F. asiaticum*	3ADON	30.94522	118.828377	15		Commercial farm
14019	2014	Anhui	Rice	*F. asiaticum*	3ADON	30.94522	118.828377	15		Commercial farm
14013	2014	Anhui	Rice	*F. asiaticum*	3ADON	30.94522	118.828377	15		Commercial farm
14011	2014	Anhui	Rice	*F. asiaticum*	3ADON	30.94522	118.828377	15		Commercial farm
14006	2014	Anhui	Rice	*F. asiaticum*	NIV	30.94522	118.828377	15		Commercial farm
14004	2014	Anhui	Rice	*F. asiaticum*	3ADON	30.94522	118.828377	15		Commercial farm
14003	2014	Anhui	Rice	*F. asiaticum*	NIV	30.94522	118.828377	15		Commercial farm
192054	2019	Anhui	Wheat	*F. asiaticum*	3ADON	31.44939297	117.345819	9.277822	Commercialized variety	Farmer owned field
192053	2019	Anhui	Wheat	*F. asiaticum*	3ADON	31.44939297	117.345819	9.277822	Commercialized variety	Farmer owned field
192052	2019	Anhui	Wheat	*F. asiaticum*	NIV	31.44939297	117.345819	9.277822	Commercialized variety	Farmer owned field
192049	2019	Anhui	Wheat	*F. asiaticum*	3ADON	31.44939297	117.345819	9.277822	Commercialized variety	Farmer owned field
192048	2019	Anhui	Wheat	*F. asiaticum*	3ADON	31.44939297	117.345819	9.277822	Commercialized variety	Farmer owned field
192047	2019	Anhui	Wheat	*F. asiaticum*	3ADON	31.44939297	117.345819	9.277822	Commercialized variety	Farmer owned field
192046	2019	Anhui	Wheat	*F. asiaticum*	3ADON	31.44939297	117.345819	9.277822	Commercialized variety	Farmer owned field
192045	2019	Anhui	Wheat	*F. asiaticum*	3ADON	31.44939297	117.345819	9.277822	Commercialized variety	Farmer owned field
192044	2019	Anhui	Wheat	*F. asiaticum*	NIV	31.44939297	117.345819	9.277822	Commercialized variety	Farmer owned field
192043	2019	Anhui	Wheat	*F. asiaticum*	3ADON	31.44939297	117.345819	9.277822	Commercialized variety	Farmer owned field
192042	2019	Anhui	Wheat	*F. asiaticum*	3ADON	31.44939297	117.345819	9.277822	Commercialized variety	Farmer owned field
192060	2019	Anhui	Wheat	*F. asiaticum*	3ADON	31.48428096	117.22002	12.403226	Commercialized variety	Farmer owned field
192059	2019	Anhui	Wheat	*F. asiaticum*	3ADON	31.48428096	117.22002	12.403226	Commercialized variety	Farmer owned field
192057	2019	Anhui	Wheat	*F. asiaticum*	3ADON	31.48428096	117.22002	12.403226	Commercialized variety	Farmer owned field
192056	2019	Anhui	Wheat	*F. asiaticum*	15ADON	31.48428096	117.22002	12.403226	Commercialized variety	Farmer owned field
191324	2019	Anhui	Wheat	*F. asiaticum*	NIV	31.48428096	117.22002	12.403226	Commercialized variety	Farmer owned field
191323	2019	Anhui	Wheat	*F. asiaticum*	3ADON	31.48428096	117.22002	12.403226	Commercialized variety	Farmer owned field
191322	2019	Anhui	Wheat	*F. asiaticum*	3ADON	31.48428096	117.22002	12.403226	Commercialized variety	Farmer owned field

191321	2019	Anhui	Wheat	*F. asiaticum*	3ADON	31.48428096	117.22002	12.403226	Commercialized variety	Farmer owned field
191320	2019	Anhui	Wheat	*F. asiaticum*	3ADON	31.48428096	117.22002	12.403226	Commercialized variety	Farmer owned field
191319	2019	Anhui	Wheat	*F. asiaticum*	3ADON	31.48428096	117.22002	12.403226	Commercialized variety	Farmer owned field
191318	2019	Anhui	Wheat	*F. asiaticum*	NIV	31.48428096	117.22002	12.403226	Commercialized variety	Farmer owned field
191317_2	2019	Anhui	Wheat	*F. asiaticum*	3ADON	31.48428096	117.22002	12.403226	Commercialized variety	Farmer owned field
191316	2019	Anhui	Wheat	*F. asiaticum*	3ADON	31.48428096	117.22002	12.403226	Commercialized variety	Farmer owned field
191315	2019	Anhui	Wheat	*F. asiaticum*	3ADON	31.48428096	117.22002	12.403226	Commercialized variety	Farmer owned field
191314	2019	Anhui	Wheat	*F. asiaticum*	NIV	31.48428096	117.22002	12.403226	Commercialized variety	Farmer owned field
191313	2019	Anhui	Wheat	*F. asiaticum*	3ADON	31.48428096	117.22002	12.403226	Commercialized variety	Farmer owned field
191312	2019	Anhui	Wheat	*F. asiaticum*	3ADON	31.48428096	117.22002	12.403226	Commercialized variety	Farmer owned field
180509	2018	Hubei	Rice	*F. asiaticum*	15ADON	30.23301403	112.352811	28.518013		Commercial farm
180508	2018	Hubei	Rice	*F. asiaticum*	NIV	30.23301403	112.352811	28.518013		Commercial farm
180507	2018	Hubei	Rice	*F. asiaticum*	NIV	30.23301403	112.352811	28.518013		Commercial farm
180506	2018	Hubei	Rice	*F. asiaticum*	3ADON	30.23301403	112.352811	28.518013		Commercial farm
180505	2018	Hubei	Rice	*F. asiaticum*	3ADON	30.23301403	112.352811	28.518013		Commercial farm
191310	2019	Anhui	Wheat	*F. asiaticum*	3ADON	31.48428096	117.22002	12.403226	Commercialized variety	Farmer owned field
191309	2019	Anhui	Wheat	*F. asiaticum*	3ADON	31.48428096	117.22002	12.403226	Commercialized variety	Farmer owned field
191308	2019	Anhui	Wheat	*F. asiaticum*	3ADON	31.48428096	117.22002	12.403226	Commercialized variety	Farmer owned field
191307	2019	Anhui	Wheat	*F. asiaticum*	3ADON	31.48428096	117.22002	12.403226	Commercialized variety	Farmer owned field
191306	2019	Anhui	Wheat	*F. asiaticum*	3ADON	31.48428096	117.22002	12.403226	Commercialized variety	Farmer owned field
191565	2019	Anhui	Wheat	*F. asiaticum*	15ADON	32.188979	117.119964	67.842056	Commercialized variety	Farmer owned field
191564	2019	Anhui	Wheat	*F. asiaticum*	3ADON	32.188979	117.119964	67.842056	Commercialized variety	Farmer owned field
191563	2019	Anhui	Wheat	*F. asiaticum*	3ADON	32.188979	117.119964	67.842056	Commercialized variety	Farmer owned field
191562	2019	Anhui	Wheat	*F. asiaticum*	NIV	32.188979	117.119964	67.842056	Commercialized variety	Farmer owned field
191561	2019	Anhui	Wheat	*F. asiaticum*	NIV	32.188979	117.119964	67.842056	Commercialized variety	Farmer owned field
191559	2019	Anhui	Wheat	*F. asiaticum*	NIV	32.188979	117.119964	67.842056	Commercialized variety	Farmer owned field
191558	2019	Anhui	Wheat	*F. asiaticum*	3ADON	32.188979	117.119964	67.842056	Commercialized variety	Farmer owned field
191557	2019	Anhui	Wheat	*F. asiaticum*	3ADON	32.188979	117.119964	67.842056	Commercialized variety	Farmer owned field
191556	2019	Anhui	Wheat	*F. asiaticum*	3ADON	32.188979	117.119964	67.842056	Commercialized variety	Farmer owned field
191555	2019	Anhui	Wheat	*F. asiaticum*	15ADON	32.188979	117.119964	67.842056	Commercialized variety	Farmer owned field
191554	2019	Anhui	Wheat	*F. asiaticum*	3ADON	32.188979	117.119964	67.842056	Commercialized variety	Farmer owned field
191553	2019	Anhui	Wheat	*F. asiaticum*	3ADON	32.188979	117.119964	67.842056	Commercialized variety	Farmer owned field
191552	2019	Anhui	Wheat	*F. asiaticum*	3ADON	32.188979	117.119964	67.842056	Commercialized variety	Farmer owned field
191551	2019	Anhui	Wheat	*F. asiaticum*	3ADON	32.188979	117.119964	67.842056	Commercialized variety	Farmer owned field
191550	2019	Anhui	Wheat	*F. asiaticum*	3ADON	32.188979	117.119964	67.842056	Commercialized variety	Farmer owned field

191549	2019	Anhui	Wheat	*F. asiaticum*	3ADON	32.188979	117.119964	67.842056	Commercialized variety	Farmer owned field
194634	2019	Anhui	Wheat	*F. asiaticum*	3ADON	32.276687	118.393262	NA	Commercialized variety	Farmer owned field
194633	2019	Anhui	Wheat	*F. asiaticum*	3ADON	32.276687	118.393262	NA	Commercialized variety	Farmer owned field
180504	2018	Hubei	Rice	*F. asiaticum*	3ADON	30.23301403	112.352811	28.518013		Commercial farm
180502	2018	Hubei	Rice	*F. asiaticum*	NIV	30.23301403	112.352811	28.518013		Commercial farm
180501	2018	Hubei	Rice	*F. asiaticum*	3ADON	30.23301403	112.352811	28.518013		Commercial farm
180500	2018	Hubei	Rice	*F. asiaticum*	3ADON	30.23301403	112.352811	28.518013		Commercial farm
180499	2018	Hubei	Rice	*F. asiaticum*	NIV	30.23301403	112.352811	28.518013		Commercial farm
180498	2018	Hubei	Rice	*F. asiaticum*	NIV	30.23301403	112.352811	28.518013		Commercial farm
180497	2018	Hubei	Rice	*F. asiaticum*	3ADON	30.23301403	112.352811	28.518013		Commercial farm
180496	2018	Hubei	Rice	*F. asiaticum*	NIV	30.23301403	112.352811	28.518013		Commercial farm
180495	2018	Hubei	Rice	*F. asiaticum*	3ADON	30.23301403	112.352811	28.518013		Commercial farm
180494	2018	Hubei	Rice	*F. asiaticum*	3ADON	30.23301403	112.352811	28.518013		Commercial farm
180493	2018	Hubei	Rice	*F. asiaticum*	3ADON	30.23301403	112.352811	28.518013		Commercial farm
180492	2018	Hubei	Rice	*F. asiaticum*	3ADON	30.23301403	112.352811	28.518013		Commercial farm
JSYZ065	2014	Jiangsu	Rice	*F. asiaticum*	3ADON	32.270763	119.254133	4		Commercial farm
JSYZ064	2014	Jiangsu	Rice	*F. asiaticum*	3ADON	32.270763	119.254133	4		Commercial farm
JSYZ063	2014	Jiangsu	Rice	*F. asiaticum*	3ADON	32.270763	119.254133	4		Commercial farm
JSYZ062	2014	Jiangsu	Rice	*F. asiaticum*	3ADON	32.270763	119.254133	4		Commercial farm
JSYZ061	2014	Jiangsu	Rice	*F. asiaticum*	3ADON	32.270763	119.254133	4		Commercial farm
JSYZ056	2014	Jiangsu	Rice	*F. asiaticum*	NIV	32.270763	119.254133	4		Commercial farm
JSYZ055	2014	Jiangsu	Rice	*F. asiaticum*	NIV	32.270763	119.254133	4		Commercial farm
JSYZ054	2014	Jiangsu	Rice	*F. asiaticum*	3ADON	32.270763	119.254133	4		Commercial farm
JSYZ049	2014	Jiangsu	Rice	*F. asiaticum*	3ADON	32.270763	119.254133	4		Commercial farm
JSYZ045	2014	Jiangsu	Rice	*F. asiaticum*	NIV	32.270763	119.254133	4		Commercial farm
JSYZ043	2014	Jiangsu	Rice	*F. asiaticum*	3ADON	32.270763	119.254133	4		Commercial farm
JSYZ042	2014	Jiangsu	Rice	*F. asiaticum*	3ADON	32.270763	119.254133	4		Commercial farm
JSYZ041	2014	Jiangsu	Rice	*F. asiaticum*	NIV	32.270763	119.254133	4		Commercial farm
JSYZ040	2014	Jiangsu	Rice	*F. asiaticum*	3ADON	32.270763	119.254133	4		Commercial farm
JSYZ039	2014	Jiangsu	Rice	*F. asiaticum*	3ADON	32.270763	119.254133	4		Commercial farm
JSYZ038	2014	Jiangsu	Rice	*F. asiaticum*	3ADON	32.270763	119.254133	4		Commercial farm
JSYZ037	2014	Jiangsu	Rice	*F. asiaticum*	NIV	32.270763	119.254133	4		Commercial farm
JSYZ032	2014	Jiangsu	Rice	*F. asiaticum*	3ADON	32.270763	119.254133	4		Commercial farm
160226	2016	Anhui	Rice	*F. asiaticum*	3ADON	32.353038	116.277911	36		Farmer owned field
160232	2016	Anhui	Rice	*F. asiaticum*	3ADON	32.353038	116.277911	36		Farmer owned field

191076	2019	Anhui	Wheat	*F. asiaticum*	3ADON	32.39227703	117.737076	62.992245	Commercialized variety	Farmer owned field
191075	2019	Anhui	Wheat	*F. asiaticum*	NIV	32.39227703	117.737076	62.992245	Commercialized variety	Farmer owned field
191074	2019	Anhui	Wheat	*F. asiaticum*	3ADON	32.39227703	117.737076	62.992245	Commercialized variety	Farmer owned field
191073	2019	Anhui	Wheat	*F. asiaticum*	3ADON	32.39227703	117.737076	62.992245	Commercialized variety	Farmer owned field
191071	2019	Anhui	Wheat	*F. asiaticum*	3ADON	32.39227703	117.737076	62.992245	Commercialized variety	Farmer owned field
191070	2019	Anhui	Wheat	*F. asiaticum*	3ADON	32.39227703	117.737076	62.992245	Commercialized variety	Farmer owned field
191069	2019	Anhui	Wheat	*F. asiaticum*	3ADON	32.39227703	117.737076	62.992245	Commercialized variety	Farmer owned field
191068	2019	Anhui	Wheat	*F. asiaticum*	3ADON	32.39227703	117.737076	62.992245	Commercialized variety	Farmer owned field
191067	2019	Anhui	Wheat	*F. asiaticum*	3ADON	32.39227703	117.737076	62.992245	Commercialized variety	Farmer owned field
191066	2019	Anhui	Wheat	*F. asiaticum*	3ADON	32.39227703	117.737076	62.992245	Commercialized variety	Farmer owned field
191065	2019	Anhui	Wheat	*F. asiaticum*	3ADON	32.39227703	117.737076	62.992245	Commercialized variety	Farmer owned field
191064	2019	Anhui	Wheat	*F. asiaticum*	3ADON	32.39227703	117.737076	62.992245	Commercialized variety	Farmer owned field
191063	2019	Anhui	Wheat	*F. asiaticum*	3ADON	32.39227703	117.737076	62.992245	Commercialized variety	Farmer owned field
191062	2019	Anhui	Wheat	*F. asiaticum*	3ADON	32.39227703	117.737076	62.992245	Commercialized variety	Farmer owned field
191061	2019	Anhui	Wheat	*F. asiaticum*	3ADON	32.39227703	117.737076	62.992245	Commercialized variety	Farmer owned field
191060	2019	Anhui	Wheat	*F. asiaticum*	3ADON	32.39227703	117.737076	62.992245	Commercialized variety	Farmer owned field
191059	2019	Anhui	Wheat	*F. asiaticum*	3ADON	32.39227703	117.737076	62.992245	Commercialized variety	Farmer owned field
191058	2019	Anhui	Wheat	*F. asiaticum*	3ADON	32.39227703	117.737076	62.992245	Commercialized variety	Farmer owned field
191057	2019	Anhui	Wheat	*F. asiaticum*	3ADON	32.39227703	117.737076	62.992245	Commercialized variety	Farmer owned field
JSYZ028	2014	Jiangsu	Rice	*F. asiaticum*	3ADON	32.270763	119.254133	4		Commercial farm
JSYZ023	2014	Jiangsu	Rice	*F. asiaticum*	3ADON	32.270763	119.254133	4		Commercial farm
JSYZ022	2014	Jiangsu	Rice	*F. asiaticum*	3ADON	32.270763	119.254133	4		Commercial farm
JSYZ021	2014	Jiangsu	Rice	*F. asiaticum*	3ADON	32.270763	119.254133	4		Commercial farm
JSYZ020	2014	Jiangsu	Rice	*F. asiaticum*	3ADON	32.270763	119.254133	4		Commercial farm
JSYZ018	2014	Jiangsu	Rice	*F. asiaticum*	3ADON	32.270763	119.254133	4		Commercial farm
JSYZ017	2014	Jiangsu	Rice	*F. asiaticum*	15ADON	32.270763	119.254133	4		Commercial farm
JSYZ013	2014	Jiangsu	Rice	*F. asiaticum*	3ADON	32.270763	119.254133	4		Commercial farm
JSYZ011	2014	Jiangsu	Rice	*F. asiaticum*	3ADON	32.270763	119.254133	4		Commercial farm
JSYZ010	2014	Jiangsu	Rice	*F. asiaticum*	3ADON	32.270763	119.254133	4		Commercial farm
JSYZ008	2014	Jiangsu	Rice	*F. asiaticum*	NIV	32.270763	119.254133	4		Commercial farm
JSYZ007	2014	Jiangsu	Rice	*F. asiaticum*	3ADON	32.270763	119.254133	4		Commercial farm
JSYZ006	2014	Jiangsu	Rice	*F. asiaticum*	3ADON	32.270763	119.254133	4		Commercial farm
JSYZ002	2014	Jiangsu	Rice	*F. asiaticum*	3ADON	32.270763	119.254133	4		Commercial farm
160952	2016	Jiangsu	Rice	*F. asiaticum*	3ADON	32.3731728	120.5719417	6		Farmer owned field
182500	2018	Jiangsu	Wheat	*F. asiaticum*	3ADON	32.48539302	118.624212	82.081017	Commercialized variety	Farmer owned field

182499	2018	Jiangsu	Wheat	*F. asiaticum*	3ADON	32.48539302	118.624212	82.081017	Commercialized variety	Farmer owned field
182498	2018	Jiangsu	Wheat	*F. asiaticum*	3ADON	32.48539302	118.624212	82.081017	Commercialized variety	Farmer owned field
182497	2018	Jiangsu	Wheat	*F. asiaticum*	3ADON	32.48539302	118.624212	82.081017	Commercialized variety	Farmer owned field
182496	2018	Jiangsu	Wheat	*F. asiaticum*	3ADON	32.48539302	118.624212	82.081017	Commercialized variety	Farmer owned field
194631	2019	Anhui	Wheat	*F. asiaticum*	3ADON	32.465517	118.445866	NA	Commercialized variety	Farmer owned field
194630	2019	Anhui	Wheat	*F. asiaticum*	3ADON	32.465517	118.445866	NA	Commercialized variety	Farmer owned field
194628	2019	Anhui	Wheat	*F. asiaticum*	3ADON	32.465517	118.445866	NA	Commercialized variety	Farmer owned field
194627	2019	Anhui	Wheat	*F. asiaticum*	3ADON	32.465517	118.445866	NA	Commercialized variety	Farmer owned field
194626	2019	Anhui	Wheat	*F. asiaticum*	3ADON	32.465517	118.445866	NA	Commercialized variety	Farmer owned field
194625	2019	Anhui	Wheat	*F. asiaticum*	3ADON	32.465517	118.445866	NA	Commercialized variety	Farmer owned field
194624	2019	Anhui	Wheat	*F. asiaticum*	3ADON	32.465517	118.445866	NA	Commercialized variety	Farmer owned field
194623	2019	Anhui	Wheat	*F. asiaticum*	3ADON	32.465517	118.445866	NA	Commercialized variety	Farmer owned field
194622	2019	Anhui	Wheat	*F. asiaticum*	3ADON	32.465517	118.445866	NA	Commercialized variety	Farmer owned field
194621	2019	Anhui	Wheat	*F. asiaticum*	3ADON	32.465517	118.445866	NA	Commercialized variety	Farmer owned field
194620	2019	Anhui	Wheat	*F. asiaticum*	3ADON	32.465517	118.445866	NA	Commercialized variety	Farmer owned field
194619	2019	Anhui	Wheat	*F. asiaticum*	3ADON	32.465517	118.445866	NA	Commercialized variety	Farmer owned field
194618	2019	Anhui	Wheat	*F. asiaticum*	3ADON	32.465517	118.445866	NA	Commercialized variety	Farmer owned field
194617	2019	Anhui	Wheat	*F. asiaticum*	3ADON	32.465517	118.445866	NA	Commercialized variety	Farmer owned field
194616	2019	Anhui	Wheat	*F. asiaticum*	3ADON	32.465517	118.445866	NA	Commercialized variety	Farmer owned field
194615	2019	Anhui	Wheat	*F. asiaticum*	3ADON	32.465517	118.445866	NA	Commercialized variety	Farmer owned field
194614	2019	Anhui	Wheat	*F. asiaticum*	3ADON	32.465517	118.445866	NA	Commercialized variety	Farmer owned field
194613	2019	Anhui	Wheat	*F. asiaticum*	3ADON	32.465517	118.445866	NA	Commercialized variety	Farmer owned field
194612	2019	Anhui	Wheat	*F. asiaticum*	3ADON	32.465517	118.445866	NA	Commercialized variety	Farmer owned field
191508	2019	Anhui	Wheat	*F. asiaticum*	3ADON	32.58042402	116.82401	47.643768	Commercialized variety	Farmer owned field
191507	2019	Anhui	Wheat	*F. asiaticum*	3ADON	32.58042402	116.82401	47.643768	Commercialized variety	Farmer owned field
191506	2019	Anhui	Wheat	*F. asiaticum*	NIV	32.58042402	116.82401	47.643768	Commercialized variety	Farmer owned field
191505	2019	Anhui	Wheat	*F. asiaticum*	NIV	32.58042402	116.82401	47.643768	Commercialized variety	Farmer owned field
191502	2019	Anhui	Wheat	*F. asiaticum*	15ADON	32.58042402	116.82401	47.643768	Commercialized variety	Farmer owned field
191501	2019	Anhui	Wheat	*F. asiaticum*	NIV	32.58042402	116.82401	47.643768	Commercialized variety	Farmer owned field
191499	2019	Anhui	Wheat	*F. asiaticum*	NIV	32.58042402	116.82401	47.643768	Commercialized variety	Farmer owned field
191498	2019	Anhui	Wheat	*F. asiaticum*	NIV	32.58042402	116.82401	47.643768	Commercialized variety	Farmer owned field
191494	2019	Anhui	Wheat	*F. asiaticum*	3ADON	32.58042402	116.82401	47.643768	Commercialized variety	Farmer owned field
191493	2019	Anhui	Wheat	*F. asiaticum*	NIV	32.58042402	116.82401	47.643768	Commercialized variety	Farmer owned field
191492	2019	Anhui	Wheat	*F. asiaticum*	NIV	32.58042402	116.82401	47.643768	Commercialized variety	Farmer owned field
191490	2019	Anhui	Wheat	*F. asiaticum*	NIV	32.58042402	116.82401	47.643768	Commercialized variety	Farmer owned field

191488	2019	Anhui	Wheat	*F. asiaticum*	3ADON	32.58042402	116.82401	47.643768	Commercialized variety	Farmer owned field
191484	2019	Anhui	Wheat	*F. asiaticum*	3ADON	32.58042402	116.82401	47.643768	Commercialized variety	Farmer owned field
191483	2019	Anhui	Wheat	*F. asiaticum*	3ADON	32.58042402	116.82401	47.643768	Commercialized variety	Farmer owned field
191482	2019	Anhui	Wheat	*F. asiaticum*	NIV	32.58042402	116.82401	47.643768	Commercialized variety	Farmer owned field
190682	2019	Anhui	Wheat	*F. asiaticum*	3ADON	32.61739399	118.149873	114.290421	Commercialized variety	Farmer owned field
190681	2019	Anhui	Wheat	*F. asiaticum*	3ADON	32.61739399	118.149873	114.290421	Commercialized variety	Farmer owned field
190680	2019	Anhui	Wheat	*F. asiaticum*	3ADON	32.61739399	118.149873	114.290421	Commercialized variety	Farmer owned field
190677	2019	Anhui	Wheat	*F. asiaticum*	NIV	32.61739399	118.149873	114.290421	Commercialized variety	Farmer owned field
190676	2019	Anhui	Wheat	*F. asiaticum*	3ADON	32.61739399	118.149873	114.290421	Commercialized variety	Farmer owned field
190674	2019	Anhui	Wheat	*F. asiaticum*	3ADON	32.61739399	118.149873	114.290421	Commercialized variety	Farmer owned field
190671	2019	Anhui	Wheat	*F. asiaticum*	NIV	32.61739399	118.149873	114.290421	Commercialized variety	Farmer owned field
190670	2019	Anhui	Wheat	*F. asiaticum*	3ADON	32.61739399	118.149873	114.290421	Commercialized variety	Farmer owned field
190669	2019	Anhui	Wheat	*F. asiaticum*	3ADON	32.61739399	118.149873	114.290421	Commercialized variety	Farmer owned field
190668	2019	Anhui	Wheat	*F. asiaticum*	3ADON	32.61739399	118.149873	114.290421	Commercialized variety	Farmer owned field
190667	2019	Anhui	Wheat	*F. asiaticum*	3ADON	32.61739399	118.149873	114.290421	Commercialized variety	Farmer owned field
191272	2019	Anhui	Wheat	*F. asiaticum*	NIV	32.64513499	116.401027	18.684174	Commercialized variety	Farmer owned field
191271	2019	Anhui	Wheat	*F. asiaticum*	3ADON	32.64513499	116.401027	18.684174	Commercialized variety	Farmer owned field
191270	2019	Anhui	Wheat	*F. asiaticum*	3ADON	32.64513499	116.401027	18.684174	Commercialized variety	Farmer owned field
191269	2019	Anhui	Wheat	*F. asiaticum*	3ADON	32.64513499	116.401027	18.684174	Commercialized variety	Farmer owned field
191267	2019	Anhui	Wheat	*F. asiaticum*	NIV	32.64513499	116.401027	18.684174	Commercialized variety	Farmer owned field
191266	2019	Anhui	Wheat	*F. asiaticum*	3ADON	32.64513499	116.401027	18.684174	Commercialized variety	Farmer owned field
191264	2019	Anhui	Wheat	*F. asiaticum*	3ADON	32.64513499	116.401027	18.684174	Commercialized variety	Farmer owned field
191262	2019	Anhui	Wheat	*F. asiaticum*	3ADON	32.64513499	116.401027	18.684174	Commercialized variety	Farmer owned field
191261	2019	Anhui	Wheat	*F. asiaticum*	3ADON	32.64513499	116.401027	18.684174	Commercialized variety	Farmer owned field
191260	2019	Anhui	Wheat	*F. asiaticum*	3ADON	32.64513499	116.401027	18.684174	Commercialized variety	Farmer owned field
191259	2019	Anhui	Wheat	*F. asiaticum*	3ADON	32.64513499	116.401027	18.684174	Commercialized variety	Farmer owned field
191258	2019	Anhui	Wheat	*F. asiaticum*	3ADON	32.64513499	116.401027	18.684174	Commercialized variety	Farmer owned field
191257	2019	Anhui	Wheat	*F. asiaticum*	3ADON	32.64513499	116.401027	18.684174	Commercialized variety	Farmer owned field
191256	2019	Anhui	Wheat	*F. asiaticum*	3ADON	32.64513499	116.401027	18.684174	Commercialized variety	Farmer owned field
191255	2019	Anhui	Wheat	*F. asiaticum*	3ADON	32.64513499	116.401027	18.684174	Commercialized variety	Farmer owned field
191254	2019	Anhui	Wheat	*F. asiaticum*	3ADON	32.64513499	116.401027	18.684174	Commercialized variety	Farmer owned field
191252	2019	Anhui	Wheat	*F. asiaticum*	3ADON	32.64513499	116.401027	18.684174	Commercialized variety	Farmer owned field
191251	2019	Anhui	Wheat	*F. asiaticum*	3ADON	32.64513499	116.401027	18.684174	Commercialized variety	Farmer owned field
191249	2019	Anhui	Wheat	*F. asiaticum*	3ADON	32.64513499	116.401027	18.684174	Commercialized variety	Farmer owned field
191247	2019	Anhui	Wheat	*F. asiaticum*	3ADON	32.64513499	116.401027	18.684174	Commercialized variety	Farmer owned field

191246	2019	Anhui	Wheat	*F. asiaticum*	3ADON	32.64513499	116.401027	18.684174	Commercialized variety	Farmer owned field
160237	2016	Anhui	Rice	*F. asiaticum*	3ADON	32.713877	116.704162	22		Farmer owned field
160242	2016	Anhui	Rice	*F. asiaticum*	3ADON	32.713877	116.704162	22		Farmer owned field
160264	2016	Anhui	Rice	*F. asiaticum*	3ADON	32.713877	116.704162	22		Farmer owned field
160270	2016	Anhui	Rice	*F. asiaticum*	3ADON	32.713877	116.704162	22		Farmer owned field
160277	2016	Anhui	Rice	*F. asiaticum*	3ADON	32.713877	116.704162	22		Farmer owned field
160280	2016	Anhui	Rice	*F. asiaticum*	3ADON	32.713877	116.704162	22		Farmer owned field
160282	2016	Anhui	Rice	*F. asiaticum*	3ADON	32.713877	116.704162	22		Farmer owned field
AH99-1	2014	Anhui	Wheat	*F. asiaticum*	3ADON	32.77738333	116.6282333	26	Commercialized variety	Commercial farm
AH98	2014	Anhui	Wheat	*F. asiaticum*	3ADON	32.77738333	116.6282333	26	Commercialized variety	Commercial farm
AH89	2014	Anhui	Wheat	*F. asiaticum*	3ADON	32.77738333	116.6282333	26	Commercialized variety	Commercial farm
AH88	2014	Anhui	Wheat	*F. asiaticum*	3ADON	32.77738333	116.6282333	26	Commercialized variety	Commercial farm
AH83	2014	Anhui	Wheat	*F. asiaticum*	3ADON	32.77738333	116.6282333	26	Commercialized variety	Commercial farm
AH82	2014	Anhui	Wheat	*F. asiaticum*	3ADON	32.77738333	116.6282333	26	Commercialized variety	Commercial farm
AH76	2014	Anhui	Wheat	*F. asiaticum*	NIV	32.77738333	116.6282333	26	Commercialized variety	Commercial farm
AH75-1	2014	Anhui	Wheat	*F. asiaticum*	NIV	32.77738333	116.6282333	26	Commercialized variety	Commercial farm
AH72	2014	Anhui	Wheat	*F. asiaticum*	3ADON	32.77738333	116.6282333	26	Commercialized variety	Commercial farm
AH71-1	2014	Anhui	Wheat	*F. asiaticum*	3ADON	32.77738333	116.6282333	26	Commercialized variety	Commercial farm
AH7-1	2014	Anhui	Wheat	*F. asiaticum*	3ADON	32.77738333	116.6282333	26	Commercialized variety	Commercial farm
AH65	2014	Anhui	Wheat	*F. asiaticum*	3ADON	32.77738333	116.6282333	26	Commercialized variety	Commercial farm
AH6-1	2014	Anhui	Wheat	*F. asiaticum*	NIV	32.77738333	116.6282333	26	Commercialized variety	Commercial farm
AH54-1	2014	Anhui	Wheat	*F. asiaticum*	3ADON	32.77738333	116.6282333	26	Commercialized variety	Commercial farm
AH51-2	2014	Anhui	Wheat	*F. asiaticum*	3ADON	32.77738333	116.6282333	26	Commercialized variety	Commercial farm
AH47-2	2014	Anhui	Wheat	*F. asiaticum*	3ADON	32.77738333	116.6282333	26	Commercialized variety	Commercial farm
AH45-1	2014	Anhui	Wheat	*F. asiaticum*	NIV	32.77738333	116.6282333	26	Commercialized variety	Commercial farm
AH44-2	2014	Anhui	Wheat	*F. asiaticum*	3ADON	32.77738333	116.6282333	26	Commercialized variety	Commercial farm
AH33-2	2014	Anhui	Wheat	*F. asiaticum*	3ADON	32.77738333	116.6282333	26	Commercialized variety	Commercial farm
AH33-1	2014	Anhui	Wheat	*F. asiaticum*	3ADON	32.77738333	116.6282333	26	Commercialized variety	Commercial farm
AH3-2	2014	Anhui	Wheat	*F. asiaticum*	3ADON	32.77738333	116.6282333	26	Commercialized variety	Commercial farm
AH31-2	2014	Anhui	Wheat	*F. asiaticum*	3ADON	32.77738333	116.6282333	26	Commercialized variety	Commercial farm
AH28-1	2014	Anhui	Wheat	*F. asiaticum*	3ADON	32.77738333	116.6282333	26	Commercialized variety	Commercial farm
AH27-1	2014	Anhui	Wheat	*F. asiaticum*	3ADON	32.77738333	116.6282333	26	Commercialized variety	Commercial farm
AH24	2014	Anhui	Wheat	*F. asiaticum*	3ADON	32.77738333	116.6282333	26	Commercialized variety	Commercial farm
AH23-1	2014	Anhui	Wheat	*F. asiaticum*	3ADON	32.77738333	116.6282333	26	Commercialized variety	Commercial farm
AH20-1	2014	Anhui	Wheat	*F. asiaticum*	NIV	32.77738333	116.6282333	26	Commercialized variety	Commercial farm

AH19-1	2014	Anhui	Wheat	*F. asiaticum*	3ADON	32.77738333	116.6282333	26	Commercialized variety	Commercial farm
AH189-2	2014	Anhui	Wheat	*F. asiaticum*	3ADON	32.77738333	116.6282333	26	Commercialized variety	Commercial farm
AH188-1	2014	Anhui	Wheat	*F. asiaticum*	3ADON	32.77738333	116.6282333	26	Commercialized variety	Commercial farm
AH18-1	2014	Anhui	Wheat	*F. asiaticum*	3ADON	32.77738333	116.6282333	26	Commercialized variety	Commercial farm
AH179-2	2014	Anhui	Wheat	*F. asiaticum*	3ADON	32.77738333	116.6282333	26	Commercialized variety	Commercial farm
AH176-2	2014	Anhui	Wheat	*F. asiaticum*	3ADON	32.77738333	116.6282333	26	Commercialized variety	Commercial farm
AH17-2	2014	Anhui	Wheat	*F. asiaticum*	3ADON	32.77738333	116.6282333	26	Commercialized variety	Commercial farm
AH171-1	2014	Anhui	Wheat	*F. asiaticum*	3ADON	32.77738333	116.6282333	26	Commercialized variety	Commercial farm
AH168-1	2014	Anhui	Wheat	*F. asiaticum*	3ADON	32.77738333	116.6282333	26	Commercialized variety	Commercial farm
AH165-1	2014	Anhui	Wheat	*F. asiaticum*	3ADON	32.77738333	116.6282333	26	Commercialized variety	Commercial farm
AH160	2014	Anhui	Wheat	*F. asiaticum*	3ADON	32.77738333	116.6282333	26	Commercialized variety	Commercial farm
AH159-2	2014	Anhui	Wheat	*F. asiaticum*	15ADON	32.77738333	116.6282333	26	Commercialized variety	Commercial farm
AH158-2	2014	Anhui	Wheat	*F. asiaticum*	NIV	32.77738333	116.6282333	26	Commercialized variety	Commercial farm
AH141-1	2014	Anhui	Wheat	*F. asiaticum*	3ADON	32.77738333	116.6282333	26	Commercialized variety	Commercial farm
AH14-1	2014	Anhui	Wheat	*F. asiaticum*	3ADON	32.77738333	116.6282333	26	Commercialized variety	Commercial farm
AH140-2	2014	Anhui	Wheat	*F. asiaticum*	3ADON	32.77738333	116.6282333	26	Commercialized variety	Commercial farm
AH123-1	2014	Anhui	Wheat	*F. asiaticum*	3ADON	32.77738333	116.6282333	26	Commercialized variety	Commercial farm
AH117	2014	Anhui	Wheat	*F. asiaticum*	3ADON	32.77738333	116.6282333	26	Commercialized variety	Commercial farm
AH116	2014	Anhui	Wheat	*F. asiaticum*	3ADON	32.77738333	116.6282333	26	Commercialized variety	Commercial farm
AH113	2014	Anhui	Wheat	*F. asiaticum*	3ADON	32.77738333	116.6282333	26	Commercialized variety	Commercial farm
AH110	2014	Anhui	Wheat	*F. asiaticum*	3ADON	32.77738333	116.6282333	26	Commercialized variety	Commercial farm
AH109	2014	Anhui	Wheat	*F. asiaticum*	NIV	32.77738333	116.6282333	26	Commercialized variety	Commercial farm
AH108	2014	Anhui	Wheat	*F. asiaticum*	3ADON	32.77738333	116.6282333	26	Commercialized variety	Commercial farm
AH106	2014	Anhui	Wheat	*F. asiaticum*	3ADON	32.77738333	116.6282333	26	Commercialized variety	Commercial farm
AH104-1	2014	Anhui	Wheat	*F. asiaticum*	3ADON	32.77738333	116.6282333	26	Commercialized variety	Commercial farm
AH012	2014	Anhui	Wheat	*F. asiaticum*	NIV	32.77738333	116.6282333	26	Commercialized variety	Commercial farm
AHFT14058	2014	Anhui	Rice	*F. asiaticum*	NIV	32.77738333	116.6282333	26		Commercial farm
AHFT14024	2014	Anhui	Rice	*F. asiaticum*	3ADON	32.77738333	116.6282333	26		Commercial farm
AHFT14023	2014	Anhui	Rice	*F. asiaticum*	NIV	32.77738333	116.6282333	26		Commercial farm
AHFT14022	2014	Anhui	Rice	*F. asiaticum*	NIV	32.77738333	116.6282333	26		Commercial farm
AHFT14021	2014	Anhui	Rice	*F. asiaticum*	NIV	32.77738333	116.6282333	26		Commercial farm
AHFT14017	2014	Anhui	Rice	*F. asiaticum*	NIV	32.77738333	116.6282333	26		Commercial farm
AHFT14016	2014	Anhui	Rice	*F. asiaticum*	3ADON	32.77738333	116.6282333	26		Commercial farm
AHFT14015	2014	Anhui	Rice	*F. asiaticum*	3ADON	32.77738333	116.6282333	26		Commercial farm
AHFT14014	2014	Anhui	Rice	*F. asiaticum*	3ADON	32.77738333	116.6282333	26		Commercial farm

AHFT14014	2014	Anhui	Rice	*F. asiaticum*	3ADON	32.77738333	116.6282333	26		Commercial farm
AHFT14013	2014	Anhui	Rice	*F. asiaticum*	3ADON	32.77738333	116.6282333	26		Commercial farm
AHFT14011	2014	Anhui	Rice	*F. asiaticum*	3ADON	32.77738333	116.6282333	26		Commercial farm
AHFT14010	2014	Anhui	Rice	*F. asiaticum*	3ADON	32.77738333	116.6282333	26		Commercial farm
AHFT14007	2014	Anhui	Rice	*F. asiaticum*	3ADON	32.77738333	116.6282333	26		Commercial farm
AHFT14005	2014	Anhui	Rice	*F. asiaticum*	3ADON	32.77738333	116.6282333	26		Commercial farm
AHFT14004	2014	Anhui	Rice	*F. asiaticum*	3ADON	32.77738333	116.6282333	26		Commercial farm
180491	2018	Hubei	Rice	*F. asiaticum*	3ADON	30.23301403	112.352811	28.518013		Commercial farm
180490	2018	Hubei	Rice	*F. asiaticum*	3ADON	30.23301403	112.352811	28.518013		Commercial farm
180489	2018	Hubei	Rice	*F. asiaticum*	3ADON	30.23301403	112.352811	28.518013		Commercial farm
180488	2018	Hubei	Rice	*F. asiaticum*	3ADON	30.23301403	112.352811	28.518013		Commercial farm
180487	2018	Hubei	Rice	*F. asiaticum*	3ADON	30.23301403	112.352811	28.518013		Commercial farm
180486	2018	Hubei	Rice	*F. asiaticum*	3ADON	30.23301403	112.352811	28.518013		Commercial farm
180485	2018	Hubei	Rice	*F. asiaticum*	3ADON	30.23301403	112.352811	28.518013		Commercial farm
180484	2018	Hubei	Rice	*F. asiaticum*	3ADON	30.23301403	112.352811	28.518013		Commercial farm
181111	2018	Hubei	Wheat	*F. asiaticum*	3ADON	30.36086798	112.058081	27.621157	Commercialized variety	Farmer owned field
181110	2018	Hubei	Wheat	*F. asiaticum*	3ADON	30.36086798	112.058081	27.621157	Commercialized variety	Farmer owned field
181109	2018	Hubei	Wheat	*F. asiaticum*	NIV	30.36086798	112.058081	27.621157	Commercialized variety	Farmer owned field
181108	2018	Hubei	Wheat	*F. asiaticum*	3ADON	30.36086798	112.058081	27.621157	Commercialized variety	Farmer owned field
181107	2018	Hubei	Wheat	*F. asiaticum*	3ADON	30.36086798	112.058081	27.621157	Commercialized variety	Farmer owned field
181106	2018	Hubei	Wheat	*F. asiaticum*	3ADON	30.36086798	112.058081	27.621157	Commercialized variety	Farmer owned field
181105	2018	Hubei	Wheat	*F. asiaticum*	3ADON	30.36086798	112.058081	27.621157	Commercialized variety	Farmer owned field
181104	2018	Hubei	Wheat	*F. asiaticum*	15ADON	30.36086798	112.058081	27.621157	Commercialized variety	Farmer owned field
181103	2018	Hubei	Wheat	*F. asiaticum*	3ADON	30.36086798	112.058081	27.621157	Commercialized variety	Farmer owned field
181102	2018	Hubei	Wheat	*F. asiaticum*	3ADON	30.36086798	112.058081	27.621157	Commercialized variety	Farmer owned field
181101	2018	Hubei	Wheat	*F. asiaticum*	3ADON	30.36086798	112.058081	27.621157	Commercialized variety	Farmer owned field
181100	2018	Hubei	Wheat	*F. asiaticum*	NIV	30.36086798	112.058081	27.621157	Commercialized variety	Farmer owned field
181099	2018	Hubei	Wheat	*F. asiaticum*	3ADON	30.36086798	112.058081	27.621157	Commercialized variety	Farmer owned field
181098	2018	Hubei	Wheat	*F. asiaticum*	3ADON	30.36086798	112.058081	27.621157	Commercialized variety	Farmer owned field
182495	2018	Jiangsu	Wheat	*F. asiaticum*	3ADON	32.48539302	118.624212	82.081017	Commercialized variety	Farmer owned field
182494	2018	Jiangsu	Wheat	*F. asiaticum*	3ADON	32.48539302	118.624212	82.081017	Commercialized variety	Farmer owned field
182493	2018	Jiangsu	Wheat	*F. asiaticum*	3ADON	32.48539302	118.624212	82.081017	Commercialized variety	Farmer owned field
191840	2019	Hubei	Wheat	*F. asiaticum*	3ADON	30.37905201	113.285998	38.968929	Commercialized variety	Farmer owned field
191839	2019	Hubei	Wheat	*F. asiaticum*	3ADON	30.37905201	113.285998	38.968929	Commercialized variety	Farmer owned field
191838	2019	Hubei	Wheat	*F. asiaticum*	3ADON	30.37905201	113.285998	38.968929	Commercialized variety	Farmer owned field

191934	2019	Hubei	Wheat	*F. asiaticum*	3ADON	30.43971403	113.753649	23.424345	Commercialized variety	Farmer owned field
191933	2019	Hubei	Wheat	*F. asiaticum*	3ADON	30.43971403	113.753649	23.424345	Commercialized variety	Farmer owned field
191932	2019	Hubei	Wheat	*F. asiaticum*	15ADON	30.43971403	113.753649	23.424345	Commercialized variety	Farmer owned field
191931	2019	Hubei	Wheat	*F. asiaticum*	3ADON	30.43971403	113.753649	23.424345	Commercialized variety	Farmer owned field
191930	2019	Hubei	Wheat	*F. asiaticum*	3ADON	30.43971403	113.753649	23.424345	Commercialized variety	Farmer owned field
191929	2019	Hubei	Wheat	*F. asiaticum*	3ADON	30.43971403	113.753649	23.424345	Commercialized variety	Farmer owned field
192907	2019	Jiangsu	Wheat	*F. asiaticum*	3ADON	32.49887698	119.624401	13.756882	Commercialized variety	Farmer owned field
192905	2019	Jiangsu	Wheat	*F. asiaticum*	3ADON	32.49887698	119.624401	13.756882	Commercialized variety	Farmer owned field
192899	2019	Jiangsu	Wheat	*F. asiaticum*	3ADON	32.49887698	119.624401	13.756882	Commercialized variety	Farmer owned field
193229	2019	Jiangsu	Wheat	*F. asiaticum*	3ADON	32.50654802	120.281255	27.485409	Commercialized variety	Farmer owned field
193228	2019	Jiangsu	Wheat	*F. asiaticum*	3ADON	32.50654802	120.281255	27.485409	Commercialized variety	Farmer owned field
193227	2019	Jiangsu	Wheat	*F. asiaticum*	3ADON	32.50654802	120.281255	27.485409	Commercialized variety	Farmer owned field
193226	2019	Jiangsu	Wheat	*F. asiaticum*	3ADON	32.50654802	120.281255	27.485409	Commercialized variety	Farmer owned field
182678	2018	Jiangsu	Wheat	*F. asiaticum*	NIV	32.50654802	120.281255	27.485409	Commercialized variety	Farmer owned field
182677	2018	Jiangsu	Wheat	*F. asiaticum*	3ADON	32.50654802	120.281255	27.485409	Commercialized variety	Farmer owned field
182676	2018	Jiangsu	Wheat	*F. asiaticum*	3ADON	32.50654802	120.281255	27.485409	Commercialized variety	Farmer owned field
182675	2018	Jiangsu	Wheat	*F. asiaticum*	3ADON	32.50654802	120.281255	27.485409	Commercialized variety	Farmer owned field
182674	2018	Jiangsu	Wheat	*F. asiaticum*	3ADON	32.50654802	120.281255	27.485409	Commercialized variety	Farmer owned field
182673	2018	Jiangsu	Wheat	*F. asiaticum*	3ADON	32.50654802	120.281255	27.485409	Commercialized variety	Farmer owned field
182672	2018	Jiangsu	Wheat	*F. asiaticum*	3ADON	32.50654802	120.281255	27.485409	Commercialized variety	Farmer owned field
182671	2018	Jiangsu	Wheat	*F. asiaticum*	3ADON	32.50654802	120.281255	27.485409	Commercialized variety	Farmer owned field
182670	2018	Jiangsu	Wheat	*F. asiaticum*	3ADON	32.50654802	120.281255	27.485409	Commercialized variety	Farmer owned field
182669	2018	Jiangsu	Wheat	*F. asiaticum*	3ADON	32.50654802	120.281255	27.485409	Commercialized variety	Farmer owned field
182668	2018	Jiangsu	Wheat	*F. asiaticum*	3ADON	32.50654802	120.281255	27.485409	Commercialized variety	Farmer owned field
182667	2018	Jiangsu	Wheat	*F. asiaticum*	3ADON	32.50654802	120.281255	27.485409	Commercialized variety	Farmer owned field
191837	2019	Jiangsu	Wheat	*F. asiaticum*	3ADON	32.53188903	118.714632	27.526869	Commercialized variety	Farmer owned field
191836	2019	Jiangsu	Wheat	*F. asiaticum*	3ADON	32.53188903	118.714632	27.526869	Commercialized variety	Farmer owned field
191835	2019	Jiangsu	Wheat	*F. asiaticum*	3ADON	32.53188903	118.714632	27.526869	Commercialized variety	Farmer owned field
191834	2019	Jiangsu	Wheat	*F. asiaticum*	3ADON	32.53188903	118.714632	27.526869	Commercialized variety	Farmer owned field
191833	2019	Jiangsu	Wheat	*F. asiaticum*	3ADON	32.53188903	118.714632	27.526869	Commercialized variety	Farmer owned field
191832	2019	Jiangsu	Wheat	*F. asiaticum*	3ADON	32.53188903	118.714632	27.526869	Commercialized variety	Farmer owned field
191831	2019	Jiangsu	Wheat	*F. asiaticum*	3ADON	32.53188903	118.714632	27.526869	Commercialized variety	Farmer owned field
182514	2018	Jiangsu	Wheat	*F. asiaticum*	3ADON	32.53188903	118.714632	27.526869	Commercialized variety	Farmer owned field
182513	2018	Jiangsu	Wheat	*F. asiaticum*	3ADON	32.53188903	118.714632	27.526869	Commercialized variety	Farmer owned field
182512	2018	Jiangsu	Wheat	*F. asiaticum*	3ADON	32.53188903	118.714632	27.526869	Commercialized variety	Farmer owned field

182511	2018	Jiangsu	Wheat	*F. asiaticum*	3ADON	32.53188903	118.714632	27.526869	Commercialized variety	Farmer owned field
182510	2018	Jiangsu	Wheat	*F. asiaticum*	3ADON	32.53188903	118.714632	27.526869	Commercialized variety	Farmer owned field
182509	2018	Jiangsu	Wheat	*F. asiaticum*	3ADON	32.53188903	118.714632	27.526869	Commercialized variety	Farmer owned field
182508	2018	Jiangsu	Wheat	*F. asiaticum*	3ADON	32.53188903	118.714632	27.526869	Commercialized variety	Farmer owned field
182507	2018	Jiangsu	Wheat	*F. asiaticum*	3ADON	32.53188903	118.714632	27.526869	Commercialized variety	Farmer owned field
182506	2018	Jiangsu	Wheat	*F. asiaticum*	NIV	32.53188903	118.714632	27.526869	Commercialized variety	Farmer owned field
182505	2018	Jiangsu	Wheat	*F. asiaticum*	3ADON	32.53188903	118.714632	27.526869	Commercialized variety	Farmer owned field
182504	2018	Jiangsu	Wheat	*F. asiaticum*	3ADON	32.53188903	118.714632	27.526869	Commercialized variety	Farmer owned field
182503	2018	Jiangsu	Wheat	*F. asiaticum*	3ADON	32.53188903	118.714632	27.526869	Commercialized variety	Farmer owned field
182502	2018	Jiangsu	Wheat	*F. asiaticum*	3ADON	32.53188903	118.714632	27.526869	Commercialized variety	Farmer owned field
182501	2018	Jiangsu	Wheat	*F. asiaticum*	3ADON	32.53188903	118.714632	27.526869	Commercialized variety	Farmer owned field
JSNTRG018	2014	Jiangsu	Rice	*F. asiaticum*	3ADON	32.540837	120.466991	5		Farmer owned field
JSNTRG017	2014	Jiangsu	Rice	*F. asiaticum*	3ADON	32.540837	120.466991	5		Farmer owned field
JSNTRG016	2014	Jiangsu	Rice	*F. asiaticum*	3ADON	32.540837	120.466991	5		Farmer owned field
JSNTRG015	2014	Jiangsu	Rice	*F. asiaticum*	NIV	32.540837	120.466991	5		Farmer owned field
JSNTRG012	2014	Jiangsu	Rice	*F. asiaticum*	3ADON	32.540837	120.466991	5		Farmer owned field
JSNTRG011	2014	Jiangsu	Rice	*F. asiaticum*	3ADON	32.540837	120.466991	5		Farmer owned field
JSNTRG010	2014	Jiangsu	Rice	*F. asiaticum*	3ADON	32.540837	120.466991	5		Farmer owned field
JSNTRG009	2014	Jiangsu	Rice	*F. asiaticum*	3ADON	32.540837	120.466991	5		Farmer owned field
JSNTRG008	2014	Jiangsu	Rice	*F. asiaticum*	NIV	32.540837	120.466991	5		Farmer owned field
JSNTRG006	2014	Jiangsu	Rice	*F. asiaticum*	3ADON	32.540837	120.466991	5		Farmer owned field
JSNTRG005	2014	Jiangsu	Rice	*F. asiaticum*	3ADON	32.540837	120.466991	5		Farmer owned field
JSNTRG004	2014	Jiangsu	Rice	*F. asiaticum*	NIV	32.540837	120.466991	5		Farmer owned field
JSNTRG003	2014	Jiangsu	Rice	*F. asiaticum*	3ADON	32.540837	120.466991	5		Farmer owned field
JSNTRG002	2014	Jiangsu	Rice	*F. asiaticum*	3ADON	32.540837	120.466991	5		Farmer owned field
JSNTHA018	2014	Jiangsu	Rice	*F. asiaticum*	3ADON	32.540837	120.466991	5		Farmer owned field
JSNTHA017	2014	Jiangsu	Rice	*F. asiaticum*	3ADON	32.540837	120.466991	5		Farmer owned field
JSNTHA015	2014	Jiangsu	Rice	*F. asiaticum*	3ADON	32.540837	120.466991	5		Farmer owned field
JSNTHA014	2014	Jiangsu	Rice	*F. asiaticum*	3ADON	32.540837	120.466991	5		Farmer owned field
JSNTHA013	2014	Jiangsu	Rice	*F. asiaticum*	NIV	32.540837	120.466991	5		Farmer owned field
JSNTHA011	2014	Jiangsu	Rice	*F. asiaticum*	3ADON	32.540837	120.466991	5		Farmer owned field
JSNTHA010	2014	Jiangsu	Rice	*F. asiaticum*	3ADON	32.540837	120.466991	5		Farmer owned field
JSNTHA009	2014	Jiangsu	Rice	*F. asiaticum*	NIV	32.540837	120.466991	5		Farmer owned field
JSNTHA008	2014	Jiangsu	Rice	*F. asiaticum*	3ADON	32.540837	120.466991	5		Farmer owned field
JSNTHA007	2014	Jiangsu	Rice	*F. asiaticum*	NIV	32.540837	120.466991	5		Farmer owned field

JSNTHA006	2014	Jiangsu	Rice	*F. asiaticum*	3ADON	32.540837	120.466991	5		Farmer owned field
JSNTHA005	2014	Jiangsu	Rice	*F. asiaticum*	NIV	32.540837	120.466991	5		Farmer owned field
JSNTHA004	2014	Jiangsu	Rice	*F. asiaticum*	3ADON	32.540837	120.466991	5		Farmer owned field
JSNTHA003	2014	Jiangsu	Rice	*F. asiaticum*	3ADON	32.540837	120.466991	5		Farmer owned field
JSNTHA002	2014	Jiangsu	Rice	*F. asiaticum*	3ADON	32.540837	120.466991	5		Farmer owned field
192874	2019	Jiangsu	Wheat	*F. asiaticum*	3ADON	32.65985898	120.517313	15.445559	Commercialized variety	Farmer owned field
192873	2019	Jiangsu	Wheat	*F. asiaticum*	3ADON	32.65985898	120.517313	15.445559	Commercialized variety	Farmer owned field
192871	2019	Jiangsu	Wheat	*F. asiaticum*	3ADON	32.65985898	120.517313	15.445559	Commercialized variety	Farmer owned field
192870	2019	Jiangsu	Wheat	*F. asiaticum*	3ADON	32.65985898	120.517313	15.445559	Commercialized variety	Farmer owned field
192967	2019	Jiangsu	Wheat	*F. asiaticum*	3ADON	32.82019104	118.740714	57.389439	Commercialized variety	Farmer owned field
192965	2019	Jiangsu	Wheat	*F. asiaticum*	3ADON	32.82019104	118.740714	57.389439	Commercialized variety	Farmer owned field
192958	2019	Jiangsu	Wheat	*F. asiaticum*	3ADON	32.82019104	118.740714	57.389439	Commercialized variety	Farmer owned field
192957	2019	Jiangsu	Wheat	*F. asiaticum*	3ADON	32.82019104	118.740714	57.389439	Commercialized variety	Farmer owned field
192956	2019	Jiangsu	Wheat	*F. asiaticum*	3ADON	32.82019104	118.740714	57.389439	Commercialized variety	Farmer owned field
192969	2019	Jiangsu	Wheat	*F. asiaticum*	3ADON	33.09202203	118.413505	57.896366	Commercialized variety	Farmer owned field
192968	2019	Jiangsu	Wheat	*F. asiaticum*	3ADON	33.09202203	118.413505	57.896366	Commercialized variety	Farmer owned field
182630	2018	Jiangsu	Wheat	*F. asiaticum*	3ADON	33.12189102	118.343823	47.342438	Commercialized variety	Farmer owned field
182629	2018	Jiangsu	Wheat	*F. asiaticum*	3ADON	33.12189102	118.343823	47.342438	Commercialized variety	Farmer owned field
182628	2018	Jiangsu	Wheat	*F. asiaticum*	3ADON	33.12189102	118.343823	47.342438	Commercialized variety	Farmer owned field
182627	2018	Jiangsu	Wheat	*F. asiaticum*	3ADON	33.12189102	118.343823	47.342438	Commercialized variety	Farmer owned field
182626	2018	Jiangsu	Wheat	*F. asiaticum*	3ADON	33.12189102	118.343823	47.342438	Commercialized variety	Farmer owned field
182625	2018	Jiangsu	Wheat	*F. asiaticum*	NIV	33.12189102	118.343823	47.342438	Commercialized variety	Farmer owned field
182624	2018	Jiangsu	Wheat	*F. asiaticum*	NIV	33.12189102	118.343823	47.342438	Commercialized variety	Farmer owned field
182623	2018	Jiangsu	Wheat	*F. asiaticum*	NIV	33.12189102	118.343823	47.342438	Commercialized variety	Farmer owned field
182622	2018	Jiangsu	Wheat	*F. asiaticum*	3ADON	33.12189102	118.343823	47.342438	Commercialized variety	Farmer owned field
182620	2018	Jiangsu	Wheat	*F. asiaticum*	3ADON	33.12189102	118.343823	47.342438	Commercialized variety	Farmer owned field
182619	2018	Jiangsu	Wheat	*F. asiaticum*	NIV	33.12189102	118.343823	47.342438	Commercialized variety	Farmer owned field
182618	2018	Jiangsu	Wheat	*F. asiaticum*	3ADON	33.12189102	118.343823	47.342438	Commercialized variety	Farmer owned field
182617	2018	Jiangsu	Wheat	*F. asiaticum*	3ADON	33.12189102	118.343823	47.342438	Commercialized variety	Farmer owned field
182616	2018	Jiangsu	Wheat	*F. asiaticum*	3ADON	33.12189102	118.343823	47.342438	Commercialized variety	Farmer owned field
182615	2018	Jiangsu	Wheat	*F. asiaticum*	3ADON	33.12189102	118.343823	47.342438	Commercialized variety	Farmer owned field
182614	2018	Jiangsu	Wheat	*F. asiaticum*	NIV	33.12189102	118.343823	47.342438	Commercialized variety	Farmer owned field
192946	2019	Jiangsu	Wheat	*F. asiaticum*	3ADON	33.15891598	120.38929	5.723974	Commercialized variety	Farmer owned field
192945	2019	Jiangsu	Wheat	*F. asiaticum*	3ADON	33.15891598	120.38929	5.723974	Commercialized variety	Farmer owned field
182666	2018	Jiangsu	Wheat	*F. asiaticum*	3ADON	33.15891598	120.38929	5.723974	Commercialized variety	Farmer owned field

182665	2018	Jiangsu	Wheat	*F. asiaticum*	3ADON	33.15891598	120.38929	5.723974	Commercialized variety	Farmer owned field
182664	2018	Jiangsu	Wheat	*F. asiaticum*	3ADON	33.15891598	120.38929	5.723974	Commercialized variety	Farmer owned field
182662	2018	Jiangsu	Wheat	*F. asiaticum*	3ADON	33.15891598	120.38929	5.723974	Commercialized variety	Farmer owned field
AHFT14003	2014	Anhui	Rice	*F. asiaticum*	3ADON	32.77738333	116.6282333	26		Commercial farm
AHFT14001	2014	Anhui	Rice	*F. asiaticum*	3ADON	32.77738333	116.6282333	26		Commercial farm
AHFT020	2014	Anhui	Rice	*F. asiaticum*	3ADON	32.77738333	116.6282333	26		Commercial farm
AHFT019	2014	Anhui	Rice	*F. asiaticum*	NIV	32.77738333	116.6282333	26		Commercial farm
AHFT012	2014	Anhui	Rice	*F. asiaticum*	3ADON	32.77738333	116.6282333	26		Commercial farm
AHFT008	2014	Anhui	Rice	*F. asiaticum*	NIV	32.77738333	116.6282333	26		Commercial farm
191533	2019	Anhui	Wheat	*F. asiaticum*	3ADON	32.78324604	115.686363	30.566353	Commercialized variety	Farmer owned field
191532	2019	Anhui	Wheat	*F. asiaticum*	3ADON	32.78324604	115.686363	30.566353	Commercialized variety	Farmer owned field
191531	2019	Anhui	Wheat	*F. asiaticum*	3ADON	32.78324604	115.686363	30.566353	Commercialized variety	Farmer owned field
191530	2019	Anhui	Wheat	*F. asiaticum*	15ADON	32.78324604	115.686363	30.566353	Commercialized variety	Farmer owned field
191529	2019	Anhui	Wheat	*F. asiaticum*	NIV	32.78324604	115.686363	30.566353	Commercialized variety	Farmer owned field
191528	2019	Anhui	Wheat	*F. asiaticum*	3ADON	32.78324604	115.686363	30.566353	Commercialized variety	Farmer owned field
191526	2019	Anhui	Wheat	*F. asiaticum*	3ADON	32.78324604	115.686363	30.566353	Commercialized variety	Farmer owned field
191524	2019	Anhui	Wheat	*F. asiaticum*	15ADON	32.78324604	115.686363	30.566353	Commercialized variety	Farmer owned field
191522	2019	Anhui	Wheat	*F. asiaticum*	15ADON	32.78324604	115.686363	30.566353	Commercialized variety	Farmer owned field
191521	2019	Anhui	Wheat	*F. asiaticum*	15ADON	32.78324604	115.686363	30.566353	Commercialized variety	Farmer owned field
191520	2019	Anhui	Wheat	*F. asiaticum*	3ADON	32.78324604	115.686363	30.566353	Commercialized variety	Farmer owned field
182661	2018	Jiangsu	Wheat	*F. asiaticum*	3ADON	33.15891598	120.38929	5.723974	Commercialized variety	Farmer owned field
191291	2019	Jiangsu	Wheat	*F. asiaticum*	3ADON	33.26554502	118.883774	18.811953	Commercialized variety	Farmer owned field
191290	2019	Jiangsu	Wheat	*F. asiaticum*	3ADON	33.26554502	118.883774	18.811953	Commercialized variety	Farmer owned field
191289	2019	Jiangsu	Wheat	*F. asiaticum*	3ADON	33.26554502	118.883774	18.811953	Commercialized variety	Farmer owned field
191288	2019	Jiangsu	Wheat	*F. asiaticum*	NIV	33.26554502	118.883774	18.811953	Commercialized variety	Farmer owned field
191287	2019	Jiangsu	Wheat	*F. asiaticum*	3ADON	33.26554502	118.883774	18.811953	Commercialized variety	Farmer owned field
191286	2019	Jiangsu	Wheat	*F. asiaticum*	3ADON	33.26554502	118.883774	18.811953	Commercialized variety	Farmer owned field
191285	2019	Jiangsu	Wheat	*F. asiaticum*	3ADON	33.26554502	118.883774	18.811953	Commercialized variety	Farmer owned field
191284	2019	Jiangsu	Wheat	*F. asiaticum*	3ADON	33.26554502	118.883774	18.811953	Commercialized variety	Farmer owned field
191282	2019	Jiangsu	Wheat	*F. asiaticum*	3ADON	33.26554502	118.883774	18.811953	Commercialized variety	Farmer owned field
191928	2019	Hubei	Wheat	*F. asiaticum*	3ADON	30.43971403	113.753649	23.424345	Commercialized variety	Farmer owned field
191927	2019	Hubei	Wheat	*F. asiaticum*	3ADON	30.43971403	113.753649	23.424345	Commercialized variety	Farmer owned field
191926	2019	Hubei	Wheat	*F. asiaticum*	3ADON	30.43971403	113.753649	23.424345	Commercialized variety	Farmer owned field
191925	2019	Hubei	Wheat	*F. asiaticum*	3ADON	30.43971403	113.753649	23.424345	Commercialized variety	Farmer owned field
191924	2019	Hubei	Wheat	*F. asiaticum*	3ADON	30.43971403	113.753649	23.424345	Commercialized variety	Farmer owned field

191923	2019	Hubei	Wheat	*F. asiaticum*	3ADON	30.43971403	113.753649	23.424345	Commercialized variety	Farmer owned field
191922	2019	Hubei	Wheat	*F. asiaticum*	3ADON	30.43971403	113.753649	23.424345	Commercialized variety	Farmer owned field
191921	2019	Hubei	Wheat	*F. asiaticum*	15ADON	30.43971403	113.753649	23.424345	Commercialized variety	Farmer owned field
191920	2019	Hubei	Wheat	*F. asiaticum*	3ADON	30.43971403	113.753649	23.424345	Commercialized variety	Farmer owned field
191917	2019	Hubei	Wheat	*F. asiaticum*	NIV	30.43971403	113.753649	23.424345	Commercialized variety	Farmer owned field
191916	2019	Hubei	Wheat	*F. asiaticum*	3ADON	30.43971403	113.753649	23.424345	Commercialized variety	Farmer owned field
191862	2019	Hubei	Wheat	*F. asiaticum*	3ADON	30.47595503	114.773393	5.96834	Commercialized variety	Farmer owned field
191860	2019	Hubei	Wheat	*F. asiaticum*	3ADON	30.47595503	114.773393	5.96834	Commercialized variety	Farmer owned field
191859	2019	Hubei	Wheat	*F. asiaticum*	3ADON	30.47595503	114.773393	5.96834	Commercialized variety	Farmer owned field
191858	2019	Hubei	Wheat	*F. asiaticum*	3ADON	30.47595503	114.773393	5.96834	Commercialized variety	Farmer owned field
191857	2019	Hubei	Wheat	*F. asiaticum*	3ADON	30.47595503	114.773393	5.96834	Commercialized variety	Farmer owned field
191856	2019	Hubei	Wheat	*F. asiaticum*	3ADON	30.47595503	114.773393	5.96834	Commercialized variety	Farmer owned field
191855	2019	Hubei	Wheat	*F. asiaticum*	3ADON	30.47595503	114.773393	5.96834	Commercialized variety	Farmer owned field
191854	2019	Hubei	Wheat	*F. asiaticum*	3ADON	30.47595503	114.773393	5.96834	Commercialized variety	Farmer owned field
191853	2019	Hubei	Wheat	*F. asiaticum*	3ADON	30.47595503	114.773393	5.96834	Commercialized variety	Farmer owned field
191852	2019	Hubei	Wheat	*F. asiaticum*	3ADON	30.47595503	114.773393	5.96834	Commercialized variety	Farmer owned field
191851	2019	Hubei	Wheat	*F. asiaticum*	3ADON	30.47595503	114.773393	5.96834	Commercialized variety	Farmer owned field
191848	2019	Hubei	Wheat	*F. asiaticum*	3ADON	30.47595503	114.773393	5.96834	Commercialized variety	Farmer owned field
181037	2018	Hubei	Wheat	*F. asiaticum*	3ADON	30.56438897	114.917469	24.37772	Commercialized variety	Commercial farm
181036	2018	Hubei	Wheat	*F. asiaticum*	NIV	30.56438897	114.917469	24.37772	Commercialized variety	Commercial farm
181034	2018	Hubei	Wheat	*F. asiaticum*	NIV	30.56438897	114.917469	24.37772	Commercialized variety	Commercial farm
181033	2018	Hubei	Wheat	*F. asiaticum*	3ADON	30.56438897	114.917469	24.37772	Commercialized variety	Commercial farm
181032	2018	Hubei	Wheat	*F. asiaticum*	3ADON	30.56438897	114.917469	24.37772	Commercialized variety	Commercial farm
181031	2018	Hubei	Wheat	*F. asiaticum*	3ADON	30.56438897	114.917469	24.37772	Commercialized variety	Commercial farm
181030	2018	Hubei	Wheat	*F. asiaticum*	3ADON	30.56438897	114.917469	24.37772	Commercialized variety	Commercial farm
181029	2018	Hubei	Wheat	*F. asiaticum*	3ADON	30.56438897	114.917469	24.37772	Commercialized variety	Commercial farm
181028	2018	Hubei	Wheat	*F. asiaticum*	NIV	30.56438897	114.917469	24.37772	Commercialized variety	Commercial farm
181027	2018	Hubei	Wheat	*F. asiaticum*	3ADON	30.56438897	114.917469	24.37772	Commercialized variety	Commercial farm
181026	2018	Hubei	Wheat	*F. asiaticum*	3ADON	30.56438897	114.917469	24.37772	Commercialized variety	Commercial farm
181025	2018	Hubei	Wheat	*F. asiaticum*	15ADON	30.56438897	114.917469	24.37772	Commercialized variety	Commercial farm
181024	2018	Hubei	Wheat	*F. asiaticum*	NIV	30.56438897	114.917469	24.37772	Commercialized variety	Commercial farm
181023	2018	Hubei	Wheat	*F. asiaticum*	3ADON	30.56438897	114.917469	24.37772	Commercialized variety	Commercial farm
181022	2018	Hubei	Wheat	*F. asiaticum*	3ADON	30.56438897	114.917469	24.37772	Commercialized variety	Commercial farm
181021	2018	Hubei	Wheat	*F. asiaticum*	3ADON	30.56438897	114.917469	24.37772	Commercialized variety	Commercial farm
181020	2018	Hubei	Wheat	*F. asiaticum*	3ADON	30.56438897	114.917469	24.37772	Commercialized variety	Commercial farm

181019	2018	Hubei	Wheat	*F. asiaticum*	3ADON	30.56438897	114.917469	24.37772	Commercialized variety	Commercial farm
181018	2018	Hubei	Wheat	*F. asiaticum*	3ADON	30.56438897	114.917469	24.37772	Commercialized variety	Commercial farm
181017	2018	Hubei	Wheat	*F. asiaticum*	3ADON	30.56438897	114.917469	24.37772	Commercialized variety	Commercial farm
180308	2018	Hubei	Rice	*F. asiaticum*	3ADON	30.56438897	114.917469	24.37772		Commercial farm
180307	2018	Hubei	Rice	*F. asiaticum*	3ADON	30.56438897	114.917469	24.37772		Commercial farm
180306	2018	Hubei	Rice	*F. asiaticum*	3ADON	30.56438897	114.917469	24.37772		Commercial farm
180305	2018	Hubei	Rice	*F. asiaticum*	3ADON	30.56438897	114.917469	24.37772		Commercial farm
180304	2018	Hubei	Rice	*F. asiaticum*	3ADON	30.56438897	114.917469	24.37772		Commercial farm
180303	2018	Hubei	Rice	*F. asiaticum*	NIV	30.56438897	114.917469	24.37772		Commercial farm
180302	2018	Hubei	Rice	*F. asiaticum*	NIV	30.56438897	114.917469	24.37772		Commercial farm
180301	2018	Hubei	Rice	*F. asiaticum*	NIV	30.56438897	114.917469	24.37772		Commercial farm
180300	2018	Hubei	Rice	*F. asiaticum*	3ADON	30.56438897	114.917469	24.37772		Commercial farm
180299	2018	Hubei	Rice	*F. asiaticum*	3ADON	30.56438897	114.917469	24.37772		Commercial farm
180298	2018	Hubei	Rice	*F. asiaticum*	3ADON	30.56438897	114.917469	24.37772		Commercial farm
180297	2018	Hubei	Rice	*F. asiaticum*	3ADON	30.56438897	114.917469	24.37772		Commercial farm
180296	2018	Hubei	Rice	*F. asiaticum*	NIV	30.56438897	114.917469	24.37772		Commercial farm
180295	2018	Hubei	Rice	*F. asiaticum*	NIV	30.56438897	114.917469	24.37772		Commercial farm
180294	2018	Hubei	Rice	*F. asiaticum*	NIV	30.56438897	114.917469	24.37772		Commercial farm
180293	2018	Hubei	Rice	*F. asiaticum*	3ADON	30.56438897	114.917469	24.37772		Commercial farm
180292	2018	Hubei	Rice	*F. asiaticum*	3ADON	30.56438897	114.917469	24.37772		Commercial farm
180291	2018	Hubei	Rice	*F. asiaticum*	3ADON	30.56438897	114.917469	24.37772		Commercial farm
180290	2018	Hubei	Rice	*F. asiaticum*	NIV	30.56438897	114.917469	24.37772		Commercial farm
180289	2018	Hubei	Rice	*F. asiaticum*	NIV	30.56438897	114.917469	24.37772		Commercial farm
180288	2018	Hubei	Rice	*F. asiaticum*	3ADON	30.56438897	114.917469	24.37772		Commercial farm
180287	2018	Hubei	Rice	*F. asiaticum*	NIV	30.56438897	114.917469	24.37772		Commercial farm
180286	2018	Hubei	Rice	*F. asiaticum*	NIV	30.56438897	114.917469	24.37772		Commercial farm
180285	2018	Hubei	Rice	*F. asiaticum*	NIV	30.56438897	114.917469	24.37772		Commercial farm
180284	2018	Hubei	Rice	*F. asiaticum*	3ADON	30.56438897	114.917469	24.37772		Commercial farm
183500	2018	Hubei	Wheat	*F. asiaticum*	15ADON	30.582745	112.581635	NA	Commercialized variety	Farmer owned field
183499	2018	Hubei	Wheat	*F. asiaticum*	15ADON	30.582745	112.581635	NA	Commercialized variety	Farmer owned field
183498	2018	Hubei	Wheat	*F. asiaticum*	15ADON	30.582745	112.581635	NA	Commercialized variety	Farmer owned field
181123	2018	Hubei	Wheat	*F. asiaticum*	3ADON	30.59299598	113.137023	27.07382	Commercialized variety	Farmer owned field
181122	2018	Hubei	Wheat	*F. asiaticum*	3ADON	30.59299598	113.137023	27.07382	Commercialized variety	Farmer owned field
181121	2018	Hubei	Wheat	*F. asiaticum*	3ADON	30.59299598	113.137023	27.07382	Commercialized variety	Farmer owned field
181120	2018	Hubei	Wheat	*F. asiaticum*	3ADON	30.59299598	113.137023	27.07382	Commercialized variety	Farmer owned field

181119	2018	Hubei	Wheat	*F. asiaticum*	3ADON	30.59299598	113.137023	27.07382	Commercialized variety	Farmer owned field
181118	2018	Hubei	Wheat	*F. asiaticum*	3ADON	30.59299598	113.137023	27.07382	Commercialized variety	Farmer owned field
181117	2018	Hubei	Wheat	*F. asiaticum*	3ADON	30.59299598	113.137023	27.07382	Commercialized variety	Farmer owned field
181116	2018	Hubei	Wheat	*F. asiaticum*	3ADON	30.59299598	113.137023	27.07382	Commercialized variety	Farmer owned field
181115	2018	Hubei	Wheat	*F. asiaticum*	3ADON	30.59299598	113.137023	27.07382	Commercialized variety	Farmer owned field
181114	2018	Hubei	Wheat	*F. asiaticum*	3ADON	30.59299598	113.137023	27.07382	Commercialized variety	Farmer owned field
181113	2018	Hubei	Wheat	*F. asiaticum*	NIV	30.59299598	113.137023	27.07382	Commercialized variety	Farmer owned field
181112	2018	Hubei	Wheat	*F. asiaticum*	3ADON	30.59299598	113.137023	27.07382	Commercialized variety	Farmer owned field
181062	2018	Hubei	Wheat	*F. asiaticum*	3ADON	30.63488597	113.115741	31.271826	Commercialized variety	Farmer owned field
181061	2018	Hubei	Wheat	*F. asiaticum*	3ADON	30.63488597	113.115741	31.271826	Commercialized variety	Farmer owned field
181060	2018	Hubei	Wheat	*F. asiaticum*	3ADON	30.63488597	113.115741	31.271826	Commercialized variety	Farmer owned field
181059	2018	Hubei	Wheat	*F. asiaticum*	3ADON	30.63488597	113.115741	31.271826	Commercialized variety	Farmer owned field
181058	2018	Hubei	Wheat	*F. asiaticum*	15ADON	30.63488597	113.115741	31.271826	Commercialized variety	Farmer owned field
181057	2018	Hubei	Wheat	*F. asiaticum*	3ADON	30.63488597	113.115741	31.271826	Commercialized variety	Farmer owned field
181056	2018	Hubei	Wheat	*F. asiaticum*	3ADON	30.63488597	113.115741	31.271826	Commercialized variety	Farmer owned field
181055	2018	Hubei	Wheat	*F. asiaticum*	3ADON	30.63488597	113.115741	31.271826	Commercialized variety	Farmer owned field
181054	2018	Hubei	Wheat	*F. asiaticum*	15ADON	30.63488597	113.115741	31.271826	Commercialized variety	Farmer owned field
181053	2018	Hubei	Wheat	*F. asiaticum*	3ADON	30.63488597	113.115741	31.271826	Commercialized variety	Farmer owned field
181052	2018	Hubei	Wheat	*F. asiaticum*	3ADON	30.63488597	113.115741	31.271826	Commercialized variety	Farmer owned field
181051	2018	Hubei	Wheat	*F. asiaticum*	3ADON	30.63488597	113.115741	31.271826	Commercialized variety	Farmer owned field
181049	2018	Hubei	Wheat	*F. asiaticum*	3ADON	30.63488597	113.115741	31.271826	Commercialized variety	Farmer owned field
181048	2018	Hubei	Wheat	*F. asiaticum*	3ADON	30.63488597	113.115741	31.271826	Commercialized variety	Farmer owned field
181047	2018	Hubei	Wheat	*F. asiaticum*	3ADON	30.63488597	113.115741	31.271826	Commercialized variety	Farmer owned field
181046	2018	Hubei	Wheat	*F. asiaticum*	15ADON	30.63488597	113.115741	31.271826	Commercialized variety	Farmer owned field
181045	2018	Hubei	Wheat	*F. asiaticum*	3ADON	30.63488597	113.115741	31.271826	Commercialized variety	Farmer owned field
181044	2018	Hubei	Wheat	*F. asiaticum*	3ADON	30.63488597	113.115741	31.271826	Commercialized variety	Farmer owned field
181043	2018	Hubei	Wheat	*F. asiaticum*	NIV	30.63488597	113.115741	31.271826	Commercialized variety	Farmer owned field
181042	2018	Hubei	Wheat	*F. asiaticum*	3ADON	30.63488597	113.115741	31.271826	Commercialized variety	Farmer owned field
181041	2018	Hubei	Wheat	*F. asiaticum*	3ADON	30.63488597	113.115741	31.271826	Commercialized variety	Farmer owned field
181040	2018	Hubei	Wheat	*F. asiaticum*	15ADON	30.63488597	113.115741	31.271826	Commercialized variety	Farmer owned field
181039	2018	Hubei	Wheat	*F. asiaticum*	3ADON	30.63488597	113.115741	31.271826	Commercialized variety	Farmer owned field
181038	2018	Hubei	Wheat	*F. asiaticum*	3ADON	30.63488597	113.115741	31.271826	Commercialized variety	Farmer owned field
180637	2018	Hubei	Wheat	*F. asiaticum*	NIV	30.63488597	113.115741	31.271826	Commercialized variety	Farmer owned field
180636	2018	Hubei	Wheat	*F. asiaticum*	3ADON	30.63488597	113.115741	31.271826	Commercialized variety	Farmer owned field
180635	2018	Hubei	Wheat	*F. asiaticum*	NIV	30.63488597	113.115741	31.271826	Commercialized variety	Farmer owned field

180634	2018	Hubei	Wheat	*F. asiaticum*	NIV	30.63488597	113.115741	31.271826	Commercialized variety	Farmer owned field
180633	2018	Hubei	Wheat	*F. asiaticum*	NIV	30.63488597	113.115741	31.271826	Commercialized variety	Farmer owned field
180632	2018	Hubei	Wheat	*F. asiaticum*	3ADON	30.63488597	113.115741	31.271826	Commercialized variety	Farmer owned field
180631	2018	Hubei	Wheat	*F. asiaticum*	3ADON	30.63488597	113.115741	31.271826	Commercialized variety	Farmer owned field
180630	2018	Hubei	Wheat	*F. asiaticum*	3ADON	30.63488597	113.115741	31.271826	Commercialized variety	Farmer owned field
180629	2018	Hubei	Wheat	*F. asiaticum*	3ADON	30.63488597	113.115741	31.271826	Commercialized variety	Farmer owned field
180628	2018	Hubei	Wheat	*F. asiaticum*	NIV	30.63488597	113.115741	31.271826	Commercialized variety	Farmer owned field
180627	2018	Hubei	Wheat	*F. asiaticum*	3ADON	30.63488597	113.115741	31.271826	Commercialized variety	Farmer owned field
180626	2018	Hubei	Wheat	*F. asiaticum*	NIV	30.63488597	113.115741	31.271826	Commercialized variety	Farmer owned field
191958	2019	Hubei	Wheat	*F. asiaticum*	3ADON	30.80450099	112.184152	151.904312	Commercialized variety	Farmer owned field
191957	2019	Hubei	Wheat	*F. asiaticum*	3ADON	30.80450099	112.184152	151.904312	Commercialized variety	Farmer owned field
191956	2019	Hubei	Wheat	*F. asiaticum*	3ADON	30.80450099	112.184152	151.904312	Commercialized variety	Farmer owned field
191955	2019	Hubei	Wheat	*F. asiaticum*	3ADON	30.80450099	112.184152	151.904312	Commercialized variety	Farmer owned field
191954	2019	Hubei	Wheat	*F. asiaticum*	3ADON	30.80450099	112.184152	151.904312	Commercialized variety	Farmer owned field
191953	2019	Hubei	Wheat	*F. asiaticum*	3ADON	30.80450099	112.184152	151.904312	Commercialized variety	Farmer owned field
191952	2019	Hubei	Wheat	*F. asiaticum*	3ADON	30.80450099	112.184152	151.904312	Commercialized variety	Farmer owned field
191951	2019	Hubei	Wheat	*F. asiaticum*	15ADON	30.80450099	112.184152	151.904312	Commercialized variety	Farmer owned field
191950	2019	Hubei	Wheat	*F. asiaticum*	3ADON	30.80450099	112.184152	151.904312	Commercialized variety	Farmer owned field
191949	2019	Hubei	Wheat	*F. asiaticum*	3ADON	30.80450099	112.184152	151.904312	Commercialized variety	Farmer owned field
191948	2019	Hubei	Wheat	*F. asiaticum*	15ADON	30.80450099	112.184152	151.904312	Commercialized variety	Farmer owned field
191947	2019	Hubei	Wheat	*F. asiaticum*	3ADON	30.80450099	112.184152	151.904312	Commercialized variety	Farmer owned field
191946	2019	Hubei	Wheat	*F. asiaticum*	3ADON	30.80450099	112.184152	151.904312	Commercialized variety	Farmer owned field
191945	2019	Hubei	Wheat	*F. asiaticum*	3ADON	30.80450099	112.184152	151.904312	Commercialized variety	Farmer owned field
191944	2019	Hubei	Wheat	*F. asiaticum*	15ADON	30.80450099	112.184152	151.904312	Commercialized variety	Farmer owned field
191943	2019	Hubei	Wheat	*F. asiaticum*	3ADON	30.80450099	112.184152	151.904312	Commercialized variety	Farmer owned field
191942	2019	Hubei	Wheat	*F. asiaticum*	3ADON	30.80450099	112.184152	151.904312	Commercialized variety	Farmer owned field
191941	2019	Hubei	Wheat	*F. asiaticum*	3ADON	30.80450099	112.184152	151.904312	Commercialized variety	Farmer owned field
191940	2019	Hubei	Wheat	*F. asiaticum*	15ADON	30.80450099	112.184152	151.904312	Commercialized variety	Farmer owned field
191939	2019	Hubei	Wheat	*F. asiaticum*	3ADON	30.80450099	112.184152	151.904312	Commercialized variety	Farmer owned field
181097	2018	Hubei	Wheat	*F. asiaticum*	3ADON	30.86147396	112.867559	68.377945	Commercialized variety	Commercial farm
181096	2018	Hubei	Wheat	*F. asiaticum*	NIV	30.86147396	112.867559	68.377945	Commercialized variety	Commercial farm
181095	2018	Hubei	Wheat	*F. asiaticum*	NIV	30.86147396	112.867559	68.377945	Commercialized variety	Commercial farm
181094	2018	Hubei	Wheat	*F. asiaticum*	3ADON	30.86147396	112.867559	68.377945	Commercialized variety	Commercial farm
181093	2018	Hubei	Wheat	*F. asiaticum*	3ADON	30.86147396	112.867559	68.377945	Commercialized variety	Commercial farm
181092	2018	Hubei	Wheat	*F. asiaticum*	3ADON	30.86147396	112.867559	68.377945	Commercialized variety	Commercial farm

181091	2018	Hubei	Wheat	*F. asiaticum*	3ADON	30.86147396	112.867559	68.377945	Commercialized variety	Commercial farm
181090	2018	Hubei	Wheat	*F. asiaticum*	3ADON	30.86147396	112.867559	68.377945	Commercialized variety	Commercial farm
181089	2018	Hubei	Wheat	*F. asiaticum*	3ADON	30.86147396	112.867559	68.377945	Commercialized variety	Commercial farm
181088	2018	Hubei	Wheat	*F. asiaticum*	3ADON	30.86147396	112.867559	68.377945	Commercialized variety	Commercial farm
181087	2018	Hubei	Wheat	*F. asiaticum*	3ADON	30.86147396	112.867559	68.377945	Commercialized variety	Commercial farm
181086	2018	Hubei	Wheat	*F. asiaticum*	3ADON	30.86147396	112.867559	68.377945	Commercialized variety	Commercial farm
181085	2018	Hubei	Wheat	*F. asiaticum*	3ADON	30.86147396	112.867559	68.377945	Commercialized variety	Commercial farm
181084	2018	Hubei	Wheat	*F. asiaticum*	NIV	30.86147396	112.867559	68.377945	Commercialized variety	Commercial farm
181083	2018	Hubei	Wheat	*F. asiaticum*	3ADON	30.86147396	112.867559	68.377945	Commercialized variety	Commercial farm
181082	2018	Hubei	Wheat	*F. asiaticum*	NIV	30.86147396	112.867559	68.377945	Commercialized variety	Commercial farm
181081	2018	Hubei	Wheat	*F. asiaticum*	3ADON	30.86147396	112.867559	68.377945	Commercialized variety	Commercial farm
181080	2018	Hubei	Wheat	*F. asiaticum*	3ADON	30.86147396	112.867559	68.377945	Commercialized variety	Commercial farm
181079	2018	Hubei	Wheat	*F. asiaticum*	15ADON	30.86147396	112.867559	68.377945	Commercialized variety	Commercial farm
181078	2018	Hubei	Wheat	*F. asiaticum*	15ADON	30.86147396	112.867559	68.377945	Commercialized variety	Commercial farm
181077	2018	Hubei	Wheat	*F. asiaticum*	3ADON	30.86147396	112.867559	68.377945	Commercialized variety	Commercial farm
181076	2018	Hubei	Wheat	*F. asiaticum*	NIV	30.86147396	112.867559	68.377945	Commercialized variety	Commercial farm
180584	2018	Hubei	Rice	*F. asiaticum*	3ADON	30.86147396	112.867559	68.377945		Commercial farm
180583	2018	Hubei	Rice	*F. asiaticum*	3ADON	30.86147396	112.867559	68.377945		Commercial farm
180582	2018	Hubei	Rice	*F. asiaticum*	NIV	30.86147396	112.867559	68.377945		Commercial farm
180581	2018	Hubei	Rice	*F. asiaticum*	NIV	30.86147396	112.867559	68.377945		Commercial farm
180580	2018	Hubei	Rice	*F. asiaticum*	3ADON	30.86147396	112.867559	68.377945		Commercial farm
SCJY2054	2014	Sichuan	Rice	*F. asiaticum*	NIV	30.446597	104.525323	384		Farmer owned field
SCJY2052	2014	Sichuan	Rice	*F. asiaticum*	NIV	30.446597	104.525323	384		Farmer owned field
SCJY2051	2014	Sichuan	Rice	*F. asiaticum*	NIV	30.446597	104.525323	384		Farmer owned field
SCJY2050	2014	Sichuan	Rice	*F. asiaticum*	NIV	30.446597	104.525323	384		Farmer owned field
SCJY2049	2014	Sichuan	Rice	*F. asiaticum*	NIV	30.446597	104.525323	384		Farmer owned field
SCJY2048	2014	Sichuan	Rice	*F. asiaticum*	NIV	30.446597	104.525323	384		Farmer owned field
SCJY2047	2014	Sichuan	Rice	*F. asiaticum*	NIV	30.446597	104.525323	384		Farmer owned field
SCJY2045	2014	Sichuan	Rice	*F. asiaticum*	NIV	30.446597	104.525323	384		Farmer owned field
SCJY2044	2014	Sichuan	Rice	*F. asiaticum*	NIV	30.446597	104.525323	384		Farmer owned field
SCJY2043	2014	Sichuan	Rice	*F. asiaticum*	NIV	30.446597	104.525323	384		Farmer owned field
SCJY2042	2014	Sichuan	Rice	*F. asiaticum*	NIV	30.446597	104.525323	384		Farmer owned field
SCJY2040	2014	Sichuan	Rice	*F. asiaticum*	NIV	30.446597	104.525323	384		Farmer owned field
SCJY2035	2014	Sichuan	Rice	*F. asiaticum*	NIV	30.446597	104.525323	384		Farmer owned field
SCJY2034	2014	Sichuan	Rice	*F. asiaticum*	NIV	30.446597	104.525323	384		Farmer owned field

SCJY2031	2014	Sichuan	Rice	*F. asiaticum*	NIV	30.446597	104.525323	384		Farmer owned field
SCJY2029	2014	Sichuan	Rice	*F. asiaticum*	NIV	30.446597	104.525323	384		Farmer owned field
SCJY2028	2014	Sichuan	Rice	*F. asiaticum*	NIV	30.446597	104.525323	384		Farmer owned field
SCJY2024	2014	Sichuan	Rice	*F. asiaticum*	NIV	30.446597	104.525323	384		Farmer owned field
SCJY2023	2014	Sichuan	Rice	*F. asiaticum*	NIV	30.446597	104.525323	384		Farmer owned field
SCJY2022	2014	Sichuan	Rice	*F. asiaticum*	NIV	30.446597	104.525323	384		Farmer owned field
SCJY2019	2014	Sichuan	Rice	*F. asiaticum*	NIV	30.446597	104.525323	384		Farmer owned field
SCJY2016	2014	Sichuan	Rice	*F. asiaticum*	NIV	30.446597	104.525323	384		Farmer owned field
SCJY2015	2014	Sichuan	Rice	*F. asiaticum*	NIV	30.446597	104.525323	384		Farmer owned field
SCJY2010	2014	Sichuan	Rice	*F. asiaticum*	NIV	30.446597	104.525323	384		Farmer owned field
SCJY2009	2014	Sichuan	Rice	*F. asiaticum*	NIV	30.446597	104.525323	384		Farmer owned field
SCJY2008	2014	Sichuan	Rice	*F. asiaticum*	NIV	30.446597	104.525323	384		Farmer owned field
SCJY2005	2014	Sichuan	Rice	*F. asiaticum*	NIV	30.446597	104.525323	384		Farmer owned field
SCJY2004	2014	Sichuan	Rice	*F. asiaticum*	NIV	30.446597	104.525323	384		Farmer owned field
SCJY2003	2014	Sichuan	Rice	*F. asiaticum*	NIV	30.446597	104.525323	384		Farmer owned field
SCJY2002	2014	Sichuan	Rice	*F. asiaticum*	NIV	30.446597	104.525323	384		Farmer owned field
SCJY2001	2014	Sichuan	Rice	*F. asiaticum*	NIV	30.446597	104.525323	384		Farmer owned field
SCJY1034	2014	Sichuan	Rice	*F. asiaticum*	NIV	30.446597	104.525323	384		Farmer owned field
SCJY1033	2014	Sichuan	Rice	*F. asiaticum*	NIV	30.446597	104.525323	384		Farmer owned field
SCJY1032	2014	Sichuan	Rice	*F. asiaticum*	NIV	30.446597	104.525323	384		Farmer owned field
SCJY1031	2014	Sichuan	Rice	*F. asiaticum*	NIV	30.446597	104.525323	384		Farmer owned field
SCJY1029	2014	Sichuan	Rice	*F. asiaticum*	NIV	30.446597	104.525323	384		Farmer owned field
SCJY1028	2014	Sichuan	Rice	*F. asiaticum*	NIV	30.446597	104.525323	384		Farmer owned field
SCJY1027	2014	Sichuan	Rice	*F. asiaticum*	NIV	30.446597	104.525323	384		Farmer owned field
SCJY1025	2014	Sichuan	Rice	*F. asiaticum*	NIV	30.446597	104.525323	384		Farmer owned field
SCJY1023	2014	Sichuan	Rice	*F. asiaticum*	NIV	30.446597	104.525323	384		Farmer owned field
SCJY1019	2014	Sichuan	Rice	*F. asiaticum*	NIV	30.446597	104.525323	384		Farmer owned field
SCJY1018	2014	Sichuan	Rice	*F. asiaticum*	NIV	30.446597	104.525323	384		Farmer owned field
SCJY1017	2014	Sichuan	Rice	*F. asiaticum*	NIV	30.446597	104.525323	384		Farmer owned field
SCJY1016	2014	Sichuan	Rice	*F. asiaticum*	NIV	30.446597	104.525323	384		Farmer owned field
SCJY1015	2014	Sichuan	Rice	*F. asiaticum*	NIV	30.446597	104.525323	384		Farmer owned field
SCJY1014	2014	Sichuan	Rice	*F. asiaticum*	NIV	30.446597	104.525323	384		Farmer owned field
SCJY1012	2014	Sichuan	Rice	*F. asiaticum*	NIV	30.446597	104.525323	384		Farmer owned field
SCJY1008	2014	Sichuan	Rice	*F. asiaticum*	NIV	30.446597	104.525323	384		Farmer owned field
SCJY1007	2014	Sichuan	Rice	*F. asiaticum*	NIV	30.446597	104.525323	384		Farmer owned field

SCJY1003	2014	Sichuan	Rice	*F. asiaticum*	NIV	30.446597	104.525323	384		Farmer owned field
SCJY1002	2014	Sichuan	Rice	*F. asiaticum*	NIV	30.446597	104.525323	384		Farmer owned field
180895	2018	Sichuan	Wheat	*F. asiaticum*	NIV	30.48600099	103.574301	504.300323	Commercialized variety	Farmer owned field
180894	2018	Sichuan	Wheat	*F. asiaticum*	NIV	30.48600099	103.574301	504.300323	Commercialized variety	Farmer owned field
180893	2018	Sichuan	Wheat	*F. asiaticum*	NIV	30.48600099	103.574301	504.300323	Commercialized variety	Farmer owned field
180889	2018	Sichuan	Wheat	*F. asiaticum*	NIV	30.48600099	103.574301	504.300323	Commercialized variety	Farmer owned field
180888	2018	Sichuan	Wheat	*F. asiaticum*	NIV	30.48600099	103.574301	504.300323	Commercialized variety	Farmer owned field
180887	2018	Sichuan	Wheat	*F. asiaticum*	NIV	30.48600099	103.574301	504.300323	Commercialized variety	Farmer owned field
180884	2018	Sichuan	Wheat	*F. asiaticum*	NIV	30.48600099	103.574301	504.300323	Commercialized variety	Farmer owned field
180882	2018	Sichuan	Wheat	*F. asiaticum*	NIV	30.48600099	103.574301	504.300323	Commercialized variety	Farmer owned field
180880	2018	Sichuan	Wheat	*F. asiaticum*	NIV	30.48600099	103.574301	504.300323	Commercialized variety	Farmer owned field
180878	2018	Sichuan	Wheat	*F. asiaticum*	NIV	30.48600099	103.574301	504.300323	Commercialized variety	Farmer owned field
180876	2018	Sichuan	Wheat	*F. asiaticum*	NIV	30.48600099	103.574301	504.300323	Commercialized variety	Farmer owned field
180873	2018	Sichuan	Wheat	*F. asiaticum*	NIV	30.48600099	103.574301	504.300323	Commercialized variety	Farmer owned field
180872	2018	Sichuan	Wheat	*F. asiaticum*	NIV	30.48600099	103.574301	504.300323	Commercialized variety	Farmer owned field
180871	2018	Sichuan	Wheat	*F. asiaticum*	NIV	30.48600099	103.574301	504.300323	Commercialized variety	Farmer owned field
180869	2018	Sichuan	Wheat	*F. asiaticum*	15ADON	30.48600099	103.574301	504.300323	Commercialized variety	Farmer owned field
180868	2018	Sichuan	Wheat	*F. asiaticum*	NIV	30.48600099	103.574301	504.300323	Commercialized variety	Farmer owned field
180867	2018	Sichuan	Wheat	*F. asiaticum*	NIV	30.48600099	103.574301	504.300323	Commercialized variety	Farmer owned field
180866	2018	Sichuan	Wheat	*F. asiaticum*	NIV	30.48600099	103.574301	504.300323	Commercialized variety	Farmer owned field
180862	2018	Sichuan	Wheat	*F. asiaticum*	NIV	30.48600099	103.574301	504.300323	Commercialized variety	Farmer owned field
180860	2018	Sichuan	Wheat	*F. asiaticum*	15ADON	30.48600099	103.574301	504.300323	Commercialized variety	Farmer owned field
180855	2018	Sichuan	Wheat	*F. asiaticum*	NIV	30.48600099	103.574301	504.300323	Commercialized variety	Farmer owned field
180853	2018	Sichuan	Wheat	*F. asiaticum*	NIV	30.48600099	103.574301	504.300323	Commercialized variety	Farmer owned field
180843	2018	Sichuan	Wheat	*F. asiaticum*	NIV	30.48600099	103.574301	504.300323	Commercialized variety	Farmer owned field
180840	2018	Sichuan	Wheat	*F. asiaticum*	NIV	30.48600099	103.574301	504.300323	Commercialized variety	Farmer owned field
180837	2018	Sichuan	Wheat	*F. asiaticum*	NIV	30.48600099	103.574301	504.300323	Commercialized variety	Farmer owned field
181686	2018	Sichuan	Rice	*F. asiaticum*	NIV	30.48600099	103.574301	504.300323		Farmer owned field
181685	2018	Sichuan	Rice	*F. asiaticum*	NIV	30.48600099	103.574301	504.300323		Farmer owned field
181684	2018	Sichuan	Rice	*F. asiaticum*	NIV	30.48600099	103.574301	504.300323		Farmer owned field
181682	2018	Sichuan	Rice	*F. asiaticum*	NIV	30.48600099	103.574301	504.300323		Farmer owned field
181681	2018	Sichuan	Rice	*F. asiaticum*	NIV	30.48600099	103.574301	504.300323		Farmer owned field
181680	2018	Sichuan	Rice	*F. asiaticum*	NIV	30.48600099	103.574301	504.300323		Farmer owned field
181679	2018	Sichuan	Rice	*F. asiaticum*	NIV	30.48600099	103.574301	504.300323		Farmer owned field
181677	2018	Sichuan	Rice	*F. asiaticum*	NIV	30.48600099	103.574301	504.300323		Farmer owned field

181676	2018	Sichuan	Rice	*F. asiaticum*	NIV	30.48600099	103.574301	504.300323		Farmer owned field
181675	2018	Sichuan	Rice	*F. asiaticum*	NIV	30.48600099	103.574301	504.300323		Farmer owned field
181674	2018	Sichuan	Rice	*F. asiaticum*	NIV	30.48600099	103.574301	504.300323		Farmer owned field
181673	2018	Sichuan	Rice	*F. asiaticum*	NIV	30.48600099	103.574301	504.300323		Farmer owned field
180834	2018	Sichuan	Wheat	*F. asiaticum*	NIV	30.55742503	103.66112	515.423706	Commercialized variety	Farmer owned field
180831	2018	Sichuan	Wheat	*F. asiaticum*	NIV	30.55742503	103.66112	515.423706	Commercialized variety	Farmer owned field
180830	2018	Sichuan	Wheat	*F. asiaticum*	NIV	30.55742503	103.66112	515.423706	Commercialized variety	Farmer owned field
180829	2018	Sichuan	Wheat	*F. asiaticum*	NIV	30.55742503	103.66112	515.423706	Commercialized variety	Farmer owned field
180828	2018	Sichuan	Wheat	*F. asiaticum*	NIV	30.55742503	103.66112	515.423706	Commercialized variety	Farmer owned field
180826	2018	Sichuan	Wheat	*F. asiaticum*	NIV	30.55742503	103.66112	515.423706	Commercialized variety	Farmer owned field
180825	2018	Sichuan	Wheat	*F. asiaticum*	NIV	30.55742503	103.66112	515.423706	Commercialized variety	Farmer owned field
180823	2018	Sichuan	Wheat	*F. asiaticum*	NIV	30.55742503	103.66112	515.423706	Commercialized variety	Farmer owned field
180822	2018	Sichuan	Wheat	*F. asiaticum*	NIV	30.55742503	103.66112	515.423706	Commercialized variety	Farmer owned field
180821	2018	Sichuan	Wheat	*F. asiaticum*	NIV	30.55742503	103.66112	515.423706	Commercialized variety	Farmer owned field
180816	2018	Sichuan	Wheat	*F. asiaticum*	NIV	30.55742503	103.66112	515.423706	Commercialized variety	Farmer owned field
180811	2018	Sichuan	Wheat	*F. asiaticum*	NIV	30.55742503	103.66112	515.423706	Commercialized variety	Farmer owned field
180579	2018	Hubei	Rice	*F. asiaticum*	NIV	30.86147396	112.867559	68.377945		Commercial farm
180578	2018	Hubei	Rice	*F. asiaticum*	3ADON	30.86147396	112.867559	68.377945		Commercial farm
180577	2018	Hubei	Rice	*F. asiaticum*	NIV	30.86147396	112.867559	68.377945		Commercial farm
180576	2018	Hubei	Rice	*F. asiaticum*	NIV	30.86147396	112.867559	68.377945		Commercial farm
180575	2018	Hubei	Rice	*F. asiaticum*	3ADON	30.86147396	112.867559	68.377945		Commercial farm
180574	2018	Hubei	Rice	*F. asiaticum*	3ADON	30.86147396	112.867559	68.377945		Commercial farm
180573	2018	Hubei	Rice	*F. asiaticum*	NIV	30.86147396	112.867559	68.377945		Commercial farm
180572	2018	Hubei	Rice	*F. asiaticum*	NIV	30.86147396	112.867559	68.377945		Commercial farm
180571	2018	Hubei	Rice	*F. asiaticum*	NIV	30.86147396	112.867559	68.377945		Commercial farm
180570	2018	Hubei	Rice	*F. asiaticum*	3ADON	30.86147396	112.867559	68.377945		Commercial farm
180569	2018	Hubei	Rice	*F. asiaticum*	NIV	30.86147396	112.867559	68.377945		Commercial farm
180568	2018	Hubei	Rice	*F. asiaticum*	15ADON	30.86147396	112.867559	68.377945		Commercial farm
180567	2018	Hubei	Rice	*F. asiaticum*	3ADON	30.86147396	112.867559	68.377945		Commercial farm
180566	2018	Hubei	Rice	*F. asiaticum*	3ADON	30.86147396	112.867559	68.377945		Commercial farm
180565	2018	Hubei	Rice	*F. asiaticum*	3ADON	30.86147396	112.867559	68.377945		Commercial farm
180564	2018	Hubei	Rice	*F. asiaticum*	15ADON	30.86147396	112.867559	68.377945		Commercial farm
180563	2018	Hubei	Rice	*F. asiaticum*	3ADON	30.86147396	112.867559	68.377945		Commercial farm
180562	2018	Hubei	Rice	*F. asiaticum*	15ADON	30.86147396	112.867559	68.377945		Commercial farm
180560	2018	Hubei	Rice	*F. asiaticum*	3ADON	30.86147396	112.867559	68.377945		Commercial farm

180559	2018	Hubei	Rice	*F. asiaticum*	3ADON	30.86147396	112.867559	68.377945		Commercial farm
180483	2018	Hubei	Rice	*F. asiaticum*	3ADON	30.89166398	113.588332	28.714087		Farmer owned field
180482	2018	Hubei	Rice	*F. asiaticum*	3ADON	30.89166398	113.588332	28.714087		Farmer owned field
180481	2018	Hubei	Rice	*F. asiaticum*	3ADON	30.89166398	113.588332	28.714087		Farmer owned field
180478	2018	Hubei	Rice	*F. asiaticum*	3ADON	30.89166398	113.588332	28.714087		Farmer owned field
180477	2018	Hubei	Rice	*F. asiaticum*	3ADON	30.89166398	113.588332	28.714087		Farmer owned field
180476	2018	Hubei	Rice	*F. asiaticum*	3ADON	30.89166398	113.588332	28.714087		Farmer owned field
180474	2018	Hubei	Rice	*F. asiaticum*	NIV	30.89166398	113.588332	28.714087		Farmer owned field
180473	2018	Hubei	Rice	*F. asiaticum*	3ADON	30.89166398	113.588332	28.714087		Farmer owned field
180472	2018	Hubei	Rice	*F. asiaticum*	NIV	30.89166398	113.588332	28.714087		Farmer owned field
180471	2018	Hubei	Rice	*F. asiaticum*	3ADON	30.89166398	113.588332	28.714087		Farmer owned field
180470	2018	Hubei	Rice	*F. asiaticum*	3ADON	30.89166398	113.588332	28.714087		Farmer owned field
180469	2018	Hubei	Rice	*F. asiaticum*	3ADON	30.89166398	113.588332	28.714087		Farmer owned field
191281	2019	Jiangsu	Wheat	*F. asiaticum*	3ADON	33.26554502	118.883774	18.811953	Commercialized variety	Farmer owned field
191280	2019	Jiangsu	Wheat	*F. asiaticum*	3ADON	33.26554502	118.883774	18.811953	Commercialized variety	Farmer owned field
191279	2019	Jiangsu	Wheat	*F. asiaticum*	3ADON	33.26554502	118.883774	18.811953	Commercialized variety	Farmer owned field
191278	2019	Jiangsu	Wheat	*F. asiaticum*	3ADON	33.26554502	118.883774	18.811953	Commercialized variety	Farmer owned field
191277	2019	Jiangsu	Wheat	*F. asiaticum*	3ADON	33.26554502	118.883774	18.811953	Commercialized variety	Farmer owned field
191276	2019	Jiangsu	Wheat	*F. asiaticum*	3ADON	33.26554502	118.883774	18.811953	Commercialized variety	Farmer owned field
191275	2019	Jiangsu	Wheat	*F. asiaticum*	3ADON	33.26554502	118.883774	18.811953	Commercialized variety	Farmer owned field
191274	2019	Jiangsu	Wheat	*F. asiaticum*	3ADON	33.26554502	118.883774	18.811953	Commercialized variety	Farmer owned field
191273	2019	Jiangsu	Wheat	*F. asiaticum*	3ADON	33.26554502	118.883774	18.811953	Commercialized variety	Farmer owned field
192926	2019	Jiangsu	Wheat	*F. asiaticum*	3ADON	33.28410398	120.603738	88.055298	Commercialized variety	Farmer owned field
192925	2019	Jiangsu	Wheat	*F. asiaticum*	3ADON	33.28410398	120.603738	88.055298	Commercialized variety	Farmer owned field
192924	2019	Jiangsu	Wheat	*F. asiaticum*	3ADON	33.28410398	120.603738	88.055298	Commercialized variety	Farmer owned field
192923	2019	Jiangsu	Wheat	*F. asiaticum*	3ADON	33.28410398	120.603738	88.055298	Commercialized variety	Farmer owned field
192922	2019	Jiangsu	Wheat	*F. asiaticum*	3ADON	33.28410398	120.603738	88.055298	Commercialized variety	Farmer owned field
192917	2019	Jiangsu	Wheat	*F. asiaticum*	3ADON	33.28410398	120.603738	88.055298	Commercialized variety	Farmer owned field
192916	2019	Jiangsu	Wheat	*F. asiaticum*	3ADON	33.28410398	120.603738	88.055298	Commercialized variety	Farmer owned field
192915	2019	Jiangsu	Wheat	*F. asiaticum*	3ADON	33.28410398	120.603738	88.055298	Commercialized variety	Farmer owned field
192913	2019	Jiangsu	Wheat	*F. asiaticum*	3ADON	33.28410398	120.603738	88.055298	Commercialized variety	Farmer owned field
192912	2019	Jiangsu	Wheat	*F. asiaticum*	3ADON	33.28410398	120.603738	88.055298	Commercialized variety	Farmer owned field
192911	2019	Jiangsu	Wheat	*F. asiaticum*	3ADON	33.28410398	120.603738	88.055298	Commercialized variety	Farmer owned field
180807	2018	Sichuan	Wheat	*F. asiaticum*	NIV	30.55742503	103.66112	515.423706	Commercialized variety	Farmer owned field
180803	2018	Sichuan	Wheat	*F. asiaticum*	NIV	30.55742503	103.66112	515.423706	Commercialized variety	Farmer owned field

180801	2018	Sichuan	Wheat	*F. asiaticum*	NIV	30.55742503	103.66112	515.423706	Commercialized variety	Farmer owned field
180800	2018	Sichuan	Wheat	*F. asiaticum*	NIV	30.55742503	103.66112	515.423706	Commercialized variety	Farmer owned field
172120	2017	Sichuan	Wheat	*F. asiaticum*	NIV	30.562533	103.657882	499.5	Commercialized variety	Farmer owned field
172119	2017	Sichuan	Wheat	*F. asiaticum*	NIV	30.562533	103.657882	499.5	Commercialized variety	Farmer owned field
172118	2017	Sichuan	Wheat	*F. asiaticum*	NIV	30.562533	103.657882	499.5	Commercialized variety	Farmer owned field
172117	2017	Sichuan	Wheat	*F. asiaticum*	NIV	30.562533	103.657882	499.5	Commercialized variety	Farmer owned field
192910	2019	Jiangsu	Wheat	*F. asiaticum*	3ADON	33.28410398	120.603738	88.055298	Commercialized variety	Farmer owned field
192909	2019	Jiangsu	Wheat	*F. asiaticum*	3ADON	33.28410398	120.603738	88.055298	Commercialized variety	Farmer owned field
182658	2018	Jiangsu	Wheat	*F. asiaticum*	3ADON	33.28812202	120.166226	1.783241	Commercialized variety	Farmer owned field
182657	2018	Jiangsu	Wheat	*F. asiaticum*	3ADON	33.28812202	120.166226	1.783241	Commercialized variety	Farmer owned field
182656	2018	Jiangsu	Wheat	*F. asiaticum*	3ADON	33.28812202	120.166226	1.783241	Commercialized variety	Farmer owned field
182655	2018	Jiangsu	Wheat	*F. asiaticum*	NIV	33.28812202	120.166226	1.783241	Commercialized variety	Farmer owned field
172114	2017	Sichuan	Wheat	*F. asiaticum*	NIV	30.562533	103.657882	499.5	Commercialized variety	Farmer owned field
171484	2017	Sichuan	Rice	*F. asiaticum*	NIV	30.710058	103.86309	517.4		Farmer owned field
171483	2017	Sichuan	Rice	*F. asiaticum*	NIV	30.710058	103.86309	517.4		Farmer owned field
171482	2017	Sichuan	Rice	*F. asiaticum*	NIV	30.710058	103.86309	517.4		Farmer owned field
171481	2017	Sichuan	Rice	*F. asiaticum*	NIV	30.710058	103.86309	517.4		Farmer owned field
171478	2017	Sichuan	Rice	*F. asiaticum*	NIV	30.710058	103.86309	517.4		Farmer owned field
171477	2017	Sichuan	Rice	*F. asiaticum*	NIV	30.710058	103.86309	517.4		Farmer owned field
171476	2017	Sichuan	Rice	*F. asiaticum*	NIV	30.710058	103.86309	517.4		Farmer owned field
171475	2017	Sichuan	Rice	*F. asiaticum*	NIV	30.710058	103.86309	517.4		Farmer owned field
171472	2017	Sichuan	Rice	*F. asiaticum*	NIV	30.710058	103.86309	517.4		Farmer owned field
171471	2017	Sichuan	Rice	*F. asiaticum*	NIV	30.710058	103.86309	517.4		Farmer owned field
171470	2017	Sichuan	Rice	*F. asiaticum*	NIV	30.710058	103.86309	517.4		Farmer owned field
171467	2017	Sichuan	Rice	*F. asiaticum*	NIV	30.710058	103.86309	517.4		Farmer owned field
171466	2017	Sichuan	Rice	*F. asiaticum*	NIV	30.710058	103.86309	517.4		Farmer owned field
171465	2017	Sichuan	Rice	*F. asiaticum*	NIV	30.710058	103.86309	517.4		Farmer owned field
180792	2018	Sichuan	Wheat	*F. asiaticum*	NIV	30.71890701	103.872721	530.571655	Commercialized variety	Farmer owned field
180790	2018	Sichuan	Wheat	*F. asiaticum*	3ADON	30.71890701	103.872721	530.571655	Commercialized variety	Farmer owned field
180786	2018	Sichuan	Wheat	*F. asiaticum*	NIV	30.71890701	103.872721	530.571655	Commercialized variety	Farmer owned field
180778	2018	Sichuan	Wheat	*F. asiaticum*	3ADON	30.71890701	103.872721	530.571655	Commercialized variety	Farmer owned field
180768	2018	Sichuan	Wheat	*F. asiaticum*	NIV	30.71890701	103.872721	530.571655	Commercialized variety	Farmer owned field
180468	2018	Hubei	Rice	*F. asiaticum*	3ADON	30.89166398	113.588332	28.714087		Farmer owned field
180467	2018	Hubei	Rice	*F. asiaticum*	3ADON	30.89166398	113.588332	28.714087		Farmer owned field
180466	2018	Hubei	Rice	*F. asiaticum*	3ADON	30.89166398	113.588332	28.714087		Farmer owned field

180465	2018	Hubei	Rice	*F. asiaticum*	3ADON	30.89166398	113.588332	28.714087		Farmer owned field
180464	2018	Hubei	Rice	*F. asiaticum*	3ADON	30.89166398	113.588332	28.714087		Farmer owned field
180463	2018	Hubei	Rice	*F. asiaticum*	NIV	30.89166398	113.588332	28.714087		Farmer owned field
180462	2018	Hubei	Rice	*F. asiaticum*	NIV	30.89166398	113.588332	28.714087		Farmer owned field
180461	2018	Hubei	Rice	*F. asiaticum*	3ADON	30.89166398	113.588332	28.714087		Farmer owned field
180460	2018	Hubei	Rice	*F. asiaticum*	NIV	30.89166398	113.588332	28.714087		Farmer owned field
180459	2018	Hubei	Rice	*F. asiaticum*	3ADON	30.89166398	113.588332	28.714087		Farmer owned field
180458	2018	Hubei	Rice	*F. asiaticum*	3ADON	30.89166398	113.588332	28.714087		Farmer owned field
180457	2018	Hubei	Rice	*F. asiaticum*	NIV	30.89166398	113.588332	28.714087		Farmer owned field
180456	2018	Hubei	Rice	*F. asiaticum*	3ADON	30.89166398	113.588332	28.714087		Farmer owned field
180455	2018	Hubei	Rice	*F. asiaticum*	3ADON	30.89166398	113.588332	28.714087		Farmer owned field
180454	2018	Hubei	Rice	*F. asiaticum*	3ADON	30.89166398	113.588332	28.714087		Farmer owned field
180453	2018	Hubei	Rice	*F. asiaticum*	3ADON	30.89166398	113.588332	28.714087		Farmer owned field
180452	2018	Hubei	Rice	*F. asiaticum*	3ADON	30.89166398	113.588332	28.714087		Farmer owned field
180451	2018	Hubei	Rice	*F. asiaticum*	3ADON	30.89166398	113.588332	28.714087		Farmer owned field
HBXG98	2014	Hubei	Wheat	*F. asiaticum*	15ADON	30.892518	113.933865	24	Commercialized variety	Commercial farm
HBXG8	2014	Hubei	Wheat	*F. asiaticum*	3ADON	30.892518	113.933865	24	Commercialized variety	Commercial farm
HBXG69	2014	Hubei	Wheat	*F. asiaticum*	3ADON	30.892518	113.933865	24	Commercialized variety	Commercial farm
HBXG40	2014	Hubei	Wheat	*F. asiaticum*	3ADON	30.892518	113.933865	24	Commercialized variety	Commercial farm
HBXG39	2014	Hubei	Wheat	*F. asiaticum*	15ADON	30.892518	113.933865	24	Commercialized variety	Commercial farm
HBXG31	2014	Hubei	Wheat	*F. asiaticum*	3ADON	30.892518	113.933865	24	Commercialized variety	Commercial farm
HBXG28	2014	Hubei	Wheat	*F. asiaticum*	3ADON	30.892518	113.933865	24	Commercialized variety	Commercial farm
HBXG27	2014	Hubei	Wheat	*F. asiaticum*	3ADON	30.892518	113.933865	24	Commercialized variety	Commercial farm
HBXG24	2014	Hubei	Wheat	*F. asiaticum*	3ADON	30.892518	113.933865	24	Commercialized variety	Commercial farm
HBXG22	2014	Hubei	Wheat	*F. asiaticum*	3ADON	30.892518	113.933865	24	Commercialized variety	Commercial farm
HBXG215	2014	Hubei	Wheat	*F. asiaticum*	3ADON	30.892518	113.933865	24	Commercialized variety	Commercial farm
HBXG2	2014	Hubei	Wheat	*F. asiaticum*	3ADON	30.892518	113.933865	24	Commercialized variety	Commercial farm
HBXG195	2014	Hubei	Wheat	*F. asiaticum*	3ADON	30.892518	113.933865	24	Commercialized variety	Commercial farm
HBXG185	2014	Hubei	Wheat	*F. asiaticum*	3ADON	30.892518	113.933865	24	Commercialized variety	Commercial farm
HBXG183	2014	Hubei	Wheat	*F. asiaticum*	3ADON	30.892518	113.933865	24	Commercialized variety	Commercial farm
HBXG180	2014	Hubei	Wheat	*F. asiaticum*	3ADON	30.892518	113.933865	24	Commercialized variety	Commercial farm
HBXG179	2014	Hubei	Wheat	*F. asiaticum*	3ADON	30.892518	113.933865	24	Commercialized variety	Commercial farm
HBXG178	2014	Hubei	Wheat	*F. asiaticum*	3ADON	30.892518	113.933865	24	Commercialized variety	Commercial farm
HBXG174	2014	Hubei	Wheat	*F. asiaticum*	3ADON	30.892518	113.933865	24	Commercialized variety	Commercial farm
HBXG173	2014	Hubei	Wheat	*F. asiaticum*	3ADON	30.892518	113.933865	24	Commercialized variety	Commercial farm

191518	2019	Anhui	Wheat	*F. asiaticum*	3ADON	32.78324604	115.686363	30.566353	Commercialized variety	Farmer owned field
191517	2019	Anhui	Wheat	*F. asiaticum*	15ADON	32.78324604	115.686363	30.566353	Commercialized variety	Farmer owned field
191516	2019	Anhui	Wheat	*F. asiaticum*	3ADON	32.78324604	115.686363	30.566353	Commercialized variety	Farmer owned field
191515	2019	Anhui	Wheat	*F. asiaticum*	3ADON	32.78324604	115.686363	30.566353	Commercialized variety	Farmer owned field
191513	2019	Anhui	Wheat	*F. asiaticum*	3ADON	32.78324604	115.686363	30.566353	Commercialized variety	Farmer owned field
191511	2019	Anhui	Wheat	*F. asiaticum*	3ADON	32.78324604	115.686363	30.566353	Commercialized variety	Farmer owned field
191510	2019	Anhui	Wheat	*F. asiaticum*	NIV	32.78324604	115.686363	30.566353	Commercialized variety	Farmer owned field
191509	2019	Anhui	Wheat	*F. asiaticum*	3ADON	32.78324604	115.686363	30.566353	Commercialized variety	Farmer owned field
182475	2018	Anhui	Wheat	*F. asiaticum*	3ADON	32.78621298	116.628252	25.395546	Commercialized variety	Farmer owned field
182474	2018	Anhui	Wheat	*F. asiaticum*	3ADON	32.78621298	116.628252	25.395546	Commercialized variety	Farmer owned field
182473	2018	Anhui	Wheat	*F. asiaticum*	3ADON	32.78621298	116.628252	25.395546	Commercialized variety	Farmer owned field
182471	2018	Anhui	Wheat	*F. asiaticum*	NIV	32.78621298	116.628252	25.395546	Commercialized variety	Farmer owned field
182470	2018	Anhui	Wheat	*F. asiaticum*	3ADON	32.78621298	116.628252	25.395546	Commercialized variety	Farmer owned field
182469	2018	Anhui	Wheat	*F. asiaticum*	NIV	32.78621298	116.628252	25.395546	Commercialized variety	Farmer owned field
182468	2018	Anhui	Wheat	*F. asiaticum*	3ADON	32.78621298	116.628252	25.395546	Commercialized variety	Farmer owned field
182467	2018	Anhui	Wheat	*F. asiaticum*	3ADON	32.78621298	116.628252	25.395546	Commercialized variety	Farmer owned field
182466	2018	Anhui	Wheat	*F. asiaticum*	3ADON	32.78621298	116.628252	25.395546	Commercialized variety	Farmer owned field
182465	2018	Anhui	Wheat	*F. asiaticum*	NIV	32.78621298	116.628252	25.395546	Commercialized variety	Farmer owned field
182464	2018	Anhui	Wheat	*F. asiaticum*	NIV	32.78621298	116.628252	25.395546	Commercialized variety	Farmer owned field
182463	2018	Anhui	Wheat	*F. asiaticum*	3ADON	32.78621298	116.628252	25.395546	Commercialized variety	Farmer owned field
190555	2019	Anhui	Wheat	*F. asiaticum*	3ADON	32.83316899	117.299769	36.723686	Commercialized variety	Farmer owned field
190554	2019	Anhui	Wheat	*F. asiaticum*	3ADON	32.83316899	117.299769	36.723686	Commercialized variety	Farmer owned field
190553	2019	Anhui	Wheat	*F. asiaticum*	15ADON	32.83316899	117.299769	36.723686	Commercialized variety	Farmer owned field
190549	2019	Anhui	Wheat	*F. asiaticum*	3ADON	32.83316899	117.299769	36.723686	Commercialized variety	Farmer owned field
190547	2019	Anhui	Wheat	*F. asiaticum*	3ADON	32.83316899	117.299769	36.723686	Commercialized variety	Farmer owned field
190546	2019	Anhui	Wheat	*F. asiaticum*	3ADON	32.83316899	117.299769	36.723686	Commercialized variety	Farmer owned field
190545	2019	Anhui	Wheat	*F. asiaticum*	3ADON	32.83316899	117.299769	36.723686	Commercialized variety	Farmer owned field
181672	2018	Sichuan	Rice	*F. asiaticum*	NIV	30.71890701	103.872721	530.571655		Farmer owned field
181671	2018	Sichuan	Rice	*F. asiaticum*	NIV	30.71890701	103.872721	530.571655		Farmer owned field
181670	2018	Sichuan	Rice	*F. asiaticum*	NIV	30.71890701	103.872721	530.571655		Farmer owned field
181669	2018	Sichuan	Rice	*F. asiaticum*	NIV	30.71890701	103.872721	530.571655		Farmer owned field
181668	2018	Sichuan	Rice	*F. asiaticum*	NIV	30.71890701	103.872721	530.571655		Farmer owned field
181667	2018	Sichuan	Rice	*F. asiaticum*	NIV	30.71890701	103.872721	530.571655		Farmer owned field
181666	2018	Sichuan	Rice	*F. asiaticum*	NIV	30.71890701	103.872721	530.571655		Farmer owned field
181662	2018	Sichuan	Rice	*F. asiaticum*	NIV	30.71890701	103.872721	530.571655		Farmer owned field

181661	2018	Sichuan	Rice	*F. asiaticum*	NIV	30.71890701	103.872721	530.571655		Farmer owned field
181660	2018	Sichuan	Rice	*F. asiaticum*	NIV	30.71890701	103.872721	530.571655		Farmer owned field
181659	2018	Sichuan	Rice	*F. asiaticum*	NIV	30.71890701	103.872721	530.571655		Farmer owned field
181658	2018	Sichuan	Rice	*F. asiaticum*	3ADON	30.71890701	103.872721	530.571655		Farmer owned field
181657	2018	Sichuan	Rice	*F. asiaticum*	NIV	30.71890701	103.872721	530.571655		Farmer owned field
181656	2018	Sichuan	Rice	*F. asiaticum*	NIV	30.71890701	103.872721	530.571655		Farmer owned field
181655	2018	Sichuan	Rice	*F. asiaticum*	NIV	30.71890701	103.872721	530.571655		Farmer owned field
181654	2018	Sichuan	Rice	*F. asiaticum*	NIV	30.71890701	103.872721	530.571655		Farmer owned field
181653	2018	Sichuan	Rice	*F. asiaticum*	NIV	30.71890701	103.872721	530.571655		Farmer owned field
181650	2018	Sichuan	Rice	*F. asiaticum*	NIV	30.71890701	103.872721	530.571655		Farmer owned field
181649	2018	Sichuan	Rice	*F. asiaticum*	15ADON	30.71890701	103.872721	530.571655		Farmer owned field
181648	2018	Sichuan	Rice	*F. asiaticum*	15ADON	30.71890701	103.872721	530.571655		Farmer owned field
181646	2018	Sichuan	Rice	*F. asiaticum*	3ADON	30.71890701	103.872721	530.571655		Farmer owned field
GH3059	2014	Sichuan	Wheat	*F. asiaticum*	NIV	31.028224	104.265138	476	Commercialized variety	Farmer owned field
GH3058	2014	Sichuan	Wheat	*F. asiaticum*	NIV	31.028224	104.265138	476	Commercialized variety	Farmer owned field
GH3057	2014	Sichuan	Wheat	*F. asiaticum*	NIV	31.028224	104.265138	476	Commercialized variety	Farmer owned field
GH3056	2014	Sichuan	Wheat	*F. asiaticum*	NIV	31.028224	104.265138	476	Commercialized variety	Farmer owned field
GH3052	2014	Sichuan	Wheat	*F. asiaticum*	NIV	31.028224	104.265138	476	Commercialized variety	Farmer owned field
GH3051	2014	Sichuan	Wheat	*F. asiaticum*	NIV	31.028224	104.265138	476	Commercialized variety	Farmer owned field
GH3038	2014	Sichuan	Wheat	*F. asiaticum*	NIV	31.028224	104.265138	476	Commercialized variety	Farmer owned field
GH3032-2	2014	Sichuan	Wheat	*F. asiaticum*	NIV	31.028224	104.265138	476	Commercialized variety	Farmer owned field
GH3030	2014	Sichuan	Wheat	*F. asiaticum*	3ADON	31.028224	104.265138	476	Commercialized variety	Farmer owned field
GH3026	2014	Sichuan	Wheat	*F. asiaticum*	NIV	31.028224	104.265138	476	Commercialized variety	Farmer owned field
GH3025	2014	Sichuan	Wheat	*F. asiaticum*	NIV	31.028224	104.265138	476	Commercialized variety	Farmer owned field
GH3024	2014	Sichuan	Wheat	*F. asiaticum*	NIV	31.028224	104.265138	476	Commercialized variety	Farmer owned field
GH3021	2014	Sichuan	Wheat	*F. asiaticum*	NIV	31.028224	104.265138	476	Commercialized variety	Farmer owned field
GH3017	2014	Sichuan	Wheat	*F. asiaticum*	NIV	31.028224	104.265138	476	Commercialized variety	Farmer owned field
GH3015	2014	Sichuan	Wheat	*F. asiaticum*	NIV	31.028224	104.265138	476	Commercialized variety	Farmer owned field
GH3013	2014	Sichuan	Wheat	*F. asiaticum*	NIV	31.028224	104.265138	476	Commercialized variety	Farmer owned field
GH3010	2014	Sichuan	Wheat	*F. asiaticum*	NIV	31.028224	104.265138	476	Commercialized variety	Farmer owned field
GH3002	2014	Sichuan	Wheat	*F. asiaticum*	NIV	31.028224	104.265138	476	Commercialized variety	Farmer owned field
GH3001	2014	Sichuan	Wheat	*F. asiaticum*	NIV	31.028224	104.265138	476	Commercialized variety	Farmer owned field
GH2037	2014	Sichuan	Wheat	*F. asiaticum*	NIV	31.028224	104.265138	476	Commercialized variety	Farmer owned field
GH2035	2014	Sichuan	Wheat	*F. asiaticum*	NIV	31.028224	104.265138	476	Commercialized variety	Farmer owned field
GH2034	2014	Sichuan	Wheat	*F. asiaticum*	NIV	31.028224	104.265138	476	Commercialized variety	Farmer owned field

GH2033	2014	Sichuan	Wheat	*F. asiaticum*	NIV	31.028224	104.265138	476	Commercialized variety	Farmer owned field
GH2031	2014	Sichuan	Wheat	*F. asiaticum*	NIV	31.028224	104.265138	476	Commercialized variety	Farmer owned field
GH2027	2014	Sichuan	Wheat	*F. asiaticum*	NIV	31.028224	104.265138	476	Commercialized variety	Farmer owned field
GH2024	2014	Sichuan	Wheat	*F. asiaticum*	NIV	31.028224	104.265138	476	Commercialized variety	Farmer owned field
GH2022	2014	Sichuan	Wheat	*F. asiaticum*	NIV	31.028224	104.265138	476	Commercialized variety	Farmer owned field
GH2020	2014	Sichuan	Wheat	*F. asiaticum*	NIV	31.028224	104.265138	476	Commercialized variety	Farmer owned field
GH2019	2014	Sichuan	Wheat	*F. asiaticum*	NIV	31.028224	104.265138	476	Commercialized variety	Farmer owned field
GH2018	2014	Sichuan	Wheat	*F. asiaticum*	NIV	31.028224	104.265138	476	Commercialized variety	Farmer owned field
GH2009	2014	Sichuan	Wheat	*F. asiaticum*	NIV	31.028224	104.265138	476	Commercialized variety	Farmer owned field
GH2008	2014	Sichuan	Wheat	*F. asiaticum*	NIV	31.028224	104.265138	476	Commercialized variety	Farmer owned field
GH2007	2014	Sichuan	Wheat	*F. asiaticum*	NIV	31.028224	104.265138	476	Commercialized variety	Farmer owned field
GH2004	2014	Sichuan	Wheat	*F. asiaticum*	NIV	31.028224	104.265138	476	Commercialized variety	Farmer owned field
GH2002	2014	Sichuan	Wheat	*F. asiaticum*	NIV	31.028224	104.265138	476	Commercialized variety	Farmer owned field
SC27068	2014	Sichuan	Rice	*F. asiaticum*	NIV	31.09316	104.94041	413		Farmer owned field
SC27067	2014	Sichuan	Rice	*F. asiaticum*	NIV	31.09316	104.94041	413		Farmer owned field
SC27065	2014	Sichuan	Rice	*F. asiaticum*	NIV	31.09316	104.94041	413		Farmer owned field
SC27064	2014	Sichuan	Rice	*F. asiaticum*	NIV	31.09316	104.94041	413		Farmer owned field
SC27060	2014	Sichuan	Rice	*F. asiaticum*	NIV	31.09316	104.94041	413		Farmer owned field
SC27059	2014	Sichuan	Rice	*F. asiaticum*	NIV	31.09316	104.94041	413		Farmer owned field
SC27058	2014	Sichuan	Rice	*F. asiaticum*	NIV	31.09316	104.94041	413		Farmer owned field
SC27056	2014	Sichuan	Rice	*F. asiaticum*	NIV	31.09316	104.94041	413		Farmer owned field
SC27055	2014	Sichuan	Rice	*F. asiaticum*	NIV	31.09316	104.94041	413		Farmer owned field
SC27053	2014	Sichuan	Rice	*F. asiaticum*	NIV	31.09316	104.94041	413		Farmer owned field
SC27047	2014	Sichuan	Rice	*F. asiaticum*	NIV	31.09316	104.94041	413		Farmer owned field
SC27040	2014	Sichuan	Rice	*F. asiaticum*	NIV	31.09316	104.94041	413		Farmer owned field
SC27039	2014	Sichuan	Rice	*F. asiaticum*	NIV	31.09316	104.94041	413		Farmer owned field
SC27036	2014	Sichuan	Rice	*F. asiaticum*	NIV	31.09316	104.94041	413		Farmer owned field
SC27035	2014	Sichuan	Rice	*F. asiaticum*	NIV	31.09316	104.94041	413		Farmer owned field
SC27034	2014	Sichuan	Rice	*F. asiaticum*	NIV	31.09316	104.94041	413		Farmer owned field
SC27031	2014	Sichuan	Rice	*F. asiaticum*	NIV	31.09316	104.94041	413		Farmer owned field
SC27028	2014	Sichuan	Rice	*F. asiaticum*	NIV	31.09316	104.94041	413		Farmer owned field
SC27026	2014	Sichuan	Rice	*F. asiaticum*	NIV	31.09316	104.94041	413		Farmer owned field
SC27024	2014	Sichuan	Rice	*F. asiaticum*	NIV	31.09316	104.94041	413		Farmer owned field
SC27017	2014	Sichuan	Rice	*F. asiaticum*	NIV	31.09316	104.94041	413		Farmer owned field
SC27015	2014	Sichuan	Rice	*F. asiaticum*	NIV	31.09316	104.94041	413		Farmer owned field

SC27014	2014	Sichuan	Rice	*F. asiaticum*	NIV	31.09316	104.94041	413	Farmer owned field
SC27013	2014	Sichuan	Rice	*F. asiaticum*	NIV	31.09316	104.94041	413	Farmer owned field
SC27010	2014	Sichuan	Rice	*F. asiaticum*	NIV	31.09316	104.94041	413	Farmer owned field
SC27009	2014	Sichuan	Rice	*F. asiaticum*	NIV	31.09316	104.94041	413	Farmer owned field
SC27004	2014	Sichuan	Rice	*F. asiaticum*	NIV	31.09316	104.94041	413	Farmer owned field
SC27001	2014	Sichuan	Rice	*F. asiaticum*	NIV	31.09316	104.94041	413	Farmer owned field
SC24224	2014	Sichuan	Rice	*F. asiaticum*	NIV	31.09316	104.94041	413	Farmer owned field
SC24221	2014	Sichuan	Rice	*F. asiaticum*	NIV	31.09316	104.94041	413	Farmer owned field
SC24220	2014	Sichuan	Rice	*F. asiaticum*	NIV	31.09316	104.94041	413	Farmer owned field
SC24219	2014	Sichuan	Rice	*F. asiaticum*	NIV	31.09316	104.94041	413	Farmer owned field
SC24217	2014	Sichuan	Rice	*F. asiaticum*	NIV	31.09316	104.94041	413	Farmer owned field
SC24216	2014	Sichuan	Rice	*F. asiaticum*	NIV	31.09316	104.94041	413	Farmer owned field
SC24215	2014	Sichuan	Rice	*F. asiaticum*	NIV	31.09316	104.94041	413	Farmer owned field
SC24214	2014	Sichuan	Rice	*F. asiaticum*	NIV	31.09316	104.94041	413	Farmer owned field
SC24213	2014	Sichuan	Rice	*F. asiaticum*	NIV	31.09316	104.94041	413	Farmer owned field
SC24212	2014	Sichuan	Rice	*F. asiaticum*	NIV	31.09316	104.94041	413	Farmer owned field
SC24210	2014	Sichuan	Rice	*F. asiaticum*	NIV	31.09316	104.94041	413	Farmer owned field
SC24208	2014	Sichuan	Rice	*F. asiaticum*	NIV	31.09316	104.94041	413	Farmer owned field
SC24204	2014	Sichuan	Rice	*F. asiaticum*	NIV	31.09316	104.94041	413	Farmer owned field
SC24203	2014	Sichuan	Rice	*F. asiaticum*	NIV	31.09316	104.94041	413	Farmer owned field
SC24202	2014	Sichuan	Rice	*F. asiaticum*	NIV	31.09316	104.94041	413	Farmer owned field
SC24200	2014	Sichuan	Rice	*F. asiaticum*	NIV	31.09316	104.94041	413	Farmer owned field
SC24198-2	2014	Sichuan	Rice	*F. asiaticum*	NIV	31.09316	104.94041	413	Farmer owned field
SC24198	2014	Sichuan	Rice	*F. asiaticum*	NIV	31.09316	104.94041	413	Farmer owned field
SC24195	2014	Sichuan	Rice	*F. asiaticum*	NIV	31.09316	104.94041	413	Farmer owned field
SC24194	2014	Sichuan	Rice	*F. asiaticum*	NIV	31.09316	104.94041	413	Farmer owned field
SC24191	2014	Sichuan	Rice	*F. asiaticum*	NIV	31.09316	104.94041	413	Farmer owned field
SC24190	2014	Sichuan	Rice	*F. asiaticum*	NIV	31.09316	104.94041	413	Farmer owned field
SC24188	2014	Sichuan	Rice	*F. asiaticum*	NIV	31.09316	104.94041	413	Farmer owned field
SC24186	2014	Sichuan	Rice	*F. asiaticum*	NIV	31.09316	104.94041	413	Farmer owned field
SC24185	2014	Sichuan	Rice	*F. asiaticum*	NIV	31.09316	104.94041	413	Farmer owned field
SC24184-2	2014	Sichuan	Rice	*F. asiaticum*	NIV	31.09316	104.94041	413	Farmer owned field
SC24184	2014	Sichuan	Rice	*F. asiaticum*	NIV	31.09316	104.94041	413	Farmer owned field
SC24182	2014	Sichuan	Rice	*F. asiaticum*	NIV	31.09316	104.94041	413	Farmer owned field
SC24181	2014	Sichuan	Rice	*F. asiaticum*	NIV	31.09316	104.94041	413	Farmer owned field

SC24181	2014	Sichuan	Rice	*F. asiaticum*	NIV	31.09316	104.94041	413	Farmer owned field
SC24179	2014	Sichuan	Rice	*F. asiaticum*	NIV	31.09316	104.94041	413	Farmer owned field
SC24178	2014	Sichuan	Rice	*F. asiaticum*	NIV	31.09316	104.94041	413	Farmer owned field
SC24176	2014	Sichuan	Rice	*F. asiaticum*	NIV	31.09316	104.94041	413	Farmer owned field
SC24172	2014	Sichuan	Rice	*F. asiaticum*	NIV	31.09316	104.94041	413	Farmer owned field
SC24169	2014	Sichuan	Rice	*F. asiaticum*	NIV	31.09316	104.94041	413	Farmer owned field
SC24168	2014	Sichuan	Rice	*F. asiaticum*	NIV	31.09316	104.94041	413	Farmer owned field
SC24167	2014	Sichuan	Rice	*F. asiaticum*	NIV	31.09316	104.94041	413	Farmer owned field
SC24166	2014	Sichuan	Rice	*F. asiaticum*	NIV	31.09316	104.94041	413	Farmer owned field
SC24164	2014	Sichuan	Rice	*F. asiaticum*	NIV	31.09316	104.94041	413	Farmer owned field
SC24163	2014	Sichuan	Rice	*F. asiaticum*	NIV	31.09316	104.94041	413	Farmer owned field
SC24162	2014	Sichuan	Rice	*F. asiaticum*	NIV	31.09316	104.94041	413	Farmer owned field
SC23161	2014	Sichuan	Rice	*F. asiaticum*	NIV	31.09316	104.94041	413	Farmer owned field
SC23160	2014	Sichuan	Rice	*F. asiaticum*	NIV	31.09316	104.94041	413	Farmer owned field
SC23159	2014	Sichuan	Rice	*F. asiaticum*	NIV	31.09316	104.94041	413	Farmer owned field
SC23157	2014	Sichuan	Rice	*F. asiaticum*	NIV	31.09316	104.94041	413	Farmer owned field
SC23156	2014	Sichuan	Rice	*F. asiaticum*	NIV	31.09316	104.94041	413	Farmer owned field
SC23153	2014	Sichuan	Rice	*F. asiaticum*	NIV	31.09316	104.94041	413	Farmer owned field
SC23152	2014	Sichuan	Rice	*F. asiaticum*	NIV	31.09316	104.94041	413	Farmer owned field
SC23151	2014	Sichuan	Rice	*F. asiaticum*	NIV	31.09316	104.94041	413	Farmer owned field
SC23149	2014	Sichuan	Rice	*F. asiaticum*	NIV	31.09316	104.94041	413	Farmer owned field
SC23148	2014	Sichuan	Rice	*F. asiaticum*	NIV	31.09316	104.94041	413	Farmer owned field
SC23141	2014	Sichuan	Rice	*F. asiaticum*	NIV	31.09316	104.94041	413	Farmer owned field
SC23141	2014	Sichuan	Rice	*F. asiaticum*	NIV	31.09316	104.94041	413	Farmer owned field
SC23139	2014	Sichuan	Rice	*F. asiaticum*	NIV	31.09316	104.94041	413	Farmer owned field
SC23138	2014	Sichuan	Rice	*F. asiaticum*	NIV	31.09316	104.94041	413	Farmer owned field
SC23135-2	2014	Sichuan	Rice	*F. asiaticum*	NIV	31.09316	104.94041	413	Farmer owned field
SC23135	2014	Sichuan	Rice	*F. asiaticum*	NIV	31.09316	104.94041	413	Farmer owned field
SC23134	2014	Sichuan	Rice	*F. asiaticum*	NIV	31.09316	104.94041	413	Farmer owned field
SC23133	2014	Sichuan	Rice	*F. asiaticum*	NIV	31.09316	104.94041	413	Farmer owned field
SC23131	2014	Sichuan	Rice	*F. asiaticum*	NIV	31.09316	104.94041	413	Farmer owned field
SC23129	2014	Sichuan	Rice	*F. asiaticum*	NIV	31.09316	104.94041	413	Farmer owned field
SC23126	2014	Sichuan	Rice	*F. asiaticum*	NIV	31.09316	104.94041	413	Farmer owned field
SC23125	2014	Sichuan	Rice	*F. asiaticum*	NIV	31.09316	104.94041	413	Farmer owned field
SC23122	2014	Sichuan	Rice	*F. asiaticum*	NIV	31.09316	104.94041	413	Farmer owned field

SC23120	2014	Sichuan	Rice	*F. asiaticum*	NIV	31.09316	104.94041	413		Farmer owned field
SC23118-2	2014	Sichuan	Rice	*F. asiaticum*	NIV	31.09316	104.94041	413		Farmer owned field
SC23118	2014	Sichuan	Rice	*F. asiaticum*	NIV	31.09316	104.94041	413		Farmer owned field
SC23117	2014	Sichuan	Rice	*F. asiaticum*	NIV	31.09316	104.94041	413		Farmer owned field
SC23116	2014	Sichuan	Rice	*F. asiaticum*	NIV	31.09316	104.94041	413		Farmer owned field
SC23115	2014	Sichuan	Rice	*F. asiaticum*	NIV	31.09316	104.94041	413		Farmer owned field
SC23114	2014	Sichuan	Rice	*F. asiaticum*	NIV	31.09316	104.94041	413		Farmer owned field
SC23112	2014	Sichuan	Rice	*F. asiaticum*	NIV	31.09316	104.94041	413		Farmer owned field
SC23111	2014	Sichuan	Rice	*F. asiaticum*	NIV	31.09316	104.94041	413		Farmer owned field
SC23109	2014	Sichuan	Rice	*F. asiaticum*	NIV	31.09316	104.94041	413		Farmer owned field
SC23108	2014	Sichuan	Rice	*F. asiaticum*	NIV	31.09316	104.94041	413		Farmer owned field
SC23107	2014	Sichuan	Rice	*F. asiaticum*	NIV	31.09316	104.94041	413		Farmer owned field
SC23105	2014	Sichuan	Rice	*F. asiaticum*	NIV	31.09316	104.94041	413		Farmer owned field
SC23104	2014	Sichuan	Rice	*F. asiaticum*	NIV	31.09316	104.94041	413		Farmer owned field
SC23103	2014	Sichuan	Rice	*F. asiaticum*	NIV	31.09316	104.94041	413		Farmer owned field
SC23098	2014	Sichuan	Rice	*F. asiaticum*	NIV	31.09316	104.94041	413		Farmer owned field
SC23095	2014	Sichuan	Rice	*F. asiaticum*	NIV	31.09316	104.94041	413		Farmer owned field
SC23093	2014	Sichuan	Rice	*F. asiaticum*	NIV	31.09316	104.94041	413		Farmer owned field
SC23092	2014	Sichuan	Rice	*F. asiaticum*	NIV	31.09316	104.94041	413		Farmer owned field
SC23091-2	2014	Sichuan	Rice	*F. asiaticum*	NIV	31.09316	104.94041	413		Farmer owned field
SC23091	2014	Sichuan	Rice	*F. asiaticum*	NIV	31.09316	104.94041	413		Farmer owned field
SC23088	2014	Sichuan	Rice	*F. asiaticum*	NIV	31.09316	104.94041	413		Farmer owned field
SC23086	2014	Sichuan	Rice	*F. asiaticum*	NIV	31.09316	104.94041	413		Farmer owned field
SC23083	2014	Sichuan	Rice	*F. asiaticum*	NIV	31.09316	104.94041	413		Farmer owned field
SC23079	2014	Sichuan	Rice	*F. asiaticum*	NIV	31.09316	104.94041	413		Farmer owned field
SCST95	2014	Sichuan	Wheat	*F. asiaticum*	NIV	31.121126	105.09804	383	Commercialized variety	Farmer owned field
SCST86	2014	Sichuan	Wheat	*F. asiaticum*	15ADON	31.121126	105.09804	383	Commercialized variety	Farmer owned field
SCST85	2014	Sichuan	Wheat	*F. asiaticum*	NIV	31.121126	105.09804	383	Commercialized variety	Farmer owned field
SCST63-2	2014	Sichuan	Wheat	*F. asiaticum*	NIV	31.121126	105.09804	383	Commercialized variety	Farmer owned field
SCST53	2014	Sichuan	Wheat	*F. asiaticum*	NIV	31.121126	105.09804	383	Commercialized variety	Farmer owned field
SCST5-2	2014	Sichuan	Wheat	*F. asiaticum*	NIV	31.121126	105.09804	383	Commercialized variety	Farmer owned field
SCST44-2	2014	Sichuan	Wheat	*F. asiaticum*	NIV	31.121126	105.09804	383	Commercialized variety	Farmer owned field
171383	2017	Fujian	Rice	*F. asiaticum*	NIV	26.37813	117.866667	112.8		Farmer owned field
171381	2017	Fujian	Rice	*F. asiaticum*	NIV	26.37813	117.866667	112.8		Farmer owned field
171380	2017	Fujian	Rice	*F. asiaticum*	NIV	26.37813	117.866667	112.8		Farmer owned field

171379	2017	Fujian	Rice	*F. asiaticum*	NIV	26.37813	117.866667	112.8	Farmer owned field
171378	2017	Fujian	Rice	*F. asiaticum*	NIV	26.37813	117.866667	112.8	Farmer owned field
171377	2017	Fujian	Rice	*F. asiaticum*	NIV	26.37813	117.866667	112.8	Farmer owned field
171376	2017	Fujian	Rice	*F. asiaticum*	NIV	26.37813	117.866667	112.8	Farmer owned field
171375	2017	Fujian	Rice	*F. asiaticum*	NIV	26.37813	117.866667	112.8	Farmer owned field
171374	2017	Fujian	Rice	*F. asiaticum*	NIV	26.37813	117.866667	112.8	Farmer owned field
171373	2017	Fujian	Rice	*F. asiaticum*	15ADON	26.37813	117.866667	112.8	Farmer owned field
171372	2017	Fujian	Rice	*F. asiaticum*	15ADON	26.37813	117.866667	112.8	Farmer owned field
171371	2017	Fujian	Rice	*F. asiaticum*	15ADON	26.37813	117.866667	112.8	Farmer owned field
171370	2017	Fujian	Rice	*F. asiaticum*	NIV	26.37813	117.866667	112.8	Farmer owned field
171369	2017	Fujian	Rice	*F. asiaticum*	NIV	26.37813	117.866667	112.8	Farmer owned field
171368	2017	Fujian	Rice	*F. asiaticum*	NIV	26.37813	117.866667	112.8	Farmer owned field
171367	2017	Fujian	Rice	*F. asiaticum*	NIV	26.37813	117.866667	112.8	Farmer owned field
171366	2017	Fujian	Rice	*F. asiaticum*	NIV	26.37813	117.866667	112.8	Farmer owned field
171365	2017	Fujian	Rice	*F. asiaticum*	15ADON	26.37813	117.866667	112.8	Farmer owned field
171364	2017	Fujian	Rice	*F. asiaticum*	NIV	26.37813	117.866667	112.8	Farmer owned field
171363	2017	Fujian	Rice	*F. asiaticum*	NIV	26.37813	117.866667	112.8	Farmer owned field
171362	2017	Fujian	Rice	*F. asiaticum*	15ADON	26.37813	117.866667	112.8	Farmer owned field
171361	2017	Fujian	Rice	*F. asiaticum*	NIV	26.37813	117.866667	112.8	Farmer owned field
171360	2017	Fujian	Rice	*F. asiaticum*	NIV	26.37813	117.866667	112.8	Farmer owned field
171359	2017	Fujian	Rice	*F. asiaticum*	NIV	26.37813	117.866667	112.8	Farmer owned field
171318	2017	Fujian	Rice	*F. asiaticum*	NIV	26.842675	117.196507	410.8	Farmer owned field
191317	2017	Fujian	Rice	*F. asiaticum*	NIV	26.842675	117.196507	410.8	Farmer owned field
171316	2017	Fujian	Rice	*F. asiaticum*	NIV	26.842675	117.196507	410.8	Farmer owned field
171315	2017	Fujian	Rice	*F. asiaticum*	NIV	26.842675	117.196507	410.8	Farmer owned field
171314	2017	Fujian	Rice	*F. asiaticum*	NIV	26.842675	117.196507	410.8	Farmer owned field
171313	2017	Fujian	Rice	*F. asiaticum*	NIV	26.842675	117.196507	410.8	Farmer owned field
171312	2017	Fujian	Rice	*F. asiaticum*	NIV	26.842675	117.196507	410.8	Farmer owned field
171311	2017	Fujian	Rice	*F. asiaticum*	NIV	26.842675	117.196507	410.8	Farmer owned field
171310	2017	Fujian	Rice	*F. asiaticum*	NIV	26.842675	117.196507	410.8	Farmer owned field
171309	2017	Fujian	Rice	*F. asiaticum*	NIV	26.842675	117.196507	410.8	Farmer owned field
171308	2017	Fujian	Rice	*F. asiaticum*	15ADON	26.842675	117.196507	410.8	Farmer owned field
171307	2017	Fujian	Rice	*F. asiaticum*	NIV	26.842675	117.196507	410.8	Farmer owned field
171306	2017	Fujian	Rice	*F. asiaticum*	NIV	26.842675	117.196507	410.8	Farmer owned field
171305	2017	Fujian	Rice	*F. asiaticum*	NIV	26.842675	117.196507	410.8	Farmer owned field

171304	2017	Fujian	Rice	*F. asiaticum*	NIV	26.842675	117.196507	410.8		Farmer owned field
171303	2017	Fujian	Rice	*F. asiaticum*	NIV	26.842675	117.196507	410.8		Farmer owned field
171302	2017	Fujian	Rice	*F. asiaticum*	NIV	26.842675	117.196507	410.8		Farmer owned field
171301	2017	Fujian	Rice	*F. asiaticum*	NIV	26.842675	117.196507	410.8		Farmer owned field
171300	2017	Fujian	Rice	*F. asiaticum*	NIV	26.842675	117.196507	410.8		Farmer owned field
171299	2017	Fujian	Rice	*F. asiaticum*	NIV	26.842675	117.196507	410.8		Farmer owned field
171298	2017	Fujian	Rice	*F. asiaticum*	NIV	26.842675	117.196507	410.8		Farmer owned field
171297	2017	Fujian	Rice	*F. asiaticum*	NIV	26.842675	117.196507	410.8		Farmer owned field
171296	2017	Fujian	Rice	*F. asiaticum*	NIV	26.842675	117.196507	410.8		Farmer owned field
171295	2017	Fujian	Rice	*F. asiaticum*	NIV	26.842675	117.196507	410.8		Farmer owned field
171294	2017	Fujian	Rice	*F. asiaticum*	NIV	26.842675	117.196507	410.8		Farmer owned field
171293	2017	Fujian	Rice	*F. asiaticum*	NIV	26.842675	117.196507	410.8		Farmer owned field
171292	2017	Fujian	Rice	*F. asiaticum*	NIV	26.842675	117.196507	410.8		Farmer owned field
171291	2017	Fujian	Rice	*F. asiaticum*	NIV	26.842675	117.196507	410.8		Farmer owned field
171290	2017	Fujian	Rice	*F. asiaticum*	NIV	26.842675	117.196507	410.8		Farmer owned field
171289	2017	Fujian	Rice	*F. asiaticum*	NIV	26.842675	117.196507	410.8		Farmer owned field
171288	2017	Fujian	Rice	*F. asiaticum*	NIV	26.842675	117.196507	410.8		Farmer owned field
171287	2017	Fujian	Rice	*F. asiaticum*	NIV	26.842675	117.196507	410.8		Farmer owned field
171286	2017	Fujian	Rice	*F. asiaticum*	NIV	26.842675	117.196507	410.8		Farmer owned field
171285	2017	Fujian	Rice	*F. asiaticum*	NIV	26.842675	117.196507	410.8		Farmer owned field
171284	2017	Fujian	Rice	*F. asiaticum*	NIV	26.842675	117.196507	410.8		Farmer owned field
171283	2017	Fujian	Rice	*F. asiaticum*	NIV	26.842675	117.196507	410.8		Farmer owned field
171282	2017	Fujian	Rice	*F. asiaticum*	NIV	26.842675	117.196507	410.8		Farmer owned field
171281	2017	Fujian	Rice	*F. asiaticum*	NIV	26.842675	117.196507	410.8		Farmer owned field
171280	2017	Fujian	Rice	*F. asiaticum*	NIV	26.842675	117.165943	327		Farmer owned field
171279	2017	Fujian	Rice	*F. asiaticum*	NIV	26.842675	117.165943	327		Farmer owned field
171278	2017	Fujian	Rice	*F. asiaticum*	15ADON	26.842675	117.165943	327		Farmer owned field
171277	2017	Fujian	Rice	*F. asiaticum*	NIV	26.842675	117.165943	327		Farmer owned field
171276	2017	Fujian	Rice	*F. asiaticum*	NIV	26.842675	117.165943	327		Farmer owned field
171275	2017	Fujian	Rice	*F. asiaticum*	NIV	26.842675	117.165943	327		Farmer owned field
171274	2017	Fujian	Rice	*F. asiaticum*	NIV	26.842675	117.165943	327		Farmer owned field
171273	2017	Fujian	Rice	*F. asiaticum*	NIV	26.842675	117.165943	327		Farmer owned field
171272	2017	Fujian	Rice	*F. asiaticum*	15ADON	26.842675	117.165943	327		Farmer owned field
171271	2017	Fujian	Rice	*F. asiaticum*	NIV	26.842675	117.165943	327		Farmer owned field
171270	2017	Fujian	Rice	*F. asiaticum*	NIV	26.842675	117.165943	327		Farmer owned field

171269	2017	Fujian	Rice	*F. asiaticum*	NIV	26.842675	117.165943	327	Farmer owned field
171268	2017	Fujian	Rice	*F. asiaticum*	NIV	26.842675	117.165943	327	Farmer owned field
171267	2017	Fujian	Rice	*F. asiaticum*	NIV	26.842675	117.165943	327	Farmer owned field
171266	2017	Fujian	Rice	*F. asiaticum*	NIV	26.842675	117.165943	327	Farmer owned field
171265	2017	Fujian	Rice	*F. asiaticum*	NIV	26.842675	117.165943	327	Farmer owned field
171264	2017	Fujian	Rice	*F. asiaticum*	NIV	26.842675	117.165943	327	Farmer owned field
171263	2017	Fujian	Rice	*F. asiaticum*	NIV	26.842675	117.165943	327	Farmer owned field
171262	2017	Fujian	Rice	*F. asiaticum*	NIV	26.842675	117.165943	327	Farmer owned field
171261	2017	Fujian	Rice	*F. asiaticum*	NIV	26.842675	117.165943	327	Farmer owned field
171260	2017	Fujian	Rice	*F. asiaticum*	NIV	26.842675	117.165943	327	Farmer owned field
171259	2017	Fujian	Rice	*F. asiaticum*	NIV	26.842675	117.165943	327	Farmer owned field
171258	2017	Fujian	Rice	*F. asiaticum*	NIV	26.842675	117.165943	327	Farmer owned field
171257	2017	Fujian	Rice	*F. asiaticum*	NIV	26.842675	117.165943	327	Farmer owned field
171256	2017	Fujian	Rice	*F. asiaticum*	NIV	26.842675	117.165943	327	Farmer owned field
171255	2017	Fujian	Rice	*F. asiaticum*	NIV	26.842675	117.165943	327	Farmer owned field
171254	2017	Fujian	Rice	*F. asiaticum*	NIV	26.842675	117.165943	327	Farmer owned field
171253	2017	Fujian	Rice	*F. asiaticum*	NIV	26.842675	117.165943	327	Farmer owned field
171252	2017	Fujian	Rice	*F. asiaticum*	NIV	26.842675	117.165943	327	Farmer owned field
171251	2017	Fujian	Rice	*F. asiaticum*	NIV	26.842675	117.165943	327	Farmer owned field
171250	2017	Fujian	Rice	*F. asiaticum*	NIV	26.842675	117.165943	327	Farmer owned field
171249	2017	Fujian	Rice	*F. asiaticum*	NIV	26.842675	117.165943	327	Farmer owned field
171248	2017	Fujian	Rice	*F. asiaticum*	NIV	26.842675	117.165943	327	Farmer owned field
171247	2017	Fujian	Rice	*F. asiaticum*	NIV	26.842675	117.165943	327	Farmer owned field
171246	2017	Fujian	Rice	*F. asiaticum*	NIV	26.842675	117.165943	327	Farmer owned field
171245	2017	Fujian	Rice	*F. asiaticum*	NIV	26.842675	117.165943	327	Farmer owned field
171244	2017	Fujian	Rice	*F. asiaticum*	NIV	26.842675	117.165943	327	Farmer owned field
171358	2017	Fujian	Rice	*F. asiaticum*	NIV	26.947053	117.227145	288	Farmer owned field
171357	2017	Fujian	Rice	*F. asiaticum*	NIV	26.947053	117.227145	288	Farmer owned field
171355	2017	Fujian	Rice	*F. asiaticum*	NIV	26.947053	117.227145	288	Farmer owned field
171354	2017	Fujian	Rice	*F. asiaticum*	NIV	26.947053	117.227145	288	Farmer owned field
171353	2017	Fujian	Rice	*F. asiaticum*	NIV	26.947053	117.227145	288	Farmer owned field
171352	2017	Fujian	Rice	*F. asiaticum*	NIV	26.947053	117.227145	288	Farmer owned field
171351	2017	Fujian	Rice	*F. asiaticum*	NIV	26.947053	117.227145	288	Farmer owned field
171350	2017	Fujian	Rice	*F. asiaticum*	NIV	26.947053	117.227145	288	Farmer owned field
171349	2017	Fujian	Rice	*F. asiaticum*	NIV	26.947053	117.227145	288	Farmer owned field

171348	2017	Fujian	Rice	*F. asiaticum*	NIV	26.947053	117.227145	288		Farmer owned field
171347	2017	Fujian	Rice	*F. asiaticum*	NIV	26.947053	117.227145	288		Farmer owned field
171346	2017	Fujian	Rice	*F. asiaticum*	NIV	26.947053	117.227145	288		Farmer owned field
171345	2017	Fujian	Rice	*F. asiaticum*	NIV	26.947053	117.227145	288		Farmer owned field
171344	2017	Fujian	Rice	*F. asiaticum*	NIV	26.947053	117.227145	288		Farmer owned field
171343	2017	Fujian	Rice	*F. asiaticum*	NIV	26.947053	117.227145	288		Farmer owned field
182654	2018	Jiangsu	Wheat	*F. asiaticum*	3ADON	33.28812202	120.166226	1.783241	Commercialized variety	Farmer owned field
182653	2018	Jiangsu	Wheat	*F. asiaticum*	3ADON	33.28812202	120.166226	1.783241	Commercialized variety	Farmer owned field
182652	2018	Jiangsu	Wheat	*F. asiaticum*	3ADON	33.28812202	120.166226	1.783241	Commercialized variety	Farmer owned field
182651	2018	Jiangsu	Wheat	*F. asiaticum*	3ADON	33.28812202	120.166226	1.783241	Commercialized variety	Farmer owned field
182650	2018	Jiangsu	Wheat	*F. asiaticum*	3ADON	33.33480301	119.988529	13.119117	Commercialized variety	Farmer owned field
182649	2018	Jiangsu	Wheat	*F. asiaticum*	NIV	33.33480301	119.988529	13.119117	Commercialized variety	Farmer owned field
182648	2018	Jiangsu	Wheat	*F. asiaticum*	3ADON	33.33480301	119.988529	13.119117	Commercialized variety	Farmer owned field
182647	2018	Jiangsu	Wheat	*F. asiaticum*	NIV	33.33480301	119.988529	13.119117	Commercialized variety	Farmer owned field
182646	2018	Jiangsu	Wheat	*F. asiaticum*	3ADON	33.33480301	119.988529	13.119117	Commercialized variety	Farmer owned field
182645	2018	Jiangsu	Wheat	*F. asiaticum*	3ADON	33.33480301	119.988529	13.119117	Commercialized variety	Farmer owned field
183584	2018	Jiangsu	Wheat	*F. asiaticum*	3ADON	33.40865403	119.748567	8.101352	Commercialized variety	Farmer owned field
183583	2018	Jiangsu	Wheat	*F. asiaticum*	3ADON	33.40865403	119.748567	8.101352	Commercialized variety	Farmer owned field
183582	2018	Jiangsu	Wheat	*F. asiaticum*	3ADON	33.40865403	119.748567	8.101352	Commercialized variety	Farmer owned field
182524	2018	Jiangsu	Wheat	*F. asiaticum*	3ADON	33.42313704	118.16273	37.0839	Commercialized variety	Farmer owned field
182523	2018	Jiangsu	Wheat	*F. asiaticum*	3ADON	33.42313704	118.16273	37.0839	Commercialized variety	Farmer owned field
182521	2018	Jiangsu	Wheat	*F. asiaticum*	3ADON	33.42313704	118.16273	37.0839	Commercialized variety	Farmer owned field
182520	2018	Jiangsu	Wheat	*F. asiaticum*	3ADON	33.42313704	118.16273	37.0839	Commercialized variety	Farmer owned field
182519	2018	Jiangsu	Wheat	*F. asiaticum*	3ADON	33.42313704	118.16273	37.0839	Commercialized variety	Farmer owned field
182518	2018	Jiangsu	Wheat	*F. asiaticum*	3ADON	33.42313704	118.16273	37.0839	Commercialized variety	Farmer owned field
182517	2018	Jiangsu	Wheat	*F. asiaticum*	3ADON	33.42313704	118.16273	37.0839	Commercialized variety	Farmer owned field
182516	2018	Jiangsu	Wheat	*F. asiaticum*	3ADON	33.42313704	118.16273	37.0839	Commercialized variety	Farmer owned field
182515	2018	Jiangsu	Wheat	*F. asiaticum*	3ADON	33.42313704	118.16273	37.0839	Commercialized variety	Farmer owned field
192934	2019	Jiangsu	Wheat	*F. asiaticum*	3ADON	33.44719101	119.344707	11.811169	Commercialized variety	Farmer owned field
192933	2019	Jiangsu	Wheat	*F. asiaticum*	3ADON	33.44719101	119.344707	11.811169	Commercialized variety	Farmer owned field
192932	2019	Jiangsu	Wheat	*F. asiaticum*	3ADON	33.44719101	119.344707	11.811169	Commercialized variety	Farmer owned field
192931	2019	Jiangsu	Wheat	*F. asiaticum*	3ADON	33.44719101	119.344707	11.811169	Commercialized variety	Farmer owned field
192930	2019	Jiangsu	Wheat	*F. asiaticum*	3ADON	33.44719101	119.344707	11.811169	Commercialized variety	Farmer owned field
192929	2019	Jiangsu	Wheat	*F. asiaticum*	3ADON	33.44719101	119.344707	11.811169	Commercialized variety	Farmer owned field
192928	2019	Jiangsu	Wheat	*F. asiaticum*	3ADON	33.44719101	119.344707	11.811169	Commercialized variety	Farmer owned field

192927	2019	Jiangsu	Wheat	*F. asiaticum*	3ADON	33.44719101	119.344707	11.811169	Commercialized variety	Farmer owned field
182538	2018	Jiangsu	Wheat	*F. asiaticum*	3ADON	33.44719101	119.344707	11.811169	Commercialized variety	Farmer owned field
182537	2018	Jiangsu	Wheat	*F. asiaticum*	NIV	33.44719101	119.344707	11.811169	Commercialized variety	Farmer owned field
182536	2018	Jiangsu	Wheat	*F. asiaticum*	3ADON	33.44719101	119.344707	11.811169	Commercialized variety	Farmer owned field
182535	2018	Jiangsu	Wheat	*F. asiaticum*	NIV	33.44719101	119.344707	11.811169	Commercialized variety	Farmer owned field
192843	2019	Jiangsu	Wheat	*F. asiaticum*	3ADON	33.54995197	118.178253	99.290443	Commercialized variety	Farmer owned field
192842	2019	Jiangsu	Wheat	*F. asiaticum*	3ADON	33.54995197	118.178253	99.290443	Commercialized variety	Farmer owned field
192841	2019	Jiangsu	Wheat	*F. asiaticum*	3ADON	33.54995197	118.178253	99.290443	Commercialized variety	Farmer owned field
192840	2019	Jiangsu	Wheat	*F. asiaticum*	3ADON	33.54995197	118.178253	99.290443	Commercialized variety	Farmer owned field
192839	2019	Jiangsu	Wheat	*F. asiaticum*	3ADON	33.54995197	118.178253	99.290443	Commercialized variety	Farmer owned field
192838	2019	Jiangsu	Wheat	*F. asiaticum*	3ADON	33.54995197	118.178253	99.290443	Commercialized variety	Farmer owned field
192837	2019	Jiangsu	Wheat	*F. asiaticum*	3ADON	33.54995197	118.178253	99.290443	Commercialized variety	Farmer owned field
192836	2019	Jiangsu	Wheat	*F. asiaticum*	3ADON	33.54995197	118.178253	99.290443	Commercialized variety	Farmer owned field
192835	2019	Jiangsu	Wheat	*F. asiaticum*	3ADON	33.54995197	118.178253	99.290443	Commercialized variety	Farmer owned field
192833	2019	Jiangsu	Wheat	*F. asiaticum*	3ADON	33.54995197	118.178253	99.290443	Commercialized variety	Farmer owned field
192832	2019	Jiangsu	Wheat	*F. asiaticum*	3ADON	33.54995197	118.178253	99.290443	Commercialized variety	Farmer owned field
192830	2019	Jiangsu	Wheat	*F. asiaticum*	3ADON	33.54995197	118.178253	99.290443	Commercialized variety	Farmer owned field
192829	2019	Jiangsu	Wheat	*F. asiaticum*	3ADON	33.54995197	118.178253	99.290443	Commercialized variety	Farmer owned field
192828	2019	Jiangsu	Wheat	*F. asiaticum*	3ADON	33.54995197	118.178253	99.290443	Commercialized variety	Farmer owned field
192827	2019	Jiangsu	Wheat	*F. asiaticum*	3ADON	33.54995197	118.178253	99.290443	Commercialized variety	Farmer owned field
192825	2019	Jiangsu	Wheat	*F. asiaticum*	3ADON	33.54995197	118.178253	99.290443	Commercialized variety	Farmer owned field
192824	2019	Jiangsu	Wheat	*F. asiaticum*	NIV	33.54995197	118.178253	99.290443	Commercialized variety	Farmer owned field
192823	2019	Jiangsu	Wheat	*F. asiaticum*	3ADON	33.54995197	118.178253	99.290443	Commercialized variety	Farmer owned field
192822	2019	Jiangsu	Wheat	*F. asiaticum*	3ADON	33.54995197	118.178253	99.290443	Commercialized variety	Farmer owned field
192821	2019	Jiangsu	Wheat	*F. asiaticum*	3ADON	33.55537296	120.206748	87.003654	Commercialized variety	Farmer owned field
192820	2019	Jiangsu	Wheat	*F. asiaticum*	3ADON	33.55537296	120.206748	87.003654	Commercialized variety	Farmer owned field
192819	2019	Jiangsu	Wheat	*F. asiaticum*	3ADON	33.55537296	120.206748	87.003654	Commercialized variety	Farmer owned field
192818	2019	Jiangsu	Wheat	*F. asiaticum*	3ADON	33.55537296	120.206748	87.003654	Commercialized variety	Farmer owned field
192817	2019	Jiangsu	Wheat	*F. asiaticum*	3ADON	33.55537296	120.206748	87.003654	Commercialized variety	Farmer owned field
192816	2019	Jiangsu	Wheat	*F. asiaticum*	3ADON	33.55537296	120.206748	87.003654	Commercialized variety	Farmer owned field
192815	2019	Jiangsu	Wheat	*F. asiaticum*	3ADON	33.55537296	120.206748	87.003654	Commercialized variety	Farmer owned field
192812	2019	Jiangsu	Wheat	*F. asiaticum*	3ADON	33.55537296	120.206748	87.003654	Commercialized variety	Farmer owned field
192811	2019	Jiangsu	Wheat	*F. asiaticum*	3ADON	33.55537296	120.206748	87.003654	Commercialized variety	Farmer owned field
192810	2019	Jiangsu	Wheat	*F. asiaticum*	3ADON	33.55537296	120.206748	87.003654	Commercialized variety	Farmer owned field
182642	2018	Jiangsu	Wheat	*F. asiaticum*	3ADON	33.61166599	118.191465	47.431026	Commercialized variety	Farmer owned field

182641	2018	Jiangsu	Wheat	*F. asiaticum*	3ADON	33.61166599	118.191465	47.431026	Commercialized variety	Farmer owned field
182639	2018	Jiangsu	Wheat	*F. asiaticum*	3ADON	33.61166599	118.191465	47.431026	Commercialized variety	Farmer owned field
182638	2018	Jiangsu	Wheat	*F. asiaticum*	3ADON	33.61166599	118.191465	47.431026	Commercialized variety	Farmer owned field
182637	2018	Jiangsu	Wheat	*F. asiaticum*	3ADON	33.61166599	118.191465	47.431026	Commercialized variety	Farmer owned field
182636	2018	Jiangsu	Wheat	*F. asiaticum*	3ADON	33.61166599	118.191465	47.431026	Commercialized variety	Farmer owned field
182635	2018	Jiangsu	Wheat	*F. asiaticum*	3ADON	33.61166599	118.191465	47.431026	Commercialized variety	Farmer owned field
182634	2018	Jiangsu	Wheat	*F. asiaticum*	3ADON	33.61166599	118.191465	47.431026	Commercialized variety	Farmer owned field
182633	2018	Jiangsu	Wheat	*F. asiaticum*	3ADON	33.61166599	118.191465	47.431026	Commercialized variety	Farmer owned field
182632	2018	Jiangsu	Wheat	*F. asiaticum*	3ADON	33.61166599	118.191465	47.431026	Commercialized variety	Farmer owned field
182631	2018	Jiangsu	Wheat	*F. asiaticum*	3ADON	33.61166599	118.191465	47.431026	Commercialized variety	Farmer owned field
191099	2019	Jiangsu	Wheat	*F. asiaticum*	3ADON	33.63022001	118.726104	91.936867	Commercialized variety	Farmer owned field
191098	2019	Jiangsu	Wheat	*F. asiaticum*	3ADON	33.63022001	118.726104	91.936867	Commercialized variety	Farmer owned field
191097	2019	Jiangsu	Wheat	*F. asiaticum*	3ADON	33.63022001	118.726104	91.936867	Commercialized variety	Farmer owned field
191095	2019	Jiangsu	Wheat	*F. asiaticum*	NIV	33.63022001	118.726104	91.936867	Commercialized variety	Farmer owned field
191094	2019	Jiangsu	Wheat	*F. asiaticum*	3ADON	33.63022001	118.726104	91.936867	Commercialized variety	Farmer owned field
191093	2019	Jiangsu	Wheat	*F. asiaticum*	3ADON	33.63022001	118.726104	91.936867	Commercialized variety	Farmer owned field
191091	2019	Jiangsu	Wheat	*F. asiaticum*	3ADON	33.63022001	118.726104	91.936867	Commercialized variety	Farmer owned field
191090	2019	Jiangsu	Wheat	*F. asiaticum*	3ADON	33.63022001	118.726104	91.936867	Commercialized variety	Farmer owned field
191088	2019	Jiangsu	Wheat	*F. asiaticum*	NIV	33.63022001	118.726104	91.936867	Commercialized variety	Farmer owned field
191087	2019	Jiangsu	Wheat	*F. asiaticum*	NIV	33.63022001	118.726104	91.936867	Commercialized variety	Farmer owned field
191086	2019	Jiangsu	Wheat	*F. asiaticum*	3ADON	33.63022001	118.726104	91.936867	Commercialized variety	Farmer owned field
191085	2019	Jiangsu	Wheat	*F. asiaticum*	3ADON	33.63022001	118.726104	91.936867	Commercialized variety	Farmer owned field
191084	2019	Jiangsu	Wheat	*F. asiaticum*	NIV	33.63022001	118.726104	91.936867	Commercialized variety	Farmer owned field
191083	2019	Jiangsu	Wheat	*F. asiaticum*	3ADON	33.63022001	118.726104	91.936867	Commercialized variety	Farmer owned field
191082	2019	Jiangsu	Wheat	*F. asiaticum*	3ADON	33.63022001	118.726104	91.936867	Commercialized variety	Farmer owned field
191081	2019	Jiangsu	Wheat	*F. asiaticum*	3ADON	33.63022001	118.726104	91.936867	Commercialized variety	Farmer owned field
191080	2019	Jiangsu	Wheat	*F. asiaticum*	3ADON	33.63022001	118.726104	91.936867	Commercialized variety	Farmer owned field
191079	2019	Jiangsu	Wheat	*F. asiaticum*	3ADON	33.63022001	118.726104	91.936867	Commercialized variety	Farmer owned field
191078	2019	Jiangsu	Wheat	*F. asiaticum*	3ADON	33.63022001	118.726104	91.936867	Commercialized variety	Farmer owned field
191077	2019	Jiangsu	Wheat	*F. asiaticum*	3ADON	33.63022001	118.726104	91.936867	Commercialized variety	Farmer owned field
192897	2019	Jiangsu	Wheat	*F. asiaticum*	3ADON	33.68832696	118.974556	50.532059	Commercialized variety	Farmer owned field
192895	2019	Jiangsu	Wheat	*F. asiaticum*	3ADON	33.68832696	118.974556	50.532059	Commercialized variety	Farmer owned field
192894	2019	Jiangsu	Wheat	*F. asiaticum*	3ADON	33.68832696	118.974556	50.532059	Commercialized variety	Farmer owned field
192893	2019	Jiangsu	Wheat	*F. asiaticum*	3ADON	33.68832696	118.974556	50.532059	Commercialized variety	Farmer owned field
192892	2019	Jiangsu	Wheat	*F. asiaticum*	3ADON	33.68832696	118.974556	50.532059	Commercialized variety	Farmer owned field

192891	2019	Jiangsu	Wheat	*F. asiaticum*	3ADON	33.68832696	118.974556	50.532059	Commercialized variety	Farmer owned field
192889	2019	Jiangsu	Wheat	*F. asiaticum*	3ADON	33.68832696	118.974556	50.532059	Commercialized variety	Farmer owned field
192887	2019	Jiangsu	Wheat	*F. asiaticum*	3ADON	33.68832696	118.974556	50.532059	Commercialized variety	Farmer owned field
192886	2019	Jiangsu	Wheat	*F. asiaticum*	3ADON	33.68832696	118.974556	50.532059	Commercialized variety	Farmer owned field
192885	2019	Jiangsu	Wheat	*F. asiaticum*	3ADON	33.68832696	118.974556	50.532059	Commercialized variety	Farmer owned field
192882	2019	Jiangsu	Wheat	*F. asiaticum*	3ADON	33.68832696	118.974556	50.532059	Commercialized variety	Farmer owned field
182534	2018	Jiangsu	Wheat	*F. asiaticum*	3ADON	33.76653297	118.356242	44.383041	Commercialized variety	Farmer owned field
182533	2018	Jiangsu	Wheat	*F. asiaticum*	3ADON	33.76653297	118.356242	44.383041	Commercialized variety	Farmer owned field
182532	2018	Jiangsu	Wheat	*F. asiaticum*	3ADON	33.76653297	118.356242	44.383041	Commercialized variety	Farmer owned field
182531	2018	Jiangsu	Wheat	*F. asiaticum*	3ADON	33.76653297	118.356242	44.383041	Commercialized variety	Farmer owned field
182530	2018	Jiangsu	Wheat	*F. asiaticum*	NIV	33.76653297	118.356242	44.383041	Commercialized variety	Farmer owned field
182529	2018	Jiangsu	Wheat	*F. asiaticum*	NIV	33.76653297	118.356242	44.383041	Commercialized variety	Farmer owned field
182527	2018	Jiangsu	Wheat	*F. asiaticum*	3ADON	33.76653297	118.356242	44.383041	Commercialized variety	Farmer owned field
182526	2018	Jiangsu	Wheat	*F. asiaticum*	3ADON	33.76653297	118.356242	44.383041	Commercialized variety	Farmer owned field
182525	2018	Jiangsu	Wheat	*F. asiaticum*	3ADON	33.76653297	118.356242	44.383041	Commercialized variety	Farmer owned field
182109	2018	Jiangsu	Wheat	*F. asiaticum*	3ADON	33.861191	120.402394	NA	Commercialized variety	Farmer owned field
182108	2018	Jiangsu	Wheat	*F. asiaticum*	3ADON	33.861191	120.402394	NA	Commercialized variety	Farmer owned field
182107	2018	Jiangsu	Wheat	*F. asiaticum*	NIV	33.861191	120.402394	NA	Commercialized variety	Farmer owned field
182106	2018	Jiangsu	Wheat	*F. asiaticum*	3ADON	33.861191	120.402394	NA	Commercialized variety	Farmer owned field
182105	2018	Jiangsu	Wheat	*F. asiaticum*	3ADON	33.861191	120.402394	NA	Commercialized variety	Farmer owned field
182104	2018	Jiangsu	Wheat	*F. asiaticum*	3ADON	33.861191	120.402394	NA	Commercialized variety	Farmer owned field
182103	2018	Jiangsu	Wheat	*F. asiaticum*	3ADON	33.861191	120.402394	NA	Commercialized variety	Farmer owned field
182102	2018	Jiangsu	Wheat	*F. asiaticum*	3ADON	33.861191	120.402394	NA	Commercialized variety	Farmer owned field
182101	2018	Jiangsu	Wheat	*F. asiaticum*	NIV	33.861191	120.402394	NA	Commercialized variety	Farmer owned field
182099	2018	Jiangsu	Wheat	*F. asiaticum*	3ADON	33.861191	120.402394	NA	Commercialized variety	Farmer owned field
180188	2018	Jiangsu	Wheat	*F. asiaticum*	3ADON	NA	NA	NA	Commercialized variety	Commercial farm
180189	2018	Jiangsu	Wheat	*F. asiaticum*	3ADON	NA	NA	NA	Commercialized variety	Commercial farm
HBXG161	2014	Hubei	Wheat	*F. asiaticum*	NIV	30.892518	113.933865	24	Commercialized variety	Commercial farm
HBXG158	2014	Hubei	Wheat	*F. asiaticum*	3ADON	30.892518	113.933865	24	Commercialized variety	Commercial farm
190537	2019	Anhui	Wheat	*F. asiaticum*	15ADON	32.83316899	117.299769	36.723686	Commercialized variety	Farmer owned field
190533	2019	Anhui	Wheat	*F. asiaticum*	3ADON	32.83316899	117.299769	36.723686	Commercialized variety	Farmer owned field
190865	2019	Anhui	Wheat	*F. asiaticum*	3ADON	32.84705202	117.592806	21.046717	Commercialized variety	Farmer owned field
190863	2019	Anhui	Wheat	*F. asiaticum*	NIV	32.84705202	117.592806	21.046717	Commercialized variety	Farmer owned field
190862	2019	Anhui	Wheat	*F. asiaticum*	3ADON	32.84705202	117.592806	21.046717	Commercialized variety	Farmer owned field
190861	2019	Anhui	Wheat	*F. asiaticum*	3ADON	32.84705202	117.592806	21.046717	Commercialized variety	Farmer owned field

190860	2019	Anhui	Wheat	*F. asiaticum*	3ADON	32.84705202	117.592806	21.046717	Commercialized variety	Farmer owned field
190859	2019	Anhui	Wheat	*F. asiaticum*	NIV	32.84705202	117.592806	21.046717	Commercialized variety	Farmer owned field
190858	2019	Anhui	Wheat	*F. asiaticum*	NIV	32.84705202	117.592806	21.046717	Commercialized variety	Farmer owned field
190857	2019	Anhui	Wheat	*F. asiaticum*	3ADON	32.84705202	117.592806	21.046717	Commercialized variety	Farmer owned field
190856	2019	Anhui	Wheat	*F. asiaticum*	NIV	32.84705202	117.592806	21.046717	Commercialized variety	Farmer owned field
190855	2019	Anhui	Wheat	*F. asiaticum*	3ADON	32.84705202	117.592806	21.046717	Commercialized variety	Farmer owned field
190854	2019	Anhui	Wheat	*F. asiaticum*	3ADON	32.84705202	117.592806	21.046717	Commercialized variety	Farmer owned field
190853	2019	Anhui	Wheat	*F. asiaticum*	3ADON	32.84705202	117.592806	21.046717	Commercialized variety	Farmer owned field
190852	2019	Anhui	Wheat	*F. asiaticum*	3ADON	32.84705202	117.592806	21.046717	Commercialized variety	Farmer owned field
190851	2019	Anhui	Wheat	*F. asiaticum*	NIV	32.84705202	117.592806	21.046717	Commercialized variety	Farmer owned field
190850	2019	Anhui	Wheat	*F. asiaticum*	3ADON	32.84705202	117.592806	21.046717	Commercialized variety	Farmer owned field
190849	2019	Anhui	Wheat	*F. asiaticum*	3ADON	32.84705202	117.592806	21.046717	Commercialized variety	Farmer owned field
190847	2019	Anhui	Wheat	*F. asiaticum*	3ADON	32.84705202	117.592806	21.046717	Commercialized variety	Farmer owned field
190846	2019	Anhui	Wheat	*F. asiaticum*	3ADON	32.84705202	117.592806	21.046717	Commercialized variety	Farmer owned field
190845	2019	Anhui	Wheat	*F. asiaticum*	NIV	32.84705202	117.592806	21.046717	Commercialized variety	Farmer owned field
190843	2019	Anhui	Wheat	*F. asiaticum*	3ADON	32.84705202	117.592806	21.046717	Commercialized variety	Farmer owned field
190842	2019	Anhui	Wheat	*F. asiaticum*	3ADON	32.84705202	117.592806	21.046717	Commercialized variety	Farmer owned field
190841	2019	Anhui	Wheat	*F. asiaticum*	NIV	32.84705202	117.592806	21.046717	Commercialized variety	Farmer owned field
182480	2018	Anhui	Wheat	*F. asiaticum*	3ADON	32.95701798	115.547312	95.442558	Commercialized variety	Farmer owned field
182476	2018	Anhui	Wheat	*F. asiaticum*	3ADON	32.95701798	115.547312	95.442558	Commercialized variety	Farmer owned field
190838	2019	Anhui	Wheat	*F. asiaticum*	3ADON	33.04581603	115.893875	44.553513	Commercialized variety	Farmer owned field
190818	2019	Anhui	Wheat	*F. asiaticum*	3ADON	33.04581603	115.893875	44.553513	Commercialized variety	Farmer owned field
190817	2019	Anhui	Wheat	*F. asiaticum*	3ADON	33.04581603	115.893875	44.553513	Commercialized variety	Farmer owned field
190816	2019	Anhui	Wheat	*F. asiaticum*	3ADON	33.04581603	115.893875	44.553513	Commercialized variety	Farmer owned field
190815	2019	Anhui	Wheat	*F. asiaticum*	3ADON	33.04581603	115.893875	44.553513	Commercialized variety	Farmer owned field
190868	2019	Anhui	Wheat	*F. asiaticum*	3ADON	33.10346299	117.86869	28.886684	Commercialized variety	Farmer owned field
190811	2019	Anhui	Wheat	*F. asiaticum*	3ADON	33.153468	117.110122	35.081619	Commercialized variety	Farmer owned field
190809	2019	Anhui	Wheat	*F. asiaticum*	3ADON	33.153468	117.110122	35.081619	Commercialized variety	Farmer owned field
190808	2019	Anhui	Wheat	*F. asiaticum*	3ADON	33.153468	117.110122	35.081619	Commercialized variety	Farmer owned field
190806	2019	Anhui	Wheat	*F. asiaticum*	3ADON	33.153468	117.110122	35.081619	Commercialized variety	Farmer owned field
190805	2019	Anhui	Wheat	*F. asiaticum*	NIV	33.153468	117.110122	35.081619	Commercialized variety	Farmer owned field
190796	2019	Anhui	Wheat	*F. asiaticum*	3ADON	33.153468	117.110122	35.081619	Commercialized variety	Farmer owned field
HBXG142	2014	Hubei	Wheat	*F. asiaticum*	3ADON	30.892518	113.933865	24	Commercialized variety	Commercial farm
HBXG141	2014	Hubei	Wheat	*F. asiaticum*	NIV	30.892518	113.933865	24	Commercialized variety	Commercial farm
190795	2019	Anhui	Wheat	*F. asiaticum*	15ADON	33.153468	117.110122	35.081619	Commercialized variety	Farmer owned field

HBXG139	2014	Hubei	Wheat	*F. asiaticum*	3ADON	30.892518	113.933865	24	Commercialized variety	Commercial farm
HBXG136	2014	Hubei	Wheat	*F. asiaticum*	15ADON	30.892518	113.933865	24	Commercialized variety	Commercial farm
HBXG127	2014	Hubei	Wheat	*F. asiaticum*	3ADON	30.892518	113.933865	24	Commercialized variety	Commercial farm
HBXG115	2014	Hubei	Wheat	*F. asiaticum*	3ADON	30.892518	113.933865	24	Commercialized variety	Commercial farm
HBXG101	2014	Hubei	Wheat	*F. asiaticum*	3ADON	30.892518	113.933865	24	Commercialized variety	Commercial farm
HB14107	2014	Hubei	Rice	*F. asiaticum*	3ADON	30.892518	113.933865	24		Commercial farm
190792	2019	Anhui	Wheat	*F. asiaticum*	NIV	33.153468	117.110122	35.081619	Commercialized variety	Farmer owned field
HB14002	2014	Hubei	Rice	*F. asiaticum*	3ADON	30.892518	113.933865	24		Commercial farm
14107-2	2014	Hubei	Rice	*F. asiaticum*	3ADON	30.892518	113.933865	24		Commercial farm
190791	2019	Anhui	Wheat	*F. asiaticum*	3ADON	33.153468	117.110122	35.081619	Commercialized variety	Farmer owned field
SCST24-2	2014	Sichuan	Wheat	*F. asiaticum*	NIV	31.121126	105.09804	383	Commercialized variety	Farmer owned field
SCST24-1	2014	Sichuan	Wheat	*F. asiaticum*	NIV	31.121126	105.09804	383	Commercialized variety	Farmer owned field
SCST21	2014	Sichuan	Wheat	*F. asiaticum*	NIV	31.121126	105.09804	383	Commercialized variety	Farmer owned field
SCST19-2	2014	Sichuan	Wheat	*F. asiaticum*	NIV	31.121126	105.09804	383	Commercialized variety	Farmer owned field
193282	2019	Hunan	Rice	*F. asiaticum*	NIV	25.95137998	112.495782	271.019287		Farmer owned field
193281	2019	Hunan	Rice	*F. asiaticum*	NIV	25.95137998	112.495782	271.019287		Farmer owned field
193280	2019	Hunan	Rice	*F. asiaticum*	NIV	25.95137998	112.495782	271.019287		Farmer owned field
193278	2019	Hunan	Rice	*F. asiaticum*	NIV	25.95137998	112.495782	271.019287		Farmer owned field
193277	2019	Hunan	Rice	*F. asiaticum*	NIV	25.95137998	112.495782	271.019287		Farmer owned field
193353	2019	Hunan	Rice	*F. asiaticum*	NIV	26.40011103	112.4549	131.49115		Farmer owned field
193352	2019	Hunan	Rice	*F. asiaticum*	NIV	26.40011103	112.4549	131.49115		Farmer owned field
193350	2019	Hunan	Rice	*F. asiaticum*	NIV	26.40011103	112.4549	131.49115		Farmer owned field
193349	2019	Hunan	Rice	*F. asiaticum*	NIV	26.40011103	112.4549	131.49115		Farmer owned field
193348	2019	Hunan	Rice	*F. asiaticum*	NIV	26.40011103	112.4549	131.49115		Farmer owned field
193347	2019	Hunan	Rice	*F. asiaticum*	NIV	26.40011103	112.4549	131.49115		Farmer owned field
193346	2019	Hunan	Rice	*F. asiaticum*	NIV	26.40011103	112.4549	131.49115		Farmer owned field
193345	2019	Hunan	Rice	*F. asiaticum*	3ADON	26.40011103	112.4549	131.49115		Farmer owned field
193344	2019	Hunan	Rice	*F. asiaticum*	15ADON	26.40011103	112.4549	131.49115		Farmer owned field
193343	2019	Hunan	Rice	*F. asiaticum*	3ADON	26.40011103	112.4549	131.49115		Farmer owned field
193342	2019	Hunan	Rice	*F. asiaticum*	NIV	26.40011103	112.4549	131.49115		Farmer owned field
193340	2019	Hunan	Rice	*F. asiaticum*	NIV	26.40011103	112.4549	131.49115		Farmer owned field
193339	2019	Hunan	Rice	*F. asiaticum*	NIV	26.40011103	112.4549	131.49115		Farmer owned field
193338	2019	Hunan	Rice	*F. asiaticum*	NIV	26.40011103	112.4549	131.49115		Farmer owned field
193337	2019	Hunan	Rice	*F. asiaticum*	15ADON	26.40011103	112.4549	131.49115		Farmer owned field
193336	2019	Hunan	Rice	*F. asiaticum*	NIV	26.40011103	112.4549	131.49115		Farmer owned field

193335	2019	Hunan	Rice	*F. asiaticum*	NIV	26.40011103	112.4549	131.49115	Farmer owned field
193334	2019	Hunan	Rice	*F. asiaticum*	NIV	26.40011103	112.4549	131.49115	Farmer owned field
193332	2019	Hunan	Rice	*F. asiaticum*	NIV	26.40011103	112.4549	131.49115	Farmer owned field
193331	2019	Hunan	Rice	*F. asiaticum*	NIV	26.40011103	112.4549	131.49115	Farmer owned field
193330	2019	Hunan	Rice	*F. asiaticum*	NIV	26.40011103	112.4549	131.49115	Farmer owned field
193329	2019	Hunan	Rice	*F. asiaticum*	NIV	26.40011103	112.4549	131.49115	Farmer owned field
193328	2019	Hunan	Rice	*F. asiaticum*	NIV	26.86069602	112.458668	115.918556	Farmer owned field
193327	2019	Hunan	Rice	*F. asiaticum*	NIV	26.86069602	112.458668	115.918556	Farmer owned field
193326	2019	Hunan	Rice	*F. asiaticum*	NIV	26.86069602	112.458668	115.918556	Farmer owned field
193325	2019	Hunan	Rice	*F. asiaticum*	NIV	26.86069602	112.458668	115.918556	Farmer owned field
193324	2019	Hunan	Rice	*F. asiaticum*	NIV	26.86069602	112.458668	115.918556	Farmer owned field
193323	2019	Hunan	Rice	*F. asiaticum*	NIV	26.86069602	112.458668	115.918556	Farmer owned field
193322	2019	Hunan	Rice	*F. asiaticum*	NIV	26.86069602	112.458668	115.918556	Farmer owned field
193321	2019	Hunan	Rice	*F. asiaticum*	NIV	26.86069602	112.458668	115.918556	Farmer owned field
193320	2019	Hunan	Rice	*F. asiaticum*	NIV	26.86069602	112.458668	115.918556	Farmer owned field
193319	2019	Hunan	Rice	*F. asiaticum*	NIV	26.86069602	112.458668	115.918556	Farmer owned field
193317	2019	Hunan	Rice	*F. asiaticum*	NIV	26.86069602	112.458668	115.918556	Farmer owned field
193316	2019	Hunan	Rice	*F. asiaticum*	NIV	26.86069602	112.458668	115.918556	Farmer owned field
193315	2019	Hunan	Rice	*F. asiaticum*	3ADON	26.86069602	112.458668	115.918556	Farmer owned field
193314	2019	Hunan	Rice	*F. asiaticum*	NIV	26.86069602	112.458668	115.918556	Farmer owned field
193313	2019	Hunan	Rice	*F. asiaticum*	NIV	26.86069602	112.458668	115.918556	Farmer owned field
193312	2019	Hunan	Rice	*F. asiaticum*	NIV	26.86069602	112.458668	115.918556	Farmer owned field
193311	2019	Hunan	Rice	*F. asiaticum*	NIV	26.86069602	112.458668	115.918556	Farmer owned field
193310	2019	Hunan	Rice	*F. asiaticum*	NIV	26.86069602	112.458668	115.918556	Farmer owned field
193417	2019	Hunan	Rice	*F. asiaticum*	NIV	27.30642501	112.564631	146.099136	Farmer owned field
193416	2019	Hunan	Rice	*F. asiaticum*	NIV	27.30642501	112.564631	146.099136	Farmer owned field
193415	2019	Hunan	Rice	*F. asiaticum*	NIV	27.30642501	112.564631	146.099136	Farmer owned field
193414	2019	Hunan	Rice	*F. asiaticum*	NIV	27.30642501	112.564631	146.099136	Farmer owned field
193411	2019	Hunan	Rice	*F. asiaticum*	NIV	27.30642501	112.564631	146.099136	Farmer owned field
193410	2019	Hunan	Rice	*F. asiaticum*	NIV	27.30642501	112.564631	146.099136	Farmer owned field
193409	2019	Hunan	Rice	*F. asiaticum*	NIV	27.30642501	112.564631	146.099136	Farmer owned field
193408	2019	Hunan	Rice	*F. asiaticum*	15ADON	27.30642501	112.564631	146.099136	Farmer owned field
193407	2019	Hunan	Rice	*F. asiaticum*	15ADON	27.30642501	112.564631	146.099136	Farmer owned field
193406	2019	Hunan	Rice	*F. asiaticum*	3ADON	27.30642501	112.564631	146.099136	Farmer owned field
193404	2019	Hunan	Rice	*F. asiaticum*	15ADON	27.30642501	112.564631	146.099136	Farmer owned field

193403	2019	Hunan	Rice	*F. asiaticum*	15ADON	27.30642501	112.564631	146.099136		Farmer owned field
193402	2019	Hunan	Rice	*F. asiaticum*	15ADON	27.30642501	112.564631	146.099136		Farmer owned field
193400	2019	Hunan	Rice	*F. asiaticum*	15ADON	27.30642501	112.564631	146.099136		Farmer owned field
193399	2019	Hunan	Rice	*F. asiaticum*	15ADON	27.30642501	112.564631	146.099136		Farmer owned field
193398	2019	Hunan	Rice	*F. asiaticum*	15ADON	27.30642501	112.564631	146.099136		Farmer owned field
193397	2019	Hunan	Rice	*F. asiaticum*	15ADON	27.30642501	112.564631	146.099136		Farmer owned field
193396	2019	Hunan	Rice	*F. asiaticum*	15ADON	27.30642501	112.564631	146.099136		Farmer owned field
193395	2019	Hunan	Rice	*F. asiaticum*	15ADON	27.30642501	112.564631	146.099136		Farmer owned field
193394	2019	Hunan	Rice	*F. asiaticum*	3ADON	27.30642501	112.564631	146.099136		Farmer owned field
193393	2019	Hunan	Rice	*F. asiaticum*	15ADON	27.30642501	112.564631	146.099136		Farmer owned field
193392	2019	Hunan	Rice	*F. asiaticum*	3ADON	27.30642501	112.564631	146.099136		Farmer owned field
193391	2019	Hunan	Rice	*F. asiaticum*	15ADON	27.30642501	112.564631	146.099136		Farmer owned field
193390	2019	Hunan	Rice	*F. asiaticum*	15ADON	27.30642501	112.564631	146.099136		Farmer owned field
193389	2019	Hunan	Rice	*F. asiaticum*	3ADON	27.30642501	112.564631	146.099136		Farmer owned field
193387	2019	Hunan	Rice	*F. asiaticum*	15ADON	27.30642501	112.564631	146.099136		Farmer owned field
174254	2017	Hunan	Rice	*F. asiaticum*	NIV	27.409341	109.658426	251		Farmer owned field
174253	2017	Hunan	Rice	*F. asiaticum*	NIV	27.409341	109.658426	251		Farmer owned field
174252	2017	Hunan	Rice	*F. asiaticum*	NIV	27.409341	109.658426	251		Farmer owned field
174251	2017	Hunan	Rice	*F. asiaticum*	NIV	27.409341	109.658426	251		Farmer owned field
174250	2017	Hunan	Rice	*F. asiaticum*	NIV	27.409341	109.658426	251		Farmer owned field
174249	2017	Hunan	Rice	*F. asiaticum*	NIV	27.409341	109.658426	251		Farmer owned field
174248	2017	Hunan	Rice	*F. asiaticum*	NIV	27.409341	109.658426	251		Farmer owned field
174247	2017	Hunan	Rice	*F. asiaticum*	NIV	27.409341	109.658426	251		Farmer owned field
174246	2017	Hunan	Rice	*F. asiaticum*	NIV	27.409341	109.658426	251		Farmer owned field
174245	2017	Hunan	Rice	*F. asiaticum*	NIV	27.409341	109.658426	251		Farmer owned field
174244	2017	Hunan	Rice	*F. asiaticum*	NIV	27.409341	109.658426	251		Farmer owned field
174243	2017	Hunan	Rice	*F. asiaticum*	NIV	27.409341	109.658426	251		Farmer owned field
174242	2017	Hunan	Rice	*F. asiaticum*	NIV	27.409341	109.658426	251		Farmer owned field
174241	2017	Hunan	Rice	*F. asiaticum*	NIV	27.409341	109.658426	251		Farmer owned field
174240	2017	Hunan	Rice	*F. asiaticum*	NIV	27.409341	109.658426	251		Farmer owned field
174238	2017	Hunan	Rice	*F. asiaticum*	NIV	27.409341	109.658426	251		Farmer owned field
174237	2017	Hunan	Rice	*F. asiaticum*	NIV	27.409341	109.658426	251		Farmer owned field
174236	2017	Hunan	Rice	*F. asiaticum*	NIV	27.409341	109.658426	251		Farmer owned field
174235	2017	Hunan	Rice	*F. asiaticum*	NIV	27.409341	109.658426	251		Farmer owned field
174234	2017	Hunan	Rice	*F. asiaticum*	NIV	27.409341	109.658426	251		Farmer owned field

174233	2017	Hunan	Rice	*F. asiaticum*	NIV	27.409341	109.658426	251	Farmer owned field
174232	2017	Hunan	Rice	*F. asiaticum*	NIV	27.409341	109.658426	251	Farmer owned field
174231	2017	Hunan	Rice	*F. asiaticum*	NIV	27.409341	109.658426	251	Farmer owned field
171119	2017	Hunan	Rice	*F. asiaticum*	NIV	27.431337	112.6122	NA	Farmer owned field
171118	2017	Hunan	Rice	*F. asiaticum*	NIV	27.431337	112.6122	NA	Farmer owned field
171117	2017	Hunan	Rice	*F. asiaticum*	NIV	27.431337	112.6122	NA	Farmer owned field
171116	2017	Hunan	Rice	*F. asiaticum*	3ADON	27.431337	112.6122	NA	Farmer owned field
171115	2017	Hunan	Rice	*F. asiaticum*	3ADON	27.431337	112.6122	NA	Farmer owned field
171114	2017	Hunan	Rice	*F. asiaticum*	NIV	27.431337	112.6122	NA	Farmer owned field
171113	2017	Hunan	Rice	*F. asiaticum*	NIV	27.431337	112.6122	NA	Farmer owned field
171112	2017	Hunan	Rice	*F. asiaticum*	3ADON	27.431337	112.6122	NA	Farmer owned field
171111	2017	Hunan	Rice	*F. asiaticum*	3ADON	27.431337	112.6122	NA	Farmer owned field
171110	2017	Hunan	Rice	*F. asiaticum*	NIV	27.431337	112.6122	NA	Farmer owned field
171109	2017	Hunan	Rice	*F. asiaticum*	NIV	27.431337	112.6122	NA	Farmer owned field
171108	2017	Hunan	Rice	*F. asiaticum*	3ADON	27.431337	112.6122	NA	Farmer owned field
171107	2017	Hunan	Rice	*F. asiaticum*	NIV	27.431337	112.6122	NA	Farmer owned field
171106	2017	Hunan	Rice	*F. asiaticum*	NIV	27.431337	112.6122	NA	Farmer owned field
171105	2017	Hunan	Rice	*F. asiaticum*	NIV	27.431337	112.6122	NA	Farmer owned field
194000	2019	Hunan	Rice	*F. asiaticum*	NIV	27.52331899	112.595131	82.949181	Farmer owned field
193999	2019	Hunan	Rice	*F. asiaticum*	3ADON	27.52331899	112.595131	82.949181	Farmer owned field
193998	2019	Hunan	Rice	*F. asiaticum*	NIV	27.52331899	112.595131	82.949181	Farmer owned field
193997	2019	Hunan	Rice	*F. asiaticum*	NIV	27.52331899	112.595131	82.949181	Farmer owned field
193996	2019	Hunan	Rice	*F. asiaticum*	NIV	27.52331899	112.595131	82.949181	Farmer owned field
193994	2019	Hunan	Rice	*F. asiaticum*	NIV	27.52331899	112.595131	82.949181	Farmer owned field
193993	2019	Hunan	Rice	*F. asiaticum*	NIV	27.52331899	112.595131	82.949181	Farmer owned field
193992	2019	Hunan	Rice	*F. asiaticum*	NIV	27.52331899	112.595131	82.949181	Farmer owned field
193991	2019	Hunan	Rice	*F. asiaticum*	3ADON	27.52331899	112.595131	82.949181	Farmer owned field
193990	2019	Hunan	Rice	*F. asiaticum*	NIV	27.52331899	112.595131	82.949181	Farmer owned field
193989	2019	Hunan	Rice	*F. asiaticum*	NIV	27.52331899	112.595131	82.949181	Farmer owned field
193988	2019	Hunan	Rice	*F. asiaticum*	NIV	27.52331899	112.595131	82.949181	Farmer owned field
193986	2019	Hunan	Rice	*F. asiaticum*	3ADON	27.52331899	112.595131	82.949181	Farmer owned field
193985	2019	Hunan	Rice	*F. asiaticum*	NIV	27.52331899	112.595131	82.949181	Farmer owned field
193984	2019	Hunan	Rice	*F. asiaticum*	NIV	27.52331899	112.595131	82.949181	Farmer owned field
193982	2019	Hunan	Rice	*F. asiaticum*	3ADON	27.52331899	112.595131	82.949181	Farmer owned field
193981	2019	Hunan	Rice	*F. asiaticum*	3ADON	27.52331899	112.595131	82.949181	Farmer owned field

193980	2019	Hunan	Rice	*F. asiaticum*	NIV	27.52331899	112.595131	82.949181		Farmer owned field
193979	2019	Hunan	Rice	*F. asiaticum*	3ADON	27.52331899	112.595131	82.949181		Farmer owned field
193978	2019	Hunan	Rice	*F. asiaticum*	NIV	27.52331899	112.595131	82.949181		Farmer owned field
171104	2017	Hunan	Rice	*F. asiaticum*	NIV	27.52331899	112.595131	82.949181		Farmer owned field
190790	2019	Anhui	Wheat	*F. asiaticum*	NIV	33.153468	117.110122	35.081619	Commercialized variety	Farmer owned field
190785	2019	Anhui	Wheat	*F. asiaticum*	3ADON	33.153468	117.110122	35.081619	Commercialized variety	Farmer owned field
190784	2019	Anhui	Wheat	*F. asiaticum*	NIV	33.153468	117.110122	35.081619	Commercialized variety	Farmer owned field
190782	2019	Anhui	Wheat	*F. asiaticum*	3ADON	33.153468	117.110122	35.081619	Commercialized variety	Farmer owned field
190780	2019	Anhui	Wheat	*F. asiaticum*	3ADON	33.153468	117.110122	35.081619	Commercialized variety	Farmer owned field
190776	2019	Anhui	Wheat	*F. asiaticum*	3ADON	33.153468	117.110122	35.081619	Commercialized variety	Farmer owned field
190775	2019	Anhui	Wheat	*F. asiaticum*	3ADON	33.153468	117.110122	35.081619	Commercialized variety	Farmer owned field
190774	2019	Anhui	Wheat	*F. asiaticum*	3ADON	33.153468	117.110122	35.081619	Commercialized variety	Farmer owned field
190773	2019	Anhui	Wheat	*F. asiaticum*	NIV	33.153468	117.110122	35.081619	Commercialized variety	Farmer owned field
191337	2019	Anhui	Wheat	*F. asiaticum*	3ADON	33.188818	116.321199	43.806122	Commercialized variety	Farmer owned field
191334	2019	Anhui	Wheat	*F. asiaticum*	3ADON	33.188818	116.321199	43.806122	Commercialized variety	Farmer owned field
191332	2019	Anhui	Wheat	*F. asiaticum*	3ADON	33.188818	116.321199	43.806122	Commercialized variety	Farmer owned field
191330	2019	Anhui	Wheat	*F. asiaticum*	3ADON	33.188818	116.321199	43.806122	Commercialized variety	Farmer owned field
191329	2019	Anhui	Wheat	*F. asiaticum*	3ADON	33.188818	116.321199	43.806122	Commercialized variety	Farmer owned field
194601	2019	Anhui	Wheat	*F. asiaticum*	3ADON	33.2755	116.14149	NA	Commercialized variety	Farmer owned field
194600	2019	Anhui	Wheat	*F. asiaticum*	3ADON	33.2755	116.14149	NA	Commercialized variety	Farmer owned field
194599	2019	Anhui	Wheat	*F. asiaticum*	3ADON	33.2755	116.14149	NA	Commercialized variety	Farmer owned field
194598	2019	Anhui	Wheat	*F. asiaticum*	3ADON	33.2755	116.14149	NA	Commercialized variety	Farmer owned field
194596	2019	Anhui	Wheat	*F. asiaticum*	3ADON	33.2755	116.14149	NA	Commercialized variety	Farmer owned field
194595	2019	Anhui	Wheat	*F. asiaticum*	3ADON	33.2755	116.14149	NA	Commercialized variety	Farmer owned field
194594	2019	Anhui	Wheat	*F. asiaticum*	3ADON	33.2755	116.14149	NA	Commercialized variety	Farmer owned field
194592	2019	Anhui	Wheat	*F. asiaticum*	3ADON	33.2755	116.14149	NA	Commercialized variety	Farmer owned field
194590	2019	Anhui	Wheat	*F. asiaticum*	3ADON	33.2755	116.14149	NA	Commercialized variety	Farmer owned field
171103	2017	Hunan	Rice	*F. asiaticum*	NIV	27.52331899	112.595131	82.949181		Farmer owned field
171102	2017	Hunan	Rice	*F. asiaticum*	NIV	27.52331899	112.595131	82.949181		Farmer owned field
171101	2017	Hunan	Rice	*F. asiaticum*	NIV	27.52331899	112.595131	82.949181		Farmer owned field
171100	2017	Hunan	Rice	*F. asiaticum*	NIV	27.52331899	112.595131	82.949181		Farmer owned field
171099	2017	Hunan	Rice	*F. asiaticum*	NIV	27.52331899	112.595131	82.949181		Farmer owned field
171098	2017	Hunan	Rice	*F. asiaticum*	NIV	27.52331899	112.595131	82.949181		Farmer owned field
171097	2017	Hunan	Rice	*F. asiaticum*	NIV	27.52331899	112.595131	82.949181		Farmer owned field
171096	2017	Hunan	Rice	*F. asiaticum*	NIV	27.52331899	112.595131	82.949181		Farmer owned field

171095	2017	Hunan	Rice	*F. asiaticum*	NIV	27.52331899	112.595131	82.949181		Farmer owned field
171094	2017	Hunan	Rice	*F. asiaticum*	NIV	27.52331899	112.595131	82.949181		Farmer owned field
171093	2017	Hunan	Rice	*F. asiaticum*	NIV	27.52331899	112.595131	82.949181		Farmer owned field
171092	2017	Hunan	Rice	*F. asiaticum*	NIV	27.52331899	112.595131	82.949181		Farmer owned field
171091	2017	Hunan	Rice	*F. asiaticum*	NIV	27.52331899	112.595131	82.949181		Farmer owned field
171090	2017	Hunan	Rice	*F. asiaticum*	NIV	27.52331899	112.595131	82.949181		Farmer owned field
171089	2017	Hunan	Rice	*F. asiaticum*	NIV	27.52331899	112.595131	82.949181		Farmer owned field
171088	2017	Hunan	Rice	*F. asiaticum*	NIV	27.52331899	112.595131	82.949181		Farmer owned field
171087	2017	Hunan	Rice	*F. asiaticum*	NIV	27.52331899	112.595131	82.949181		Farmer owned field
171085	2017	Hunan	Rice	*F. asiaticum*	NIV	27.52331899	112.595131	82.949181		Farmer owned field
171084	2017	Hunan	Rice	*F. asiaticum*	NIV	27.52331899	112.595131	82.949181		Farmer owned field
194174	2019	Hunan	Rice	*F. asiaticum*	NIV	27.699346	112.522798	85.075966		Farmer owned field
180190	2018	Jiangsu	Wheat	*F. asiaticum*	3ADON	NA	NA	NA	Commercialized variety	Commercial farm
180192	2018	Jiangsu	Wheat	*F. asiaticum*	3ADON	NA	NA	NA	Commercialized variety	Commercial farm
180193	2018	Jiangsu	Wheat	*F. asiaticum*	NIV	NA	NA	NA	Commercialized variety	Commercial farm
180194	2018	Jiangsu	Wheat	*F. asiaticum*	3ADON	NA	NA	NA	Commercialized variety	Commercial farm
180195	2018	Jiangsu	Wheat	*F. asiaticum*	3ADON	NA	NA	NA	Commercialized variety	Commercial farm
180197	2018	Jiangsu	Wheat	*F. asiaticum*	3ADON	NA	NA	NA	Commercialized variety	Commercial farm
180198	2018	Jiangsu	Wheat	*F. asiaticum*	NIV	NA	NA	NA	Commercialized variety	Commercial farm
180199	2018	Jiangsu	Wheat	*F. asiaticum*	3ADON	NA	NA	NA	Commercialized variety	Commercial farm
180200	2018	Jiangsu	Wheat	*F. asiaticum*	3ADON	NA	NA	NA	Commercialized variety	Commercial farm
180202	2018	Jiangsu	Wheat	*F. asiaticum*	3ADON	NA	NA	NA	Commercialized variety	Commercial farm
180203	2018	Jiangsu	Wheat	*F. asiaticum*	3ADON	NA	NA	NA	Commercialized variety	Commercial farm
180204	2018	Jiangsu	Wheat	*F. asiaticum*	3ADON	NA	NA	NA	Commercialized variety	Commercial farm
180205	2018	Jiangsu	Wheat	*F. asiaticum*	3ADON	NA	NA	NA	Commercialized variety	Commercial farm
180207	2018	Jiangsu	Wheat	*F. asiaticum*	3ADON	NA	NA	NA	Commercialized variety	Commercial farm
180209	2018	Jiangsu	Wheat	*F. asiaticum*	3ADON	NA	NA	NA	Commercialized variety	Commercial farm
180210	2018	Jiangsu	Wheat	*F. asiaticum*	3ADON	NA	NA	NA	Commercialized variety	Commercial farm
180211	2018	Jiangsu	Wheat	*F. asiaticum*	3ADON	NA	NA	NA	Commercialized variety	Commercial farm
180212	2018	Jiangsu	Wheat	*F. asiaticum*	3ADON	NA	NA	NA	Commercialized variety	Commercial farm
194173	2019	Hunan	Rice	*F. asiaticum*	NIV	27.699346	112.522798	85.075966		Farmer owned field
194172	2019	Hunan	Rice	*F. asiaticum*	NIV	27.699346	112.522798	85.075966		Farmer owned field
194171	2019	Hunan	Rice	*F. asiaticum*	NIV	27.699346	112.522798	85.075966		Farmer owned field
194170	2019	Hunan	Rice	*F. asiaticum*	NIV	27.699346	112.522798	85.075966		Farmer owned field
194169	2019	Hunan	Rice	*F. asiaticum*	NIV	27.699346	112.522798	85.075966		Farmer owned field

194168	2019	Hunan	Rice	*F. asiaticum*	NIV	27.699346	112.522798	85.075966	Farmer owned field
194167	2019	Hunan	Rice	*F. asiaticum*	NIV	27.699346	112.522798	85.075966	Farmer owned field
194166	2019	Hunan	Rice	*F. asiaticum*	3ADON	27.699346	112.522798	85.075966	Farmer owned field
194165	2019	Hunan	Rice	*F. asiaticum*	NIV	27.699346	112.522798	85.075966	Farmer owned field
194163	2019	Hunan	Rice	*F. asiaticum*	NIV	27.699346	112.522798	85.075966	Farmer owned field
194162	2019	Hunan	Rice	*F. asiaticum*	3ADON	27.699346	112.522798	85.075966	Farmer owned field
194161	2019	Hunan	Rice	*F. asiaticum*	NIV	27.699346	112.522798	85.075966	Farmer owned field
194159	2019	Hunan	Rice	*F. asiaticum*	NIV	27.699346	112.522798	85.075966	Farmer owned field
194158	2019	Hunan	Rice	*F. asiaticum*	3ADON	27.699346	112.522798	85.075966	Farmer owned field
194157	2019	Hunan	Rice	*F. asiaticum*	NIV	27.699346	112.522798	85.075966	Farmer owned field
194156	2019	Hunan	Rice	*F. asiaticum*	3ADON	27.699346	112.522798	85.075966	Farmer owned field
194154	2019	Hunan	Rice	*F. asiaticum*	15ADON	27.699346	112.522798	85.075966	Farmer owned field
194153	2019	Hunan	Rice	*F. asiaticum*	NIV	27.699346	112.522798	85.075966	Farmer owned field
194151	2019	Hunan	Rice	*F. asiaticum*	NIV	27.699346	112.522798	85.075966	Farmer owned field
194150	2019	Hunan	Rice	*F. asiaticum*	NIV	27.699346	112.522798	85.075966	Farmer owned field
194149	2019	Hunan	Rice	*F. asiaticum*	NIV	27.699346	112.522798	85.075966	Farmer owned field
171083	2017	Hunan	Rice	*F. asiaticum*	NIV	27.699346	112.522798	101	Farmer owned field
171082	2017	Hunan	Rice	*F. asiaticum*	3ADON	27.699346	112.522798	101	Farmer owned field
171081	2017	Hunan	Rice	*F. asiaticum*	NIV	27.699346	112.522798	101	Farmer owned field
171080	2017	Hunan	Rice	*F. asiaticum*	NIV	27.699346	112.522798	101	Farmer owned field
171079	2017	Hunan	Rice	*F. asiaticum*	NIV	27.699346	112.522798	101	Farmer owned field
171078	2017	Hunan	Rice	*F. asiaticum*	3ADON	27.699346	112.522798	101	Farmer owned field
171077	2017	Hunan	Rice	*F. asiaticum*	NIV	27.699346	112.522798	101	Farmer owned field
171076	2017	Hunan	Rice	*F. asiaticum*	NIV	27.699346	112.522798	101	Farmer owned field
171075	2017	Hunan	Rice	*F. asiaticum*	3ADON	27.699346	112.522798	101	Farmer owned field
171074	2017	Hunan	Rice	*F. asiaticum*	NIV	27.699346	112.522798	101	Farmer owned field
171073	2017	Hunan	Rice	*F. asiaticum*	NIV	27.699346	112.522798	101	Farmer owned field
171072	2017	Hunan	Rice	*F. asiaticum*	NIV	27.699346	112.522798	101	Farmer owned field
171071	2017	Hunan	Rice	*F. asiaticum*	NIV	27.699346	112.522798	101	Farmer owned field
171070	2017	Hunan	Rice	*F. asiaticum*	NIV	27.699346	112.522798	101	Farmer owned field
171069	2017	Hunan	Rice	*F. asiaticum*	15ADON	27.699346	112.522798	101	Farmer owned field
171068	2017	Hunan	Rice	*F. asiaticum*	NIV	27.699346	112.522798	101	Farmer owned field
171067	2017	Hunan	Rice	*F. asiaticum*	3ADON	27.699346	112.522798	101	Farmer owned field
171066	2017	Hunan	Rice	*F. asiaticum*	NIV	27.699346	112.522798	101	Farmer owned field
171065	2017	Hunan	Rice	*F. asiaticum*	NIV	27.699346	112.522798	101	Farmer owned field

171064	2017	Hunan	Rice	*F. asiaticum*	NIV	27.699346	112.522798	101	Farmer owned field
171063	2017	Hunan	Rice	*F. asiaticum*	NIV	27.699346	112.522798	101	Farmer owned field
171062	2017	Hunan	Rice	*F. asiaticum*	3ADON	27.699346	112.522798	101	Farmer owned field
171061	2017	Hunan	Rice	*F. asiaticum*	NIV	27.699346	112.522798	101	Farmer owned field
171060	2017	Hunan	Rice	*F. asiaticum*	NIV	27.699346	112.522798	101	Farmer owned field
171059	2017	Hunan	Rice	*F. asiaticum*	NIV	27.699346	112.522798	101	Farmer owned field
194127	2019	Hunan	Rice	*F. asiaticum*	NIV	27.92500301	112.552357	73.673798	Farmer owned field
194126	2019	Hunan	Rice	*F. asiaticum*	NIV	27.92500301	112.552357	73.673798	Farmer owned field
194125	2019	Hunan	Rice	*F. asiaticum*	NIV	27.92500301	112.552357	73.673798	Farmer owned field
194124	2019	Hunan	Rice	*F. asiaticum*	NIV	27.92500301	112.552357	73.673798	Farmer owned field
194123	2019	Hunan	Rice	*F. asiaticum*	3ADON	27.92500301	112.552357	73.673798	Farmer owned field
194122	2019	Hunan	Rice	*F. asiaticum*	NIV	27.92500301	112.552357	73.673798	Farmer owned field
194121	2019	Hunan	Rice	*F. asiaticum*	NIV	27.92500301	112.552357	73.673798	Farmer owned field
194120	2019	Hunan	Rice	*F. asiaticum*	NIV	27.92500301	112.552357	73.673798	Farmer owned field
194119	2019	Hunan	Rice	*F. asiaticum*	NIV	27.92500301	112.552357	73.673798	Farmer owned field
194118	2019	Hunan	Rice	*F. asiaticum*	NIV	27.92500301	112.552357	73.673798	Farmer owned field
194117	2019	Hunan	Rice	*F. asiaticum*	NIV	27.92500301	112.552357	73.673798	Farmer owned field
194116	2019	Hunan	Rice	*F. asiaticum*	3ADON	27.92500301	112.552357	73.673798	Farmer owned field
194115	2019	Hunan	Rice	*F. asiaticum*	NIV	27.92500301	112.552357	73.673798	Farmer owned field
194114	2019	Hunan	Rice	*F. asiaticum*	15ADON	27.92500301	112.552357	73.673798	Farmer owned field
194113	2019	Hunan	Rice	*F. asiaticum*	NIV	27.92500301	112.552357	73.673798	Farmer owned field
194112	2019	Hunan	Rice	*F. asiaticum*	NIV	27.92500301	112.552357	73.673798	Farmer owned field
194111	2019	Hunan	Rice	*F. asiaticum*	NIV	27.92500301	112.552357	73.673798	Farmer owned field
194110	2019	Hunan	Rice	*F. asiaticum*	NIV	27.92500301	112.552357	73.673798	Farmer owned field
194109	2019	Hunan	Rice	*F. asiaticum*	NIV	27.92500301	112.552357	73.673798	Farmer owned field
194108	2019	Hunan	Rice	*F. asiaticum*	NIV	27.92500301	112.552357	73.673798	Farmer owned field
194107	2019	Hunan	Rice	*F. asiaticum*	NIV	27.92500301	112.552357	73.673798	Farmer owned field
194106	2019	Hunan	Rice	*F. asiaticum*	NIV	27.92500301	112.552357	73.673798	Farmer owned field
194105	2019	Hunan	Rice	*F. asiaticum*	NIV	27.92500301	112.552357	73.673798	Farmer owned field
194104	2019	Hunan	Rice	*F. asiaticum*	3ADON	27.92500301	112.552357	73.673798	Farmer owned field
194103	2019	Hunan	Rice	*F. asiaticum*	NIV	27.92500301	112.552357	73.673798	Farmer owned field
171058	2017	Hunan	Rice	*F. asiaticum*	NIV	27.92500301	112.552357	75.3	Farmer owned field
171057	2017	Hunan	Rice	*F. asiaticum*	NIV	27.92500301	112.552357	75.3	Farmer owned field
171056	2017	Hunan	Rice	*F. asiaticum*	NIV	27.92500301	112.552357	75.3	Farmer owned field
171055	2017	Hunan	Rice	*F. asiaticum*	NIV	27.92500301	112.552357	75.3	Farmer owned field

171054	2017	Hunan	Rice	*F. asiaticum*	NIV	27.92500301	112.552357	75.3		Farmer owned field
171053	2017	Hunan	Rice	*F. asiaticum*	NIV	27.92500301	112.552357	75.3		Farmer owned field
171052	2017	Hunan	Rice	*F. asiaticum*	NIV	27.92500301	112.552357	75.3		Farmer owned field
171051	2017	Hunan	Rice	*F. asiaticum*	3ADON	27.92500301	112.552357	75.3		Farmer owned field
171050	2017	Hunan	Rice	*F. asiaticum*	NIV	27.92500301	112.552357	75.3		Farmer owned field
171049	2017	Hunan	Rice	*F. asiaticum*	3ADON	27.92500301	112.552357	75.3		Farmer owned field
171048	2017	Hunan	Rice	*F. asiaticum*	NIV	27.92500301	112.552357	75.3		Farmer owned field
171047	2017	Hunan	Rice	*F. asiaticum*	3ADON	27.92500301	112.552357	75.3		Farmer owned field
171046	2017	Hunan	Rice	*F. asiaticum*	3ADON	27.92500301	112.552357	75.3		Farmer owned field
171045	2017	Hunan	Rice	*F. asiaticum*	NIV	27.92500301	112.552357	75.3		Farmer owned field
171044	2017	Hunan	Rice	*F. asiaticum*	NIV	27.92500301	112.552357	75.3		Farmer owned field
171043	2017	Hunan	Rice	*F. asiaticum*	NIV	27.92500301	112.552357	75.3		Farmer owned field
171042	2017	Hunan	Rice	*F. asiaticum*	NIV	27.92500301	112.552357	75.3		Farmer owned field
171041	2017	Hunan	Rice	*F. asiaticum*	NIV	27.92500301	112.552357	75.3		Farmer owned field
171040	2017	Hunan	Rice	*F. asiaticum*	NIV	27.92500301	112.552357	75.3		Farmer owned field
171039	2017	Hunan	Rice	*F. asiaticum*	NIV	27.92500301	112.552357	75.3		Farmer owned field
171038	2017	Hunan	Rice	*F. asiaticum*	NIV	27.92500301	112.552357	75.3		Farmer owned field
171037	2017	Hunan	Rice	*F. asiaticum*	NIV	27.92500301	112.552357	75.3		Farmer owned field
171036	2017	Hunan	Rice	*F. asiaticum*	3ADON	27.92500301	112.552357	75.3		Farmer owned field
171035	2017	Hunan	Rice	*F. asiaticum*	3ADON	27.92500301	112.552357	75.3		Farmer owned field
171034	2017	Hunan	Rice	*F. asiaticum*	NIV	27.92500301	112.552357	75.3		Farmer owned field
171033	2017	Hunan	Rice	*F. asiaticum*	NIV	27.92500301	112.552357	75.3		Farmer owned field
171032	2017	Hunan	Rice	*F. asiaticum*	3ADON	27.92500301	112.552357	75.3		Farmer owned field
171031	2017	Hunan	Rice	*F. asiaticum*	NIV	27.92500301	112.552357	75.3		Farmer owned field
SCST131-2	2014	Sichuan	Wheat	*F. asiaticum*	3ADON	31.121126	105.09804	383	Commercialized variety	Farmer owned field
SCST122-2	2014	Sichuan	Wheat	*F. asiaticum*	3ADON	31.121126	105.09804	383	Commercialized variety	Farmer owned field
140026	2014	Sichuan	Wheat	*F. asiaticum*	NIV	31.121126	105.09804	383	Commercialized variety	Farmer owned field
140027	2014	Sichuan	Wheat	*F. asiaticum*	NIV	31.121126	105.09804	383	Commercialized variety	Farmer owned field
180943	2018	Sichuan	Wheat	*F. asiaticum*	NIV	31.13249601	105.82188	496.201172	Commercialized variety	Farmer owned field
193780	2019	Jiangxi	Rice	*F. asiaticum*	NIV	25.95091998	115.484893	130.256805		Farmer owned field
193779	2019	Jiangxi	Rice	*F. asiaticum*	15ADON	25.95091998	115.484893	130.256805		Farmer owned field
193778	2019	Jiangxi	Rice	*F. asiaticum*	NIV	25.95091998	115.484893	130.256805		Farmer owned field
193777	2019	Jiangxi	Rice	*F. asiaticum*	15ADON	25.95091998	115.484893	130.256805		Farmer owned field
193774	2019	Jiangxi	Rice	*F. asiaticum*	15ADON	25.95091998	115.484893	130.256805		Farmer owned field
193773	2019	Jiangxi	Rice	*F. asiaticum*	15ADON	25.95091998	115.484893	130.256805		Farmer owned field

193772	2019	Jiangxi	Rice	*F. asiaticum*	NIV	25.95091998	115.484893	130.256805		Farmer owned field
193771	2019	Jiangxi	Rice	*F. asiaticum*	15ADON	25.95091998	115.484893	130.256805		Farmer owned field
193770	2019	Jiangxi	Rice	*F. asiaticum*	NIV	25.95091998	115.484893	130.256805		Farmer owned field
193769	2019	Jiangxi	Rice	*F. asiaticum*	15ADON	25.95091998	115.484893	130.256805		Farmer owned field
193768	2019	Jiangxi	Rice	*F. asiaticum*	NIV	25.95091998	115.484893	130.256805		Farmer owned field
193767	2019	Jiangxi	Rice	*F. asiaticum*	NIV	25.95091998	115.484893	130.256805		Farmer owned field
193766	2019	Jiangxi	Rice	*F. asiaticum*	NIV	25.95091998	115.484893	130.256805		Farmer owned field
193764	2019	Jiangxi	Rice	*F. asiaticum*	NIV	25.95091998	115.484893	130.256805		Farmer owned field
193763	2019	Jiangxi	Rice	*F. asiaticum*	NIV	25.95091998	115.484893	130.256805		Farmer owned field
193762	2019	Jiangxi	Rice	*F. asiaticum*	NIV	25.95091998	115.484893	130.256805		Farmer owned field
193761	2019	Jiangxi	Rice	*F. asiaticum*	NIV	25.95091998	115.484893	130.256805		Farmer owned field
193760	2019	Jiangxi	Rice	*F. asiaticum*	15ADON	25.95091998	115.484893	130.256805		Farmer owned field
193759	2019	Jiangxi	Rice	*F. asiaticum*	15ADON	26.35685303	115.85511	NA		Farmer owned field
193758	2019	Jiangxi	Rice	*F. asiaticum*	NIV	26.35685303	115.85511	NA		Farmer owned field
193757	2019	Jiangxi	Rice	*F. asiaticum*	15ADON	26.35685303	115.85511	NA		Farmer owned field
193756	2019	Jiangxi	Rice	*F. asiaticum*	NIV	26.35685303	115.85511	NA		Farmer owned field
193755	2019	Jiangxi	Rice	*F. asiaticum*	NIV	26.35685303	115.85511	NA		Farmer owned field
193754	2019	Jiangxi	Rice	*F. asiaticum*	15ADON	26.35685303	115.85511	NA		Farmer owned field
193753	2019	Jiangxi	Rice	*F. asiaticum*	NIV	26.35685303	115.85511	NA		Farmer owned field
193752	2019	Jiangxi	Rice	*F. asiaticum*	15ADON	26.35685303	115.85511	NA		Farmer owned field
193751	2019	Jiangxi	Rice	*F. asiaticum*	NIV	26.35685303	115.85511	NA		Farmer owned field
193750	2019	Jiangxi	Rice	*F. asiaticum*	NIV	26.35685303	115.85511	NA		Farmer owned field
193749	2019	Jiangxi	Rice	*F. asiaticum*	NIV	26.35685303	115.85511	NA		Farmer owned field
193748	2019	Jiangxi	Rice	*F. asiaticum*	NIV	26.35685303	115.85511	NA		Farmer owned field
193747	2019	Jiangxi	Rice	*F. asiaticum*	NIV	26.35685303	115.85511	NA		Farmer owned field
193746	2019	Jiangxi	Rice	*F. asiaticum*	15ADON	26.35685303	115.85511	NA		Farmer owned field
193745	2019	Jiangxi	Rice	*F. asiaticum*	15ADON	26.35685303	115.85511	NA		Farmer owned field
193744	2019	Jiangxi	Rice	*F. asiaticum*	NIV	26.35685303	115.85511	NA		Farmer owned field
193743	2019	Jiangxi	Rice	*F. asiaticum*	NIV	26.35685303	115.85511	NA		Farmer owned field
193742	2019	Jiangxi	Rice	*F. asiaticum*	NIV	26.35685303	115.85511	NA		Farmer owned field
193741	2019	Jiangxi	Rice	*F. asiaticum*	NIV	26.35685303	115.85511	NA		Farmer owned field
193740	2019	Jiangxi	Rice	*F. asiaticum*	15ADON	26.35685303	115.85511	NA		Farmer owned field
193739	2019	Jiangxi	Rice	*F. asiaticum*	NIV	26.35685303	115.85511	NA		Farmer owned field
193738	2019	Jiangxi	Rice	*F. asiaticum*	NIV	26.35685303	115.85511	NA		Farmer owned field
193737	2019	Jiangxi	Rice	*F. asiaticum*	NIV	26.35685303	115.85511	NA		Farmer owned field

193736	2019	Jiangxi	Rice	*F. asiaticum*	NIV	26.35685303	115.85511	NA	Farmer owned field
193735	2019	Jiangxi	Rice	*F. asiaticum*	15ADON	26.35685303	115.85511	NA	Farmer owned field
193633	2019	Jiangxi	Rice	*F. asiaticum*	NIV	26.69128099	115.894336	274.29126	Farmer owned field
193632	2019	Jiangxi	Rice	*F. asiaticum*	NIV	26.69128099	115.894336	274.29126	Farmer owned field
193629	2019	Jiangxi	Rice	*F. asiaticum*	NIV	26.69128099	115.894336	274.29126	Farmer owned field
193628	2019	Jiangxi	Rice	*F. asiaticum*	NIV	26.69128099	115.894336	274.29126	Farmer owned field
193627	2019	Jiangxi	Rice	*F. asiaticum*	NIV	26.69128099	115.894336	274.29126	Farmer owned field
193625	2019	Jiangxi	Rice	*F. asiaticum*	15ADON	26.69128099	115.894336	274.29126	Farmer owned field
193624	2019	Jiangxi	Rice	*F. asiaticum*	NIV	26.69128099	115.894336	274.29126	Farmer owned field
193623	2019	Jiangxi	Rice	*F. asiaticum*	NIV	26.69128099	115.894336	274.29126	Farmer owned field
193622	2019	Jiangxi	Rice	*F. asiaticum*	NIV	26.69128099	115.894336	274.29126	Farmer owned field
193621	2019	Jiangxi	Rice	*F. asiaticum*	NIV	26.69128099	115.894336	274.29126	Farmer owned field
193620	2019	Jiangxi	Rice	*F. asiaticum*	NIV	26.69128099	115.894336	274.29126	Farmer owned field
193619	2019	Jiangxi	Rice	*F. asiaticum*	NIV	26.69128099	115.894336	274.29126	Farmer owned field
193618	2019	Jiangxi	Rice	*F. asiaticum*	NIV	26.69128099	115.894336	274.29126	Farmer owned field
193617	2019	Jiangxi	Rice	*F. asiaticum*	15ADON	26.69128099	115.894336	274.29126	Farmer owned field
193616	2019	Jiangxi	Rice	*F. asiaticum*	NIV	26.69128099	115.894336	274.29126	Farmer owned field
193615	2019	Jiangxi	Rice	*F. asiaticum*	NIV	26.69128099	115.894336	274.29126	Farmer owned field
193614	2019	Jiangxi	Rice	*F. asiaticum*	NIV	26.69128099	115.894336	274.29126	Farmer owned field
193613	2019	Jiangxi	Rice	*F. asiaticum*	NIV	26.69128099	115.894336	274.29126	Farmer owned field
193689	2019	Jiangxi	Rice	*F. asiaticum*	NIV	27.21012298	115.726427	122.476799	Farmer owned field
193688	2019	Jiangxi	Rice	*F. asiaticum*	NIV	27.21012298	115.726427	122.476799	Farmer owned field
193687	2019	Jiangxi	Rice	*F. asiaticum*	NIV	27.21012298	115.726427	122.476799	Farmer owned field
193686	2019	Jiangxi	Rice	*F. asiaticum*	NIV	27.21012298	115.726427	122.476799	Farmer owned field
193685	2019	Jiangxi	Rice	*F. asiaticum*	NIV	27.21012298	115.726427	122.476799	Farmer owned field
193684	2019	Jiangxi	Rice	*F. asiaticum*	NIV	27.21012298	115.726427	122.476799	Farmer owned field
193683	2019	Jiangxi	Rice	*F. asiaticum*	NIV	27.21012298	115.726427	122.476799	Farmer owned field
193682	2019	Jiangxi	Rice	*F. asiaticum*	NIV	27.21012298	115.726427	122.476799	Farmer owned field
193681	2019	Jiangxi	Rice	*F. asiaticum*	NIV	27.21012298	115.726427	122.476799	Farmer owned field
193680	2019	Jiangxi	Rice	*F. asiaticum*	15ADON	27.21012298	115.726427	122.476799	Farmer owned field
193679	2019	Jiangxi	Rice	*F. asiaticum*	NIV	27.21012298	115.726427	122.476799	Farmer owned field
193678	2019	Jiangxi	Rice	*F. asiaticum*	NIV	27.21012298	115.726427	122.476799	Farmer owned field
193677	2019	Jiangxi	Rice	*F. asiaticum*	NIV	27.21012298	115.726427	122.476799	Farmer owned field
193676	2019	Jiangxi	Rice	*F. asiaticum*	NIV	27.21012298	115.726427	122.476799	Farmer owned field
193675	2019	Jiangxi	Rice	*F. asiaticum*	NIV	27.21012298	115.726427	122.476799	Farmer owned field

193674	2019	Jiangxi	Rice	*F. asiaticum*	NIV	27.21012298	115.726427	122.476799	Farmer owned field
193673	2019	Jiangxi	Rice	*F. asiaticum*	NIV	27.21012298	115.726427	122.476799	Farmer owned field
193672	2019	Jiangxi	Rice	*F. asiaticum*	NIV	27.21012298	115.726427	122.476799	Farmer owned field
193671	2019	Jiangxi	Rice	*F. asiaticum*	NIV	27.21012298	115.726427	122.476799	Farmer owned field
193670	2019	Jiangxi	Rice	*F. asiaticum*	NIV	27.21012298	115.726427	122.476799	Farmer owned field
193668	2019	Jiangxi	Rice	*F. asiaticum*	NIV	27.21012298	115.726427	122.476799	Farmer owned field
193667	2019	Jiangxi	Rice	*F. asiaticum*	NIV	27.21012298	115.726427	122.476799	Farmer owned field
193666	2019	Jiangxi	Rice	*F. asiaticum*	NIV	27.21012298	115.726427	122.476799	Farmer owned field
193665	2019	Jiangxi	Rice	*F. asiaticum*	NIV	27.21012298	115.726427	122.476799	Farmer owned field
193664	2019	Jiangxi	Rice	*F. asiaticum*	NIV	27.21012298	115.726427	122.476799	Farmer owned field
193663	2019	Jiangxi	Rice	*F. asiaticum*	NIV	27.21012298	115.726427	122.476799	Farmer owned field
193662	2019	Jiangxi	Rice	*F. asiaticum*	NIV	27.21012298	115.726427	122.476799	Farmer owned field
193660	2019	Jiangxi	Rice	*F. asiaticum*	NIV	27.21012298	115.726427	122.476799	Farmer owned field
193600	2019	Jiangxi	Rice	*F. asiaticum*	NIV	27.21012298	115.726427	122.476799	Farmer owned field
193599	2019	Jiangxi	Rice	*F. asiaticum*	NIV	27.21012298	115.726427	122.476799	Farmer owned field
193597	2019	Jiangxi	Rice	*F. asiaticum*	NIV	27.21012298	115.726427	122.476799	Farmer owned field
193596	2019	Jiangxi	Rice	*F. asiaticum*	NIV	27.21012298	115.726427	122.476799	Farmer owned field
193445	2019	Jiangxi	Rice	*F. asiaticum*	NIV	27.62524604	115.775083	97.48848	Farmer owned field
193444	2019	Jiangxi	Rice	*F. asiaticum*	NIV	27.62524604	115.775083	97.48848	Farmer owned field
193443	2019	Jiangxi	Rice	*F. asiaticum*	NIV	27.62524604	115.775083	97.48848	Farmer owned field
193442	2019	Jiangxi	Rice	*F. asiaticum*	NIV	27.62524604	115.775083	97.48848	Farmer owned field
193441	2019	Jiangxi	Rice	*F. asiaticum*	NIV	27.62524604	115.775083	97.48848	Farmer owned field
193440	2019	Jiangxi	Rice	*F. asiaticum*	NIV	27.62524604	115.775083	97.48848	Farmer owned field
193439	2019	Jiangxi	Rice	*F. asiaticum*	NIV	27.62524604	115.775083	97.48848	Farmer owned field
193438	2019	Jiangxi	Rice	*F. asiaticum*	NIV	27.62524604	115.775083	97.48848	Farmer owned field
193436	2019	Jiangxi	Rice	*F. asiaticum*	NIV	27.62524604	115.775083	97.48848	Farmer owned field
193435	2019	Jiangxi	Rice	*F. asiaticum*	NIV	27.62524604	115.775083	97.48848	Farmer owned field
193434	2019	Jiangxi	Rice	*F. asiaticum*	NIV	27.62524604	115.775083	97.48848	Farmer owned field
193433	2019	Jiangxi	Rice	*F. asiaticum*	NIV	27.62524604	115.775083	97.48848	Farmer owned field
193432	2019	Jiangxi	Rice	*F. asiaticum*	NIV	27.62524604	115.775083	97.48848	Farmer owned field
193431	2019	Jiangxi	Rice	*F. asiaticum*	NIV	27.62524604	115.775083	97.48848	Farmer owned field
193430	2019	Jiangxi	Rice	*F. asiaticum*	NIV	27.62524604	115.775083	97.48848	Farmer owned field
193429	2019	Jiangxi	Rice	*F. asiaticum*	NIV	27.62524604	115.775083	97.48848	Farmer owned field
193428	2019	Jiangxi	Rice	*F. asiaticum*	NIV	27.62524604	115.775083	97.48848	Farmer owned field
193427	2019	Jiangxi	Rice	*F. asiaticum*	NIV	27.62524604	115.775083	97.48848	Farmer owned field

193425	2019	Jiangxi	Rice	*F. asiaticum*	NIV	27.62524604	115.775083	97.48848		Farmer owned field
193424	2019	Jiangxi	Rice	*F. asiaticum*	NIV	27.62524604	115.775083	97.48848		Farmer owned field
193564	2019	Jiangxi	Rice	*F. asiaticum*	NIV	27.99478797	115.827084	32.675232		Farmer owned field
193563	2019	Jiangxi	Rice	*F. asiaticum*	NIV	27.99478797	115.827084	32.675232		Farmer owned field
193561	2019	Jiangxi	Rice	*F. asiaticum*	NIV	27.99478797	115.827084	32.675232		Farmer owned field
193560	2019	Jiangxi	Rice	*F. asiaticum*	NIV	27.99478797	115.827084	32.675232		Farmer owned field
193559	2019	Jiangxi	Rice	*F. asiaticum*	NIV	27.99478797	115.827084	32.675232		Farmer owned field
193557	2019	Jiangxi	Rice	*F. asiaticum*	NIV	27.99478797	115.827084	32.675232		Farmer owned field
193556	2019	Jiangxi	Rice	*F. asiaticum*	NIV	27.99478797	115.827084	32.675232		Farmer owned field
193555	2019	Jiangxi	Rice	*F. asiaticum*	3ADON	27.99478797	115.827084	32.675232		Farmer owned field
193554	2019	Jiangxi	Rice	*F. asiaticum*	NIV	27.99478797	115.827084	32.675232		Farmer owned field
193552	2019	Jiangxi	Rice	*F. asiaticum*	NIV	27.99478797	115.827084	32.675232		Farmer owned field
193551	2019	Jiangxi	Rice	*F. asiaticum*	3ADON	27.99478797	115.827084	32.675232		Farmer owned field
193550	2019	Jiangxi	Rice	*F. asiaticum*	NIV	27.99478797	115.827084	32.675232		Farmer owned field
193549	2019	Jiangxi	Rice	*F. asiaticum*	NIV	27.99478797	115.827084	32.675232		Farmer owned field
193548	2019	Jiangxi	Rice	*F. asiaticum*	15ADON	27.99478797	115.827084	32.675232		Farmer owned field
193547	2019	Jiangxi	Rice	*F. asiaticum*	15ADON	27.99478797	115.827084	32.675232		Farmer owned field
193546	2019	Jiangxi	Rice	*F. asiaticum*	15ADON	27.99478797	115.827084	32.675232		Farmer owned field
193544	2019	Jiangxi	Rice	*F. asiaticum*	NIV	27.99478797	115.827084	32.675232		Farmer owned field
193543	2019	Jiangxi	Rice	*F. asiaticum*	NIV	27.99478797	115.827084	32.675232		Farmer owned field
193542	2019	Jiangxi	Rice	*F. asiaticum*	NIV	27.99478797	115.827084	32.675232		Farmer owned field
193540	2019	Jiangxi	Rice	*F. asiaticum*	15ADON	27.99478797	115.827084	32.675232		Farmer owned field
193612	2019	Jiangxi	Rice	*F. asiaticum*	NIV	28.17531204	115.858429	21.978415		Farmer owned field
193611	2019	Jiangxi	Rice	*F. asiaticum*	NIV	28.17531204	115.858429	21.978415		Farmer owned field
193610	2019	Jiangxi	Rice	*F. asiaticum*	NIV	28.17531204	115.858429	21.978415		Farmer owned field
193609	2019	Jiangxi	Rice	*F. asiaticum*	NIV	28.17531204	115.858429	21.978415		Farmer owned field
193608	2019	Jiangxi	Rice	*F. asiaticum*	NIV	28.17531204	115.858429	21.978415		Farmer owned field
193607	2019	Jiangxi	Rice	*F. asiaticum*	NIV	28.17531204	115.858429	21.978415		Farmer owned field
193606	2019	Jiangxi	Rice	*F. asiaticum*	NIV	28.17531204	115.858429	21.978415		Farmer owned field
193605	2019	Jiangxi	Rice	*F. asiaticum*	NIV	28.17531204	115.858429	21.978415		Farmer owned field
193604	2019	Jiangxi	Rice	*F. asiaticum*	15ADON	28.17531204	115.858429	21.978415		Farmer owned field
193603	2019	Jiangxi	Rice	*F. asiaticum*	NIV	28.17531204	115.858429	21.978415		Farmer owned field
193602	2019	Jiangxi	Rice	*F. asiaticum*	NIV	28.17531204	115.858429	21.978415		Farmer owned field
193601	2019	Jiangxi	Rice	*F. asiaticum*	NIV	28.17531204	115.858429	21.978415		Farmer owned field
193456	2019	Jiangxi	Rice	*F. asiaticum*	NIV	28.17531204	115.858429	21.978415		Farmer owned field

193455	2019	Jiangxi	Rice	*F. asiaticum*	NIV	28.17531204	115.858429	21.978415		Farmer owned field
193454	2019	Jiangxi	Rice	*F. asiaticum*	NIV	28.17531204	115.858429	21.978415		Farmer owned field
193453	2019	Jiangxi	Rice	*F. asiaticum*	NIV	28.17531204	115.858429	21.978415		Farmer owned field
193452	2019	Jiangxi	Rice	*F. asiaticum*	NIV	28.17531204	115.858429	21.978415		Farmer owned field
193451	2019	Jiangxi	Rice	*F. asiaticum*	NIV	28.17531204	115.858429	21.978415		Farmer owned field
193450	2019	Jiangxi	Rice	*F. asiaticum*	NIV	28.17531204	115.858429	21.978415		Farmer owned field
193449	2019	Jiangxi	Rice	*F. asiaticum*	NIV	28.17531204	115.858429	21.978415		Farmer owned field
193448	2019	Jiangxi	Rice	*F. asiaticum*	NIV	28.17531204	115.858429	21.978415		Farmer owned field
193446	2019	Jiangxi	Rice	*F. asiaticum*	NIV	28.17531204	115.858429	21.978415		Farmer owned field
193481	2019	Jiangxi	Rice	*F. asiaticum*	15ADON	28.43460797	115.918535	19.022579		Farmer owned field
193480	2019	Jiangxi	Rice	*F. asiaticum*	NIV	28.43460797	115.918535	19.022579		Farmer owned field
193479	2019	Jiangxi	Rice	*F. asiaticum*	NIV	28.43460797	115.918535	19.022579		Farmer owned field
193478	2019	Jiangxi	Rice	*F. asiaticum*	NIV	28.43460797	115.918535	19.022579		Farmer owned field
193477	2019	Jiangxi	Rice	*F. asiaticum*	3ADON	28.43460797	115.918535	19.022579		Farmer owned field
193476	2019	Jiangxi	Rice	*F. asiaticum*	NIV	28.43460797	115.918535	19.022579		Farmer owned field
193475	2019	Jiangxi	Rice	*F. asiaticum*	NIV	28.43460797	115.918535	19.022579		Farmer owned field
193474	2019	Jiangxi	Rice	*F. asiaticum*	15ADON	28.43460797	115.918535	19.022579		Farmer owned field
193473	2019	Jiangxi	Rice	*F. asiaticum*	NIV	28.43460797	115.918535	19.022579		Farmer owned field
193472	2019	Jiangxi	Rice	*F. asiaticum*	3ADON	28.43460797	115.918535	19.022579		Farmer owned field
193471	2019	Jiangxi	Rice	*F. asiaticum*	NIV	28.43460797	115.918535	19.022579		Farmer owned field
193469	2019	Jiangxi	Rice	*F. asiaticum*	NIV	28.43460797	115.918535	19.022579		Farmer owned field
193468	2019	Jiangxi	Rice	*F. asiaticum*	3ADON	28.43460797	115.918535	19.022579		Farmer owned field
193467	2019	Jiangxi	Rice	*F. asiaticum*	NIV	28.43460797	115.918535	19.022579		Farmer owned field
193466	2019	Jiangxi	Rice	*F. asiaticum*	NIV	28.43460797	115.918535	19.022579		Farmer owned field
193465	2019	Jiangxi	Rice	*F. asiaticum*	NIV	28.43460797	115.918535	19.022579		Farmer owned field
193464	2019	Jiangxi	Rice	*F. asiaticum*	NIV	28.43460797	115.918535	19.022579		Farmer owned field
193463	2019	Jiangxi	Rice	*F. asiaticum*	NIV	28.43460797	115.918535	19.022579		Farmer owned field
193462	2019	Jiangxi	Rice	*F. asiaticum*	NIV	28.43460797	115.918535	19.022579		Farmer owned field
193461	2019	Jiangxi	Rice	*F. asiaticum*	NIV	28.43460797	115.918535	19.022579		Farmer owned field
193460	2019	Jiangxi	Rice	*F. asiaticum*	3ADON	28.43460797	115.918535	19.022579		Farmer owned field
193459	2019	Jiangxi	Rice	*F. asiaticum*	NIV	28.43460797	115.918535	19.022579		Farmer owned field
193458	2019	Jiangxi	Rice	*F. asiaticum*	NIV	28.43460797	115.918535	19.022579		Farmer owned field
193711	2019	Jiangxi	Rice	*F. asiaticum*	NIV	28.65760004	116.138187	34.28743		Farmer owned field
193709	2019	Jiangxi	Rice	*F. asiaticum*	NIV	28.65760004	116.138187	34.28743		Farmer owned field
193708	2019	Jiangxi	Rice	*F. asiaticum*	NIV	28.65760004	116.138187	34.28743		Farmer owned field

193707	2019	Jiangxi	Rice	*F. asiaticum*	3ADON	28.65760004	116.138187	34.28743	Farmer owned field
193706	2019	Jiangxi	Rice	*F. asiaticum*	NIV	28.65760004	116.138187	34.28743	Farmer owned field
193705	2019	Jiangxi	Rice	*F. asiaticum*	NIV	28.65760004	116.138187	34.28743	Farmer owned field
193704	2019	Jiangxi	Rice	*F. asiaticum*	NIV	28.65760004	116.138187	34.28743	Farmer owned field
193703	2019	Jiangxi	Rice	*F. asiaticum*	NIV	28.65760004	116.138187	34.28743	Farmer owned field
193702	2019	Jiangxi	Rice	*F. asiaticum*	3ADON	28.65760004	116.138187	34.28743	Farmer owned field
193700	2019	Jiangxi	Rice	*F. asiaticum*	NIV	28.65760004	116.138187	34.28743	Farmer owned field
193699	2019	Jiangxi	Rice	*F. asiaticum*	NIV	28.65760004	116.138187	34.28743	Farmer owned field
193698	2019	Jiangxi	Rice	*F. asiaticum*	NIV	28.65760004	116.138187	34.28743	Farmer owned field
193697	2019	Jiangxi	Rice	*F. asiaticum*	NIV	28.65760004	116.138187	34.28743	Farmer owned field
193696	2019	Jiangxi	Rice	*F. asiaticum*	NIV	28.65760004	116.138187	34.28743	Farmer owned field
193695	2019	Jiangxi	Rice	*F. asiaticum*	NIV	28.65760004	116.138187	34.28743	Farmer owned field
193693	2019	Jiangxi	Rice	*F. asiaticum*	NIV	28.65760004	116.138187	34.28743	Farmer owned field
193692	2019	Jiangxi	Rice	*F. asiaticum*	NIV	28.65760004	116.138187	34.28743	Farmer owned field
193691	2019	Jiangxi	Rice	*F. asiaticum*	NIV	28.65760004	116.138187	34.28743	Farmer owned field
193690	2019	Jiangxi	Rice	*F. asiaticum*	15ADON	28.65760004	116.138187	34.28743	Farmer owned field
193271	2019	Jiangxi	Rice	*F. asiaticum*	NIV	28.78960001	116.651892	21.585388	Farmer owned field
193252	2019	Jiangxi	Rice	*F. asiaticum*	NIV	28.78960001	116.651892	21.585388	Farmer owned field
193251	2019	Jiangxi	Rice	*F. asiaticum*	NIV	28.78960001	116.651892	21.585388	Farmer owned field
193250	2019	Jiangxi	Rice	*F. asiaticum*	NIV	28.78960001	116.651892	21.585388	Farmer owned field
193249	2019	Jiangxi	Rice	*F. asiaticum*	NIV	28.78960001	116.651892	21.585388	Farmer owned field
193248	2019	Jiangxi	Rice	*F. asiaticum*	NIV	28.78960001	116.651892	21.585388	Farmer owned field
193247	2019	Jiangxi	Rice	*F. asiaticum*	NIV	28.78960001	116.651892	21.585388	Farmer owned field
193246	2019	Jiangxi	Rice	*F. asiaticum*	NIV	28.78960001	116.651892	21.585388	Farmer owned field
193245	2019	Jiangxi	Rice	*F. asiaticum*	NIV	28.78960001	116.651892	21.585388	Farmer owned field
193244	2019	Jiangxi	Rice	*F. asiaticum*	NIV	28.78960001	116.651892	21.585388	Farmer owned field
193243	2019	Jiangxi	Rice	*F. asiaticum*	NIV	28.78960001	116.651892	21.585388	Farmer owned field
193242	2019	Jiangxi	Rice	*F. asiaticum*	NIV	28.78960001	116.651892	21.585388	Farmer owned field
193241	2019	Jiangxi	Rice	*F. asiaticum*	NIV	28.78960001	116.651892	21.585388	Farmer owned field
193240	2019	Jiangxi	Rice	*F. asiaticum*	NIV	28.78960001	116.651892	21.585388	Farmer owned field
193239	2019	Jiangxi	Rice	*F. asiaticum*	NIV	28.78960001	116.651892	21.585388	Farmer owned field
193238	2019	Jiangxi	Rice	*F. asiaticum*	NIV	28.78960001	116.651892	21.585388	Farmer owned field
193237	2019	Jiangxi	Rice	*F. asiaticum*	NIV	28.78960001	116.651892	21.585388	Farmer owned field
193236	2019	Jiangxi	Rice	*F. asiaticum*	NIV	28.78960001	116.651892	21.585388	Farmer owned field
193235	2019	Jiangxi	Rice	*F. asiaticum*	NIV	28.78960001	116.651892	21.585388	Farmer owned field

193234	2019	Jiangxi	Rice	*F. asiaticum*	NIV	28.78960001	116.651892	21.585388		Farmer owned field
193233	2019	Jiangxi	Rice	*F. asiaticum*	NIV	28.78960001	116.651892	21.585388		Farmer owned field
193232	2019	Jiangxi	Rice	*F. asiaticum*	NIV	28.78960001	116.651892	21.585388		Farmer owned field
193231	2019	Jiangxi	Rice	*F. asiaticum*	NIV	28.78960001	116.651892	21.585388		Farmer owned field
193230	2019	Jiangxi	Rice	*F. asiaticum*	NIV	28.78960001	116.651892	21.585388		Farmer owned field
193595	2019	Jiangxi	Rice	*F. asiaticum*	NIV	28.995206	115.793063	56.386806		Farmer owned field
193594	2019	Jiangxi	Rice	*F. asiaticum*	3ADON	28.995206	115.793063	56.386806		Farmer owned field
193593	2019	Jiangxi	Rice	*F. asiaticum*	NIV	28.995206	115.793063	56.386806		Farmer owned field
193592	2019	Jiangxi	Rice	*F. asiaticum*	NIV	28.995206	115.793063	56.386806		Farmer owned field
193590	2019	Jiangxi	Rice	*F. asiaticum*	NIV	28.995206	115.793063	56.386806		Farmer owned field
193587	2019	Jiangxi	Rice	*F. asiaticum*	NIV	28.995206	115.793063	56.386806		Farmer owned field
193585	2019	Jiangxi	Rice	*F. asiaticum*	NIV	28.995206	115.793063	56.386806		Farmer owned field
193584	2019	Jiangxi	Rice	*F. asiaticum*	3ADON	28.995206	115.793063	56.386806		Farmer owned field
193583	2019	Jiangxi	Rice	*F. asiaticum*	NIV	28.995206	115.793063	56.386806		Farmer owned field
193582	2019	Jiangxi	Rice	*F. asiaticum*	NIV	28.995206	115.793063	56.386806		Farmer owned field
193580	2019	Jiangxi	Rice	*F. asiaticum*	15ADON	28.995206	115.793063	56.386806		Farmer owned field
193579	2019	Jiangxi	Rice	*F. asiaticum*	NIV	28.995206	115.793063	56.386806		Farmer owned field
193578	2019	Jiangxi	Rice	*F. asiaticum*	NIV	28.995206	115.793063	56.386806		Farmer owned field
193577	2019	Jiangxi	Rice	*F. asiaticum*	NIV	28.995206	115.793063	56.386806		Farmer owned field
193576	2019	Jiangxi	Rice	*F. asiaticum*	NIV	28.995206	115.793063	56.386806		Farmer owned field
193575	2019	Jiangxi	Rice	*F. asiaticum*	3ADON	28.995206	115.793063	56.386806		Farmer owned field
193574	2019	Jiangxi	Rice	*F. asiaticum*	NIV	28.995206	115.793063	56.386806		Farmer owned field
193573	2019	Jiangxi	Rice	*F. asiaticum*	NIV	28.995206	115.793063	56.386806		Farmer owned field
193571	2019	Jiangxi	Rice	*F. asiaticum*	3ADON	28.995206	115.793063	56.386806		Farmer owned field
193569	2019	Jiangxi	Rice	*F. asiaticum*	NIV	28.995206	115.793063	56.386806		Farmer owned field
193568	2019	Jiangxi	Rice	*F. asiaticum*	NIV	28.995206	115.793063	56.386806		Farmer owned field
193567	2019	Jiangxi	Rice	*F. asiaticum*	NIV	28.995206	115.793063	56.386806		Farmer owned field
193566	2019	Jiangxi	Rice	*F. asiaticum*	NIV	28.995206	115.793063	56.386806		Farmer owned field
193734	2019	Jiangxi	Rice	*F. asiaticum*	NIV	29.03102196	117.045928	35.818413		Farmer owned field
193733	2019	Jiangxi	Rice	*F. asiaticum*	NIV	29.03102196	117.045928	35.818413		Farmer owned field
193732	2019	Jiangxi	Rice	*F. asiaticum*	NIV	29.03102196	117.045928	35.818413		Farmer owned field
193731	2019	Jiangxi	Rice	*F. asiaticum*	NIV	29.03102196	117.045928	35.818413		Farmer owned field
193730	2019	Jiangxi	Rice	*F. asiaticum*	NIV	29.03102196	117.045928	35.818413		Farmer owned field
193729	2019	Jiangxi	Rice	*F. asiaticum*	NIV	29.03102196	117.045928	35.818413		Farmer owned field
193728	2019	Jiangxi	Rice	*F. asiaticum*	NIV	29.03102196	117.045928	35.818413		Farmer owned field

193727	2019	Jiangxi	Rice	*F. asiaticum*	NIV	29.03102196	117.045928	35.818413		Farmer owned field
193726	2019	Jiangxi	Rice	*F. asiaticum*	NIV	29.03102196	117.045928	35.818413		Farmer owned field
193725	2019	Jiangxi	Rice	*F. asiaticum*	NIV	29.03102196	117.045928	35.818413		Farmer owned field
193724	2019	Jiangxi	Rice	*F. asiaticum*	NIV	29.03102196	117.045928	35.818413		Farmer owned field
193723	2019	Jiangxi	Rice	*F. asiaticum*	NIV	29.03102196	117.045928	35.818413		Farmer owned field
193722	2019	Jiangxi	Rice	*F. asiaticum*	NIV	29.03102196	117.045928	35.818413		Farmer owned field
193721	2019	Jiangxi	Rice	*F. asiaticum*	NIV	29.03102196	117.045928	35.818413		Farmer owned field
193720	2019	Jiangxi	Rice	*F. asiaticum*	NIV	29.03102196	117.045928	35.818413		Farmer owned field
193719	2019	Jiangxi	Rice	*F. asiaticum*	NIV	29.03102196	117.045928	35.818413		Farmer owned field
193718	2019	Jiangxi	Rice	*F. asiaticum*	NIV	29.03102196	117.045928	35.818413		Farmer owned field
193717	2019	Jiangxi	Rice	*F. asiaticum*	NIV	29.03102196	117.045928	35.818413		Farmer owned field
193715	2019	Jiangxi	Rice	*F. asiaticum*	NIV	29.03102196	117.045928	35.818413		Farmer owned field
193714	2019	Jiangxi	Rice	*F. asiaticum*	NIV	29.03102196	117.045928	35.818413		Farmer owned field
193713	2019	Jiangxi	Rice	*F. asiaticum*	NIV	29.03102196	117.045928	35.818413		Farmer owned field
193712	2019	Jiangxi	Rice	*F. asiaticum*	NIV	29.03102196	117.045928	35.818413		Farmer owned field
193309	2019	Jiangxi	Rice	*F. asiaticum*	NIV	29.23951199	115.740063	79.27137		Farmer owned field
193308	2019	Jiangxi	Rice	*F. asiaticum*	NIV	29.23951199	115.740063	79.27137		Farmer owned field
193307	2019	Jiangxi	Rice	*F. asiaticum*	3ADON	29.23951199	115.740063	79.27137		Farmer owned field
193306	2019	Jiangxi	Rice	*F. asiaticum*	3ADON	29.23951199	115.740063	79.27137		Farmer owned field
193305	2019	Jiangxi	Rice	*F. asiaticum*	NIV	29.23951199	115.740063	79.27137		Farmer owned field
193304	2019	Jiangxi	Rice	*F. asiaticum*	NIV	29.23951199	115.740063	79.27137		Farmer owned field
193303	2019	Jiangxi	Rice	*F. asiaticum*	NIV	29.23951199	115.740063	79.27137		Farmer owned field
193302	2019	Jiangxi	Rice	*F. asiaticum*	NIV	29.23951199	115.740063	79.27137		Farmer owned field
193300	2019	Jiangxi	Rice	*F. asiaticum*	3ADON	29.23951199	115.740063	79.27137		Farmer owned field
193299	2019	Jiangxi	Rice	*F. asiaticum*	NIV	29.23951199	115.740063	79.27137		Farmer owned field
193298	2019	Jiangxi	Rice	*F. asiaticum*	NIV	29.23951199	115.740063	79.27137		Farmer owned field
193297	2019	Jiangxi	Rice	*F. asiaticum*	NIV	29.23951199	115.740063	79.27137		Farmer owned field
193296	2019	Jiangxi	Rice	*F. asiaticum*	NIV	29.23951199	115.740063	79.27137		Farmer owned field
193295	2019	Jiangxi	Rice	*F. asiaticum*	NIV	29.23951199	115.740063	79.27137		Farmer owned field
193294	2019	Jiangxi	Rice	*F. asiaticum*	3ADON	29.23951199	115.740063	79.27137		Farmer owned field
193293	2019	Jiangxi	Rice	*F. asiaticum*	NIV	29.23951199	115.740063	79.27137		Farmer owned field
193292	2019	Jiangxi	Rice	*F. asiaticum*	NIV	29.23951199	115.740063	79.27137		Farmer owned field
193291	2019	Jiangxi	Rice	*F. asiaticum*	NIV	29.23951199	115.740063	79.27137		Farmer owned field
193290	2019	Jiangxi	Rice	*F. asiaticum*	NIV	29.23951199	115.740063	79.27137		Farmer owned field
193289	2019	Jiangxi	Rice	*F. asiaticum*	NIV	29.23951199	115.740063	79.27137		Farmer owned field

193288	2019	Jiangxi	Rice	*F. asiaticum*	NIV	29.23951199	115.740063	79.27137		Farmer owned field
193287	2019	Jiangxi	Rice	*F. asiaticum*	NIV	29.23951199	115.740063	79.27137		Farmer owned field
193286	2019	Jiangxi	Rice	*F. asiaticum*	NIV	29.23951199	115.740063	79.27137		Farmer owned field
193285	2019	Jiangxi	Rice	*F. asiaticum*	NIV	29.23951199	115.740063	79.27137		Farmer owned field
193284	2019	Jiangxi	Rice	*F. asiaticum*	NIV	29.23951199	115.740063	79.27137		Farmer owned field
193659	2019	Jiangxi	Rice	*F. asiaticum*	NIV	29.44213796	117.38522	49.038155		Farmer owned field
193658	2019	Jiangxi	Rice	*F. asiaticum*	NIV	29.44213796	117.38522	49.038155		Farmer owned field
193657	2019	Jiangxi	Rice	*F. asiaticum*	NIV	29.44213796	117.38522	49.038155		Farmer owned field
193656	2019	Jiangxi	Rice	*F. asiaticum*	15ADON	29.44213796	117.38522	49.038155		Farmer owned field
193655	2019	Jiangxi	Rice	*F. asiaticum*	NIV	29.44213796	117.38522	49.038155		Farmer owned field
193654	2019	Jiangxi	Rice	*F. asiaticum*	NIV	29.44213796	117.38522	49.038155		Farmer owned field
193653	2019	Jiangxi	Rice	*F. asiaticum*	NIV	29.44213796	117.38522	49.038155		Farmer owned field
193652	2019	Jiangxi	Rice	*F. asiaticum*	NIV	29.44213796	117.38522	49.038155		Farmer owned field
193651	2019	Jiangxi	Rice	*F. asiaticum*	NIV	29.44213796	117.38522	49.038155		Farmer owned field
193650	2019	Jiangxi	Rice	*F. asiaticum*	NIV	29.44213796	117.38522	49.038155		Farmer owned field
193649	2019	Jiangxi	Rice	*F. asiaticum*	NIV	29.44213796	117.38522	49.038155		Farmer owned field
193648	2019	Jiangxi	Rice	*F. asiaticum*	15ADON	29.44213796	117.38522	49.038155		Farmer owned field
193647	2019	Jiangxi	Rice	*F. asiaticum*	NIV	29.44213796	117.38522	49.038155		Farmer owned field
193646	2019	Jiangxi	Rice	*F. asiaticum*	NIV	29.44213796	117.38522	49.038155		Farmer owned field
193645	2019	Jiangxi	Rice	*F. asiaticum*	NIV	29.44213796	117.38522	49.038155		Farmer owned field
193644	2019	Jiangxi	Rice	*F. asiaticum*	NIV	29.44213796	117.38522	49.038155		Farmer owned field
193643	2019	Jiangxi	Rice	*F. asiaticum*	NIV	29.44213796	117.38522	49.038155		Farmer owned field
193642	2019	Jiangxi	Rice	*F. asiaticum*	NIV	29.44213796	117.38522	49.038155		Farmer owned field
193641	2019	Jiangxi	Rice	*F. asiaticum*	NIV	29.44213796	117.38522	49.038155		Farmer owned field
193640	2019	Jiangxi	Rice	*F. asiaticum*	NIV	29.44213796	117.38522	49.038155		Farmer owned field
193639	2019	Jiangxi	Rice	*F. asiaticum*	NIV	29.44213796	117.38522	49.038155		Farmer owned field
193638	2019	Jiangxi	Rice	*F. asiaticum*	NIV	29.44213796	117.38522	49.038155		Farmer owned field
193637	2019	Jiangxi	Rice	*F. asiaticum*	NIV	29.44213796	117.38522	49.038155		Farmer owned field
193636	2019	Jiangxi	Rice	*F. asiaticum*	NIV	29.44213796	117.38522	49.038155		Farmer owned field
180942	2018	Sichuan	Wheat	*F. asiaticum*	NIV	31.13249601	105.82188	496.201172	Commercialized variety	Farmer owned field
171030	2017	Hunan	Rice	*F. asiaticum*	3ADON	27.92500301	112.552357	75.3		Farmer owned field
171029	2017	Hunan	Rice	*F. asiaticum*	3ADON	27.92500301	112.552357	75.3		Farmer owned field
194053	2019	Hunan	Rice	*F. asiaticum*	3ADON	28.02993396	112.623799	68.926636		Farmer owned field
194052	2019	Hunan	Rice	*F. asiaticum*	NIV	28.02993396	112.623799	68.926636		Farmer owned field
194051	2019	Hunan	Rice	*F. asiaticum*	NIV	28.02993396	112.623799	68.926636		Farmer owned field

194050	2019	Hunan	Rice	*F. asiaticum*	NIV	28.02993396	112.623799	68.926636			Farmer owned field
194049	2019	Hunan	Rice	*F. asiaticum*	NIV	28.02993396	112.623799	68.926636			Farmer owned field
194048	2019	Hunan	Rice	*F. asiaticum*	NIV	28.02993396	112.623799	68.926636			Farmer owned field
194047	2019	Hunan	Rice	*F. asiaticum*	3ADON	28.02993396	112.623799	68.926636			Farmer owned field
194046	2019	Hunan	Rice	*F. asiaticum*	NIV	28.02993396	112.623799	68.926636			Farmer owned field
194045	2019	Hunan	Rice	*F. asiaticum*	NIV	28.02993396	112.623799	68.926636			Farmer owned field
194044	2019	Hunan	Rice	*F. asiaticum*	3ADON	28.02993396	112.623799	68.926636			Farmer owned field
194043	2019	Hunan	Rice	*F. asiaticum*	15ADON	28.02993396	112.623799	68.926636			Farmer owned field
194042	2019	Hunan	Rice	*F. asiaticum*	NIV	28.02993396	112.623799	68.926636			Farmer owned field
194041	2019	Hunan	Rice	*F. asiaticum*	NIV	28.02993396	112.623799	68.926636			Farmer owned field
194040	2019	Hunan	Rice	*F. asiaticum*	3ADON	28.02993396	112.623799	68.926636			Farmer owned field
194039	2019	Hunan	Rice	*F. asiaticum*	NIV	28.02993396	112.623799	68.926636			Farmer owned field
194038	2019	Hunan	Rice	*F. asiaticum*	NIV	28.02993396	112.623799	68.926636			Farmer owned field
194037	2019	Hunan	Rice	*F. asiaticum*	NIV	28.02993396	112.623799	68.926636			Farmer owned field
194036	2019	Hunan	Rice	*F. asiaticum*	3ADON	28.02993396	112.623799	68.926636			Farmer owned field
194035	2019	Hunan	Rice	*F. asiaticum*	NIV	28.02993396	112.623799	68.926636			Farmer owned field
194034	2019	Hunan	Rice	*F. asiaticum*	NIV	28.02993396	112.623799	68.926636			Farmer owned field
194033	2019	Hunan	Rice	*F. asiaticum*	NIV	28.02993396	112.623799	68.926636			Farmer owned field
194032	2019	Hunan	Rice	*F. asiaticum*	NIV	28.02993396	112.623799	68.926636			Farmer owned field
194031	2019	Hunan	Rice	*F. asiaticum*	NIV	28.02993396	112.623799	68.926636			Farmer owned field
194030	2019	Hunan	Rice	*F. asiaticum*	NIV	28.02993396	112.623799	68.926636			Farmer owned field
194029	2019	Hunan	Rice	*F. asiaticum*	NIV	28.02993396	112.623799	68.926636			Farmer owned field
194028	2019	Hunan	Rice	*F. asiaticum*	NIV	28.02993396	112.623799	68.926636			Farmer owned field
194027	2019	Hunan	Rice	*F. asiaticum*	NIV	28.02993396	112.623799	68.926636			Farmer owned field
194026	2019	Hunan	Rice	*F. asiaticum*	NIV	28.02993396	112.623799	68.926636			Farmer owned field
171028	2017	Hunan	Rice	*F. asiaticum*	NIV	28.02993396	112.623799	60.99			Farmer owned field
171027	2017	Hunan	Rice	*F. asiaticum*	NIV	28.02993396	112.623799	60.99			Farmer owned field
171026	2017	Hunan	Rice	*F. asiaticum*	NIV	28.02993396	112.623799	60.99			Farmer owned field
171025	2017	Hunan	Rice	*F. asiaticum*	3ADON	28.02993396	112.623799	60.99			Farmer owned field
171024	2017	Hunan	Rice	*F. asiaticum*	3ADON	28.02993396	112.623799	60.99			Farmer owned field
171023	2017	Hunan	Rice	*F. asiaticum*	NIV	28.02993396	112.623799	60.99			Farmer owned field
171022	2017	Hunan	Rice	*F. asiaticum*	3ADON	28.02993396	112.623799	60.99			Farmer owned field
171021	2017	Hunan	Rice	*F. asiaticum*	NIV	28.02993396	112.623799	60.99			Farmer owned field
171020	2017	Hunan	Rice	*F. asiaticum*	3ADON	28.02993396	112.623799	60.99			Farmer owned field
171019	2017	Hunan	Rice	*F. asiaticum*	3ADON	28.02993396	112.623799	60.99			Farmer owned field

171018	2017	Hunan	Rice	*F. asiaticum*	NIV	28.02993396	112.623799	60.99	Farmer owned field
171017	2017	Hunan	Rice	*F. asiaticum*	NIV	28.02993396	112.623799	60.99	Farmer owned field
171016	2017	Hunan	Rice	*F. asiaticum*	NIV	28.02993396	112.623799	60.99	Farmer owned field
171015	2017	Hunan	Rice	*F. asiaticum*	3ADON	28.02993396	112.623799	60.99	Farmer owned field
171014	2017	Hunan	Rice	*F. asiaticum*	NIV	28.02993396	112.623799	60.99	Farmer owned field
171013	2017	Hunan	Rice	*F. asiaticum*	3ADON	28.02993396	112.623799	60.99	Farmer owned field
171012	2017	Hunan	Rice	*F. asiaticum*	NIV	28.02993396	112.623799	60.99	Farmer owned field
171011	2017	Hunan	Rice	*F. asiaticum*	NIV	28.02993396	112.623799	60.99	Farmer owned field
194145	2019	Hunan	Rice	*F. asiaticum*	NIV	28.12392502	112.623662	96.315208	Farmer owned field
194144	2019	Hunan	Rice	*F. asiaticum*	3ADON	28.12392502	112.623662	96.315208	Farmer owned field
194143	2019	Hunan	Rice	*F. asiaticum*	NIV	28.12392502	112.623662	96.315208	Farmer owned field
194142	2019	Hunan	Rice	*F. asiaticum*	NIV	28.12392502	112.623662	96.315208	Farmer owned field
194140	2019	Hunan	Rice	*F. asiaticum*	3ADON	28.12392502	112.623662	96.315208	Farmer owned field
194139	2019	Hunan	Rice	*F. asiaticum*	NIV	28.12392502	112.623662	96.315208	Farmer owned field
194137	2019	Hunan	Rice	*F. asiaticum*	NIV	28.12392502	112.623662	96.315208	Farmer owned field
194136	2019	Hunan	Rice	*F. asiaticum*	NIV	28.12392502	112.623662	96.315208	Farmer owned field
194135	2019	Hunan	Rice	*F. asiaticum*	NIV	28.12392502	112.623662	96.315208	Farmer owned field
194134	2019	Hunan	Rice	*F. asiaticum*	NIV	28.12392502	112.623662	96.315208	Farmer owned field
194132	2019	Hunan	Rice	*F. asiaticum*	NIV	28.12392502	112.623662	96.315208	Farmer owned field
171009	2017	Hunan	Rice	*F. asiaticum*	NIV	28.12392502	112.623662	173.31	Farmer owned field
171008	2017	Hunan	Rice	*F. asiaticum*	3ADON	28.12392502	112.623662	173.31	Farmer owned field
171007	2017	Hunan	Rice	*F. asiaticum*	NIV	28.12392502	112.623662	173.31	Farmer owned field
171006	2017	Hunan	Rice	*F. asiaticum*	NIV	28.12392502	112.623662	173.31	Farmer owned field
171005	2017	Hunan	Rice	*F. asiaticum*	NIV	28.12392502	112.623662	173.31	Farmer owned field
171004	2017	Hunan	Rice	*F. asiaticum*	15ADON	28.12392502	112.623662	173.31	Farmer owned field
171003	2017	Hunan	Rice	*F. asiaticum*	3ADON	28.12392502	112.623662	173.31	Farmer owned field
171002	2017	Hunan	Rice	*F. asiaticum*	NIV	28.12392502	112.623662	173.31	Farmer owned field
171001	2017	Hunan	Rice	*F. asiaticum*	NIV	28.12392502	112.623662	173.31	Farmer owned field
171000	2017	Hunan	Rice	*F. asiaticum*	NIV	28.12392502	112.623662	173.31	Farmer owned field
170999	2017	Hunan	Rice	*F. asiaticum*	15ADON	28.12392502	112.623662	173.31	Farmer owned field
170998	2017	Hunan	Rice	*F. asiaticum*	15ADON	28.12392502	112.623662	173.31	Farmer owned field
170997	2017	Hunan	Rice	*F. asiaticum*	3ADON	28.12392502	112.623662	173.31	Farmer owned field
170996	2017	Hunan	Rice	*F. asiaticum*	NIV	28.12392502	112.623662	173.31	Farmer owned field
170995	2017	Hunan	Rice	*F. asiaticum*	NIV	28.12392502	112.623662	173.31	Farmer owned field
170994	2017	Hunan	Rice	*F. asiaticum*	NIV	28.12392502	112.623662	173.31	Farmer owned field

170993	2017	Hunan	Rice	*F. asiaticum*	NIV	28.12392502	112.623662	173.31	Farmer owned field
170992	2017	Hunan	Rice	*F. asiaticum*	15ADON	28.12392502	112.623662	173.31	Farmer owned field
170991	2017	Hunan	Rice	*F. asiaticum*	3ADON	28.12392502	112.623662	173.31	Farmer owned field
170990	2017	Hunan	Rice	*F. asiaticum*	NIV	28.12392502	112.623662	173.31	Farmer owned field
170989	2017	Hunan	Rice	*F. asiaticum*	NIV	28.12392502	112.623662	173.31	Farmer owned field
170988	2017	Hunan	Rice	*F. asiaticum*	NIV	28.12392502	112.623662	173.31	Farmer owned field
170987	2017	Hunan	Rice	*F. asiaticum*	15ADON	28.12392502	112.623662	173.31	Farmer owned field
170986	2017	Hunan	Rice	*F. asiaticum*	15ADON	28.12392502	112.623662	173.31	Farmer owned field
194075	2019	Hunan	Rice	*F. asiaticum*	NIV	28.19698798	112.766222	47.181259	Farmer owned field
194074	2019	Hunan	Rice	*F. asiaticum*	NIV	28.19698798	112.766222	47.181259	Farmer owned field
194073	2019	Hunan	Rice	*F. asiaticum*	3ADON	28.19698798	112.766222	47.181259	Farmer owned field
194072	2019	Hunan	Rice	*F. asiaticum*	NIV	28.19698798	112.766222	47.181259	Farmer owned field
194071	2019	Hunan	Rice	*F. asiaticum*	NIV	28.19698798	112.766222	47.181259	Farmer owned field
194070	2019	Hunan	Rice	*F. asiaticum*	NIV	28.19698798	112.766222	47.181259	Farmer owned field
194069	2019	Hunan	Rice	*F. asiaticum*	NIV	28.19698798	112.766222	47.181259	Farmer owned field
194068	2019	Hunan	Rice	*F. asiaticum*	NIV	28.19698798	112.766222	47.181259	Farmer owned field
194067	2019	Hunan	Rice	*F. asiaticum*	3ADON	28.19698798	112.766222	47.181259	Farmer owned field
193635	2019	Jiangxi	Rice	*F. asiaticum*	NIV	29.44213796	117.38522	49.038155	Farmer owned field
193634	2019	Jiangxi	Rice	*F. asiaticum*	NIV	29.44213796	117.38522	49.038155	Farmer owned field
193510	2019	Jiangxi	Rice	*F. asiaticum*	NIV	29.49758501	115.854249	64.232147	Farmer owned field
193509	2019	Jiangxi	Rice	*F. asiaticum*	NIV	29.49758501	115.854249	64.232147	Farmer owned field
193508	2019	Jiangxi	Rice	*F. asiaticum*	NIV	29.49758501	115.854249	64.232147	Farmer owned field
193507	2019	Jiangxi	Rice	*F. asiaticum*	NIV	29.49758501	115.854249	64.232147	Farmer owned field
193506	2019	Jiangxi	Rice	*F. asiaticum*	NIV	29.49758501	115.854249	64.232147	Farmer owned field
193504	2019	Jiangxi	Rice	*F. asiaticum*	3ADON	29.49758501	115.854249	64.232147	Farmer owned field
193503	2019	Jiangxi	Rice	*F. asiaticum*	3ADON	29.49758501	115.854249	64.232147	Farmer owned field
193502	2019	Jiangxi	Rice	*F. asiaticum*	NIV	29.49758501	115.854249	64.232147	Farmer owned field
193501	2019	Jiangxi	Rice	*F. asiaticum*	3ADON	29.49758501	115.854249	64.232147	Farmer owned field
193500	2019	Jiangxi	Rice	*F. asiaticum*	NIV	29.49758501	115.854249	64.232147	Farmer owned field
193498	2019	Jiangxi	Rice	*F. asiaticum*	NIV	29.49758501	115.854249	64.232147	Farmer owned field
193497	2019	Jiangxi	Rice	*F. asiaticum*	3ADON	29.49758501	115.854249	64.232147	Farmer owned field
193496	2019	Jiangxi	Rice	*F. asiaticum*	NIV	29.49758501	115.854249	64.232147	Farmer owned field
193495	2019	Jiangxi	Rice	*F. asiaticum*	NIV	29.49758501	115.854249	64.232147	Farmer owned field
193494	2019	Jiangxi	Rice	*F. asiaticum*	NIV	29.49758501	115.854249	64.232147	Farmer owned field
193493	2019	Jiangxi	Rice	*F. asiaticum*	NIV	29.49758501	115.854249	64.232147	Farmer owned field

193492	2019	Jiangxi	Rice	*F. asiaticum*	3ADON	29.49758501	115.854249	64.232147	Farmer owned field
193491	2019	Jiangxi	Rice	*F. asiaticum*	NIV	29.49758501	115.854249	64.232147	Farmer owned field
193490	2019	Jiangxi	Rice	*F. asiaticum*	NIV	29.49758501	115.854249	64.232147	Farmer owned field
193488	2019	Jiangxi	Rice	*F. asiaticum*	NIV	29.49758501	115.854249	64.232147	Farmer owned field
193487	2019	Jiangxi	Rice	*F. asiaticum*	NIV	29.49758501	115.854249	64.232147	Farmer owned field
193485	2019	Jiangxi	Rice	*F. asiaticum*	3ADON	29.49758501	115.854249	64.232147	Farmer owned field
193484	2019	Jiangxi	Rice	*F. asiaticum*	15ADON	29.49758501	115.854249	64.232147	Farmer owned field
194066	2019	Hunan	Rice	*F. asiaticum*	NIV	28.19698798	112.766222	47.181259	Farmer owned field
194065	2019	Hunan	Rice	*F. asiaticum*	3ADON	28.19698798	112.766222	47.181259	Farmer owned field
194064	2019	Hunan	Rice	*F. asiaticum*	NIV	28.19698798	112.766222	47.181259	Farmer owned field
194063	2019	Hunan	Rice	*F. asiaticum*	NIV	28.19698798	112.766222	47.181259	Farmer owned field
194062	2019	Hunan	Rice	*F. asiaticum*	NIV	28.19698798	112.766222	47.181259	Farmer owned field
194061	2019	Hunan	Rice	*F. asiaticum*	3ADON	28.19698798	112.766222	47.181259	Farmer owned field
194060	2019	Hunan	Rice	*F. asiaticum*	NIV	28.19698798	112.766222	47.181259	Farmer owned field
194059	2019	Hunan	Rice	*F. asiaticum*	NIV	28.19698798	112.766222	47.181259	Farmer owned field
194058	2019	Hunan	Rice	*F. asiaticum*	NIV	28.19698798	112.766222	47.181259	Farmer owned field
194057	2019	Hunan	Rice	*F. asiaticum*	NIV	28.19698798	112.766222	47.181259	Farmer owned field
193483	2019	Jiangxi	Rice	*F. asiaticum*	NIV	29.49758501	115.854249	64.232147	Farmer owned field
194056	2019	Hunan	Rice	*F. asiaticum*	3ADON	28.19698798	112.766222	47.181259	Farmer owned field
194055	2019	Hunan	Rice	*F. asiaticum*	NIV	28.19698798	112.766222	47.181259	Farmer owned field
194054	2019	Hunan	Rice	*F. asiaticum*	3ADON	28.19698798	112.766222	47.181259	Farmer owned field
170952	2017	Hunan	Rice	*F. asiaticum*	NIV	28.19698798	112.766222	72.16	Farmer owned field
170951	2017	Hunan	Rice	*F. asiaticum*	15ADON	28.19698798	112.766222	72.16	Farmer owned field
170950	2017	Hunan	Rice	*F. asiaticum*	NIV	28.19698798	112.766222	72.16	Farmer owned field
170949	2017	Hunan	Rice	*F. asiaticum*	15ADON	28.19698798	112.766222	72.16	Farmer owned field
170948	2017	Hunan	Rice	*F. asiaticum*	3ADON	28.19698798	112.766222	72.16	Farmer owned field
170947	2017	Hunan	Rice	*F. asiaticum*	3ADON	28.19698798	112.766222	72.16	Farmer owned field
170946	2017	Hunan	Rice	*F. asiaticum*	NIV	28.19698798	112.766222	72.16	Farmer owned field
170945	2017	Hunan	Rice	*F. asiaticum*	NIV	28.19698798	112.766222	72.16	Farmer owned field
193482	2019	Jiangxi	Rice	*F. asiaticum*	NIV	29.49758501	115.854249	64.232147	Farmer owned field
193539	2019	Jiangxi	Rice	*F. asiaticum*	3ADON	29.72240098	116.305288	26.955158	Farmer owned field
193537	2019	Jiangxi	Rice	*F. asiaticum*	3ADON	29.72240098	116.305288	26.955158	Farmer owned field
193536	2019	Jiangxi	Rice	*F. asiaticum*	NIV	29.72240098	116.305288	26.955158	Farmer owned field
193535	2019	Jiangxi	Rice	*F. asiaticum*	NIV	29.72240098	116.305288	26.955158	Farmer owned field
193534	2019	Jiangxi	Rice	*F. asiaticum*	3ADON	29.72240098	116.305288	26.955158	Farmer owned field

193533	2019	Jiangxi	Rice	*F. asiaticum*	NIV	29.72240098	116.305288	26.955158		Farmer owned field
193532	2019	Jiangxi	Rice	*F. asiaticum*	3ADON	29.72240098	116.305288	26.955158		Farmer owned field
193531	2019	Jiangxi	Rice	*F. asiaticum*	15ADON	29.72240098	116.305288	26.955158		Farmer owned field
193530	2019	Jiangxi	Rice	*F. asiaticum*	NIV	29.72240098	116.305288	26.955158		Farmer owned field
193529	2019	Jiangxi	Rice	*F. asiaticum*	NIV	29.72240098	116.305288	26.955158		Farmer owned field
193528	2019	Jiangxi	Rice	*F. asiaticum*	3ADON	29.72240098	116.305288	26.955158		Farmer owned field
193526	2019	Jiangxi	Rice	*F. asiaticum*	3ADON	29.72240098	116.305288	26.955158		Farmer owned field
193525	2019	Jiangxi	Rice	*F. asiaticum*	3ADON	29.72240098	116.305288	26.955158		Farmer owned field
193524	2019	Jiangxi	Rice	*F. asiaticum*	15ADON	29.72240098	116.305288	26.955158		Farmer owned field
193523	2019	Jiangxi	Rice	*F. asiaticum*	NIV	29.72240098	116.305288	26.955158		Farmer owned field
193520	2019	Jiangxi	Rice	*F. asiaticum*	NIV	29.72240098	116.305288	26.955158		Farmer owned field
193518	2019	Jiangxi	Rice	*F. asiaticum*	3ADON	29.72240098	116.305288	26.955158		Farmer owned field
193517	2019	Jiangxi	Rice	*F. asiaticum*	NIV	29.72240098	116.305288	26.955158		Farmer owned field
193516	2019	Jiangxi	Rice	*F. asiaticum*	NIV	29.72240098	116.305288	26.955158		Farmer owned field
193515	2019	Jiangxi	Rice	*F. asiaticum*	3ADON	29.72240098	116.305288	26.955158		Farmer owned field
193513	2019	Jiangxi	Rice	*F. asiaticum*	NIV	29.72240098	116.305288	26.955158		Farmer owned field
193511	2019	Jiangxi	Rice	*F. asiaticum*	NIV	29.72240098	116.305288	26.955158		Farmer owned field
180941	2018	Sichuan	Wheat	*F. asiaticum*	NIV	31.13249601	105.82188	496.201172	Commercialized variety	Farmer owned field
180940	2018	Sichuan	Wheat	*F. asiaticum*	NIV	31.13249601	105.82188	496.201172	Commercialized variety	Farmer owned field
180939	2018	Sichuan	Wheat	*F. asiaticum*	NIV	31.13249601	105.82188	496.201172	Commercialized variety	Farmer owned field
180937	2018	Sichuan	Wheat	*F. asiaticum*	NIV	31.13249601	105.82188	496.201172	Commercialized variety	Farmer owned field
180936	2018	Sichuan	Wheat	*F. asiaticum*	NIV	31.13249601	105.82188	496.201172	Commercialized variety	Farmer owned field
180935	2018	Sichuan	Wheat	*F. asiaticum*	NIV	31.13249601	105.82188	496.201172	Commercialized variety	Farmer owned field
180934	2018	Sichuan	Wheat	*F. asiaticum*	NIV	31.13249601	105.82188	496.201172	Commercialized variety	Farmer owned field
180931	2018	Sichuan	Wheat	*F. asiaticum*	NIV	31.13249601	105.82188	496.201172	Commercialized variety	Farmer owned field
180928	2018	Sichuan	Wheat	*F. asiaticum*	NIV	31.13249601	105.82188	496.201172	Commercialized variety	Farmer owned field
180927	2018	Sichuan	Wheat	*F. asiaticum*	NIV	31.13249601	105.82188	496.201172	Commercialized variety	Farmer owned field
180925	2018	Sichuan	Wheat	*F. asiaticum*	NIV	31.13249601	105.82188	496.201172	Commercialized variety	Farmer owned field
181706	2018	Sichuan	Rice	*F. asiaticum*	NIV	31.13249601	105.82188	496.201172		Farmer owned field
181705	2018	Sichuan	Rice	*F. asiaticum*	NIV	31.13249601	105.82188	496.201172		Farmer owned field
181704	2018	Sichuan	Rice	*F. asiaticum*	NIV	31.13249601	105.82188	496.201172		Farmer owned field
181703	2018	Sichuan	Rice	*F. asiaticum*	NIV	31.13249601	105.82188	496.201172		Farmer owned field
181702	2018	Sichuan	Rice	*F. asiaticum*	NIV	31.13249601	105.82188	496.201172		Farmer owned field
181701	2018	Sichuan	Rice	*F. asiaticum*	NIV	31.13249601	105.82188	496.201172		Farmer owned field
181700	2018	Sichuan	Rice	*F. asiaticum*	NIV	31.13249601	105.82188	496.201172		Farmer owned field

181698	2018	Sichuan	Rice	*F. asiaticum*	NIV	31.13249601	105.82188	496.201172		Farmer owned field
181697	2018	Sichuan	Rice	*F. asiaticum*	NIV	31.13249601	105.82188	496.201172		Farmer owned field
181696	2018	Sichuan	Rice	*F. asiaticum*	NIV	31.13249601	105.82188	496.201172		Farmer owned field
181695	2018	Sichuan	Rice	*F. asiaticum*	NIV	31.13249601	105.82188	496.201172		Farmer owned field
181694	2018	Sichuan	Rice	*F. asiaticum*	NIV	31.13249601	105.82188	496.201172		Farmer owned field
181693	2018	Sichuan	Rice	*F. asiaticum*	NIV	31.13249601	105.82188	496.201172		Farmer owned field
181692	2018	Sichuan	Rice	*F. asiaticum*	NIV	31.13249601	105.82188	496.201172		Farmer owned field
181691	2018	Sichuan	Rice	*F. asiaticum*	NIV	31.13249601	105.82188	496.201172		Farmer owned field
181690	2018	Sichuan	Rice	*F. asiaticum*	NIV	31.13249601	105.82188	496.201172		Farmer owned field
181689	2018	Sichuan	Rice	*F. asiaticum*	NIV	31.13249601	105.82188	496.201172		Farmer owned field
181688	2018	Sichuan	Rice	*F. asiaticum*	NIV	31.13249601	105.82188	496.201172		Farmer owned field
181687	2018	Sichuan	Rice	*F. asiaticum*	NIV	31.13249601	105.82188	496.201172		Farmer owned field
180971	2018	Sichuan	Wheat	*F. asiaticum*	NIV	31.17314799	105.109974	562.97052	Commercialized variety	Farmer owned field
180970	2018	Sichuan	Wheat	*F. asiaticum*	NIV	31.17314799	105.109974	562.97052	Commercialized variety	Farmer owned field
180975	2018	Sichuan	Wheat	*F. asiaticum*	NIV	31.30192	104.935951	522.26239	Commercialized variety	Farmer owned field
180974	2018	Sichuan	Wheat	*F. asiaticum*	NIV	31.30192	104.935951	522.26239	Commercialized variety	Farmer owned field
193800	2019	Sichuan	Rice	*F. asiaticum*	NIV	31.58071702	105.167829	461.370758		Farmer owned field
193794	2019	Sichuan	Rice	*F. asiaticum*	NIV	31.58071702	105.167829	461.370758		Farmer owned field
193793	2019	Sichuan	Rice	*F. asiaticum*	NIV	31.58071702	105.167829	461.370758		Farmer owned field
193792	2019	Sichuan	Rice	*F. asiaticum*	NIV	31.58071702	105.167829	461.370758		Farmer owned field
193790	2019	Sichuan	Rice	*F. asiaticum*	NIV	31.58071702	105.167829	461.370758		Farmer owned field
193419	2019	Sichuan	Rice	*F. asiaticum*	NIV	31.58348297	104.860531	504.207428		Farmer owned field
194656	2019	Sichuan	Wheat	*F. asiaticum*	NIV	31.78453297	104.781081	NA	Commercialized variety	Farmer owned field
140024	2014	Sichuan	Wheat	*F. asiaticum*	NIV	31.80769402	104.749059	609.975159	Commercialized variety	Farmer owned field
140025	2014	Sichuan	Wheat	*F. asiaticum*	NIV	31.80769402	104.749059	609.975159	Commercialized variety	Farmer owned field
194653	2019	Sichuan	Wheat	*F. asiaticum*	NIV	31.80769402	104.749059	609.975159	Commercialized variety	Farmer owned field
194652	2019	Sichuan	Wheat	*F. asiaticum*	NIV	31.80769402	104.749059	609.975159	Commercialized variety	Farmer owned field
194651	2019	Sichuan	Wheat	*F. asiaticum*	NIV	31.80769402	104.749059	609.975159	Commercialized variety	Farmer owned field
194648	2019	Sichuan	Wheat	*F. asiaticum*	NIV	31.80769402	104.749059	609.975159	Commercialized variety	Farmer owned field
194647	2019	Sichuan	Wheat	*F. asiaticum*	NIV	31.80769402	104.749059	609.975159	Commercialized variety	Farmer owned field
180995	2018	Sichuan	Wheat	*F. asiaticum*	NIV	31.80769402	104.749059	609.975159	Commercialized variety	Farmer owned field
180994	2018	Sichuan	Wheat	*F. asiaticum*	NIV	31.80769402	104.749059	609.975159	Commercialized variety	Farmer owned field
180991	2018	Sichuan	Wheat	*F. asiaticum*	NIV	31.80769402	104.749059	609.975159	Commercialized variety	Farmer owned field
180990	2018	Sichuan	Wheat	*F. asiaticum*	NIV	31.80769402	104.749059	609.975159	Commercialized variety	Farmer owned field
180987	2018	Sichuan	Wheat	*F. asiaticum*	NIV	31.80769402	104.749059	609.975159	Commercialized variety	Farmer owned field

180986	2018	Sichuan	Wheat	*F. asiaticum*	NIV	31.80769402	104.749059	609.975159	Commercialized variety	Farmer owned field
180985	2018	Sichuan	Wheat	*F. asiaticum*	NIV	31.80769402	104.749059	609.975159	Commercialized variety	Farmer owned field
194643	2019	Sichuan	Wheat	*F. asiaticum*	NIV	31.89135304	104.792138	603.32428	Commercialized variety	Farmer owned field
194639	2019	Sichuan	Wheat	*F. asiaticum*	NIV	31.89135304	104.792138	603.32428	Commercialized variety	Farmer owned field
194638	2019	Sichuan	Wheat	*F. asiaticum*	NIV	31.89135304	104.792138	603.32428	Commercialized variety	Farmer owned field
194637	2019	Sichuan	Wheat	*F. asiaticum*	NIV	31.89135304	104.792138	603.32428	Commercialized variety	Farmer owned field
194635	2019	Sichuan	Wheat	*F. asiaticum*	NIV	31.89135304	104.792138	603.32428	Commercialized variety	Farmer owned field
180981	2018	Sichuan	Wheat	*F. asiaticum*	NIV	31.89135304	104.792138	603.32428	Commercialized variety	Farmer owned field
180978	2018	Sichuan	Wheat	*F. asiaticum*	NIV	31.89135304	104.792138	603.32428	Commercialized variety	Farmer owned field
180976	2018	Sichuan	Wheat	*F. asiaticum*	NIV	31.89135304	104.792138	603.32428	Commercialized variety	Farmer owned field
181735	2018	Sichuan	Rice	*F. asiaticum*	NIV	31.89135304	104.792138	603.32428		Farmer owned field
181734	2018	Sichuan	Rice	*F. asiaticum*	NIV	31.89135304	104.792138	603.32428		Farmer owned field
181731	2018	Sichuan	Rice	*F. asiaticum*	NIV	31.89135304	104.792138	603.32428		Farmer owned field
181729	2018	Sichuan	Rice	*F. asiaticum*	NIV	31.89135304	104.792138	603.32428		Farmer owned field
181728	2018	Sichuan	Rice	*F. asiaticum*	NIV	31.89135304	104.792138	603.32428		Farmer owned field
181727	2018	Sichuan	Rice	*F. asiaticum*	NIV	31.89135304	104.792138	603.32428		Farmer owned field
181726	2018	Sichuan	Rice	*F. asiaticum*	NIV	31.89135304	104.792138	603.32428		Farmer owned field
181725	2018	Sichuan	Rice	*F. asiaticum*	NIV	31.89135304	104.792138	603.32428		Farmer owned field
181724	2018	Sichuan	Rice	*F. asiaticum*	NIV	31.89135304	104.792138	603.32428		Farmer owned field
181723	2018	Sichuan	Rice	*F. asiaticum*	NIV	31.89135304	104.792138	603.32428		Farmer owned field
181722	2018	Sichuan	Rice	*F. asiaticum*	NIV	31.89135304	104.792138	603.32428		Farmer owned field
181721	2018	Sichuan	Rice	*F. asiaticum*	NIV	31.89135304	104.792138	603.32428		Farmer owned field
14097-2	2014	Hubei	Rice	*F. asiaticum*	3ADON	30.892518	113.933865	24		Commercial farm
14081-2	2014	Hubei	Rice	*F. asiaticum*	NIV	30.892518	113.933865	24		Commercial farm
14080-2	2014	Hubei	Rice	*F. asiaticum*	3ADON	30.892518	113.933865	24		Commercial farm
14077-2	2014	Hubei	Rice	*F. asiaticum*	NIV	30.892518	113.933865	24		Commercial farm
14062-2	2014	Hubei	Rice	*F. asiaticum*	NIV	30.892518	113.933865	24		Commercial farm
14058-2	2014	Hubei	Rice	*F. asiaticum*	3ADON	30.892518	113.933865	24		Commercial farm
14013-2	2014	Hubei	Rice	*F. asiaticum*	3ADON	30.892518	113.933865	24		Commercial farm
14010-2	2014	Hubei	Rice	*F. asiaticum*	NIV	30.892518	113.933865	24		Commercial farm
14010-2	2014	Hubei	Rice	*F. asiaticum*	NIV	30.892518	113.933865	24		Commercial farm
14122	2014	Hubei	Rice	*F. asiaticum*	3ADON	30.892518	113.933865	24		Commercial farm
14116	2014	Hubei	Rice	*F. asiaticum*	3ADON	30.892518	113.933865	24		Commercial farm
14114	2014	Hubei	Rice	*F. asiaticum*	NIV	30.892518	113.933865	24		Commercial farm
14110	2014	Hubei	Rice	*F. asiaticum*	3ADON	30.892518	113.933865	24		Commercial farm

14109	2014	Hubei	Rice	*F. asiaticum*	15ADON	30.892518	113.933865	24	Commercial farm
14106	2014	Hubei	Rice	*F. asiaticum*	3ADON	30.892518	113.933865	24	Commercial farm
14104	2014	Hubei	Rice	*F. asiaticum*	3ADON	30.892518	113.933865	24	Commercial farm
14098	2014	Hubei	Rice	*F. asiaticum*	3ADON	30.892518	113.933865	24	Commercial farm
14096	2014	Hubei	Rice	*F. asiaticum*	3ADON	30.892518	113.933865	24	Commercial farm
14096	2014	Hubei	Rice	*F. asiaticum*	NIV	30.892518	113.933865	24	Commercial farm
14092	2014	Hubei	Rice	*F. asiaticum*	3ADON	30.892518	113.933865	24	Commercial farm
14090	2014	Hubei	Rice	*F. asiaticum*	3ADON	30.892518	113.933865	24	Commercial farm
14089	2014	Hubei	Rice	*F. asiaticum*	NIV	30.892518	113.933865	24	Commercial farm
14088	2014	Hubei	Rice	*F. asiaticum*	3ADON	30.892518	113.933865	24	Commercial farm
14083	2014	Hubei	Rice	*F. asiaticum*	NIV	30.892518	113.933865	24	Commercial farm
14081	2014	Hubei	Rice	*F. asiaticum*	NIV	30.892518	113.933865	24	Commercial farm
14077	2014	Hubei	Rice	*F. asiaticum*	NIV	30.892518	113.933865	24	Commercial farm
14076	2014	Hubei	Rice	*F. asiaticum*	NIV	30.892518	113.933865	24	Commercial farm
14075	2014	Hubei	Rice	*F. asiaticum*	15ADON	30.892518	113.933865	24	Commercial farm
14069	2014	Hubei	Rice	*F. asiaticum*	3ADON	30.892518	113.933865	24	Commercial farm
14068	2014	Hubei	Rice	*F. asiaticum*	3ADON	30.892518	113.933865	24	Commercial farm
14066	2014	Hubei	Rice	*F. asiaticum*	3ADON	30.892518	113.933865	24	Commercial farm
14065	2014	Hubei	Rice	*F. asiaticum*	3ADON	30.892518	113.933865	24	Commercial farm
14064	2014	Hubei	Rice	*F. asiaticum*	3ADON	30.892518	113.933865	24	Commercial farm
14062	2014	Hubei	Rice	*F. asiaticum*	NIV	30.892518	113.933865	24	Commercial farm
14060	2014	Hubei	Rice	*F. asiaticum*	3ADON	30.892518	113.933865	24	Commercial farm
14057	2014	Hubei	Rice	*F. asiaticum*	NIV	30.892518	113.933865	24	Commercial farm
14055	2014	Hubei	Rice	*F. asiaticum*	3ADON	30.892518	113.933865	24	Commercial farm
14047	2014	Hubei	Rice	*F. asiaticum*	3ADON	30.892518	113.933865	24	Commercial farm
14045	2014	Hubei	Rice	*F. asiaticum*	NIV	30.892518	113.933865	24	Commercial farm
14041	2014	Hubei	Rice	*F. asiaticum*	3ADON	30.892518	113.933865	24	Commercial farm
14039	2014	Hubei	Rice	*F. asiaticum*	3ADON	30.892518	113.933865	24	Commercial farm
14038	2014	Hubei	Rice	*F. asiaticum*	NIV	30.892518	113.933865	24	Commercial farm
14030	2014	Hubei	Rice	*F. asiaticum*	NIV	30.892518	113.933865	24	Commercial farm
14029	2014	Hubei	Rice	*F. asiaticum*	NIV	30.892518	113.933865	24	Commercial farm
14028	2014	Hubei	Rice	*F. asiaticum*	3ADON	30.892518	113.933865	24	Commercial farm
14027	2014	Hubei	Rice	*F. asiaticum*	3ADON	30.892518	113.933865	24	Commercial farm
14024	2014	Hubei	Rice	*F. asiaticum*	NIV	30.892518	113.933865	24	Commercial farm
14022	2014	Hubei	Rice	*F. asiaticum*	3ADON	30.892518	113.933865	24	Commercial farm

14021	2014	Hubei	Rice	*F. asiaticum*	3ADON	30.892518	113.933865	24		Commercial farm
14019	2014	Hubei	Rice	*F. asiaticum*	3ADON	30.892518	113.933865	24		Commercial farm
14012	2014	Hubei	Rice	*F. asiaticum*	NIV	30.892518	113.933865	24		Commercial farm
14007	2014	Hubei	Rice	*F. asiaticum*	NIV	30.892518	113.933865	24		Commercial farm
14002	2014	Hubei	Rice	*F. asiaticum*	15ADON	30.892518	113.933865	24		Commercial farm
140001	2014	Hubei	Rice	*F. asiaticum*	3ADON	30.892518	113.933865	24		Commercial farm
140002	2014	Hubei	Rice	*F. asiaticum*	3ADON	30.892518	113.933865	24		Commercial farm
140003	2014	Hubei	Rice	*F. asiaticum*	3ADON	30.892518	113.933865	24		Commercial farm
140004	2014	Hubei	Rice	*F. asiaticum*	3ADON	30.892518	113.933865	24		Commercial farm
140015	2014	Hubei	Wheat	*F. asiaticum*	3ADON	30.892518	113.933865	24	Commercialized variety	Commercial farm
140016	2014	Hubei	Wheat	*F. asiaticum*	3ADON	30.892518	113.933865	24	Commercialized variety	Commercial farm
140017	2014	Hubei	Wheat	*F. asiaticum*	3ADON	30.892518	113.933865	24	Commercialized variety	Commercial farm
140018	2014	Hubei	Wheat	*F. asiaticum*	3ADON	30.892518	113.933865	24	Commercialized variety	Commercial farm
140019	2014	Hubei	Wheat	*F. asiaticum*	3ADON	30.892518	113.933865	24	Commercialized variety	Commercial farm
140020	2014	Hubei	Wheat	*F. asiaticum*	3ADON	30.892518	113.933865	24	Commercialized variety	Commercial farm
140021	2014	Hubei	Wheat	*F. asiaticum*	3ADON	30.892518	113.933865	24	Commercialized variety	Commercial farm
140022	2014	Hubei	Wheat	*F. asiaticum*	3ADON	30.892518	113.933865	24	Commercialized variety	Commercial farm
140023	2014	Hubei	Wheat	*F. asiaticum*	3ADON	30.892518	113.933865	24	Commercialized variety	Commercial farm
183593	2018	Hubei	Wheat	*F. asiaticum*	3ADON	30.922397	112.539262	NA	Commercialized variety	Farmer owned field
183592	2018	Hubei	Wheat	*F. asiaticum*	3ADON	30.922397	112.539262	NA	Commercialized variety	Farmer owned field
183591	2018	Hubei	Wheat	*F. asiaticum*	3ADON	30.922397	112.539262	NA	Commercialized variety	Farmer owned field
183590	2018	Hubei	Wheat	*F. asiaticum*	3ADON	30.922397	112.539262	NA	Commercialized variety	Farmer owned field
183589	2018	Hubei	Wheat	*F. asiaticum*	3ADON	30.922397	112.539262	NA	Commercialized variety	Farmer owned field
183588	2018	Hubei	Wheat	*F. asiaticum*	3ADON	30.922397	112.539262	NA	Commercialized variety	Farmer owned field
183587	2018	Hubei	Wheat	*F. asiaticum*	3ADON	30.922397	112.539262	NA	Commercialized variety	Farmer owned field
183586	2018	Hubei	Wheat	*F. asiaticum*	3ADON	30.922397	112.539262	NA	Commercialized variety	Farmer owned field
183585	2018	Hubei	Wheat	*F. asiaticum*	3ADON	30.922397	112.539262	NA	Commercialized variety	Farmer owned field
180418	2018	Hubei	Rice	*F. asiaticum*	3ADON	30.93555498	113.873914	27.244297		Farmer owned field
180417	2018	Hubei	Rice	*F. asiaticum*	3ADON	30.93555498	113.873914	27.244297		Farmer owned field
180416	2018	Hubei	Rice	*F. asiaticum*	3ADON	30.93555498	113.873914	27.244297		Farmer owned field
180415	2018	Hubei	Rice	*F. asiaticum*	3ADON	30.93555498	113.873914	27.244297		Farmer owned field
180414	2018	Hubei	Rice	*F. asiaticum*	3ADON	30.93555498	113.873914	27.244297		Farmer owned field
180413	2018	Hubei	Rice	*F. asiaticum*	NIV	30.93555498	113.873914	27.244297		Farmer owned field
180412	2018	Hubei	Rice	*F. asiaticum*	3ADON	30.93555498	113.873914	27.244297		Farmer owned field
180411	2018	Hubei	Rice	*F. asiaticum*	3ADON	30.93555498	113.873914	27.244297		Farmer owned field

180410	2018	Hubei	Rice	*F. asiaticum*	3ADON	30.93555498	113.873914	27.244297		Farmer owned field
180409	2018	Hubei	Rice	*F. asiaticum*	3ADON	30.93555498	113.873914	27.244297		Farmer owned field
180408	2018	Hubei	Rice	*F. asiaticum*	NIV	30.93555498	113.873914	27.244297		Farmer owned field
180407	2018	Hubei	Rice	*F. asiaticum*	NIV	30.93555498	113.873914	27.244297		Farmer owned field
180406	2018	Hubei	Rice	*F. asiaticum*	3ADON	30.93555498	113.873914	27.244297		Farmer owned field
180405	2018	Hubei	Rice	*F. asiaticum*	3ADON	30.93555498	113.873914	27.244297		Farmer owned field
180404	2018	Hubei	Rice	*F. asiaticum*	NIV	30.93555498	113.873914	27.244297		Farmer owned field
180403	2018	Hubei	Rice	*F. asiaticum*	NIV	30.93555498	113.873914	27.244297		Farmer owned field
180402	2018	Hubei	Rice	*F. asiaticum*	NIV	30.93555498	113.873914	27.244297		Farmer owned field
180401	2018	Hubei	Rice	*F. asiaticum*	NIV	30.93555498	113.873914	27.244297		Farmer owned field
180400	2018	Hubei	Rice	*F. asiaticum*	NIV	30.93555498	113.873914	27.244297		Farmer owned field
180399	2018	Hubei	Rice	*F. asiaticum*	3ADON	30.93555498	113.873914	27.244297		Farmer owned field
180398	2018	Hubei	Rice	*F. asiaticum*	3ADON	30.93555498	113.873914	27.244297		Farmer owned field
180397	2018	Hubei	Rice	*F. asiaticum*	3ADON	30.93555498	113.873914	27.244297		Farmer owned field
180396	2018	Hubei	Rice	*F. asiaticum*	3ADON	30.93555498	113.873914	27.244297		Farmer owned field
180395	2018	Hubei	Rice	*F. asiaticum*	3ADON	30.93555498	113.873914	27.244297		Farmer owned field
180394	2018	Hubei	Rice	*F. asiaticum*	3ADON	30.93555498	113.873914	27.244297		Farmer owned field
180393	2018	Hubei	Rice	*F. asiaticum*	3ADON	30.93555498	113.873914	27.244297		Farmer owned field
180392	2018	Hubei	Rice	*F. asiaticum*	3ADON	30.93555498	113.873914	27.244297		Farmer owned field
180391	2018	Hubei	Rice	*F. asiaticum*	NIV	30.93555498	113.873914	27.244297		Farmer owned field
180390	2018	Hubei	Rice	*F. asiaticum*	3ADON	30.93555498	113.873914	27.244297		Farmer owned field
180389	2018	Hubei	Rice	*F. asiaticum*	3ADON	30.93555498	113.873914	27.244297		Farmer owned field
180388	2018	Hubei	Rice	*F. asiaticum*	3ADON	30.93555498	113.873914	27.244297		Farmer owned field
180387	2018	Hubei	Rice	*F. asiaticum*	3ADON	30.93555498	113.873914	27.244297		Farmer owned field
180386	2018	Hubei	Rice	*F. asiaticum*	3ADON	30.93555498	113.873914	27.244297		Farmer owned field
180385	2018	Hubei	Rice	*F. asiaticum*	3ADON	30.93555498	113.873914	27.244297		Farmer owned field
180384	2018	Hubei	Rice	*F. asiaticum*	3ADON	30.93555498	113.873914	27.244297		Farmer owned field
180383	2018	Hubei	Rice	*F. asiaticum*	3ADON	30.93555498	113.873914	27.244297		Farmer owned field
180382	2018	Hubei	Rice	*F. asiaticum*	3ADON	30.93555498	113.873914	27.244297		Farmer owned field
180381	2018	Hubei	Rice	*F. asiaticum*	3ADON	30.93555498	113.873914	27.244297		Farmer owned field
180448	2018	Hubei	Rice	*F. asiaticum*	3ADON	30.93952197	113.853011	31.46199		Farmer owned field
180447	2018	Hubei	Rice	*F. asiaticum*	3ADON	30.93952197	113.853011	31.46199		Farmer owned field
180446	2018	Hubei	Rice	*F. asiaticum*	3ADON	30.93952197	113.853011	31.46199		Farmer owned field
180445	2018	Hubei	Rice	*F. asiaticum*	NIV	30.93952197	113.853011	31.46199		Farmer owned field
180444	2018	Hubei	Rice	*F. asiaticum*	3ADON	30.93952197	113.853011	31.46199		Farmer owned field

180443	2018	Hubei	Rice	*F. asiaticum*	NIV	30.93952197	113.853011	31.46199		Farmer owned field
180442	2018	Hubei	Rice	*F. asiaticum*	NIV	30.93952197	113.853011	31.46199		Farmer owned field
180441	2018	Hubei	Rice	*F. asiaticum*	NIV	30.93952197	113.853011	31.46199		Farmer owned field
180440	2018	Hubei	Rice	*F. asiaticum*	3ADON	30.93952197	113.853011	31.46199		Farmer owned field
180439	2018	Hubei	Rice	*F. asiaticum*	3ADON	30.93952197	113.853011	31.46199		Farmer owned field
180438	2018	Hubei	Rice	*F. asiaticum*	NIV	30.93952197	113.853011	31.46199		Farmer owned field
180437	2018	Hubei	Rice	*F. asiaticum*	NIV	30.93952197	113.853011	31.46199		Farmer owned field
180436	2018	Hubei	Rice	*F. asiaticum*	NIV	30.93952197	113.853011	31.46199		Farmer owned field
180435	2018	Hubei	Rice	*F. asiaticum*	NIV	30.93952197	113.853011	31.46199		Farmer owned field
180434	2018	Hubei	Rice	*F. asiaticum*	NIV	30.93952197	113.853011	31.46199		Farmer owned field
180433	2018	Hubei	Rice	*F. asiaticum*	3ADON	30.93952197	113.853011	31.46199		Farmer owned field
180432	2018	Hubei	Rice	*F. asiaticum*	3ADON	30.93952197	113.853011	31.46199		Farmer owned field
180431	2018	Hubei	Rice	*F. asiaticum*	NIV	30.93952197	113.853011	31.46199		Farmer owned field
180430	2018	Hubei	Rice	*F. asiaticum*	NIV	30.93952197	113.853011	31.46199		Farmer owned field
180429	2018	Hubei	Rice	*F. asiaticum*	3ADON	30.93952197	113.853011	31.46199		Farmer owned field
180428	2018	Hubei	Rice	*F. asiaticum*	3ADON	30.93952197	113.853011	31.46199		Farmer owned field
180427	2018	Hubei	Rice	*F. asiaticum*	NIV	30.93952197	113.853011	31.46199		Farmer owned field
180426	2018	Hubei	Rice	*F. asiaticum*	3ADON	30.93952197	113.853011	31.46199		Farmer owned field
180425	2018	Hubei	Rice	*F. asiaticum*	NIV	30.93952197	113.853011	31.46199		Farmer owned field
180423	2018	Hubei	Rice	*F. asiaticum*	3ADON	30.93952197	113.853011	31.46199		Farmer owned field
180422	2018	Hubei	Rice	*F. asiaticum*	3ADON	30.93952197	113.853011	31.46199		Farmer owned field
180421	2018	Hubei	Rice	*F. asiaticum*	3ADON	30.93952197	113.853011	31.46199		Farmer owned field
180420	2018	Hubei	Rice	*F. asiaticum*	NIV	30.93952197	113.853011	31.46199		Farmer owned field
192041	2019	Hubei	Wheat	*F. asiaticum*	3ADON	30.96510898	112.474366	85.33107	Commercialized variety	Farmer owned field
192040	2019	Hubei	Wheat	*F. asiaticum*	3ADON	30.96510898	112.474366	85.33107	Commercialized variety	Farmer owned field
192039	2019	Hubei	Wheat	*F. asiaticum*	3ADON	30.96510898	112.474366	85.33107	Commercialized variety	Farmer owned field
192037	2019	Hubei	Wheat	*F. asiaticum*	3ADON	30.96510898	112.474366	85.33107	Commercialized variety	Farmer owned field
192036	2019	Hubei	Wheat	*F. asiaticum*	3ADON	30.96510898	112.474366	85.33107	Commercialized variety	Farmer owned field
192035	2019	Hubei	Wheat	*F. asiaticum*	3ADON	30.96510898	112.474366	85.33107	Commercialized variety	Farmer owned field
192033	2019	Hubei	Wheat	*F. asiaticum*	3ADON	30.96510898	112.474366	85.33107	Commercialized variety	Farmer owned field
192032	2019	Hubei	Wheat	*F. asiaticum*	3ADON	30.96510898	112.474366	85.33107	Commercialized variety	Farmer owned field
192030	2019	Hubei	Wheat	*F. asiaticum*	3ADON	30.96510898	112.474366	85.33107	Commercialized variety	Farmer owned field
192029	2019	Hubei	Wheat	*F. asiaticum*	3ADON	30.96510898	112.474366	85.33107	Commercialized variety	Farmer owned field
192028	2019	Hubei	Wheat	*F. asiaticum*	3ADON	30.96510898	112.474366	85.33107	Commercialized variety	Farmer owned field
192027	2019	Hubei	Wheat	*F. asiaticum*	15ADON	30.96510898	112.474366	85.33107	Commercialized variety	Farmer owned field

192025	2019	Hubei	Wheat	*F. asiaticum*	3ADON	30.96510898	112.474366	85.33107	Commercialized variety	Farmer owned field
192023	2019	Hubei	Wheat	*F. asiaticum*	3ADON	30.96510898	112.474366	85.33107	Commercialized variety	Farmer owned field
181132	2018	Hubei	Wheat	*F. asiaticum*	3ADON	31.04326101	112.282253	86.576973	Commercialized variety	Farmer owned field
181131	2018	Hubei	Wheat	*F. asiaticum*	3ADON	31.04326101	112.282253	86.576973	Commercialized variety	Farmer owned field
181130	2018	Hubei	Wheat	*F. asiaticum*	NIV	31.04326101	112.282253	86.576973	Commercialized variety	Farmer owned field
181129	2018	Hubei	Wheat	*F. asiaticum*	NIV	31.04326101	112.282253	86.576973	Commercialized variety	Farmer owned field
181128	2018	Hubei	Wheat	*F. asiaticum*	15ADON	31.04326101	112.282253	86.576973	Commercialized variety	Farmer owned field
181127	2018	Hubei	Wheat	*F. asiaticum*	NIV	31.04326101	112.282253	86.576973	Commercialized variety	Farmer owned field
181126	2018	Hubei	Wheat	*F. asiaticum*	NIV	31.04326101	112.282253	86.576973	Commercialized variety	Farmer owned field
181125	2018	Hubei	Wheat	*F. asiaticum*	3ADON	31.04326101	112.282253	86.576973	Commercialized variety	Farmer owned field
181124	2018	Hubei	Wheat	*F. asiaticum*	3ADON	31.04326101	112.282253	86.576973	Commercialized variety	Farmer owned field
183755	2018	Hubei	Wheat	*F. asiaticum*	15ADON	31.168547	112.521419	NA	Commercialized variety	Farmer owned field
183754	2018	Hubei	Wheat	*F. asiaticum*	15ADON	31.168547	112.521419	NA	Commercialized variety	Farmer owned field
191433	2019	Hubei	Wheat	*F. asiaticum*	3ADON	31.27414204	112.328259	112.179672	Commercialized variety	Farmer owned field
191428	2019	Hubei	Wheat	*F. asiaticum*	NIV	31.27414204	112.328259	112.179672	Commercialized variety	Farmer owned field
191427	2019	Hubei	Wheat	*F. asiaticum*	15ADON	31.27414204	112.328259	112.179672	Commercialized variety	Farmer owned field
191420	2019	Hubei	Wheat	*F. asiaticum*	15ADON	31.27414204	112.328259	112.179672	Commercialized variety	Farmer owned field
191416	2019	Hubei	Wheat	*F. asiaticum*	NIV	31.27414204	112.328259	112.179672	Commercialized variety	Farmer owned field
192022	2019	Hubei	Wheat	*F. asiaticum*	3ADON	31.59498998	113.318392	139.44841	Commercialized variety	Farmer owned field
192021	2019	Hubei	Wheat	*F. asiaticum*	NIV	31.59498998	113.318392	139.44841	Commercialized variety	Farmer owned field
192020	2019	Hubei	Wheat	*F. asiaticum*	3ADON	31.59498998	113.318392	139.44841	Commercialized variety	Farmer owned field
192019	2019	Hubei	Wheat	*F. asiaticum*	15ADON	31.59498998	113.318392	139.44841	Commercialized variety	Farmer owned field
192018	2019	Hubei	Wheat	*F. asiaticum*	3ADON	31.59498998	113.318392	139.44841	Commercialized variety	Farmer owned field
192017	2019	Hubei	Wheat	*F. asiaticum*	3ADON	31.59498998	113.318392	139.44841	Commercialized variety	Farmer owned field
192016	2019	Hubei	Wheat	*F. asiaticum*	3ADON	31.59498998	113.318392	139.44841	Commercialized variety	Farmer owned field
192015	2019	Hubei	Wheat	*F. asiaticum*	NIV	31.59498998	113.318392	139.44841	Commercialized variety	Farmer owned field
192014	2019	Hubei	Wheat	*F. asiaticum*	15ADON	31.59498998	113.318392	139.44841	Commercialized variety	Farmer owned field
192013	2019	Hubei	Wheat	*F. asiaticum*	3ADON	31.59498998	113.318392	139.44841	Commercialized variety	Farmer owned field
192012	2019	Hubei	Wheat	*F. asiaticum*	3ADON	31.59498998	113.318392	139.44841	Commercialized variety	Farmer owned field
192011	2019	Hubei	Wheat	*F. asiaticum*	3ADON	31.59498998	113.318392	139.44841	Commercialized variety	Farmer owned field
192010	2019	Hubei	Wheat	*F. asiaticum*	3ADON	31.59498998	113.318392	139.44841	Commercialized variety	Farmer owned field
192009	2019	Hubei	Wheat	*F. asiaticum*	NIV	31.59498998	113.318392	139.44841	Commercialized variety	Farmer owned field
192008	2019	Hubei	Wheat	*F. asiaticum*	3ADON	31.59498998	113.318392	139.44841	Commercialized variety	Farmer owned field
192007	2019	Hubei	Wheat	*F. asiaticum*	NIV	31.59498998	113.318392	139.44841	Commercialized variety	Farmer owned field
192006	2019	Hubei	Wheat	*F. asiaticum*	3ADON	31.59498998	113.318392	139.44841	Commercialized variety	Farmer owned field

192005	2019	Hubei	Wheat	*F. asiaticum*	NIV	31.59498998	113.318392	139.44841	Commercialized variety	Farmer owned field
192004	2019	Hubei	Wheat	*F. asiaticum*	3ADON	31.59498998	113.318392	139.44841	Commercialized variety	Farmer owned field
192003	2019	Hubei	Wheat	*F. asiaticum*	3ADON	31.59498998	113.318392	139.44841	Commercialized variety	Farmer owned field
192002	2019	Hubei	Wheat	*F. asiaticum*	3ADON	31.59498998	113.318392	139.44841	Commercialized variety	Farmer owned field
192001	2019	Hubei	Wheat	*F. asiaticum*	3ADON	31.59498998	113.318392	139.44841	Commercialized variety	Farmer owned field
192000	2019	Hubei	Wheat	*F. asiaticum*	3ADON	31.59498998	113.318392	139.44841	Commercialized variety	Farmer owned field
191999	2019	Hubei	Wheat	*F. asiaticum*	3ADON	31.59498998	113.318392	139.44841	Commercialized variety	Farmer owned field
191998	2019	Hubei	Wheat	*F. asiaticum*	3ADON	31.59498998	113.318392	139.44841	Commercialized variety	Farmer owned field
191997	2019	Hubei	Wheat	*F. asiaticum*	3ADON	31.59498998	113.318392	139.44841	Commercialized variety	Farmer owned field
191996	2019	Hubei	Wheat	*F. asiaticum*	NIV	31.59498998	113.318392	139.44841	Commercialized variety	Farmer owned field
180625	2018	Hubei	Rice	*F. asiaticum*	3ADON	31.66113703	112.234185	88.139984		Farmer owned field
180624	2018	Hubei	Rice	*F. asiaticum*	3ADON	31.66113703	112.234185	88.139984		Farmer owned field
180623	2018	Hubei	Rice	*F. asiaticum*	3ADON	31.66113703	112.234185	88.139984		Farmer owned field
180622	2018	Hubei	Rice	*F. asiaticum*	3ADON	31.66113703	112.234185	88.139984		Farmer owned field
180620	2018	Hubei	Rice	*F. asiaticum*	3ADON	31.66113703	112.234185	88.139984		Farmer owned field
180619	2018	Hubei	Rice	*F. asiaticum*	3ADON	31.66113703	112.234185	88.139984		Farmer owned field
180618	2018	Hubei	Rice	*F. asiaticum*	3ADON	31.66113703	112.234185	88.139984		Farmer owned field
180617	2018	Hubei	Rice	*F. asiaticum*	3ADON	31.66113703	112.234185	88.139984		Farmer owned field
180616	2018	Hubei	Rice	*F. asiaticum*	3ADON	31.66113703	112.234185	88.139984		Farmer owned field
180615	2018	Hubei	Rice	*F. asiaticum*	3ADON	31.66113703	112.234185	88.139984		Farmer owned field
180614	2018	Hubei	Rice	*F. asiaticum*	3ADON	31.66113703	112.234185	88.139984		Farmer owned field
180613	2018	Hubei	Rice	*F. asiaticum*	3ADON	31.66113703	112.234185	88.139984		Farmer owned field
180612	2018	Hubei	Rice	*F. asiaticum*	3ADON	31.66113703	112.234185	88.139984		Farmer owned field
180611	2018	Hubei	Rice	*F. asiaticum*	3ADON	31.66113703	112.234185	88.139984		Farmer owned field
180610	2018	Hubei	Rice	*F. asiaticum*	3ADON	31.66113703	112.234185	88.139984		Farmer owned field
180609	2018	Hubei	Rice	*F. asiaticum*	3ADON	31.66113703	112.234185	88.139984		Farmer owned field
180608	2018	Hubei	Rice	*F. asiaticum*	3ADON	31.66113703	112.234185	88.139984		Farmer owned field
180607	2018	Hubei	Rice	*F. asiaticum*	3ADON	31.66113703	112.234185	88.139984		Farmer owned field
180606	2018	Hubei	Rice	*F. asiaticum*	3ADON	31.66113703	112.234185	88.139984		Farmer owned field
180605	2018	Hubei	Rice	*F. asiaticum*	3ADON	31.66113703	112.234185	88.139984		Farmer owned field
180604	2018	Hubei	Rice	*F. asiaticum*	3ADON	31.66113703	112.234185	88.139984		Farmer owned field
180603	2018	Hubei	Rice	*F. asiaticum*	NIV	31.66113703	112.234185	88.139984		Farmer owned field
180602	2018	Hubei	Rice	*F. asiaticum*	15ADON	31.66113703	112.234185	88.139984		Farmer owned field
180601	2018	Hubei	Rice	*F. asiaticum*	NIV	31.66113703	112.234185	88.139984		Farmer owned field
180600	2018	Hubei	Rice	*F. asiaticum*	15ADON	31.66113703	112.234185	88.139984		Farmer owned field

180599	2018	Hubei	Rice	*F. asiaticum*	15ADON	31.66113703	112.234185	88.139984		Farmer owned field
180598	2018	Hubei	Rice	*F. asiaticum*	3ADON	31.66113703	112.234185	88.139984		Farmer owned field
180597	2018	Hubei	Rice	*F. asiaticum*	3ADON	31.66113703	112.234185	88.139984		Farmer owned field
180596	2018	Hubei	Rice	*F. asiaticum*	3ADON	31.66113703	112.234185	88.139984		Farmer owned field
180595	2018	Hubei	Rice	*F. asiaticum*	3ADON	31.66113703	112.234185	88.139984		Farmer owned field
180594	2018	Hubei	Rice	*F. asiaticum*	3ADON	31.66113703	112.234185	88.139984		Farmer owned field
180593	2018	Hubei	Rice	*F. asiaticum*	3ADON	31.66113703	112.234185	88.139984		Farmer owned field
180592	2018	Hubei	Rice	*F. asiaticum*	3ADON	31.66113703	112.234185	88.139984		Farmer owned field
180591	2018	Hubei	Rice	*F. asiaticum*	NIV	31.66113703	112.234185	88.139984		Farmer owned field
180589	2018	Hubei	Rice	*F. asiaticum*	3ADON	31.66113703	112.234185	88.139984		Farmer owned field
180588	2018	Hubei	Rice	*F. asiaticum*	3ADON	31.66113703	112.234185	88.139984		Farmer owned field
180587	2018	Hubei	Rice	*F. asiaticum*	3ADON	31.66113703	112.234185	88.139984		Farmer owned field
180586	2018	Hubei	Rice	*F. asiaticum*	3ADON	31.66113703	112.234185	88.139984		Farmer owned field
180585	2018	Hubei	Rice	*F. asiaticum*	3ADON	31.66113703	112.234185	88.139984		Farmer owned field
191798	2019	Hubei	Wheat	*F. asiaticum*	3ADON	31.66168998	112.270017	81.660889	Commercialized variety	Farmer owned field
191796	2019	Hubei	Wheat	*F. asiaticum*	15ADON	31.66168998	112.270017	81.660889	Commercialized variety	Farmer owned field
191794	2019	Hubei	Wheat	*F. asiaticum*	3ADON	31.66168998	112.270017	81.660889	Commercialized variety	Farmer owned field
191793	2019	Hubei	Wheat	*F. asiaticum*	3ADON	31.66168998	112.270017	81.660889	Commercialized variety	Farmer owned field
191792	2019	Hubei	Wheat	*F. asiaticum*	3ADON	31.66168998	112.270017	81.660889	Commercialized variety	Farmer owned field
191790	2019	Hubei	Wheat	*F. asiaticum*	3ADON	31.66168998	112.270017	81.660889	Commercialized variety	Farmer owned field
191789	2019	Hubei	Wheat	*F. asiaticum*	3ADON	31.66168998	112.270017	81.660889	Commercialized variety	Farmer owned field
191788	2019	Hubei	Wheat	*F. asiaticum*	3ADON	31.66168998	112.270017	81.660889	Commercialized variety	Farmer owned field
191787	2019	Hubei	Wheat	*F. asiaticum*	3ADON	31.66168998	112.270017	81.660889	Commercialized variety	Farmer owned field
191786	2019	Hubei	Wheat	*F. asiaticum*	3ADON	31.66168998	112.270017	81.660889	Commercialized variety	Farmer owned field
181143	2018	Hubei	Wheat	*F. asiaticum*	3ADON	31.66168998	112.270017	81.660889	Commercialized variety	Farmer owned field
181142	2018	Hubei	Wheat	*F. asiaticum*	3ADON	31.66168998	112.270017	81.660889	Commercialized variety	Farmer owned field
181140	2018	Hubei	Wheat	*F. asiaticum*	3ADON	31.66168998	112.270017	81.660889	Commercialized variety	Farmer owned field
181137	2018	Hubei	Wheat	*F. asiaticum*	3ADON	31.66168998	112.270017	81.660889	Commercialized variety	Farmer owned field
181136	2018	Hubei	Wheat	*F. asiaticum*	3ADON	31.66168998	112.270017	81.660889	Commercialized variety	Farmer owned field
181135	2018	Hubei	Wheat	*F. asiaticum*	3ADON	31.66168998	112.270017	81.660889	Commercialized variety	Farmer owned field
181134	2018	Hubei	Wheat	*F. asiaticum*	3ADON	31.66168998	112.270017	81.660889	Commercialized variety	Farmer owned field
181133	2018	Hubei	Wheat	*F. asiaticum*	3ADON	31.66168998	112.270017	81.660889	Commercialized variety	Farmer owned field
191915	2019	Hubei	Wheat	*F. asiaticum*	3ADON	31.80608704	111.937191	156.221863	Commercialized variety	Farmer owned field
191914	2019	Hubei	Wheat	*F. asiaticum*	3ADON	31.80608704	111.937191	156.221863	Commercialized variety	Farmer owned field
191913	2019	Hubei	Wheat	*F. asiaticum*	3ADON	31.80608704	111.937191	156.221863	Commercialized variety	Farmer owned field

191912	2019	Hubei	Wheat	*F. asiaticum*	3ADON	31.80608704	111.937191	156.221863	Commercialized variety	Farmer owned field
191911	2019	Hubei	Wheat	*F. asiaticum*	3ADON	31.80608704	111.937191	156.221863	Commercialized variety	Farmer owned field
191910	2019	Hubei	Wheat	*F. asiaticum*	3ADON	31.80608704	111.937191	156.221863	Commercialized variety	Farmer owned field
191909	2019	Hubei	Wheat	*F. asiaticum*	3ADON	31.80608704	111.937191	156.221863	Commercialized variety	Farmer owned field
191908	2019	Hubei	Wheat	*F. asiaticum*	3ADON	31.80608704	111.937191	156.221863	Commercialized variety	Farmer owned field
191907	2019	Hubei	Wheat	*F. asiaticum*	3ADON	31.80608704	111.937191	156.221863	Commercialized variety	Farmer owned field
191906	2019	Hubei	Wheat	*F. asiaticum*	15ADON	31.80608704	111.937191	156.221863	Commercialized variety	Farmer owned field
191904	2019	Hubei	Wheat	*F. asiaticum*	15ADON	31.80608704	111.937191	156.221863	Commercialized variety	Farmer owned field
191903	2019	Hubei	Wheat	*F. asiaticum*	3ADON	31.80608704	111.937191	156.221863	Commercialized variety	Farmer owned field
181181	2018	Hubei	Wheat	*F. asiaticum*	3ADON	31.87097403	113.315341	118.555344	Commercialized variety	Farmer owned field
181180	2018	Hubei	Wheat	*F. asiaticum*	3ADON	31.87097403	113.315341	118.555344	Commercialized variety	Farmer owned field
181179	2018	Hubei	Wheat	*F. asiaticum*	3ADON	31.87097403	113.315341	118.555344	Commercialized variety	Farmer owned field
181178	2018	Hubei	Wheat	*F. asiaticum*	3ADON	31.87097403	113.315341	118.555344	Commercialized variety	Farmer owned field
181177	2018	Hubei	Wheat	*F. asiaticum*	3ADON	31.87097403	113.315341	118.555344	Commercialized variety	Farmer owned field
181176	2018	Hubei	Wheat	*F. asiaticum*	3ADON	31.87097403	113.315341	118.555344	Commercialized variety	Farmer owned field
181175	2018	Hubei	Wheat	*F. asiaticum*	3ADON	31.87097403	113.315341	118.555344	Commercialized variety	Farmer owned field
181471	2018	Hubei	Rice	*F. asiaticum*	3ADON	31.87097403	113.315341	118.555344		Farmer owned field
181470	2018	Hubei	Rice	*F. asiaticum*	NIV	31.87097403	113.315341	118.555344		Farmer owned field
181469	2018	Hubei	Rice	*F. asiaticum*	NIV	31.87097403	113.315341	118.555344		Farmer owned field
181468	2018	Hubei	Rice	*F. asiaticum*	NIV	31.87097403	113.315341	118.555344		Farmer owned field
181467	2018	Hubei	Rice	*F. asiaticum*	NIV	31.87097403	113.315341	118.555344		Farmer owned field
181466	2018	Hubei	Rice	*F. asiaticum*	3ADON	31.87097403	113.315341	118.555344		Farmer owned field
181465	2018	Hubei	Rice	*F. asiaticum*	3ADON	31.87097403	113.315341	118.555344		Farmer owned field
181464	2018	Hubei	Rice	*F. asiaticum*	NIV	31.87097403	113.315341	118.555344		Farmer owned field
181463	2018	Hubei	Rice	*F. asiaticum*	3ADON	31.87097403	113.315341	118.555344		Farmer owned field
181462	2018	Hubei	Rice	*F. asiaticum*	3ADON	31.87097403	113.315341	118.555344		Farmer owned field
181461	2018	Hubei	Rice	*F. asiaticum*	3ADON	31.87097403	113.315341	118.555344		Farmer owned field
181460	2018	Hubei	Rice	*F. asiaticum*	3ADON	31.87097403	113.315341	118.555344		Farmer owned field
181459	2018	Hubei	Rice	*F. asiaticum*	3ADON	31.87097403	113.315341	118.555344		Farmer owned field
181458	2018	Hubei	Rice	*F. asiaticum*	3ADON	31.87097403	113.315341	118.555344		Farmer owned field
181457	2018	Hubei	Rice	*F. asiaticum*	NIV	31.87097403	113.315341	118.555344		Farmer owned field
181456	2018	Hubei	Rice	*F. asiaticum*	NIV	31.87097403	113.315341	118.555344		Farmer owned field
181455	2018	Hubei	Rice	*F. asiaticum*	NIV	31.87097403	113.315341	118.555344		Farmer owned field
181454	2018	Hubei	Rice	*F. asiaticum*	NIV	31.87097403	113.315341	118.555344		Farmer owned field
181453	2018	Hubei	Rice	*F. asiaticum*	NIV	31.87097403	113.315341	118.555344		Farmer owned field

181452	2018	Hubei	Rice	*F. asiaticum*	NIV	31.87097403	113.315341	118.555344		Farmer owned field
181451	2018	Hubei	Rice	*F. asiaticum*	NIV	31.87097403	113.315341	118.555344		Farmer owned field
181450	2018	Hubei	Rice	*F. asiaticum*	3ADON	31.87097403	113.315341	118.555344		Farmer owned field
181449	2018	Hubei	Rice	*F. asiaticum*	3ADON	31.87097403	113.315341	118.555344		Farmer owned field
181448	2018	Hubei	Rice	*F. asiaticum*	NIV	31.87097403	113.315341	118.555344		Farmer owned field
181164	2018	Hubei	Wheat	*F. asiaticum*	15ADON	31.88455699	112.030749	87.801369	Commercialized variety	Farmer owned field
181163	2018	Hubei	Wheat	*F. asiaticum*	3ADON	31.88455699	112.030749	87.801369	Commercialized variety	Farmer owned field
181162	2018	Hubei	Wheat	*F. asiaticum*	15ADON	31.88455699	112.030749	87.801369	Commercialized variety	Farmer owned field
181161	2018	Hubei	Wheat	*F. asiaticum*	15ADON	31.88455699	112.030749	87.801369	Commercialized variety	Farmer owned field
181160	2018	Hubei	Wheat	*F. asiaticum*	3ADON	31.88455699	112.030749	87.801369	Commercialized variety	Farmer owned field
181159	2018	Hubei	Wheat	*F. asiaticum*	3ADON	31.88455699	112.030749	87.801369	Commercialized variety	Farmer owned field
181158	2018	Hubei	Wheat	*F. asiaticum*	15ADON	31.88455699	112.030749	87.801369	Commercialized variety	Farmer owned field
181155	2018	Hubei	Wheat	*F. asiaticum*	NIV	31.88455699	112.030749	87.801369	Commercialized variety	Farmer owned field
181154	2018	Hubei	Wheat	*F. asiaticum*	15ADON	31.88455699	112.030749	87.801369	Commercialized variety	Farmer owned field
181153	2018	Hubei	Wheat	*F. asiaticum*	3ADON	31.88455699	112.030749	87.801369	Commercialized variety	Farmer owned field
181152	2018	Hubei	Wheat	*F. asiaticum*	3ADON	31.88455699	112.030749	87.801369	Commercialized variety	Farmer owned field
181151	2018	Hubei	Wheat	*F. asiaticum*	15ADON	31.88455699	112.030749	87.801369	Commercialized variety	Farmer owned field
181150	2018	Hubei	Wheat	*F. asiaticum*	15ADON	31.88455699	112.030749	87.801369	Commercialized variety	Farmer owned field
181149	2018	Hubei	Wheat	*F. asiaticum*	3ADON	31.88455699	112.030749	87.801369	Commercialized variety	Farmer owned field
181148	2018	Hubei	Wheat	*F. asiaticum*	15ADON	31.88455699	112.030749	87.801369	Commercialized variety	Farmer owned field
181146	2018	Hubei	Wheat	*F. asiaticum*	15ADON	31.88455699	112.030749	87.801369	Commercialized variety	Farmer owned field
181145	2018	Hubei	Wheat	*F. asiaticum*	3ADON	31.88455699	112.030749	87.801369	Commercialized variety	Farmer owned field
183715	2018	Hubei	Wheat	*F. asiaticum*	3ADON	31.963216	112.49149	NA	Commercialized variety	Farmer owned field
183713	2018	Hubei	Wheat	*F. asiaticum*	3ADON	31.963216	112.49149	NA	Commercialized variety	Farmer owned field
191305	2019	Hubei	Wheat	*F. asiaticum*	3ADON	32.076591	112.603559	138.409988	Commercialized variety	Farmer owned field
191304	2019	Hubei	Wheat	*F. asiaticum*	3ADON	32.076591	112.603559	138.409988	Commercialized variety	Farmer owned field
191303	2019	Hubei	Wheat	*F. asiaticum*	3ADON	32.076591	112.603559	138.409988	Commercialized variety	Farmer owned field
191301	2019	Hubei	Wheat	*F. asiaticum*	3ADON	32.076591	112.603559	138.409988	Commercialized variety	Farmer owned field
191300	2019	Hubei	Wheat	*F. asiaticum*	3ADON	32.076591	112.603559	138.409988	Commercialized variety	Farmer owned field
191299	2019	Hubei	Wheat	*F. asiaticum*	3ADON	32.076591	112.603559	138.409988	Commercialized variety	Farmer owned field
191297	2019	Hubei	Wheat	*F. asiaticum*	3ADON	32.076591	112.603559	138.409988	Commercialized variety	Farmer owned field
191296	2019	Hubei	Wheat	*F. asiaticum*	3ADON	32.076591	112.603559	138.409988	Commercialized variety	Farmer owned field
191295	2019	Hubei	Wheat	*F. asiaticum*	3ADON	32.076591	112.603559	138.409988	Commercialized variety	Farmer owned field
191294	2019	Hubei	Wheat	*F. asiaticum*	3ADON	32.076591	112.603559	138.409988	Commercialized variety	Farmer owned field
191293	2019	Hubei	Wheat	*F. asiaticum*	3ADON	32.076591	112.603559	138.409988	Commercialized variety	Farmer owned field

191292	2019	Hubei	Wheat	*F. asiaticum*	3ADON	32.076591	112.603559	138.409988	Commercialized variety	Farmer owned field
183737	2018	Hubei	Wheat	*F. asiaticum*	3ADON	32.121861	112.364484	NA	Commercialized variety	Farmer owned field
183736	2018	Hubei	Wheat	*F. asiaticum*	3ADON	32.121861	112.364484	NA	Commercialized variety	Farmer owned field
183735	2018	Hubei	Wheat	*F. asiaticum*	NIV	32.121861	112.364484	NA	Commercialized variety	Farmer owned field
191995	2019	Hubei	Wheat	*F. asiaticum*	3ADON	32.13446603	112.697802	169.103165	Commercialized variety	Farmer owned field
191994	2019	Hubei	Wheat	*F. asiaticum*	3ADON	32.13446603	112.697802	169.103165	Commercialized variety	Farmer owned field
191993	2019	Hubei	Wheat	*F. asiaticum*	3ADON	32.13446603	112.697802	169.103165	Commercialized variety	Farmer owned field
191992	2019	Hubei	Wheat	*F. asiaticum*	3ADON	32.13446603	112.697802	169.103165	Commercialized variety	Farmer owned field
191991	2019	Hubei	Wheat	*F. asiaticum*	3ADON	32.13446603	112.697802	169.103165	Commercialized variety	Farmer owned field
190040	2019	Hubei	Wheat	*F. asiaticum*	15ADON	32.28376399	112.255411	115.217537	Commercialized variety	Farmer owned field
190038	2019	Hubei	Wheat	*F. asiaticum*	3ADON	32.28376399	112.255411	115.217537	Commercialized variety	Farmer owned field
190035	2019	Hubei	Wheat	*F. asiaticum*	3ADON	32.28376399	112.255411	115.217537	Commercialized variety	Farmer owned field
190034	2019	Hubei	Wheat	*F. asiaticum*	3ADON	32.28376399	112.255411	115.217537	Commercialized variety	Farmer owned field
190032	2019	Hubei	Wheat	*F. asiaticum*	15ADON	32.28376399	112.255411	115.217537	Commercialized variety	Farmer owned field
190030	2019	Hubei	Wheat	*F. asiaticum*	3ADON	32.28376399	112.255411	115.217537	Commercialized variety	Farmer owned field
183481	2018	Hubei	Wheat	*F. asiaticum*	3ADON	32.287139	112.256175	NA	Commercialized variety	Farmer owned field
183480	2018	Hubei	Wheat	*F. asiaticum*	15ADON	32.287139	112.256175	NA	Commercialized variety	Farmer owned field
183479	2018	Hubei	Wheat	*F. asiaticum*	NIV	32.287139	112.256175	NA	Commercialized variety	Farmer owned field
183478	2018	Hubei	Wheat	*F. asiaticum*	3ADON	32.287139	112.256175	NA	Commercialized variety	Farmer owned field
183477	2018	Hubei	Wheat	*F. asiaticum*	3ADON	32.287139	112.256175	NA	Commercialized variety	Farmer owned field
183476	2018	Hubei	Wheat	*F. asiaticum*	NIV	32.287139	112.256175	NA	Commercialized variety	Farmer owned field
HBXG193	2014	Hubei	Wheat	*F. asiaticum*	3ADON	32.77738333	116.6282333	26	Commercialized variety	Farmer owned field
181016	2018	Hubei	Wheat	*F. asiaticum*	3ADON	NA	NA	NA	Commercialized variety	Farmer owned field
181015	2018	Hubei	Wheat	*F. asiaticum*	3ADON	NA	NA	NA	Commercialized variety	Farmer owned field
180187	2018	Guangxi	Rice	*F. asiaticum*	15ADON	25.21622	110.048376	NA		Farmer owned field
180180	2018	Guangxi	Rice	*F. asiaticum*	NIV	24.06901	109.415228	NA		Farmer owned field
180178	2018	Guangxi	Rice	*F. asiaticum*	15ADON	24.06901	109.415228	NA		Farmer owned field
180177	2018	Guangxi	Rice	*F. asiaticum*	15ADON	24.06901	109.415228	NA		Farmer owned field
180176	2018	Guangxi	Rice	*F. asiaticum*	15ADON	24.06901	109.415228	NA		Farmer owned field
180175	2018	Guangxi	Rice	*F. asiaticum*	15ADON	24.06901	109.415228	NA		Farmer owned field
180174	2018	Guangxi	Rice	*F. asiaticum*	15ADON	24.06901	109.415228	NA		Farmer owned field
180091	2018	Guangxi	Rice	*F. asiaticum*	NIV	25.129269	110.030149	NA		Farmer owned field
180090	2018	Guangxi	Rice	*F. asiaticum*	NIV	25.129269	110.030149	NA		Farmer owned field
180089	2018	Guangxi	Rice	*F. asiaticum*	NIV	25.129269	110.030149	NA		Farmer owned field
180088	2018	Guangxi	Rice	*F. asiaticum*	NIV	25.129269	110.030149	NA		Farmer owned field

180087	2018	Guangxi	Rice	*F. asiaticum*	NIV	25.129269	110.030149	NA	Farmer owned field
180086	2018	Guangxi	Rice	*F. asiaticum*	NIV	25.129269	110.030149	NA	Farmer owned field
180085	2018	Guangxi	Rice	*F. asiaticum*	NIV	25.129269	110.030149	NA	Farmer owned field
180084	2018	Guangxi	Rice	*F. asiaticum*	NIV	24.963552	110.005226	NA	Farmer owned field
180083	2018	Guangxi	Rice	*F. asiaticum*	NIV	24.963552	110.005226	NA	Farmer owned field
180082	2018	Guangxi	Rice	*F. asiaticum*	NIV	24.963552	110.005226	NA	Farmer owned field
180081	2018	Guangxi	Rice	*F. asiaticum*	NIV	24.963552	110.005226	NA	Farmer owned field
180079	2018	Guangxi	Rice	*F. asiaticum*	NIV	24.963552	110.005226	NA	Farmer owned field
180078	2018	Guangxi	Rice	*F. asiaticum*	NIV	25.129269	110.030149	NA	Farmer owned field
180077	2018	Guangxi	Rice	*F. asiaticum*	NIV	25.129269	110.030149	NA	Farmer owned field
180076	2018	Guangxi	Rice	*F. asiaticum*	NIV	25.129269	110.030149	NA	Farmer owned field
180075	2018	Guangxi	Rice	*F. asiaticum*	NIV	25.129269	110.030149	NA	Farmer owned field
180074	2018	Guangxi	Rice	*F. asiaticum*	NIV	25.129269	110.030149	NA	Farmer owned field
180073	2018	Guangxi	Rice	*F. asiaticum*	NIV	25.129269	110.030149	NA	Farmer owned field
180072	2018	Guangxi	Rice	*F. asiaticum*	NIV	25.129269	110.030149	NA	Farmer owned field
180071	2018	Guangxi	Rice	*F. asiaticum*	NIV	25.129269	110.030149	NA	Farmer owned field
180070	2018	Guangxi	Rice	*F. asiaticum*	NIV	25.129269	110.030149	NA	Farmer owned field
180069	2018	Guangxi	Rice	*F. asiaticum*	NIV	25.129269	110.030149	NA	Farmer owned field
180068	2018	Guangxi	Rice	*F. asiaticum*	NIV	25.129269	110.030149	NA	Farmer owned field
180067	2018	Guangxi	Rice	*F. asiaticum*	NIV	25.129269	110.030149	NA	Farmer owned field
180066	2018	Guangxi	Rice	*F. asiaticum*	NIV	25.129269	110.030149	NA	Farmer owned field
180065	2018	Guangxi	Rice	*F. asiaticum*	NIV	25.129269	110.030149	NA	Farmer owned field
180064	2018	Guangxi	Rice	*F. asiaticum*	NIV	25.129269	110.030149	NA	Farmer owned field
180063	2018	Guangxi	Rice	*F. asiaticum*	NIV	25.129269	110.030149	NA	Farmer owned field
180062	2018	Guangxi	Rice	*F. asiaticum*	NIV	25.129269	110.030149	NA	Farmer owned field
180061	2018	Guangxi	Rice	*F. asiaticum*	NIV	25.209222	110.128887	NA	Farmer owned field
180058	2018	Guangxi	Rice	*F. asiaticum*	NIV	25.209222	110.128887	NA	Farmer owned field
180056	2018	Guangxi	Rice	*F. asiaticum*	NIV	25.209222	110.128887	NA	Farmer owned field
180055	2018	Guangxi	Rice	*F. asiaticum*	NIV	25.209222	110.128887	NA	Farmer owned field
180054	2018	Guangxi	Rice	*F. asiaticum*	NIV	25.209222	110.128887	NA	Farmer owned field
180053	2018	Guangxi	Rice	*F. asiaticum*	NIV	25.209222	110.128887	NA	Farmer owned field
180052	2018	Guangxi	Rice	*F. asiaticum*	NIV	25.209222	110.128887	NA	Farmer owned field
180051	2018	Guangxi	Rice	*F. asiaticum*	NIV	25.209222	110.128887	NA	Farmer owned field
180050	2018	Guangxi	Rice	*F. asiaticum*	NIV	25.209222	110.128887	NA	Farmer owned field
180049	2018	Guangxi	Rice	*F. asiaticum*	NIV	25.209222	110.128887	NA	Farmer owned field

180048	2018	Guangxi	Rice	*F. asiaticum*	NIV	25.209222	110.128887	NA	Farmer owned field
180047	2018	Guangxi	Rice	*F. asiaticum*	NIV	25.209222	110.128887	NA	Farmer owned field
180046	2018	Guangxi	Rice	*F. asiaticum*	NIV	25.209222	110.128887	NA	Farmer owned field
180045	2018	Guangxi	Rice	*F. asiaticum*	NIV	25.209222	110.128887	NA	Farmer owned field
180044	2018	Guangxi	Rice	*F. asiaticum*	NIV	25.209222	110.128887	NA	Farmer owned field
180043	2018	Guangxi	Rice	*F. asiaticum*	NIV	25.209222	110.128887	NA	Farmer owned field
180042	2018	Guangxi	Rice	*F. asiaticum*	NIV	25.209222	110.128887	NA	Farmer owned field
180041	2018	Guangxi	Rice	*F. asiaticum*	NIV	25.193347	110.052213	NA	Farmer owned field
180040	2018	Guangxi	Rice	*F. asiaticum*	NIV	25.193347	110.052213	NA	Farmer owned field
180039	2018	Guangxi	Rice	*F. asiaticum*	NIV	25.193347	110.052213	NA	Farmer owned field
180038	2018	Guangxi	Rice	*F. asiaticum*	NIV	25.193347	110.052213	NA	Farmer owned field
180037	2018	Guangxi	Rice	*F. asiaticum*	NIV	25.193347	110.052213	NA	Farmer owned field
180036	2018	Guangxi	Rice	*F. asiaticum*	NIV	25.193347	110.052213	NA	Farmer owned field
180034	2018	Guangxi	Rice	*F. asiaticum*	NIV	25.193347	110.052213	NA	Farmer owned field
180033	2018	Guangxi	Rice	*F. asiaticum*	NIV	25.193347	110.052213	NA	Farmer owned field
180032	2018	Guangxi	Rice	*F. asiaticum*	NIV	25.193347	110.052213	NA	Farmer owned field
180031	2018	Guangxi	Rice	*F. asiaticum*	NIV	25.193347	110.052213	NA	Farmer owned field
180030	2018	Guangxi	Rice	*F. asiaticum*	NIV	25.193347	110.052213	NA	Farmer owned field
180029	2018	Guangxi	Rice	*F. asiaticum*	NIV	25.193347	110.052213	NA	Farmer owned field
180027	2018	Guangxi	Rice	*F. asiaticum*	NIV	25.193347	110.052213	NA	Farmer owned field
180026	2018	Guangxi	Rice	*F. asiaticum*	NIV	25.193347	110.052213	NA	Farmer owned field
180025	2018	Guangxi	Rice	*F. asiaticum*	NIV	25.193347	110.052213	NA	Farmer owned field
180024	2018	Guangxi	Rice	*F. asiaticum*	NIV	25.193347	110.052213	NA	Farmer owned field
180023	2018	Guangxi	Rice	*F. asiaticum*	NIV	25.193347	110.052213	NA	Farmer owned field
180022	2018	Guangxi	Rice	*F. asiaticum*	NIV	25.193347	110.052213	NA	Farmer owned field
180021	2018	Guangxi	Rice	*F. asiaticum*	NIV	25.193347	110.052213	NA	Farmer owned field
180020	2018	Guangxi	Rice	*F. asiaticum*	NIV	25.193347	110.052213	NA	Farmer owned field
180019	2018	Guangxi	Rice	*F. asiaticum*	NIV	25.193347	110.052213	NA	Farmer owned field
180018	2018	Guangxi	Rice	*F. asiaticum*	NIV	25.193347	110.052213	NA	Farmer owned field
180017	2018	Guangxi	Rice	*F. asiaticum*	NIV	25.193347	110.052213	NA	Farmer owned field
180016	2018	Guangxi	Rice	*F. asiaticum*	NIV	25.193347	110.052213	NA	Farmer owned field
180015	2018	Guangxi	Rice	*F. asiaticum*	NIV	25.193347	110.052213	NA	Farmer owned field
180014	2018	Guangxi	Rice	*F. asiaticum*	NIV	25.193347	110.052213	NA	Farmer owned field
180013	2018	Guangxi	Rice	*F. asiaticum*	NIV	25.193347	110.052213	NA	Farmer owned field
180012	2018	Guangxi	Rice	*F. asiaticum*	NIV	25.193347	110.052213	NA	Farmer owned field

180011	2018	Guangxi	Rice	*F. asiaticum*	NIV	25.193347	110.052213	NA	Farmer owned field
180010	2018	Guangxi	Rice	*F. asiaticum*	NIV	25.193347	110.052213	NA	Farmer owned field
180009	2018	Guangxi	Rice	*F. asiaticum*	NIV	25.193347	110.052213	NA	Farmer owned field
180008	2018	Guangxi	Rice	*F. asiaticum*	NIV	25.193347	110.052213	NA	Farmer owned field
180007	2018	Guangxi	Rice	*F. asiaticum*	NIV	25.193347	110.052213	NA	Farmer owned field
180006	2018	Guangxi	Rice	*F. asiaticum*	NIV	25.193347	110.052213	NA	Farmer owned field
180005	2018	Guangxi	Rice	*F. asiaticum*	NIV	25.193347	110.052213	NA	Farmer owned field
180004	2018	Guangxi	Rice	*F. asiaticum*	NIV	25.193347	110.052213	NA	Farmer owned field
180003	2018	Guangxi	Rice	*F. asiaticum*	NIV	25.193347	110.052213	NA	Farmer owned field
180002	2018	Guangxi	Rice	*F. asiaticum*	NIV	25.193347	110.052213	NA	Farmer owned field
180001	2018	Guangxi	Rice	*F. asiaticum*	NIV	25.193347	110.052213	NA	Farmer owned field
170944	2017	Hunan	Rice	*F. asiaticum*	3ADON	28.19698798	112.766222	72.16	Farmer owned field
170985	2017	Hunan	Rice	*F. asiaticum*	NIV	28.19918202	112.683053	93.43	Farmer owned field
170984	2017	Hunan	Rice	*F. asiaticum*	NIV	28.19918202	112.683053	93.43	Farmer owned field
170983	2017	Hunan	Rice	*F. asiaticum*	NIV	28.19918202	112.683053	93.43	Farmer owned field
170982	2017	Hunan	Rice	*F. asiaticum*	NIV	28.19918202	112.683053	93.43	Farmer owned field
170981	2017	Hunan	Rice	*F. asiaticum*	NIV	28.19918202	112.683053	93.43	Farmer owned field
170980	2017	Hunan	Rice	*F. asiaticum*	NIV	28.19918202	112.683053	93.43	Farmer owned field
170979	2017	Hunan	Rice	*F. asiaticum*	NIV	28.19918202	112.683053	93.43	Farmer owned field
170978	2017	Hunan	Rice	*F. asiaticum*	3ADON	28.19918202	112.683053	93.43	Farmer owned field
170977	2017	Hunan	Rice	*F. asiaticum*	NIV	28.19918202	112.683053	93.43	Farmer owned field
170975	2017	Hunan	Rice	*F. asiaticum*	NIV	28.19918202	112.683053	93.43	Farmer owned field
170974	2017	Hunan	Rice	*F. asiaticum*	3ADON	28.19918202	112.683053	93.43	Farmer owned field
170973	2017	Hunan	Rice	*F. asiaticum*	NIV	28.19918202	112.683053	93.43	Farmer owned field
170972	2017	Hunan	Rice	*F. asiaticum*	3ADON	28.19918202	112.683053	93.43	Farmer owned field
170971	2017	Hunan	Rice	*F. asiaticum*	3ADON	28.19918202	112.683053	93.43	Farmer owned field
170970	2017	Hunan	Rice	*F. asiaticum*	NIV	28.19918202	112.683053	93.43	Farmer owned field
170969	2017	Hunan	Rice	*F. asiaticum*	NIV	28.19918202	112.683053	93.43	Farmer owned field
170968	2017	Hunan	Rice	*F. asiaticum*	3ADON	28.19918202	112.683053	93.43	Farmer owned field
170967	2017	Hunan	Rice	*F. asiaticum*	15ADON	28.19918202	112.683053	93.43	Farmer owned field
170966	2017	Hunan	Rice	*F. asiaticum*	3ADON	28.19918202	112.683053	93.43	Farmer owned field
170965	2017	Hunan	Rice	*F. asiaticum*	NIV	28.19918202	112.683053	93.43	Farmer owned field
170964	2017	Hunan	Rice	*F. asiaticum*	NIV	28.19918202	112.683053	93.43	Farmer owned field
170963	2017	Hunan	Rice	*F. asiaticum*	3ADON	28.19918202	112.683053	93.43	Farmer owned field
181720	2018	Sichuan	Rice	*F. asiaticum*	NIV	31.89135304	104.792138	603.32428	Farmer owned field

181718	2018	Sichuan	Rice	*F. asiaticum*	NIV	31.89135304	104.792138	603.32428		Farmer owned field
181717	2018	Sichuan	Rice	*F. asiaticum*	NIV	31.89135304	104.792138	603.32428		Farmer owned field
181715	2018	Sichuan	Rice	*F. asiaticum*	NIV	31.89135304	104.792138	603.32428		Farmer owned field
181714	2018	Sichuan	Rice	*F. asiaticum*	NIV	31.89135304	104.792138	603.32428		Farmer owned field
181713	2018	Sichuan	Rice	*F. asiaticum*	NIV	31.89135304	104.792138	603.32428		Farmer owned field
181712	2018	Sichuan	Rice	*F. asiaticum*	NIV	31.89135304	104.792138	603.32428		Farmer owned field
181711	2018	Sichuan	Rice	*F. asiaticum*	NIV	31.89135304	104.792138	603.32428		Farmer owned field
181710	2018	Sichuan	Rice	*F. asiaticum*	NIV	31.89135304	104.792138	603.32428		Farmer owned field
181709	2018	Sichuan	Rice	*F. asiaticum*	NIV	31.89135304	104.792138	603.32428		Farmer owned field
181708	2018	Sichuan	Rice	*F. asiaticum*	NIV	31.89135304	104.792138	603.32428		Farmer owned field
JY76-1	2014	Sichuan	Wheat	*F. asiaticum*	NIV	31.98287	105.0974	622	Commercialized variety	Farmer owned field
JY75-2	2014	Sichuan	Wheat	*F. asiaticum*	NIV	31.98287	105.0974	622	Commercialized variety	Farmer owned field
JY5-2	2014	Sichuan	Wheat	*F. asiaticum*	NIV	31.98287	105.0974	622	Commercialized variety	Farmer owned field
JY39	2014	Sichuan	Wheat	*F. asiaticum*	NIV	31.98287	105.0974	622	Commercialized variety	Farmer owned field
JY26	2014	Sichuan	Wheat	*F. asiaticum*	NIV	31.98287	105.0974	622	Commercialized variety	Farmer owned field
JY20-2	2014	Sichuan	Wheat	*F. asiaticum*	NIV	31.98287	105.0974	622	Commercialized variety	Farmer owned field
JY123-1	2014	Sichuan	Wheat	*F. asiaticum*	NIV	31.98287	105.0974	622	Commercialized variety	Farmer owned field
JY120	2014	Sichuan	Wheat	*F. asiaticum*	NIV	31.98287	105.0974	622	Commercialized variety	Farmer owned field
JY100	2014	Sichuan	Wheat	*F. asiaticum*	NIV	31.98287	105.0974	622	Commercialized variety	Farmer owned field
180250	2018	Sichuan	Wheat	*F. asiaticum*	NIV	NA	NA	NA	Commercialized variety	Farmer owned field
180249	2018	Sichuan	Wheat	*F. asiaticum*	NIV	NA	NA	NA	Commercialized variety	Farmer owned field
180246	2018	Sichuan	Wheat	*F. asiaticum*	NIV	NA	NA	NA	Commercialized variety	Farmer owned field
180243	2018	Sichuan	Wheat	*F. asiaticum*	NIV	NA	NA	NA	Commercialized variety	Farmer owned field
180242	2018	Sichuan	Wheat	*F. asiaticum*	NIV	NA	NA	NA	Commercialized variety	Farmer owned field
180222	2018	Sichuan	Wheat	*F. asiaticum*	NIV	NA	NA	NA	Commercialized variety	Farmer owned field
180221	2018	Sichuan	Wheat	*F. asiaticum*	NIV	NA	NA	NA	Commercialized variety	Farmer owned field
180220	2018	Sichuan	Wheat	*F. asiaticum*	NIV	NA	NA	NA	Commercialized variety	Farmer owned field
180264	2018	sichuan	Wheat	*F. asiaticum*	NIV	NA	NA	NA	Commercialized variety	Farmer owned field
180265	2018	sichuan	Wheat	*F. asiaticum*	NIV	NA	NA	NA	Commercialized variety	Farmer owned field
171629	2017	Chongqing	Rice	*F. asiaticum*	NIV	29.625162	107.337275	537.2		Farmer owned field
171624	2017	Chongqing	Rice	*F. asiaticum*	NIV	29.625162	107.337275	537.2		Farmer owned field
171623	2017	Chongqing	Rice	*F. asiaticum*	NIV	29.625162	107.337275	537.2		Farmer owned field
171622	2017	Chongqing	Rice	*F. asiaticum*	NIV	29.625162	107.337275	537.2		Farmer owned field
171621	2017	Chongqing	Rice	*F. asiaticum*	NIV	29.625162	107.337275	537.2		Farmer owned field
171620	2017	Chongqing	Rice	*F. asiaticum*	NIV	29.625162	107.337275	537.2		Farmer owned field

171619	2017	Chongqing	Rice	*F. asiaticum*	NIV	29.625162	107.337275	537.2		Farmer owned field
171618	2017	Chongqing	Rice	*F. asiaticum*	NIV	29.625162	107.337275	537.2		Farmer owned field
171617	2017	Chongqing	Rice	*F. asiaticum*	NIV	29.625162	107.337275	537.2		Farmer owned field
171616	2017	Chongqing	Rice	*F. asiaticum*	NIV	29.625162	107.337275	537.2		Farmer owned field
171615	2017	Chongqing	Rice	*F. asiaticum*	NIV	29.625162	107.337275	537.2		Farmer owned field
180213	2018	Jiangsu	Wheat	*F. asiaticum*	3ADON	NA	NA	NA	Commercialized variety	Commercial farm
180214	2018	Jiangsu	Wheat	*F. asiaticum*	3ADON	NA	NA	NA	Commercialized variety	Commercial farm
180215	2018	Jiangsu	Wheat	*F. asiaticum*	3ADON	NA	NA	NA	Commercialized variety	Commercial farm
180266	2018	sichuan	Wheat	*F. asiaticum*	NIV	NA	NA	NA	Commercialized variety	Farmer owned field
180267	2018	sichuan	Wheat	*F. asiaticum*	NIV	NA	NA	NA	Commercialized variety	Farmer owned field
180268	2018	sichuan	Wheat	*F. asiaticum*	NIV	NA	NA	NA	Commercialized variety	Farmer owned field
180269	2018	sichuan	Wheat	*F. asiaticum*	NIV	NA	NA	NA	Commercialized variety	Farmer owned field
180270	2018	sichuan	Wheat	*F. asiaticum*	NIV	NA	NA	NA	Commercialized variety	Farmer owned field
180271	2018	sichuan	Wheat	*F. asiaticum*	NIV	NA	NA	NA	Commercialized variety	Farmer owned field
180272	2018	sichuan	Wheat	*F. asiaticum*	NIV	NA	NA	NA	Commercialized variety	Farmer owned field
180273	2018	sichuan	Wheat	*F. asiaticum*	NIV	NA	NA	NA	Commercialized variety	Farmer owned field
180274	2018	sichuan	Wheat	*F. asiaticum*	NIV	NA	NA	NA	Commercialized variety	Farmer owned field
180275	2018	sichuan	Wheat	*F. asiaticum*	NIV	NA	NA	NA	Commercialized variety	Farmer owned field
180276	2018	sichuan	Wheat	*F. asiaticum*	NIV	NA	NA	NA	Commercialized variety	Farmer owned field
180277	2018	sichuan	Wheat	*F. asiaticum*	NIV	NA	NA	NA	Commercialized variety	Farmer owned field
180278	2018	sichuan	Wheat	*F. asiaticum*	NIV	NA	NA	NA	Commercialized variety	Farmer owned field
180279	2018	sichuan	Wheat	*F. asiaticum*	NIV	NA	NA	NA	Commercialized variety	Farmer owned field
171342	2017	Fujian	Rice	*F. asiaticum*	NIV	26.947053	117.227145	288		Farmer owned field
171341	2017	Fujian	Rice	*F. asiaticum*	NIV	26.947053	117.227145	288		Farmer owned field
171340	2017	Fujian	Rice	*F. asiaticum*	NIV	26.947053	117.227145	288		Farmer owned field
171339	2017	Fujian	Rice	*F. asiaticum*	NIV	26.947053	117.227145	288		Farmer owned field
171338	2017	Fujian	Rice	*F. asiaticum*	NIV	26.947053	117.227145	288		Farmer owned field
171337	2017	Fujian	Rice	*F. asiaticum*	NIV	26.947053	117.227145	288		Farmer owned field
171336	2017	Fujian	Rice	*F. asiaticum*	NIV	26.947053	117.227145	288		Farmer owned field
171335	2017	Fujian	Rice	*F. asiaticum*	NIV	26.947053	117.227145	288		Farmer owned field
171334	2017	Fujian	Rice	*F. asiaticum*	NIV	26.947053	117.227145	288		Farmer owned field
171333	2017	Fujian	Rice	*F. asiaticum*	NIV	26.947053	117.227145	288		Farmer owned field
171332	2017	Fujian	Rice	*F. asiaticum*	15ADON	26.947053	117.227145	288		Farmer owned field
171331	2017	Fujian	Rice	*F. asiaticum*	NIV	26.947053	117.227145	288		Farmer owned field
171330	2017	Fujian	Rice	*F. asiaticum*	NIV	26.947053	117.227145	288		Farmer owned field

171329	2017	Fujian	Rice	*F. asiaticum*	15ADON	26.947053	117.227145	288	Farmer owned field
171328	2017	Fujian	Rice	*F. asiaticum*	NIV	26.947053	117.227145	288	Farmer owned field
171327	2017	Fujian	Rice	*F. asiaticum*	NIV	26.947053	117.227145	288	Farmer owned field
171326	2017	Fujian	Rice	*F. asiaticum*	NIV	26.947053	117.227145	288	Farmer owned field
171325	2017	Fujian	Rice	*F. asiaticum*	15ADON	26.947053	117.227145	288	Farmer owned field
171324	2017	Fujian	Rice	*F. asiaticum*	NIV	26.947053	117.227145	288	Farmer owned field
171323	2017	Fujian	Rice	*F. asiaticum*	NIV	26.947053	117.227145	288	Farmer owned field
171322	2017	Fujian	Rice	*F. asiaticum*	15ADON	26.947053	117.227145	288	Farmer owned field
171321	2017	Fujian	Rice	*F. asiaticum*	NIV	26.947053	117.227145	288	Farmer owned field
171320	2017	Fujian	Rice	*F. asiaticum*	NIV	26.947053	117.227145	288	Farmer owned field
171319	2017	Fujian	Rice	*F. asiaticum*	15ADON	26.947053	117.227145	288	Farmer owned field
NA	2015	Fujian	Rice	*F. asiaticum*	NIV	27.13868	118.13741	NA	Farmer owned field
NA	2015	Fujian	Rice	*F. asiaticum*	NIV	27.13868	118.13741	NA	Farmer owned field
NA	2015	Fujian	Rice	*F. asiaticum*	15ADON	27.13868	118.13741	NA	Farmer owned field
NA	2015	Fujian	Rice	*F. asiaticum*	NIV	27.13868	118.13741	NA	Farmer owned field
NA	2015	Fujian	Rice	*F. asiaticum*	NIV	27.13868	118.13741	NA	Farmer owned field
NA	2015	Fujian	Rice	*F. asiaticum*	15ADON	27.13868	118.13741	NA	Farmer owned field
NA	2015	Fujian	Rice	*F. asiaticum*	NIV	27.13868	118.13741	NA	Farmer owned field
NA	2015	Fujian	Rice	*F. asiaticum*	NIV	27.13868	118.13741	NA	Farmer owned field
NA	2015	Fujian	Rice	*F. asiaticum*	NIV	27.13868	118.13741	NA	Farmer owned field
NA	2015	Fujian	Rice	*F. asiaticum*	NIV	27.13868	118.13741	NA	Farmer owned field
NA	2015	Fujian	Rice	*F. asiaticum*	NIV	27.13868	118.13741	NA	Farmer owned field
NA	2015	Fujian	Rice	*F. asiaticum*	NIV	27.13868	118.13741	NA	Farmer owned field
NA	2015	Fujian	Rice	*F. asiaticum*	NIV	27.13868	118.13741	NA	Farmer owned field
NA	2015	Fujian	Rice	*F. asiaticum*	NIV	27.13868	118.13741	NA	Farmer owned field
NA	2015	Fujian	Rice	*F. asiaticum*	NIV	27.13868	118.13741	NA	Farmer owned field
NA	2015	Fujian	Rice	*F. asiaticum*	NIV	27.13868	118.13741	NA	Farmer owned field
NA	2015	Fujian	Rice	*F. asiaticum*	15ADON	27.13868	118.13741	NA	Farmer owned field
NA	2015	Fujian	Rice	*F. asiaticum*	NIV	27.13868	118.13741	NA	Farmer owned field
NA	2015	Fujian	Rice	*F. asiaticum*	NIV	27.13868	118.13741	NA	Farmer owned field
NA	2015	Fujian	Rice	*F. asiaticum*	15ADON	27.13868	118.13741	NA	Farmer owned field
NA	2015	Fujian	Rice	*F. asiaticum*	15ADON	27.13868	118.13741	NA	Farmer owned field
NA	2015	Fujian	Rice	*F. asiaticum*	NIV	27.13868	118.13741	NA	Farmer owned field
NA	2015	Fujian	Rice	*F. asiaticum*	NIV	27.13868	118.13741	NA	Farmer owned field
NA	2015	Fujian	Rice	*F. asiaticum*	15ADON	27.13868	118.13741	NA	Farmer owned field

NA	2015	Fujian	Rice	*F. asiaticum*	15ADON	27.13868	118.13741	NA	Farmer owned field
NA	2015	Fujian	Rice	*F. asiaticum*	15ADON	27.13868	118.13741	NA	Farmer owned field
NA	2015	Fujian	Rice	*F. asiaticum*	NIV	27.13868	118.13741	NA	Farmer owned field
NA	2015	Fujian	Rice	*F. asiaticum*	NIV	27.13868	118.13741	NA	Farmer owned field
NA	2015	Fujian	Rice	*F. asiaticum*	NIV	27.13868	118.13741	NA	Farmer owned field
NA	2015	Fujian	Rice	*F. asiaticum*	15ADON	27.13868	118.13741	NA	Farmer owned field
NA	2015	Fujian	Rice	*F. asiaticum*	15ADON	27.13868	118.13741	NA	Farmer owned field
NA	2015	Fujian	Rice	*F. asiaticum*	15ADON	27.13868	118.13741	NA	Farmer owned field
NA	2015	Fujian	Rice	*F. asiaticum*	15ADON	27.13868	118.13741	NA	Farmer owned field
NA	2015	Fujian	Rice	*F. asiaticum*	NIV	27.13868	118.13741	NA	Farmer owned field
NA	2015	Fujian	Rice	*F. asiaticum*	NIV	27.13868	118.13741	NA	Farmer owned field
NA	2015	Fujian	Rice	*F. asiaticum*	15ADON	27.13868	118.13741	NA	Farmer owned field
NA	2015	Fujian	Rice	*F. asiaticum*	15ADON	27.13868	118.13741	NA	Farmer owned field
NA	2015	Fujian	Rice	*F. asiaticum*	15ADON	27.13868	118.13741	NA	Farmer owned field
NA	2015	Fujian	Rice	*F. asiaticum*	15ADON	27.13868	118.13741	NA	Farmer owned field
NA	2015	Fujian	Rice	*F. asiaticum*	15ADON	27.13868	118.13741	NA	Farmer owned field
NA	2015	Fujian	Rice	*F. asiaticum*	15ADON	27.13868	118.13741	NA	Farmer owned field
NA	2015	Fujian	Rice	*F. asiaticum*	15ADON	27.13868	118.13741	NA	Farmer owned field
NA	2015	Fujian	Rice	*F. asiaticum*	NIV	27.13868	118.13741	NA	Farmer owned field
NA	2015	Fujian	Rice	*F. asiaticum*	15ADON	27.13868	118.13741	NA	Farmer owned field
NA	2015	Fujian	Rice	*F. asiaticum*	15ADON	27.13868	118.13741	NA	Farmer owned field
NA	2015	Fujian	Rice	*F. asiaticum*	NIV	27.13868	118.13741	NA	Farmer owned field
NA	2015	Fujian	Rice	*F. asiaticum*	15ADON	27.13868	118.13741	NA	Farmer owned field
NA	2015	Fujian	Rice	*F. asiaticum*	NIV	27.13868	118.13741	NA	Farmer owned field
NA	2015	Fujian	Rice	*F. asiaticum*	15ADON	27.13868	118.13741	NA	Farmer owned field
NA	2015	Fujian	Rice	*F. asiaticum*	NIV	27.13868	118.13741	NA	Farmer owned field
NA	2015	Fujian	Rice	*F. asiaticum*	NIV	27.13868	118.13741	NA	Farmer owned field
NA	2015	Fujian	Rice	*F. asiaticum*	NIV	27.13868	118.13741	NA	Farmer owned field
NA	2015	Fujian	Rice	*F. asiaticum*	NIV	27.13868	118.13741	NA	Farmer owned field
NA	2015	Fujian	Rice	*F. asiaticum*	NIV	27.13868	118.13741	NA	Farmer owned field
NA	2015	Fujian	Rice	*F. asiaticum*	NIV	27.13868	118.13741	NA	Farmer owned field
NA	2015	Fujian	Rice	*F. asiaticum*	NIV	27.13868	118.13741	NA	Farmer owned field
NA	2015	Fujian	Rice	*F. asiaticum*	NIV	27.13868	118.13741	NA	Farmer owned field
NA	2015	Fujian	Rice	*F. asiaticum*	15ADON	27.13868	118.13741	NA	Farmer owned field
NA	2015	Fujian	Rice	*F. asiaticum*	NIV	27.13868	118.13741	NA	Farmer owned field

NA	2015	Fujian	Rice	*F. asiaticum*	15ADON	27.13868	118.13741	NA	Farmer owned field
NA	2015	Fujian	Rice	*F. asiaticum*	NIV	27.13868	118.13741	NA	Farmer owned field
NA	2015	Fujian	Rice	*F. asiaticum*	15ADON	27.13868	118.13741	NA	Farmer owned field
NA	2015	Fujian	Rice	*F. asiaticum*	15ADON	27.13868	118.13741	NA	Farmer owned field
NA	2015	Fujian	Rice	*F. asiaticum*	15ADON	27.13868	118.13741	NA	Farmer owned field
NA	2015	Fujian	Rice	*F. asiaticum*	NIV	27.13868	118.13741	NA	Farmer owned field
NA	2015	Fujian	Rice	*F. asiaticum*	15ADON	27.13868	118.13741	NA	Farmer owned field
NA	2015	Fujian	Rice	*F. asiaticum*	NIV	27.13868	118.13741	NA	Farmer owned field
NA	2015	Fujian	Rice	*F. asiaticum*	NIV	27.13868	118.13741	NA	Farmer owned field
NA	2015	Fujian	Rice	*F. asiaticum*	NIV	27.13868	118.13741	NA	Farmer owned field
NA	2015	Fujian	Rice	*F. asiaticum*	NIV	27.13868	118.13741	NA	Farmer owned field
NA	2015	Fujian	Rice	*F. asiaticum*	15ADON	27.13868	118.13741	NA	Farmer owned field
NA	2015	Fujian	Rice	*F. asiaticum*	15ADON	27.13868	118.13741	NA	Farmer owned field
NA	2015	Fujian	Rice	*F. asiaticum*	15ADON	27.13868	118.13741	NA	Farmer owned field
NA	2015	Fujian	Rice	*F. asiaticum*	NIV	27.13868	118.13741	NA	Farmer owned field
NA	2015	Fujian	Rice	*F. asiaticum*	NIV	27.13868	118.13741	NA	Farmer owned field
NA	2015	Fujian	Rice	*F. asiaticum*	15ADON	27.13868	118.13741	NA	Farmer owned field
NA	2015	Fujian	Rice	*F. asiaticum*	NIV	27.13868	118.13741	NA	Farmer owned field
NA	2015	Fujian	Rice	*F. asiaticum*	NIV	27.13868	118.13741	NA	Farmer owned field
NA	2015	Fujian	Rice	*F. asiaticum*	NIV	27.13868	118.13741	NA	Farmer owned field
NA	2015	Fujian	Rice	*F. asiaticum*	NIV	27.13868	118.13741	NA	Farmer owned field
NA	2015	Fujian	Rice	*F. asiaticum*	NIV	27.13868	118.13741	NA	Farmer owned field
NA	2015	Fujian	Rice	*F. asiaticum*	15ADON	27.13868	118.13741	NA	Farmer owned field
NA	2015	Fujian	Rice	*F. asiaticum*	NIV	27.13868	118.13741	NA	Farmer owned field
NA	2015	Fujian	Rice	*F. asiaticum*	NIV	27.13868	118.13741	NA	Farmer owned field
NA	2015	Fujian	Rice	*F. asiaticum*	NIV	27.13868	118.13741	NA	Farmer owned field
NA	2015	Fujian	Rice	*F. asiaticum*	NIV	27.13868	118.13741	NA	Farmer owned field
NA	2015	Fujian	Rice	*F. asiaticum*	15ADON	27.13868	118.13741	NA	Farmer owned field
NA	2015	Fujian	Rice	*F. asiaticum*	15ADON	27.13868	118.13741	NA	Farmer owned field
NA	2015	Fujian	Rice	*F. asiaticum*	NIV	27.13868	118.13741	NA	Farmer owned field
NA	2015	Fujian	Rice	*F. asiaticum*	15ADON	27.13868	118.13741	NA	Farmer owned field
NA	2015	Fujian	Rice	*F. asiaticum*	NIV	27.13868	118.13741	NA	Farmer owned field
NA	2015	Fujian	Rice	*F. asiaticum*	15ADON	27.13868	118.13741	NA	Farmer owned field
NA	2015	Fujian	Rice	*F. asiaticum*	15ADON	27.13868	118.13741	NA	Farmer owned field
NA	2015	Fujian	Rice	*F. asiaticum*	15ADON	27.13868	118.13741	NA	Farmer owned field

NA	2015	Fujian	Rice	*F. asiaticum*	NIV	27.13868	118.13741	NA	Farmer owned field
NA	2015	Fujian	Rice	*F. asiaticum*	15ADON	27.13868	118.13741	NA	Farmer owned field
NA	2015	Fujian	Rice	*F. asiaticum*	15ADON	27.13868	118.13741	NA	Farmer owned field
NA	2015	Fujian	Rice	*F. asiaticum*	NIV	27.13868	118.13741	NA	Farmer owned field
NA	2015	Fujian	Rice	*F. asiaticum*	15ADON	27.13868	118.13741	NA	Farmer owned field
NA	2015	Fujian	Rice	*F. asiaticum*	15ADON	27.13868	118.13741	NA	Farmer owned field
NA	2015	Fujian	Rice	*F. asiaticum*	NIV	27.13868	118.13741	NA	Farmer owned field
NA	2015	Fujian	Rice	*F. asiaticum*	15ADON	27.13868	118.13741	NA	Farmer owned field
NA	2015	Fujian	Rice	*F. asiaticum*	15ADON	27.13868	118.13741	NA	Farmer owned field
NA	2015	Fujian	Rice	*F. asiaticum*	NIV	27.13868	118.13741	NA	Farmer owned field
NA	2015	Fujian	Rice	*F. asiaticum*	15ADON	27.13868	118.13741	NA	Farmer owned field
NA	2015	Fujian	Rice	*F. asiaticum*	NIV	27.13868	118.13741	NA	Farmer owned field
NA	2015	Fujian	Rice	*F. asiaticum*	15ADON	27.13868	118.13741	NA	Farmer owned field
NA	2015	Fujian	Rice	*F. asiaticum*	15ADON	27.13868	118.13741	NA	Farmer owned field
NA	2015	Fujian	Rice	*F. asiaticum*	NIV	27.13868	118.13741	NA	Farmer owned field
NA	2015	Fujian	Rice	*F. asiaticum*	15ADON	27.13868	118.13741	NA	Farmer owned field
NA	2015	Fujian	Rice	*F. asiaticum*	15ADON	27.13868	118.13741	NA	Farmer owned field
NA	2015	Fujian	Rice	*F. asiaticum*	NIV	27.13868	118.13741	NA	Farmer owned field
NA	2015	Fujian	Rice	*F. asiaticum*	15ADON	27.13868	118.13741	NA	Farmer owned field
NA	2015	Fujian	Rice	*F. asiaticum*	15ADON	27.13868	118.13741	NA	Farmer owned field
171243	2017	Fujian	Rice	*F. asiaticum*	NIV	27.296453	117.34371	260.2	Farmer owned field
171242	2017	Fujian	Rice	*F. asiaticum*	NIV	27.296453	117.34371	260.2	Farmer owned field
171241	2017	Fujian	Rice	*F. asiaticum*	NIV	27.296453	117.34371	260.2	Farmer owned field
171240	2017	Fujian	Rice	*F. asiaticum*	NIV	27.296453	117.34371	260.2	Farmer owned field
171239	2017	Fujian	Rice	*F. asiaticum*	NIV	27.296453	117.34371	260.2	Farmer owned field
171238	2017	Fujian	Rice	*F. asiaticum*	NIV	27.296453	117.34371	260.2	Farmer owned field
171237	2017	Fujian	Rice	*F. asiaticum*	NIV	27.296453	117.34371	260.2	Farmer owned field
171236	2017	Fujian	Rice	*F. asiaticum*	NIV	27.296453	117.34371	260.2	Farmer owned field
171235	2017	Fujian	Rice	*F. asiaticum*	NIV	27.296453	117.34371	260.2	Farmer owned field
171234	2017	Fujian	Rice	*F. asiaticum*	NIV	27.296453	117.34371	260.2	Farmer owned field
171233	2017	Fujian	Rice	*F. asiaticum*	NIV	27.296453	117.34371	260.2	Farmer owned field
171232	2017	Fujian	Rice	*F. asiaticum*	NIV	27.296453	117.34371	260.2	Farmer owned field
171231	2017	Fujian	Rice	*F. asiaticum*	NIV	27.296453	117.34371	260.2	Farmer owned field
171230	2017	Fujian	Rice	*F. asiaticum*	NIV	27.296453	117.34371	260.2	Farmer owned field
171229	2017	Fujian	Rice	*F. asiaticum*	NIV	27.296453	117.34371	260.2	Farmer owned field

171228	2017	Fujian	Rice	*F. asiaticum*	NIV	27.296453	117.34371	260.2	Farmer owned field
171227	2017	Fujian	Rice	*F. asiaticum*	NIV	27.296453	117.34371	260.2	Farmer owned field
171226	2017	Fujian	Rice	*F. asiaticum*	NIV	27.296453	117.34371	260.2	Farmer owned field
171225	2017	Fujian	Rice	*F. asiaticum*	NIV	27.296453	117.34371	260.2	Farmer owned field
171224	2017	Fujian	Rice	*F. asiaticum*	NIV	27.296453	117.34371	260.2	Farmer owned field
171223	2017	Fujian	Rice	*F. asiaticum*	NIV	27.296453	117.34371	260.2	Farmer owned field
171222	2017	Fujian	Rice	*F. asiaticum*	NIV	27.296453	117.34371	260.2	Farmer owned field
171219	2017	Fujian	Rice	*F. asiaticum*	NIV	27.296453	117.34371	260.2	Farmer owned field
171218	2017	Fujian	Rice	*F. asiaticum*	NIV	27.296453	117.34371	260.2	Farmer owned field
171217	2017	Fujian	Rice	*F. asiaticum*	NIV	27.296453	117.34371	260.2	Farmer owned field
171216	2017	Fujian	Rice	*F. asiaticum*	NIV	27.296453	117.34371	260.2	Farmer owned field
171215	2017	Fujian	Rice	*F. asiaticum*	NIV	27.296453	117.34371	260.2	Farmer owned field
171214	2017	Fujian	Rice	*F. asiaticum*	NIV	27.296453	117.34371	260.2	Farmer owned field
171213	2017	Fujian	Rice	*F. asiaticum*	NIV	27.296453	117.34371	260.2	Farmer owned field
171212	2017	Fujian	Rice	*F. asiaticum*	15ADON	27.296453	117.34371	260.2	Farmer owned field
171211	2017	Fujian	Rice	*F. asiaticum*	NIV	27.296453	117.34371	260.2	Farmer owned field
171210	2017	Fujian	Rice	*F. asiaticum*	NIV	27.296453	117.34371	260.2	Farmer owned field
171209	2017	Fujian	Rice	*F. asiaticum*	NIV	27.296453	117.34371	260.2	Farmer owned field
171208	2017	Fujian	Rice	*F. asiaticum*	NIV	27.296453	117.34371	260.2	Farmer owned field
171207	2017	Fujian	Rice	*F. asiaticum*	NIV	27.296453	117.34371	260.2	Farmer owned field
171169	2017	Fujian	Rice	*F. asiaticum*	NIV	27.516527	118.04489	156.4	Farmer owned field
171168	2017	Fujian	Rice	*F. asiaticum*	NIV	27.516527	118.04489	156.4	Farmer owned field
171167	2017	Fujian	Rice	*F. asiaticum*	NIV	27.516527	118.04489	156.4	Farmer owned field
171166	2017	Fujian	Rice	*F. asiaticum*	15ADON	27.516527	118.04489	156.4	Farmer owned field
171165	2017	Fujian	Rice	*F. asiaticum*	15ADON	27.516527	118.04489	156.4	Farmer owned field
171164	2017	Fujian	Rice	*F. asiaticum*	15ADON	27.516527	118.04489	156.4	Farmer owned field
171163	2017	Fujian	Rice	*F. asiaticum*	15ADON	27.516527	118.04489	156.4	Farmer owned field
171162	2017	Fujian	Rice	*F. asiaticum*	15ADON	27.516527	118.04489	156.4	Farmer owned field
171161	2017	Fujian	Rice	*F. asiaticum*	15ADON	27.516527	118.04489	156.4	Farmer owned field
171160	2017	Fujian	Rice	*F. asiaticum*	NIV	27.516527	118.04489	156.4	Farmer owned field
171159	2017	Fujian	Rice	*F. asiaticum*	NIV	27.516527	118.04489	156.4	Farmer owned field
171158	2017	Fujian	Rice	*F. asiaticum*	NIV	27.516527	118.04489	156.4	Farmer owned field
171157	2017	Fujian	Rice	*F. asiaticum*	NIV	27.516527	118.04489	156.4	Farmer owned field
171156	2017	Fujian	Rice	*F. asiaticum*	NIV	27.516527	118.04489	156.4	Farmer owned field
171155	2017	Fujian	Rice	*F. asiaticum*	NIV	27.516527	118.04489	156.4	Farmer owned field

171154	2017	Fujian	Rice	*F. asiaticum*	NIV	27.516527	118.04489	156.4	Farmer owned field
171153	2017	Fujian	Rice	*F. asiaticum*	NIV	27.516527	118.04489	156.4	Farmer owned field
171152	2017	Fujian	Rice	*F. asiaticum*	NIV	27.516527	118.04489	156.4	Farmer owned field
171151	2017	Fujian	Rice	*F. asiaticum*	NIV	27.516527	118.04489	156.4	Farmer owned field
171150	2017	Fujian	Rice	*F. asiaticum*	NIV	27.532357	118.080683	154.7	Farmer owned field
171149	2017	Fujian	Rice	*F. asiaticum*	NIV	27.532357	118.080683	154.7	Farmer owned field
171148	2017	Fujian	Rice	*F. asiaticum*	NIV	27.532357	118.080683	154.7	Farmer owned field
171147	2017	Fujian	Rice	*F. asiaticum*	NIV	27.532357	118.080683	154.7	Farmer owned field
171146	2017	Fujian	Rice	*F. asiaticum*	NIV	27.532357	118.080683	154.7	Farmer owned field
171145	2017	Fujian	Rice	*F. asiaticum*	NIV	27.532357	118.080683	154.7	Farmer owned field
171144	2017	Fujian	Rice	*F. asiaticum*	NIV	27.532357	118.080683	154.7	Farmer owned field
171142	2017	Fujian	Rice	*F. asiaticum*	NIV	27.532357	118.080683	154.7	Farmer owned field
171141	2017	Fujian	Rice	*F. asiaticum*	NIV	27.532357	118.080683	154.7	Farmer owned field
171140	2017	Fujian	Rice	*F. asiaticum*	NIV	27.532357	118.080683	154.7	Farmer owned field
171139	2017	Fujian	Rice	*F. asiaticum*	NIV	27.532357	118.080683	154.7	Farmer owned field
171138	2017	Fujian	Rice	*F. asiaticum*	NIV	27.532357	118.080683	154.7	Farmer owned field
171137	2017	Fujian	Rice	*F. asiaticum*	NIV	27.532357	118.080683	154.7	Farmer owned field
171135	2017	Fujian	Rice	*F. asiaticum*	NIV	27.532357	118.080683	154.7	Farmer owned field
171134	2017	Fujian	Rice	*F. asiaticum*	NIV	27.532357	118.080683	154.7	Farmer owned field
171133	2017	Fujian	Rice	*F. asiaticum*	NIV	27.532357	118.080683	154.7	Farmer owned field
171132	2017	Fujian	Rice	*F. asiaticum*	NIV	27.532357	118.080683	154.7	Farmer owned field
171131	2017	Fujian	Rice	*F. asiaticum*	NIV	27.532357	118.080683	154.7	Farmer owned field
171130	2017	Fujian	Rice	*F. asiaticum*	15ADON	27.532357	118.080683	154.7	Farmer owned field
171129	2017	Fujian	Rice	*F. asiaticum*	NIV	27.532357	118.080683	154.7	Farmer owned field
171128	2017	Fujian	Rice	*F. asiaticum*	NIV	27.532357	118.080683	154.7	Farmer owned field
171127	2017	Fujian	Rice	*F. asiaticum*	NIV	27.532357	118.080683	154.7	Farmer owned field
171126	2017	Fujian	Rice	*F. asiaticum*	NIV	27.532357	118.080683	154.7	Farmer owned field
171125	2017	Fujian	Rice	*F. asiaticum*	NIV	27.532357	118.080683	154.7	Farmer owned field
171124	2017	Fujian	Rice	*F. asiaticum*	NIV	27.532357	118.080683	154.7	Farmer owned field
171123	2017	Fujian	Rice	*F. asiaticum*	NIV	27.532357	118.080683	154.7	Farmer owned field
171122	2017	Fujian	Rice	*F. asiaticum*	NIV	27.532357	118.080683	154.7	Farmer owned field
171121	2017	Fujian	Rice	*F. asiaticum*	NIV	27.532357	118.080683	154.7	Farmer owned field
171120	2017	Fujian	Rice	*F. asiaticum*	NIV	27.532357	118.080683	154.7	Farmer owned field
171206	2017	Fujian	Rice	*F. asiaticum*	NIV	28.01743	118.482725	313.6	Farmer owned field
171205	2017	Fujian	Rice	*F. asiaticum*	15ADON	28.01743	118.482725	313.6	Farmer owned field

171203	2017	Fujian	Rice	*F. asiaticum*	NIV	28.01743	118.482725	313.6	Farmer owned field
171202	2017	Fujian	Rice	*F. asiaticum*	NIV	28.01743	118.482725	313.6	Farmer owned field
171201	2017	Fujian	Rice	*F. asiaticum*	NIV	28.01743	118.482725	313.6	Farmer owned field
171200	2017	Fujian	Rice	*F. asiaticum*	NIV	28.01743	118.482725	313.6	Farmer owned field
171199	2017	Fujian	Rice	*F. asiaticum*	NIV	28.01743	118.482725	313.6	Farmer owned field
171198	2017	Fujian	Rice	*F. asiaticum*	NIV	28.01743	118.482725	313.6	Farmer owned field
171196	2017	Fujian	Rice	*F. asiaticum*	NIV	28.01743	118.482725	313.6	Farmer owned field
171195	2017	Fujian	Rice	*F. asiaticum*	NIV	28.01743	118.482725	313.6	Farmer owned field
171194	2017	Fujian	Rice	*F. asiaticum*	NIV	28.01743	118.482725	313.6	Farmer owned field
171193	2017	Fujian	Rice	*F. asiaticum*	NIV	28.01743	118.482725	313.6	Farmer owned field
171192	2017	Fujian	Rice	*F. asiaticum*	NIV	28.01743	118.482725	313.6	Farmer owned field
171191	2017	Fujian	Rice	*F. asiaticum*	NIV	28.01743	118.482725	313.6	Farmer owned field
171190	2017	Fujian	Rice	*F. asiaticum*	NIV	28.01743	118.482725	313.6	Farmer owned field
171189	2017	Fujian	Rice	*F. asiaticum*	NIV	28.01743	118.482725	313.6	Farmer owned field
171188	2017	Fujian	Rice	*F. asiaticum*	15ADON	28.01743	118.482725	313.6	Farmer owned field
171186	2017	Fujian	Rice	*F. asiaticum*	NIV	28.01743	118.482725	313.6	Farmer owned field
171185	2017	Fujian	Rice	*F. asiaticum*	NIV	28.01743	118.482725	313.6	Farmer owned field
171184	2017	Fujian	Rice	*F. asiaticum*	NIV	28.01743	118.482725	313.6	Farmer owned field
171183	2017	Fujian	Rice	*F. asiaticum*	NIV	28.01743	118.482725	313.6	Farmer owned field
171182	2017	Fujian	Rice	*F. asiaticum*	NIV	28.01743	118.482725	313.6	Farmer owned field
171181	2017	Fujian	Rice	*F. asiaticum*	NIV	28.01743	118.482725	313.6	Farmer owned field
171180	2017	Fujian	Rice	*F. asiaticum*	NIV	28.01743	118.482725	313.6	Farmer owned field
171179	2017	Fujian	Rice	*F. asiaticum*	NIV	28.01743	118.482725	313.6	Farmer owned field
171178	2017	Fujian	Rice	*F. asiaticum*	NIV	28.01743	118.482725	313.6	Farmer owned field
171177	2017	Fujian	Rice	*F. asiaticum*	NIV	28.01743	118.482725	313.6	Farmer owned field
171176	2017	Fujian	Rice	*F. asiaticum*	NIV	28.01743	118.482725	313.6	Farmer owned field
171175	2017	Fujian	Rice	*F. asiaticum*	NIV	28.01743	118.482725	313.6	Farmer owned field
171174	2017	Fujian	Rice	*F. asiaticum*	NIV	28.01743	118.482725	313.6	Farmer owned field
171173	2017	Fujian	Rice	*F. asiaticum*	NIV	28.01743	118.482725	313.6	Farmer owned field
171172	2017	Fujian	Rice	*F. asiaticum*	NIV	28.01743	118.482725	313.6	Farmer owned field
171171	2017	Fujian	Rice	*F. asiaticum*	NIV	28.01743	118.482725	313.6	Farmer owned field
171170	2017	Fujian	Rice	*F. asiaticum*	NIV	28.01743	118.482725	313.6	Farmer owned field
170962	2017	Hunan	Rice	*F. asiaticum*	3ADON	28.19918202	112.683053	93.43	Farmer owned field
170961	2017	Hunan	Rice	*F. asiaticum*	NIV	28.19918202	112.683053	93.43	Farmer owned field
170960	2017	Hunan	Rice	*F. asiaticum*	3ADON	28.19918202	112.683053	93.43	Farmer owned field

170958	2017	Hunan	Rice	*F. asiaticum*	NIV	28.19918202	112.683053	93.43	Farmer owned field
170957	2017	Hunan	Rice	*F. asiaticum*	NIV	28.19918202	112.683053	93.43	Farmer owned field
170956	2017	Hunan	Rice	*F. asiaticum*	NIV	28.19918202	112.683053	93.43	Farmer owned field
170955	2017	Hunan	Rice	*F. asiaticum*	NIV	28.19918202	112.683053	93.43	Farmer owned field
170954	2017	Hunan	Rice	*F. asiaticum*	NIV	28.19918202	112.683053	93.43	Farmer owned field
170953	2017	Hunan	Rice	*F. asiaticum*	NIV	28.19918202	112.683053	93.43	Farmer owned field
170379	2017	Hunan	Rice	*F. asiaticum*	3ADON	28.62087197	112.339678	34.65	Farmer owned field
170378	2017	Hunan	Rice	*F. asiaticum*	NIV	28.62087197	112.339678	34.65	Farmer owned field
170377	2017	Hunan	Rice	*F. asiaticum*	15ADON	28.62087197	112.339678	34.65	Farmer owned field
170376	2017	Hunan	Rice	*F. asiaticum*	15ADON	28.62087197	112.339678	34.65	Farmer owned field
170375	2017	Hunan	Rice	*F. asiaticum*	NIV	28.62087197	112.339678	34.65	Farmer owned field
170374	2017	Hunan	Rice	*F. asiaticum*	NIV	28.62087197	112.339678	34.65	Farmer owned field
170373	2017	Hunan	Rice	*F. asiaticum*	NIV	28.62087197	112.339678	34.65	Farmer owned field
170372	2017	Hunan	Rice	*F. asiaticum*	NIV	28.62087197	112.339678	34.65	Farmer owned field
170371	2017	Hunan	Rice	*F. asiaticum*	NIV	28.62087197	112.339678	34.65	Farmer owned field
170370	2017	Hunan	Rice	*F. asiaticum*	3ADON	28.62087197	112.339678	34.65	Farmer owned field
170369	2017	Hunan	Rice	*F. asiaticum*	15ADON	28.62087197	112.339678	34.65	Farmer owned field
170368	2017	Hunan	Rice	*F. asiaticum*	15ADON	28.62087197	112.339678	34.65	Farmer owned field
170367	2017	Hunan	Rice	*F. asiaticum*	NIV	28.62087197	112.339678	34.65	Farmer owned field
170366	2017	Hunan	Rice	*F. asiaticum*	NIV	28.62087197	112.339678	34.65	Farmer owned field
170365	2017	Hunan	Rice	*F. asiaticum*	NIV	28.62087197	112.339678	34.65	Farmer owned field
170364	2017	Hunan	Rice	*F. asiaticum*	3ADON	28.62087197	112.339678	34.65	Farmer owned field
170363	2017	Hunan	Rice	*F. asiaticum*	15ADON	28.62087197	112.339678	34.65	Farmer owned field
170362	2017	Hunan	Rice	*F. asiaticum*	3ADON	28.62087197	112.339678	34.65	Farmer owned field
170361	2017	Hunan	Rice	*F. asiaticum*	3ADON	28.62087197	112.339678	34.65	Farmer owned field
170360	2017	Hunan	Rice	*F. asiaticum*	15ADON	28.62087197	112.339678	34.65	Farmer owned field
170358	2017	Hunan	Rice	*F. asiaticum*	NIV	28.62087197	112.339678	34.65	Farmer owned field
170357	2017	Hunan	Rice	*F. asiaticum*	NIV	28.62087197	112.339678	34.65	Farmer owned field
170356	2017	Hunan	Rice	*F. asiaticum*	3ADON	28.62087197	112.339678	34.65	Farmer owned field
170355	2017	Hunan	Rice	*F. asiaticum*	NIV	28.62087197	112.339678	34.65	Farmer owned field
170354	2017	Hunan	Rice	*F. asiaticum*	NIV	28.62087197	112.339678	34.65	Farmer owned field
170352	2017	Hunan	Rice	*F. asiaticum*	NIV	28.62087197	112.339678	34.65	Farmer owned field
170351	2017	Hunan	Rice	*F. asiaticum*	3ADON	28.62087197	112.339678	34.65	Farmer owned field
170350	2017	Hunan	Rice	*F. asiaticum*	3ADON	28.62087197	112.339678	34.65	Farmer owned field
170349	2017	Hunan	Rice	*F. asiaticum*	NIV	28.62087197	112.339678	34.65	Farmer owned field

170348	2017	Hunan	Rice	*F. asiaticum*	3ADON	28.62087197	112.339678	34.65	Farmer owned field
170347	2017	Hunan	Rice	*F. asiaticum*	3ADON	28.62087197	112.339678	34.65	Farmer owned field
170346	2017	Hunan	Rice	*F. asiaticum*	NIV	28.62087197	112.339678	34.65	Farmer owned field
170345	2017	Hunan	Rice	*F. asiaticum*	NIV	28.62087197	112.339678	34.65	Farmer owned field
170343	2017	Hunan	Rice	*F. asiaticum*	NIV	28.62087197	112.339678	34.65	Farmer owned field
193851	2019	Hunan	Rice	*F. asiaticum*	3ADON	28.73773397	112.32034	42.570229	Farmer owned field
193850	2019	Hunan	Rice	*F. asiaticum*	3ADON	28.73773397	112.32034	42.570229	Farmer owned field
193849	2019	Hunan	Rice	*F. asiaticum*	NIV	28.73773397	112.32034	42.570229	Farmer owned field
193848	2019	Hunan	Rice	*F. asiaticum*	NIV	28.73773397	112.32034	42.570229	Farmer owned field
193847	2019	Hunan	Rice	*F. asiaticum*	3ADON	28.73773397	112.32034	42.570229	Farmer owned field
193846	2019	Hunan	Rice	*F. asiaticum*	NIV	28.73773397	112.32034	42.570229	Farmer owned field
193845	2019	Hunan	Rice	*F. asiaticum*	NIV	28.73773397	112.32034	42.570229	Farmer owned field
193844	2019	Hunan	Rice	*F. asiaticum*	NIV	28.73773397	112.32034	42.570229	Farmer owned field
193842	2019	Hunan	Rice	*F. asiaticum*	15ADON	28.73773397	112.32034	42.570229	Farmer owned field
193840	2019	Hunan	Rice	*F. asiaticum*	3ADON	28.73773397	112.32034	42.570229	Farmer owned field
193839	2019	Hunan	Rice	*F. asiaticum*	NIV	28.73773397	112.32034	42.570229	Farmer owned field
193837	2019	Hunan	Rice	*F. asiaticum*	3ADON	28.73773397	112.32034	42.570229	Farmer owned field
193836	2019	Hunan	Rice	*F. asiaticum*	15ADON	28.73773397	112.32034	42.570229	Farmer owned field
193834	2019	Hunan	Rice	*F. asiaticum*	3ADON	28.73773397	112.32034	42.570229	Farmer owned field
193831	2019	Hunan	Rice	*F. asiaticum*	NIV	28.73773397	112.32034	42.570229	Farmer owned field
193829	2019	Hunan	Rice	*F. asiaticum*	3ADON	28.73773397	112.32034	42.570229	Farmer owned field
170429	2017	Hunan	Rice	*F. asiaticum*	NIV	28.73773397	112.32034	NA	Farmer owned field
170428	2017	Hunan	Rice	*F. asiaticum*	3ADON	28.73773397	112.32034	NA	Farmer owned field
170427	2017	Hunan	Rice	*F. asiaticum*	NIV	28.73773397	112.32034	NA	Farmer owned field
170426	2017	Hunan	Rice	*F. asiaticum*	3ADON	28.73773397	112.32034	NA	Farmer owned field
170425	2017	Hunan	Rice	*F. asiaticum*	3ADON	28.73773397	112.32034	NA	Farmer owned field
170424	2017	Hunan	Rice	*F. asiaticum*	NIV	28.73773397	112.32034	NA	Farmer owned field
170423	2017	Hunan	Rice	*F. asiaticum*	NIV	28.73773397	112.32034	NA	Farmer owned field
170422	2017	Hunan	Rice	*F. asiaticum*	3ADON	28.73773397	112.32034	NA	Farmer owned field
170421	2017	Hunan	Rice	*F. asiaticum*	15ADON	28.73773397	112.32034	NA	Farmer owned field
170420	2017	Hunan	Rice	*F. asiaticum*	NIV	28.73773397	112.32034	NA	Farmer owned field
170419	2017	Hunan	Rice	*F. asiaticum*	3ADON	28.73773397	112.32034	NA	Farmer owned field
170418	2017	Hunan	Rice	*F. asiaticum*	3ADON	28.73773397	112.32034	NA	Farmer owned field
170417	2017	Hunan	Rice	*F. asiaticum*	NIV	28.73773397	112.32034	NA	Farmer owned field
170416	2017	Hunan	Rice	*F. asiaticum*	NIV	28.73773397	112.32034	NA	Farmer owned field

170415	2017	Hunan	Rice	*F. asiaticum*	3ADON	28.73773397	112.32034	NA	Farmer owned field
170414	2017	Hunan	Rice	*F. asiaticum*	NIV	28.73773397	112.32034	NA	Farmer owned field
170413	2017	Hunan	Rice	*F. asiaticum*	15ADON	28.73773397	112.32034	NA	Farmer owned field
170412	2017	Hunan	Rice	*F. asiaticum*	3ADON	28.73773397	112.32034	NA	Farmer owned field
170411	2017	Hunan	Rice	*F. asiaticum*	3ADON	28.73773397	112.32034	NA	Farmer owned field
170410	2017	Hunan	Rice	*F. asiaticum*	15ADON	28.73773397	112.32034	NA	Farmer owned field
170409	2017	Hunan	Rice	*F. asiaticum*	15ADON	28.73773397	112.32034	NA	Farmer owned field
170408	2017	Hunan	Rice	*F. asiaticum*	3ADON	28.73773397	112.32034	NA	Farmer owned field
170407	2017	Hunan	Rice	*F. asiaticum*	15ADON	28.73773397	112.32034	NA	Farmer owned field
170406	2017	Hunan	Rice	*F. asiaticum*	3ADON	28.73773397	112.32034	NA	Farmer owned field
170405	2017	Hunan	Rice	*F. asiaticum*	3ADON	28.73773397	112.32034	NA	Farmer owned field
170404	2017	Hunan	Rice	*F. asiaticum*	3ADON	28.73773397	112.32034	NA	Farmer owned field
170403	2017	Hunan	Rice	*F. asiaticum*	15ADON	28.73773397	112.32034	NA	Farmer owned field
170402	2017	Hunan	Rice	*F. asiaticum*	NIV	28.73773397	112.32034	NA	Farmer owned field
170401	2017	Hunan	Rice	*F. asiaticum*	NIV	28.73773397	112.32034	NA	Farmer owned field
170400	2017	Hunan	Rice	*F. asiaticum*	3ADON	28.73773397	112.32034	NA	Farmer owned field
170399	2017	Hunan	Rice	*F. asiaticum*	3ADON	28.73773397	112.32034	NA	Farmer owned field
170398	2017	Hunan	Rice	*F. asiaticum*	3ADON	28.73773397	112.32034	NA	Farmer owned field
170397	2017	Hunan	Rice	*F. asiaticum*	3ADON	28.73773397	112.32034	NA	Farmer owned field
170396	2017	Hunan	Rice	*F. asiaticum*	NIV	28.73773397	112.32034	NA	Farmer owned field
170395	2017	Hunan	Rice	*F. asiaticum*	NIV	28.73773397	112.32034	NA	Farmer owned field
170394	2017	Hunan	Rice	*F. asiaticum*	NIV	28.73773397	112.32034	NA	Farmer owned field
170393	2017	Hunan	Rice	*F. asiaticum*	3ADON	28.73773397	112.32034	NA	Farmer owned field
170392	2017	Hunan	Rice	*F. asiaticum*	3ADON	28.73773397	112.32034	NA	Farmer owned field
170391	2017	Hunan	Rice	*F. asiaticum*	15ADON	28.73773397	112.32034	NA	Farmer owned field
170390	2017	Hunan	Rice	*F. asiaticum*	3ADON	28.73773397	112.32034	NA	Farmer owned field
170389	2017	Hunan	Rice	*F. asiaticum*	3ADON	28.73773397	112.32034	NA	Farmer owned field
170388	2017	Hunan	Rice	*F. asiaticum*	NIV	28.73773397	112.32034	NA	Farmer owned field
170387	2017	Hunan	Rice	*F. asiaticum*	3ADON	28.73773397	112.32034	NA	Farmer owned field
170386	2017	Hunan	Rice	*F. asiaticum*	3ADON	28.73773397	112.32034	NA	Farmer owned field
170385	2017	Hunan	Rice	*F. asiaticum*	3ADON	28.73773397	112.32034	NA	Farmer owned field
170384	2017	Hunan	Rice	*F. asiaticum*	3ADON	28.73773397	112.32034	NA	Farmer owned field
170383	2017	Hunan	Rice	*F. asiaticum*	NIV	28.73773397	112.32034	NA	Farmer owned field
170382	2017	Hunan	Rice	*F. asiaticum*	3ADON	28.73773397	112.32034	NA	Farmer owned field
170943	2017	Hunan	Rice	*F. asiaticum*	3ADON	28.793654	113.167283	40.16	Farmer owned field

170942	2017	Hunan	Rice	*F. asiaticum*	NIV	28.793654	113.167283	40.16	Farmer owned field
170941	2017	Hunan	Rice	*F. asiaticum*	NIV	28.793654	113.167283	40.16	Farmer owned field
170940	2017	Hunan	Rice	*F. asiaticum*	NIV	28.793654	113.167283	40.16	Farmer owned field
170939	2017	Hunan	Rice	*F. asiaticum*	NIV	28.793654	113.167283	40.16	Farmer owned field
170938	2017	Hunan	Rice	*F. asiaticum*	NIV	28.793654	113.167283	40.16	Farmer owned field
170937	2017	Hunan	Rice	*F. asiaticum*	NIV	28.793654	113.167283	40.16	Farmer owned field
170936	2017	Hunan	Rice	*F. asiaticum*	NIV	28.793654	113.167283	40.16	Farmer owned field
170935	2017	Hunan	Rice	*F. asiaticum*	3ADON	28.793654	113.167283	40.16	Farmer owned field
170934	2017	Hunan	Rice	*F. asiaticum*	NIV	28.793654	113.167283	40.16	Farmer owned field
170933	2017	Hunan	Rice	*F. asiaticum*	NIV	28.793654	113.167283	40.16	Farmer owned field
170932	2017	Hunan	Rice	*F. asiaticum*	3ADON	28.793654	113.167283	40.16	Farmer owned field
170931	2017	Hunan	Rice	*F. asiaticum*	3ADON	28.793654	113.167283	40.16	Farmer owned field
170930	2017	Hunan	Rice	*F. asiaticum*	NIV	28.793654	113.167283	40.16	Farmer owned field
170929	2017	Hunan	Rice	*F. asiaticum*	3ADON	28.793654	113.167283	40.16	Farmer owned field
170928	2017	Hunan	Rice	*F. asiaticum*	NIV	28.793654	113.167283	40.16	Farmer owned field
170927	2017	Hunan	Rice	*F. asiaticum*	NIV	28.793654	113.167283	40.16	Farmer owned field
170926	2017	Hunan	Rice	*F. asiaticum*	3ADON	28.793654	113.167283	40.16	Farmer owned field
170924	2017	Hunan	Rice	*F. asiaticum*	NIV	28.793654	113.167283	40.16	Farmer owned field
170923	2017	Hunan	Rice	*F. asiaticum*	NIV	28.793654	113.167283	40.16	Farmer owned field
170922	2017	Hunan	Rice	*F. asiaticum*	3ADON	28.793654	113.167283	40.16	Farmer owned field
170921	2017	Hunan	Rice	*F. asiaticum*	15ADON	28.793654	113.167283	40.16	Farmer owned field
170920	2017	Hunan	Rice	*F. asiaticum*	3ADON	28.793654	113.167283	40.16	Farmer owned field
170919	2017	Hunan	Rice	*F. asiaticum*	NIV	28.793654	113.167283	40.16	Farmer owned field
170918	2017	Hunan	Rice	*F. asiaticum*	NIV	28.793654	113.167283	40.16	Farmer owned field
170916	2017	Hunan	Rice	*F. asiaticum*	NIV	28.793654	113.167283	40.16	Farmer owned field
170915	2017	Hunan	Rice	*F. asiaticum*	NIV	28.793654	113.167283	40.16	Farmer owned field
170914	2017	Hunan	Rice	*F. asiaticum*	3ADON	28.793654	113.167283	40.16	Farmer owned field
170913	2017	Hunan	Rice	*F. asiaticum*	NIV	28.793654	113.167283	40.16	Farmer owned field
170912	2017	Hunan	Rice	*F. asiaticum*	3ADON	28.793654	113.167283	40.16	Farmer owned field
170911	2017	Hunan	Rice	*F. asiaticum*	NIV	28.793654	113.167283	40.16	Farmer owned field
170910	2017	Hunan	Rice	*F. asiaticum*	NIV	28.793654	113.167283	40.16	Farmer owned field
170909	2017	Hunan	Rice	*F. asiaticum*	NIV	28.793654	113.167283	40.16	Farmer owned field
170908	2017	Hunan	Rice	*F. asiaticum*	NIV	28.793654	113.167283	40.16	Farmer owned field
170907	2017	Hunan	Rice	*F. asiaticum*	3ADON	28.793654	113.167283	40.16	Farmer owned field
193947	2019	Hunan	Rice	*F. asiaticum*	3ADON	28.883176	112.295044	40.6068	Farmer owned field

193944	2019	Hunan	Rice	*F. asiaticum*	NIV	28.883176	112.295044	40.6068	Farmer owned field
193943	2019	Hunan	Rice	*F. asiaticum*	15ADON	28.883176	112.295044	40.6068	Farmer owned field
193942	2019	Hunan	Rice	*F. asiaticum*	NIV	28.883176	112.295044	40.6068	Farmer owned field
193941	2019	Hunan	Rice	*F. asiaticum*	NIV	28.883176	112.295044	40.6068	Farmer owned field
193940	2019	Hunan	Rice	*F. asiaticum*	NIV	28.883176	112.295044	40.6068	Farmer owned field
193938	2019	Hunan	Rice	*F. asiaticum*	NIV	28.883176	112.295044	40.6068	Farmer owned field
193934	2019	Hunan	Rice	*F. asiaticum*	3ADON	28.883176	112.295044	40.6068	Farmer owned field
193933	2019	Hunan	Rice	*F. asiaticum*	NIV	28.883176	112.295044	40.6068	Farmer owned field
170458	2017	Hunan	Rice	*F. asiaticum*	3ADON	28.883176	112.295044	NA	Farmer owned field
170457	2017	Hunan	Rice	*F. asiaticum*	3ADON	28.883176	112.295044	NA	Farmer owned field
170456	2017	Hunan	Rice	*F. asiaticum*	NIV	28.883176	112.295044	NA	Farmer owned field
170455	2017	Hunan	Rice	*F. asiaticum*	NIV	28.883176	112.295044	NA	Farmer owned field
170454	2017	Hunan	Rice	*F. asiaticum*	3ADON	28.883176	112.295044	NA	Farmer owned field
170453	2017	Hunan	Rice	*F. asiaticum*	3ADON	28.883176	112.295044	NA	Farmer owned field
170452	2017	Hunan	Rice	*F. asiaticum*	3ADON	28.883176	112.295044	NA	Farmer owned field
170451	2017	Hunan	Rice	*F. asiaticum*	NIV	28.883176	112.295044	NA	Farmer owned field
170450	2017	Hunan	Rice	*F. asiaticum*	NIV	28.883176	112.295044	NA	Farmer owned field
170449	2017	Hunan	Rice	*F. asiaticum*	3ADON	28.883176	112.295044	NA	Farmer owned field
170448	2017	Hunan	Rice	*F. asiaticum*	NIV	28.883176	112.295044	NA	Farmer owned field
170447	2017	Hunan	Rice	*F. asiaticum*	3ADON	28.883176	112.295044	NA	Farmer owned field
170446	2017	Hunan	Rice	*F. asiaticum*	3ADON	28.883176	112.295044	NA	Farmer owned field
170445	2017	Hunan	Rice	*F. asiaticum*	3ADON	28.883176	112.295044	NA	Farmer owned field
170444	2017	Hunan	Rice	*F. asiaticum*	NIV	28.883176	112.295044	NA	Farmer owned field
170443	2017	Hunan	Rice	*F. asiaticum*	NIV	28.883176	112.295044	NA	Farmer owned field
170442	2017	Hunan	Rice	*F. asiaticum*	15ADON	28.883176	112.295044	NA	Farmer owned field
170441	2017	Hunan	Rice	*F. asiaticum*	NIV	28.883176	112.295044	NA	Farmer owned field
170440	2017	Hunan	Rice	*F. asiaticum*	NIV	28.883176	112.295044	NA	Farmer owned field
170439	2017	Hunan	Rice	*F. asiaticum*	3ADON	28.883176	112.295044	NA	Farmer owned field
170438	2017	Hunan	Rice	*F. asiaticum*	NIV	28.883176	112.295044	NA	Farmer owned field
170437	2017	Hunan	Rice	*F. asiaticum*	NIV	28.883176	112.295044	NA	Farmer owned field
170436	2017	Hunan	Rice	*F. asiaticum*	3ADON	28.883176	112.295044	NA	Farmer owned field
170435	2017	Hunan	Rice	*F. asiaticum*	3ADON	28.883176	112.295044	NA	Farmer owned field
170434	2017	Hunan	Rice	*F. asiaticum*	3ADON	28.883176	112.295044	NA	Farmer owned field
170432	2017	Hunan	Rice	*F. asiaticum*	3ADON	28.883176	112.295044	NA	Farmer owned field
170431	2017	Hunan	Rice	*F. asiaticum*	NIV	28.883176	112.295044	NA	Farmer owned field

170430	2017	Hunan	Rice	*F. asiaticum*	3ADON	28.883176	112.295044	NA	Farmer owned field
194094	2019	Hunan	Rice	*F. asiaticum*	NIV	28.93416203	113.242577	60.157688	Farmer owned field
194093	2019	Hunan	Rice	*F. asiaticum*	3ADON	28.93416203	113.242577	60.157688	Farmer owned field
194092	2019	Hunan	Rice	*F. asiaticum*	NIV	28.93416203	113.242577	60.157688	Farmer owned field
194091	2019	Hunan	Rice	*F. asiaticum*	NIV	28.93416203	113.242577	60.157688	Farmer owned field
194090	2019	Hunan	Rice	*F. asiaticum*	NIV	28.93416203	113.242577	60.157688	Farmer owned field
194089	2019	Hunan	Rice	*F. asiaticum*	NIV	28.93416203	113.242577	60.157688	Farmer owned field
194088	2019	Hunan	Rice	*F. asiaticum*	3ADON	28.93416203	113.242577	60.157688	Farmer owned field
194087	2019	Hunan	Rice	*F. asiaticum*	3ADON	28.93416203	113.242577	60.157688	Farmer owned field
194086	2019	Hunan	Rice	*F. asiaticum*	NIV	28.93416203	113.242577	60.157688	Farmer owned field
194085	2019	Hunan	Rice	*F. asiaticum*	3ADON	28.93416203	113.242577	60.157688	Farmer owned field
194084	2019	Hunan	Rice	*F. asiaticum*	3ADON	28.93416203	113.242577	60.157688	Farmer owned field
194083	2019	Hunan	Rice	*F. asiaticum*	3ADON	28.93416203	113.242577	60.157688	Farmer owned field
194082	2019	Hunan	Rice	*F. asiaticum*	3ADON	28.93416203	113.242577	60.157688	Farmer owned field
194081	2019	Hunan	Rice	*F. asiaticum*	NIV	28.93416203	113.242577	60.157688	Farmer owned field
194080	2019	Hunan	Rice	*F. asiaticum*	3ADON	28.93416203	113.242577	60.157688	Farmer owned field
170906	2017	Hunan	Rice	*F. asiaticum*	NIV	28.93416203	113.242577	58.17	Farmer owned field
170905	2017	Hunan	Rice	*F. asiaticum*	NIV	28.93416203	113.242577	58.17	Farmer owned field
170904	2017	Hunan	Rice	*F. asiaticum*	NIV	28.93416203	113.242577	58.17	Farmer owned field
170903	2017	Hunan	Rice	*F. asiaticum*	NIV	28.93416203	113.242577	58.17	Farmer owned field
170902	2017	Hunan	Rice	*F. asiaticum*	NIV	28.93416203	113.242577	58.17	Farmer owned field
170901	2017	Hunan	Rice	*F. asiaticum*	NIV	28.93416203	113.242577	58.17	Farmer owned field
170900	2017	Hunan	Rice	*F. asiaticum*	15ADON	28.93416203	113.242577	58.17	Farmer owned field
170899	2017	Hunan	Rice	*F. asiaticum*	NIV	28.93416203	113.242577	58.17	Farmer owned field
170898	2017	Hunan	Rice	*F. asiaticum*	NIV	28.93416203	113.242577	58.17	Farmer owned field
170897	2017	Hunan	Rice	*F. asiaticum*	NIV	28.93416203	113.242577	58.17	Farmer owned field
170896	2017	Hunan	Rice	*F. asiaticum*	NIV	28.93416203	113.242577	58.17	Farmer owned field
170895	2017	Hunan	Rice	*F. asiaticum*	NIV	28.93416203	113.242577	58.17	Farmer owned field
170894	2017	Hunan	Rice	*F. asiaticum*	NIV	28.93416203	113.242577	58.17	Farmer owned field
170893	2017	Hunan	Rice	*F. asiaticum*	NIV	28.93416203	113.242577	58.17	Farmer owned field
170892	2017	Hunan	Rice	*F. asiaticum*	3ADON	28.93416203	113.242577	58.17	Farmer owned field
170891	2017	Hunan	Rice	*F. asiaticum*	NIV	28.93416203	113.242577	58.17	Farmer owned field
170890	2017	Hunan	Rice	*F. asiaticum*	3ADON	28.93416203	113.242577	58.17	Farmer owned field
170889	2017	Hunan	Rice	*F. asiaticum*	NIV	28.93416203	113.242577	58.17	Farmer owned field
170888	2017	Hunan	Rice	*F. asiaticum*	NIV	28.93416203	113.242577	58.17	Farmer owned field

170887	2017	Hunan	Rice	*F. asiaticum*	3ADON	28.93416203	113.242577	58.17	Farmer owned field
170886	2017	Hunan	Rice	*F. asiaticum*	NIV	28.93416203	113.242577	58.17	Farmer owned field
170885	2017	Hunan	Rice	*F. asiaticum*	NIV	28.93416203	113.242577	58.17	Farmer owned field
170884	2017	Hunan	Rice	*F. asiaticum*	NIV	28.93416203	113.242577	58.17	Farmer owned field
170883	2017	Hunan	Rice	*F. asiaticum*	NIV	28.93416203	113.242577	58.17	Farmer owned field
170882	2017	Hunan	Rice	*F. asiaticum*	NIV	28.93416203	113.242577	58.17	Farmer owned field
170881	2017	Hunan	Rice	*F. asiaticum*	3ADON	28.93416203	113.242577	58.17	Farmer owned field
170880	2017	Hunan	Rice	*F. asiaticum*	3ADON	28.93416203	113.242577	58.17	Farmer owned field
170879	2017	Hunan	Rice	*F. asiaticum*	NIV	28.93416203	113.242577	58.17	Farmer owned field
170878	2017	Hunan	Rice	*F. asiaticum*	NIV	28.93416203	113.242577	58.17	Farmer owned field
170877	2017	Hunan	Rice	*F. asiaticum*	NIV	28.93416203	113.242577	58.17	Farmer owned field
170876	2017	Hunan	Rice	*F. asiaticum*	3ADON	28.93416203	113.242577	58.17	Farmer owned field
170875	2017	Hunan	Rice	*F. asiaticum*	NIV	28.93416203	113.242577	58.17	Farmer owned field
170874	2017	Hunan	Rice	*F. asiaticum*	NIV	28.93416203	113.242577	58.17	Farmer owned field
170873	2017	Hunan	Rice	*F. asiaticum*	NIV	28.93416203	113.242577	58.17	Farmer owned field
170872	2017	Hunan	Rice	*F. asiaticum*	NIV	28.93416203	113.242577	58.17	Farmer owned field
170871	2017	Hunan	Rice	*F. asiaticum*	NIV	28.93416203	113.242577	58.17	Farmer owned field
170870	2017	Hunan	Rice	*F. asiaticum*	NIV	28.93416203	113.242577	58.17	Farmer owned field
170869	2017	Hunan	Rice	*F. asiaticum*	NIV	28.93416203	113.242577	58.17	Farmer owned field
170868	2017	Hunan	Rice	*F. asiaticum*	NIV	28.93416203	113.242577	58.17	Farmer owned field
170867	2017	Hunan	Rice	*F. asiaticum*	NIV	28.93416203	113.242577	58.17	Farmer owned field
170589	2017	Hunan	Rice	*F. asiaticum*	NIV	28.94113996	112.486655	28.18	Farmer owned field
170588	2017	Hunan	Rice	*F. asiaticum*	NIV	28.94113996	112.486655	28.18	Farmer owned field
170587	2017	Hunan	Rice	*F. asiaticum*	NIV	28.94113996	112.486655	28.18	Farmer owned field
170586	2017	Hunan	Rice	*F. asiaticum*	3ADON	28.94113996	112.486655	28.18	Farmer owned field
170585	2017	Hunan	Rice	*F. asiaticum*	NIV	28.94113996	112.486655	28.18	Farmer owned field
170584	2017	Hunan	Rice	*F. asiaticum*	3ADON	28.94113996	112.486655	28.18	Farmer owned field
170583	2017	Hunan	Rice	*F. asiaticum*	3ADON	28.94113996	112.486655	28.18	Farmer owned field
170582	2017	Hunan	Rice	*F. asiaticum*	3ADON	28.94113996	112.486655	28.18	Farmer owned field
170581	2017	Hunan	Rice	*F. asiaticum*	3ADON	28.94113996	112.486655	28.18	Farmer owned field
170580	2017	Hunan	Rice	*F. asiaticum*	3ADON	28.94113996	112.486655	28.18	Farmer owned field
170579	2017	Hunan	Rice	*F. asiaticum*	NIV	28.94113996	112.486655	28.18	Farmer owned field
170578	2017	Hunan	Rice	*F. asiaticum*	3ADON	28.94113996	112.486655	28.18	Farmer owned field
170577	2017	Hunan	Rice	*F. asiaticum*	NIV	28.94113996	112.486655	28.18	Farmer owned field
170574	2017	Hunan	Rice	*F. asiaticum*	3ADON	28.94113996	112.486655	28.18	Farmer owned field

170573	2017	Hunan	Rice	*F. asiaticum*	15ADON	28.94113996	112.486655	28.18		Farmer owned field
170572	2017	Hunan	Rice	*F. asiaticum*	NIV	28.94113996	112.486655	28.18		Farmer owned field
170571	2017	Hunan	Rice	*F. asiaticum*	NIV	28.94113996	112.486655	28.18		Farmer owned field
170570	2017	Hunan	Rice	*F. asiaticum*	3ADON	28.94113996	112.486655	28.18		Farmer owned field
170569	2017	Hunan	Rice	*F. asiaticum*	3ADON	28.94113996	112.486655	28.18		Farmer owned field
170568	2017	Hunan	Rice	*F. asiaticum*	NIV	28.94113996	112.486655	28.18		Farmer owned field
170567	2017	Hunan	Rice	*F. asiaticum*	3ADON	28.94113996	112.486655	28.18		Farmer owned field
170566	2017	Hunan	Rice	*F. asiaticum*	NIV	28.94113996	112.486655	28.18		Farmer owned field
170565	2017	Hunan	Rice	*F. asiaticum*	3ADON	28.94113996	112.486655	28.18		Farmer owned field
170564	2017	Hunan	Rice	*F. asiaticum*	3ADON	28.94113996	112.486655	28.18		Farmer owned field
170563	2017	Hunan	Rice	*F. asiaticum*	3ADON	28.94113996	112.486655	28.18		Farmer owned field
170562	2017	Hunan	Rice	*F. asiaticum*	NIV	28.94113996	112.486655	28.18		Farmer owned field
170561	2017	Hunan	Rice	*F. asiaticum*	NIV	28.94113996	112.486655	28.18		Farmer owned field
170560	2017	Hunan	Rice	*F. asiaticum*	3ADON	28.94113996	112.486655	28.18		Farmer owned field
170559	2017	Hunan	Rice	*F. asiaticum*	3ADON	28.94113996	112.486655	28.18		Farmer owned field
170558	2017	Hunan	Rice	*F. asiaticum*	3ADON	28.94113996	112.486655	28.18		Farmer owned field
170557	2017	Hunan	Rice	*F. asiaticum*	NIV	28.94113996	112.486655	28.18		Farmer owned field
170556	2017	Hunan	Rice	*F. asiaticum*	3ADON	28.94113996	112.486655	28.18		Farmer owned field
170555	2017	Hunan	Rice	*F. asiaticum*	3ADON	28.94113996	112.486655	28.18		Farmer owned field
170554	2017	Hunan	Rice	*F. asiaticum*	3ADON	28.94113996	112.486655	28.18		Farmer owned field
170553	2017	Hunan	Rice	*F. asiaticum*	NIV	28.94113996	112.486655	28.18		Farmer owned field
170552	2017	Hunan	Rice	*F. asiaticum*	NIV	28.94113996	112.486655	28.18		Farmer owned field
170551	2017	Hunan	Rice	*F. asiaticum*	NIV	28.94113996	112.486655	28.18		Farmer owned field
170550	2017	Hunan	Rice	*F. asiaticum*	3ADON	28.94113996	112.486655	28.18		Farmer owned field
170549	2017	Hunan	Rice	*F. asiaticum*	3ADON	28.94113996	112.486655	28.18		Farmer owned field
170547	2017	Hunan	Rice	*F. asiaticum*	3ADON	28.94113996	112.486655	28.18		Farmer owned field
170546	2017	Hunan	Rice	*F. asiaticum*	3ADON	28.94113996	112.486655	28.18		Farmer owned field
170545	2017	Hunan	Rice	*F. asiaticum*	NIV	28.94113996	112.486655	28.18		Farmer owned field
170544	2017	Hunan	Rice	*F. asiaticum*	3ADON	28.94113996	112.486655	28.18		Farmer owned field
170542	2017	Hunan	Rice	*F. asiaticum*	3ADON	28.94113996	112.486655	28.18		Farmer owned field
170502	2017	Hunan	Rice	*F. asiaticum*	NIV	28.97530401	112.363732	NA		Farmer owned field
170501	2017	Hunan	Rice	*F. asiaticum*	3ADON	28.97530401	112.363732	NA		Farmer owned field
170500	2017	Hunan	Rice	*F. asiaticum*	3ADON	28.97530401	112.363732	NA		Farmer owned field
170499	2017	Hunan	Rice	*F. asiaticum*	3ADON	28.97530401	112.363732	NA		Farmer owned field
170498	2017	Hunan	Rice	*F. asiaticum*	3ADON	28.97530401	112.363732	NA		Farmer owned field

170497	2017	Hunan	Rice	*F. asiaticum*	NIV	28.97530401	112.363732	NA		Farmer owned field
170496	2017	Hunan	Rice	*F. asiaticum*	NIV	28.97530401	112.363732	NA		Farmer owned field
170495	2017	Hunan	Rice	*F. asiaticum*	3ADON	28.97530401	112.363732	NA		Farmer owned field
170494	2017	Hunan	Rice	*F. asiaticum*	3ADON	28.97530401	112.363732	NA		Farmer owned field
170493	2017	Hunan	Rice	*F. asiaticum*	NIV	28.97530401	112.363732	NA		Farmer owned field
170492	2017	Hunan	Rice	*F. asiaticum*	15ADON	28.97530401	112.363732	NA		Farmer owned field
170491	2017	Hunan	Rice	*F. asiaticum*	NIV	28.97530401	112.363732	NA		Farmer owned field
170490	2017	Hunan	Rice	*F. asiaticum*	NIV	28.97530401	112.363732	NA		Farmer owned field
170489	2017	Hunan	Rice	*F. asiaticum*	NIV	28.97530401	112.363732	NA		Farmer owned field
170488	2017	Hunan	Rice	*F. asiaticum*	NIV	28.97530401	112.363732	NA		Farmer owned field
170487	2017	Hunan	Rice	*F. asiaticum*	3ADON	28.97530401	112.363732	NA		Farmer owned field
170486	2017	Hunan	Rice	*F. asiaticum*	NIV	28.97530401	112.363732	NA		Farmer owned field
170485	2017	Hunan	Rice	*F. asiaticum*	15ADON	28.97530401	112.363732	NA		Farmer owned field
170484	2017	Hunan	Rice	*F. asiaticum*	3ADON	28.97530401	112.363732	NA		Farmer owned field
170483	2017	Hunan	Rice	*F. asiaticum*	NIV	28.97530401	112.363732	NA		Farmer owned field
170481	2017	Hunan	Rice	*F. asiaticum*	NIV	28.97530401	112.363732	NA		Farmer owned field
170480	2017	Hunan	Rice	*F. asiaticum*	NIV	28.97530401	112.363732	NA		Farmer owned field
170478	2017	Hunan	Rice	*F. asiaticum*	3ADON	28.97530401	112.363732	NA		Farmer owned field
170477	2017	Hunan	Rice	*F. asiaticum*	3ADON	28.97530401	112.363732	NA		Farmer owned field
170476	2017	Hunan	Rice	*F. asiaticum*	3ADON	28.97530401	112.363732	NA		Farmer owned field
170475	2017	Hunan	Rice	*F. asiaticum*	NIV	28.97530401	112.363732	NA		Farmer owned field
170474	2017	Hunan	Rice	*F. asiaticum*	3ADON	28.97530401	112.363732	NA		Farmer owned field
170472	2017	Hunan	Rice	*F. asiaticum*	NIV	28.97530401	112.363732	NA		Farmer owned field
170471	2017	Hunan	Rice	*F. asiaticum*	3ADON	28.97530401	112.363732	NA		Farmer owned field
170469	2017	Hunan	Rice	*F. asiaticum*	3ADON	28.97530401	112.363732	NA		Farmer owned field
170467	2017	Hunan	Rice	*F. asiaticum*	NIV	28.97530401	112.363732	NA		Farmer owned field
170466	2017	Hunan	Rice	*F. asiaticum*	NIV	28.97530401	112.363732	NA		Farmer owned field
170465	2017	Hunan	Rice	*F. asiaticum*	NIV	28.97530401	112.363732	NA		Farmer owned field
170464	2017	Hunan	Rice	*F. asiaticum*	NIV	28.97530401	112.363732	NA		Farmer owned field
170462	2017	Hunan	Rice	*F. asiaticum*	3ADON	28.97530401	112.363732	NA		Farmer owned field
170461	2017	Hunan	Rice	*F. asiaticum*	3ADON	28.97530401	112.363732	NA		Farmer owned field
170460	2017	Hunan	Rice	*F. asiaticum*	3ADON	28.97530401	112.363732	NA		Farmer owned field
193878	2019	Hunan	Rice	*F. asiaticum*	3ADON	29.04777001	112.372014	33.342587		Farmer owned field
193877	2019	Hunan	Rice	*F. asiaticum*	3ADON	29.04777001	112.372014	33.342587		Farmer owned field
193876	2019	Hunan	Rice	*F. asiaticum*	3ADON	29.04777001	112.372014	33.342587		Farmer owned field

193875	2019	Hunan	Rice	*F. asiaticum*	3ADON	29.04777001	112.372014	33.342587		Farmer owned field
193874	2019	Hunan	Rice	*F. asiaticum*	NIV	29.04777001	112.372014	33.342587		Farmer owned field
193873	2019	Hunan	Rice	*F. asiaticum*	3ADON	29.04777001	112.372014	33.342587		Farmer owned field
193872	2019	Hunan	Rice	*F. asiaticum*	3ADON	29.04777001	112.372014	33.342587		Farmer owned field
193871	2019	Hunan	Rice	*F. asiaticum*	NIV	29.04777001	112.372014	33.342587		Farmer owned field
193870	2019	Hunan	Rice	*F. asiaticum*	NIV	29.04777001	112.372014	33.342587		Farmer owned field
193869	2019	Hunan	Rice	*F. asiaticum*	3ADON	29.04777001	112.372014	33.342587		Farmer owned field
193868	2019	Hunan	Rice	*F. asiaticum*	15ADON	29.04777001	112.372014	33.342587		Farmer owned field
193867	2019	Hunan	Rice	*F. asiaticum*	3ADON	29.04777001	112.372014	33.342587		Farmer owned field
193866	2019	Hunan	Rice	*F. asiaticum*	NIV	29.04777001	112.372014	33.342587		Farmer owned field
193865	2019	Hunan	Rice	*F. asiaticum*	NIV	29.04777001	112.372014	33.342587		Farmer owned field
193864	2019	Hunan	Rice	*F. asiaticum*	3ADON	29.04777001	112.372014	33.342587		Farmer owned field
193863	2019	Hunan	Rice	*F. asiaticum*	3ADON	29.04777001	112.372014	33.342587		Farmer owned field
193862	2019	Hunan	Rice	*F. asiaticum*	3ADON	29.04777001	112.372014	33.342587		Farmer owned field
193861	2019	Hunan	Rice	*F. asiaticum*	3ADON	29.04777001	112.372014	33.342587		Farmer owned field
193860	2019	Hunan	Rice	*F. asiaticum*	3ADON	29.04777001	112.372014	33.342587		Farmer owned field
193859	2019	Hunan	Rice	*F. asiaticum*	NIV	29.04777001	112.372014	33.342587		Farmer owned field
193858	2019	Hunan	Rice	*F. asiaticum*	3ADON	29.04777001	112.372014	33.342587		Farmer owned field
193857	2019	Hunan	Rice	*F. asiaticum*	NIV	29.04777001	112.372014	33.342587		Farmer owned field
193856	2019	Hunan	Rice	*F. asiaticum*	3ADON	29.04777001	112.372014	33.342587		Farmer owned field
193855	2019	Hunan	Rice	*F. asiaticum*	3ADON	29.04777001	112.372014	33.342587		Farmer owned field
193854	2019	Hunan	Rice	*F. asiaticum*	NIV	29.04777001	112.372014	33.342587		Farmer owned field
193852	2019	Hunan	Rice	*F. asiaticum*	15ADON	29.04777001	112.372014	33.342587		Farmer owned field
170629	2017	Hunan	Rice	*F. asiaticum*	15ADON	29.04777001	112.372014	32		Farmer owned field
170628	2017	Hunan	Rice	*F. asiaticum*	3ADON	29.04777001	112.372014	32		Farmer owned field
170627	2017	Hunan	Rice	*F. asiaticum*	3ADON	29.04777001	112.372014	32		Farmer owned field
170626	2017	Hunan	Rice	*F. asiaticum*	3ADON	29.04777001	112.372014	32		Farmer owned field
170625	2017	Hunan	Rice	*F. asiaticum*	3ADON	29.04777001	112.372014	32		Farmer owned field
170624	2017	Hunan	Rice	*F. asiaticum*	NIV	29.04777001	112.372014	32		Farmer owned field
170623	2017	Hunan	Rice	*F. asiaticum*	15ADON	29.04777001	112.372014	32		Farmer owned field
170622	2017	Hunan	Rice	*F. asiaticum*	3ADON	29.04777001	112.372014	32		Farmer owned field
170621	2017	Hunan	Rice	*F. asiaticum*	NIV	29.04777001	112.372014	32		Farmer owned field
170620	2017	Hunan	Rice	*F. asiaticum*	3ADON	29.04777001	112.372014	32		Farmer owned field
170619	2017	Hunan	Rice	*F. asiaticum*	3ADON	29.04777001	112.372014	32		Farmer owned field
170618	2017	Hunan	Rice	*F. asiaticum*	3ADON	29.04777001	112.372014	32		Farmer owned field

170617	2017	Hunan	Rice	*F. asiaticum*	3ADON	29.04777001	112.372014	32	Farmer owned field
170616	2017	Hunan	Rice	*F. asiaticum*	3ADON	29.04777001	112.372014	32	Farmer owned field
170615	2017	Hunan	Rice	*F. asiaticum*	3ADON	29.04777001	112.372014	32	Farmer owned field
170614	2017	Hunan	Rice	*F. asiaticum*	3ADON	29.04777001	112.372014	32	Farmer owned field
170613	2017	Hunan	Rice	*F. asiaticum*	3ADON	29.04777001	112.372014	32	Farmer owned field
170612	2017	Hunan	Rice	*F. asiaticum*	3ADON	29.04777001	112.372014	32	Farmer owned field
170611	2017	Hunan	Rice	*F. asiaticum*	3ADON	29.04777001	112.372014	32	Farmer owned field
170610	2017	Hunan	Rice	*F. asiaticum*	3ADON	29.04777001	112.372014	32	Farmer owned field
170609	2017	Hunan	Rice	*F. asiaticum*	3ADON	29.04777001	112.372014	32	Farmer owned field
170608	2017	Hunan	Rice	*F. asiaticum*	3ADON	29.04777001	112.372014	32	Farmer owned field
170607	2017	Hunan	Rice	*F. asiaticum*	3ADON	29.04777001	112.372014	32	Farmer owned field
170606	2017	Hunan	Rice	*F. asiaticum*	3ADON	29.04777001	112.372014	32	Farmer owned field
170605	2017	Hunan	Rice	*F. asiaticum*	NIV	29.04777001	112.372014	32	Farmer owned field
170604	2017	Hunan	Rice	*F. asiaticum*	NIV	29.04777001	112.372014	32	Farmer owned field
170603	2017	Hunan	Rice	*F. asiaticum*	3ADON	29.04777001	112.372014	32	Farmer owned field
170602	2017	Hunan	Rice	*F. asiaticum*	3ADON	29.04777001	112.372014	32	Farmer owned field
170601	2017	Hunan	Rice	*F. asiaticum*	NIV	29.04777001	112.372014	32	Farmer owned field
170600	2017	Hunan	Rice	*F. asiaticum*	3ADON	29.04777001	112.372014	32	Farmer owned field
170599	2017	Hunan	Rice	*F. asiaticum*	3ADON	29.04777001	112.372014	32	Farmer owned field
170598	2017	Hunan	Rice	*F. asiaticum*	3ADON	29.04777001	112.372014	32	Farmer owned field
170597	2017	Hunan	Rice	*F. asiaticum*	3ADON	29.04777001	112.372014	32	Farmer owned field
170596	2017	Hunan	Rice	*F. asiaticum*	NIV	29.04777001	112.372014	32	Farmer owned field
170594	2017	Hunan	Rice	*F. asiaticum*	NIV	29.04777001	112.372014	32	Farmer owned field
170593	2017	Hunan	Rice	*F. asiaticum*	3ADON	29.04777001	112.372014	32	Farmer owned field
170592	2017	Hunan	Rice	*F. asiaticum*	15ADON	29.04777001	112.372014	32	Farmer owned field
170591	2017	Hunan	Rice	*F. asiaticum*	NIV	29.04777001	112.372014	32	Farmer owned field
170590	2017	Hunan	Rice	*F. asiaticum*	3ADON	29.04777001	112.372014	32	Farmer owned field
170856	2017	Hunan	Rice	*F. asiaticum*	3ADON	29.14365001	113.237246	37.35	Farmer owned field
170855	2017	Hunan	Rice	*F. asiaticum*	NIV	29.14365001	113.237246	37.35	Farmer owned field
170854	2017	Hunan	Rice	*F. asiaticum*	NIV	29.14365001	113.237246	37.35	Farmer owned field
170853	2017	Hunan	Rice	*F. asiaticum*	NIV	29.14365001	113.237246	37.35	Farmer owned field
170852	2017	Hunan	Rice	*F. asiaticum*	15ADON	29.14365001	113.237246	37.35	Farmer owned field
170851	2017	Hunan	Rice	*F. asiaticum*	3ADON	29.14365001	113.237246	37.35	Farmer owned field
170850	2017	Hunan	Rice	*F. asiaticum*	NIV	29.14365001	113.237246	37.35	Farmer owned field
170849	2017	Hunan	Rice	*F. asiaticum*	NIV	29.14365001	113.237246	37.35	Farmer owned field

170847	2017	Hunan	Rice	*F. asiaticum*	3ADON	29.14365001	113.237246	37.35	Farmer owned field
170846	2017	Hunan	Rice	*F. asiaticum*	NIV	29.14365001	113.237246	37.35	Farmer owned field
170845	2017	Hunan	Rice	*F. asiaticum*	NIV	29.14365001	113.237246	37.35	Farmer owned field
170844	2017	Hunan	Rice	*F. asiaticum*	3ADON	29.14365001	113.237246	37.35	Farmer owned field
170843	2017	Hunan	Rice	*F. asiaticum*	3ADON	29.14365001	113.237246	37.35	Farmer owned field
170842	2017	Hunan	Rice	*F. asiaticum*	NIV	29.14365001	113.237246	37.35	Farmer owned field
170841	2017	Hunan	Rice	*F. asiaticum*	3ADON	29.14365001	113.237246	37.35	Farmer owned field
170840	2017	Hunan	Rice	*F. asiaticum*	NIV	29.14365001	113.237246	37.35	Farmer owned field
170839	2017	Hunan	Rice	*F. asiaticum*	3ADON	29.14365001	113.237246	37.35	Farmer owned field
170838	2017	Hunan	Rice	*F. asiaticum*	3ADON	29.14365001	113.237246	37.35	Farmer owned field
170836	2017	Hunan	Rice	*F. asiaticum*	3ADON	29.14365001	113.237246	37.35	Farmer owned field
170835	2017	Hunan	Rice	*F. asiaticum*	NIV	29.14365001	113.237246	37.35	Farmer owned field
170833	2017	Hunan	Rice	*F. asiaticum*	15ADON	29.14365001	113.237246	37.35	Farmer owned field
170832	2017	Hunan	Rice	*F. asiaticum*	NIV	29.14365001	113.237246	37.35	Farmer owned field
170831	2017	Hunan	Rice	*F. asiaticum*	NIV	29.14365001	113.237246	37.35	Farmer owned field
170830	2017	Hunan	Rice	*F. asiaticum*	3ADON	29.14365001	113.237246	37.35	Farmer owned field
170829	2017	Hunan	Rice	*F. asiaticum*	3ADON	29.14365001	113.237246	37.35	Farmer owned field
170828	2017	Hunan	Rice	*F. asiaticum*	3ADON	29.14365001	113.237246	37.35	Farmer owned field
170827	2017	Hunan	Rice	*F. asiaticum*	3ADON	29.14365001	113.237246	37.35	Farmer owned field
170826	2017	Hunan	Rice	*F. asiaticum*	NIV	29.14365001	113.237246	37.35	Farmer owned field
170824	2017	Hunan	Rice	*F. asiaticum*	NIV	29.14365001	113.237246	37.35	Farmer owned field
193932	2019	Hunan	Rice	*F. asiaticum*	3ADON	29.19206598	112.320635	33.244308	Farmer owned field
193931	2019	Hunan	Rice	*F. asiaticum*	3ADON	29.19206598	112.320635	33.244308	Farmer owned field
193930	2019	Hunan	Rice	*F. asiaticum*	3ADON	29.19206598	112.320635	33.244308	Farmer owned field
193928	2019	Hunan	Rice	*F. asiaticum*	15ADON	29.19206598	112.320635	33.244308	Farmer owned field
193927	2019	Hunan	Rice	*F. asiaticum*	3ADON	29.19206598	112.320635	33.244308	Farmer owned field
193926	2019	Hunan	Rice	*F. asiaticum*	NIV	29.19206598	112.320635	33.244308	Farmer owned field
193925	2019	Hunan	Rice	*F. asiaticum*	3ADON	29.19206598	112.320635	33.244308	Farmer owned field
193924	2019	Hunan	Rice	*F. asiaticum*	3ADON	29.19206598	112.320635	33.244308	Farmer owned field
193923	2019	Hunan	Rice	*F. asiaticum*	3ADON	29.19206598	112.320635	33.244308	Farmer owned field
193922	2019	Hunan	Rice	*F. asiaticum*	15ADON	29.19206598	112.320635	33.244308	Farmer owned field
193921	2019	Hunan	Rice	*F. asiaticum*	3ADON	29.19206598	112.320635	33.244308	Farmer owned field
193920	2019	Hunan	Rice	*F. asiaticum*	3ADON	29.19206598	112.320635	33.244308	Farmer owned field
193919	2019	Hunan	Rice	*F. asiaticum*	3ADON	29.19206598	112.320635	33.244308	Farmer owned field
193917	2019	Hunan	Rice	*F. asiaticum*	NIV	29.19206598	112.320635	33.244308	Farmer owned field

193916	2019	Hunan	Rice	*F. asiaticum*	3ADON	29.19206598	112.320635	33.244308		Farmer owned field
193915	2019	Hunan	Rice	*F. asiaticum*	NIV	29.19206598	112.320635	33.244308		Farmer owned field
193914	2019	Hunan	Rice	*F. asiaticum*	3ADON	29.19206598	112.320635	33.244308		Farmer owned field
193913	2019	Hunan	Rice	*F. asiaticum*	3ADON	29.19206598	112.320635	33.244308		Farmer owned field
193912	2019	Hunan	Rice	*F. asiaticum*	3ADON	29.19206598	112.320635	33.244308		Farmer owned field
193911	2019	Hunan	Rice	*F. asiaticum*	NIV	29.19206598	112.320635	33.244308		Farmer owned field
193910	2019	Hunan	Rice	*F. asiaticum*	NIV	29.19206598	112.320635	33.244308		Farmer owned field
193909	2019	Hunan	Rice	*F. asiaticum*	NIV	29.19206598	112.320635	33.244308		Farmer owned field
193908	2019	Hunan	Rice	*F. asiaticum*	3ADON	29.19206598	112.320635	33.244308		Farmer owned field
193907	2019	Hunan	Rice	*F. asiaticum*	3ADON	29.19206598	112.320635	33.244308		Farmer owned field
193906	2019	Hunan	Rice	*F. asiaticum*	3ADON	29.19206598	112.320635	33.244308		Farmer owned field
193905	2019	Hunan	Rice	*F. asiaticum*	NIV	29.19206598	112.320635	33.244308		Farmer owned field
170664	2017	Hunan	Rice	*F. asiaticum*	15ADON	29.19206598	112.320635	28		Farmer owned field
170663	2017	Hunan	Rice	*F. asiaticum*	NIV	29.19206598	112.320635	28		Farmer owned field
170662	2017	Hunan	Rice	*F. asiaticum*	15ADON	29.19206598	112.320635	28		Farmer owned field
170661	2017	Hunan	Rice	*F. asiaticum*	3ADON	29.19206598	112.320635	28		Farmer owned field
170659	2017	Hunan	Rice	*F. asiaticum*	15ADON	29.19206598	112.320635	28		Farmer owned field
170658	2017	Hunan	Rice	*F. asiaticum*	NIV	29.19206598	112.320635	28		Farmer owned field
170657	2017	Hunan	Rice	*F. asiaticum*	15ADON	29.19206598	112.320635	28		Farmer owned field
170656	2017	Hunan	Rice	*F. asiaticum*	3ADON	29.19206598	112.320635	28		Farmer owned field
170655	2017	Hunan	Rice	*F. asiaticum*	3ADON	29.19206598	112.320635	28		Farmer owned field
170654	2017	Hunan	Rice	*F. asiaticum*	NIV	29.19206598	112.320635	28		Farmer owned field
170653	2017	Hunan	Rice	*F. asiaticum*	3ADON	29.19206598	112.320635	28		Farmer owned field
170652	2017	Hunan	Rice	*F. asiaticum*	3ADON	29.19206598	112.320635	28		Farmer owned field
170651	2017	Hunan	Rice	*F. asiaticum*	3ADON	29.19206598	112.320635	28		Farmer owned field
170650	2017	Hunan	Rice	*F. asiaticum*	3ADON	29.19206598	112.320635	28		Farmer owned field
170649	2017	Hunan	Rice	*F. asiaticum*	NIV	29.19206598	112.320635	28		Farmer owned field
170648	2017	Hunan	Rice	*F. asiaticum*	3ADON	29.19206598	112.320635	28		Farmer owned field
170647	2017	Hunan	Rice	*F. asiaticum*	NIV	29.19206598	112.320635	28		Farmer owned field
170646	2017	Hunan	Rice	*F. asiaticum*	3ADON	29.19206598	112.320635	28		Farmer owned field
170645	2017	Hunan	Rice	*F. asiaticum*	15ADON	29.19206598	112.320635	28		Farmer owned field
170644	2017	Hunan	Rice	*F. asiaticum*	15ADON	29.19206598	112.320635	28		Farmer owned field
170643	2017	Hunan	Rice	*F. asiaticum*	3ADON	29.19206598	112.320635	28		Farmer owned field
170642	2017	Hunan	Rice	*F. asiaticum*	15ADON	29.19206598	112.320635	28		Farmer owned field
170641	2017	Hunan	Rice	*F. asiaticum*	3ADON	29.19206598	112.320635	28		Farmer owned field

170640	2017	Hunan	Rice	*F. asiaticum*	15ADON	29.19206598	112.320635	28		Farmer owned field
170639	2017	Hunan	Rice	*F. asiaticum*	NIV	29.19206598	112.320635	28		Farmer owned field
170638	2017	Hunan	Rice	*F. asiaticum*	15ADON	29.19206598	112.320635	28		Farmer owned field
170637	2017	Hunan	Rice	*F. asiaticum*	15ADON	29.19206598	112.320635	28		Farmer owned field
170636	2017	Hunan	Rice	*F. asiaticum*	NIV	29.19206598	112.320635	28		Farmer owned field
170635	2017	Hunan	Rice	*F. asiaticum*	NIV	29.19206598	112.320635	28		Farmer owned field
170634	2017	Hunan	Rice	*F. asiaticum*	NIV	29.19206598	112.320635	28		Farmer owned field
170633	2017	Hunan	Rice	*F. asiaticum*	15ADON	29.19206598	112.320635	28		Farmer owned field
170632	2017	Hunan	Rice	*F. asiaticum*	3ADON	29.19206598	112.320635	28		Farmer owned field
170631	2017	Hunan	Rice	*F. asiaticum*	NIV	29.19206598	112.320635	28		Farmer owned field
170630	2017	Hunan	Rice	*F. asiaticum*	3ADON	29.19206598	112.320635	28		Farmer owned field
193385	2019	Hunan	Rice	*F. asiaticum*	15ADON	29.29586504	113.17728	NA		Farmer owned field
193384	2019	Hunan	Rice	*F. asiaticum*	3ADON	29.29586504	113.17728	NA		Farmer owned field
193383	2019	Hunan	Rice	*F. asiaticum*	3ADON	29.29586504	113.17728	NA		Farmer owned field
193382	2019	Hunan	Rice	*F. asiaticum*	NIV	29.29586504	113.17728	NA		Farmer owned field
193381	2019	Hunan	Rice	*F. asiaticum*	3ADON	29.29586504	113.17728	NA		Farmer owned field
193380	2019	Hunan	Rice	*F. asiaticum*	NIV	29.29586504	113.17728	NA		Farmer owned field
193379	2019	Hunan	Rice	*F. asiaticum*	NIV	29.29586504	113.17728	NA		Farmer owned field
193378	2019	Hunan	Rice	*F. asiaticum*	NIV	29.29586504	113.17728	NA		Farmer owned field
193377	2019	Hunan	Rice	*F. asiaticum*	15ADON	29.29586504	113.17728	NA		Farmer owned field
193375	2019	Hunan	Rice	*F. asiaticum*	NIV	29.29586504	113.17728	NA		Farmer owned field
193374	2019	Hunan	Rice	*F. asiaticum*	NIV	29.29586504	113.17728	NA		Farmer owned field
193373	2019	Hunan	Rice	*F. asiaticum*	NIV	29.29586504	113.17728	NA		Farmer owned field
193372	2019	Hunan	Rice	*F. asiaticum*	15ADON	29.29586504	113.17728	NA		Farmer owned field
193370	2019	Hunan	Rice	*F. asiaticum*	NIV	29.29586504	113.17728	NA		Farmer owned field
193368	2019	Hunan	Rice	*F. asiaticum*	3ADON	29.29586504	113.17728	NA		Farmer owned field
193366	2019	Hunan	Rice	*F. asiaticum*	NIV	29.29586504	113.17728	NA		Farmer owned field
193365	2019	Hunan	Rice	*F. asiaticum*	3ADON	29.29586504	113.17728	NA		Farmer owned field
193364	2019	Hunan	Rice	*F. asiaticum*	NIV	29.29586504	113.17728	NA		Farmer owned field
193363	2019	Hunan	Rice	*F. asiaticum*	NIV	29.29586504	113.17728	NA		Farmer owned field
193362	2019	Hunan	Rice	*F. asiaticum*	NIV	29.29586504	113.17728	NA		Farmer owned field
193360	2019	Hunan	Rice	*F. asiaticum*	NIV	29.29586504	113.17728	NA		Farmer owned field
193359	2019	Hunan	Rice	*F. asiaticum*	3ADON	29.29586504	113.17728	NA		Farmer owned field
193357	2019	Hunan	Rice	*F. asiaticum*	15ADON	29.29586504	113.17728	NA		Farmer owned field
193356	2019	Hunan	Rice	*F. asiaticum*	NIV	29.29586504	113.17728	NA		Farmer owned field

193355	2019	Hunan	Rice	*F. asiaticum*	3ADON	29.29586504	113.17728	NA	Farmer owned field
193354	2019	Hunan	Rice	*F. asiaticum*	3ADON	29.29586504	113.17728	NA	Farmer owned field
170823	2017	Hunan	Rice	*F. asiaticum*	3ADON	29.31476397	113.179366	35.34	Farmer owned field
170822	2017	Hunan	Rice	*F. asiaticum*	3ADON	29.31476397	113.179366	35.34	Farmer owned field
170821	2017	Hunan	Rice	*F. asiaticum*	3ADON	29.31476397	113.179366	35.34	Farmer owned field
170820	2017	Hunan	Rice	*F. asiaticum*	3ADON	29.31476397	113.179366	35.34	Farmer owned field
170819	2017	Hunan	Rice	*F. asiaticum*	3ADON	29.31476397	113.179366	35.34	Farmer owned field
170818	2017	Hunan	Rice	*F. asiaticum*	NIV	29.31476397	113.179366	35.34	Farmer owned field
170817	2017	Hunan	Rice	*F. asiaticum*	NIV	29.31476397	113.179366	35.34	Farmer owned field
170816	2017	Hunan	Rice	*F. asiaticum*	NIV	29.31476397	113.179366	35.34	Farmer owned field
170815	2017	Hunan	Rice	*F. asiaticum*	3ADON	29.31476397	113.179366	35.34	Farmer owned field
170814	2017	Hunan	Rice	*F. asiaticum*	NIV	29.31476397	113.179366	35.34	Farmer owned field
170813	2017	Hunan	Rice	*F. asiaticum*	3ADON	29.31476397	113.179366	35.34	Farmer owned field
170812	2017	Hunan	Rice	*F. asiaticum*	NIV	29.31476397	113.179366	35.34	Farmer owned field
170811	2017	Hunan	Rice	*F. asiaticum*	NIV	29.31476397	113.179366	35.34	Farmer owned field
170810	2017	Hunan	Rice	*F. asiaticum*	3ADON	29.31476397	113.179366	35.34	Farmer owned field
170809	2017	Hunan	Rice	*F. asiaticum*	NIV	29.31476397	113.179366	35.34	Farmer owned field
170806	2017	Hunan	Rice	*F. asiaticum*	3ADON	29.31476397	113.179366	35.34	Farmer owned field
170805	2017	Hunan	Rice	*F. asiaticum*	3ADON	29.31476397	113.179366	35.34	Farmer owned field
170804	2017	Hunan	Rice	*F. asiaticum*	3ADON	29.31476397	113.179366	35.34	Farmer owned field
170803	2017	Hunan	Rice	*F. asiaticum*	NIV	29.31476397	113.179366	35.34	Farmer owned field
170801	2017	Hunan	Rice	*F. asiaticum*	NIV	29.31476397	113.179366	35.34	Farmer owned field
170800	2017	Hunan	Rice	*F. asiaticum*	3ADON	29.31476397	113.179366	35.34	Farmer owned field
170799	2017	Hunan	Rice	*F. asiaticum*	15ADON	29.31476397	113.179366	35.34	Farmer owned field
170798	2017	Hunan	Rice	*F. asiaticum*	3ADON	29.31476397	113.179366	35.34	Farmer owned field
170797	2017	Hunan	Rice	*F. asiaticum*	3ADON	29.31476397	113.179366	35.34	Farmer owned field
170796	2017	Hunan	Rice	*F. asiaticum*	NIV	29.31476397	113.179366	35.34	Farmer owned field
170795	2017	Hunan	Rice	*F. asiaticum*	NIV	29.31476397	113.179366	35.34	Farmer owned field
170793	2017	Hunan	Rice	*F. asiaticum*	15ADON	29.31476397	113.179366	35.34	Farmer owned field
170792	2017	Hunan	Rice	*F. asiaticum*	3ADON	29.31476397	113.179366	35.34	Farmer owned field
170790	2017	Hunan	Rice	*F. asiaticum*	NIV	29.31476397	113.179366	35.34	Farmer owned field
170789	2017	Hunan	Rice	*F. asiaticum*	NIV	29.31476397	113.179366	35.34	Farmer owned field
193904	2019	Hunan	Rice	*F. asiaticum*	3ADON	29.34845896	112.384461	35.926159	Farmer owned field
193903	2019	Hunan	Rice	*F. asiaticum*	NIV	29.34845896	112.384461	35.926159	Farmer owned field
193902	2019	Hunan	Rice	*F. asiaticum*	NIV	29.34845896	112.384461	35.926159	Farmer owned field

193901	2019	Hunan	Rice	*F. asiaticum*	3ADON	29.34845896	112.384461	35.926159	Farmer owned field
193900	2019	Hunan	Rice	*F. asiaticum*	3ADON	29.34845896	112.384461	35.926159	Farmer owned field
193899	2019	Hunan	Rice	*F. asiaticum*	NIV	29.34845896	112.384461	35.926159	Farmer owned field
193898	2019	Hunan	Rice	*F. asiaticum*	3ADON	29.34845896	112.384461	35.926159	Farmer owned field
193897	2019	Hunan	Rice	*F. asiaticum*	3ADON	29.34845896	112.384461	35.926159	Farmer owned field
193896	2019	Hunan	Rice	*F. asiaticum*	3ADON	29.34845896	112.384461	35.926159	Farmer owned field
193895	2019	Hunan	Rice	*F. asiaticum*	3ADON	29.34845896	112.384461	35.926159	Farmer owned field
193894	2019	Hunan	Rice	*F. asiaticum*	3ADON	29.34845896	112.384461	35.926159	Farmer owned field
193891	2019	Hunan	Rice	*F. asiaticum*	NIV	29.34845896	112.384461	35.926159	Farmer owned field
193890	2019	Hunan	Rice	*F. asiaticum*	NIV	29.34845896	112.384461	35.926159	Farmer owned field
193889	2019	Hunan	Rice	*F. asiaticum*	NIV	29.34845896	112.384461	35.926159	Farmer owned field
193888	2019	Hunan	Rice	*F. asiaticum*	3ADON	29.34845896	112.384461	35.926159	Farmer owned field
193887	2019	Hunan	Rice	*F. asiaticum*	NIV	29.34845896	112.384461	35.926159	Farmer owned field
193886	2019	Hunan	Rice	*F. asiaticum*	NIV	29.34845896	112.384461	35.926159	Farmer owned field
193884	2019	Hunan	Rice	*F. asiaticum*	3ADON	29.34845896	112.384461	35.926159	Farmer owned field
193883	2019	Hunan	Rice	*F. asiaticum*	3ADON	29.34845896	112.384461	35.926159	Farmer owned field
193882	2019	Hunan	Rice	*F. asiaticum*	NIV	29.34845896	112.384461	35.926159	Farmer owned field
193881	2019	Hunan	Rice	*F. asiaticum*	3ADON	29.34845896	112.384461	35.926159	Farmer owned field
193880	2019	Hunan	Rice	*F. asiaticum*	NIV	29.34845896	112.384461	35.926159	Farmer owned field
170704	2017	Hunan	Rice	*F. asiaticum*	NIV	29.34845896	112.384461	30	Farmer owned field
170703	2017	Hunan	Rice	*F. asiaticum*	15ADON	29.34845896	112.384461	30	Farmer owned field
170702	2017	Hunan	Rice	*F. asiaticum*	NIV	29.34845896	112.384461	30	Farmer owned field
170701	2017	Hunan	Rice	*F. asiaticum*	3ADON	29.34845896	112.384461	30	Farmer owned field
170700	2017	Hunan	Rice	*F. asiaticum*	3ADON	29.34845896	112.384461	30	Farmer owned field
170699	2017	Hunan	Rice	*F. asiaticum*	3ADON	29.34845896	112.384461	30	Farmer owned field
170698	2017	Hunan	Rice	*F. asiaticum*	3ADON	29.34845896	112.384461	30	Farmer owned field
170697	2017	Hunan	Rice	*F. asiaticum*	NIV	29.34845896	112.384461	30	Farmer owned field
170696	2017	Hunan	Rice	*F. asiaticum*	15ADON	29.34845896	112.384461	30	Farmer owned field
170695	2017	Hunan	Rice	*F. asiaticum*	3ADON	29.34845896	112.384461	30	Farmer owned field
170694	2017	Hunan	Rice	*F. asiaticum*	3ADON	29.34845896	112.384461	30	Farmer owned field
170693	2017	Hunan	Rice	*F. asiaticum*	3ADON	29.34845896	112.384461	30	Farmer owned field
170692	2017	Hunan	Rice	*F. asiaticum*	15ADON	29.34845896	112.384461	30	Farmer owned field
170691	2017	Hunan	Rice	*F. asiaticum*	NIV	29.34845896	112.384461	30	Farmer owned field
170690	2017	Hunan	Rice	*F. asiaticum*	3ADON	29.34845896	112.384461	30	Farmer owned field
170689	2017	Hunan	Rice	*F. asiaticum*	3ADON	29.34845896	112.384461	30	Farmer owned field

170688	2017	Hunan	Rice	*F. asiaticum*	3ADON	29.34845896	112.384461	30		Farmer owned field
170687	2017	Hunan	Rice	*F. asiaticum*	NIV	29.34845896	112.384461	30		Farmer owned field
170686	2017	Hunan	Rice	*F. asiaticum*	3ADON	29.34845896	112.384461	30		Farmer owned field
170685	2017	Hunan	Rice	*F. asiaticum*	3ADON	29.34845896	112.384461	30		Farmer owned field
170683	2017	Hunan	Rice	*F. asiaticum*	NIV	29.34845896	112.384461	30		Farmer owned field
170682	2017	Hunan	Rice	*F. asiaticum*	15ADON	29.34845896	112.384461	30		Farmer owned field
170681	2017	Hunan	Rice	*F. asiaticum*	3ADON	29.34845896	112.384461	30		Farmer owned field
170680	2017	Hunan	Rice	*F. asiaticum*	3ADON	29.34845896	112.384461	30		Farmer owned field
170679	2017	Hunan	Rice	*F. asiaticum*	3ADON	29.34845896	112.384461	30		Farmer owned field
170678	2017	Hunan	Rice	*F. asiaticum*	3ADON	29.34845896	112.384461	30		Farmer owned field
170677	2017	Hunan	Rice	*F. asiaticum*	NIV	29.34845896	112.384461	30		Farmer owned field
170676	2017	Hunan	Rice	*F. asiaticum*	3ADON	29.34845896	112.384461	30		Farmer owned field
170675	2017	Hunan	Rice	*F. asiaticum*	3ADON	29.34845896	112.384461	30		Farmer owned field
170674	2017	Hunan	Rice	*F. asiaticum*	3ADON	29.34845896	112.384461	30		Farmer owned field
170673	2017	Hunan	Rice	*F. asiaticum*	3ADON	29.34845896	112.384461	30		Farmer owned field
170672	2017	Hunan	Rice	*F. asiaticum*	3ADON	29.34845896	112.384461	30		Farmer owned field
170671	2017	Hunan	Rice	*F. asiaticum*	3ADON	29.34845896	112.384461	30		Farmer owned field
170670	2017	Hunan	Rice	*F. asiaticum*	3ADON	29.34845896	112.384461	30		Farmer owned field
170669	2017	Hunan	Rice	*F. asiaticum*	3ADON	29.34845896	112.384461	30		Farmer owned field
170668	2017	Hunan	Rice	*F. asiaticum*	3ADON	29.34845896	112.384461	30		Farmer owned field
170667	2017	Hunan	Rice	*F. asiaticum*	3ADON	29.34845896	112.384461	30		Farmer owned field
170666	2017	Hunan	Rice	*F. asiaticum*	3ADON	29.34845896	112.384461	30		Farmer owned field
170665	2017	Hunan	Rice	*F. asiaticum*	3ADON	29.34845896	112.384461	30		Farmer owned field
170724	2017	Hunan	Rice	*F. asiaticum*	NIV	29.41471397	112.476406	28.48		Farmer owned field
170723	2017	Hunan	Rice	*F. asiaticum*	3ADON	29.41471397	112.476406	28.48		Farmer owned field
170722	2017	Hunan	Rice	*F. asiaticum*	NIV	29.41471397	112.476406	28.48		Farmer owned field
170721	2017	Hunan	Rice	*F. asiaticum*	15ADON	29.41471397	112.476406	28.48		Farmer owned field
170720	2017	Hunan	Rice	*F. asiaticum*	15ADON	29.41471397	112.476406	28.48		Farmer owned field
170719	2017	Hunan	Rice	*F. asiaticum*	3ADON	29.41471397	112.476406	28.48		Farmer owned field
170718	2017	Hunan	Rice	*F. asiaticum*	3ADON	29.41471397	112.476406	28.48		Farmer owned field
170717	2017	Hunan	Rice	*F. asiaticum*	NIV	29.41471397	112.476406	28.48		Farmer owned field
170716	2017	Hunan	Rice	*F. asiaticum*	NIV	29.41471397	112.476406	28.48		Farmer owned field
170714	2017	Hunan	Rice	*F. asiaticum*	15ADON	29.41471397	112.476406	28.48		Farmer owned field
170713	2017	Hunan	Rice	*F. asiaticum*	3ADON	29.41471397	112.476406	28.48		Farmer owned field
170712	2017	Hunan	Rice	*F. asiaticum*	3ADON	29.41471397	112.476406	28.48		Farmer owned field

170711	2017	Hunan	Rice	*F. asiaticum*	3ADON	29.41471397	112.476406	28.48		Farmer owned field
170710	2017	Hunan	Rice	*F. asiaticum*	15ADON	29.41471397	112.476406	28.48		Farmer owned field
170709	2017	Hunan	Rice	*F. asiaticum*	3ADON	29.41471397	112.476406	28.48		Farmer owned field
170708	2017	Hunan	Rice	*F. asiaticum*	3ADON	29.41471397	112.476406	28.48		Farmer owned field
170707	2017	Hunan	Rice	*F. asiaticum*	3ADON	29.41471397	112.476406	28.48		Farmer owned field
170706	2017	Hunan	Rice	*F. asiaticum*	NIV	29.41471397	112.476406	28.48		Farmer owned field
170705	2017	Hunan	Rice	*F. asiaticum*	3ADON	29.41471397	112.476406	28.48		Farmer owned field
193276	2019	Hunan	Rice	*F. asiaticum*	3ADON	29.53115302	112.624226	27.716511		Farmer owned field
193275	2019	Hunan	Rice	*F. asiaticum*	3ADON	29.53115302	112.624226	27.716511		Farmer owned field
193274	2019	Hunan	Rice	*F. asiaticum*	3ADON	29.53115302	112.624226	27.716511		Farmer owned field
193273	2019	Hunan	Rice	*F. asiaticum*	3ADON	29.53115302	112.624226	27.716511		Farmer owned field
193272	2019	Hunan	Rice	*F. asiaticum*	3ADON	29.53115302	112.624226	27.716511		Farmer owned field
193268	2019	Hunan	Rice	*F. asiaticum*	3ADON	29.53115302	112.624226	27.716511		Farmer owned field
193267	2019	Hunan	Rice	*F. asiaticum*	NIV	29.53115302	112.624226	27.716511		Farmer owned field
193264	2019	Hunan	Rice	*F. asiaticum*	15ADON	29.53115302	112.624226	27.716511		Farmer owned field
193263	2019	Hunan	Rice	*F. asiaticum*	3ADON	29.53115302	112.624226	27.716511		Farmer owned field
193262	2019	Hunan	Rice	*F. asiaticum*	3ADON	29.53115302	112.624226	27.716511		Farmer owned field
193261	2019	Hunan	Rice	*F. asiaticum*	3ADON	29.53115302	112.624226	27.716511		Farmer owned field
193260	2019	Hunan	Rice	*F. asiaticum*	3ADON	29.53115302	112.624226	27.716511		Farmer owned field
193257	2019	Hunan	Rice	*F. asiaticum*	3ADON	29.53115302	112.624226	27.716511		Farmer owned field
193255	2019	Hunan	Rice	*F. asiaticum*	3ADON	29.53115302	112.624226	27.716511		Farmer owned field
193254	2019	Hunan	Rice	*F. asiaticum*	3ADON	29.53115302	112.624226	27.716511		Farmer owned field
193253	2019	Hunan	Rice	*F. asiaticum*	3ADON	29.53115302	112.624226	27.716511		Farmer owned field
170764	2017	Hunan	Rice	*F. asiaticum*	3ADON	29.53115302	112.624226	30.63		Farmer owned field
170763	2017	Hunan	Rice	*F. asiaticum*	3ADON	29.53115302	112.624226	30.63		Farmer owned field
170762	2017	Hunan	Rice	*F. asiaticum*	15ADON	29.53115302	112.624226	30.63		Farmer owned field
170761	2017	Hunan	Rice	*F. asiaticum*	3ADON	29.53115302	112.624226	30.63		Farmer owned field
170760	2017	Hunan	Rice	*F. asiaticum*	NIV	29.53115302	112.624226	30.63		Farmer owned field
170759	2017	Hunan	Rice	*F. asiaticum*	3ADON	29.53115302	112.624226	30.63		Farmer owned field
170757	2017	Hunan	Rice	*F. asiaticum*	3ADON	29.53115302	112.624226	30.63		Farmer owned field
170756	2017	Hunan	Rice	*F. asiaticum*	NIV	29.53115302	112.624226	30.63		Farmer owned field
170755	2017	Hunan	Rice	*F. asiaticum*	3ADON	29.53115302	112.624226	30.63		Farmer owned field
170754	2017	Hunan	Rice	*F. asiaticum*	NIV	29.53115302	112.624226	30.63		Farmer owned field
170752	2017	Hunan	Rice	*F. asiaticum*	3ADON	29.53115302	112.624226	30.63		Farmer owned field
170751	2017	Hunan	Rice	*F. asiaticum*	3ADON	29.53115302	112.624226	30.63		Farmer owned field

170750	2017	Hunan	Rice	*F. asiaticum*	NIV	29.53115302	112.624226	30.63		Farmer owned field
170749	2017	Hunan	Rice	*F. asiaticum*	3ADON	29.53115302	112.624226	30.63		Farmer owned field
170748	2017	Hunan	Rice	*F. asiaticum*	3ADON	29.53115302	112.624226	30.63		Farmer owned field
170747	2017	Hunan	Rice	*F. asiaticum*	NIV	29.53115302	112.624226	30.63		Farmer owned field
170746	2017	Hunan	Rice	*F. asiaticum*	3ADON	29.53115302	112.624226	30.63		Farmer owned field
170745	2017	Hunan	Rice	*F. asiaticum*	3ADON	29.53115302	112.624226	30.63		Farmer owned field
170744	2017	Hunan	Rice	*F. asiaticum*	3ADON	29.53115302	112.624226	30.63		Farmer owned field
170743	2017	Hunan	Rice	*F. asiaticum*	3ADON	29.53115302	112.624226	30.63		Farmer owned field
170742	2017	Hunan	Rice	*F. asiaticum*	3ADON	29.53115302	112.624226	30.63		Farmer owned field
170741	2017	Hunan	Rice	*F. asiaticum*	3ADON	29.53115302	112.624226	30.63		Farmer owned field
170740	2017	Hunan	Rice	*F. asiaticum*	15ADON	29.53115302	112.624226	30.63		Farmer owned field
170739	2017	Hunan	Rice	*F. asiaticum*	3ADON	29.53115302	112.624226	30.63		Farmer owned field
170738	2017	Hunan	Rice	*F. asiaticum*	NIV	29.53115302	112.624226	30.63		Farmer owned field
170737	2017	Hunan	Rice	*F. asiaticum*	3ADON	29.53115302	112.624226	30.63		Farmer owned field
170736	2017	Hunan	Rice	*F. asiaticum*	15ADON	29.53115302	112.624226	30.63		Farmer owned field
170735	2017	Hunan	Rice	*F. asiaticum*	3ADON	29.53115302	112.624226	30.63		Farmer owned field
170734	2017	Hunan	Rice	*F. asiaticum*	NIV	29.53115302	112.624226	30.63		Farmer owned field
170733	2017	Hunan	Rice	*F. asiaticum*	3ADON	29.53115302	112.624226	30.63		Farmer owned field
170731	2017	Hunan	Rice	*F. asiaticum*	3ADON	29.53115302	112.624226	30.63		Farmer owned field
170729	2017	Hunan	Rice	*F. asiaticum*	3ADON	29.53115302	112.624226	30.63		Farmer owned field
170728	2017	Hunan	Rice	*F. asiaticum*	NIV	29.53115302	112.624226	30.63		Farmer owned field
170727	2017	Hunan	Rice	*F. asiaticum*	15ADON	29.53115302	112.624226	30.63		Farmer owned field
170726	2017	Hunan	Rice	*F. asiaticum*	3ADON	29.53115302	112.624226	30.63		Farmer owned field
194186	2019	Hunan	Rice	*F. asiaticum*	NIV	29.55987697	112.806131	36.176529		Farmer owned field
194185	2019	Hunan	Rice	*F. asiaticum*	NIV	29.55987697	112.806131	36.176529		Farmer owned field
194184	2019	Hunan	Rice	*F. asiaticum*	3ADON	29.55987697	112.806131	36.176529		Farmer owned field
194183	2019	Hunan	Rice	*F. asiaticum*	3ADON	29.55987697	112.806131	36.176529		Farmer owned field
194182	2019	Hunan	Rice	*F. asiaticum*	3ADON	29.55987697	112.806131	36.176529		Farmer owned field
194181	2019	Hunan	Rice	*F. asiaticum*	NIV	29.55987697	112.806131	36.176529		Farmer owned field
194176	2019	Hunan	Rice	*F. asiaticum*	3ADON	29.55987697	112.806131	36.176529		Farmer owned field
170788	2017	Hunan	Rice	*F. asiaticum*	3ADON	29.55987697	112.806131	36.98		Farmer owned field
170787	2017	Hunan	Rice	*F. asiaticum*	NIV	29.55987697	112.806131	36.98		Farmer owned field
170786	2017	Hunan	Rice	*F. asiaticum*	3ADON	29.55987697	112.806131	36.98		Farmer owned field
170784	2017	Hunan	Rice	*F. asiaticum*	NIV	29.55987697	112.806131	36.98		Farmer owned field
170783	2017	Hunan	Rice	*F. asiaticum*	3ADON	29.55987697	112.806131	36.98		Farmer owned field

170782	2017	Hunan	Rice	*F. asiaticum*	NIV	29.55987697	112.806131	36.98		Farmer owned field
170781	2017	Hunan	Rice	*F. asiaticum*	3ADON	29.55987697	112.806131	36.98		Farmer owned field
170780	2017	Hunan	Rice	*F. asiaticum*	3ADON	29.55987697	112.806131	36.98		Farmer owned field
170779	2017	Hunan	Rice	*F. asiaticum*	3ADON	29.55987697	112.806131	36.98		Farmer owned field
170778	2017	Hunan	Rice	*F. asiaticum*	3ADON	29.55987697	112.806131	36.98		Farmer owned field
170777	2017	Hunan	Rice	*F. asiaticum*	NIV	29.55987697	112.806131	36.98		Farmer owned field
170775	2017	Hunan	Rice	*F. asiaticum*	NIV	29.55987697	112.806131	36.98		Farmer owned field
170774	2017	Hunan	Rice	*F. asiaticum*	3ADON	29.55987697	112.806131	36.98		Farmer owned field
170773	2017	Hunan	Rice	*F. asiaticum*	3ADON	29.55987697	112.806131	36.98		Farmer owned field
170772	2017	Hunan	Rice	*F. asiaticum*	NIV	29.55987697	112.806131	36.98		Farmer owned field
170771	2017	Hunan	Rice	*F. asiaticum*	3ADON	29.55987697	112.806131	36.98		Farmer owned field
170770	2017	Hunan	Rice	*F. asiaticum*	3ADON	29.55987697	112.806131	36.98		Farmer owned field
170769	2017	Hunan	Rice	*F. asiaticum*	NIV	29.55987697	112.806131	36.98		Farmer owned field
170768	2017	Hunan	Rice	*F. asiaticum*	3ADON	29.55987697	112.806131	36.98		Farmer owned field
170767	2017	Hunan	Rice	*F. asiaticum*	NIV	29.55987697	112.806131	36.98		Farmer owned field
170766	2017	Hunan	Rice	*F. asiaticum*	3ADON	29.55987697	112.806131	36.98		Farmer owned field
170765	2017	Hunan	Rice	*F. asiaticum*	3ADON	29.55987697	112.806131	36.98		Farmer owned field
181014	2018	Hubei	Wheat	*F. asiaticum*	3ADON	NA	NA	NA	Commercialized variety	Farmer owned field
181013	2018	Hubei	Wheat	*F. asiaticum*	3ADON	NA	NA	NA	Commercialized variety	Farmer owned field
191587	2019	Anhui	Wheat	*F. asiaticum*	3ADON	33.324217	116.352031	NA	Commercialized variety	Farmer owned field
181012	2018	Hubei	Wheat	*F. asiaticum*	3ADON	NA	NA	NA	Commercialized variety	Farmer owned field
191583	2019	Anhui	Wheat	*F. asiaticum*	3ADON	33.324217	116.352031	NA	Commercialized variety	Farmer owned field
191581	2019	Anhui	Wheat	*F. asiaticum*	3ADON	33.324217	116.352031	NA	Commercialized variety	Farmer owned field
191580	2019	Anhui	Wheat	*F. asiaticum*	3ADON	33.324217	116.352031	NA	Commercialized variety	Farmer owned field
181011	2018	Hubei	Wheat	*F. asiaticum*	3ADON	NA	NA	NA	Commercialized variety	Farmer owned field
181010	2018	Hubei	Wheat	*F. asiaticum*	3ADON	NA	NA	NA	Commercialized variety	Farmer owned field
191578	2019	Anhui	Wheat	*F. asiaticum*	3ADON	33.324217	116.352031	NA	Commercialized variety	Farmer owned field
181009	2018	Hubei	Wheat	*F. asiaticum*	3ADON	NA	NA	NA	Commercialized variety	Farmer owned field
191570	2019	Anhui	Wheat	*F. asiaticum*	3ADON	33.324217	116.352031	NA	Commercialized variety	Farmer owned field
181008	2018	Hubei	Wheat	*F. asiaticum*	15ADON	NA	NA	NA	Commercialized variety	Farmer owned field
191566	2019	Anhui	Wheat	*F. asiaticum*	15ADON	33.324217	116.352031	NA	Commercialized variety	Farmer owned field
190772	2019	Anhui	Wheat	*F. asiaticum*	3ADON	33.51352103	116.959197	37.586994	Commercialized variety	Farmer owned field
190771	2019	Anhui	Wheat	*F. asiaticum*	3ADON	33.51352103	116.959197	37.586994	Commercialized variety	Farmer owned field
190767	2019	Anhui	Wheat	*F. asiaticum*	3ADON	33.51352103	116.959197	37.586994	Commercialized variety	Farmer owned field
181007	2018	Hubei	Wheat	*F. asiaticum*	3ADON	NA	NA	NA	Commercialized variety	Farmer owned field

181006	2018	Hubei	Wheat	*F. asiaticum*	15ADON	NA	NA	NA	Commercialized variety	Farmer owned field
181005	2018	Hubei	Wheat	*F. asiaticum*	15ADON	NA	NA	NA	Commercialized variety	Farmer owned field
190765	2019	Anhui	Wheat	*F. asiaticum*	3ADON	33.51352103	116.959197	37.586994	Commercialized variety	Farmer owned field
190763	2019	Anhui	Wheat	*F. asiaticum*	3ADON	33.51352103	116.959197	37.586994	Commercialized variety	Farmer owned field
181004	2018	Hubei	Wheat	*F. asiaticum*	3ADON	NA	NA	NA	Commercialized variety	Farmer owned field
190762	2019	Anhui	Wheat	*F. asiaticum*	3ADON	33.51352103	116.959197	37.586994	Commercialized variety	Farmer owned field
190760	2019	Anhui	Wheat	*F. asiaticum*	3ADON	33.51352103	116.959197	37.586994	Commercialized variety	Farmer owned field
181003	2018	Hubei	Wheat	*F. asiaticum*	15ADON	NA	NA	NA	Commercialized variety	Farmer owned field
181002	2018	Hubei	Wheat	*F. asiaticum*	3ADON	NA	NA	NA	Commercialized variety	Farmer owned field
181001	2018	Hubei	Wheat	*F. asiaticum*	3ADON	NA	NA	NA	Commercialized variety	Farmer owned field
190759	2019	Anhui	Wheat	*F. asiaticum*	NIV	33.51352103	116.959197	37.586994	Commercialized variety	Farmer owned field
190758	2019	Anhui	Wheat	*F. asiaticum*	3ADON	33.51352103	116.959197	37.586994	Commercialized variety	Farmer owned field
181000	2018	Hubei	Wheat	*F. asiaticum*	3ADON	NA	NA	NA	Commercialized variety	Farmer owned field
190757	2019	Anhui	Wheat	*F. asiaticum*	3ADON	33.51352103	116.959197	37.586994	Commercialized variety	Farmer owned field
180999	2018	Hubei	Wheat	*F. asiaticum*	15ADON	NA	NA	NA	Commercialized variety	Farmer owned field
190756	2019	Anhui	Wheat	*F. asiaticum*	3ADON	33.51352103	116.959197	37.586994	Commercialized variety	Farmer owned field
190752	2019	Anhui	Wheat	*F. asiaticum*	3ADON	33.51352103	116.959197	37.586994	Commercialized variety	Farmer owned field
180998	2018	Hubei	Wheat	*F. asiaticum*	3ADON	NA	NA	NA	Commercialized variety	Farmer owned field
190751	2019	Anhui	Wheat	*F. asiaticum*	3ADON	33.51352103	116.959197	37.586994	Commercialized variety	Farmer owned field
180997	2018	Hubei	Wheat	*F. asiaticum*	NIV	NA	NA	NA	Commercialized variety	Farmer owned field
180996	2018	Hubei	Wheat	*F. asiaticum*	3ADON	NA	NA	NA	Commercialized variety	Farmer owned field
190747	2019	Anhui	Wheat	*F. asiaticum*	3ADON	33.51352103	116.959197	37.586994	Commercialized variety	Farmer owned field
190746	2019	Anhui	Wheat	*F. asiaticum*	3ADON	33.51352103	116.959197	37.586994	Commercialized variety	Farmer owned field
190744	2019	Anhui	Wheat	*F. asiaticum*	NIV	33.51352103	116.959197	37.586994	Commercialized variety	Farmer owned field
180165	2018	Hubei	Wheat	*F. asiaticum*	3ADON	NA	NA	NA	Commercialized variety	Farmer owned field
180164	2018	Hubei	Wheat	*F. asiaticum*	NIV	NA	NA	NA	Commercialized variety	Farmer owned field
190743	2019	Anhui	Wheat	*F. asiaticum*	3ADON	33.51352103	116.959197	37.586994	Commercialized variety	Farmer owned field
190741	2019	Anhui	Wheat	*F. asiaticum*	3ADON	33.51352103	116.959197	37.586994	Commercialized variety	Farmer owned field
180163	2018	Hubei	Wheat	*F. asiaticum*	NIV	NA	NA	NA	Commercialized variety	Farmer owned field
180162	2018	Hubei	Wheat	*F. asiaticum*	15ADON	NA	NA	NA	Commercialized variety	Farmer owned field
180161	2018	Hubei	Wheat	*F. asiaticum*	3ADON	NA	NA	NA	Commercialized variety	Farmer owned field
190738	2019	Anhui	Wheat	*F. asiaticum*	3ADON	33.51352103	116.959197	37.586994	Commercialized variety	Farmer owned field
190737	2019	Anhui	Wheat	*F. asiaticum*	3ADON	33.51352103	116.959197	37.586994	Commercialized variety	Farmer owned field
180160	2018	Hubei	Wheat	*F. asiaticum*	NIV	NA	NA	NA	Commercialized variety	Farmer owned field
190735	2019	Anhui	Wheat	*F. asiaticum*	3ADON	33.51352103	116.959197	37.586994	Commercialized variety	Farmer owned field

182385	2018	Anhui	Wheat	*F. asiaticum*	NIV	33.55115804	116.098637	15.645923	Commercialized variety	Farmer owned field
180159	2018	Hubei	Wheat	*F. asiaticum*	3ADON	NA	NA	NA	Commercialized variety	Farmer owned field
180158	2018	Hubei	Wheat	*F. asiaticum*	3ADON	NA	NA	NA	Commercialized variety	Farmer owned field
182379	2018	Anhui	Wheat	*F. asiaticum*	3ADON	33.55115804	116.098637	15.645923	Commercialized variety	Farmer owned field
180157	2018	Hubei	Wheat	*F. asiaticum*	3ADON	NA	NA	NA	Commercialized variety	Farmer owned field
182378	2018	Anhui	Wheat	*F. asiaticum*	3ADON	33.55115804	116.098637	15.645923	Commercialized variety	Farmer owned field
191048	2019	Anhui	Wheat	*F. asiaticum*	3ADON	33.95522201	116.89019	44.893009	Commercialized variety	Farmer owned field
191047	2019	Anhui	Wheat	*F. asiaticum*	3ADON	33.95522201	116.89019	44.893009	Commercialized variety	Farmer owned field
191046	2019	Anhui	Wheat	*F. asiaticum*	3ADON	33.95522201	116.89019	44.893009	Commercialized variety	Farmer owned field
191045	2019	Anhui	Wheat	*F. asiaticum*	3ADON	33.95522201	116.89019	44.893009	Commercialized variety	Farmer owned field
191044	2019	Anhui	Wheat	*F. asiaticum*	3ADON	33.95522201	116.89019	44.893009	Commercialized variety	Farmer owned field
191043	2019	Anhui	Wheat	*F. asiaticum*	3ADON	33.95522201	116.89019	44.893009	Commercialized variety	Farmer owned field
180156	2018	Hubei	Wheat	*F. asiaticum*	3ADON	NA	NA	NA	Commercialized variety	Farmer owned field
191042	2019	Anhui	Wheat	*F. asiaticum*	3ADON	33.95522201	116.89019	44.893009	Commercialized variety	Farmer owned field
180155	2018	Hubei	Wheat	*F. asiaticum*	3ADON	NA	NA	NA	Commercialized variety	Farmer owned field
191040	2019	Anhui	Wheat	*F. asiaticum*	3ADON	33.95522201	116.89019	44.893009	Commercialized variety	Farmer owned field
191039	2019	Anhui	Wheat	*F. asiaticum*	3ADON	33.95522201	116.89019	44.893009	Commercialized variety	Farmer owned field
191037	2019	Anhui	Wheat	*F. asiaticum*	3ADON	33.95522201	116.89019	44.893009	Commercialized variety	Farmer owned field
180154	2018	Hubei	Wheat	*F. asiaticum*	3ADON	NA	NA	NA	Commercialized variety	Farmer owned field
191035	2019	Anhui	Wheat	*F. asiaticum*	3ADON	33.95522201	116.89019	44.893009	Commercialized variety	Farmer owned field
191034	2019	Anhui	Wheat	*F. asiaticum*	3ADON	33.95522201	116.89019	44.893009	Commercialized variety	Farmer owned field
180153	2018	Hubei	Wheat	*F. asiaticum*	3ADON	NA	NA	NA	Commercialized variety	Farmer owned field
180152	2018	Hubei	Wheat	*F. asiaticum*	NIV	NA	NA	NA	Commercialized variety	Farmer owned field
191030	2019	Anhui	Wheat	*F. asiaticum*	3ADON	33.95522201	116.89019	44.893009	Commercialized variety	Farmer owned field
191027	2019	Anhui	Wheat	*F. asiaticum*	NIV	33.95522201	116.89019	44.893009	Commercialized variety	Farmer owned field
180121	2018	Anhui	Wheat	*F. asiaticum*	NIV	NA	NA	NA	Commercialized variety	Farmer owned field
180120	2018	Anhui	Wheat	*F. asiaticum*	3ADON	NA	NA	NA	Commercialized variety	Farmer owned field
180119	2018	Anhui	Wheat	*F. asiaticum*	3ADON	NA	NA	NA	Commercialized variety	Farmer owned field
180118	2018	Anhui	Wheat	*F. asiaticum*	3ADON	NA	NA	NA	Commercialized variety	Farmer owned field
180151	2018	Hubei	Wheat	*F. asiaticum*	3ADON	NA	NA	NA	Commercialized variety	Farmer owned field
180117	2018	Anhui	Wheat	*F. asiaticum*	3ADON	NA	NA	NA	Commercialized variety	Farmer owned field
180150	2018	Hubei	Wheat	*F. asiaticum*	NIV	NA	NA	NA	Commercialized variety	Farmer owned field
180116	2018	Anhui	Wheat	*F. asiaticum*	3ADON	NA	NA	NA	Commercialized variety	Farmer owned field
180149	2018	Hubei	Wheat	*F. asiaticum*	3ADON	NA	NA	NA	Commercialized variety	Farmer owned field
180114	2018	Anhui	Wheat	*F. asiaticum*	3ADON	NA	NA	NA	Commercialized variety	Farmer owned field

180148	2018	Hubei	Wheat	*F. asiaticum*	3ADON	NA	NA	NA	Commercialized variety	Farmer owned field
180113	2018	Anhui	Wheat	*F. asiaticum*	3ADON	NA	NA	NA	Commercialized variety	Farmer owned field
180112	2018	Anhui	Wheat	*F. asiaticum*	3ADON	NA	NA	NA	Commercialized variety	Farmer owned field
180111	2018	Anhui	Wheat	*F. asiaticum*	3ADON	NA	NA	NA	Commercialized variety	Farmer owned field
180147	2018	Hubei	Wheat	*F. asiaticum*	NIV	NA	NA	NA	Commercialized variety	Farmer owned field
180110	2018	Anhui	Wheat	*F. asiaticum*	3ADON	NA	NA	NA	Commercialized variety	Farmer owned field
180146	2018	Hubei	Wheat	*F. asiaticum*	NIV	NA	NA	NA	Commercialized variety	Farmer owned field
180145	2018	Hubei	Wheat	*F. asiaticum*	3ADON	NA	NA	NA	Commercialized variety	Farmer owned field
180141	2018	Hubei	Wheat	*F. asiaticum*	3ADON	NA	NA	NA	Commercialized variety	Farmer owned field
180109	2018	Anhui	Wheat	*F. asiaticum*	NIV	NA	NA	NA	Commercialized variety	Farmer owned field
180108	2018	Anhui	Wheat	*F. asiaticum*	NIV	NA	NA	NA	Commercialized variety	Farmer owned field
180140	2018	Hubei	Wheat	*F. asiaticum*	3ADON	NA	NA	NA	Commercialized variety	Farmer owned field
180107	2018	Anhui	Wheat	*F. asiaticum*	3ADON	NA	NA	NA	Commercialized variety	Farmer owned field
180139	2018	Hubei	Wheat	*F. asiaticum*	3ADON	NA	NA	NA	Commercialized variety	Farmer owned field
180106	2018	Anhui	Wheat	*F. asiaticum*	3ADON	NA	NA	NA	Commercialized variety	Farmer owned field
180105	2018	Anhui	Wheat	*F. asiaticum*	3ADON	NA	NA	NA	Commercialized variety	Farmer owned field
180104	2018	Anhui	Wheat	*F. asiaticum*	3ADON	NA	NA	NA	Commercialized variety	Farmer owned field
180138	2018	Hubei	Wheat	*F. asiaticum*	3ADON	NA	NA	NA	Commercialized variety	Farmer owned field
180216	2018	Jiangsu	Wheat	*F. asiaticum*	3ADON	NA	NA	NA	Commercialized variety	Commercial farm
180218	2018	Jiangsu	Wheat	*F. asiaticum*	NIV	NA	NA	NA	Commercialized variety	Commercial farm
180219	2018	Jiangsu	Wheat	*F. asiaticum*	3ADON	NA	NA	NA	Commercialized variety	Commercial farm
140005	2014	Jiangsu	Rice	*F. asiaticum*	3ADON	NA	NA	NA		NA
140006	2014	Jiangsu	Rice	*F. asiaticum*	3ADON	NA	NA	NA		NA
140007	2014	Jiangsu	Rice	*F. asiaticum*	3ADON	NA	NA	NA		NA
140008	2014	Jiangsu	Rice	*F. asiaticum*	3ADON	NA	NA	NA		NA
180103	2018	Anhui	Wheat	*F. asiaticum*	3ADON	NA	NA	NA	Commercialized variety	Farmer owned field
180102	2018	Anhui	Wheat	*F. asiaticum*	3ADON	NA	NA	NA	Commercialized variety	Farmer owned field
180101	2018	Anhui	Wheat	*F. asiaticum*	3ADON	NA	NA	NA	Commercialized variety	Farmer owned field
180280	2018	sichuan	Wheat	*F. asiaticum*	NIV	NA	NA	NA	Commercialized variety	Farmer owned field
180281	2018	sichuan	Wheat	*F. asiaticum*	NIV	NA	NA	NA	Commercialized variety	Farmer owned field
180282	2018	sichuan	Wheat	*F. asiaticum*	NIV	NA	NA	NA	Commercialized variety	Farmer owned field
180283	2018	sichuan	Wheat	*F. asiaticum*	NIV	NA	NA	NA	Commercialized variety	Farmer owned field
140052	2014	Fujian	Rice	*F. asiaticum*	15ADON	NA	NA	NA		NA
180100	2018	Anhui	Wheat	*F. asiaticum*	3ADON	NA	NA	NA	Commercialized variety	Farmer owned field
180099	2018	Anhui	Wheat	*F. asiaticum*	NIV	NA	NA	NA	Commercialized variety	Farmer owned field

180098	2018	Anhui	Wheat	*F. asiaticum*	NIV	NA	NA	NA	Commercialized variety	Farmer owned field
180097	2018	Anhui	Wheat	*F. asiaticum*	NIV	NA	NA	NA	Commercialized variety	Farmer owned field
180096	2018	Anhui	Wheat	*F. asiaticum*	NIV	NA	NA	NA	Commercialized variety	Farmer owned field
180095	2018	Anhui	Wheat	*F. asiaticum*	NIV	NA	NA	NA	Commercialized variety	Farmer owned field
180094	2018	Anhui	Wheat	*F. asiaticum*	NIV	NA	NA	NA	Commercialized variety	Farmer owned field
140012	2014	Anhui	Wheat	*F. asiaticum*	3ADON	NA	NA	NA	Commercialized variety	NA
140013	2014	Anhui	Wheat	*F. asiaticum*	3ADON	NA	NA	NA	Commercialized variety	NA
140014	2014	Anhui	Wheat	*F. asiaticum*	3ADON	NA	NA	NA	Commercialized variety	NA
140009	2014	Jiangsu	Rice	*F. asiaticum*	3ADON	NA	NA	NA		NA
140010	2014	Jiangsu	Rice	*F. asiaticum*	3ADON	NA	NA	NA		NA
140011	2014	Jiangsu	Rice	*F. asiaticum*	3ADON	NA	NA	NA		NA
180137	2018	Hubei	Wheat	*F. asiaticum*	3ADON	NA	NA	NA	Commercialized variety	Farmer owned field
180136	2018	Hubei	Wheat	*F. asiaticum*	15ADON	NA	NA	NA	Commercialized variety	Farmer owned field
180135	2018	Hubei	Wheat	*F. asiaticum*	3ADON	NA	NA	NA	Commercialized variety	Farmer owned field
180133	2018	Hubei	Wheat	*F. asiaticum*	3ADON	NA	NA	NA	Commercialized variety	Farmer owned field
180131	2018	Hubei	Wheat	*F. asiaticum*	3ADON	NA	NA	NA	Commercialized variety	Farmer owned field
180130	2018	Hubei	Wheat	*F. asiaticum*	3ADON	NA	NA	NA	Commercialized variety	Farmer owned field
180129	2018	Hubei	Wheat	*F. asiaticum*	3ADON	NA	NA	NA	Commercialized variety	Farmer owned field
180128	2018	Hubei	Wheat	*F. asiaticum*	3ADON	NA	NA	NA	Commercialized variety	Farmer owned field
180127	2018	Hubei	Wheat	*F. asiaticum*	NIV	NA	NA	NA	Commercialized variety	Farmer owned field
180126	2018	Hubei	Wheat	*F. asiaticum*	3ADON	NA	NA	NA	Commercialized variety	Farmer owned field
180125	2018	Hubei	Wheat	*F. asiaticum*	3ADON	NA	NA	NA	Commercialized variety	Farmer owned field
180124	2018	Hubei	Wheat	*F. asiaticum*	3ADON	NA	NA	NA	Commercialized variety	Farmer owned field
180123	2018	Hubei	Wheat	*F. asiaticum*	3ADON	NA	NA	NA	Commercialized variety	Farmer owned field

Table S1 Sheet2 245 *F. asiaticum* sequencing strains used for constructing the pangenome dataset

Strain	Species	Chemotype	Origin	Province	Host	Crop_Rotation	Year	Latitude	Longitude	Altitude	Wheat variety	Field type
140001	*F. asiaticum*	3ADON	MLYRP	Hubei	Rice	Wheat-Rice	2014	30.892518	113.933865	24		Commercial farm
140002	*F. asiaticum*	3ADON	MLYRP	Hubei	Rice	Wheat-Rice	2014	30.892518	113.933865	24		Commercial farm
140003	*F. asiaticum*	3ADON	MLYRP	Hubei	Rice	Wheat-Rice	2014	30.892518	113.933865	24		Commercial farm
140004	*F. asiaticum*	3ADON	MLYRP	Hubei	Rice	Wheat-Rice	2014	30.892518	113.933865	24		Commercial farm
140005	*F. asiaticum*	3ADON	MLYRP	Jiangsu	Rice	Wheat-Rice	2014	NA	NA	NA		NA
140006	*F. asiaticum*	3ADON	MLYRP	Jiangsu	Rice	Wheat-Rice	2014	NA	NA	NA		NA
140007	*F. asiaticum*	3ADON	MLYRP	Jiangsu	Rice	Wheat-Rice	2014	NA	NA	NA		NA
140008	*F. asiaticum*	3ADON	MLYRP	Jiangsu	Rice	Wheat-Rice	2014	NA	NA	NA		NA
140009	*F. asiaticum*	3ADON	MLYRP	Jiangsu	Rice	Wheat-Rice	2014	NA	NA	NA		NA
140010	*F. asiaticum*	3ADON	MLYRP	Jiangsu	Rice	Wheat-Rice	2014	NA	NA	NA		NA
140011	*F. asiaticum*	3ADON	MLYRP	Jiangsu	Rice	Wheat-Rice	2014	NA	NA	NA		NA
140012	*F. asiaticum*	3ADON	MLYRP	Anhui	Wheat	Wheat-Rice	2014	NA	NA	NA	Commercialized variety	NA
140013	*F. asiaticum*	3ADON	MLYRP	Anhui	Wheat	Wheat-Rice	2014	NA	NA	NA	Commercialized variety	NA
140014	*F. asiaticum*	3ADON	MLYRP	Anhui	Wheat	Wheat-Rice	2014	NA	NA	NA	Commercialized variety	NA
140015	*F. asiaticum*	3ADON	MLYRP	Hubei	Wheat	Wheat-Rice	2014	30.892518	113.933865	24	Commercialized variety	Commercial farm
140016	*F. asiaticum*	3ADON	MLYRP	Hubei	Wheat	Wheat-Rice	2014	30.892518	113.933865	24	Commercialized variety	Commercial farm
140017	*F. asiaticum*	3ADON	MLYRP	Hubei	Wheat	Wheat-Rice	2014	30.892518	113.933865	24	Commercialized variety	Commercial farm
140018	*F. asiaticum*	3ADON	MLYRP	Hubei	Wheat	Wheat-Rice	2014	30.892518	113.933865	24	Commercialized variety	Commercial farm
140019	*F. asiaticum*	3ADON	MLYRP	Hubei	Wheat	Wheat-Rice	2014	30.892518	113.933865	24	Commercialized variety	Commercial farm
140020	*F. asiaticum*	3ADON	MLYRP	Hubei	Wheat	Wheat-Rice	2014	30.892518	113.933865	24	Commercialized variety	Commercial farm
140021	*F. asiaticum*	3ADON	MLYRP	Hubei	Wheat	Wheat-Rice	2014	30.892518	113.933865	24	Commercialized variety	Commercial farm
140022	*F. asiaticum*	3ADON	MLYRP	Hubei	Wheat	Wheat-Rice	2014	30.892518	113.933865	24	Commercialized variety	Commercial farm
140023	*F. asiaticum*	3ADON	MLYRP	Hubei	Wheat	Wheat-Rice	2014	30.892518	113.933865	24	Commercialized variety	Commercial farm
140024	*F. asiaticum*	NIV	Sichuan	Sichuan	Wheat	Wheat-Rice	2014	31.80769402	104.749059	609.975159	Commercialized variety	Farmer owned field
140025	*F. asiaticum*	NIV	Sichuan	Sichuan	Wheat	Wheat-Rice	2014	31.80769402	104.749059	609.975159	Commercialized variety	Farmer owned field
140026	*F. asiaticum*	NIV	Sichuan	Sichuan	Wheat	Wheat-Rice	2014	31.121126	105.09804	383	Commercialized variety	Farmer owned field
140027	*F. asiaticum*	NIV	Sichuan	Sichuan	Wheat	Wheat-Rice	2014	31.121126	105.09804	383	Commercialized variety	Farmer owned field
140052	*F. asiaticum*	15ADON	Fujian	Fujian	Rice	Rice	2014	NA	NA	NA		Farmer owned field
160205	*F. asiaticum*	3ADON	MLYRP	Anhui	Rice	Wheat-Rice	2016	30.6542879	117.4921052	14		Farmer owned field
160226	*F. asiaticum*	3ADON	MLYRP	Anhui	Rice	Wheat-Rice	2016	32.353038	116.277911	36		Farmer owned field
160232	*F. asiaticum*	3ADON	MLYRP	Anhui	Rice	Wheat-Rice	2016	32.353038	116.277911	36		Farmer owned field
160237	*F. asiaticum*	3ADON	MLYRP	Anhui	Rice	Wheat-Rice	2016	32.713877	116.704162	22		Farmer owned field

160242	*F. asiaticum*	3ADON	MLYRP	Anhui	Rice	Wheat-Rice	2016	32.713877	116.704162	22	Farmer owned field
160264	*F. asiaticum*	3ADON	MLYRP	Anhui	Rice	Wheat-Rice	2016	32.713877	116.704162	22	Farmer owned field
160270	*F. asiaticum*	3ADON	MLYRP	Anhui	Rice	Wheat-Rice	2016	32.713877	116.704162	22	Farmer owned field
160277	*F. asiaticum*	3ADON	MLYRP	Anhui	Rice	Wheat-Rice	2016	32.713877	116.704162	22	Farmer owned field
160280	*F. asiaticum*	3ADON	MLYRP	Anhui	Rice	Wheat-Rice	2016	32.713877	116.704162	22	Farmer owned field
160282	*F. asiaticum*	3ADON	MLYRP	Anhui	Rice	Wheat-Rice	2016	32.713877	116.704162	22	Farmer owned field
160863	*F. asiaticum*	3ADON	MLYRP	Jiangsu	Rice	Wheat-Rice	2016	31.457735	121.13055	4	Farmer owned field
160898	*F. asiaticum*	3ADON	MLYRP	Jiangsu	Rice	Wheat-Rice	2016	31.982751	120.277138	2	Farmer owned field
160952	*F. asiaticum*	3ADON	MLYRP	Jiangsu	Rice	Wheat-Rice	2016	32.3731728	120.5719417	6	Farmer owned field
170343	*F. asiaticum*	NIV	Hunan	Hunan	Rice	Rice	2017	28.62087197	112.339678	34.65	Farmer owned field
170347	*F. asiaticum*	3ADON	Hunan	Hunan	Rice	Rice	2017	28.62087197	112.339678	34.65	Farmer owned field
170364	*F. asiaticum*	3ADON	Hunan	Hunan	Rice	Rice	2017	28.62087197	112.339678	34.65	Farmer owned field
170372	*F. asiaticum*	NIV	Hunan	Hunan	Rice	Rice	2017	28.62087197	112.339678	34.65	Farmer owned field
170382	*F. asiaticum*	3ADON	Hunan	Hunan	Rice	Rice	2017	28.73773397	112.32034	NA	Farmer owned field
170383	*F. asiaticum*	NIV	Hunan	Hunan	Rice	Rice	2017	28.73773397	112.32034	NA	Farmer owned field
170430	*F. asiaticum*	3ADON	Hunan	Hunan	Rice	Rice	2017	28.883176	112.295044	NA	Farmer owned field
170431	*F. asiaticum*	NIV	Hunan	Hunan	Rice	Rice	2017	28.883176	112.295044	NA	Farmer owned field
170460	*F. asiaticum*	3ADON	Hunan	Hunan	Rice	Rice	2017	28.97530401	112.363732	NA	Farmer owned field
170464	*F. asiaticum*	NIV	Hunan	Hunan	Rice	Rice	2017	28.97530401	112.363732	NA	Farmer owned field
170542	*F. asiaticum*	3ADON	Hunan	Hunan	Rice	Rice	2017	28.94113996	112.486655	28.18	Farmer owned field
170545	*F. asiaticum*	NIV	Hunan	Hunan	Rice	Rice	2017	28.94113996	112.486655	28.18	Farmer owned field
170566	*F. asiaticum*	NIV	Hunan	Hunan	Rice	Rice	2017	28.94113996	112.486655	28.18	Farmer owned field
170630	*F. asiaticum*	3ADON	Hunan	Hunan	Rice	Rice	2017	29.19206598	112.320635	28	Farmer owned field
170631	*F. asiaticum*	NIV	Hunan	Hunan	Rice	Rice	2017	29.19206598	112.320635	28	Farmer owned field
170665	*F. asiaticum*	3ADON	Hunan	Hunan	Rice	Rice	2017	29.34845896	112.384461	30	Farmer owned field
170677	*F. asiaticum*	NIV	Hunan	Hunan	Rice	Rice	2017	29.34845896	112.384461	30	Farmer owned field
170705	*F. asiaticum*	3ADON	Hunan	Hunan	Rice	Rice	2017	29.41471397	112.476406	28.48	Farmer owned field
170706	*F. asiaticum*	NIV	Hunan	Hunan	Rice	Rice	2017	29.41471397	112.476406	28.48	Farmer owned field
170719	*F. asiaticum*	3ADON	Hunan	Hunan	Rice	Rice	2017	29.41471397	112.476406	28.48	Farmer owned field
170726	*F. asiaticum*	3ADON	Hunan	Hunan	Rice	Rice	2017	29.53115302	112.624226	30.63	Farmer owned field
170728	*F. asiaticum*	NIV	Hunan	Hunan	Rice	Rice	2017	29.53115302	112.624226	30.63	Farmer owned field
170765	*F. asiaticum*	3ADON	Hunan	Hunan	Rice	Rice	2017	29.55987697	112.806131	36.98	Farmer owned field
170767	*F. asiaticum*	NIV	Hunan	Hunan	Rice	Rice	2017	29.55987697	112.806131	36.98	Farmer owned field
170784	*F. asiaticum*	NIV	Hunan	Hunan	Rice	Rice	2017	29.55987697	112.806131	36.98	Farmer owned field
170788	*F. asiaticum*	3ADON	Hunan	Hunan	Rice	Rice	2017	29.55987697	112.806131	36.98	Farmer owned field

170789	*F. asiaticum*	NIV	Hunan	Hunan	Rice	Rice	2017	29.31476397	113.179366	35.34	Farmer owned field
170792	*F. asiaticum*	3ADON	Hunan	Hunan	Rice	Rice	2017	29.31476397	113.179366	35.34	Farmer owned field
170824	*F. asiaticum*	NIV	Hunan	Hunan	Rice	Rice	2017	29.14365001	113.237246	37.35	Farmer owned field
170827	*F. asiaticum*	3ADON	Hunan	Hunan	Rice	Rice	2017	29.14365001	113.237246	37.35	Farmer owned field
170867	*F. asiaticum*	NIV	Hunan	Hunan	Rice	Rice	2017	28.93416203	113.242577	58.17	Farmer owned field
170876	*F. asiaticum*	3ADON	Hunan	Hunan	Rice	Rice	2017	28.93416203	113.242577	58.17	Farmer owned field
170880	*F. asiaticum*	3ADON	Hunan	Hunan	Rice	Rice	2017	28.93416203	113.242577	58.17	Farmer owned field
170888	*F. asiaticum*	NIV	Hunan	Hunan	Rice	Rice	2017	28.93416203	113.242577	58.17	Farmer owned field
170907	*F. asiaticum*	3ADON	Hunan	Hunan	Rice	Rice	2017	28.793654	113.167283	40.16	Farmer owned field
170908	*F. asiaticum*	NIV	Hunan	Hunan	Rice	Rice	2017	28.793654	113.167283	40.16	Farmer owned field
170944	*F. asiaticum*	3ADON	Hunan	Hunan	Rice	Rice	2017	28.19698798	112.766222	72.16	Farmer owned field
170945	*F. asiaticum*	NIV	Hunan	Hunan	Rice	Rice	2017	28.19698798	112.766222	72.16	Farmer owned field
170948	*F. asiaticum*	3ADON	Hunan	Hunan	Rice	Rice	2017	28.19698798	112.766222	72.16	Farmer owned field
170960	*F. asiaticum*	3ADON	Hunan	Hunan	Rice	Rice	2017	28.19918202	112.683053	93.43	Farmer owned field
170988	*F. asiaticum*	NIV	Hunan	Hunan	Rice	Rice	2017	28.12392502	112.623662	173.31	Farmer owned field
170991	*F. asiaticum*	3ADON	Hunan	Hunan	Rice	Rice	2017	28.12392502	112.623662	173.31	Farmer owned field
171005	*F. asiaticum*	NIV	Hunan	Hunan	Rice	Rice	2017	28.12392502	112.623662	173.31	Farmer owned field
171011	*F. asiaticum*	NIV	Hunan	Hunan	Rice	Rice	2017	28.02993396	112.623799	60.99	Farmer owned field
171013	*F. asiaticum*	3ADON	Hunan	Hunan	Rice	Rice	2017	28.02993396	112.623799	60.99	Farmer owned field
171025	*F. asiaticum*	3ADON	Hunan	Hunan	Rice	Rice	2017	28.02993396	112.623799	60.99	Farmer owned field
171029	*F. asiaticum*	3ADON	Hunan	Hunan	Rice	Rice	2017	27.92500301	112.552357	75.3	Farmer owned field
171030	*F. asiaticum*	3ADON	Hunan	Hunan	Rice	Rice	2017	27.92500301	112.552357	75.3	Farmer owned field
171042	*F. asiaticum*	NIV	Hunan	Hunan	Rice	Rice	2017	27.92500301	112.552357	75.3	Farmer owned field
171059	*F. asiaticum*	NIV	Hunan	Hunan	Rice	Rice	2017	27.699346	112.522798	101	Farmer owned field
171067	*F. asiaticum*	3ADON	Hunan	Hunan	Rice	Rice	2017	27.699346	112.522798	101	Farmer owned field
171075	*F. asiaticum*	3ADON	Hunan	Hunan	Rice	Rice	2017	27.699346	112.522798	101	Farmer owned field
171077	*F. asiaticum*	NIV	Hunan	Hunan	Rice	Rice	2017	27.699346	112.522798	101	Farmer owned field
171084	*F. asiaticum*	NIV	Hunan	Hunan	Rice	Rice	2017	27.52331899	112.595131	NA	Farmer owned field
171108	*F. asiaticum*	3ADON	Hunan	Hunan	Rice	Rice	2017	27.431337	112.6122	NA	Farmer owned field
171115	*F. asiaticum*	3ADON	Hunan	Hunan	Rice	Rice	2017	27.431337	112.6122	NA	Farmer owned field
171117	*F. asiaticum*	NIV	Hunan	Hunan	Rice	Rice	2017	27.431337	112.6122	NA	Farmer owned field
171120	*F. asiaticum*	NIV	Fujian	Fujian	Rice	Rice	2017	27.532357	118.080683	154.7	Farmer owned field
171130	*F. asiaticum*	15ADON	Fujian	Fujian	Rice	Rice	2017	27.532357	118.080683	154.7	Farmer owned field
171131	*F. asiaticum*	NIV	Fujian	Fujian	Rice	Rice	2017	27.532357	118.080683	154.7	Farmer owned field
171146	*F. asiaticum*	NIV	Fujian	Fujian	Rice	Rice	2017	27.532357	118.080683	154.7	Farmer owned field

171151	*F. asiaticum*	NIV	Fujian	Fujian	Rice	Rice	2017	27.516527	118.04489	156.4	Farmer owned field
171153	*F. asiaticum*	NIV	Fujian	Fujian	Rice	Rice	2017	27.516527	118.04489	156.4	Farmer owned field
171156	*F. asiaticum*	NIV	Fujian	Fujian	Rice	Rice	2017	27.516527	118.04489	156.4	Farmer owned field
171169	*F. asiaticum*	NIV	Fujian	Fujian	Rice	Rice	2017	27.516527	118.04489	156.4	Farmer owned field
171184	*F. asiaticum*	NIV	Fujian	Fujian	Rice	Rice	2017	28.01743	118.482725	313.6	Farmer owned field
171195	*F. asiaticum*	NIV	Fujian	Fujian	Rice	Rice	2017	28.01743	118.482725	313.6	Farmer owned field
171205	*F. asiaticum*	15ADON	Fujian	Fujian	Rice	Rice	2017	28.01743	118.482725	313.6	Farmer owned field
171206	*F. asiaticum*	NIV	Fujian	Fujian	Rice	Rice	2017	28.01743	118.482725	313.6	Farmer owned field
171207	*F. asiaticum*	NIV	Fujian	Fujian	Rice	Rice	2017	27.296453	117.34371	260.2	Farmer owned field
171210	*F. asiaticum*	NIV	Fujian	Fujian	Rice	Rice	2017	27.296453	117.34371	260.2	Farmer owned field
171212	*F. asiaticum*	15ADON	Fujian	Fujian	Rice	Rice	2017	27.296453	117.34371	260.2	Farmer owned field
171218	*F. asiaticum*	NIV	Fujian	Fujian	Rice	Rice	2017	27.296453	117.34371	260.2	Farmer owned field
171239	*F. asiaticum*	NIV	Fujian	Fujian	Rice	Rice	2017	27.296453	117.34371	260.2	Farmer owned field
171244	*F. asiaticum*	NIV	Fujian	Fujian	Rice	Rice	2017	26.842675	117.165943	327	Farmer owned field
171252	*F. asiaticum*	NIV	Fujian	Fujian	Rice	Rice	2017	26.842675	117.165943	327	Farmer owned field
171274	*F. asiaticum*	NIV	Fujian	Fujian	Rice	Rice	2017	26.842675	117.165943	327	Farmer owned field
171278	*F. asiaticum*	15ADON	Fujian	Fujian	Rice	Rice	2017	26.842675	117.165943	327	Farmer owned field
171287	*F. asiaticum*	NIV	Fujian	Fujian	Rice	Rice	2017	26.842675	117.196507	410.8	Farmer owned field
171290	*F. asiaticum*	NIV	Fujian	Fujian	Rice	Rice	2017	26.842675	117.196507	410.8	Farmer owned field
171301	*F. asiaticum*	NIV	Fujian	Fujian	Rice	Rice	2017	26.842675	117.196507	410.8	Farmer owned field
171308	*F. asiaticum*	15ADON	Fujian	Fujian	Rice	Rice	2017	26.842675	117.196507	410.8	Farmer owned field
171322	*F. asiaticum*	15ADON	Fujian	Fujian	Rice	Rice	2017	26.947053	117.227145	288	Farmer owned field
171323	*F. asiaticum*	NIV	Fujian	Fujian	Rice	Rice	2017	26.947053	117.227145	288	Farmer owned field
171330	*F. asiaticum*	NIV	Fujian	Fujian	Rice	Rice	2017	26.947053	117.227145	288	Farmer owned field
171338	*F. asiaticum*	NIV	Fujian	Fujian	Rice	Rice	2017	26.947053	117.227145	288	Farmer owned field
171349	*F. asiaticum*	NIV	Fujian	Fujian	Rice	Rice	2017	26.947053	117.227145	288	Farmer owned field
171366	*F. asiaticum*	NIV	Fujian	Fujian	Rice	Rice	2017	26.37813	117.866667	112.8	Farmer owned field
171369	*F. asiaticum*	NIV	Fujian	Fujian	Rice	Rice	2017	26.37813	117.866667	112.8	Farmer owned field
171375	*F. asiaticum*	NIV	Fujian	Fujian	Rice	Rice	2017	26.37813	117.866667	112.8	Farmer owned field
171377	*F. asiaticum*	NIV	Fujian	Fujian	Rice	Rice	2017	26.37813	117.866667	112.8	Farmer owned field
171383	*F. asiaticum*	NIV	Fujian	Fujian	Rice	Rice	2017	26.37813	117.866667	112.8	Farmer owned field
171465	*F. asiaticum*	NIV	Sichuan	Sichuan	Rice	Wheat-Rice	2017	30.710058	103.86309	517.4	Farmer owned field
171466	*F. asiaticum*	NIV	Sichuan	Sichuan	Rice	Wheat-Rice	2017	30.710058	103.86309	517.4	Farmer owned field
171467	*F. asiaticum*	NIV	Sichuan	Sichuan	Rice	Wheat-Rice	2017	30.710058	103.86309	517.4	Farmer owned field
171470	*F. asiaticum*	NIV	Sichuan	Sichuan	Rice	Wheat-Rice	2017	30.710058	103.86309	517.4	Farmer owned field

171471	*F. asiaticum*	NIV	Sichuan	Sichuan	Rice	Wheat-Rice	2017	30.710058	103.86309	517.4		Farmer owned field
171472	*F. asiaticum*	NIV	Sichuan	Sichuan	Rice	Wheat-Rice	2017	30.710058	103.86309	517.4		Farmer owned field
171475	*F. asiaticum*	NIV	Sichuan	Sichuan	Rice	Wheat-Rice	2017	30.710058	103.86309	517.4		Farmer owned field
171476	*F. asiaticum*	NIV	Sichuan	Sichuan	Rice	Wheat-Rice	2017	30.710058	103.86309	517.4		Farmer owned field
171477	*F. asiaticum*	NIV	Sichuan	Sichuan	Rice	Wheat-Rice	2017	30.710058	103.86309	517.4		Farmer owned field
171478	*F. asiaticum*	NIV	Sichuan	Sichuan	Rice	Wheat-Rice	2017	30.710058	103.86309	517.4		Farmer owned field
171481	*F. asiaticum*	NIV	Sichuan	Sichuan	Rice	Wheat-Rice	2017	30.710058	103.86309	517.4		Farmer owned field
171482	*F. asiaticum*	NIV	Sichuan	Sichuan	Rice	Wheat-Rice	2017	30.710058	103.86309	517.4		Farmer owned field
171483	*F. asiaticum*	NIV	Sichuan	Sichuan	Rice	Wheat-Rice	2017	30.710058	103.86309	517.4		Farmer owned field
171484	*F. asiaticum*	NIV	Sichuan	Sichuan	Rice	Wheat-Rice	2017	30.710058	103.86309	517.4		Farmer owned field
171639	*F. asiaticum*	NIV	Sichuan	Sichuan	Rice	Wheat-Rice	2017	29.618008	105.025732	305.3		Farmer owned field
171640	*F. asiaticum*	NIV	Sichuan	Sichuan	Rice	Wheat-Rice	2017	29.618008	105.025732	305.3		Farmer owned field
171642	*F. asiaticum*	NIV	Sichuan	Sichuan	Rice	Wheat-Rice	2017	29.618008	105.025732	305.3		Farmer owned field
171643	*F. asiaticum*	NIV	Sichuan	Sichuan	Rice	Wheat-Rice	2017	29.618008	105.025732	305.3		Farmer owned field
171644	*F. asiaticum*	NIV	Sichuan	Sichuan	Rice	Wheat-Rice	2017	29.618008	105.025732	305.3		Farmer owned field
171645	*F. asiaticum*	NIV	Sichuan	Sichuan	Rice	Wheat-Rice	2017	29.618008	105.025732	305.3		Farmer owned field
171646	*F. asiaticum*	NIV	Sichuan	Sichuan	Rice	Wheat-Rice	2017	29.618008	105.025732	305.3		Farmer owned field
171647	*F. asiaticum*	NIV	Sichuan	Sichuan	Rice	Wheat-Rice	2017	29.618008	105.025732	305.3		Farmer owned field
171651	*F. asiaticum*	NIV	Sichuan	Sichuan	Rice	Wheat-Rice	2017	29.618008	105.025732	305.3		Farmer owned field
171653	*F. asiaticum*	NIV	Sichuan	Sichuan	Rice	Wheat-Rice	2017	29.618008	105.025732	305.3		Farmer owned field
171658	*F. asiaticum*	NIV	Sichuan	Sichuan	Rice	Wheat-Rice	2017	29.618008	105.025732	305.3		Farmer owned field
171660	*F. asiaticum*	NIV	Sichuan	Sichuan	Rice	Wheat-Rice	2017	29.618008	105.025732	305.3		Farmer owned field
171663	*F. asiaticum*	NIV	Sichuan	Sichuan	Rice	Wheat-Rice	2017	29.618008	105.025732	305.3		Farmer owned field
171668	*F. asiaticum*	NIV	Sichuan	Sichuan	Rice	Wheat-Rice	2017	29.618008	105.025732	305.3		Farmer owned field
171669	*F. asiaticum*	NIV	Sichuan	Sichuan	Rice	Wheat-Rice	2017	29.618008	105.025732	305.3		Farmer owned field
171670	*F. asiaticum*	NIV	Sichuan	Sichuan	Rice	Wheat-Rice	2017	29.618008	105.025732	305.3		Farmer owned field
172114	*F. asiaticum*	NIV	Sichuan	Sichuan	Wheat	Wheat-Rice	2017	30.562533	103.657882	499.5	Commercialized variety	Farmer owned field
172117	*F. asiaticum*	NIV	Sichuan	Sichuan	Wheat	Wheat-Rice	2017	30.562533	103.657882	499.5	Commercialized variety	Farmer owned field
172118	*F. asiaticum*	NIV	Sichuan	Sichuan	Wheat	Wheat-Rice	2017	30.562533	103.657882	499.5	Commercialized variety	Farmer owned field
172119	*F. asiaticum*	NIV	Sichuan	Sichuan	Wheat	Wheat-Rice	2017	30.562533	103.657882	499.5	Commercialized variety	Farmer owned field
172120	*F. asiaticum*	NIV	Sichuan	Sichuan	Wheat	Wheat-Rice	2017	30.562533	103.657882	499.5	Commercialized variety	Farmer owned field
172122	*F. asiaticum*	NIV	Sichuan	Sichuan	Wheat	Wheat-Rice	2017	29.590293	104.975397	328	Commercialized variety	Farmer owned field
172123	*F. asiaticum*	NIV	Sichuan	Sichuan	Wheat	Wheat-Rice	2017	29.590293	104.975397	328	Commercialized variety	Farmer owned field
174231	*F. asiaticum*	NIV	Hunan	Hunan	Rice	Rice	2017	27.409341	109.658426	251		Farmer owned field
174240	*F. asiaticum*	NIV	Hunan	Hunan	Rice	Rice	2017	27.409341	109.658426	251		Farmer owned field

174251	*F. asiaticum*	NIV	Hunan	Hunan	Rice	Rice	2017	27.409341	109.658426	251		Farmer owned field
180096	*F. asiaticum*	NIV	MLYRP	Anhui	Wheat	Wheat-Rice	2018	NA	NA	NA	Commercialized variety	NA
180099	*F. asiaticum*	NIV	MLYRP	Anhui	Wheat	Wheat-Rice	2018	NA	NA	NA	Commercialized variety	NA
180100	*F. asiaticum*	3ADON	MLYRP	Anhui	Wheat	Wheat-Rice	2018	NA	NA	NA	Commercialized variety	NA
180101	*F. asiaticum*	3ADON	MLYRP	Anhui	Wheat	Wheat-Rice	2018	NA	NA	NA	Commercialized variety	NA
180102	*F. asiaticum*	3ADON	MLYRP	Anhui	Wheat	Wheat-Rice	2018	NA	NA	NA	Commercialized variety	NA
180103	*F. asiaticum*	3ADON	MLYRP	Anhui	Wheat	Wheat-Rice	2018	NA	NA	NA	Commercialized variety	NA
180104	*F. asiaticum*	3ADON	MLYRP	Anhui	Wheat	Wheat-Rice	2018	NA	NA	NA	Commercialized variety	NA
180105	*F. asiaticum*	3ADON	MLYRP	Anhui	Wheat	Wheat-Rice	2018	NA	NA	NA	Commercialized variety	NA
180106	*F. asiaticum*	3ADON	MLYRP	Anhui	Wheat	Wheat-Rice	2018	NA	NA	NA	Commercialized variety	NA
180123	*F. asiaticum*	3ADON	MLYRP	Hubei	Wheat	Wheat-Rice	2018	NA	NA	NA	Commercialized variety	NA
180188	*F. asiaticum*	3ADON	MLYRP	Jiangsu	Wheat	Wheat-Rice	2018	NA	NA	NA	Commercialized variety	NA
180192	*F. asiaticum*	3ADON	MLYRP	Jiangsu	Wheat	Wheat-Rice	2018	NA	NA	NA	Commercialized variety	NA
180194	*F. asiaticum*	3ADON	MLYRP	Jiangsu	Wheat	Wheat-Rice	2018	NA	NA	NA	Commercialized variety	NA
180197	*F. asiaticum*	3ADON	MLYRP	Jiangsu	Wheat	Wheat-Rice	2018	NA	NA	NA	Commercialized variety	NA
180200	*F. asiaticum*	3ADON	MLYRP	Jiangsu	Wheat	Wheat-Rice	2018	NA	NA	NA	Commercialized variety	NA
180203	*F. asiaticum*	3ADON	MLYRP	Jiangsu	Wheat	Wheat-Rice	2018	NA	NA	NA	Commercialized variety	NA
180207	*F. asiaticum*	3ADON	MLYRP	Jiangsu	Wheat	Wheat-Rice	2018	NA	NA	NA	Commercialized variety	NA
180212	*F. asiaticum*	3ADON	MLYRP	Jiangsu	Wheat	Wheat-Rice	2018	NA	NA	NA	Commercialized variety	NA
180216	*F. asiaticum*	3ADON	MLYRP	Jiangsu	Wheat	Wheat-Rice	2018	NA	NA	NA	Commercialized variety	NA
180219	*F. asiaticum*	3ADON	MLYRP	Jiangsu	Wheat	Wheat-Rice	2018	NA	NA	NA	Commercialized variety	NA
180220	*F. asiaticum*	NIV	Sichuan	Sichuan	Wheat	Wheat-Rice	2018	NA	NA	NA	Commercialized variety	Farmer owned field
180221	*F. asiaticum*	NIV	Sichuan	Sichuan	Wheat	Wheat-Rice	2018	NA	NA	NA	Commercialized variety	Farmer owned field
180222	*F. asiaticum*	NIV	Sichuan	Sichuan	Wheat	Wheat-Rice	2018	NA	NA	NA	Commercialized variety	Farmer owned field
180242	*F. asiaticum*	NIV	Sichuan	Sichuan	Wheat	Wheat-Rice	2018	NA	NA	NA	Commercialized variety	Farmer owned field
180243	*F. asiaticum*	NIV	Sichuan	Sichuan	Wheat	Wheat-Rice	2018	NA	NA	NA	Commercialized variety	Farmer owned field
180246	*F. asiaticum*	NIV	Sichuan	Sichuan	Wheat	Wheat-Rice	2018	NA	NA	NA	Commercialized variety	Farmer owned field
180249	*F. asiaticum*	NIV	Sichuan	Sichuan	Wheat	Wheat-Rice	2018	NA	NA	NA	Commercialized variety	Farmer owned field
180250	*F. asiaticum*	NIV	Sichuan	Sichuan	Wheat	Wheat-Rice	2018	NA	NA	NA	Commercialized variety	Farmer owned field
180265	*F. asiaticum*	NIV	Sichuan	Sichuan	Wheat	Wheat-Rice	2018	NA	NA	NA	Commercialized variety	Farmer owned field
180267	*F. asiaticum*	NIV	Sichuan	Sichuan	Wheat	Wheat-Rice	2018	NA	NA	NA	Commercialized variety	Farmer owned field
180269	*F. asiaticum*	NIV	Sichuan	Sichuan	Wheat	Wheat-Rice	2018	NA	NA	NA	Commercialized variety	Farmer owned field
180271	*F. asiaticum*	NIV	Sichuan	Sichuan	Wheat	Wheat-Rice	2018	NA	NA	NA	Commercialized variety	Farmer owned field
180273	*F. asiaticum*	NIV	Sichuan	Sichuan	Wheat	Wheat-Rice	2018	NA	NA	NA	Commercialized variety	Farmer owned field
180275	*F. asiaticum*	NIV	Sichuan	Sichuan	Wheat	Wheat-Rice	2018	NA	NA	NA	Commercialized variety	Farmer owned field

180277	*F. asiaticum*	NIV	Sichuan	Sichuan	Wheat	Wheat-Rice	2018	NA	NA	NA	Commercialized variety	Farmer owned field
180279	*F. asiaticum*	NIV	Sichuan	Sichuan	Wheat	Wheat-Rice	2018	NA	NA	NA	Commercialized variety	Farmer owned field
180281	*F. asiaticum*	NIV	Sichuan	Sichuan	Wheat	Wheat-Rice	2018	NA	NA	NA	Commercialized variety	Farmer owned field
180282	*F. asiaticum*	NIV	Sichuan	Sichuan	Wheat	Wheat-Rice	2018	NA	NA	NA	Commercialized variety	Farmer owned field
180283	*F. asiaticum*	NIV	Sichuan	Sichuan	Wheat	Wheat-Rice	2018	NA	NA	NA	Commercialized variety	Farmer owned field
180288	*F. asiaticum*	3ADON	MLYRP	Hubei	Rice	Wheat-Rice	2018	30.56438897	114.917469	24.37772		Commercial farm
180341	*F. asiaticum*	3ADON	MLYRP	Hubei	Rice	Wheat-Rice	2018	29.989779	114.76211	72.181686		Farmer owned field
180363	*F. asiaticum*	3ADON	MLYRP	Hubei	Rice	Wheat-Rice	2018	29.90943502	114.504874	44.087009		Farmer owned field
180388	*F. asiaticum*	3ADON	MLYRP	Hubei	Rice	Wheat-Rice	2018	30.93555498	113.873914	27.244297		Farmer owned field
180432	*F. asiaticum*	3ADON	MLYRP	Hubei	Rice	Wheat-Rice	2018	30.93952197	113.853011	31.46199		Farmer owned field
180459	*F. asiaticum*	3ADON	MLYRP	Hubei	Rice	Wheat-Rice	2018	30.89166398	113.588332	28.714087		Farmer owned field
180648	*F. asiaticum*	NIV	MLYRP	Hubei	Wheat	Wheat-Rice	2018	30.04730299	112.153793	35.333096	Commercialized variety	Farmer owned field
181006	*F. asiaticum*	15ADON	MLYRP	Hubei	Wheat	Wheat-Rice	2018	NA	NA	NA	Commercialized variety	NA
181028	*F. asiaticum*	NIV	MLYRP	Hubei	Wheat	Wheat-Rice	2018	30.56438897	114.917469	24.37772	Commercialized variety	Commercial farm
181043	*F. asiaticum*	NIV	MLYRP	Hubei	Wheat	Wheat-Rice	2018	30.63488597	113.115741	31.271826	Commercialized variety	Farmer owned field
181054	*F. asiaticum*	15ADON	MLYRP	Hubei	Wheat	Wheat-Rice	2018	30.63488597	113.115741	31.271826	Commercialized variety	Farmer owned field
181076	*F. asiaticum*	NIV	MLYRP	Hubei	Wheat	Wheat-Rice	2018	30.86147396	112.867559	68.377945	Commercialized variety	Commercial farm
181084	*F. asiaticum*	NIV	MLYRP	Hubei	Wheat	Wheat-Rice	2018	30.86147396	112.867559	68.377945	Commercialized variety	Commercial farm
181096	*F. asiaticum*	NIV	MLYRP	Hubei	Wheat	Wheat-Rice	2018	30.86147396	112.867559	68.377945	Commercialized variety	Commercial farm
181100	*F. asiaticum*	NIV	MLYRP	Hubei	Wheat	Wheat-Rice	2018	30.36086798	112.058081	27.621157	Commercialized variety	Farmer owned field
181104	*F. asiaticum*	15ADON	MLYRP	Hubei	Wheat	Wheat-Rice	2018	30.36086798	112.058081	27.621157	Commercialized variety	Farmer owned field
181126	*F. asiaticum*	NIV	MLYRP	Hubei	Wheat	Wheat-Rice	2018	31.04326101	112.282253	86.576973	Commercialized variety	Farmer owned field
181150	*F. asiaticum*	15ADON	MLYRP	Hubei	Wheat	Wheat-Rice	2018	31.88455699	112.030749	87.801369	Commercialized variety	Farmer owned field
182625	*F. asiaticum*	NIV	MLYRP	Jiangsu	Wheat	Wheat-Rice	2018	33.12189102	118.343823	47.342438	Commercialized variety	Farmer owned field
182647	*F. asiaticum*	NIV	MLYRP	Jiangsu	Wheat	Wheat-Rice	2018	33.33480301	119.988529	13.119117	Commercialized variety	Farmer owned field
191317	*F. asiaticum*	NIV	Fujian	Fujian	Rice	Rice	2018	26.842675	117.196507	410.8		Farmer owned field
191882	*F. asiaticum*	15ADON	MLYRP	Hubei	Wheat	Wheat-Rice	2019	30.06588198	114.882799	26.362423	Commercialized variety	Farmer owned field
192019	*F. asiaticum*	15ADON	MLYRP	Hubei	Wheat	Wheat-Rice	2019	31.59498998	113.318392	139.44841	Commercialized variety	Farmer owned field
193955	*F. asiaticum*	NIV	MLYRP	Jiangsu	Rice	Wheat-Rice	2019	28.62087197	112.339678	77.464882		Farmer owned field
193959	*F. asiaticum*	NIV	MLYRP	Jiangsu	Rice	Wheat-Rice	2019	28.62087197	112.339678	77.464882		Farmer owned field
193963	*F. asiaticum*	NIV	MLYRP	Jiangsu	Rice	Wheat-Rice	2019	28.62087197	112.339678	77.464882		Farmer owned field
193966	*F. asiaticum*	NIV	MLYRP	Jiangsu	Rice	Wheat-Rice	2019	28.62087197	112.339678	77.464882		Farmer owned field
193968	*F. asiaticum*	NIV	MLYRP	Jiangsu	Rice	Wheat-Rice	2019	28.62087197	112.339678	77.464882		Farmer owned field
194004	*F. asiaticum*	NIV	MLYRP	Anhui	Rice	Wheat-Rice	2019	30.19380701	118.865659	650.646667		Farmer owned field
194006	*F. asiaticum*	NIV	MLYRP	Anhui	Rice	Wheat-Rice	2019	30.19380701	118.865659	650.646667		Farmer owned field

194008	*F. asiaticum*	NIV	MLYRP	Anhui	Rice	Wheat-Rice	2019	30.19380701	118.865659	650.646667	Farmer owned field
194010	*F. asiaticum*	NIV	MLYRP	Anhui	Rice	Wheat-Rice	2019	30.19380701	118.865659	650.646667	Farmer owned field
194012	*F. asiaticum*	NIV	MLYRP	Anhui	Rice	Wheat-Rice	2019	30.19380701	118.865659	650.646667	Farmer owned field

Table S2 Pangenome homology groups identified by PanTools.

#grouping_v5

[illegible]

46872281 Fasi171011_G01221.t1#85 Fasi171206_G01217.t1#110 Fasi170880_G01222.t1#74 Fasi171476_G01224.t1#141 Fasi170542_G01210.t1#52 Fasi171075_G01210.t1#93 Fasi170824_G01218.t1#70 Fasi170765_G01199.t1#64 Fasi140002_G00803.t1#2 Fasi140021_G01204.t1#21 Fasi170706_G01189.t1#60 Fasi160264_G01221.t1#34 Fasi180105_G01221.t1#181 Fasi140009_G01214.t1#9 Fasi160282_G01208.t1#38 Fasi170383_G01448.t1#47 Fasi170784_G01213.t1#66 Fasi170372_G01220.t1#45 [illegible]
[illegible]

46874061 Fasi140019_G02828.t1#19 Fasi160205_G02825.t1#29 Fasi171153_G02890.t1#104 Fasi171244_G02853.t1#116 Fasi140052_G02836.t1#28 Fasi170364_G02830.t1#44 Fasi180281_G02970.t1#210 Fasi171481_G03147.t1#144 Fasi180104_G03239.t1#180 Fasi171349_G02824.t1#128 Fasi140005_G02325.t1#5 Fasi170705_G03194.t1#59 Fasi171470_G02815.t1#137 Fasi170767_G02826.t1#65 Fasi180192_G02385.t1#185 Fasi170944_G02815.t1#78 Fasi171025_G02848.t1#87 Fasi140023_G02969.t1#23 [illegible]
46874062 Fasi170888_G02841.t1#75 Fasi180267_G02740.t1#203 Fasi170792_G02205.t1#69 Fasi181076_G02835.t1#224 Fasi180216_G02831.t1#192 Fasi170788_G02836.t1#67 Fasi171466_G02972.t1#135 Fasi182625_G02828.t1#231 Fasi171383_G02835.t1#133 Fasi180246_G03290.t1#199 Fasi170908_G03320.t1#77 Fasi170431_G02817.t1#49 Fasi171195_G02848.t1#108 Fasi170347_G02826.t1#43 Fasi171644_G02823.t1#152 Fasi180281_G02971.t1#210 Fasi171108_G02835.t1#96 Fasi180200_G02836.t1#188 [illegible]
46874063 Fasi172123_G02832.t1#170 Fasi170430_G02831.t1#48 Fasi181006_G02837.t1#220 Fasi171156_G02905.t1#105 Fasi170945_G03026.t1#79 Fasi171483_G02851.t1#146 Fasi171210_G02826.t1#112 Fasi180212_G02830.t1#191 Fasi140002_G02656.t1#2 Fasi171212_G02853.t1#113 Fasi171476_G02836.t1#141 Fasi180269_G02487.t1#204 Fasi171669_G02838.t1#162 Fasi160264_G02851.t1#34 Fasi172119_G03058.t1#167 Fasi171651_G02577.t1#156 Fasi171084_G02835.t1#95 Fasi180282_G02827.t1#211 [illegible]
[illegible]

46875841 Fasi171274_G04384.t1#118 Fasi171472_G04481.t1#139 Fasi180250_G04540.t1#201 Fasi191882_G04190.t1#234 Fasi160277_G04594.t1#36 Fasi171481_G04416.t1#144 Fasi171108_G04440.t1#96 Fasi172120_G04293.t1#168 [illegible]
46875842 Fasi171349_G04409.t1#128 Fasi160242_G04480.t1#33 Fasi140026_G07343.t1#26 Fasi171669_G04421.t1#162 Fasi180100_G06647.t1#176 [illegible]
[illegible]

46877620 Fasi171475_G05739.t1#140 Fasi171646_G06029.t1#154 Fasi171029_G06054.t1#88 [illegible]
46877621 Fasi192019_G06429.t1#235 Fasi170867_G06421.t1#72 [illegible]
46877622 Fasi171663_G05988.t1#160 [illegible]
46877623 Fasi171210_G06027.t1#112 [illegible]
46877624 Fasi170372_G06071.t1#45 [illegible]
46877625 Fasi170367_G06099.t1#92 [illegible]
[illegible]

[illegible]

46881179 Fasi170343_G09514.t1#42 Fasi171195_G09410.t1#108 Fasi140023_G09555.t1#23 Fasi180288_G09405.t1#213 Fasi170383_G09657.t1#47 Fasi160277_G09431.t1#36 Fasi171640_G09383.t1#149 Fasi140004_G09622.t1#17 [illegible]
46881180 Fasi160952_G09374.t1#41 Fasi180222_G09435.t1#196 Fasi180432_G09486.t1#217 [illegible]
46881181 Fasi170460_G09564.t1#50 [illegible]
46881182 Fasi140001_G09479.t1#1 [illegible]
46881183 Fasi140022_G09426.t1#22 [illegible]
[illegible]

[illegible]

46884739 Fasi171130_G12839.t1#100 Fasi170545_G12408.t1#53 Fasi180243_G12819.t1#198 Fasi180275_G12855.t1#207 Fasi171383_G12832.t1#133 Fasi180212_G12843.t1#191 Fasi171077_G12912.t1#94 [illegible]
46884740 Fasi180271_G12970.t1#205 Fasi171244_G12920.t1#116 Fasi174240_G12898.t1#172 Fasi194009_G12918.t1#243 Fasi160270_G12893.t1#35 Fasi171476_G12841.t1#141 [illegible]
46884741 Fasi194010_G12876.t1#244 Fasi140026_G12847.t1#26 Fasi172123_G12916.t1#170 Fasi140020_G12847.t1#20 Fasi170542_G12943.t1#52 [illegible]
46884742 Fasi170908_G13117.t1#77 Fasi171322_G12885.t1#124 Fasi171005_G12942.t1#84 Fasi170792_G12844.t1#69 Fasi170460_G13069.t1#50 [illegible]
46884743 Fasi180188_G12871.t1#184 Fasi180279_G12936.t1#209 Fasi170944_G12836.t1#78 Fasi140020_G12849.t1#20 [illegible]
46884744 Fasi181100_G12870.t1#227 Fasi171484_G12881.t1#147 Fasi174251_G12887.t1#173 Fasi180200_G12919.t1#188 [illegible]
46884745 Fasi171475_G12855.t1#140 Fasi180341_G12923.t1#214 Fasi140012_G12863.t1#12 Fasi193966_G12908.t1#239 [illegible]
46884746 Fasi180388_G12823.t1#216
[illegible]

46886519 Fasi170908_G13525.t1#77 Fasi171330_G13488.t1#126 Fasi170542_G10514.t1#52 Fasi174240_G13278.t1#172 Fasi170460_G07664.t1#50 Fasi170677_G10066.t1#58 Fasi170988_G13489.t1#82 Fasi170464_G13442.t1#51 Fasi170880_G13377.t1#74 Fasi140025_G10523.t1#25
46886520 Fasi140025_G10527.t1#25 Fasi171330_G13492.t1#126 Fasi170677_G10070.t1#58 Fasi170988_G13477.t1#82 Fasi174240_G13273.t1#172 Fasi170880_G13373.t1#74 Fasi170460_G13546.t1#50 Fasi170908_G13501.t1#77 Fasi170464_G13446.t1#51 Fasi170542_G10517.t1#52
46886521 Fasi170908_G13502.t1#77 Fasi170460_G13547.t1#50 Fasi170880_G13374.t1#74 Fasi171330_G13491.t1#126 Fasi170464_G13445.t1#51 Fasi170677_G10069.t1#58 Fasi170542_G10516.t1#52 Fasi140025_G10526.t1#25 Fasi174240_G13274.t1#172 Fasi170988_G13476.t1#82
46886522 Fasi171330_G13495.t1#126 Fasi140025_G10530.t1#25 Fasi170908_G13497.t1#77 Fasi170988_G13481.t1#82 Fasi174240_G13269.t1#172
46886523 Fasi170542_G10520.t1#52 Fasi170908_G13560.t1#77 Fasi170460_G13542.t1#50 Fasi170988_G13551.t1#82 Fasi140025_G13572.t1#25 Fasi170677_G10073.t1#58 Fasi170464_G13448.t1#51
46886524 Fasi140025_G10531.t1#25 Fasi171330_G13496.t1#126 Fasi170880_G13369.t1#74 Fasi174240_G13268.t1#172
46886525 Fasi170542_G13315.t1#52 Fasi140025_G13393.t1#25 Fasi174240_G13258.t1#172
46886526 Fasi171330_G13518.t1#126 Fasi174240_G13353.t1#172
46886527 Fasi170464_G13384.t1#51 Fasi171330_G13378.t1#126
46886528 Fasi170460_G13532.t1#50 Fasi140025_G10533.t1#25 Fasi170542_G13437.t1#52 Fasi170988_G13405.t1#82 Fasi170677_G10081.t1#58 Fasi170880_G13461.t1#74 Fasi170464_G13530.t1#51
46886529 Fasi170908_G13532.t1#77 Fasi170460_G13531.t1#50 Fasi170988_G13404.t1#82 Fasi170880_G13462.t1#74 Fasi140025_G10532.t1#25 Fasi170464_G13529.t1#51
46886530 Fasi170988_G13363.t1#82 Fasi170880_G13270.t1#74 Fasi170677_G07091.t1#58 Fasi170542_G07858.t1#52 Fasi170464_G13616.t1#51 Fasi171330_G13343.t1#126
46886531 Fasi170464_G13528.t1#51 Fasi171330_G13310.t1#126 Fasi170677_G10080.t1#58 Fasi170542_G13436.t1#52
46886532 Fasi170542_G07859.t1#52 Fasi170464_G13615.t1#51 Fasi171330_G13344.t1#126 Fasi170908_G13327.t1#77
46886533 Fasi140025_G13391.t1#25 Fasi170460_G13490.t1#50 Fasi170988_G13362.t1#82
46886534 Fasi174240_G13304.t1#172 Fasi170460_G13491.t1#50
46886535 Fasi171330_G13383.t1#126 Fasi174240_G13303.t1#172
46886536 Fasi170988_G13406.t1#82 Fasi170460_G13533.t1#50 Fasi170908_G13533.t1#77 Fasi140025_G10534.t1#25 Fasi170880_G13460.t1#74
46886537 Fasi170988_G13407.t1#82 Fasi170908_G13534.t1#77 Fasi170464_G13531.t1#51 Fasi140025_G10535.t1#25 Fasi170542_G13438.t1#52 Fasi170460_G13534.t1#50 Fasi170880_G13459.t1#74 Fasi170677_G10082.t1#58
46886538 Fasi170908_G13535.t1#77 Fasi170880_G13458.t1#74 Fasi140025_G10536.t1#25 Fasi170988_G13408.t1#82 Fasi170677_G10083.t1#58 Fasi170464_G13532.t1#51 Fasi171330_G13311.t1#126 Fasi170542_G13439.t1#52 Fasi170460_G13535.t1#50
46886539 Fasi171059_G10451.t1#91 Fasi171030_G10487.t1#89 Fasi171146_G10464.t1#102 Fasi171077_G10517.t1#94 Fasi171301_G10439.t1#122 Fasi171647_G10583.t1#155 Fasi170789_G10588.t1#68 Fasi170383_G10698.t1#47 Fasi171668_G10466.t1#161 Fasi171646_G10345.t1#154 Fasi193966_G10496.t1#239 Fasi172123_G10388.t1#170 Fasi140026_G10498.t1#26 Fasi171470_G10445.t1#137 Fasi181006_G10461.t1#220 Fasi171323_G10608.t1#125 Fasi193963_G10399.t1#238 Fasi170991_G10320.t1#83 [illegible]
46886540 Fasi170372_G10502.t1#45 Fasi171670_G10537.t1#163 Fasi171151_G10415.t1#103 Fasi171153_G10442.t1#104 Fasi172114_G10439.t1#164 Fasi171278_G10384.t1#119 Fasi171323_G10609.t1#125 Fasi171471_G10604.t1#138 Fasi171290_G10904.t1#121 Fasi140027_G10502.t1#27 Fasi171115_G10508.t1#97 Fasi170464_G10594.t1#51 Fasi171084_G10517.t1#95 Fasi170383_G10699.t1#47 Fasi181126_G10517.t1#229 Fasi192019_G10407.t1#235 Fasi171030_G10488.t1#89 Fasi170789_G10589.t1#68 [illegible]
46886541 Fasi171120_G10617.t1#99 Fasi140025_G10576.t1#25 Fasi193966_G10500.t1#239 Fasi180273_G10565.t1#206 Fasi180249_G10003.t1#200 Fasi180269_G10448.t1#204 Fasi171005_G10528.t1#84 Fasi171663_G10466.t1#160 Fasi170991_G10324.t1#83 Fasi181006_G10465.t1#220 Fasi170945_G10416.t1#79 Fasi193955_G10429.t1#236 Fasi171668_G10470.t1#161 Fasi171059_G10455.t1#91 Fasi193968_G10519.t1#240 Fasi181126_G10520.t1#229 Fasi181054_G10416.t1#223 Fasi170383_G10702.t1#47 [illegible]
46886542 Fasi170383_G10700.t1#47 Fasi180271_G10548.t1#205 Fasi193966_G10498.t1#239 Fasi174231_G02948.t1#171 Fasi170988_G10488.t1#82 Fasi171195_G10492.t1#108 Fasi140027_G10503.t1#27 Fasi193963_G10401.t1#238 Fasi171481_G10470.t1#144 Fasi171668_G10468.t1#161 Fasi193968_G10517.t1#240 Fasi180277_G09650.t1#208 Fasi170464_G10595.t1#51 Fasi180250_G10580.t1#201 Fasi171642_G10644.t1#150 Fasi140025_G10574.t1#25 Fasi171645_G10496.t1#153 Fasi194010_G10479.t1#244 [illegible]
46886543 Fasi170566_G10530.t1#54 Fasi170789_G10593.t1#68 Fasi172114_G10443.t1#164 Fasi180249_G10004.t1#200 Fasi171668_G10471.t1#161 Fasi170372_G10506.t1#45 Fasi194010_G10482.t1#244 Fasi170945_G10417.t1#79 Fasi172117_G10515.t1#165 Fasi171117_G10492.t1#98 Fasi171646_G10350.t1#154 Fasi181043_G10964.t1#222 Fasi171375_G10483.t1#131 Fasi171084_G10521.t1#95 Fasi171481_G10473.t1#144 Fasi180250_G10583.t1#201 Fasi171115_G10512.t1#97 Fasi171642_G10647.t1#150 [illegible]
46886544 Fasi171218_G10737.t1#114 Fasi193968_G10744.t1#240 Fasi140026_G10726.t1#26 Fasi172123_G10616.t1#170 Fasi170988_G10718.t1#82 Fasi171660_G10683.t1#159 Fasi171377_G10590.t1#132 Fasi171476_G10669.t1#141 Fasi180648_G10710.t1#219 Fasi160898_G10901.t1#40 Fasi171153_G10666.t1#104 Fasi140025_G10801.t1#25 Fasi170867_G10621.t1#72
46886545 Fasi171668_G10472.t1#161 Fasi193963_G10405.t1#238 Fasi171278_G10389.t1#119 Fasi171239_G09706.t1#115 Fasi170566_G10531.t1#54 Fasi171349_G10390.t1#128 Fasi170988_G10492.t1#82 Fasi171153_G10447.t1#104 Fasi193968_G10521.t1#240 Fasi180273_G10567.t1#206 Fasi171290_G10909.t1#121 Fasi171011_G09969.t1#85 Fasi170888_G10496.t1#75 Fasi171481_G10474.t1#144 Fasi171195_G10496.t1#108 Fasi171030_G10493.t1#89 Fasi171274_G10215.t1#118 Fasi194008_G10520.t1#243 [illegible]
46886546 Fasi140025_G11803.t1#25 Fasi174240_G13345.t1#172 Fasi170988_G11787.t1#82 Fasi171330_G13636.t1#126
46886547 Fasi171330_G13443.t1#126 Fasi140025_G11825.t1#25
46886548 Fasi170542_G13290.t1#52 Fasi170677_G13632.t1#58 Fasi170988_G11789.t1#82 Fasi170880_G13544.t1#74 Fasi170464_G13613.t1#51 Fasi171330_G13638.t1#126 Fasi170460_G13647.t1#50 Fasi140025_G11805.t1#25
46886549 Fasi171330_G07104.t1#126 Fasi140025_G11807.t1#25
46886550 Fasi170880_G07588.t1#74 Fasi170908_G13395.t1#77 Fasi140025_G11808.t1#25 Fasi170460_G07193.t1#50 Fasi170464_G13479.t1#51 Fasi170988_G07574.t1#82 Fasi171330_G07103.t1#126 Fasi170677_G13453.t1#58 Fasi170542_G07340.t1#52
46886551 Fasi140025_G13526.t1#25 Fasi170908_G13475.t1#77 Fasi170880_G13338.t1#74 Fasi170542_G13443.t1#52 Fasi171330_G12770.t1#126 Fasi170460_G13585.t1#50 Fasi170677_G13489.t1#58 Fasi170464_G13538.t1#51 Fasi170988_G13398.t1#82
46886552 Fasi140025_G13410.t1#25 Fasi170908_G13355.t1#77 Fasi171330_G13417.t1#126 Fasi170988_G13324.t1#82 Fasi170880_G13324.t1#74 Fasi170677_G13432.t1#58
46886553 Fasi170460_G07191.t1#50 Fasi170464_G13477.t1#51 Fasi140025_G11810.t1#25 Fasi170677_G13451.t1#58 Fasi170908_G13393.t1#77 Fasi170542_G07338.t1#52 Fasi170880_G07590.t1#74 Fasi171330_G07101.t1#126 Fasi170988_G13505.t1#82
46886554 Fasi170677_G13449.t1#58 Fasi140025_G11812.t1#25 Fasi170542_G07336.t1#52 Fasi170988_G13507.t1#82 Fasi170464_G13475.t1#51 Fasi171330_G07099.t1#126 Fasi170880_G07592.t1#74 Fasi170460_G07189.t1#50 Fasi170908_G13391.t1#77
46886555 Fasi170677_G13447.t1#58 Fasi170988_G13314.t1#82 Fasi170908_G13389.t1#77 Fasi170542_G13386.t1#52 Fasi170464_G13493.t1#51 Fasi171330_G13453.t1#126 Fasi170880_G10453.t1#74 Fasi170460_G13474.t1#50 Fasi140025_G11814.t1#25
46886556 Fasi140025_G11816.t1#25 Fasi171330_G13451.t1#126 Fasi170908_G13387.t1#77 Fasi170542_G13388.t1#52 Fasi170880_G10455.t1#74 Fasi170988_G13316.t1#82 Fasi170677_G13445.t1#58 Fasi170464_G13495.t1#51 Fasi170460_G13476.t1#50
46886557 Fasi170908_G13386.t1#77 Fasi170880_G10456.t1#74 Fasi140025_G11817.t1#25 Fasi171330_G13450.t1#126 Fasi170460_G13477.t1#50 Fasi170464_G13496.t1#51 Fasi170988_G13317.t1#82 Fasi170677_G13444.t1#58 Fasi170542_G13389.t1#52
46886558 Fasi170988_G13320.t1#82 Fasi170464_G13497.t1#51 Fasi170542_G13390.t1#52 Fasi170908_G13384.t1#77 Fasi140025_G11820.t1#25 Fasi170880_G10459.t1#74 Fasi170460_G13478.t1#50 Fasi171330_G13449.t1#126
46886559 Fasi170460_G13480.t1#50 Fasi170464_G13499.t1#51 Fasi170880_G10462.t1#74 Fasi171330_G13446.t1#126 Fasi140025_G11822.t1#25
46886560 Fasi170677_G13443.t1#58 Fasi140025_G11821.t1#25 Fasi170988_G13322.t1#82 Fasi170542_G13391.t1#52 Fasi170460_G13479.t1#50 Fasi171330_G13447.t1#126 Fasi170880_G10461.t1#74 Fasi170908_G13382.t1#77 Fasi170464_G13498.t1#51
46886561 Fasi170677_G07111.t1#58 Fasi170988_G13324.t1#82 Fasi170908_G13555.t1#77 Fasi170880_G10464.t1#74 Fasi171330_G13442.t1#126 Fasi170460_G11903.t1#50 Fasi140025_G11826.t1#25 Fasi170464_G07660.t1#51 Fasi170542_G07921.t1#52
46886562 Fasi140025_G11823.t1#25 Fasi170988_G13323.t1#82 Fasi170677_G13442.t1#58 Fasi171330_G13445.t1#126 Fasi170542_G13392.t1#52 Fasi170908_G13552.t1#77 Fasi170460_G13481.t1#50 Fasi170464_G13500.t1#51 Fasi170880_G10463.t1#74
46886563 Fasi170542_G13383.t1#52 Fasi170677_G13520.t1#58 Fasi170464_G11896.t1#51 Fasi170908_G13403.t1#77 Fasi170880_G12221.t1#74 Fasi171330_G13441.t1#126 Fasi170460_G13472.t1#50 Fasi140025_G11829.t1#25 Fasi174240_G13335.t1#172
46886564 Fasi170677_G13519.t1#58 Fasi170908_G13402.t1#77 Fasi170988_G12093.t1#82 Fasi170464_G11897.t1#51 Fasi174240_G13336.t1#172 Fasi170460_G13471.t1#50 Fasi140025_G11830.t1#25 Fasi171330_G13440.t1#126 Fasi170880_G12222.t1#74 Fasi170542_G13382.t1#52
46886565 Fasi170464_G11901.t1#51 Fasi140025_G11834.t1#25 Fasi170880_G12226.t1#74 Fasi170677_G13516.t1#58 Fasi171330_G13437.t1#126 Fasi170908_G13398.t1#77 Fasi170988_G12097.t1#82 Fasi170460_G13468.t1#50 Fasi170542_G13379.t1#52 Fasi174240_G13340.t1#172
46886566 Fasi180277_G11010.t1#208 Fasi193955_G11782.t1#236 Fasi171663_G11823.t1#160 Fasi171084_G11836.t1#95 Fasi194010_G11839.t1#244 Fasi170888_G11870.t1#75 Fasi181126_G11887.t1#229 Fasi180281_G11817.t1#210 Fasi140025_G11975.t1#25 Fasi172114_G11796.t1#164 Fasi180279_G11885.t1#209 Fasi171470_G11798.t1#137 Fasi171647_G11955.t1#155 Fasi140027_G11871.t1#27 Fasi193968_G11894.t1#240 Fasi171042_G11755.t1#90 Fasi171077_G11886.t1#94 Fasi180246_G11834.t1#199
46886567 Fasi171651_G12936.t1#156 Fasi181100_G12946.t1#227 Fasi170566_G13020.t1#54 Fasi171642_G12969.t1#150 Fasi171130_G12923.t1#100 Fasi180279_G13016.t1#209 Fasi140025_G13099.t1#25 Fasi181043_G12972.t1#222 Fasi191317_G13035.t1#233 Fasi140026_G13002.t1#26 Fasi171322_G12963.t1#124 Fasi193955_G12899.t1#236 Fasi171375_G12963.t1#131 Fasi171308_G13010.t1#123 Fasi171205_G12956.t1#109 Fasi171369_G12954.t1#130 Fasi171323_G12973.t1#125 Fasi171207_G12950.t1#111 [illegible]
46886568 Fasi140026_G13003.t1#26 Fasi171042_G12911.t1#90 Fasi181100_G12947.t1#227 Fasi170677_G13130.t1#58 Fasi140052_G12983.t1#28 Fasi171642_G12970.t1#150 Fasi180279_G13017.t1#209 Fasi171287_G12993.t1#120 Fasi171308_G13011.t1#123 Fasi171205_G12957.t1#109 Fasi171646_G12990.t1#154 Fasi193955_G12900.t1#236 Fasi140025_G13100.t1#25 Fasi180281_G12948.t1#210 Fasi171278_G12861.t1#119 Fasi171323_G12974.t1#125 Fasi171322_G12964.t1#124 Fasi171653_G12988.t1#157 [illegible]
46886569 Fasi193955_G12901.t1#236 Fasi170888_G12999.t1#75 Fasi171207_G12952.t1#111 Fasi171210_G12922.t1#112 Fasi171369_G12956.t1#130 Fasi170566_G13022.t1#54 Fasi140025_G13101.t1#25 Fasi171322_G12965.t1#124 Fasi180246_G12974.t1#199 Fasi171131_G13000.t1#101 Fasi171212_G12928.t1#113 Fasi171290_G04227.t1#121 Fasi174240_G12983.t1#172 Fasi171308_G13012.t1#123 Fasi194006_G12588.t1#242 Fasi194008_G13002.t1#243 Fasi140026_G13004.t1#26 Fasi181043_G12974.t1#222 [illegible]
46886570 Fasi171642_G12972.t1#150 Fasi171205_G12959.t1#109 Fasi193955_G12902.t1#236 Fasi171646_G12992.t1#154 Fasi171287_G12995.t1#120 Fasi171130_G12926.t1#100 Fasi170991_G12899.t1#83 Fasi171290_G04226.t1#121 Fasi171207_G12953.t1#111 Fasi171322_G12966.t1#124 Fasi171658_G12937.t1#158 Fasi181100_G12949.t1#227 Fasi191317_G13038.t1#233 Fasi171212_G12929.t1#113 Fasi140052_G12985.t1#28 [illegible]
46886571 Fasi140026_G13049.t1#26 Fasi171647_G13004.t1#155 Fasi171644_G12879.t1#152 Fasi171375_G13011.t1#131 Fasi171077_G13037.t1#94 Fasi193968_G13066.t1#240 Fasi140025_G13146.t1#25
46886572 Fasi194006_G12590.t1#242 Fasi171130_G12927.t1#100 Fasi171375_G12967.t1#131 Fasi180279_G13020.t1#209 Fasi171323_G12977.t1#125 Fasi171658_G12938.t1#158 Fasi171210_G12924.t1#112 Fasi140026_G13006.t1#26 Fasi193955_G12903.t1#236 Fasi191317_G13039.t1#233 Fasi193959_G12929.t1#237 Fasi140052_G12986.t1#28 Fasi194008_G13004.t1#243 Fasi180246_G12976.t1#199 Fasi181100_G12950.t1#227 Fasi171287_G12996.t1#120 Fasi170566_G13024.t1#54 Fasi140025_G13103.t1#25 [illegible]
46886573 Fasi140025_G13356.t1#25 Fasi170464_G13580.t1#51 Fasi170460_G10584.t1#50 Fasi171330_G13556.t1#126 Fasi170542_G13482.t1#52 Fasi170988_G13355.t1#82 Fasi170908_G13572.t1#77 Fasi170880_G10469.t1#74
46886574 Fasi171330_G13555.t1#126 Fasi170464_G13579.t1#51 Fasi140025_G13357.t1#25 Fasi170460_G10585.t1#50 Fasi170542_G13481.t1#52 Fasi170908_G13571.t1#77 Fasi170880_G10470.t1#74
46886575 Fasi170880_G13293.t1#74 Fasi170988_G13283.t1#82 Fasi170460_G13429.t1#50 Fasi171330_G13459.t1#126 Fasi170677_G13410.t1#58 Fasi170908_G07183.t1#77 Fasi174240_G07420.t1#172
46886576 Fasi170460_G13423.t1#50 Fasi170880_G13284.t1#74 Fasi170677_G13402.t1#58 Fasi140025_G13395.t1#25 Fasi170542_G13312.t1#52 Fasi170880_G10446.t1#74 Fasi170464_G13378.t1#51
46886577 Fasi170880_G13282.t1#74 Fasi170464_G13374.t1#51 Fasi140025_G13359.t1#25 Fasi170988_G13419.t1#82 Fasi170460_G13505.t1#50
46886578 Fasi170988_G13248.t1#82 Fasi170464_G07598.t1#51 Fasi170880_G13536.t1#74 Fasi170460_G07666.t1#50 Fasi170542_G13283.t1#52
46886579 Fasi140025_G13382.t1#25 Fasi170542_G13306.t1#52
46886580 Fasi170460_G07668.t1#50 Fasi174240_G07422.t1#172 Fasi170464_G07596.t1#51 Fasi140025_G13361.t1#25 Fasi170542_G13281.t1#52 Fasi170908_G07185.t1#77 Fasi170880_G13534.t1#74 Fasi170677_G13640.t1#58 Fasi170988_G13250.t1#82 Fasi171330_G13461.t1#126
46886581 Fasi140025_G13363.t1#25 Fasi170460_G13502.t1#50 Fasi170988_G13416.t1#82 Fasi170908_G13593.t1#77 Fasi170880_G13437.t1#74
46886582 Fasi170542_G13490.t1#52 Fasi170460_G13503.t1#50 Fasi140025_G13364.t1#25 Fasi170880_G13436.t1#74 Fasi170464_G13588.t1#51 Fasi170988_G13417.t1#82 Fasi170677_G13594.t1#58
46886583 Fasi170677_G07089.t1#58 Fasi170908_G13474.t1#77 Fasi170464_G13618.t1#51 Fasi170880_G13335.t1#74 Fasi170988_G13401.t1#82 Fasi170464_G13537.t1#51 Fasi171330_G12769.t1#126 Fasi140025_G13523.t1#25 Fasi140025_G13366.t1#25 Fasi170460_G13493.t1#50 Fasi170677_G13490.t1#58
46886584 Fasi170460_G13583.t1#50 Fasi170880_G13336.t1#74 Fasi170988_G13400.t1#82 Fasi171330_G12768.t1#126 Fasi170908_G13473.t1#77 Fasi140025_G13524.t1#25 Fasi170677_G13491.t1#58 Fasi170464_G13536.t1#51
46886585 Fasi170542_G13442.t1#52
46886586 Fasi140025_G13371.t1#25
46886587 Fasi170464_G13604.t1#51 Fasi170908_G13506.t1#77 Fasi170988_G07099.t1#82 Fasi140025_G13369.t1#25 Fasi170460_G13528.t1#50 Fasi170880_G13560.t1#74 Fasi171330_G13331.t1#126 Fasi170542_G13517.t1#52 Fasi170677_G13625.t1#58
46886588 Fasi170464_G13516.t1#51 Fasi170460_G13484.t1#50 Fasi140025_G13377.t1#25 Fasi170542_G13424.t1#52 Fasi170908_G13414.t1#77 Fasi170677_G13530.t1#58 Fasi171330_G13504.t1#126 Fasi170880_G13263.t1#74 Fasi174240_G13297.t1#172
46886589 Fasi170677_G10038.t1#58 Fasi170464_G13481.t1#51 Fasi170988_G13492.t1#82 Fasi170542_G10441.t1#52 Fasi170460_G07660.t1#50 Fasi170880_G13380.t1#74 Fasi171330_G13364.t1#126 Fasi140025_G13376.t1#25 Fasi174240_G13280.t1#172 Fasi170908_G13522.t1#77
46886590 Fasi171330_G13330.t1#126 Fasi170464_G13603.t1#51 Fasi170880_G13559.t1#74 Fasi170542_G13516.t1#52 Fasi140025_G13380.t1#25 Fasi174240_G13312.t1#172
46886591 Fasi174240_G13302.t1#172 Fasi170464_G13390.t1#51 Fasi170677_G13409.t1#58 Fasi170542_G13418.t1#52 Fasi170880_G13463.t1#74 Fasi170908_G13338.t1#77 Fasi170988_G13402.t1#82 Fasi140025_G13396.t1#25 Fasi170460_G13530.t1#50
46886592 Fasi171330_G13407.t1#126 Fasi170542_G13409.t1#52 Fasi170464_G13439.t1#51 Fasi140025_G13400.t1#25 Fasi170880_G13333.t1#74 Fasi170460_G13570.t1#50 Fasi170908_G13363.t1#77 Fasi170677_G13440.t1#58 Fasi170880_G13290.t1#82
46886593 Fasi170677_G13429.t1#58 Fasi140025_G13401.t1#25 Fasi170542_G13410.t1#52 Fasi170880_G13332.t1#74 Fasi170908_G13362.t1#77 Fasi170464_G13438.t1#51 Fasi170460_G13569.t1#50
46886594 Fasi170908_G13360.t1#77 Fasi140025_G13403.t1#25 Fasi170677_G13437.t1#58 Fasi171330_G13409.t1#126 Fasi170460_G13567.t1#50
46886595 Fasi140025_G13407.t1#25 Fasi170542_G13416.t1#52 Fasi170677_G13435.t1#58 Fasi170460_G13438.t1#50 Fasi170988_G13295.t1#82 Fasi170908_G13358.t1#77 Fasi170880_G13327.t1#74 Fasi171330_G13414.t1#126 Fasi170464_G13433.t1#51
46886596 Fasi170677_G13433.t1#58 Fasi170988_G13297.t1#82 Fasi170542_G13324.t1#52 Fasi170460_G13440.t1#50 Fasi140025_G13409.t1#25 Fasi171330_G13416.t1#126 Fasi170908_G13356.t1#77 Fasi170880_G13325.t1#74 Fasi170464_G13431.t1#51
46886597 Fasi170464_G13429.t1#51 Fasi140025_G13412.t1#25 Fasi170988_G13300.t1#82 Fasi170677_G13430.t1#58 Fasi171330_G13419.t1#126 Fasi170460_G13442.t1#50 Fasi170542_G13326.t1#52 Fasi170908_G13353.t1#77 Fasi170880_G13322.t1#74
46886598 Fasi170542_G13325.t1#52 Fasi170464_G13430.t1#51 Fasi170908_G13354.t1#77 Fasi170677_G13431.t1#58 Fasi140025_G13411.t1#25 Fasi170880_G13323.t1#74 Fasi171330_G13418.t1#126 Fasi170460_G13441.t1#50 Fasi170988_G13299.t1#82
46886599 Fasi170880_G13320.t1#74 Fasi170542_G13328.t1#52 Fasi170988_G13302.t1#82 Fasi171330_G13506.t1#126 Fasi170677_G13428.t1#58 Fasi170464_G13426.t1#51 Fasi170908_G13351.t1#77 Fasi140025_G13413.t1#25 Fasi170460_G13444.t1#50
46886600 Fasi170880_G13349.t1#74 Fasi140025_G13478.t1#25 Fasi171330_G13553.t1#126 Fasi170460_G13627.t1#50 Fasi170464_G13402.t1#51
46886601 Fasi170880_G13319.t1#74 Fasi170542_G13329.t1#52 Fasi170908_G13350.t1#77 Fasi140025_G13414.t1#25 Fasi171330_G13507.t1#126 Fasi170464_G13425.t1#51 Fasi170460_G13445.t1#50 Fasi170677_G13427.t1#58 Fasi170988_G13303.t1#82
46886602 Fasi170542_G13330.t1#52 Fasi170464_G13424.t1#51 Fasi170988_G13304.t1#82 Fasi170460_G13446.t1#50 Fasi140025_G13415.t1#25 Fasi170677_G13426.t1#58 Fasi170908_G13349.t1#77 Fasi171330_G13508.t1#126 Fasi170880_G13318.t1#74
46886603 Fasi170988_G13305.t1#82 Fasi170460_G13447.t1#50 Fasi171330_G13509.t1#126 Fasi140025_G13416.t1#25 Fasi170677_G13425.t1#58 Fasi170464_G13423.t1#51 Fasi170880_G13317.t1#74 Fasi170542_G13331.t1#52 Fasi170908_G13348.t1#77
46886604 Fasi170677_G13422.t1#58 Fasi170460_G13449.t1#50 Fasi170464_G13420.t1#51 Fasi171330_G13512.t1#126 Fasi170988_G13307.t1#82 Fasi170542_G13333.t1#52 Fasi170880_G13314.t1#74 Fasi170908_G13346.t1#77
46886605 Fasi170908_G13344.t1#77 Fasi170460_G13451.t1#50 Fasi170464_G13418.t1#51 Fasi170677_G13420.t1#58 Fasi170880_G13312.t1#74 Fasi171330_G13514.t1#126 Fasi140025_G13420.t1#25 Fasi170542_G13335.t1#52 Fasi170988_G13309.t1#82
46886606 Fasi170988_G13321.t1#82 Fasi170880_G10460.t1#74
46886607 Fasi170880_G13311.t1#74 Fasi170464_G13417.t1#51 Fasi170460_G13452.t1#50 Fasi140025_G13421.t1#25 Fasi170988_G13310.t1#82 Fasi170542_G13336.t1#52 Fasi170677_G13419.t1#58 Fasi171330_G13515.t1#126 Fasi170908_G13343.t1#77
46886608 Fasi171330_G13448.t1#126 Fasi170908_G13383.t1#77
46886609 Fasi170677_G13415.t1#58 Fasi171330_G13396.t1#126 Fasi140025_G13425.t1#25 Fasi170880_G13307.t1#74 Fasi170908_G13482.t1#77 Fasi170460_G07571.t1#50 Fasi170542_G13340.t1#52 Fasi170988_G13351.t1#82 Fasi170464_G13413.t1#51
46886610 Fasi140025_G13424.t1#25 Fasi170677_G13416.t1#58 Fasi170908_G13481.t1#77 Fasi170988_G13352.t1#82 Fasi171330_G13395.t1#126 Fasi170464_G13414.t1#51 Fasi170880_G13308.t1#74 Fasi170460_G07572.t1#50 Fasi170542_G13339.t1#52
46886611 Fasi170908_G13483.t1#77 Fasi170880_G13306.t1#74 Fasi170464_G13412.t1#51 Fasi170988_G13350.t1#82 Fasi140025_G13426.t1#25 Fasi170460_G07570.t1#50 Fasi170542_G13341.t1#52 Fasi171330_G13397.t1#126 Fasi170677_G13414.t1#58
46886612 Fasi171330_G13398.t1#126 Fasi170677_G13413.t1#58 Fasi170464_G13411.t1#51 Fasi170460_G07569.t1#50 Fasi170880_G13305.t1#74 Fasi140025_G13427.t1#25 Fasi170908_G13484.t1#77 Fasi170542_G13342.t1#52 Fasi170988_G13349.t1#82
46886613 Fasi170677_G13525.t1#58 Fasi171205_G01271.t1#109 Fasi171117_G00941.t1#98 Fasi171290_G00939.t1#121 Fasi180101_G00934.t1#177 Fasi171120_G00933.t1#99 Fasi180269_G00603.t1#204 Fasi180100_G00937.t1#176 Fasi180271_G00922.t1#205 Fasi171278_G00922.t1#119 Fasi171642_G00942.t1#150 Fasi170372_G00931.t1#45 Fasi170542_G13345.t1#52 Fasi171011_G00934.t1#85 Fasi170908_G13488.t1#77 Fasi170988_G13346.t1#82 Fasi172117_G00768.t1#165 Fasi170464_G13408.t1#51 [illegible]
46886614 Fasi171011_G00933.t1#85 Fasi171212_G00949.t1#113 Fasi171005_G00937.t1#84 Fasi171330_G13402.t1#126 Fasi171290_G00938.t1#121 Fasi180100_G00936.t1#176 Fasi180273_G01165.t1#206 Fasi170372_G00930.t1#45 Fasi171120_G00932.t1#99 Fasi180101_G00933.t1#177 Fasi170988_G13345.t1#82 Fasi170677_G13526.t1#58 Fasi172119_G00905.t1#167 Fasi140025_G13431.t1#25 Fasi170460_G07565.t1#50 Fasi171117_G00940.t1#98 Fasi172117_G00767.t1#165 [illegible]
46886615 Fasi170464_G13407.t1#51 Fasi170542_G13346.t1#52 Fasi140025_G13432.t1#25 Fasi170880_G13300.t1#74 Fasi170908_G13490.t1#77 Fasi170988_G13344.t1#82 Fasi170460_G07564.t1#50
46886616 Fasi170542_G13347.t1#52 Fasi170677_G13527.t1#58 Fasi170908_G13446.t1#77 Fasi171330_G13403.t1#126 Fasi140025_G13433.t1#25 Fasi170988_G13343.t1#82 Fasi170880_G13353.t1#74 Fasi170460_G07563.t1#50
46886617 Fasi170988_G13342.t1#82 Fasi170460_G07562.t1#50 Fasi140025_G13434.t1#25 Fasi171330_G13404.t1#126 Fasi170542_G13348.t1#52 Fasi170908_G13447.t1#77 Fasi170880_G13352.t1#74 Fasi170677_G13528.t1#58 Fasi170464_G13405.t1#51
46886618 Fasi170464_G13404.t1#51 Fasi140025_G13435.t1#25 Fasi171330_G13405.t1#126 Fasi170460_G07561.t1#50 Fasi170880_G13351.t1#74
46886619 Fasi170542_G13372.t1#52 Fasi170880_G13354.t1#74 Fasi140025_G13436.t1#25
46886620 Fasi170677_G13508.t1#58 Fasi170908_G13424.t1#77 Fasi140025_G13437.t1#25 Fasi170464_G13469.t1#51 Fasi171330_G07671.t1#126 Fasi170542_G13371.t1#52 Fasi174240_G13334.t1#172 Fasi170460_G07694.t1#50 Fasi170880_G13355.t1#74 Fasi170988_G13333.t1#82
46886621 Fasi174240_G13333.t1#172 Fasi170677_G13509.t1#58 Fasi170542_G13370.t1#52 Fasi170988_G13334.t1#82 Fasi170464_G13470.t1#51 Fasi140025_G13438.t1#25 Fasi171330_G07672.t1#126 Fasi170880_G13356.t1#74
46886622 Fasi170880_G13357.t1#74 Fasi170464_G13471.t1#51 Fasi170988_G13335.t1#82 Fasi170542_G13369.t1#52 Fasi171330_G07673.t1#126 Fasi170677_G13510.t1#58 Fasi174240_G13332.t1#172
46886623 Fasi170464_G07649.t1#51 Fasi140025_G13442.t1#25 Fasi170988_G13338.t1#82 Fasi170880_G13360.t1#74 Fasi174240_G13329.t1#172 Fasi170542_G13366.t1#52 Fasi171330_G07676.t1#126 Fasi170677_G13513.t1#58
[illegible]

Fasi171478_G09515.t1#143 Fasi181150_G09645.t1#230 Fasi181100_G09598.t1#227 Fasi140020_G09564.t1#20

Fasi170765_G09254.t1#64 Fasi171471_G09731.t1#138 Fasi180212_G09910.t1#191 Fasi172114_G09565.t1#164 Fasi172119_G09575.t1#167 Fasi170372_G09632.t1#45 Fasi140024_G09615.t1#24 Fasi193955_G09584.t1#236 Fasi171669_G09603.t1#162 Fasi180282_G09543.t1#211 Fasi171042_G09544.t1#90 Fasi171467_G10359.t1#136 Fasi191882_G09294.t1#234 Fasi171645_G09637.t1#153 Fasi180277_G08792.t1#208 Fasi174251_G09519.t1#173 Fasi194010_G09593.t1#244 Fasi180102_G09557.t1#178 [illegible]

[illegible]

Table S3 GO enrichment of pangenome accessory genes.

ID	Description	GeneRatio	BgRatio	pvalue	p.adjust	qvalue	geneID	Count	Factor	ONTOLOGY
GO:0003964	RNA-directed DNA polymerase activity	27/253	31/4104	1.39E-29	2.59E-26	2.31E-26	Fasi180341_G05623.t1/Fasi171646_G13245.t1/Fasi160270_G07411.t1/Fasi171042_G07537.t1/Fasi180099_G07564.t1/Fasi140011_G10419.t1/Fasi194010_G07522.t1/Fasi171308_G13331.t1/Fasi172120_G10389.t1/Fasi180188_G00003.t1/Fasi170460_G13497.t1/Fasi160226_G07976.t1/Fasi180341_G07680.t1/Fasi181006_G07603.t1/Fasi171290_G07966.t1/Fasi140006_G10434.t1/Fasi171669_G13242.t1/Fasi170908_G13312.t1/Fasi193963_G13202.t1/Fasi180648_G07580.t1/Fasi160277_G07744.t1/Fasi180273_G13260.t1/Fasi180106_G10511.t1/Fasi140025_G13363.t1/Fasi180220_G13201.t1/Fasi180099_G10411.t1/Fasi193963_G13201.t1	27	Accessory groups	MF
GO:0034061	DNA polymerase activity	28/253	44/4104	5.28E-24	4.93E-21	4.40E-21	Fasi140052_G00902.t1/Fasi180341_G05623.t1/Fasi171646_G13245.t1/Fasi160270_G07411.t1/Fasi171042_G07537.t1/Fasi180099_G07564.t1/Fasi140011_G10419.t1/Fasi194010_G07522.t1/Fasi171308_G13331.t1/Fasi172120_G10389.t1/Fasi180188_G00003.t1/Fasi170460_G13497.t1/Fasi160226_G07976.t1/Fasi180341_G07680.t1/Fasi181006_G07603.t1/Fasi171290_G07966.t1/Fasi140006_G10434.t1/Fasi171669_G13242.t1/Fasi170908_G13312.t1/Fasi193963_G13202.t1/Fasi180648_G07580.t1/Fasi160277_G07744.t1/Fasi180273_G13260.t1/Fasi180106_G10511.t1/Fasi140025_G13363.t1/Fasi180220_G13201.t1/Fasi180099_G10411.t1/Fasi193963_G13201.t1	28	Accessory groups	MF
GO:0006278	RNA-templated DNA biosynthetic process	27/253	41/4104	8.97E-24	5.58E-21	4.98E-21	Fasi180341_G05623.t1/Fasi171646_G13245.t1/Fasi160270_G07411.t1/Fasi171042_G07537.t1/Fasi180099_G07564.t1/Fasi140011_G10419.t1/Fasi194010_G07522.t1/Fasi171308_G13331.t1/Fasi172120_G10389.t1/Fasi180188_G00003.t1/Fasi170460_G13497.t1/Fasi160226_G07976.t1/Fasi180341_G07680.t1/Fasi181006_G07603.t1/Fasi171290_G07966.t1/Fasi140006_G10434.t1/Fasi171669_G13242.t1/Fasi170908_G13312.t1/Fasi193963_G13202.t1/Fasi180648_G07580.t1/Fasi160277_G07744.t1/Fasi180273_G13260.t1/Fasi180106_G10511.t1/Fasi140025_G13363.t1/Fasi180220_G13201.t1/Fasi180099_G10411.t1/Fasi193963_G13201.t1	27	Accessory groups	BP
GO:0140097	catalytic	39/253	132/41	1.18E-	5.49E-15	4.90E-15	Fasi170880_G13549.t1/Fasi170908_G07187.t1/Fasi171330_G133	39	Accessory	MF

	activity, acting on DNA		04	17			41.t1/Fasi170867_G07532.t1/Fasi170464_G13385.t1/Fasi160280_G13160.t1/Fasi171330_G07612.t1/Fasi170991_G13151.t1/Fasi194008_G13260.t1/Fasi171330_G13468.t1/Fasi140027_G07584.t1/Fasi140052_G00902.t1/Fasi180341_G05623.t1/Fasi171646_G13245.t1/Fasi160270_G07411.t1/Fasi171042_G07537.t1/Fasi180099_G07564.t1/Fasi140011_G10419.t1/Fasi194010_G07522.t1/Fasi171308_G13331.t1/Fasi172120_G10389.t1/Fasi180188_G00003.t1/Fasi170460_G13497.t1/Fasi160226_G07976.t1/Fasi180341_G07680.t1/Fasi181006_G07603.t1/Fasi171290_G07966.t1/Fasi140006_G10434.t1/Fasi171669_G13242.t1/Fasi170908_G13312.t1/Fasi193963_G13202.t1/Fasi180648_G07580.t1/Fasi160277_G07744.t1/Fasi180273_G13260.t1/Fasi180106_G10511.t1/Fasi140025_G13363.t1/Fasi180220_G13201.t1/Fasi180099_G10411.t1/Fasi193963_G13201.t1		groups	
GO:0071897	DNA biosynthetic process	28/253	67/4104	2.10E-17	7.83E-15	6.99E-15	Fasi140052_G00902.t1/Fasi180341_G05623.t1/Fasi171646_G13245.t1/Fasi160270_G07411.t1/Fasi171042_G07537.t1/Fasi180099_G07564.t1/Fasi140011_G10419.t1/Fasi194010_G07522.t1/Fasi171308_G13331.t1/Fasi172120_G10389.t1/Fasi180188_G00003.t1/Fasi170460_G13497.t1/Fasi160226_G07976.t1/Fasi180341_G07680.t1/Fasi181006_G07603.t1/Fasi171290_G07966.t1/Fasi140006_G10434.t1/Fasi171669_G13242.t1/Fasi170908_G13312.t1/Fasi193963_G13202.t1/Fasi180648_G07580.t1/Fasi160277_G07744.t1/Fasi180273_G13260.t1/Fasi180106_G10511.t1/Fasi140025_G13363.t1/Fasi180220_G13201.t1/Fasi180099_G10411.t1/Fasi193963_G13201.t1	28	Accessory groups	BP
GO:1990961	xenobiotic detoxification by transmembrane export across the plasma membrane	16/253	20/4104	1.07E-16	3.33E-14	2.97E-14	Fasi171005_G07405.t1/Fasi180220_G04579.t1/Fasi170382_G04561.t1/Fasi180242_G07569.t1/Fasi170719_G06635.t1/Fasi180222_G06592.t1/Fasi171274_G12770.t1/Fasi180192_G10156.t1/Fasi171301_G07643.t1/Fasi180188_G01292.t1/Fasi180242_G12788.t1/Fasi170382_G07580.t1/Fasi180222_G12818.t1/Fasi180281_G09711.t1/Fasi181100_G10113.t1/Fasi160270_G10105.t1	16	Accessory groups	BP
GO:0016779	nucleotidyltransferase activity	29/253	81/4104	7.89E-16	2.11E-13	1.88E-13	Fasi140052_G00902.t1/Fasi180283_G08052.t1/Fasi180341_G05623.t1/Fasi171646_G13245.t1/Fasi160270_G07411.t1/Fasi171042_G07537.t1/Fasi180099_G07564.t1/Fasi140011_G10419.t1/Fasi194	29	Accessory groups	MF

							010_G07522.t1/Fasi171308_G13331.t1/Fasi172120_G10389.t1/Fasi180188_G00003.t1/Fasi170460_G13497.t1/Fasi160226_G07976.t1/Fasi180341_G07680.t1/Fasi181006_G07603.t1/Fasi171290_G07966.t1/Fasi140006_G10434.t1/Fasi171669_G13242.t1/Fasi170908_G13312.t1/Fasi193963_G13202.t1/Fasi180648_G07580.t1/Fasi160277_G07744.t1/Fasi180273_G13260.t1/Fasi180106_G10511.t1/Fasi140025_G13363.t1/Fasi180220_G13201.t1/Fasi180099_G10411.t1/Fasi193963_G13201.t1			
GO:0006259	DNA metabolic process	62/253	388/4104	2.45E-13	5.72E-11	5.11E-11	Fasi170880_G13549.t1/Fasi170908_G07187.t1/Fasi171330_G13341.t1/Fasi170867_G07532.t1/Fasi170464_G13385.t1/Fasi160280_G13160.t1/Fasi171330_G07612.t1/Fasi170991_G13151.t1/Fasi194008_G13260.t1/Fasi171330_G13468.t1/Fasi140027_G07584.t1/Fasi171330_G00515.t1/Fasi170988_G13513.t1/Fasi140052_G00902.t1/Fasi170677_G10032.t1/Fasi171115_G03161.t1/Fasi194004_G03166.t1/Fasi171005_G07405.t1/Fasi180220_G04579.t1/Fasi170382_G04561.t1/Fasi180242_G07569.t1/Fasi180267_G06278.t1/Fasi180222_G05219.t1/Fasi171274_G12770.t1/Fasi180192_G10156.t1/Fasi171301_G07643.t1/Fasi180188_G01292.t1/Fasi180242_G12788.t1/Fasi170382_G07580.t1/Fasi180222_G12818.t1/Fasi140019_G09559.t1/Fasi180341_G11247.t1/Fasi180341_G05623.t1/Fasi171646_G13245.t1/Fasi160270_G07411.t1/Fasi171042_G07537.t1/Fasi180099_G07564.t1/Fasi140011_G10419.t1/Fasi194010_G07522.t1/Fasi171308_G13331.t1/Fasi172120_G10389.t1/Fasi180188_G00003.t1/Fasi170460_G13497.t1/Fasi160226_G07976.t1/Fasi180341_G07680.t1/Fasi181006_G07603.t1/Fasi171290_G07966.t1/Fasi140006_G10434.t1/Fasi171669_G13242.t1/Fasi170908_G13312.t1/Fasi170728_G01973.t1/Fasi193963_G13202.t1/Fasi180648_G07580.t1/Fasi160277_G07744.t1/Fasi180273_G13260.t1/Fasi180106_G10511.t1/Fasi160280_G00505.t1/Fasi140025_G13363.t1/Fasi180220_G13201.t1/Fasi180099_G10411.t1/Fasi171484_G13024.t1/Fasi193963_G13201.t1	62	Accessory groups	BP
GO:0042562	hormone binding	11/253	12/4104	4.51E-13	6.48E-11	5.78E-11	Fasi171005_G07405.t1/Fasi180220_G04579.t1/Fasi170382_G04561.t1/Fasi180242_G07569.t1/Fasi171274_G12770.t1/Fasi180192_G10156.t1/Fasi171301_G07643.t1/Fasi180188_G01292.t1/Fasi180242_G12788.t1/Fasi170382_G07580.t1/Fasi180222_G12818.t1	11	Accessory groups	MF

GO:0099130	estrogen binding	11/253	12/4104	4.51E-13	6.48E-11	5.78E-11	Fasi171005_G07405.t1/Fasi180220_G04579.t1/Fasi170382_G04561.t1/Fasi180242_G07569.t1/Fasi171274_G12770.t1/Fasi180192_G10156.t1/Fasi171301_G07643.t1/Fasi180188_G01292.t1/Fasi180242_G12788.t1/Fasi170382_G07580.t1/Fasi180222_G12818.t1	11	Accessory groups	MF
GO:1903875	corticosterone binding	11/253	12/4104	4.51E-13	6.48E-11	5.78E-11	Fasi171005_G07405.t1/Fasi180220_G04579.t1/Fasi170382_G04561.t1/Fasi180242_G07569.t1/Fasi171274_G12770.t1/Fasi180192_G10156.t1/Fasi171301_G07643.t1/Fasi180188_G01292.t1/Fasi180242_G12788.t1/Fasi170382_G07580.t1/Fasi180222_G12818.t1	11	Accessory groups	MF
GO:1903924	estradiol binding	11/253	12/4104	4.51E-13	6.48E-11	5.78E-11	Fasi171005_G07405.t1/Fasi180220_G04579.t1/Fasi170382_G04561.t1/Fasi180242_G07569.t1/Fasi171274_G12770.t1/Fasi180192_G10156.t1/Fasi171301_G07643.t1/Fasi180188_G01292.t1/Fasi180242_G12788.t1/Fasi170382_G07580.t1/Fasi180222_G12818.t1	11	Accessory groups	MF
GO:1990239	steroid hormone binding	11/253	12/4104	4.51E-13	6.48E-11	5.78E-11	Fasi171005_G07405.t1/Fasi180220_G04579.t1/Fasi170382_G04561.t1/Fasi180242_G07569.t1/Fasi171274_G12770.t1/Fasi180192_G10156.t1/Fasi171301_G07643.t1/Fasi180188_G01292.t1/Fasi180242_G12788.t1/Fasi170382_G07580.t1/Fasi180222_G12818.t1	11	Accessory groups	MF
GO:0015244	fluconazole transmembrane transporter activity	11/253	13/4104	2.77E-12	2.88E-10	2.57E-10	Fasi171005_G07405.t1/Fasi180220_G04579.t1/Fasi170382_G04561.t1/Fasi180242_G07569.t1/Fasi171274_G12770.t1/Fasi180192_G10156.t1/Fasi171301_G07643.t1/Fasi180188_G01292.t1/Fasi180242_G12788.t1/Fasi170382_G07580.t1/Fasi180222_G12818.t1	11	Accessory groups	MF
GO:0015903	fluconazole transport	11/253	13/4104	2.77E-12	2.88E-10	2.57E-10	Fasi171005_G07405.t1/Fasi180220_G04579.t1/Fasi170382_G04561.t1/Fasi180242_G07569.t1/Fasi171274_G12770.t1/Fasi180192_G10156.t1/Fasi171301_G07643.t1/Fasi180188_G01292.t1/Fasi180242_G12788.t1/Fasi170382_G07580.t1/Fasi180222_G12818.t1	11	Accessory groups	BP
GO:0043178	alcohol binding	11/253	13/4104	2.77E-12	2.88E-10	2.57E-10	Fasi171005_G07405.t1/Fasi180220_G04579.t1/Fasi170382_G04561.t1/Fasi180242_G07569.t1/Fasi171274_G12770.t1/Fasi180192_G10156.t1/Fasi171301_G07643.t1/Fasi180188_G01292.t1/Fasi180242_G12788.t1/Fasi170382_G07580.t1/Fasi180222_G12818.t1	11	Accessory groups	MF
GO:0048545	response to steroid hormone	11/253	13/4104	2.77E-12	2.88E-10	2.57E-10	Fasi171005_G07405.t1/Fasi180220_G04579.t1/Fasi170382_G04561.t1/Fasi180242_G07569.t1/Fasi171274_G12770.t1/Fasi180192_G10156.t1/Fasi171301_G07643.t1/Fasi180188_G01292.t1/Fasi180242_G12788.t1/Fasi170382_G07580.t1/Fasi180222_G12818.t1	11	Accessory groups	BP
GO:0071383	cellular response to steroid	11/253	13/4104	2.77E-12	2.88E-10	2.57E-10	Fasi171005_G07405.t1/Fasi180220_G04579.t1/Fasi170382_G04561.t1/Fasi180242_G07569.t1/Fasi171274_G12770.t1/Fasi180192_G10156.t1/Fasi171301_G07643.t1/Fasi180188_G01292.t1/Fasi180	11	Accessory groups	BP

	hormone stimulus						242_G12788.t1/Fasi170382_G07580.t1/Fasi180222_G12818.t1			
GO:0000724	double-strand break repair via homologous recombination	26/253	87/4104	3.47E-12	2.95E-10	2.63E-10	Fasi170880_G13549.t1/Fasi170908_G07187.t1/Fasi171330_G13341.t1/Fasi170867_G07532.t1/Fasi170464_G13385.t1/Fasi160280_G13160.t1/Fasi171330_G07612.t1/Fasi170991_G13151.t1/Fasi194008_G13260.t1/Fasi171330_G13468.t1/Fasi140027_G07584.t1/Fasi170988_G13513.t1/Fasi171115_G03161.t1/Fasi194004_G03166.t1/Fasi171005_G07405.t1/Fasi180220_G04579.t1/Fasi170382_G04561.t1/Fasi180242_G07569.t1/Fasi171274_G12770.t1/Fasi180192_G10156.t1/Fasi171301_G07643.t1/Fasi180188_G01292.t1/Fasi180242_G12788.t1/Fasi170382_G07580.t1/Fasi180222_G12818.t1/Fasi140019_G09559.t1	26	Accessory groups	BP
GO:0009725	response to hormone	12/253	16/4104	3.42E-12	2.95E-10	2.63E-10	Fasi171005_G07405.t1/Fasi180220_G04579.t1/Fasi170382_G04561.t1/Fasi180242_G07569.t1/Fasi171274_G12770.t1/Fasi180192_G10156.t1/Fasi171301_G07643.t1/Fasi180188_G01292.t1/Fasi180242_G12788.t1/Fasi170382_G07580.t1/Fasi180222_G12818.t1/Fasi170464_G07649.t1	12	Accessory groups	BP
GO:0032870	cellular response to hormone stimulus	12/253	16/4104	3.42E-12	2.95E-10	2.63E-10	Fasi171005_G07405.t1/Fasi180220_G04579.t1/Fasi170382_G04561.t1/Fasi180242_G07569.t1/Fasi171274_G12770.t1/Fasi180192_G10156.t1/Fasi171301_G07643.t1/Fasi180188_G01292.t1/Fasi180242_G12788.t1/Fasi170382_G07580.t1/Fasi180222_G12818.t1/Fasi170464_G07649.t1	12	Accessory groups	BP
GO:1901618	organic hydroxy compound transmembrane transporter activity	12/253	16/4104	3.42E-12	2.95E-10	2.63E-10	Fasi171005_G07405.t1/Fasi180220_G04579.t1/Fasi170382_G04561.t1/Fasi180242_G07569.t1/Fasi180341_G05926.t1/Fasi171274_G12770.t1/Fasi180192_G10156.t1/Fasi171301_G07643.t1/Fasi180188_G01292.t1/Fasi180242_G12788.t1/Fasi170382_G07580.t1/Fasi180222_G12818.t1	12	Accessory groups	MF
GO:0015665	alcohol transmembrane transporter activity	11/253	14/4104	1.22E-11	9.93E-10	8.86E-10	Fasi171005_G07405.t1/Fasi180220_G04579.t1/Fasi170382_G04561.t1/Fasi180242_G07569.t1/Fasi171274_G12770.t1/Fasi180192_G10156.t1/Fasi171301_G07643.t1/Fasi180188_G01292.t1/Fasi180242_G12788.t1/Fasi170382_G07580.t1/Fasi180222_G12818.t1	11	Accessory groups	MF
GO:0000725	recombinational repair	26/253	92/4104	1.45E-11	1.13E-09	1.01E-09	Fasi170880_G13549.t1/Fasi170908_G07187.t1/Fasi171330_G13341.t1/Fasi170867_G07532.t1/Fasi170464_G13385.t1/Fasi160280_G13160.t1/Fasi171330_G07612.t1/Fasi170991_G13151.t1/Fasi194008_G13260.t1/Fasi171330_G13468.t1/Fasi140027_G07584.t1/Fa	26	Accessory groups	BP

							si170988_G13513.t1/Fasi171115_G03161.t1/Fasi194004_G03166.t1/Fasi171005_G07405.t1/Fasi180220_G04579.t1/Fasi170382_G04561.t1/Fasi180242_G07569.t1/Fasi171274_G12770.t1/Fasi180192_G10156.t1/Fasi171301_G07643.t1/Fasi180188_G01292.t1/Fasi180242_G12788.t1/Fasi170382_G07580.t1/Fasi180222_G12818.t1/Fasi140019_G09559.t1			
GO:0008559	ABC-type xenobiotic transporter activity	12/253	18/4104	3.12E-11	2.33E-09	2.08E-09	Fasi171005_G07405.t1/Fasi180220_G04579.t1/Fasi170382_G04561.t1/Fasi180242_G07569.t1/Fasi171274_G12770.t1/Fasi180192_G10156.t1/Fasi171301_G07643.t1/Fasi180188_G01292.t1/Fasi180242_G12788.t1/Fasi170382_G07580.t1/Fasi180222_G12818.t1/Fasi180275_G11532.t1	12	Accessory groups	MF
GO:0046618	xenobiotic export from cell	16/253	35/4104	3.86E-11	2.77E-09	2.47E-09	Fasi171005_G07405.t1/Fasi180220_G04579.t1/Fasi170382_G04561.t1/Fasi180242_G07569.t1/Fasi170719_G06635.t1/Fasi180222_G06592.t1/Fasi171274_G12770.t1/Fasi180192_G10156.t1/Fasi171301_G07643.t1/Fasi180188_G01292.t1/Fasi180242_G12788.t1/Fasi170382_G07580.t1/Fasi180222_G12818.t1/Fasi180281_G09711.t1/Fasi181100_G10113.t1/Fasi160270_G10105.t1	16	Accessory groups	BP
GO:0042908	xenobiotic transport	12/253	19/4104	8.00E-11	5.34E-09	4.76E-09	Fasi171005_G07405.t1/Fasi180220_G04579.t1/Fasi170382_G04561.t1/Fasi180242_G07569.t1/Fasi171274_G12770.t1/Fasi180192_G10156.t1/Fasi171301_G07643.t1/Fasi180188_G01292.t1/Fasi180242_G12788.t1/Fasi170382_G07580.t1/Fasi180222_G12818.t1/Fasi180275_G11532.t1	12	Accessory groups	BP
GO:0042910	xenobiotic transmembrane transporter activity	12/253	19/4104	8.00E-11	5.34E-09	4.76E-09	Fasi171005_G07405.t1/Fasi180220_G04579.t1/Fasi170382_G04561.t1/Fasi180242_G07569.t1/Fasi171274_G12770.t1/Fasi180192_G10156.t1/Fasi171301_G07643.t1/Fasi180188_G01292.t1/Fasi180242_G12788.t1/Fasi170382_G07580.t1/Fasi180222_G12818.t1/Fasi180275_G11532.t1	12	Accessory groups	MF
GO:1990414	replication-born double-strand break repair via sister chromatid exchange	12/253	20/4104	1.89E-10	1.18E-08	1.05E-08	Fasi170988_G13513.t1/Fasi171005_G07405.t1/Fasi180220_G04579.t1/Fasi170382_G04561.t1/Fasi180242_G07569.t1/Fasi171274_G12770.t1/Fasi180192_G10156.t1/Fasi171301_G07643.t1/Fasi180188_G01292.t1/Fasi180242_G12788.t1/Fasi170382_G07580.t1/Fasi180222_G12818.t1	12	Accessory groups	BP
GO:0042626	ATPase-coupled transmembrane transporter	22/253	75/4104	2.64E-10	1.59E-08	1.42E-08	Fasi171075_G07733.t1/Fasi171005_G07405.t1/Fasi180220_G04579.t1/Fasi170382_G04561.t1/Fasi180242_G07569.t1/Fasi170719_G06635.t1/Fasi180222_G06592.t1/Fasi171274_G12770.t1/Fasi180	22	Accessory groups	MF

	activity						192_G10156.t1/Fasi171301_G07643.t1/Fasi180188_G01292.t1/Fasi180242_G12788.t1/Fasi170382_G07580.t1/Fasi180222_G12818.t1/Fasi180281_G09711.t1/Fasi181100_G10113.t1/Fasi180104_G10679.t1/Fasi180279_G10743.t1/Fasi170460_G13556.t1/Fasi160270_G10105.t1/Fasi194006_G13141.t1/Fasi180275_G11532.t1			
GO:0009378	four-way junction helicase activity	11/253	17/4104	3.52E-10	1.99E-08	1.78E-08	Fasi170880_G13549.t1/Fasi170908_G07187.t1/Fasi171330_G13341.t1/Fasi170867_G07532.t1/Fasi170464_G13385.t1/Fasi160280_G13160.t1/Fasi171330_G07612.t1/Fasi170991_G13151.t1/Fasi194008_G13260.t1/Fasi171330_G13468.t1/Fasi140027_G07584.t1	11	Accessory groups	MF
GO:0015399	primary active transmembrane transporter activity	22/253	79/4104	8.04E-10	4.29E-08	3.83E-08	Fasi171075_G07733.t1/Fasi171005_G07405.t1/Fasi180220_G04579.t1/Fasi170382_G04561.t1/Fasi180242_G07569.t1/Fasi170719_G06635.t1/Fasi180222_G06592.t1/Fasi171274_G12770.t1/Fasi180192_G10156.t1/Fasi171301_G07643.t1/Fasi180188_G01292.t1/Fasi180242_G12788.t1/Fasi170382_G07580.t1/Fasi180222_G12818.t1/Fasi180281_G09711.t1/Fasi181100_G10113.t1/Fasi180104_G10679.t1/Fasi180279_G10743.t1/Fasi170460_G13556.t1/Fasi160270_G10105.t1/Fasi194006_G13141.t1/Fasi180275_G11532.t1	22	Accessory groups	MF
GO:0071407	cellular response to organic cyclic compound	13/253	27/4104	1.27E-09	6.39E-08	5.71E-08	Fasi171005_G07405.t1/Fasi180220_G04579.t1/Fasi170382_G04561.t1/Fasi180242_G07569.t1/Fasi171274_G12770.t1/Fasi180192_G10156.t1/Fasi171301_G07643.t1/Fasi180188_G01292.t1/Fasi180242_G12788.t1/Fasi170382_G07580.t1/Fasi180222_G12818.t1/Fasi180104_G08685.t1/Fasi170665_G08985.t1	13	Accessory groups	BP
GO:0005496	steroid binding	12/253	23/4104	1.72E-09	8.44E-08	7.53E-08	Fasi171005_G07405.t1/Fasi180220_G04579.t1/Fasi170382_G04561.t1/Fasi180242_G07569.t1/Fasi171274_G12770.t1/Fasi180192_G10156.t1/Fasi171301_G07643.t1/Fasi180188_G01292.t1/Fasi180242_G12788.t1/Fasi170382_G07580.t1/Fasi180222_G12818.t1/Fasi160242_G04131.t1	12	Accessory groups	MF
GO:1901474	azole transmembrane transporter activity	11/253	19/4104	1.92E-09	8.98E-08	8.01E-08	Fasi171005_G07405.t1/Fasi180220_G04579.t1/Fasi170382_G04561.t1/Fasi180242_G07569.t1/Fasi171274_G12770.t1/Fasi180192_G10156.t1/Fasi171301_G07643.t1/Fasi180188_G01292.t1/Fasi180242_G12788.t1/Fasi170382_G07580.t1/Fasi180222_G12818.t1	11	Accessory groups	MF
GO:0006302	double-strand break repair	27/253	124/4104	3.68E-09	1.68E-07	1.50E-07	Fasi170880_G13549.t1/Fasi170908_G07187.t1/Fasi171330_G13341.t1/Fasi170867_G07532.t1/Fasi170464_G13385.t1/Fasi160280_G13160.t1/Fasi171330_G07612.t1/Fasi170991_G13151.t1/Fasi194008_G13260.t1/Fasi171330_G13468.t1/Fasi140027_G07584.t1/Fa	27	Accessory groups	BP

							si170988_G13513.t1/Fasi140052_G00902.t1/Fasi171115_G03161.t1/Fasi194004_G03166.t1/Fasi171005_G07405.t1/Fasi180220_G04579.t1/Fasi170382_G04561.t1/Fasi180242_G07569.t1/Fasi171274_G12770.t1/Fasi180192_G10156.t1/Fasi171301_G07643.t1/Fasi180188_G01292.t1/Fasi180242_G12788.t1/Fasi170382_G07580.t1/Fasi180222_G12818.t1/Fasi140019_G09559.t1			
GO:0014070	response to organic cyclic compound	13/253	29/4104	3.83E-09	1.70E-07	1.52E-07	Fasi171005_G07405.t1/Fasi180220_G04579.t1/Fasi170382_G04561.t1/Fasi180242_G07569.t1/Fasi171274_G12770.t1/Fasi180192_G10156.t1/Fasi171301_G07643.t1/Fasi180188_G01292.t1/Fasi180242_G12788.t1/Fasi170382_G07580.t1/Fasi180222_G12818.t1/Fasi180104_G08685.t1/Fasi170665_G08985.t1	13	Accessory groups	BP
GO:0045117	azole transmembrane transport	11/253	20/4104	4.04E-09	1.72E-07	1.53E-07	Fasi171005_G07405.t1/Fasi180220_G04579.t1/Fasi170382_G04561.t1/Fasi180242_G07569.t1/Fasi171274_G12770.t1/Fasi180192_G10156.t1/Fasi171301_G07643.t1/Fasi180188_G01292.t1/Fasi180242_G12788.t1/Fasi170382_G07580.t1/Fasi180222_G12818.t1	11	Accessory groups	BP
GO:0043138	3'-5' DNA helicase activity	11/253	22/4104	1.52E-08	6.04E-07	5.39E-07	Fasi170880_G13549.t1/Fasi170908_G07187.t1/Fasi171330_G13341.t1/Fasi170867_G07532.t1/Fasi170464_G13385.t1/Fasi160280_G13160.t1/Fasi171330_G07612.t1/Fasi170991_G13151.t1/Fasi194008_G13260.t1/Fasi171330_G13468.t1/Fasi140027_G07584.t1	11	Accessory groups	MF
GO:0045332	phospholipid translocation	11/253	22/4104	1.52E-08	6.04E-07	5.39E-07	Fasi171005_G07405.t1/Fasi180220_G04579.t1/Fasi170382_G04561.t1/Fasi180242_G07569.t1/Fasi171274_G12770.t1/Fasi180192_G10156.t1/Fasi171301_G07643.t1/Fasi180188_G01292.t1/Fasi180242_G12788.t1/Fasi170382_G07580.t1/Fasi180222_G12818.t1	11	Accessory groups	BP
GO:0016772	transferase activity, transferring phosphorus-containing groups	43/253	288/4104	1.96E-08	7.61E-07	6.79E-07	Fasi170343_G00559.t2/Fasi180269_G10003.t1/Fasi140052_G00902.t1/Fasi180283_G08052.t1/Fasi191882_G09425.t1/Fasi171252_G11327.t1/Fasi170788_G13123.t1/Fasi180363_G13001.t1/Fasi180341_G05623.t1/Fasi171646_G13245.t1/Fasi160270_G07411.t1/Fasi171042_G07537.t1/Fasi180099_G07564.t1/Fasi140011_G10419.t1/Fasi194010_G07522.t1/Fasi171308_G13331.t1/Fasi172120_G10389.t1/Fasi180188_G00003.t1/Fasi170460_G13497.t1/Fasi160226_G07976.t1/Fasi180341_G07680.t1/Fasi181006_G07603.t1/Fasi171290_G07966.t1/Fasi140006_G10434.t1/Fasi171669_G13242.t1/Fasi170908_G13312.t1/Fasi170728_G01973.t1/Fasi193963_G13202.t1/Fasi180648_G07580.t1/Fasi171025_G13092.t1/Fasi171067_G00804.t1/Fasi160277_G07744.t1/Fasi180273_G13260.t1/Fasi180	43	Accessory groups	MF

							106_G10511.t1/Fasi170908_G13535.t1/Fasi171330_G07104.t1/Fasi170988_G13320.t1/Fasi140025_G13363.t1/Fasi171330_G13407.t1/Fasi140025_G13424.t1/Fasi180220_G13201.t1/Fasi180099_G10411.t1/Fasi193963_G13201.t1			
GO:0034204	lipid translocation	11/253	23/4104	2.75E-08	1.05E-06	9.37E-07	Fasi171005_G07405.t1/Fasi180220_G04579.t1/Fasi170382_G04561.t1/Fasi180242_G07569.t1/Fasi171274_G12770.t1/Fasi180192_G10156.t1/Fasi171301_G07643.t1/Fasi180188_G01292.t1/Fasi180242_G12788.t1/Fasi170382_G07580.t1/Fasi180222_G12818.t1	11	Accessory groups	BP
GO:0005548	phospholipid transporter activity	11/253	24/4104	4.81E-08	1.69E-06	1.51E-06	Fasi171005_G07405.t1/Fasi180220_G04579.t1/Fasi170382_G04561.t1/Fasi180242_G07569.t1/Fasi171274_G12770.t1/Fasi180192_G10156.t1/Fasi171301_G07643.t1/Fasi180188_G01292.t1/Fasi180242_G12788.t1/Fasi170382_G07580.t1/Fasi180222_G12818.t1	11	Accessory groups	MF
GO:0045121	membrane raft	11/253	24/4104	4.81E-08	1.69E-06	1.51E-06	Fasi171005_G07405.t1/Fasi180220_G04579.t1/Fasi170382_G04561.t1/Fasi180242_G07569.t1/Fasi171274_G12770.t1/Fasi180192_G10156.t1/Fasi171301_G07643.t1/Fasi180188_G01292.t1/Fasi180242_G12788.t1/Fasi170382_G07580.t1/Fasi180222_G12818.t1	11	Accessory groups	CC
GO:0098857	membrane microdomain	11/253	24/4104	4.81E-08	1.69E-06	1.51E-06	Fasi171005_G07405.t1/Fasi180220_G04579.t1/Fasi170382_G04561.t1/Fasi180242_G07569.t1/Fasi171274_G12770.t1/Fasi180192_G10156.t1/Fasi171301_G07643.t1/Fasi180188_G01292.t1/Fasi180242_G12788.t1/Fasi170382_G07580.t1/Fasi180222_G12818.t1	11	Accessory groups	CC
GO:0019748	secondary metabolic process	17/253	63/4104	1.23E-07	4.24E-06	3.78E-06	Fasi171642_G07469.t1/Fasi180249_G02296.t1/Fasi140026_G04030.t1/Fasi171301_G04059.t1/Fasi140013_G08044.t1/Fasi160242_G10513.t1/Fasi170382_G07571.t1/Fasi171470_G07274.t1/Fasi171658_G10961.t1/Fasi140011_G02301.t1/Fasi170960_G01915.t1/Fasi170705_G02450.t1/Fasi160863_G02276.t1/Fasi140011_G02303.t1/Fasi194004_G02299.t1/Fasi160898_G02315.t1/Fasi171108_G02307.t1	17	Accessory groups	BP
GO:0015850	organic hydroxy compound transport	13/253	37/4104	1.29E-07	4.37E-06	3.90E-06	Fasi171005_G07405.t1/Fasi180220_G04579.t1/Fasi170382_G04561.t1/Fasi180242_G07569.t1/Fasi180341_G05926.t1/Fasi171274_G12770.t1/Fasi180192_G10156.t1/Fasi171301_G07643.t1/Fasi180188_G01292.t1/Fasi180242_G12788.t1/Fasi170382_G07580.t1/Fasi180222_G12818.t1/Fasi160242_G04131.t1	13	Accessory groups	BP
GO:0097035	regulation of membrane lipid distribution	11/253	26/4104	1.33E-07	4.44E-06	3.96E-06	Fasi171005_G07405.t1/Fasi180220_G04579.t1/Fasi170382_G04561.t1/Fasi180242_G07569.t1/Fasi171274_G12770.t1/Fasi180192_G10156.t1/Fasi171301_G07643.t1/Fasi180188_G01292.t1/Fasi180	11	Accessory groups	BP

							242_G12788.t1/Fasi170382_G07580.t1/Fasi180222_G12818.t1			
GO:0006310	DNA recombination	26/253	142/4104	3.09E-07	1.01E-05	9.03E-06	Fasi170880_G13549.t1/Fasi170908_G07187.t1/Fasi171330_G13341.t1/Fasi170867_G07532.t1/Fasi170464_G13385.t1/Fasi160280_G13160.t1/Fasi171330_G07612.t1/Fasi170991_G13151.t1/Fasi194008_G13260.t1/Fasi171330_G13468.t1/Fasi140027_G07584.t1/Fasi170988_G13513.t1/Fasi171115_G03161.t1/Fasi194004_G03166.t1/Fasi171005_G07405.t1/Fasi180220_G04579.t1/Fasi170382_G04561.t1/Fasi180242_G07569.t1/Fasi171274_G12770.t1/Fasi180192_G10156.t1/Fasi171301_G07643.t1/Fasi180188_G01292.t1/Fasi180242_G12788.t1/Fasi170382_G07580.t1/Fasi180222_G12818.t1/Fasi140019_G09559.t1	26	Accessory groups	BP
GO:0009719	response to endogenous stimulus	15/253	53/4104	3.52E-07	1.11E-05	9.93E-06	Fasi171005_G07405.t1/Fasi180220_G04579.t1/Fasi170382_G04561.t1/Fasi180242_G07569.t1/Fasi171274_G12770.t1/Fasi180192_G10156.t1/Fasi171301_G07643.t1/Fasi180188_G01292.t1/Fasi180242_G12788.t1/Fasi170382_G07580.t1/Fasi180222_G12818.t1/Fasi170665_G08985.t1/Fasi181150_G09801.t1/Fasi170677_G13519.t1/Fasi170464_G07649.t1	15	Accessory groups	BP
GO:0071495	cellular response to endogenous stimulus	15/253	53/4104	3.52E-07	1.11E-05	9.93E-06	Fasi171005_G07405.t1/Fasi180220_G04579.t1/Fasi170382_G04561.t1/Fasi180242_G07569.t1/Fasi171274_G12770.t1/Fasi180192_G10156.t1/Fasi171301_G07643.t1/Fasi180188_G01292.t1/Fasi180242_G12788.t1/Fasi170382_G07580.t1/Fasi180222_G12818.t1/Fasi170665_G08985.t1/Fasi181150_G09801.t1/Fasi170677_G13519.t1/Fasi170464_G07649.t1	15	Accessory groups	BP
GO:0006855	xenobiotic transmembrane transport	18/253	76/4104	4.47E-07	1.39E-05	1.24E-05	Fasi171005_G07405.t1/Fasi180220_G04579.t1/Fasi170382_G04561.t1/Fasi180242_G07569.t1/Fasi170719_G06635.t1/Fasi180222_G06592.t1/Fasi171274_G12770.t1/Fasi180192_G10156.t1/Fasi171301_G07643.t1/Fasi180188_G01292.t1/Fasi180242_G12788.t1/Fasi170382_G07580.t1/Fasi180222_G12818.t1/Fasi170343_G13059.t1/Fasi180281_G09711.t1/Fasi181100_G10113.t1/Fasi160270_G10105.t1/Fasi180275_G11532.t1	18	Accessory groups	BP
GO:0016887	ATP hydrolysis activity	38/253	266/4104	4.81E-07	1.47E-05	1.31E-05	Fasi170880_G13549.t1/Fasi170908_G07187.t1/Fasi171330_G13341.t1/Fasi170867_G07532.t1/Fasi170464_G13385.t1/Fasi160280_G13160.t1/Fasi171330_G07612.t1/Fasi170991_G13151.t1/Fasi194008_G13260.t1/Fasi171330_G13468.t1/Fasi140027_G07584.t1/Fasi171115_G03161.t1/Fasi194004_G03166.t1/Fasi171075_G07733.	38	Accessory groups	MF

							t1/Fasi171005_G07405.t1/Fasi180220_G04579.t1/Fasi170382_G04561.t1/Fasi180242_G07569.t1/Fasi170719_G06635.t1/Fasi180222_G06592.t1/Fasi171274_G12770.t1/Fasi180192_G10156.t1/Fasi171301_G07643.t1/Fasi180188_G01292.t1/Fasi180242_G12788.t1/Fasi170382_G07580.t1/Fasi180222_G12818.t1/Fasi180250_G07688.t1/Fasi171483_G13319.t1/Fasi180281_G09711.t1/Fasi181100_G10113.t1/Fasi180104_G10679.t1/Fasi180279_G10743.t1/Fasi170880_G13357.t1/Fasi170460_G13556.t1/Fasi160270_G10105.t1/Fasi194006_G13141.t1/Fasi180275_G11532.t1			
GO:0000722	telomere maintenance via recombination	11/253	29/4104	5.05E-07	1.47E-05	1.31E-05	Fasi170880_G13549.t1/Fasi170908_G07187.t1/Fasi171330_G13341.t1/Fasi170867_G07532.t1/Fasi170464_G13385.t1/Fasi160280_G13160.t1/Fasi171330_G07612.t1/Fasi170991_G13151.t1/Fasi194008_G13260.t1/Fasi171330_G13468.t1/Fasi140027_G07584.t1	11	Accessory groups	BP
GO:0015914	phospholipid transport	11/253	29/4104	5.05E-07	1.47E-05	1.31E-05	Fasi171005_G07405.t1/Fasi180220_G04579.t1/Fasi170382_G04561.t1/Fasi180242_G07569.t1/Fasi171274_G12770.t1/Fasi180192_G10156.t1/Fasi171301_G07643.t1/Fasi180188_G01292.t1/Fasi180242_G12788.t1/Fasi170382_G07580.t1/Fasi180222_G12818.t1	11	Accessory groups	BP
GO:0071396	cellular response to lipid	11/253	29/4104	5.05E-07	1.47E-05	1.31E-05	Fasi171005_G07405.t1/Fasi180220_G04579.t1/Fasi170382_G04561.t1/Fasi180242_G07569.t1/Fasi171274_G12770.t1/Fasi180192_G10156.t1/Fasi171301_G07643.t1/Fasi180188_G01292.t1/Fasi180242_G12788.t1/Fasi170382_G07580.t1/Fasi180222_G12818.t1	11	Accessory groups	BP
GO:0005319	lipid transporter activity	12/253	35/4104	5.43E-07	1.56E-05	1.39E-05	Fasi171005_G07405.t1/Fasi180220_G04579.t1/Fasi170382_G04561.t1/Fasi180242_G07569.t1/Fasi171274_G12770.t1/Fasi180192_G10156.t1/Fasi171301_G07643.t1/Fasi180188_G01292.t1/Fasi180242_G12788.t1/Fasi170382_G07580.t1/Fasi180222_G12818.t1/Fasi160242_G04131.t1	12	Accessory groups	MF
GO:0033993	response to lipid	11/253	30/4104	7.54E-07	2.13E-05	1.90E-05	Fasi171005_G07405.t1/Fasi180220_G04579.t1/Fasi170382_G04561.t1/Fasi180242_G07569.t1/Fasi171274_G12770.t1/Fasi180192_G10156.t1/Fasi171301_G07643.t1/Fasi180188_G01292.t1/Fasi180242_G12788.t1/Fasi170382_G07580.t1/Fasi180222_G12818.t1	11	Accessory groups	BP
GO:0009986	cell surface	17/253	74/4104	1.50E-06	4.11E-05	3.67E-05	Fasi170788_G01663.t1/Fasi171639_G01882.t1/Fasi171005_G07405.t1/Fasi180220_G04579.t1/Fasi170382_G04561.t1/Fasi180242_G07569.t1/Fasi171244_G07424.t1/Fasi171274_G12770.t1/Fasi180192_G10156.t1/Fasi171301_G07643.t1/Fasi180188_G01292.t1/Fasi180242_G12788.t1/Fasi170382_G07580.t1/Fasi180222_G12818.	17	Accessory groups	CC

							t1/Fasi171330_G07667.t1/Fasi170728_G01944.t1/Fasi170460_G10561.t1			
GO:0022804	active transmembrane transporter activity	23/253	137/4104	7.28E-06	0.000196923	0.000175756	Fasi171075_G07733.t1/Fasi171005_G07405.t1/Fasi180220_G04579.t1/Fasi170382_G04561.t1/Fasi180242_G07569.t1/Fasi170988_G13462.t1/Fasi170719_G06635.t1/Fasi180222_G06592.t1/Fasi171274_G12770.t1/Fasi180192_G10156.t1/Fasi171301_G07643.t1/Fasi180188_G01292.t1/Fasi180242_G12788.t1/Fasi170382_G07580.t1/Fasi180222_G12818.t1/Fasi180281_G09711.t1/Fasi181100_G10113.t1/Fasi180104_G10679.t1/Fasi180279_G10743.t1/Fasi170460_G13556.t1/Fasi160270_G10105.t1/Fasi194006_G13141.t1/Fasi180275_G11532.t1	23	Accessory groups	MF
GO:0034654	nucleobase-containing compound biosynthetic process	47/253	404/4104	8.21E-06	0.000219082	0.000195533	Fasi171330_G00515.t1/Fasi140052_G00902.t1/Fasi170364_G01638.t1/Fasi180222_G05219.t1/Fasi180219_G07541.t1/Fasi170430_G07497.t1/Fasi180104_G08685.t1/Fasi180104_G10679.t1/Fasi171663_G10692.t1/Fasi171645_G10724.t1/Fasi180279_G10743.t1/Fasi170382_G11119.t1/Fasi171252_G11327.t1/Fasi180341_G11247.t1/Fasi180341_G05623.t1/Fasi171646_G13245.t1/Fasi160270_G07411.t1/Fasi171042_G07537.t1/Fasi180099_G07564.t1/Fasi140011_G10419.t1/Fasi194010_G07522.t1/Fasi171308_G13331.t1/Fasi172120_G10389.t1/Fasi180188_G00003.t1/Fasi170460_G13497.t1/Fasi160226_G07976.t1/Fasi180341_G07680.t1/Fasi181006_G07603.t1/Fasi171290_G07966.t1/Fasi140006_G10434.t1/Fasi171669_G13242.t1/Fasi170908_G13312.t1/Fasi193963_G13202.t1/Fasi180648_G07580.t1/Fasi140006_G12678.t1/Fasi160277_G07744.t1/Fasi180273_G13260.t1/Fasi180106_G10511.t1/Fasi160280_G00505.t1/Fasi181150_G09801.t1/Fasi140025_G13363.t1/Fasi170464_G07649.t1/Fasi180220_G13201.t1/Fasi180099_G10411.t1/Fasi171669_G10431.t1/Fasi193963_G13201.t1/Fasi171377_G07341.t1	47	Accessory groups	BP
GO:0030287	cell wall-bounded periplasmic space	6/253	10/4104	8.82E-06	0.000231856	0.000206934	Fasi171330_G07667.t1/Fasi170728_G01944.t1/Fasi170464_G13604.t1/Fasi170908_G13465.t1/Fasi140025_G13488.t1/Fasi170460_G10561.t1	6	Accessory groups	CC
GO:0042592	homeostatic process	38/253	300/4104	9.81E-06	0.000254487	0.000227132	Fasi170880_G13549.t1/Fasi170908_G07187.t1/Fasi171330_G13341.t1/Fasi170867_G07532.t1/Fasi170464_G13385.t1/Fasi160280_G13160.t1/Fasi171330_G07612.t1/Fasi170991_G13151.t1/Fasi194	38	Accessory groups	BP

							008_G13260.t1/Fasi171330_G13468.t1/Fasi140027_G07584.t1/Fasi171075_G02705.t1/Fasi180101_G02972.t1/Fasi171115_G03161.t1/Fasi194004_G03166.t1/Fasi171075_G07733.t1/Fasi171005_G07405.t1/Fasi180220_G04579.t1/Fasi170382_G04561.t1/Fasi180242_G07569.t1/Fasi170988_G13462.t1/Fasi170908_G06273.t1/Fasi171025_G08213.t1/Fasi171274_G12770.t1/Fasi180192_G10156.t1/Fasi171301_G07643.t1/Fasi180188_G01292.t1/Fasi180242_G12788.t1/Fasi170382_G07580.t1/Fasi180222_G12818.t1/Fasi170784_G09614.t1/Fasi170705_G10893.t1/Fasi160270_G09528.t1/Fasi171205_G09498.t2/Fasi170464_G07649.t1/Fasi171484_G13024.t1/Fasi194006_G13141.t1/Fasi180275_G11532.t1			
GO:0006869	lipid transport	13/253	53/4104	1.24E-05	0.000318171	0.000283971	Fasi171075_G02705.t1/Fasi171005_G07405.t1/Fasi180220_G04579.t1/Fasi170382_G04561.t1/Fasi180242_G07569.t1/Fasi171274_G12770.t1/Fasi180192_G10156.t1/Fasi171301_G07643.t1/Fasi180188_G01292.t1/Fasi180242_G12788.t1/Fasi170382_G07580.t1/Fasi180222_G12818.t1/Fasi160242_G04131.t1	13	Accessory groups	BP
GO:0006359	regulation of transcription by RNA polymerase III	7/253	15/4104	1.31E-05	0.000325717	0.000290705	Fasi171030_G03430.t1/Fasi160277_G03611.t2/Fasi170908_G13535.t1/Fasi171330_G07104.t1/Fasi170988_G13320.t1/Fasi171330_G13407.t1/Fasi140025_G13424.t1	7	Accessory groups	BP
GO:0042597	periplasmic space	7/253	15/4104	1.31E-05	0.000325717	0.000290705	Fasi171244_G07424.t1/Fasi171330_G07667.t1/Fasi170728_G01944.t1/Fasi170464_G13604.t1/Fasi170908_G13465.t1/Fasi140025_G13488.t1/Fasi170460_G10561.t1	7	Accessory groups	CC
GO:0003678	DNA helicase activity	11/253	42/4104	3.04E-05	0.000737106	0.000657874	Fasi170880_G13549.t1/Fasi170908_G07187.t1/Fasi171330_G13341.t1/Fasi170867_G07532.t1/Fasi170464_G13385.t1/Fasi160280_G13160.t1/Fasi171330_G07612.t1/Fasi170991_G13151.t1/Fasi194008_G13260.t1/Fasi171330_G13468.t1/Fasi140027_G07584.t1	11	Accessory groups	MF
GO:0010876	lipid localization	13/253	58/4104	3.53E-05	0.000834522	0.000744819	Fasi171075_G02705.t1/Fasi171005_G07405.t1/Fasi180220_G04579.t1/Fasi170382_G04561.t1/Fasi180242_G07569.t1/Fasi171274_G12770.t1/Fasi180192_G10156.t1/Fasi171301_G07643.t1/Fasi180188_G01292.t1/Fasi180242_G12788.t1/Fasi170382_G07580.t1/Fasi180222_G12818.t1/Fasi160242_G04131.t1	13	Accessory groups	BP
GO:0019237	centromeric DNA binding	6/253	12/4104	3.49E-05	0.000834522	0.000744819	Fasi180242_G08398.t1/Fasi194008_G08244.t1/Fasi180269_G07857.t1/Fasi171643_G08222.t1/Fasi171206_G08196.t1/Fasi170464_G07649.t1	6	Accessory groups	MF

GO:0006312	mitotic recombination	11/253	43/4104	3.87E-05	0.000902046	0.000805085	Fasi170880_G13549.t1/Fasi170908_G07187.t1/Fasi171330_G13341.t1/Fasi170867_G07532.t1/Fasi170464_G13385.t1/Fasi160280_G13160.t1/Fasi171330_G07612.t1/Fasi170991_G13151.t1/Fasi194008_G13260.t1/Fasi171330_G13468.t1/Fasi140027_G07584.t1	11	Accessory groups	BP
GO:0000723	telomere maintenance	14/253	68/4104	4.87E-05	0.001107947	0.000988854	Fasi170880_G13549.t1/Fasi170908_G07187.t1/Fasi171330_G13341.t1/Fasi170867_G07532.t1/Fasi170464_G13385.t1/Fasi160280_G13160.t1/Fasi171330_G07612.t1/Fasi170991_G13151.t1/Fasi194008_G13260.t1/Fasi171330_G13468.t1/Fasi140027_G07584.t1/Fasi171115_G03161.t1/Fasi194004_G03166.t1/Fasi171484_G13024.t1	14	Accessory groups	BP
GO:0032200	telomere organization	14/253	68/4104	4.87E-05	0.001107947	0.000988854	Fasi170880_G13549.t1/Fasi170908_G07187.t1/Fasi171330_G13341.t1/Fasi170867_G07532.t1/Fasi170464_G13385.t1/Fasi160280_G13160.t1/Fasi171330_G07612.t1/Fasi170991_G13151.t1/Fasi194008_G13260.t1/Fasi171330_G13468.t1/Fasi140027_G07584.t1/Fasi171115_G03161.t1/Fasi194004_G03166.t1/Fasi171484_G13024.t1	14	Accessory groups	BP
GO:0072329	monocarboxylic acid catabolic process	12/253	52/4104	5.22E-05	0.001173761	0.001047593	Fasi171644_G06563.t1/Fasi174240_G10350.t1/Fasi140013_G08044.t1/Fasi160242_G10513.t1/Fasi170382_G07571.t1/Fasi171470_G07274.t1/Fasi170460_G13465.t1/Fasi171330_G07667.t1/Fasi171476_G10186.t1/Fasi170728_G01944.t1/Fasi170460_G10561.t1/Fasi180269_G07174.t1	12	Accessory groups	BP
GO:0060249	anatomical structure homeostasis	14/253	69/4104	5.77E-05	0.001283222	0.001145288	Fasi170880_G13549.t1/Fasi170908_G07187.t1/Fasi171330_G13341.t1/Fasi170867_G07532.t1/Fasi170464_G13385.t1/Fasi160280_G13160.t1/Fasi171330_G07612.t1/Fasi170991_G13151.t1/Fasi194008_G13260.t1/Fasi171330_G13468.t1/Fasi140027_G07584.t1/Fasi171115_G03161.t1/Fasi194004_G03166.t1/Fasi171484_G13024.t1	14	Accessory groups	BP
GO:0006281	DNA repair	32/253	256/4104	6.97E-05	0.001530098	0.001365627	Fasi170880_G13549.t1/Fasi170908_G07187.t1/Fasi171330_G13341.t1/Fasi170867_G07532.t1/Fasi170464_G13385.t1/Fasi160280_G13160.t1/Fasi171330_G07612.t1/Fasi170991_G13151.t1/Fasi194008_G13260.t1/Fasi171330_G13468.t1/Fasi140027_G07584.t1/Fasi171330_G00515.t1/Fasi170988_G13513.t1/Fasi140052_G00902.t1/Fasi170677_G10032.t1/Fasi171115_G03161.t1/Fasi194004_G03166.t1/Fasi171005_G07405.t1/Fasi180220_G04579.t1/Fasi170382_G04561.t1/Fasi180242_G07569.t1/Fasi180222_G05219.t1/Fasi1	32	Accessory groups	BP

							71274_G12770.t1/Fasi180192_G10156.t1/Fasi171301_G07643.t1/Fasi180188_G01292.t1/Fasi180242_G12788.t1/Fasi170382_G07580.t1/Fasi180222_G12818.t1/Fasi140019_G09559.t1/Fasi180341_G11247.t1/Fasi160280_G00505.t1			
GO:0009068	aspartate family amino acid catabolic process	6/253	14/4104	0.000102098	0.002190992	0.001955482	Fasi140024_G05774.t1/Fasi170460_G13465.t1/Fasi171330_G07667.t1/Fasi171476_G10186.t1/Fasi170728_G01944.t1/Fasi170460_G10561.t1	6	Accessory groups	BP
GO:0032392	DNA geometric change	12/253	57/4104	0.000135521	0.002875207	0.00256615	Fasi170880_G13549.t1/Fasi170908_G07187.t1/Fasi171330_G13341.t1/Fasi170867_G07532.t1/Fasi170464_G13385.t1/Fasi160280_G13160.t1/Fasi171330_G07612.t1/Fasi170991_G13151.t1/Fasi194008_G13260.t1/Fasi171330_G13468.t1/Fasi140027_G07584.t1/Fasi140019_G09559.t1	12	Accessory groups	BP
GO:0017111	ribonucleoside triphosphate phosphatase activity	38/253	337/4104	0.000137063	0.002875251	0.00256619	Fasi170880_G13549.t1/Fasi170908_G07187.t1/Fasi171330_G13341.t1/Fasi170867_G07532.t1/Fasi170464_G13385.t1/Fasi160280_G13160.t1/Fasi171330_G07612.t1/Fasi170991_G13151.t1/Fasi194008_G13260.t1/Fasi171330_G13468.t1/Fasi140027_G07584.t1/Fasi171115_G03161.t1/Fasi194004_G03166.t1/Fasi171075_G07733.t1/Fasi171005_G07405.t1/Fasi180220_G04579.t1/Fasi170382_G04561.t1/Fasi180242_G07569.t1/Fasi170719_G06635.t1/Fasi180222_G06592.t1/Fasi171274_G12770.t1/Fasi180192_G10156.t1/Fasi171301_G07643.t1/Fasi180188_G01292.t1/Fasi180242_G12788.t1/Fasi170382_G07580.t1/Fasi180222_G12818.t1/Fasi180250_G07688.t1/Fasi171483_G13319.t1/Fasi180281_G09711.t1/Fasi181100_G10113.t1/Fasi180104_G10679.t1/Fasi180279_G10743.t1/Fasi170880_G13357.t1/Fasi170460_G13556.t1/Fasi160270_G10105.t1/Fasi194006_G13141.t1/Fasi180275_G11532.t1	38	Accessory groups	MF
GO:0032508	DNA duplex unwinding	11/253	49/4104	0.000140547	0.002915563	0.002602168	Fasi170880_G13549.t1/Fasi170908_G07187.t1/Fasi171330_G13341.t1/Fasi170867_G07532.t1/Fasi170464_G13385.t1/Fasi160280_G13160.t1/Fasi171330_G07612.t1/Fasi170991_G13151.t1/Fasi194008_G13260.t1/Fasi171330_G13468.t1/Fasi140027_G07584.t1	11	Accessory groups	BP
GO:0006528	asparagine metabolic process	5/253	10/4104	0.000167003	0.003426306	0.003058011	Fasi170460_G13465.t1/Fasi171330_G07667.t1/Fasi171476_G10186.t1/Fasi170728_G01944.t1/Fasi170460_G10561.t1	5	Accessory groups	BP
GO:0019438	aromatic	48/253	470/41	0.0002	0.00404	0.0036120	Fasi171330_G00515.t1/Fasi140052_G00902.t1/Fasi170364_G016	48	Accessory	BP

	compound biosynthetic process		04	01596	7101	78	38.t1/Fasi180222_G05219.t1/Fasi180219_G07541.t1/Fasi170430_G07497.t1/Fasi180104_G08685.t1/Fasi180104_G10679.t1/Fasi171663_G10692.t1/Fasi171645_G10724.t1/Fasi180279_G10743.t1/Fasi170382_G11119.t1/Fasi171252_G11327.t1/Fasi180341_G11247.t1/Fasi180341_G05623.t1/Fasi171646_G13245.t1/Fasi160270_G07411.t1/Fasi171042_G07537.t1/Fasi180099_G07564.t1/Fasi140011_G10419.t1/Fasi194010_G07522.t1/Fasi171308_G13331.t1/Fasi172120_G10389.t1/Fasi180188_G00003.t1/Fasi170460_G13497.t1/Fasi160226_G07976.t1/Fasi180341_G07680.t1/Fasi181006_G07603.t1/Fasi171290_G07966.t1/Fasi140006_G10434.t1/Fasi171669_G13242.t1/Fasi170908_G13312.t1/Fasi193963_G13202.t1/Fasi180648_G07580.t1/Fasi140006_G12678.t1/Fasi160277_G07744.t1/Fasi180273_G13260.t1/Fasi180106_G10511.t1/Fasi160280_G00505.t1/Fasi181150_G09801.t1/Fasi174251_G07361.t1/Fasi140025_G13363.t1/Fasi170464_G07649.t1/Fasi180220_G13201.t1/Fasi180099_G10411.t1/Fasi171669_G10431.t1/Fasi193963_G13201.t1/Fasi171377_G07341.t1		groups	
GO:0008236	serine-type peptidase activity	7/253	22/4104	0.000238397	0.004734971	0.004226008	Fasi170545_G09975.t1/Fasi180363_G04135.t1/Fasi181104_G10919.t1/Fasi171658_G10961.t1/Fasi170464_G13604.t1/Fasi170908_G13465.t1/Fasi140025_G13488.t1	7	Accessory groups	MF
GO:0004713	protein tyrosine kinase activity	5/253	11/4104	0.000290838	0.00559789	0.004996172	Fasi170908_G13535.t1/Fasi171330_G07104.t1/Fasi170988_G13320.t1/Fasi171330_G13407.t1/Fasi140025_G13424.t1	5	Accessory groups	MF
GO:0018108	peptidyl-tyrosine phosphorylation	5/253	11/4104	0.000290838	0.00559789	0.004996172	Fasi170908_G13535.t1/Fasi171330_G07104.t1/Fasi170988_G13320.t1/Fasi171330_G13407.t1/Fasi140025_G13424.t1	5	Accessory groups	BP
GO:0033120	positive regulation of RNA splicing	5/253	11/4104	0.000290838	0.00559789	0.004996172	Fasi170908_G13535.t1/Fasi171330_G07104.t1/Fasi170988_G13320.t1/Fasi171330_G13407.t1/Fasi140025_G13424.t1	5	Accessory groups	BP
GO:0017171	serine hydrolase activity	7/253	23/4104	0.000324889	0.006189466	0.005524159	Fasi170545_G09975.t1/Fasi180363_G04135.t1/Fasi181104_G10919.t1/Fasi171658_G10961.t1/Fasi170464_G13604.t1/Fasi170908_G13465.t1/Fasi140025_G13488.t1	7	Accessory groups	MF
GO:0018130	heterocycle biosynthetic process	49/253	494/4104	0.000349552	0.00659205	0.005883469	Fasi171330_G00515.t1/Fasi140052_G00902.t1/Fasi171642_G07469.t1/Fasi170364_G01638.t1/Fasi180222_G05219.t1/Fasi180219_G07541.t1/Fasi170430_G07497.t1/Fasi180104_G08685.t1/Fasi180104_G10679.t1/Fasi171663_G10692.t1/Fasi171645_G10724.t1/Fa	49	Accessory groups	BP

							si180279_G10743.t1/Fasi170382_G11119.t1/Fasi171252_G11327.t1/Fasi180341_G11247.t1/Fasi180341_G05623.t1/Fasi171646_G13245.t1/Fasi160270_G07411.t1/Fasi171042_G07537.t1/Fasi180099_G07564.t1/Fasi140011_G10419.t1/Fasi194010_G07522.t1/Fasi171308_G13331.t1/Fasi172120_G10389.t1/Fasi180188_G00003.t1/Fasi170460_G13497.t1/Fasi160226_G07976.t1/Fasi180341_G07680.t1/Fasi181006_G07603.t1/Fasi171290_G07966.t1/Fasi140006_G10434.t1/Fasi171669_G13242.t1/Fasi170908_G13312.t1/Fasi193963_G13202.t1/Fasi180648_G07580.t1/Fasi140006_G12678.t1/Fasi160277_G07744.t1/Fasi180273_G13260.t1/Fasi180106_G10511.t1/Fasi160280_G00505.t1/Fasi181150_G09801.t1/Fasi174251_G07361.t1/Fasi140025_G13363.t1/Fasi170464_G07649.t1/Fasi180220_G13201.t1/Fasi180099_G10411.t1/Fasi171669_G10431.t1/Fasi193963_G13201.t1/Fasi171377_G07341.t1			
GO:0000430	regulation of transcription from RNA polymerase II promoter by glucose	6/253	17/4104	0.000359378	0.006709592	0.005988377	Fasi170908_G13535.t1/Fasi171330_G07104.t1/Fasi170988_G13320.t1/Fasi171330_G13407.t1/Fasi140025_G13424.t1/Fasi170464_G07649.t1	6	Accessory groups	BP
GO:0006979	response to oxidative stress	20/253	142/4104	0.000364096	0.006730372	0.006006923	Fasi171005_G07405.t1/Fasi180220_G04579.t1/Fasi170382_G04561.t1/Fasi180242_G07569.t1/Fasi170908_G06273.t1/Fasi171274_G12770.t1/Fasi180192_G10156.t1/Fasi171301_G07643.t1/Fasi180188_G01292.t1/Fasi180242_G12788.t1/Fasi170382_G07580.t1/Fasi180222_G12818.t1/Fasi170665_G08985.t1/Fasi180267_G08932.t1/Fasi180271_G13241.t1/Fasi170908_G13535.t1/Fasi171330_G07104.t1/Fasi170988_G13320.t1/Fasi171330_G13407.t1/Fasi140025_G13424.t1	20	Accessory groups	BP
GO:0016462	pyrophosphatase activity	38/253	355/4104	0.000405354	0.007276875	0.006494682	Fasi170880_G13549.t1/Fasi170908_G07187.t1/Fasi171330_G13341.t1/Fasi170867_G07532.t1/Fasi170464_G13385.t1/Fasi160280_G13160.t1/Fasi171330_G07612.t1/Fasi170991_G13151.t1/Fasi194008_G13260.t1/Fasi171330_G13468.t1/Fasi140027_G07584.t1/Fasi171115_G03161.t1/Fasi194004_G03166.t1/Fasi171075_G07733.t1/Fasi171005_G07405.t1/Fasi180220_G04579.t1/Fasi170382_G04561.t1/Fasi180242_G07569.t1/Fasi170719_G06635.t1/Fasi18022	38	Accessory groups	MF

							2_G06592.t1/Fasi171274_G12770.t1/Fasi180192_G10156.t1/Fasi171301_G07643.t1/Fasi180188_G01292.t1/Fasi180242_G12788.t1/Fasi170382_G07580.t1/Fasi180222_G12818.t1/Fasi180250_G07688.t1/Fasi171483_G13319.t1/Fasi180281_G09711.t1/Fasi181100_G10113.t1/Fasi180104_G10679.t1/Fasi180279_G10743.t1/Fasi170880_G13357.t1/Fasi170460_G13556.t1/Fasi160270_G10105.t1/Fasi194006_G13141.t1/Fasi180275_G11532.t1			
GO:0016817	hydrolase activity, acting on acid anhydrides	38/253	355/4104	0.000405354	0.007276875	0.006494682	Fasi170880_G13549.t1/Fasi170908_G07187.t1/Fasi171330_G13341.t1/Fasi170867_G07532.t1/Fasi170464_G13385.t1/Fasi160280_G13160.t1/Fasi171330_G07612.t1/Fasi170991_G13151.t1/Fasi194008_G13260.t1/Fasi171330_G13468.t1/Fasi140027_G07584.t1/Fasi171115_G03161.t1/Fasi194004_G03166.t1/Fasi171075_G07733.t1/Fasi171005_G07405.t1/Fasi180220_G04579.t1/Fasi170382_G04561.t1/Fasi180242_G07569.t1/Fasi170719_G06635.t1/Fasi180222_G06592.t1/Fasi171274_G12770.t1/Fasi180192_G10156.t1/Fasi171301_G07643.t1/Fasi180188_G01292.t1/Fasi180242_G12788.t1/Fasi170382_G07580.t1/Fasi180222_G12818.t1/Fasi180250_G07688.t1/Fasi171483_G13319.t1/Fasi180281_G09711.t1/Fasi181100_G10113.t1/Fasi180104_G10679.t1/Fasi180279_G10743.t1/Fasi170880_G13357.t1/Fasi170460_G13556.t1/Fasi160270_G10105.t1/Fasi194006_G13141.t1/Fasi180275_G11532.t1	38	Accessory groups	MF
GO:0016818	hydrolase activity, acting on acid anhydrides, in phosphorus-containing anhydrides	38/253	355/4104	0.000405354	0.007276875	0.006494682	Fasi170880_G13549.t1/Fasi170908_G07187.t1/Fasi171330_G13341.t1/Fasi170867_G07532.t1/Fasi170464_G13385.t1/Fasi160280_G13160.t1/Fasi171330_G07612.t1/Fasi170991_G13151.t1/Fasi194008_G13260.t1/Fasi171330_G13468.t1/Fasi140027_G07584.t1/Fasi171115_G03161.t1/Fasi194004_G03166.t1/Fasi171075_G07733.t1/Fasi171005_G07405.t1/Fasi180220_G04579.t1/Fasi170382_G04561.t1/Fasi180242_G07569.t1/Fasi170719_G06635.t1/Fasi180222_G06592.t1/Fasi171274_G12770.t1/Fasi180192_G10156.t1/Fasi171301_G07643.t1/Fasi180188_G01292.t1/Fasi180242_G12788.t1/Fasi170382_G07580.t1/Fasi180222_G12818.t1/Fasi180250_G07688.t1/Fasi171483_G13319.t1/Fasi180281_G09711.t1/Fasi181100_G10113.t1/Fasi180104_G10679.t1/Fasi180279_G10743.t1/Fasi170880_G13357.t1/Fasi170460_G13556.t1/Fasi160270_G10105.t1/Fasi194006_G13141.t1/Fasi180275_G11532.t1	38	Accessory groups	MF

GO:0000781	chromosome, telomeric region	11/253	55/4104	0.000414618	0.007336947	0.006548297	Fasi170880_G13549.t1/Fasi170908_G07187.t1/Fasi171330_G13341.t1/Fasi170867_G07532.t1/Fasi170464_G13385.t1/Fasi160280_G13160.t1/Fasi171330_G07612.t1/Fasi170991_G13151.t1/Fasi194008_G13260.t1/Fasi171330_G13468.t1/Fasi140027_G07584.t1	11	Accessory groups	CC
GO:0022857	transmembrane transporter activity	32/253	282/4104	0.000434367	0.007579101	0.006764422	Fasi171308_G00008.t1/Fasi170347_G05825.t1/Fasi181006_G06193.t1/Fasi171075_G07733.t1/Fasi171005_G07405.t1/Fasi180220_G04579.t1/Fasi170382_G04561.t1/Fasi180242_G07569.t1/Fasi140001_G04975.t1/Fasi170988_G13462.t1/Fasi180341_G05926.t1/Fasi170719_G06635.t1/Fasi180222_G06592.t1/Fasi140015_G07513.t1/Fasi171274_G12770.t1/Fasi180192_G10156.t1/Fasi171301_G07643.t1/Fasi180188_G01292.t1/Fasi180242_G12788.t1/Fasi170382_G07580.t1/Fasi180222_G12818.t1/Fasi170343_G13059.t1/Fasi180281_G09711.t1/Fasi181100_G10113.t1/Fasi170705_G10893.t1/Fasi180104_G10679.t1/Fasi180279_G10743.t1/Fasi170460_G13556.t1/Fasi171330_G13456.t1/Fasi160270_G10105.t1/Fasi194006_G13141.t1/Fasi180275_G11532.t1	32	Accessory groups	MF
GO:0002252	immune effector process	5/253	12/4104	0.000473647	0.008112828	0.007240779	Fasi171075_G02705.t1/Fasi170545_G09975.t1/Fasi180363_G04135.t1/Fasi180104_G10679.t1/Fasi180279_G10743.t1	5	Accessory groups	BP
GO:0018212	peptidyl-tyrosine modification	5/253	12/4104	0.000473647	0.008112828	0.007240779	Fasi170908_G13535.t1/Fasi171330_G07104.t1/Fasi170988_G13320.t1/Fasi171330_G13407.t1/Fasi140025_G13424.t1	5	Accessory groups	BP
GO:0098687	chromosomal region	21/253	156/4104	0.000487158	0.008268405	0.007379633	Fasi170880_G13549.t1/Fasi170908_G07187.t1/Fasi171330_G13341.t1/Fasi170867_G07532.t1/Fasi170464_G13385.t1/Fasi160280_G13160.t1/Fasi171330_G07612.t1/Fasi170991_G13151.t1/Fasi194008_G13260.t1/Fasi171330_G13468.t1/Fasi140027_G07584.t1/Fasi170988_G13513.t1/Fasi170460_G10586.t1/Fasi180222_G05219.t1/Fasi180242_G08398.t1/Fasi194008_G08244.t1/Fasi180269_G07857.t1/Fasi171643_G08222.t1/Fasi171206_G08196.t1/Fasi180341_G11247.t1/Fasi170464_G07649.t1	21	Accessory groups	CC
GO:0016831	carboxy-lyase activity	6/253	18/4104	0.00051152	0.00860368	0.007678869	Fasi181096_G03883.t1/Fasi140013_G08044.t1/Fasi160242_G10513.t1/Fasi170382_G07571.t1/Fasi171470_G07274.t1/Fasi180269_G07174.t1	6	Accessory groups	MF
GO:0071103	DNA conformation change	18/253	125/4104	0.000546658	0.009112601	0.008133085	Fasi170880_G13549.t1/Fasi170908_G07187.t1/Fasi171330_G13341.t1/Fasi170867_G07532.t1/Fasi170464_G13385.t1/Fasi160280_G13160.t1/Fasi171330_G07612.t1/Fasi170991_G13151.t1/Fasi194008_G13260.t1/Fasi171330_G13468.t1/Fasi140027_G07584.t1/Fa	18	Accessory groups	BP

							si171330_G00515.t1/Fasi170988_G13513.t1/Fasi180222_G05219.t1/Fasi140019_G09559.t1/Fasi180341_G11247.t1/Fasi160280_G00505.t1/Fasi171484_G13024.t1			
GO:0034599	cellular response to oxidative stress	19/253	137/4104	0.000622407	0.010283483	0.009178109	Fasi171005_G07405.t1/Fasi180220_G04579.t1/Fasi170382_G04561.t1/Fasi180242_G07569.t1/Fasi170908_G06273.t1/Fasi171274_G12770.t1/Fasi180192_G10156.t1/Fasi171301_G07643.t1/Fasi180188_G01292.t1/Fasi180242_G12788.t1/Fasi170382_G07580.t1/Fasi180222_G12818.t1/Fasi170665_G08985.t1/Fasi180267_G08932.t1/Fasi170908_G13535.t1/Fasi171330_G07104.t1/Fasi170988_G13320.t1/Fasi171330_G13407.t1/Fasi140025_G13424.t1	19	Accessory groups	BP
GO:0008233	peptidase activity	17/253	116/4104	0.000633391	0.010373163	0.00925815	Fasi170630_G00081.t1/Fasi170788_G01663.t1/Fasi171639_G01882.t1/Fasi140012_G02954.t1/Fasi170545_G09975.t1/Fasi180363_G04135.t1/Fasi181104_G10919.t1/Fasi171658_G10961.t1/Fasi170542_G13511.t1/Fasi170908_G13470.t1/Fasi171670_G13344.t1/Fasi171252_G13281.t1/Fasi160232_G12432.t1/Fasi170464_G13604.t1/Fasi170908_G13465.t1/Fasi140025_G13488.t1/Fasi170464_G11886.t1	17	Accessory groups	MF
GO:0030003	cellular cation homeostasis	21/253	160/4104	0.000687039	0.011153927	0.009954989	Fasi180101_G02972.t1/Fasi171075_G07733.t1/Fasi171005_G07405.t1/Fasi180220_G04579.t1/Fasi170382_G04561.t1/Fasi180242_G07569.t1/Fasi170988_G13462.t1/Fasi171025_G08213.t1/Fasi171274_G12770.t1/Fasi180192_G10156.t1/Fasi171301_G07643.t1/Fasi180188_G01292.t1/Fasi180242_G12788.t1/Fasi170382_G07580.t1/Fasi180222_G12818.t1/Fasi170784_G09614.t1/Fasi170705_G10893.t1/Fasi160270_G09528.t1/Fasi171205_G09498.t2/Fasi194006_G13141.t1/Fasi180275_G11532.t1	21	Accessory groups	BP
GO:0006955	immune response	5/253	13/4104	0.00073124	0.011769182	0.01050411	Fasi171075_G02705.t1/Fasi170545_G09975.t1/Fasi180363_G04135.t1/Fasi180104_G10679.t1/Fasi180279_G10743.t1	5	Accessory groups	BP
GO:0055080	cation homeostasis	21/253	163/4104	0.000880395	0.01404869	0.012538593	Fasi180101_G02972.t1/Fasi171075_G07733.t1/Fasi171005_G07405.t1/Fasi180220_G04579.t1/Fasi170382_G04561.t1/Fasi180242_G07569.t1/Fasi170988_G13462.t1/Fasi171025_G08213.t1/Fasi171274_G12770.t1/Fasi180192_G10156.t1/Fasi171301_G07643.t1/Fasi180188_G01292.t1/Fasi180242_G12788.t1/Fasi170382_G07580.t1/Fasi180222_G12818.t1/Fasi170784_G09614.t1/Fasi170705_G10893.t1/Fasi160270_G09528.t1/Fasi171205_G09498.t2/Fasi194006_G13141.t1/Fasi180275_G11532.t1	21	Accessory groups	BP

GO:0006469	negative regulation of protein kinase activity	7/253	27/4104	0.00095109	0.014921728	0.013317789	Fasi180243_G01125.t1/Fasi170665_G08985.t1/Fasi170908_G13535.t1/Fasi171330_G07104.t1/Fasi170988_G13320.t1/Fasi171330_G13407.t1/Fasi140025_G13424.t1	7	Accessory groups	BP
GO:0033673	negative regulation of kinase activity	7/253	27/4104	0.00095109	0.014921728	0.013317789	Fasi180243_G01125.t1/Fasi170665_G08985.t1/Fasi170908_G13535.t1/Fasi171330_G07104.t1/Fasi170988_G13320.t1/Fasi171330_G13407.t1/Fasi140025_G13424.t1	7	Accessory groups	BP
GO:0071901	negative regulation of protein serine/threonine kinase activity	6/253	20/4104	0.000961814	0.014964218	0.013355711	Fasi180243_G01125.t1/Fasi170908_G13535.t1/Fasi171330_G07104.t1/Fasi170988_G13320.t1/Fasi171330_G13407.t1/Fasi140025_G13424.t1	6	Accessory groups	BP
GO:0004175	endopeptidase activity	11/253	61/4104	0.00104147	0.015937902	0.014224733	Fasi170788_G01663.t1/Fasi171639_G01882.t1/Fasi170545_G09975.t1/Fasi180363_G04135.t1/Fasi181104_G10919.t1/Fasi170542_G13511.t1/Fasi170908_G13470.t1/Fasi171670_G13344.t1/Fasi171252_G13281.t1/Fasi160232_G12432.t1/Fasi170464_G11886.t1	11	Accessory groups	MF
GO:0015748	organophosphate ester transport	11/253	61/4104	0.00104147	0.015937902	0.014224733	Fasi171005_G07405.t1/Fasi180220_G04579.t1/Fasi170382_G04561.t1/Fasi180242_G07569.t1/Fasi171274_G12770.t1/Fasi180192_G10156.t1/Fasi171301_G07643.t1/Fasi180188_G01292.t1/Fasi180242_G12788.t1/Fasi170382_G07580.t1/Fasi180222_G12818.t1	11	Accessory groups	BP
GO:0000433	carbon catabolite repression of transcription from RNA polymerase II promoter by glucose	5/253	14/4104	0.001080764	0.016142285	0.014407147	Fasi170908_G13535.t1/Fasi171330_G07104.t1/Fasi170988_G13320.t1/Fasi171330_G13407.t1/Fasi140025_G13424.t1	5	Accessory groups	BP
GO:0048024	regulation of mRNA splicing, via spliceosome	5/253	14/4104	0.001080764	0.016142285	0.014407147	Fasi170908_G13535.t1/Fasi171330_G07104.t1/Fasi170988_G13320.t1/Fasi171330_G13407.t1/Fasi140025_G13424.t1	5	Accessory groups	BP
GO:0061987	negative regulation of transcription from RNA	5/253	14/4104	0.001080764	0.016142285	0.014407147	Fasi170908_G13535.t1/Fasi171330_G07104.t1/Fasi170988_G13320.t1/Fasi171330_G13407.t1/Fasi140025_G13424.t1	5	Accessory groups	BP

	polymerase II promoter by glucose									
GO:0006873	cellular ion homeostasis	21/253	169/4104	0.001411447	0.020587281	0.01837435	Fasi180101_G02972.t1/Fasi171075_G07733.t1/Fasi171005_G07405.t1/Fasi180220_G04579.t1/Fasi170382_G04561.t1/Fasi180242_G07569.t1/Fasi170988_G13462.t1/Fasi171025_G08213.t1/Fasi171274_G12770.t1/Fasi180192_G10156.t1/Fasi171301_G07643.t1/Fasi180188_G01292.t1/Fasi180242_G12788.t1/Fasi170382_G07580.t1/Fasi180222_G12818.t1/Fasi170784_G09614.t1/Fasi170705_G10893.t1/Fasi160270_G09528.t1/Fasi171205_G09498.t2/Fasi194006_G13141.t1/Fasi180275_G11532.t1	21	Accessory groups	BP
GO:0005215	transporter activity	34/253	328/4104	0.001486642	0.021515969	0.019203213	Fasi171308_G00008.t1/Fasi180250_G00011.t1/Fasi170347_G05825.t1/Fasi181006_G06193.t1/Fasi171075_G07733.t1/Fasi171005_G07405.t1/Fasi180220_G04579.t1/Fasi170382_G04561.t1/Fasi180242_G07569.t1/Fasi140001_G04975.t1/Fasi170988_G13462.t1/Fasi180341_G05926.t1/Fasi170719_G06635.t1/Fasi180222_G06592.t1/Fasi140015_G07513.t1/Fasi171274_G12770.t1/Fasi180192_G10156.t1/Fasi171301_G07643.t1/Fasi180188_G01292.t1/Fasi180242_G12788.t1/Fasi170382_G07580.t1/Fasi180222_G12818.t1/Fasi170343_G13059.t1/Fasi180281_G09711.t1/Fasi181100_G10113.t1/Fasi170705_G10893.t1/Fasi180104_G10679.t1/Fasi180279_G10743.t1/Fasi160242_G04131.t1/Fasi170460_G13556.t1/Fasi171330_G13456.t1/Fasi160270_G10105.t1/Fasi194006_G13141.t1/Fasi180275_G11532.t1	34	Accessory groups	MF
GO:0006974	cellular response to DNA damage stimulus	32/253	303/4104	0.001514473	0.021750156	0.019412227	Fasi170880_G13549.t1/Fasi170908_G07187.t1/Fasi171330_G13341.t1/Fasi170867_G07532.t1/Fasi170464_G13385.t1/Fasi160280_G13160.t1/Fasi171330_G07612.t1/Fasi170991_G13151.t1/Fasi194008_G13260.t1/Fasi171330_G13468.t1/Fasi140027_G07584.t1/Fasi171330_G00515.t1/Fasi170988_G13513.t1/Fasi140052_G00902.t1/Fasi170677_G10032.t1/Fasi171115_G03161.t1/Fasi194004_G03166.t1/Fasi171005_G07405.t1/Fasi180220_G04579.t1/Fasi170382_G04561.t1/Fasi180242_G07569.t1/Fasi180222_G05219.t1/Fasi171274_G12770.t1/Fasi180192_G10156.t1/Fasi171301_G07643.t1/Fasi180188_G01292.t1/Fasi180242_G12788.t1/Fasi170382_G07580.t1/Fasi180222_G12818.t1/Fasi140019_G09559.t1/Fasi180341_	32	Accessory groups	BP

							G11247.t1/Fasi160280_G00505.t1			
GO:0042537	benzene-containing compound metabolic process	5/253	15/4104	0.001540426	0.021787685	0.019445722	Fasi140013_G08044.t1/Fasi160242_G10513.t1/Fasi170382_G07571.t1/Fasi171470_G07274.t1/Fasi180269_G07174.t1	5	Accessory groups	BP
GO:1903313	positive regulation of mRNA metabolic process	5/253	15/4104	0.001540426	0.021787685	0.019445722	Fasi170908_G13535.t1/Fasi171330_G07104.t1/Fasi170988_G13320.t1/Fasi171330_G13407.t1/Fasi140025_G13424.t1	5	Accessory groups	BP
GO:0006808	regulation of nitrogen utilization	6/253	22/4104	0.001667789	0.023237037	0.020739283	Fasi171330_G13332.t1/Fasi174240_G13328.t1/Fasi174240_G13314.t1/Fasi171668_G13314.t1/Fasi181150_G09801.t1/Fasi170677_G13519.t1	6	Accessory groups	BP
GO:0046015	regulation of transcription by glucose	6/253	22/4104	0.001667789	0.023237037	0.020739283	Fasi170908_G13535.t1/Fasi171330_G07104.t1/Fasi170988_G13320.t1/Fasi171330_G13407.t1/Fasi140025_G13424.t1/Fasi170464_G07649.t1	6	Accessory groups	BP
GO:0071310	cellular response to organic substance	21/253	172/4104	0.001766935	0.024436062	0.021809425	Fasi170364_G01638.t1/Fasi171075_G02705.t1/Fasi171005_G07405.t1/Fasi180220_G04579.t1/Fasi170382_G04561.t1/Fasi180242_G07569.t1/Fasi171274_G12770.t1/Fasi180192_G10156.t1/Fasi171301_G07643.t1/Fasi180188_G01292.t1/Fasi180242_G12788.t1/Fasi170382_G07580.t1/Fasi180222_G12818.t1/Fasi180104_G08685.t1/Fasi170665_G08985.t1/Fasi180104_G10679.t1/Fasi180279_G10743.t1/Fasi181150_G09801.t1/Fasi170677_G13519.t1/Fasi170880_G13357.t1/Fasi170464_G07649.t1	21	Accessory groups	BP
GO:0016811	hydrolase activity, acting on carbon-nitrogen (but not peptide) bonds, in linear amides	8/253	38/4104	0.001807675	0.024815657	0.022148217	Fasi170988_G13513.t1/Fasi170944_G11293.t1/Fasi170460_G13465.t1/Fasi171330_G07667.t1/Fasi170705_G11415.t1/Fasi171476_G10186.t1/Fasi170728_G01944.t1/Fasi170460_G10561.t1	8	Accessory groups	MF
GO:0004386	helicase activity	13/253	85/4104	0.00184637	0.02516185	0.022457197	Fasi170880_G13549.t1/Fasi170908_G07187.t1/Fasi171330_G13341.t1/Fasi170867_G07532.t1/Fasi170464_G13385.t1/Fasi160280_G13160.t1/Fasi171330_G07612.t1/Fasi170991_G13151.t1/Fasi194008_G13260.t1/Fasi171330_G13468.t1/Fasi140027_G07584.t1/Fa	13	Accessory groups	MF

GO:0009066	aspartate family amino acid metabolic process	10/253	56/4104	0.001893731	0.025620255	0.022866328	si180250_G07688.t1/Fasi171483_G13319.t1 Fasi140024_G05774.t1/Fasi170908_G06273.t1/Fasi170460_G13465.t1/Fasi171330_G07667.t1/Fasi171476_G10186.t1/Fasi170728_G01944.t1/Fasi171668_G07580.t1/Fasi170464_G07649.t1/Fasi170460_G10561.t1/Fasi180279_G09113.t1	10	Accessory groups	BP
GO:0008094	ATP-dependent activity, acting on DNA	11/253	66/4104	0.002033376	0.027311607	0.024375877	Fasi170880_G13549.t1/Fasi170908_G07187.t1/Fasi171330_G13341.t1/Fasi170867_G07532.t1/Fasi170464_G13385.t1/Fasi160280_G13160.t1/Fasi171330_G07612.t1/Fasi170991_G13151.t1/Fasi194008_G13260.t1/Fasi171330_G13468.t1/Fasi140027_G07584.t1	11	Accessory groups	MF
GO:0002376	immune system process	5/253	16/4104	0.002129221	0.027798987	0.024810869	Fasi171075_G02705.t1/Fasi170545_G09975.t1/Fasi180363_G04135.t1/Fasi180104_G10679.t1/Fasi180279_G10743.t1	5	Accessory groups	BP
GO:0043484	regulation of RNA splicing	5/253	16/4104	0.002129221	0.027798987	0.024810869	Fasi170908_G13535.t1/Fasi171330_G07104.t1/Fasi170988_G13320.t1/Fasi171330_G13407.t1/Fasi140025_G13424.t1	5	Accessory groups	BP
GO:0045014	carbon catabolite repression of transcription by glucose	5/253	16/4104	0.002129221	0.027798987	0.024810869	Fasi170908_G13535.t1/Fasi171330_G07104.t1/Fasi170988_G13320.t1/Fasi171330_G13407.t1/Fasi140025_G13424.t1	5	Accessory groups	BP
GO:0061986	negative regulation of transcription by glucose	5/253	16/4104	0.002129221	0.027798987	0.024810869	Fasi170908_G13535.t1/Fasi171330_G07104.t1/Fasi170988_G13320.t1/Fasi171330_G13407.t1/Fasi140025_G13424.t1	5	Accessory groups	BP
GO:0008237	metallopeptidase activity	8/253	39/4104	0.002155419	0.02794561	0.02494173	Fasi170542_G13511.t1/Fasi170908_G13470.t1/Fasi171670_G13344.t1/Fasi171252_G13281.t1/Fasi170464_G13604.t1/Fasi170908_G13465.t1/Fasi140025_G13488.t1/Fasi170464_G11886.t1	8	Accessory groups	MF
GO:0007323	peptide pheromone maturation	4/253	10/4104	0.002203083	0.027980653	0.024973007	Fasi170542_G13511.t1/Fasi170908_G13470.t1/Fasi171670_G13344.t1/Fasi171252_G13281.t1	4	Accessory groups	BP
GO:0031930	mitochondria-nucleus signaling pathway	4/253	10/4104	0.002203083	0.027980653	0.024973007	Fasi171330_G13332.t1/Fasi174240_G13328.t1/Fasi174240_G13314.t1/Fasi171668_G13314.t1	4	Accessory groups	BP
GO:0050801	ion homeostasis	21/253	175/4104	0.002196086	0.027980653	0.024973007	Fasi180101_G02972.t1/Fasi171075_G07733.t1/Fasi171005_G07405.t1/Fasi180220_G04579.t1/Fasi170382_G04561.t1/Fasi180242_G07569.t1/Fasi170988_G13462.t1/Fasi171025_G08213.t1/Fasi171274_G12770.t1/Fasi180192_G10156.t1/Fasi171301_G07643.t1/Fa	21	Accessory groups	BP

							si180188_G01292.t1/Fasi180242_G12788.t1/Fasi170382_G07580.t1/Fasi180222_G12818.t1/Fasi170784_G09614.t1/Fasi170705_G10893.t1/Fasi160270_G09528.t1/Fasi171205_G09498.t2/Fasi194006_G13141.t1/Fasi180275_G11532.t1			
GO:0061984	catabolite repression	6/253	24/4104	0.002710721	0.033965884	0.030314884	Fasi170908_G13535.t1/Fasi171330_G07104.t1/Fasi170988_G13320.t1/Fasi170677_G13519.t1/Fasi171330_G13407.t1/Fasi140025_G13424.t1	6	Accessory groups	BP
GO:0005753	mitochondrial proton-transporting ATP synthase complex	5/253	17/4104	0.002866655	0.035210824	0.031426005	Fasi180104_G10679.t1/Fasi171663_G10692.t1/Fasi171645_G10724.t1/Fasi180279_G10743.t1/Fasi140006_G12678.t1	5	Accessory groups	CC
GO:0045259	proton-transporting ATP synthase complex	5/253	17/4104	0.002866655	0.035210824	0.031426005	Fasi180104_G10679.t1/Fasi171663_G10692.t1/Fasi171645_G10724.t1/Fasi180279_G10743.t1/Fasi140006_G12678.t1	5	Accessory groups	CC
GO:0048878	chemical homeostasis	23/253	204/4104	0.003114031	0.037999316	0.033914762	Fasi171075_G02705.t1/Fasi180101_G02972.t1/Fasi171075_G07733.t1/Fasi171005_G07405.t1/Fasi180220_G04579.t1/Fasi170382_G04561.t1/Fasi180242_G07569.t1/Fasi170988_G13462.t1/Fasi171025_G08213.t1/Fasi171274_G12770.t1/Fasi180192_G10156.t1/Fasi171301_G07643.t1/Fasi180188_G01292.t1/Fasi180242_G12788.t1/Fasi170382_G07580.t1/Fasi180222_G12818.t1/Fasi170784_G09614.t1/Fasi170705_G10893.t1/Fasi160270_G09528.t1/Fasi171205_G09498.t2/Fasi170464_G07649.t1/Fasi194006_G13141.t1/Fasi180275_G11532.t1	23	Accessory groups	BP
GO:0003677	DNA binding	34/253	343/4104	0.003167185	0.038396976	0.034269677	Fasi170880_G13549.t1/Fasi170908_G07187.t1/Fasi171330_G13341.t1/Fasi170867_G07532.t1/Fasi170464_G13385.t1/Fasi160280_G13160.t1/Fasi171330_G07612.t1/Fasi170991_G13151.t1/Fasi194008_G13260.t1/Fasi171330_G13468.t1/Fasi140027_G07584.t1/Fasi171330_G00515.t1/Fasi170364_G01638.t1/Fasi170677_G10032.t1/Fasi171115_G03161.t1/Fasi194004_G03166.t1/Fasi180222_G05219.t1/Fasi180219_G07541.t1/Fasi170430_G07497.t1/Fasi180242_G08398.t1/Fasi194008_G08244.t1/Fasi180269_G07857.t1/Fasi171643_G08222.t1/Fasi171206_G08196.t1/Fasi140019_G09559.t1/Fasi170382_G11119.t1/Fasi180341_G11247.t1/Fasi140001_G133	34	Accessory groups	MF

							34.t1/Fasi171287_G13324.t1/Fasi171670_G13334.t1/Fasi160280_G00505.t1/Fasi170677_G13519.t1/Fasi170464_G07649.t1/Fasi171669_G10431.t1			
GO:0001933	negative regulation of protein phosphorylation	7/253	33/4104	0.003328977	0.040098066	0.035787917	Fasi180243_G01125.t1/Fasi170665_G08985.t1/Fasi170908_G13535.t1/Fasi171330_G07104.t1/Fasi170988_G13320.t1/Fasi171330_G13407.t1/Fasi140025_G13424.t1	7	Accessory groups	BP
GO:0005775	vacuolar lumen	6/253	25/4104	0.003385907	0.040522363	0.036166606	Fasi170630_G00081.t1/Fasi171075_G02705.t1/Fasi171658_G10961.t1/Fasi170464_G13604.t1/Fasi170908_G13465.t1/Fasi140025_G13488.t1	6	Accessory groups	CC
GO:0055085	transmembrane transport	36/253	372/4104	0.003599239	0.04253025	0.037958665	Fasi171308_G00008.t1/Fasi180250_G00011.t1/Fasi170347_G05825.t1/Fasi181006_G06193.t1/Fasi171075_G07733.t1/Fasi171005_G07405.t1/Fasi180220_G04579.t1/Fasi170382_G04561.t1/Fasi180242_G07569.t1/Fasi140001_G04975.t1/Fasi170988_G13462.t1/Fasi180341_G05926.t1/Fasi170719_G06635.t1/Fasi180222_G06592.t1/Fasi140015_G07513.t1/Fasi171274_G12770.t1/Fasi180192_G10156.t1/Fasi171301_G07643.t1/Fasi180188_G01292.t1/Fasi180242_G12788.t1/Fasi170382_G07580.t1/Fasi180222_G12818.t1/Fasi170343_G13059.t1/Fasi180281_G09711.t1/Fasi181100_G10113.t1/Fasi170705_G10893.t1/Fasi180104_G10679.t1/Fasi171663_G10692.t1/Fasi171645_G10724.t1/Fasi180279_G10743.t1/Fasi140006_G12678.t1/Fasi170460_G13556.t1/Fasi171330_G13456.t1/Fasi160270_G10105.t1/Fasi194006_G13141.t1/Fasi180275_G11532.t1	36	Accessory groups	BP
GO:0000429	carbon catabolite regulation of transcription from RNA polymerase II promoter	7/253	34/4104	0.003976331	0.046398811	0.041411394	Fasi170908_G13535.t1/Fasi171330_G07104.t1/Fasi170988_G13320.t1/Fasi171330_G13407.t1/Fasi140025_G13424.t1/Fasi170464_G07649.t1/Fasi171669_G10431.t1	7	Accessory groups	BP
GO:0051348	negative regulation of transferase activity	7/253	34/4104	0.003976331	0.046398811	0.041411394	Fasi180243_G01125.t1/Fasi170665_G08985.t1/Fasi170908_G13535.t1/Fasi171330_G07104.t1/Fasi170988_G13320.t1/Fasi171330_G13407.t1/Fasi140025_G13424.t1	7	Accessory groups	BP

Table S4

Table S4 Sheet1 Significant homology groups of transcription factors were detected among the F. asiaticum pangenome using the production of 3ADON.

Homology group	p < 0.0001	Transcription factors (TF)	Chemotype
46871355	significant	TF	3ADON
46874044	significant	TF	3ADON
46875317	significant	TF	3ADON
46879262	significant	TF	3ADON
46879305	significant	TF	3ADON
46882291	significant	TF	3ADON
46885100	significant	TF	3ADON
46885127	significant	TF	3ADON
46885136	significant	TF	3ADON
46885149	significant	TF	3ADON
46885353	significant	TF	3ADON
46885834	significant	TF	3ADON
46886432	significant	TF	3ADON
46886539	significant	TF	3ADON

Table S4 Sheet2 Significant homology groups of transcription factors were detected among the F. asiaticum pangenome using the production of 15ADON.

Homology group	p < 0.0001	Transcription factors (TF)	Chemotype
46870522	significant	TF	15ADON
46879203	significant	TF	15ADON
46879247	significant	TF	15ADON
46883081	significant	TF	15ADON
46885100	significant	TF	15ADON
46885675	significant	TF	15ADON
46886432	significant	TF	15ADON

Table S4 Sheet3 Significant homology groups of transcription factors were detected among the F. asiaticum pangenome using the production of DON.

Homology group	p < 0.0001	Transcription factors (TF)	Chemotype
46879247	significant	TF	DON
46885353	significant	TF	DON
46885649	significant	TF	DON
46886432	significant	TF	DON
46887168	significant	TF	DON

Table S4 Sheet4 Significant homology groups of transcription factors were detected among the F. asiaticum pangenome using the production of NIV.

Homology group	p < 0.0001	Transcription factors (TF)	Chemotype
46871653	significant	TF	NIV
46879247	significant	TF	NIV

Table S5

Table S5 Sheet1 Summary of the effects of the SNPs by SnpEff

Effect	Impact	Count	Percent	Region
Upstream gene variant	Modifier	1070700	39.94%	Upstream
Downstream gene variant	Modifier	1069597	39.90%	Downstream
Intergenic region	Modifier	235914	8.80%	Intergenic
Synonymous variant	Low	135341	5.05%	Exon
Missense variant	Moderate	118920	4.44%	Missense
Intron variant	Modifier	37812	1.41%	Intron
Splice region variant	Low	8935	0.33%	Splice
Stop gained	High	2247	0.08%	Exon
Splice donor variant	High	398	0.02%	Splice
Splice acceptor variant	High	282	0.01%	Splice
Stop retained variant	Low	260	0.01%	Exon
Stop lost	High	207	0.01%	Exon
Start lost	High	147	0.01%	Exon
Initiator codon variant	Low	9	0.00%	Exon

Table S5 Sheet2 Summary of the effects of the Indels by SnpEff.

Effect	Impact	Count	Percent	Region
Upstream gene variant	Modifier	76803	41.08%	Upstream
Downstream gene variant	Modifier	73202	39.16%	Downstream
Intergenic region	Modifier	25387	13.58%	Intergenic
Intron variant	Modifier	3945	2.11%	Intron
Frameshift variant	High	3379	1.81%	Any
Disruptive inframe deletion	Moderate	1148	0.61%	Exon
Disruptive inframe insertion	Moderate	840	0.45%	Exon
Conservative inframe insertion	Moderate	769	0.41%	Exon
Splice region variant	Low	645	0.35%	Splice
Conservative inframe deletion	Moderate	567	0.30%	Exon
Stop gained	High	90	0.05%	Exon
Splice donor variant	High	48	0.03%	Splice
Splice acceptor variant	High	49	0.03%	Splice
Stop lost	High	38	0.02%	Exon
Start lost	High	27	0.01%	Exon
Exon loss variant	High	7	0.00%	Exon
Intragenic variant	Modifier	1	0.00%	Gene

Table S6 Optimal homology groups test on the BUSCO genes with protein sequences similarity from 95% to 25%.

Relaxation mode	Minimum sequence similarity	Homology groups	Core groups	Single copy orthologous groups	Unique groups	Correct groups(3698)	TRUE Positives	FALSE Positives	FALSE Negatives	Recall	Precision	F-score
D1	95	37074	8571	8438	10126	3432	904773	208	4935	0.99458	0.99977	0.99717
D2	85	26373	10114	9835	4854	3631	908879	563	829	0.99909	0.99938	0.99923
D3	75	23003	10507	10137	3645	3649	908950	787	758	0.99917	0.99913	0.99915
D4	65	21056	10691	10246	3038	3655	909409	1168	299	0.99967	0.99872	0.99919
D5	55	19754	10829	10277	2694	3649	909365	1095	343	0.99962	0.9988	0.99921
D6	45	18597	10946	10243	2376	3638	909468	1896	240	0.99974	0.99792	0.99883
D7	35	17742	10994	10130	2147	3626	909575	3055	133	0.99985	0.99665	0.99825
D8	25	16345	10453	9094	2010	3534	909683	41233	25	0.99997	0.95664	0.97783

Figure S1

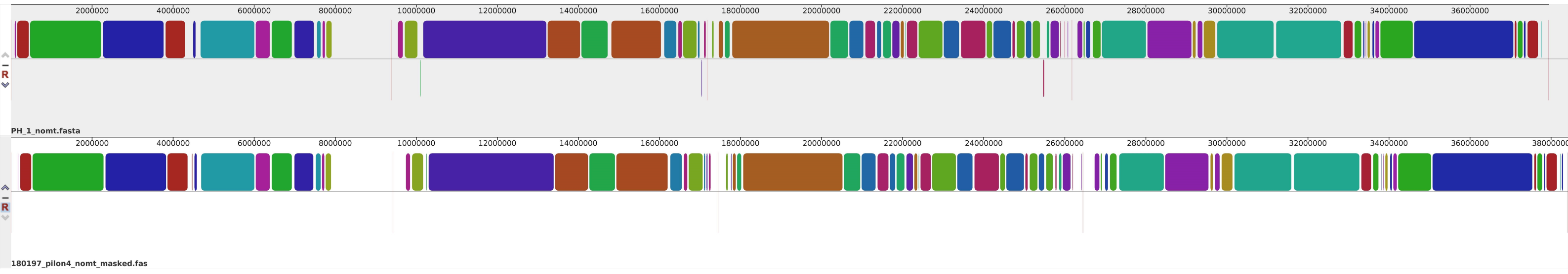
2000000
4000000
6000000
8000000
10000000
12000000
14000000
16000000
18000000
20000000
22000000
24000000
26000000
28000000
30000000
32000000
34000000
36000000
PH_1_nomt.fasta
180197_pilon4_nomt_masked.fas

Figure S2

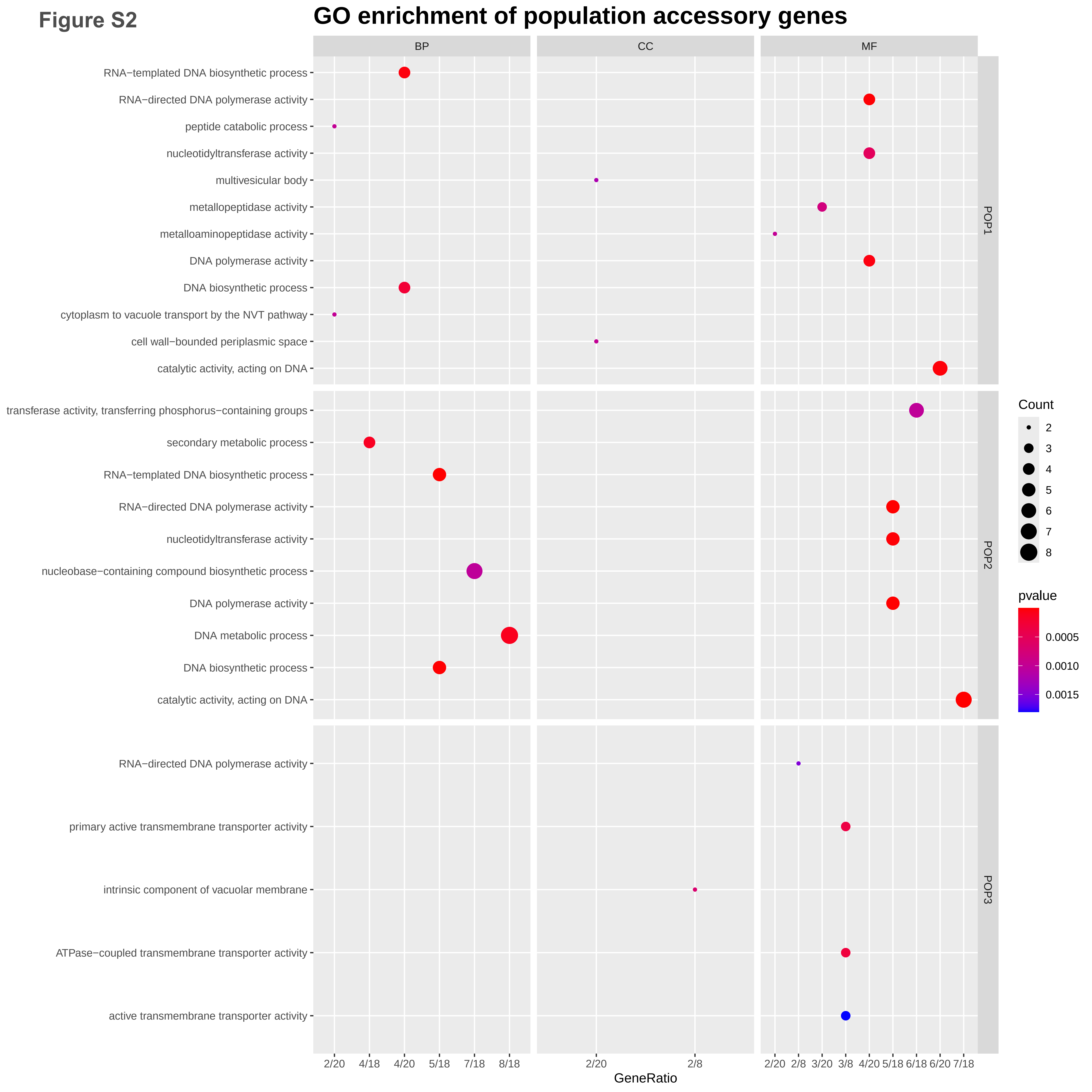

GO enrichment of population accessory genes
BP
CC
MF
POP1
POP2
POP3
RNA−templated DNA biosynthetic process
RNA−directed DNA polymerase activity
peptide catabolic process
nucleotidyltransferase activity
multivesicular body
metallopeptidase activity
metalloaminopeptidase activity
DNA polymerase activity
DNA biosynthetic process
cytoplasm to vacuole transport by the NVT pathway
cell wall−bounded periplasmic space
catalytic activity, acting on DNA
transferase activity, transferring phosphorus−containing groups
secondary metabolic process
nucleobase−containing compound biosynthetic process
DNA metabolic process
primary active transmembrane transporter activity
intrinsic component of vacuolar membrane
ATPase−coupled transmembrane transporter activity
active transmembrane transporter activity
2/20 4/18 4/20 5/18 7/18 8/18
2/20 2/8
2/20 2/8 3/20 3/8 4/20 5/18 6/18 6/20 7/18
GeneRatio
Count
2
3
4
5
6
7
8
pvalue
0.0005
0.0010
0.0015

Figure S3

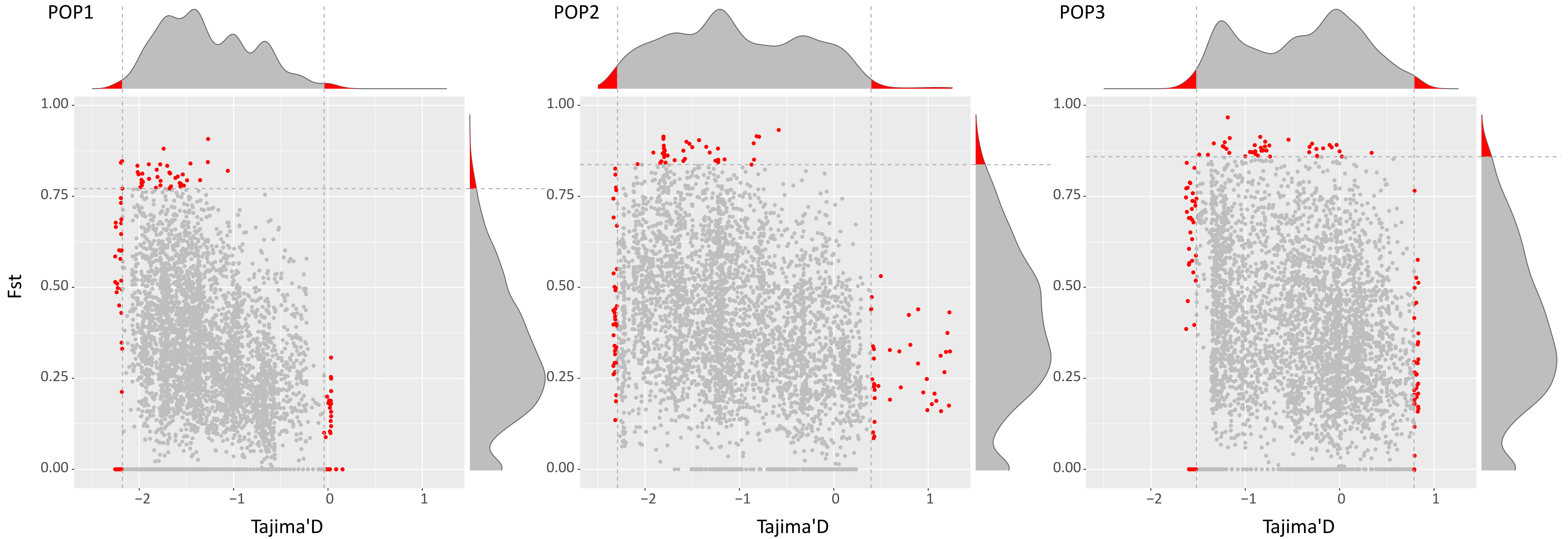

Figure S4

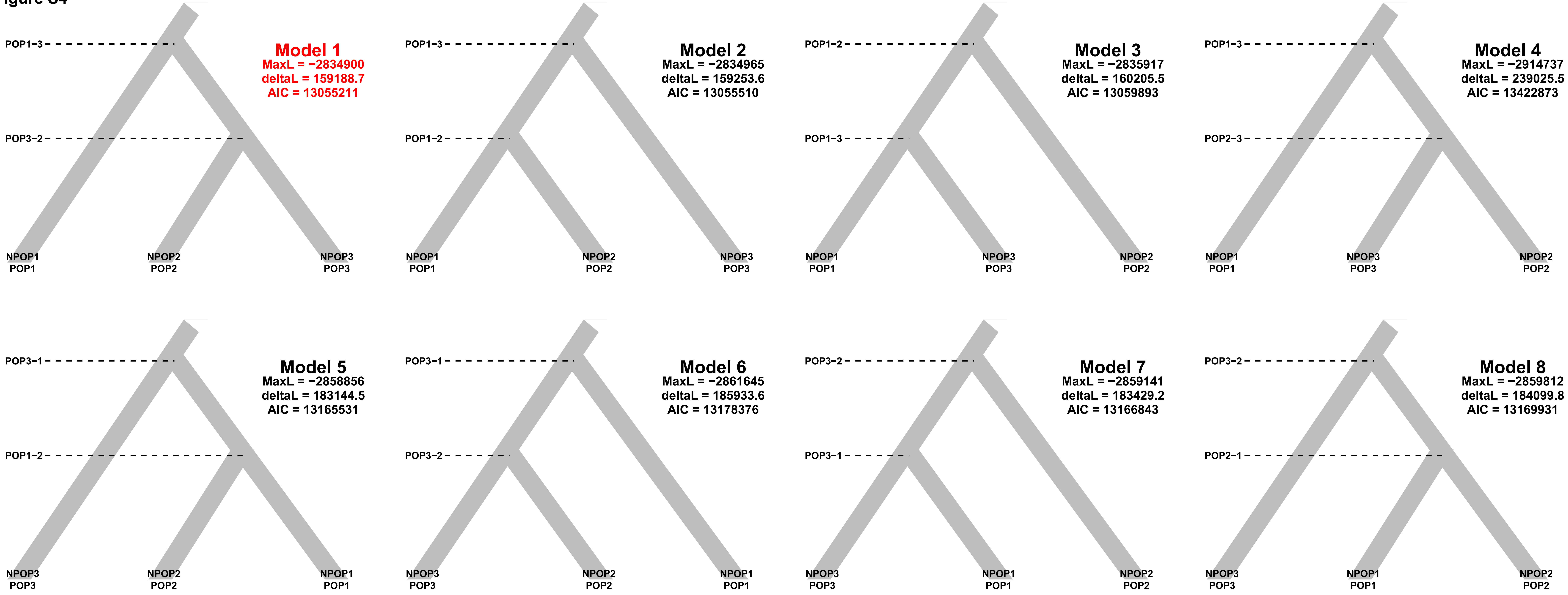
Model 1
MaxL = −2834900
deltaL = 159188.7
AIC = 13055211
POP1−3
POP3−2
NPOP1
POP1
NPOP2
POP2
NPOP3
POP3
Model 2
MaxL = −2834965
deltaL = 159253.6
AIC = 13055510
POP1−3
POP1−2
NPOP1
POP1
NPOP2
POP2
NPOP3
POP3
Model 3
MaxL = −2835917
deltaL = 160205.5
AIC = 13059893
POP1−2
POP1−3
NPOP1
POP1
NPOP3
POP3
NPOP2
POP2
Model 4
MaxL = −2914737
deltaL = 239025.5
AIC = 13422873
POP1−3
POP2−3
NPOP1
POP1
NPOP3
POP3
NPOP2
POP2
Model 5
MaxL = −2858856
deltaL = 183144.5
AIC = 13165531
POP3−1
POP1−2
NPOP3
POP3
NPOP2
POP2
NPOP1
POP1
Model 6
MaxL = −2861645
deltaL = 185933.6
AIC = 13178376
POP3−1
POP3−2
NPOP3
POP3
NPOP2
POP2
NPOP1
POP1
Model 7
MaxL = −2859141
deltaL = 183429.2
AIC = 13166843
POP3−2
POP3−1
NPOP3
POP3
NPOP1
POP1
NPOP2
POP2
Model 8
MaxL = −2859812
deltaL = 184099.8
AIC = 13169931
POP3−2
POP2−1
NPOP3
POP3
NPOP1
POP1
NPOP2
POP2

Figure S5

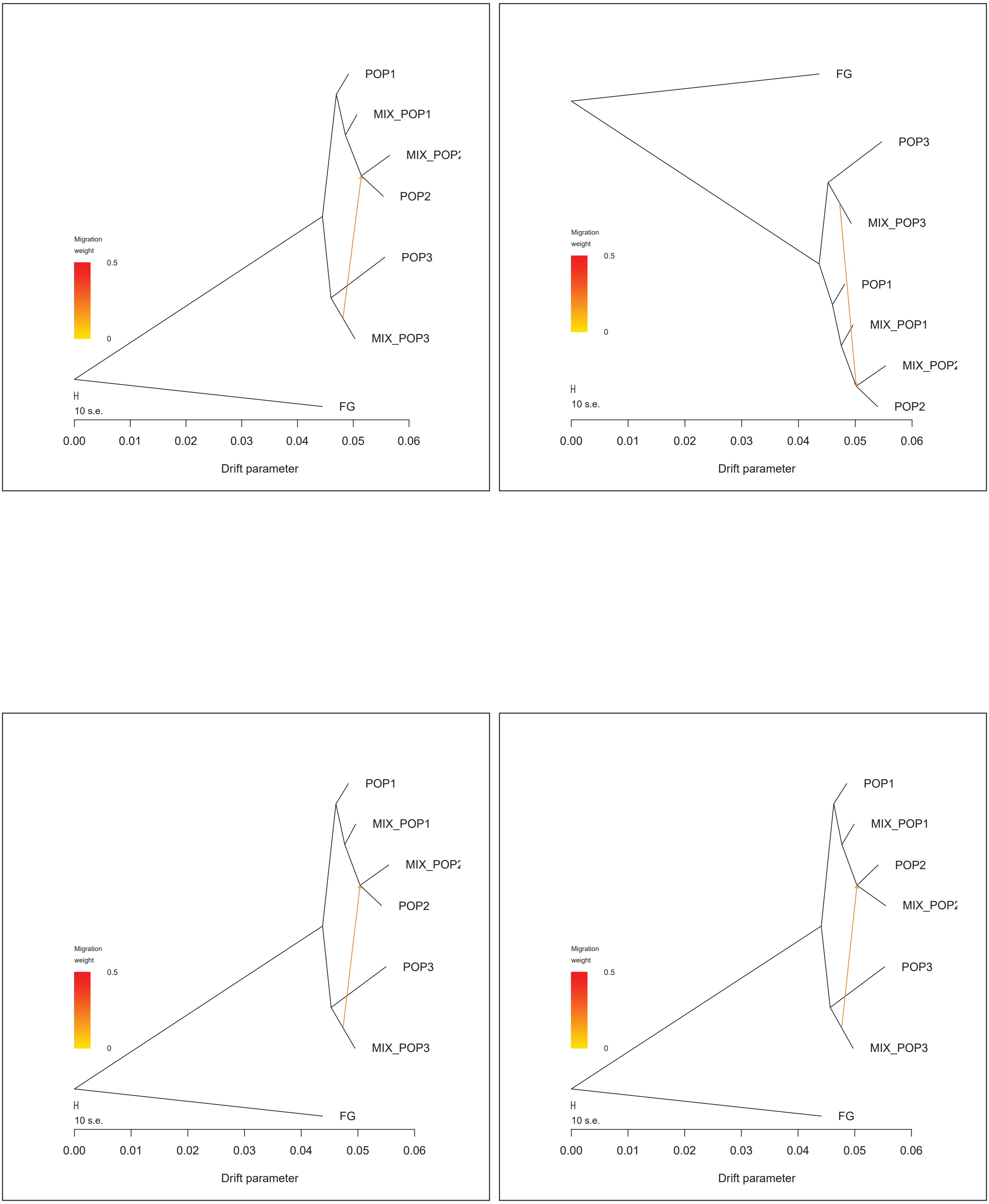
POP1
MIX_POP1
MIX_POP2
POP2
POP3
MIX_POP3
FG
Migration weight
0.5
0
10 s.e.
0.00
0.01
0.02
0.03
0.04
0.05
0.06
Drift parameter
FG
POP3
MIX_POP3
POP1
MIX_POP1
MIX_POP2
POP2
Migration weight
0.5
0
10 s.e.
0.00
0.01
0.02
0.03
0.04
0.05
0.06
Drift parameter
POP1
MIX_POP1
MIX_POP2
POP2
POP3
MIX_POP3
FG
Migration weight
0.5
0
10 s.e.
0.00
0.01
0.02
0.03
0.04
0.05
0.06
Drift parameter
POP1
MIX_POP1
POP2
MIX_POP2
POP3
MIX_POP3
FG
Migration weight
0.5
0
10 s.e.
0.00
0.01
0.02
0.03
0.04
0.05
0.06
Drift parameter

Figure S6

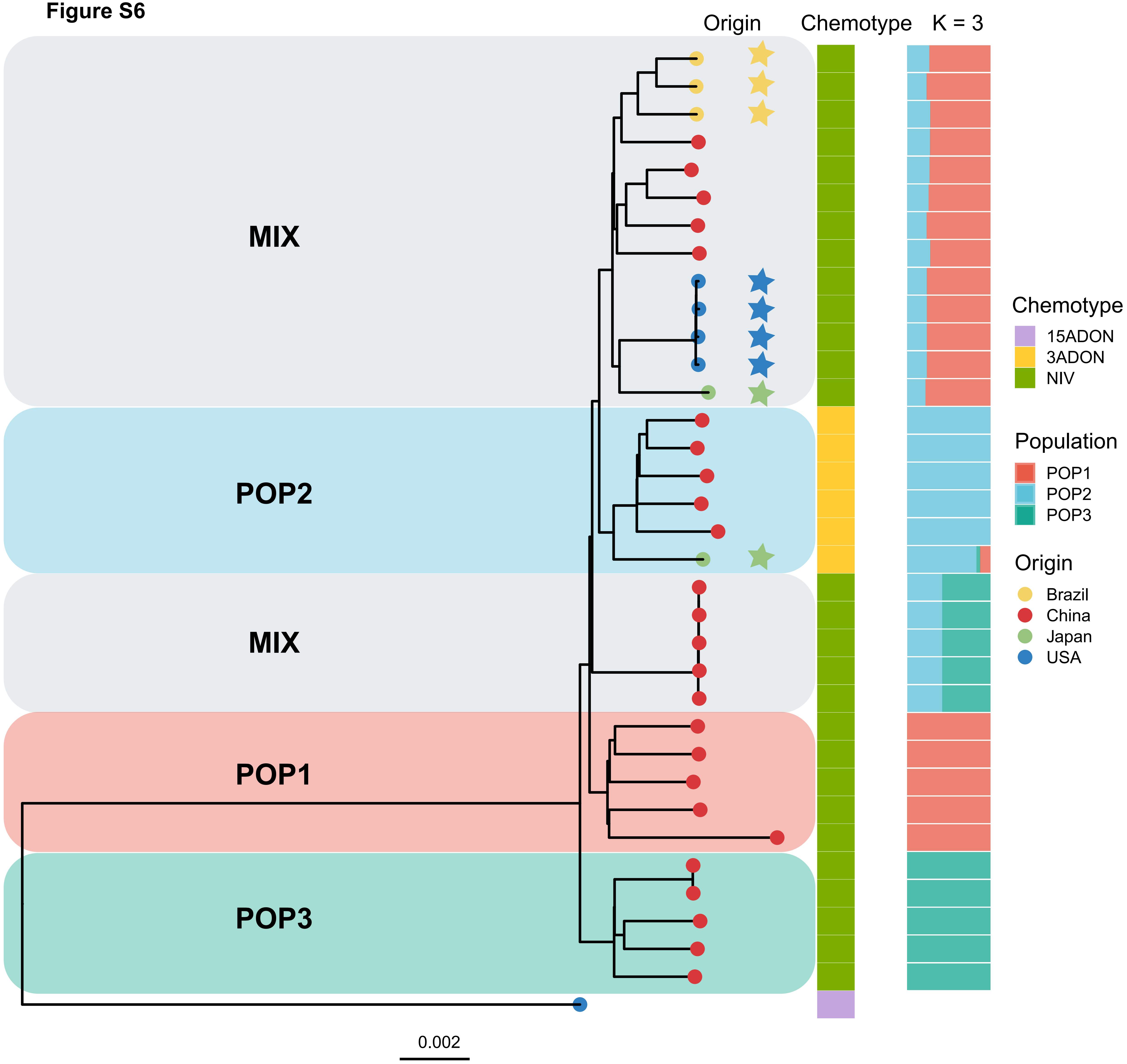
Origin
Chemotype
K = 3
MIX
POP2
MIX
POP1
POP3
0.002
Chemotype
15ADON
3ADON
NIV
Population
POP1
POP2
POP3
Origin
Brazil
China
Japan
USA

Figure S7

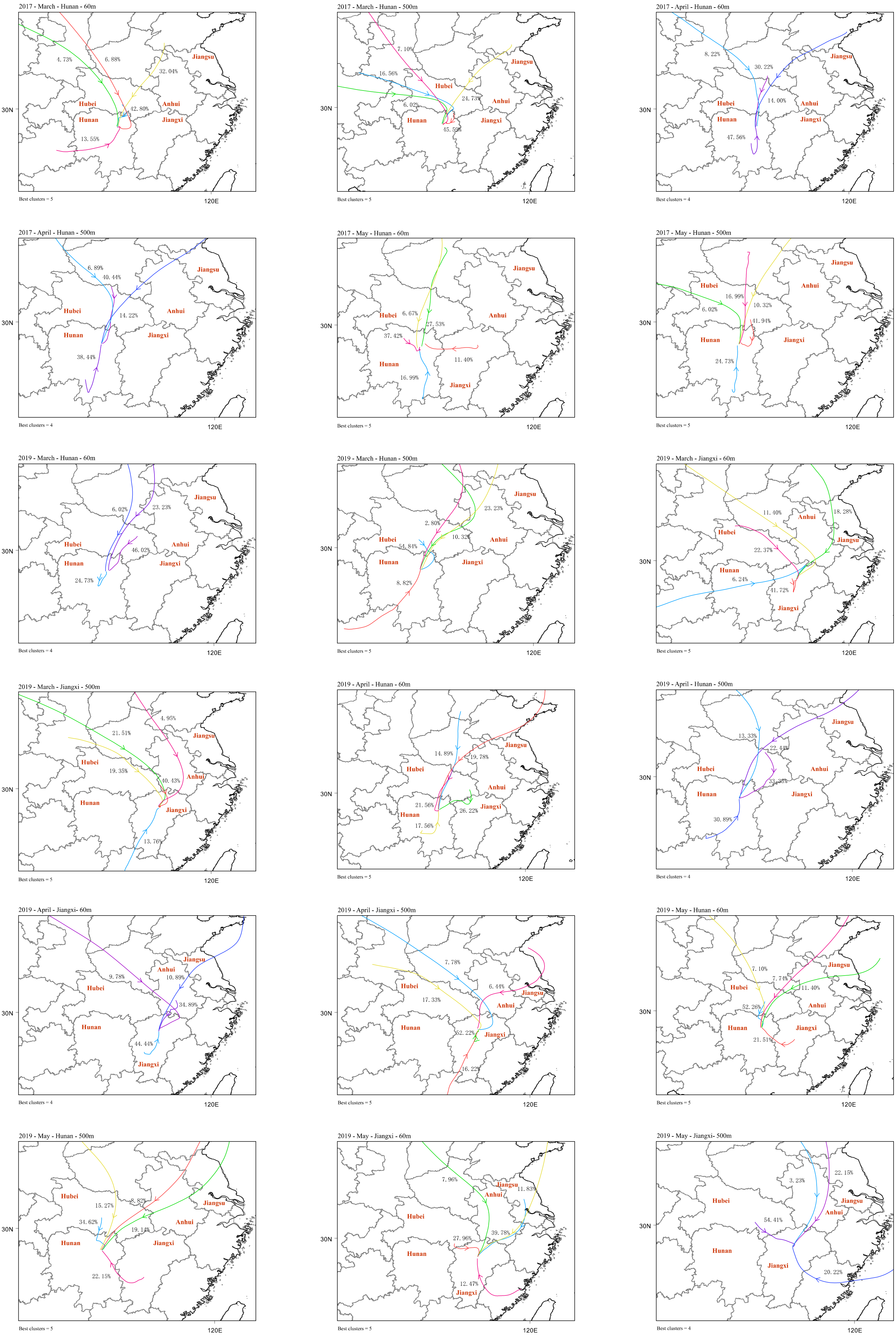
2017 - March - Hunan - 60m
Best clusters = 5
2017 - March - Hunan - 500m
Best clusters = 5
2017 - April - Hunan - 60m
Best clusters = 4
2017 - April - Hunan - 500m
Best clusters = 4
2017 - May - Hunan - 60m
Best clusters = 5
2017 - May - Hunan - 500m
Best clusters = 5
2019 - March - Hunan - 60m
Best clusters = 4
2019 - March - Hunan - 500m
Best clusters = 5
2019 - March - Jiangxi - 60m
Best clusters = 5
2019 - March - Jiangxi - 500m
Best clusters = 5
2019 - April - Hunan - 60m
Best clusters = 5
2019 - April - Hunan - 500m
Best clusters = 4
2019 - April - Jiangxi- 60m
Best clusters = 4
2019 - April - Jiangxi - 500m
Best clusters = 5
2019 - May - Hunan - 60m
Best clusters = 5
2019 - May - Hunan - 500m
Best clusters = 5
2019 - May - Jiangxi - 60m
Best clusters = 5
2019 - May - Jiangxi- 500m
Best clusters = 4

Figure and table captions

Figure S1: A syntenic analysis comparing the reference genomes of PH-1 (*F. graminearum*) and 180197 (*F. asiaticum*).

Figure S2: GO enrichment of accessory genes of three populations.

Figure S3: Genes under selection in each population were identified using a combination of Tajima's D, Fst, and HapFLK analyses.

Figure S4: Results of splits events inference with model schemes by fastsimcoal2. MaxL, deltaL, and AIC values are shown below the model number in bold letters, respectively.

Figure S5: The gene flow events were detected from POP3 to POP2.

Figure S6: The k-mer distance-based phylogenetic tree matched with the population structure results and chemotype information using global *F. asiaticum* strains.

Figure S7: Trajectory analyses of *F. asiaticum* dissemination routes to Hunan and Jiangxi.

Table S1: The collection information of all *F. asiaticum* strains in this study (Sheet 1) and 245 sequencing strains used for constructing the pangenome dataset (Sheet 2).

Table S2: Pangenome homology groups identified by PanTools.

Table S3: GO enrichment of pangenome accessory genes.

Table S4: Significant homology groups of transcription factors were detected among the *F. asiaticum* pangenome, using the production of 3ADON, 15ADON, DON, and NIV, respectively (Sheet 1-4). Significant homology groups of secreted proteins were detected among the *F. asiaticum* pangenome based on pathegenicity (Sheet 5).

Table S5: Summary of the effects of the SNPs and Indels by SnpEff.

Table S6: Optimal homology groups test on the BUSCO genes with protein sequences similarity from 95% to 25%.

Supplemental Text S1: Over 84% of the homology groups in the *F. asiaticum* pangenome belong to the core group.

Supplemental Text S2: Structural variation analysis of *F. asiaticum* pangenome.

Supplemental Text S3: Accessory homology groups associated with plant infection and

mycotoxin production.

Supplemental Text S4: The high diversity of SM gene clusters in *F. aisaticum* pangenome is reflected not only in presence/absence, absence rates, but also in the similarity of backbone genes sequence.

Supplemental Text S5: Identification of three distinct populations.
